# Long non-coding RNA dysregulation is a frequent event in non-small cell lung carcinoma pathogenesis

**DOI:** 10.1038/s41416-020-0742-9

**Published:** 2020-02-05

**Authors:** Amelia Acha-Sagredo, Bubaraye Uko, Paschalia Pantazi, Naiara G. Bediaga, Chryssanthi Moschandrea, Lucille Rainbow, Michael W. Marcus, Michael P. A. Davies, John K. Field, Triantafillos Liloglou

**Affiliations:** 1 Roy Castle Lung Cancer Programme, Department of Molecular & Clinical Cancer Medicine, University of Liverpool, Liverpool, UK; 2 Department of Surgery and Cancer, Institute of Reproductive and Developmental Biology (IRDB), Imperial College London, London, UK; 3 Centre for Genomic Research, Institute of Integrative Biology, University of Liverpool, Liverpool, UK

**Keywords:** Non-small-cell lung cancer, Long non-coding RNAs

## Abstract

**Background:**

Long non-coding RNAs compose an important level of epigenetic regulation in normal physiology and disease. Despite the plethora of publications of lncRNAs in human cancer, the landscape is still unclear.

**Methods:**

Microarray analysis in 44 NSCLC paired specimens was followed by qPCR-based validation in 29 (technical) and 38 (independent) tissue pairs. Cross-validation of the selected targets was achieved in 850 NSCLC tumours from TCGA datasets.

**Results:**

Twelve targets were successfully validated by qPCR (upregulated: FEZF1-AS1, LINC01214, LINC00673, PCAT6, NUTM2A-AS1, LINC01929; downregulated: PCAT19, FENDRR, SVIL-AS1, LANCL1-AS1, ADAMTS9-AS2 and LINC00968). All of them were successfully cross validated in the TCGA datasets. Abnormal DNA methylation was observed in the promoters of FENDRR, FEZF1-AS1 and SVIL-AS1. FEZF1-AS1 and LINC01929 were associated with survival in the TCGA set.

**Conclusions:**

Our study provides through multiple levels of internal and external validation, a comprehensive list of dysregulated lncRNAs in NSCLC. We therefore envisage this dataset to serve as an important source for the lung cancer research community assisting future investigations on the involvement of lncRNAs in the pathogenesis of the disease and providing novel biomarkers for diagnosis, prognosis and therapeutic stratification.

## Background

Long non-coding RNAs (lncRNAs) comprise a heterogeneous group of transcripts >200 nucleotides with no protein coding capacity. In relation to their topography of the closest protein-coding gene, lncRNAs have been broadly classified into: sense, antisense, bidirectional, intronic and intergenic lncRNAs.^1^ They are largely transcribed by RNA polymerase II, spliced, 5′ capped, polyadenylated and regulated at multiple levels similarly to the mRNAs.^2^ GENCODE release v29 reports 16,066 lncRNA transcripts.^3^ Although the function of the majority of lncRNAs is still largely unknown, evidence reveals their participation in many regulatory pathways,^4^ including chromatin organisation, transcriptional regulation, post-transcriptional and post-translational processing.^5^ These functions are exerted by acting as guides, tethers, decoys, scaffolds, competitive endogenous RNAs and miRNA sponges.^4^ In general, lncRNAs show lower expression levels, significantly shorter transcript half-lives and higher cell and tissue specificity than mRNAs.^1,6,7^

Dysregulation of lncRNA expression appears to be extensive in cancer, where they act either as oncogenes or tumour repressors.^8–10^ In the past few years, several pan-cancer transcriptomic analyses have been carried out after thorough realignment and reannotation of thousands of RNA-seq libraries retrieved from TCGA, providing an overview of lncRNA transcriptional alterations in different human cancers. These studies suggest that (a) lncRNAs are frequently dysregulated in tumours, (b) many of these alterations are highly tumour-specific and (c) they are often associated with other cancer-related (epi)genetic alterations.^9,10^

Lung cancer is the most frequent cause of cancer-related deaths, and non-small cell lung cancer accounts for the majority of the reported cases.^11^ Early screening and improved therapies are critical unmet clinical needs^12^ and the field of lncRNAs has attracted a lot of interest in an effort to identify novel diagnostic biomarkers and therapeutic targets. Analysis of publicly available RNA-seq data indicated 111 intergenic lung cancer-associated lncRNAs that were differentially expressed between tumour and adjacent normal lung tissue.^13^ Furthermore, following a pan-cancer analysis of RNA-seq data from seven additional TCGA solid tumour types, 58 of these were shown to be specific to lung cancer. To date, there are a number of additional studies analysing publicly available lncRNA expression data in lung cancer^14–16^ and fewer attempting validation in independent sample sets.^17–19^ However, despite the plethora of published papers, the lncRNA landscape in NSCLC is far from comprehensive, mainly because of the limited independent validation, inconsistency of targets and sample sets. In this study, we undertook a lncRNA transcriptome-wide microarray approach and performed both technical and biological validation of a number of highly differential expressed lncRNA targets. Qualified targets from this process were cross validated in TCGA datasets. Expression was also tested in a series of NSCLC cell lines. Finally, we investigated the DNA methylation status of those dysregulated lncRNAs bearing CpG islands in their promoters.

## Methods

### Patients and samples

A set of 82 NSCLC patients from the Liverpool Lung Project (LLP) were used for expression analysis. All patients were recruited, following voluntary informed consent, from Liverpool Heart and Chest Hospital, in accordance with the Declaration of Helsinki. Ethical approval was obtained from the Liverpool Central Research Ethics Committee (ref 97/141). Frozen tissues were macro-dissected to ensure >70% tumour content. For DNA methylation analysis, we utilised DNAs from 129 tumour and 36 normal samples available from previous studies.^20,21^ Demographic and clinical characteristics of the cancer patients are summarised in Supplementary Table S1.

### Analysis of the Cancer Genome Atlas expression data

Clinical and gene expression data was downloaded from the Genomics Data Commons (GDC) data portal using GDC Data Transfer Tool from the following The Cancer Genome Atlas (TCGA) datasets: TCGA-LUAD (Lung adenocarcinoma; *N* = 585) and TCGA-LUSC (Lung squamous cell carcinoma; *N* = 504). Patients with prior malignancies, subject to prior treatment and/or synchronous tumours were removed from our analysis. The main demographic and clinical characteristics of the TCGA patients analysed are summarised in Supplementary Table S2. Normalised gene expression values (FPKM-UQ; Fragments Per Kilobase of transcript per Million mapped reads upper quartile) were employed.

### Cell lines and growth conditions

Thirteen NSCLC cell lines (A549, Calu-1, Calu-3, Calu-6, COR-L23, DMS53, H358, H2073, HTB-59, HTB-182, LUDLU-1, SK-LU-1 and SK-MES-1) and a non-tumorigenic human foetal lung fibroblast cell line (IMR-90) were cultured in Dulbecco’s Modified Eagle’s Medium (DMEM)/Ham’s Nutrient Mixture F-12 (1: 1) containing 10% foetal bovine serum (Sigma-Aldrich, Gillingham, UK) at 37 °C and 5% CO_2_. All cell lines were mycoplasma tested (e-Myco™ plus Kit, iNtRON Biotechnology, Gyeonggi-do, Korea) and authenticated (GenePrint 10 System, Promega, Southampton, UK).

### RNA isolation

Total RNA was extracted from both tissue (10 × 10 μm thick) and cell lines using the Direct-zol mini kit (ZymoResearch, Irvine, CA, USA). RNA concentration and purity were determined on a NanoDrop ND-1000 spectrophotometer (Thermo Fisher Scientific, Hemel Hempstead, UK). RNA integrity was determined using the RNA 6000 Nano Lab-on-a-Chip kit on a Bioanalyzer 2100 (Agilent Technologies, Warrington, UK).

### lncRNA array profiling

lncRNA profiling was performed on 44 normal-tumour pairs, using an Agilent Custom 8×60K expression array (AMADID: 047718) designed by the GENCODE consortium based on the Gencode v15 catalogue of human lncRNA.

Sample labelling and array hybridisation were performed according to the Two-Color Microarray-Based Gene Expression Analysis—Low Input Quick Amp Labeling—protocol version 6.7 (Agilent Technologies, Warrington, UK). Tumour samples were labelled with Cy3 and Cy5 was used with normal tissue. The labelled cRNA was hybridised to the Agilent SurePrint G3 gene expression 8×60K microarray and scanned on an Agilent G2565CA microarray scanner at 100% R + G PMT, 3 μm resolution and 20 bit TIFF with no XDR. Agilent’s Feature Extraction 11.0.1.1 software was used to analyse acquired array images and extract intensity data. The microarray data have been deposited in the Gene Expression Omnibus (GEO) database (GSE130740).

### lncRNA RT–qPCR

Technical validation of the selected targets was performed in 29/44 array samples. Biological validation was undertaken on 38 independent NSCLC normal-tumour pairs. Additionally, the expression levels of the validated lncRNA targets were assessed in 14 cell lines. One microgram of total RNA was reverse transcribed (QuantiTect Reverse Transcription Kit, Qiagen, Crawley, UK) according to the manufacturer’s instructions. Quantitative real-time PCR was performed in triplicate using QuantiTect probe PCR mastermix (Qiagen, Crawley, UK). Primers and probes as well as the thermal profiles for each lncRNA qPCR assay are provided in Supplementary Table S3. lncRNA expression was quantified using the 2^−ΔΔCt^ method and TBP as endogenous control.

### DNA extraction and methylation analysis

DNA has been previously isolated.^20,21^ Pyrosequencing methylation analysis assays were designed for those lncRNAs harbouring a CpG island in their promoter (i.e. *FENDRR, FEZF1-AS1, LIN00673, NUTM2A-AS1, PCAT6, RNF139-AS1, SVIL-AS1*). One microgram of DNA was bisulphite-converted (EZ-96 DNA Methylation-Gold Kit, ZymoResearch, Irvine, CA, USA). DNA methylation analysis was performed by pyrosequencing (Qiagen, Crawley, UK) as previously described.^20^ Primers and PCR thermal profiles for each pyrosequencing assay are provided in Supplementary Table S4.

### Statistical analysis

#### Statistical analysis of the microarray

Raw data were corrected for background noise using the normexp method and normalised using the loess method in Limma.^22^ Differential expression analysis was carried out on non-control probes using linear models and robust empirical Bayes moderated *t*-statistics.^23^ We compared lung tumours to their matched normal tissue to identify NSCLC-specific lncRNA transcriptional signatures. *i*-values were adjusted to control the false discovery rate (FDR) below 5% using the Benjamini and Hochberg method. Differentially expressed genes were identified using the double-filtering criterion: adjusted p-value (FDR) < 0.01 and an absolute log2 fold-change (absolute log2 fold-change) > 0.5. All statistical analyses were performed with Bioconductor in the *R* statistical environment.^24^ For the visualisation of the 44 pairs of tissue (tumour and normal) data were subtracted for Cy3 and Cy5 separately after background correction, normalised using cyclicloess method, log2 transformed and plotted using the coolmap function in Limma.^22^

#### Validation of differentially expressed lncRNA by RT-qPCR

lncRNA targets showing the highest differential expression between tumour and normal tissues were selected for further biological validation. Wilcoxon signed rank tests were used to establish statistically significant changes between the paired tumours and adjacent tissues while Mann–Whitney *U* and Kruskal–Wallis H tests were used to assess possible associations between expression and clinicopathological features in tumours. ROC curves and AUC were calculated for all the validated targets following binary logistic regression analysis. For each lncRNA, the patient cohort was dichotomised into low and high expression subgroups using the relative quantity (RQ) median; patients’ survival was calculated using the log rank test and plotted using Kaplan-Meier curves.

#### DNA methylation analysis

Hypermethylation thresholds were calculated using the 95% reference interval (mean + 2 × SD) of the normal sample values. Differences between the methylation status (hypermethylated vs normal) of tumours and clinicopathological features were assessed using Chi-square and Fisher’s exact tests.

#### Analysis of differentially expressed lncRNAs in the TCGA dataset

Mann–Whitney U tests were used to establish statistically significant changes between the tumours and normal tissues. Associations between expression and clinicopathological features in tumours were assessed using Mann–Whitney *U* and Kruskal–Wallis H tests. Pairwise multiple comparisons were carried out using Dunn’s post hoc test and *p*-values were adjusted using the Bonferroni error correction. Patients’ survival was calculated as described above.

## Results

### Discovery of aberrant lncRNA expression profiles in NSCLC

On the Gencode v15 microarray, each of the 17,535 randomly selected protein coding and 22,001 lncRNA transcripts is targeted by two probes. Three samples that were analysed in duplicate and hybridised on separate arrays were used as technical replicates. Technical replicates showed a strong pairwise Pearson correlation coefficient (*r* ≥ 0.86, *p*-value < 10^–16^). An initial comparison between the lncRNA expression profiles of NSCLC tissue and adjacent normal tissue using unsupervised hierarchical clustering analysis showed that samples could be neatly classified based on the expression levels of antisense and lincRNAs (Supplementary Figs. S1C and S1B) as well as protein coding transcripts (Supplementary Fig. S1A). Gene expression analysis identified the following numbers of differentially expressed transcripts in the NSCLC vs normal comparison: 581 (down) and 368 (up) lincRNAs, 541 (down) and 403 (up) antisense RNAs as well as 1992 (up) and 1,448 (down) protein-coding RNAs, (Supplementary Table S5). In this study, we focused only in the validation of the lncRNAs (lincRNA and antisense RNA transcripts).

### Validation of differentially expressed lncRNAs

The selection criteria for the validation step included the *p*-value, fold-change and the clarity of annotation of the sequences. Regarding the latter, validation was performed only for targets where the probe sequence matched the location or Ensembl_ID indicated in the array manifest. For the validation step, we selected fourteen targets across a range of adjusted p-values (7.6 × 10^–3^ to 2.7 × 10^–23^) in order to assess the range of confidence in this microarray dataset.

The selected lncRNAs for technical and biological qPCR-based validation (Supplementary Table S5) were *FEZF1-AS1, LINC01214, LINC00673, PCAT6, NUTM2A-AS1, RNF139-AS1, LINC01929, HMGA1P4* (upregulated) and *PCAT19, FENDRR, SVIL-AS1, LANCL1-AS1, ADAMTS9-AS2 and LINC00968* (downregulated).

**Fig. 1:**
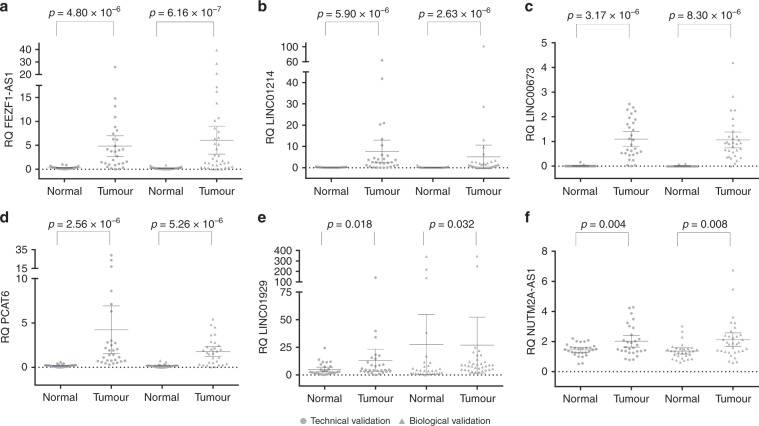
Overexpressed lncRNAs in NSCLC. Scatterplot diagrams demonstrating the expression levels of lncRNAs *FEZF1-AS1* (**a**), *LINC01214* (**b**), *LINC00673* (**c**), *PCAT6* (**d**), *LINC01929* (**e**) and *NUTM2A-AS1* (**f**), which are overexpressed in lung tumours compared to adjacent normal lung tissue. Means are also indicated along with the 95% confidence intervals. Technical validation values are given by circles, while biological independent validation values are given as triangles. *p*-values are derived from Wilcoxon ranked tests. RQ relative quantity.

**Fig. 2:**
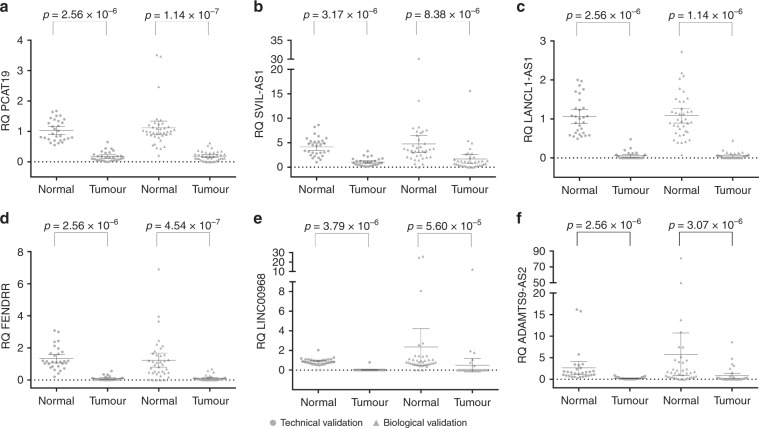
Downregulated lncRNAs in NSCLC. Scatterplot diagrams demonstrating the expression levels of lncRNAs *PCAT19* (**a**), *SVIL-AS1* (**b**), *LANCL1-AS1* (**c**), *FENDRR* (**d**), *LINC00968* (**e**) and *ADAMTS9-AS2* (**f**), which are downregulated in lung tumours compared to adjacent normal lung tissue. Means are also indicated along with the 95% confidence intervals. Technical validation values are given by circles, while biological independent validation values are given as triangles. *p*-values are derived from Wilcoxon ranked tests. RQ relative quantity.

Out of the eight over-expressed lncRNAs identified on the microarray, six were confirmed as upregulated in lung tumours compared with paired normal tissue both in the technical and biological sets (Fig. 1). *HMGA1P4* showed no significant difference in expression between tumour and normal paired samples in any of the validation sets (*p* > 0.05 in both), whereas *RNF139-AS1* showed insignificant expression difference in the technical validation set (*p* = 0.325) and borderline difference in the biological validation set (*p* = 0.03). All six selected under-expressed lncRNA targets were significantly differentially expressed in lung tumours compared with paired normal lung in both sets (Fig. 2).

In order to estimate the efficiency of each validated target to discriminate between normal and tumour tissue, binary logistic regression analysis was performed, and ROC curves were constructed (Supplementary Fig. S2). Expression of LANCL1-AS1 appeared to discriminate best (AUC = 0.995, 95% CI: 0.987–1), whilst assessment of NUTM2A-AS1 was the least informative (AUC = 0.689, 95% CI: 0.594–0.784).

### Correlation between clinical data and the expression of the validated lncRNAs

qPCR-based expression was analysed in relation to the epidemiological and clinicopathopathological parameters. No association with age or histology was observed, while a number of borderline associations between individual lncRNA expression and certain clinicopathological parameters were noticed (Table 1). Higher expression of *FEZF1-AS1* (*p* = 0.03) and *ADAMTS9-AS2* (*p* = 0.03) was observed in males compared to females. In addition, the expression of *LINC00968* (*p* = 0.03) was significantly higher in patients with lymph node involvement (N1/N2 stages when compared with N0), while the opposite relationship was found for *LINC01929* (*p* = 0.05). Finally, *PCAT6* (*p* = 0.02) and *LANCL1-AS1* (*p* = 0.01) expression was significantly lower in poorly differentiated tumours when compared to well and moderately differentiated ones.

**Table 1:** Correlation between clinical data and the expression of the validated lncRNAs.

		*N*	Relative quantity (RQ)		Mann–Whitney test	
			Mean	SD	*U*-score	*p*-value
*Gender*						
*FEZF1-AS1*	Male	49	6.49	8.31	268	0.03
	Female	17	2.72	3.68		
*ADAMTS9-AS2*	Male	50	0.66	1.51	255	0.03
	Female	16	0.11	0.16		
*Lymph node involvement*						
*LINC01929*	N(−)	33	31.63	74.00	339	0.05
	N(+)	29	8.00	7.39		
*LINC00968*	N(−)	34	0.20	0.50	435	0.03
	N(+)	32	0.38	2.18		
*Differentiation*						
*PCAT6*	Poor	18	2.03	3.85	212	0.02
	Well-Moderate	38	3.60	5.83		
*LANCL1-AS1*	Poor	20	0.02	0.05	266	0.01
	Well-Moderate	41	0.07	0.11		

*SD* standard deviation.

Only the expression of LINC01214 and FENDRR appeared to have an effect in survival (Supplementary Fig. S3). Longer survival was observed among the patients with high LINC01214 expression level (mean survival time: 73.24 months, 95% CI: 52.12–94.35) compared to low LINC01214 level (mean survival time: 41.14 months, 95% CI: 23.22–59.05, log rank *p* = 0.031). Likewise, patients with high FENDRR expression levels showed a better outcome (mean survival time: 75.29 months, 95% CI: 49.90–100.69) than those with low FENDRR expression levels (mean survival time: 43.71 months, 95% CI: 28.65–58.78, log rank *p* = 0.021).

### Cross-validation of selected targets with TCGA datasets

Out of the 14 lncRNAs selected for validation in our study, 13 were confirmed as being differentially expressed between tumour and normal tissue in the TCGA dataset (Supplementary Figs. S4 and S5). The only significant difference between our dataset and TCGA was for RNF139-AS1; only a borderline expression difference was seen in our biological validation set (*p* = 0.03), but it was significantly higher in tumours compared to normal tissues in the TCGA set (*p* = 1.27 × 10^–22^).

A number of significant associations were observed between tumour histology and the expression of the validated lncRNAs (Supplementary Fig. S6). Adenocarcinomas showed a significantly higher expression for PCAT19 (*p* = 4.14 × 10^–18^), FENDRR (*p* = 0.011), LANCL1-AS1 (*p* = 1.26 × 10^–4^), LINC00968 (*p* = 3.97 × 10^–12^) and ADAMTS9-AS2 (*p* = 1.92 × 10^–44^) when compared to squamous cell carcinomas. On the other hand, the expression of LINC00673 (*p* = 6.42 × 10^–30^), PCAT6 (*p* = 1.01 × 10^–7^), NUTM2A-AS1 (*p* = 3.69 × 10^–5^) and LINC01929 (*p* = 3.26 × 10^–6^) was significantly higher in squamous cell carcinomas than in adenocarcinomas.

Tumour stage significantly associated with the expression of LINC00673 (*p* = 0.005), LINC01929 (*p* = 0.002), PCAT19 (*p* = 0.001), FENDRR (*p* = 8.33 × 10^–6^), SVIL-AS1 (*p* = 0.037), LANCL1-AS1 (*p* = 8.69 × 10^–7^), LINC00968 (*p* = 2.32 × 10^–7^) and ADAMTS9-AS2 (8.29 × 10^–6^) as determined by Kruskal–Wallis test (Supplementary Fig. S7). Dunn’s post hoc pairwise test revealed that median expression level was significantly lower in T1 compared to T2 stage tumours for LINC00673 (*p* = 0.004) and LINC01929 (*p* = 0.001). SVIL-AS1 median expression was significantly lower in T1 compared to T3 NSCLC tumours (*p* = 0.027). On the other hand, for PCAT19, FENDRR, LANCL1-AS1, LINC00968 and ADAMTS9-AS2 the median expression level was significantly higher in T1 compared to T2 (*p*-values ≤ 0.001) and in T1 compared to T3 stage tumours (*p*-values ≤ 0.01) (Supplementary Fig. S7).

Regarding nodal stage (Supplementary Fig. S8), the expression of LINC00673 (*p* = 0.021) was significantly higher in patients with lymph node involvement (N1/N2 stages when compared with N0), while the opposite relationship was found for LANCL1-AS1 (*p* = 0.001), LINC00968 (*p* = 0.009) and ADAMTS9-AS2 (*p* = 0.003).

The expression of FEZF1-AS1 and LINC01929 appeared to be related to survival in the TCGA dataset (Supplementary Fig. S9). A trend for longer survival was observed among the patients with high FEZF1-AS1 level (mean survival time: 92.65 months, 95% CI: 74.46–110.84) compared to the low FEZF1-AS1 expression group (mean survival time: 76.25 months, 95% CI: 62.81–89.69, log rank *p* = 0.040). In addition, patients with low LINC01929 expression levels had better outcome (mean survival time: 95.38 months, 95% CI: 79.03–111.73) than those with high expression (mean survival time: 67.68 months, 95% CI: 59.63–75.73, log rank *p* = 0.022).

### Expression of the validated lncRNAs in NSCLC cell lines

**Fig. 3:**
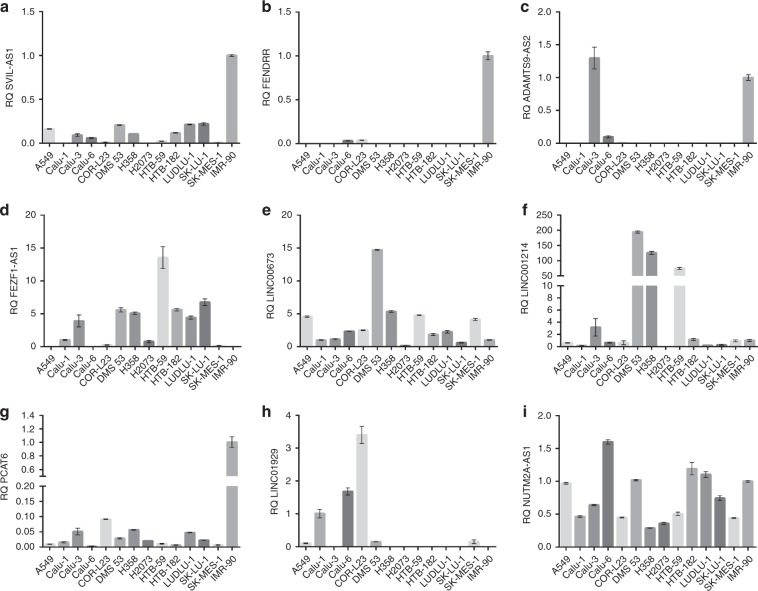
Bar chart diagrams showing the expression levels of the validated lncRNAs in NSCLC cell lines and a normal lung fibroblast cell line (IMR90), which was used as a calibrator (RQ = 1) for the analysis, with the exception of FEZF1-AS1 and LINC01929, which are not expressed in IMR90. In this case Calu-1 was used as calibrator. All downregulated lncRNAs in lung cancer tissue were reduced (*SVIL-AS1* (**a**), *FENDRR* (**b**), *ADAMTS9-AS2* (**c**)) or completely undetected (*PCAT19*, *LANCL1-AS1* and *LINC00968*, data not shown). Upregulated in lung cancer tissue lncRNAs did not necessarily follow the same pattern in cell lines. *FEZF1-AS1* (**d**), *LIN00673* (**e**) and *LINC01214* (**f**) were higher in NSCLC cell lines, *PCAT6* (**g**) and *LINC01929* (**h**) were lower in NSCLC cell lines compared to IMR90, while *NUTM2A-AS1* (**i**) was highly variable. Error bars represent standard error of the mean. RQ relative quantity.

The expression profiles of the 12 validated lncRNAs were also assessed in a panel of 13 lung cancer cell lines in comparison to a normal lung fibroblast line (IMR-90). Expression of the 6 downregulated targets in lung cancer tissue was significantly reduced (*SVIL-AS1, FENDRR, ADAMTS9-AS2*) or completely absent (*PCAT19, LANCL1-AS1 and LINC00968*) (Fig. 3) in lung cancer cell lines while positive controls (pooled normal lung tissue RNA) performed as expected in these experiments.

All the lncRNAs found upregulated in lung cancer tissue were widely expressed in the lung cancer cell lines analysed, however, not all of them followed the tissue pattern. When compared with the non-tumorigenic IMR-90, the expression of *FEZF1-AS1*, *LIN00673* and *LINC01214* was markedly higher in most lung cancer cell lines; the expression of *PCAT6* and *LINC01929* was strikingly lower and no clear trend was observed for the expression of *NUTM2A-AS1* (Fig. 3).

### Methylation of lncRNA loci in NSCLC patients

**Fig. 4:**
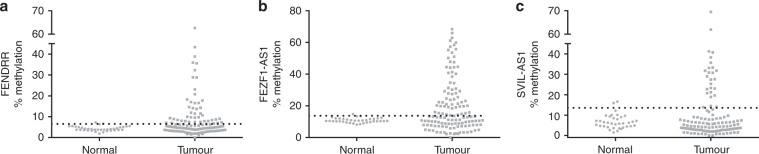
DNA methylation of lncRNA promoters in NSCLC. Scatterplot diagrams demonstrating the DNA methylation levels of lncRNAs *FENDRR* (**a**), *FEZF1-AS1* (**b**) and *SVIL-AS1* (**c**) in lung tumours compared to adjacent normal lung tissue. The horizontal dotted line in each diagram defines the 95% reference range (= mean + 2 × Standard deviation) of the normal tissues. Tumour samples with values over this line are classified as hypermethylated.

Only 7 out of the 14 lncRNAs selected from the microarray phase harbour a CpG island in their promoter. Pyrosequencing analysis demonstrated that *PCAT6*, *RNF139-AS1*, *LINC00673* and *NUTM2A-AS1* had <5% DNA methylation levels in both normal or tumour tissue, which is usually classified as noise.^25^ In contrast, variable methylation levels were observed for the remaining three targets. Hypermethylation in tumours (defined as > 95% reference range of normal values) was observed in 45/126 (35.7%) of tumours for *FENDRR*, 61/128 (47.7%) for *FEZF1-AS1* and 26/128 (20.3%) for *SVIL-AS1* (Fig. 4). DNA and corresponding RNA was available for 42 common samples between the two (expression and methylation analysis) sets. No direct correlation was established between RNA expression and hypermethylation status in any of these three genes.

### Association between clinical data and the methylation of the validated lncRNAs

*FENDRR* hypermethylation was more frequent in adenocarcinomas (56.9%) than in squamous cell carcinomas (21.6%, *p* = 5.5 × 10^–5^) while an opposite borderline trend was found for *FEZF1-AS1* (37.3% vs 55.3%, *p* = 0.05, Table 2). Non-significant trends were found between, *FEZF1-AS1* (*p* = 0.08) and *SVIL-AS1* (*p* = 0.06) hypermethylation and the differentiation status of the tumours, while no associations were obtained between hypermethylation of any promoter and age, gender and nodal stage.

**Table 2:** Association between clinical data and hypermethylation status of FENDRR and FEZF1-AS1.

		*N*	Hypermethylated (%)	Pearson *χ*^2^ test *p*-value
*Histology*				
*FENDRR*	AdenoCa	51	29 (56.9%)	**5.5** **×** **10**^**–5**^
	SqCCa	74	16 (21.6%)	
*FEZF1-AS1*	AdenoCa	51	19 (37.3%)	**0.05**
	SqCCa	76	42 (55.3%)	
*Differentiation*				
*FEZF1-AS1*	Poor	36	12 (34.3%)	0.08
	Well-Moderate	87	45 (51.7%)	
*SVIL-AS1*	Poor	35	11 (31.4%)	0.06
	Well-Moderate	87	14 (16.1%)	

*AdenoCa* adenocarcinoma, *SqCCa* squamous cell carcinoma.

Bold values indicate statistical significance *p*-values.

## Discussion

The discovery of long non-coding RNAs has revolutionised biological research, from basic gene function to clinical exploitation as disease specific biomarkers or therapeutic targets. The lncRNA transcriptome constitutes a whole new level of regulation in cell physiology. The promise of utilisation of lncRNAs in translational research and clinical management will rely on validation of differential gene expression levels associated with clinically relevant disease states and outcomes. Although there is still a lot to learn about lncRNA biology, there are already an increasing number of attempts to address lncRNA detection in body fluids to assist lung cancer diagnosis.^26^

The existing lncRNA profiling data in lung cancer to date is substantial. This includes high-throughput approaches^13,14,16–18^ and targeted studies.^27–31^ However, despite the plethora of reports, the landscape is less clear than one would hope. The main reason is the marked diversity of approaches and validation methods. In addition, with the exception of studies mining existing data in the public domain, most studies utilise limited discovery sample sets, which leads to high FDR.

In this study, we aimed to strengthen the existing data and contribute to a more comprehensive landscape of lncRNA dysregulation in NSCLC. We used a comparatively large discovery set in microarray analysis (44 pairs) and performed both technical (29 pairs) and independent biological (38 pairs) validation of targets by RT-qPCR, in order to identify lncRNA profiles significantly distinguishing NSCLCs tissue from paired normal lung tissue.

Following microarray data analysis and having set the desired thresholds (adjusted p-value (FDR) < 0.01 and absolute log2 fold-change > 0.5) we selected targets for validation with different *p*-values and fold-change values, in order to obtain a representative estimation of validity for our microarray analysis. An important selection factor was gene annotation and verification of probe sequences against locus sequence. For validation, we only selected among targets for which we had cross-referenced the hybridisation probe in the microarray with the annotated gene name in the databases (NCBI Genbank and ENSEMBL). Twelve out of the 14 targets selected for qPCR validation (six up- and six down-regulated in tumours) were successfully validated to demonstrate a strong and consistent difference of expression between NSCLC tissue and normal lung, suggesting a role in lung cancer pathogenesis. Interestingly, the two targets that failed in validation did not represent the highest *p*-values in the microarray analysis, indicating that even 10^–2^ adjusted *p*-values can be trusted. Surprisingly, a target with a very low p-value in microarray analysis (10^–19^) failed to validate, probably indicating potential problems with probe specificity and/or target annotation.

To the best of our knowledge, from the 12 validated lncRNAs, this is the first study where *LANCL1-AS1*, *LINC01214*, *LINC01929*, *NUTM2A-AS1*, *PCAT19* and *SVIL-AS1* are shown to be aberrantly expressed in cancer, while *ADAMTS9-AS2*, *FENDRR*, *FEZF1-AS1*, *LINC00673*, *LINC00968* and *PCAT6* have been previously reported to be dysregulated in lung and other types of cancer. Interestingly, our analysis of the available TCGA datasets demonstrated that all the 12 targets show significant differential expression in NSCLC, increasing further the confidence in this result. Apart from the validation of the RNF139-AS1, the main difference between our dataset and TCGA is centred around clinicopathological associations. A substantial number of associations of these lncRNA targets were found in TCGA, while we only found a few associations of borderline significance. This is not surprising, as TCGA datasets contained 850 tumours as opposed to 67 for our set used for clinicopathological comparisons. The clinicopathological associations revealed within the TCGA dataset point to future research in establishing the potential prognostic value of these targets. In addition, no major associations were found between lncRNA expression and overall survival, with the exception of a few statistical trends (0.02 < *p* < 0.05) for FEZF1-AS1 and LINC01929 in TCGA dataset and LINC01214 and FENDRR in our dataset. The differences in this finding probably reflects the size difference of the two datasets. Nevertheless, one has to consider that overall survival in lung cancer is a challenging parameter as the cause of death is frequently related to comorbidities and/or therapy related toxicity.

*ADAMTS9-AS2* was previously found dysregulated in several cancer types including NSCLC.^32,33^ Overexpression of *ADAMTS9-AS2* reduces proliferation, cell migration, invasion, and induces apoptosis in vitro while restrains tumour growth in vivo.^32–34^ Recent evidence suggests that *ADAMTS9-AS2* might exert its function by ‘sponging’ common microRNAs.^33,34^ For instance, in NSCLC, *ADAMTS9-AS2* represses cancer progression by regulating the miR-223-3p/TGFBR3 axis,^33^ while in ovarian cancer it regulates the miR-182-5p/FOXF2 pathway.^34^

A few studies have analysed the role of *FENDDR* in various cancer types suggesting that *FENDRR* plays an inhibitory role in tumour progression by regulating cell migration, invasion and metastasis.^35–37^ *FENDRR* was found to be downregulated in gastric cancer tissues, which increased cell migration and invasion in vitro through up-regulation of FN1 and MMP2 and MMP9.^35^ *FENDRR* expression was also shown to be suppressed in gastric cancer associated fibroblasts (CAFs) by hypermethylation.^38^ In NSCLC, we have observed that *FENDRR* expression is reduced in lung tumours compared with paired normal tissue and lung cancer cell lines showed a similar trend. This result is in agreement with a previous study reporting a significant downregulation of *FENDRR* in NSCLC as well as a potential involvement of *FENDRR* in regulating epithelial mesenchymal transition (EMT), stemness and metastasis of lung cancer cells via EZH2.^37^

Our finding on *FEZF1-AS1* upregulation in NSCLC is in agreement with previous reports on many tumour types including NSCLC^39,40^ and breast.^41^ *FEZF1-AS1* exerts regulatory functions at a transcriptional and post-transcriptional level, promoting cell proliferation, migration, and invasion. In fact, it has been reported to modulate the expression of Nanog by sponging miR-30a to promote cancer stemness in breast cancer.^41^ However, *FEZF1-AS1* can also act at a transcriptional level by recruiting chromatin-modifying enzymes to target genes. For instance, *FEZF1-AS1* can bind to LSD1 and EZH2 and epigenetically repress E-cadherin in NSCLC^39^ or p57 in lung adenocarcinomas.^40^

In our dataset, *LINC00673* was overexpressed in lung tumours, which is in agreement with previous reports on NSCLC.^42–44^ Upregulation of *LINC00673* has also been reported in gastric cancer,^45,46^ having a role in cell proliferation, migration, and invasion. *LINC00673* transcripts have been localised both in the nucleus and the cytoplasm,^42,44–46^ suggesting that it can function at a transcriptional and post-transcriptional level. In NSCLC, Lu *et al* proposed that *LINC00673* promotes TGF-β induced EMT by sponging miR-150-5p and thereby modulating ZEB1 expression indirectly.^43^ In addition, Roth *et al* have reported that *LINC00673* negatively regulates p53 translation by directly interacting with its 3′UTR leading to senescence bypass.^44^ *LINC00673* can also regulate the expression of target genes epigenetically. Notably, in lung cancer, *LINC00673* represses HOXA5 expression through recruiting the epigenetic repressor EZH2 to its promoter region.^47^ Shi *et al* observed that *LINC00673* epigenetically inhibited NCALD expression through direct binding to LSD1.^42^ In a similar way, in gastric cancer *LINC00673* suppressed KLF4 expression by interacting with EZH2 and DNMT1^46^ and direct binding to LSD1 and EZH2 repressed KLF2 and LATS2 expression.^45^

Regarding *PCAT6*, our findings are in line with previous studies reporting its upregulation in various cancer types including NSCLC^48^ and colon.^49^ Functional studies have shown that *PCAT6* exerts its oncogenic activity, at least partly, by leading chromatin-modifying complexes to specific promoter regions. For instance, in NSCLC, *PCAT6* interacts with EZH2 epigenetically, suppressing LATS2,^48^ while in colon cancer, the association of *PCAT6* with EZH2 modulates ARC expression.^49^

We observed that *LINC00968* was significantly downregulated in lung tumours compared with paired normal lung and completely absent in lung cancer cell lines, which is in contrast to a previous study reporting upregulation of *LINC00968* in NSCLC.^50^ However, that study evaluated *LINC00968* expression using datasets from the Oncomine database and four NSCLC cell lines, whereas we performed wet validation in 82 NSCLC tissue samples and 13 NSCLC cell lines. We speculate that inconsistency of results may lie with incorrect annotation in the previous bioinformatic analysis.

To explore the molecular mechanisms responsible for the modulated expression observed, we hypothesised that DNA methylation might be one of the mechanisms responsible. Therefore, we undertook DNA methylation analysis in seven of our target genes that were found to bear a CpG island in their promoter. Of those, hypermethylation was detected only in three, namely *FENDRR*, *SVIL-AS1* and *FEZF1-AS1*. There was a general lack of correlation between expression and DNA methylation in the genes tested. This may be due to the low number of common samples in the two analyses, but it most likely indicates the presence of strong alternative regulation factors besides DNA methylation. *FENDRR* hypermethylation has previously been shown in gastric CAFs.^38^ *FENDRR* promoter methylation demonstrated a significant increase in adenocarcinoma, however this was not the case for *FENDRR* expression.

This study contributes significantly to the wider knowledge of lncRNA involvement in NSCLC pathogenesis. Its strength lies with the technical and independent biological validation in patient tissues as well as its agreement with the available relevant TCGA datasets. Out of the 12 validated targets, half are shown to be differentially expressed in cancer for the first time, while our results for the other six lncRNAs confirm previous findings. Certainly, there are still a large number of dysregulated candidate lncRNAs in our microarray analysis list that require independent validation. Our data have been made publicly available, therefore open to other research groups to attempt independent validation. To date, the involvement of lncRNAs in NSCLC development is undisputable; however, further studies are needed to establish additional lncRNA targets and to identify their functional role in the pathogenesis of NSCLC.

## Supplementary information

Supplementary Information

## Data Availability

Microarray data have been deposited in the Gene Expression Omnibus (GEO) database (GSE130740).

## References

[1] Derrien T, Johnson R, Bussotti G, Tanzer A, Djebali S, Tilgner H (2012). The GENCODE v7 catalog of human long noncoding RNAs: analysis of their gene structure, evolution, and expression. Genome Res..

[2] Guttman M, Amit I, Garber M, French C, Lin MF, Feldser D (2009). Chromatin signature reveals over a thousand highly conserved large non-coding RNAs in mammals. Nature.

[3] Frankish A, Diekhans M, Ferreira AM, Johnson R, Jungreis I, Loveland J (2019). GENCODE reference annotation for the human and mouse genomes. Nucleic Acids Res..

[4] Yang G, Lu X, Yuan L (2014). LncRNA: a link between RNA and cancer. Biochim. Biophys. Acta.

[5] Ulitsky I, Bartel DP (2013). lincRNAs: genomics, evolution, and mechanisms. Cell.

[6] Cabili MN, Trapnell C, Goff L, Koziol M, Tazon-Vega B, Regev A (2011). Integrative annotation of human large intergenic noncoding RNAs reveals global properties and specific subclasses. Genes Dev..

[7] Clark MB, Johnston RL, Inostroza-Ponta M, Fox AH, Fortini E, Moscato P (2012). Genome-wide analysis of long noncoding RNA stability. Genome Res..

[8] Iyer MK, Niknafs YS, Malik R, Singhal U, Sahu A, Hosono Y (2015). The landscape of long noncoding RNAs in the human transcriptome. Nat. Genet.

[9] Yan X, Hu Z, Feng Y, Hu X, Yuan J, Zhao SD (2015). Comprehensive genomic characterization of long non-coding RNAs across human cancers. Cancer Cell.

[10] Ashouri A, Sayin VI, Van den Eynden J, Singh SX, Papagiannakopoulos T, Larsson E (2016). Pan-cancer transcriptomic analysis associates long non-coding RNAs with key mutational driver events. Nat. Commun..

[11] Herbst RS, Morgensztern D, Boshoff C (2018). The biology and management of non-small cell lung cancer. Nature.

[12] Field JK, Duffy SW, Devaraj A, Baldwin DR (2016). Implementation planning for lung cancer screening: five major challenges. Lancet Respir. Med..

[13] White NM, Cabanski CR, Silva-Fisher JM, Dang HX, Govindan R, Maher CA (2014). Transcriptome sequencing reveals altered long intergenic non-coding RNAs in lung cancer. Genome Biol..

[14] Yang J, Lin J, Liu T, Chen T, Pan S, Huang W (2014). Analysis of lncRNA expression profiles in non-small cell lung cancers (NSCLC) and their clinical subtypes. Lung Cancer.

[15] Zhao W, Luo J, Jiao S (2014). Comprehensive characterization of cancer subtype associated long non-coding RNAs and their clinical implications. Sci. Rep..

[16] Zhou M, Guo M, He D, Wang X, Cui Y, Yang H (2015). A potential signature of eight long non-coding RNAs predicts survival in patients with non-small cell lung cancer. J. Transl. Med..

[17] Yu H, Xu Q, Liu F, Ye X, Wang J, Meng X (2015). Identification and validation of long noncoding RNA biomarkers in human non-small-cell lung carcinomas. J. Thorac. Oncol..

[18] Feng N, Ching T, Wang Y, Liu B, Lin H, Shi O (2016). Analysis of microarray data on gene expression and methylation to identify long non-coding RNAs in non-small cell lung cancer. Sci. Rep..

[19] Cheng Z, Bai Y, Wang P, Wu Z, Zhou L, Zhong M (2017). Identification of long noncoding RNAs for the detection of early stage lung squamous cell carcinoma by microarray analysis. Oncotarget.

[20] Nikolaidis G, Raji OY, Markopoulou S, Gosney JR, Bryan J, Warburton C (2012). DNA methylation biomarkers offer improved diagnostic efficiency in lung cancer. Cancer Res..

[21] Bediaga NG, Davies MP, Acha-Sagredo A, Hyde R, Raji OY, Page R (2013). A microRNA-based prediction algorithm for diagnosis of non-small lung cell carcinoma in minimal biopsy material. Br. J. Cancer.

[22] Ritchie ME, Phipson B, Wu D, Hu Y, Law CW, Shi W (2015). limma powers differential expression analyses for RNA-sequencing and microarray studies. Nucleic Acids Res..

[23] Phipson B, Lee S, Majewski IJ, Alexander WS, Smyth GK (2016). Robust Hyperparameter estimation protects against hypervariable genes and improves power to detect differential expression. Ann. Appl. Stat..

[24] Gentleman RC, Carey VJ, Bates DM, Bolstad B, Dettling M, Dudoit S (2004). Bioconductor: open software development for computational biology and bioinformatics. Genome Biol..

[25] Shaw RJ, Liloglou T, Rogers SN, Brown JS, Vaughan ED, Lowe D (2006). Promoter methylation of P16, RARbeta, E-cadherin, cyclin A1 and cytoglobin in oral cancer: quantitative evaluation using pyrosequencing. Br. J. Cancer.

[26] Wan L, Zhang L, Fan K, Wang JJ (2017). Diagnostic significance of circulating long noncoding RNA PCAT6 in patients with non-small cell lung cancer. Onco Targets Ther..

[27] Nie W, Ge HJ, Yang XQ, Sun X, Huang H, Tao X (2016). LncRNA-UCA1 exerts oncogenic functions in non-small cell lung cancer by targeting miR-193a-3p. Cancer Lett..

[28] Shi X, Sun M, Liu H, Yao Y, Kong R, Chen F (2015). A critical role for the long non-coding RNA GAS5 in proliferation and apoptosis in non-small-cell lung cancer. Mol. Carcinog..

[29] Sun M, Liu XH, Wang KM, Nie FQ, Kong R, Yang JS (2014). Downregulation of BRAF activated non-coding RNA is associated with poor prognosis for non-small cell lung cancer and promotes metastasis by affecting epithelial-mesenchymal transition. Mol. Cancer.

[30] Han L, Zhang EB, Yin DD, Kong R, Xu TP, Chen WM (2015). Low expression of long noncoding RNA PANDAR predicts a poor prognosis of non-small cell lung cancer and affects cell apoptosis by regulating Bcl-2. Cell Death Dis..

[31] Nie FQ, Sun M, Yang JS, Xie M, Xu TP, Xia R (2015). Long noncoding RNA ANRIL promotes non-small cell lung cancer cell proliferation and inhibits apoptosis by silencing KLF2 and P21 expression. Mol. Cancer Ther..

[32] Cao B, Liu C, Yang G (2018). Down-regulation of lncRNA ADAMTS9-AS2 contributes to gastric cancer development via activation of PI3K/Akt pathway. Biomed. Pharmacother..

[33] Liu C, Yang Z, Deng Z, Zhou Y, Gong Q, Zhao R (2018). Upregulated lncRNA ADAMTS9-AS2 suppresses progression of lung cancer through inhibition of miR-223-3p and promotion of TGFBR3. IUBMB Life.

[34] Wang A, Jin C, Li H, Qin Q, Li L (2018). LncRNA ADAMTS9-AS2 regulates ovarian cancer progression by targeting miR-182-5p/FOXF2 signaling pathway. Int J. Biol. Macromol..

[35] Xu TP, Huang MD, Xia R, Liu XX, Sun M, Yin L (2014). Decreased expression of the long non-coding RNA FENDRR is associated with poor prognosis in gastric cancer and FENDRR regulates gastric cancer cell metastasis by affecting fibronectin1 expression. J. Hematol. Oncol..

[36] Kun-Peng Z, Chun-Lin Z, Xiao-Long M (2017). Antisense lncRNA FOXF1-AS1 Promotes Migration and Invasion of Osteosarcoma Cells Through the FOXF1/MMP-2/-9 Pathway. Int J. Biol. Sci..

[37] Miao L, Huang Z, Zengli Z, Li H, Chen Q, Yao C (2016). Loss of long noncoding RNA FOXF1-AS1 regulates epithelial-mesenchymal transition, stemness and metastasis of non-small cell lung cancer cells. Oncotarget.

[38] Najgebauer H, Liloglou T, Jithesh PV, Giger OT, Varro A, Sanderson CM (2019). Integrated omics profiling reveals novel patterns of epigenetic programming in cancer-associated myofibroblasts. Carcinogenesis.

[39] He R, Zhang FH, Shen N (2017). LncRNA FEZF1-AS1 enhances epithelial-mesenchymal transition (EMT) through suppressing E-cadherin and regulating WNT pathway in non-small cell lung cancer (NSCLC). Biomed. Pharmacother..

[40] Jin S, Chen S, Ma Y, Yang B, Liu Y (2017). LincRNA FEZF1-AS1 contributes to the proliferation of LAD cells by silencing p57 expression. Oncotarget.

[41] Zhang Z, Sun L, Zhang Y, Lu G, Li Y, Wei Z (2018). Long non-coding RNA FEZF1-AS1 promotes breast cancer stemness and tumorigenesis via targeting miR-30a/Nanog axis. J. Cell Physiol..

[42] Shi X, Ma C, Zhu Q, Yuan D, Sun M, Gu X (2016). Upregulation of long intergenic noncoding RNA 00673 promotes tumor proliferation via LSD1 interaction and repression of NCALD in non-small-cell lung cancer. Oncotarget.

[43] Lu W, Zhang H, Niu Y, Wu Y, Sun W, Li H (2017). Long non-coding RNA linc00673 regulated non-small cell lung cancer proliferation, migration, invasion and epithelial mesenchymal transition by sponging miR-150-5p. Mol. Cancer.

[44] Roth A, Boulay K, Gross M, Polycarpou-Schwarz M, Mallette FA, Regnier M (2018). Targeting LINC00673 expression triggers cellular senescence in lung cancer. RNA Biol..

[45] Huang M, Hou J, Wang Y, Xie M, Wei C, Nie F (2017). Long noncoding RNA LINC00673 is activated by SP1 and exerts oncogenic properties by interacting with LSD1 and EZH2 in gastric cancer. Mol. Ther..

[46] Ba MC, Long H, Cui SZ, Gong YF, Yan ZF, Wu YB (2017). Long noncoding RNA LINC00673 epigenetically suppresses KLF4 by interacting with EZH2 and DNMT1 in gastric cancer. Oncotarget.

[47] Ma C, Wu G, Zhu Q, Liu H, Yao Y, Yuan D (2017). Long intergenic noncoding RNA 00673 promotes non-small-cell lung cancer metastasis by binding with EZH2 and causing epigenetic silencing of HOXA5. Oncotarget.

[48] Shi X, Liu Z, Liu Z, Feng X, Hua F, Hu X (2018). Long noncoding RNA PCAT6 functions as an oncogene by binding to EZH2 and suppressing LATS2 in non-small-cell lung cancer. EBioMedicine.

[49] Huang W, Su G, Huang X, Zou A, Wu J, Yang Y (2019). Long noncoding RNA PCAT6 inhibits colon cancer cell apoptosis by regulating anti-apoptotic protein ARC expression via EZH2. Cell Cycle.

[50] Wang Y, Zhou J, Xu YJ, Hu HB (2018). Long non-coding RNA LINC00968 acts as oncogene in NSCLC by activating the Wnt signaling pathway. J. Cell Physiol..

