# Supplementary material for: Long non-coding RNA dysregulation is a frequent event in non-small cell lung carcinoma pathogenesis
Source: Br J Cancer. 2020 Feb 5;122(7):1050–8. doi: 10.1038/s41416-020-0742-9 (PMC7109049; doi:10.1038/s41416-020-0742-9)

Supplementary Table S1. Clinical characteristics of non-small cell lung carcinoma patients in the expression and DNA methylation analyses.

	Array set	Validation set	Total (expression set)	DNA Methylation set
N	44	38	82	129
**Age**				
mean (SD)	65.9 (9.7)	67.6 (8.4)	66.7 (9.1)	65.57 (9.14)
**Gender**				
Male : Female	31 : 13	29 : 9	60 : 22	88 : 41
**Histology**				
Adenocarcinoma	21	12	33	52
Squamous	23	24	47	76
Other	-	2	2	1
**Tumor stage**				
T1	2	4	6	10
T2	32	30	62	107
T3	8	3	11	10
T4	2	1	3	1
Missing				1
**Nodal stage**				
N0	22	19	41	70
N1	11	12	23	41
N2	11	7	18	16
Missing				2
**Differentiation**				
Well	4	4	8	10
Moderate	23	19	42	78
Poor	17	11	28	35
Missing		4	4	6

Supplementary Table S2. Clinical characteristics of non-small cell lung carcinoma patients in the TCGA expression dataset.

	**TCGA - Tumours**	**TCGA Non-Tumours**
N	850	87
**Age**		
mean (s.d.)	66.15 (9.47)	67.42 (9.99)
**Gender**		
Male : Female	511:339	49:38
**Histology**		
Adenocarcinoma	424	
Squamous	426	
**Tumour stage**		
T1	227	
T2	481	
T3	101	
T4	38	
Missing	3	
**Nodal stage**		
N0	541	
N1	191	
N2	96	
N3	7	
Missing	15	

Supplementary Table S3. Primer/probe sequences, amplicon size and annealing temperatures of qPCR assays used for lncRNA expression measurement in this study.

Locus	Primer sequence	Ta
FEZF1-AS1	Fwd: 5'-TGGCTATGGTTACTGCAATTC-3'	53°C
	Rev: 5'-CCATAAAGTCCAACCCTGAGT-3'	
	Probe: 5'-AAAAGGCCTGTGAGGTGTGTCCC-3'	
	Product size: 89 bp	
LINC01214	Fwd: 5'-GCATACTTCTGGTAGCAATGG-3'	54°C
	Rev: 5'-TAGGGATTATGTGTCTTCATTCTG-3'	
	Probe: 5'-ACCTCAAGTCCCCTTTGACCCG-3'	
	Product size: 82 bp	
HMGA1P4	Fwd: 5'-CACCCTCCTCCACTGTCCT-3'	54°C
	Rev: 5'-CCTCCTGCTTTGTTTCCTGT-3'	
	Probe: 5'-TCCCTGCACTAGGTCAGACAATCCC-3'	
	Product size: 84 bp	
PCAT6	Fwd: 5'-ACCCCACTTTCCAGCCTG-3'	55°C
	Rev: 5'-AGGGAGGCTCACGGACAC-3'	
	Probe: 5'-CCAGATCTGCAGCCTTCGCCC-3'	
	Product size: 115 bp	
LINC01929	Fwd: 5'-CTTGACACGACTTCAGAAGCCTC-3'	58°C
	Rev: 5'-GCAGAGCTCGACCAGGACAG-3'	
	Probe: 5'-TCTGGCCCATCGTGGCATGGT-3'	
	Product size: 118 bp	
LIN00673	Fwd: 5'-GAAAGGACAAGAAAGAGGATGG -3'	54°C
	Rev: 5'-AGAGGTGGTCCAGCCTGA-3'	
	Probe: 5'-TTCCCACCAGGAAGTTTAGCAGAACC-3'	
	Product size: 118 bp	
NUTM2A-AS1	Fwd: 5'-GGCTCATATGACATTAACAGACAA-3'	54°C
	Rev: 5'-TATCGCCTCCTGTACTATCAAAAT-3'	
	Probe: 5'-AAGACAGGCAACGTGTTGGACCTTC-3'	
	Product size: 138 bp	
RNF139-AS1	Fwd: 5'-GCGACTGAAGGGCAAGAAC-3'	58°C
	Rev: 5'-CCAACTTGTGTTTTAGATGAGTCCT-3'	
	Probe: 5'-CCCATAATGGCCTCTCTCCTTTTGCT-3'	
	Product size: 119 bp	
PCAT19	Fwd: 5'-TGTTATTTGGCTGGAGTGAGG-3'	60°C
	Rev: 5'-AATTCATTCCACTGTAAGCCTTC-3'	
	Probe: 5'-ATGAGTATCTCCAATGGTTCCCTGTTCTG-3'	
	Product size: 113 bp	
SVIL-AS1	Fwd: 5'-ACCTTTGATCCAGAACTTGCAG-3'	59°C
	Rev: 5'-CTAAGGGGTGGCTGCATTC-3'	
	Probe: 5'-TCTTCGGTTGTGAATCCGGCCC-3'	
	Product size: 78 bp	

LANCL1-AS1	Fwd: 5'-GTGAAAGTATTCTCTGACTGCAA-3'	55°C
	Rev: 5'-TGCATTGGCCAGAACATA-3'	
	Probe: 5'-TGACCACCTGTCTTTCTATATCAGAACCC-3'	
	Product size: 128 bp	
FENDRR	Fwd: 5'-GCTTCTGTCCAAGGCACT-3'	55°C
	Rev: 5'-CAAGCTTGCTAACTTCTTTGC-3'	
	Probe: 5'-AGCCTACTCGTCAAAAGCCCGA-3'	
	Product size: 111 bp	
LINC00968	Fwd: 5'-CTACAGCAAGGCAACTTATCTCAC-3'	57°C
	Rev: 5'-TGGGAGGGAAGGATGACAA-3'	
	Probe: 5'-TCACCAAGATATTCTGCACTTTCAGTGGC-3'	
	Product size: 124 bp	
ADAMTS9-AS2	Fwd: 5'-TCGGCAGTTTCCATTACC-3'	58°C
	Rev: 5'-TTAGATCAGTGGAATTAGTGAACA-3'	
	Probe: 5'-GAACTCATGGAATTTTCAGGCTTCA-3'	
	Product size: 149 bp	
TBP	Fwd: 5’-GGGGAGCTGTGATGTGAAGTTT-3'	53°-60°C
	Rev: 5'-AAACCAGGAAATAACTCTGGCTCA-3'	
	Probe: 5'-AAGGCCTTGTGCTCACCCACCAAC-3'	
	Product size: 96 bp	

The probes for all the lncRNA assays were labelled with FAM at the 5’ and BHQ2 at the 3’ end. The probe for the TBP assay was labelled with TAMRA at the 5’ and BHQ2 at the 3’ end.

For most of the lncRNAs, thermal profiles were as follows: 95°C for 15 min followed by 50 cycles of 94°C for 15 sec, Ta for 20 sec and 60°C for 45 sec.

For ADAMTS9-AS2 the thermal profile was: 95°C for 15 min followed by 50 cycles of 94°C for 15 sec, 58°C for 40 sec and 60°C for 40 sec.

While for RNF139-AS1 the thermal profile was: 95°C for 15 min followed by 50 cycles of 94°C for 60 sec, 58°C for 20 sec and 60°C for 45 sec.

Supplementary Table S4. Primer/probe sequences, amplicon size and annealing temperatures of pyrosequencing assays in this study.

Locus	Primer sequence	Ta
FEZF1-AS1	Fwd: 5’-TTTTGGGTTTGGTATTAGGA-3’	54°C
	Rev: 5’-BIO-CCCAAACTCAACTACAACATT-3’	
	Seq: 5’-GGGTTTGGTATTAGGAG-3’	
	PCR product: 111 bp	
LIN00673	Fwd: 5’-TATATAAGGGTAGAATGGTTTAGT-3’	51°C
	Rev: 5’-BIO-CAAAACCACAAATACCAAAAAC-3’	
	Seq: 5’-GTTTAGTAGATTGTAGGG-3’	
	PCR product: 113 bp	
NUTM2A-AS1	Fwd: 5’-GGAGGAAGAATGTAGGGAGA-3’	57°C
	Rev: 5’-BIO-AAACCAACACCAACCAATAA-3’	
	Seq: 5’-GGGAGATTTTTAGTGGAT-3’	
	PCR product: 145 bp	
PCAT6	Fwd: 5’-TTAGTTAAGGGAGTTGATTGGT-3’	53°C
	Rev: 5’-BIO-CAAACTAAAATTACCCAAATCC-3’	
	Seq: 5’-GTTGATTGGTAGGTAGT-3’	
	PCR product: 126 bp	
RNF139-AS1	Fwd: 5’-BIO-TATTTTAGGGAGTTTGAAAGT-3’	52°C
	Rev: 5’-ACCACACCCAACCTAATA-3’	
	Seq: 5’-CCACACCCAACCTAATACC-3’	
	PCR product: 126 bp	
FENDRR	Fwd: 5’-BIO-TGTTTTTAGGAATTTGGTTTG-3’	53°C
	Rev: 5’-CCTTAACCTAATACCCCTATAAACTC-3’	
	Seq: 5’-ACCTAATACCCCTATAAACT-3’	
	PCR product: 84 bp	
SVIL-AS1	Fwd: 5’-TGGTGTTAGTAAAGATATATAAGGAA-3’	55°C
	Rev: 5’-BIO-CCCTCTCAACTATTCAACAAC-3’	
	Seq: 5’-GTTAGTAAAGATATATAAGGAA-3’	
	PCR product: 121 bp	

Thermal profiles were as follows: 95°C for 15 min followed by 40 cycles of 94°C for 30 sec, Ta for 30 sec and 72°C for 30 sec and a final extension step at 72°C for 10 min.

Supplementary Table S5. Differentially expressed probes in the microarray.

ProbeName	GeneName	logFC	AveExpr	P.Value	adj.P.Val	transcript_type
A_33_P3258362	HBA2	-4.94	13.90	7.79E-31	3.37E-26	protein_coding
A_23_P4096	CA4	-6.21	7.91	1.10E-30	3.37E-26	protein_coding
A_23_P69497	CLEC3B	-6.83	12.45	3.91E-30	8.01E-26	protein_coding
A_33_P3243429	GPR152	-5.42	11.30	7.80E-30	1.16E-25	protein_coding
A_23_P95213	SFTPC	-9.30	15.77	9.41E-30	1.16E-25	protein_coding
A_23_P81280	BTNL9	-3.20	6.17	2.39E-29	2.45E-25	protein_coding
A_33_P3396008	AGER	-5.72	9.21	3.89E-29	3.42E-25	protein_coding
A_23_P130194	PYCR1	5.04	11.45	5.23E-29	4.02E-25	protein_coding
A_24_P53778	ITLN2	-7.03	8.76	8.65E-29	5.82E-25	protein_coding
A_23_P55544	CCBE1	-3.81	6.38	9.46E-29	5.82E-25	protein_coding
A_33_P3308347	ADAMTS8	-6.36	9.08	3.18E-28	1.68E-24	protein_coding
A_33_P3240752	CLDN18	-7.76	11.78	3.27E-28	1.68E-24	protein_coding
A_23_P68610	TPX2	5.10	7.74	1.05E-27	4.95E-24	protein_coding
A_24_P75190	HBD	-8.42	12.68	4.16E-27	1.83E-23	protein_coding
A_23_P166823	TNNC1	-5.39	9.14	6.23E-27	2.56E-23	protein_coding
CUST_34659_PI428871386	ENST00000595886.1	-5.14	7.01	7.63E-27	2.76E-23	lincRNA
A_23_P74115	RAD54L	5.21	7.37	8.58E-27	2.93E-23	protein_coding
A_33_P3395008	ACOXL	-5.23	9.29	9.65E-27	3.02E-23	protein_coding
A_33_P3252781	PLAC9	-5.25	8.39	1.43E-26	4.18E-23	protein_coding
A_33_P3312466	BTNL9	-5.47	7.67	1.97E-26	5.27E-23	protein_coding
A_23_P416774	CLIC5	-6.29	11.67	2.47E-26	6.35E-23	protein_coding
A_23_P26557	C16orf59	5.60	7.40	3.90E-26	9.27E-23	protein_coding
A_23_P115246	FCN3	-7.39	10.55	5.52E-26	1.26E-22	protein_coding
CUST_21802_PI428871386	ENST00000411904.1	-5.64	8.33	6.90E-26	1.46E-22	antisense
CUST_22088_PI428871386	ENST00000420825.1	-3.47	6.18	9.36E-26	1.92E-22	lincRNA
A_23_P311895	CLIC5	-7.08	8.38	1.07E-25	2.10E-22	protein_coding
A_33_P3283824	SLC39A8	-5.77	8.10	1.09E-25	2.10E-22	protein_coding
A_23_P217428	ARHGAP6	-3.96	6.54	1.62E-25	2.93E-22	protein_coding
A_33_P3329378	CNTN6	-3.40	6.93	2.39E-25	4.09E-22	protein_coding
A_24_P13041	RTKN2	-5.88	8.71	3.13E-25	5.08E-22	protein_coding
CUST_21801_PI428871386	ENST00000411904.1	-5.55	8.12	3.73E-25	5.89E-22	antisense
A_33_P3807062	HJURP	6.39	8.37	4.24E-25	6.52E-22	protein_coding
A_33_P3214948	SPOCK2	-4.67	12.09	4.77E-25	7.16E-22	protein_coding
A_23_P107421	TK1	6.04	12.54	5.38E-25	7.70E-22	protein_coding
A_23_P157914	MAMDC2	-4.51	7.07	5.81E-25	8.13E-22	protein_coding
A_33_P3419460	VAPA	-3.28	6.95	6.85E-25	9.37E-22	protein_coding
CUST_5501_PI428871386	ENST00000425887.1	-5.14	7.07	7.06E-25	9.44E-22	lincRNA
A_33_P3303717	ACADL	-5.56	7.61	8.08E-25	1.06E-21	protein_coding
A_23_P88740	CENPN	4.56	8.80	8.40E-25	1.08E-21	protein_coding
A_23_P156890	TCF21	-4.41	7.37	9.30E-25	1.17E-21	protein_coding
A_23_P166566	CCDC48	-6.69	9.26	1.02E-24	1.25E-21	protein_coding
A_23_P52227	GDF10	-7.40	8.63	1.27E-24	1.50E-21	protein_coding
CUST_39817_PI428871386	ENST00000588495.1	-3.42	7.37	1.29E-24	1.50E-21	lincRNA
A_23_P148475	KIF4A	4.49	6.82	1.48E-24	1.68E-21	protein_coding
CUST_39816_PI428871386	ENST00000588495.1	-3.56	7.68	1.50E-24	1.68E-21	lincRNA
A_32_P95729	FANCI	4.42	8.40	1.59E-24	1.75E-21	protein_coding
A_23_P346900	CACNA2D2	-5.66	9.21	1.72E-24	1.80E-21	protein_coding
A_33_P3209491	TNS1	-4.92	10.56	1.73E-24	1.80E-21	protein_coding
CUST_5500_PI428871386	ENST00000425887.1	-5.05	7.03	1.87E-24	1.92E-21	lincRNA
A_33_P3228435	FXYD1	-4.28	7.74	1.93E-24	1.94E-21	protein_coding
A_23_P209700	NMUR1	-3.46	6.91	2.24E-24	2.16E-21	protein_coding
A_24_P782308	NEDD4L	-3.55	7.16	2.70E-24	2.55E-21	protein_coding
A_33_P3397443	PKMYT1	3.94	8.98	5.31E-24	4.71E-21	protein_coding
A_23_P150316	MMP12	9.78	7.86	5.40E-24	4.71E-21	protein_coding
A_33_P3323847	RECQL4	5.07	9.41	5.43E-24	4.71E-21	protein_coding
A_33_P3378514	PDE5A	-5.11	10.51	5.43E-24	4.71E-21	protein_coding
CUST_31893_PI428871386	ENST00000560278.1	4.64	8.95	6.62E-24	5.66E-21	protein_coding
CUST_33192_PI428871386	ENST00000535363.1	-4.21	7.84	6.77E-24	5.70E-21	antisense
A_23_P150935	TROAP	4.56	8.17	7.83E-24	6.42E-21	protein_coding
CUST_34663_PI428871386	ENST00000598996.1	-3.76	7.32	8.58E-24	6.94E-21	lincRNA

CUST_34658_PI428871386	ENST00000595886.1	-3.92	6.61	1.12E-23	8.83E-21	lincRNA
A_32_P171328	UBE2S	3.06	10.86	1.32E-23	1.02E-20	protein_coding
CUST_20289_PI428871386	ENST00000531875.1	5.40	9.04	1.68E-23	1.28E-20	protein_coding
A_23_P18579	PTTG2	4.75	8.91	1.68E-23	1.28E-20	protein_coding
CUST_39821_PI428871386	ENST00000594315.1	-3.83	7.60	1.85E-23	1.39E-20	lincRNA
A_23_P51085	SPC25	6.05	7.24	1.91E-23	1.42E-20	protein_coding
CUST_25779_PI428871386	ENST00000504932.2	-2.59	6.61	2.23E-23	1.63E-20	antisense
CUST_7150_PI428871386	ENST00000420418.1	-3.02	5.89	2.92E-23	2.12E-20	antisense
CUST_34664_PI428871386	ENST00000599749.1	-4.44	7.47	3.02E-23	2.16E-20	lincRNA
A_23_P147711	NPR1	-3.90	7.53	3.29E-23	2.31E-20	protein_coding
CUST_4020_PI428871386	ENST00000456450.1	-3.95	6.62	3.30E-23	2.31E-20	antisense
CUST_4021_PI428871386	ENST00000456450.1	-3.67	6.72	3.42E-23	2.37E-20	antisense
CUST_33190_PI428871386	ENST00000566787.1	-5.30	7.91	3.47E-23	2.37E-20	lincRNA
A_23_P146456	CTSL2	5.82	8.03	3.75E-23	2.54E-20	protein_coding
A_23_P215048	KIAA0408	-5.72	7.86	4.32E-23	2.89E-20	protein_coding
A_23_P88331	DLGAP5	5.19	7.05	4.46E-23	2.95E-20	protein_coding
A_24_P218979	CDCA3	3.78	7.54	4.79E-23	3.14E-20	protein_coding
CUST_20290_PI428871386	ENST00000531875.1	5.13	9.32	4.90E-23	3.18E-20	protein_coding
A_33_P3275702	FMO2	-6.21	10.58	4.96E-23	3.18E-20	protein_coding
A_33_P3291831	CEP55	5.45	6.99	5.16E-23	3.27E-20	protein_coding
CUST_34662_PI428871386	ENST00000598996.1	-4.20	7.34	5.65E-23	3.55E-20	lincRNA
A_33_P3414157	MLPH	-3.41	7.38	6.16E-23	3.81E-20	protein_coding
CUST_34665_PI428871386	ENST00000599749.1	-3.74	7.37	6.20E-23	3.81E-20	lincRNA
A_33_P3275801	DES	-4.09	10.63	6.25E-23	3.81E-20	protein_coding
CUST_24751_PI428871386	ENST00000544553.1	5.01	7.40	8.88E-23	5.25E-20	antisense
A_23_P59637	DOCK4	-4.04	8.78	8.98E-23	5.27E-20	protein_coding
CUST_33193_PI428871386	ENST00000535363.1	-4.29	7.81	1.10E-22	6.40E-20	antisense
A_33_P3325262	SLC6A8	4.30	9.01	1.14E-22	6.58E-20	protein_coding
A_23_P124417	BUB1	4.91	7.02	1.18E-22	6.70E-20	protein_coding
A_23_P72668	SDPR	-6.73	9.51	1.32E-22	7.47E-20	protein_coding
CUST_33191_PI428871386	ENST00000566787.1	-5.00	7.98	1.34E-22	7.50E-20	lincRNA
A_33_P3310189	ADRB1	-6.01	8.63	1.40E-22	7.74E-20	protein_coding
A_24_P108311	NEDD4L	-4.47	10.66	1.43E-22	7.87E-20	protein_coding
CUST_39824_PI428871386	ENST00000598215.1	-4.15	8.74	1.51E-22	8.24E-20	lincRNA
A_23_P144807	SEPT8_	-3.96	7.22	1.68E-22	9.04E-20	protein_coding
CUST_36634_PI428871386	ENST00000588604.1	-2.63	6.04	1.69E-22	9.04E-20	antisense
A_24_P100517	C9orf140	4.69	7.07	1.73E-22	9.17E-20	protein_coding
A_24_P225970	SGOL1	3.66	6.88	1.82E-22	9.55E-20	protein_coding
A_24_P10233	DAPK2	-3.71	8.71	1.83E-22	9.55E-20	protein_coding
A_23_P125265	KPNA2	3.45	12.12	1.90E-22	9.80E-20	protein_coding
A_23_P401	CENPF	6.39	9.58	1.92E-22	9.82E-20	protein_coding
CUST_19805_PI428871386	ENST00000500705.2	-3.03	6.93	1.94E-22	9.82E-20	lincRNA
A_24_P945059	MYCT1	-3.58	6.77	1.95E-22	9.82E-20	protein_coding
A_23_P94422	MELK	5.29	7.09	2.14E-22	1.07E-19	protein_coding
A_23_P163306	CGNL1	-4.87	11.01	2.23E-22	1.10E-19	protein_coding
A_24_P55148	HIST1H2BJ	3.52	8.08	2.25E-22	1.10E-19	protein_coding
A_32_P310335	JAM2	-4.21	7.26	2.26E-22	1.10E-19	protein_coding
A_33_P3306110	CALCRL	-4.42	7.87	2.36E-22	1.14E-19	protein_coding
A_32_P100439	C7orf41	-2.90	10.32	2.37E-22	1.14E-19	protein_coding
A_33_P3257678	HIST2H3A	6.32	10.62	2.61E-22	1.24E-19	protein_coding
CUST_24752_PI428871386	ENST00000538355.1	4.02	7.36	2.66E-22	1.25E-19	antisense
A_23_P52017	ASPM	6.43	8.25	2.78E-22	1.29E-19	protein_coding
CUST_39820_PI428871386	ENST00000594315.1	-4.05	8.10	2.86E-22	1.31E-19	lincRNA
A_33_P3230548	KIF14	5.31	6.74	3.06E-22	1.40E-19	protein_coding
A_33_P3307903	CDKN3	4.40	6.61	3.15E-22	1.42E-19	protein_coding
CUST_12642_PI428871386	ENST00000515227.1	-2.68	5.79	3.40E-22	1.52E-19	lincRNA
A_23_P129144	MYZAP	-4.11	7.00	3.74E-22	1.64E-19	protein_coding
A_24_P940115	DLC1	-5.19	8.82	3.76E-22	1.64E-19	protein_coding
A_33_P3400477	STIL	4.76	7.69	4.43E-22	1.92E-19	protein_coding
A_33_P3258472	SPTBN1	-2.39	6.14	4.86E-22	2.09E-19	protein_coding
CUST_33861_PI428871386	ENST00000563424.2	-4.94	8.66	4.96E-22	2.12E-19	lincRNA
CUST_39825_PI428871386	ENST00000598215.1	-4.09	8.73	5.21E-22	2.21E-19	lincRNA
A_23_P382065	EMCN	-5.64	9.23	5.25E-22	2.21E-19	protein_coding

CUST_7149_PI428871386	ENST00000420418.1	-2.79	5.86	5.36E-22	2.24E-19	antisense
CUST_4627_PI428871386	ENST00000421976.2	-4.52	7.42	5.50E-22	2.27E-19	lincRNA
A_33_P3293918	SH2D3C	-2.94	11.51	5.51E-22	2.27E-19	protein_coding
CUST_254_PI428871386	ENST00000321399.3	-3.60	6.93	6.33E-22	2.56E-19	antisense
CUST_21602_PI428871386	ENST00000428643.1	3.74	12.83	6.48E-22	2.60E-19	antisense
CUST_21601_PI428871386	ENST00000428643.1	3.70	12.66	6.95E-22	2.78E-19	antisense
A_33_P3344127	HIST1H2AC	4.18	8.56	7.16E-22	2.83E-19	protein_coding
A_32_P150300	ENST00000432195	-2.61	7.01	7.20E-22	2.83E-19	antisense
A_23_P301521	KIAA1462	-4.62	10.24	7.26E-22	2.83E-19	protein_coding
A_33_P3275873	SFTPB	-4.74	15.68	7.37E-22	2.85E-19	protein_coding
CUST_22071_PI428871386	ENST00000415590.1	-6.05	8.71	7.68E-22	2.96E-19	lincRNA
CUST_22070_PI428871386	ENST00000415590.1	-4.40	9.24	7.98E-22	3.04E-19	lincRNA
A_33_P3311755	KIF23	3.58	6.22	8.00E-22	3.04E-19	protein_coding
A_23_P164057	MFAP4	-5.76	13.15	1.09E-21	4.00E-19	protein_coding
A_23_P21086	LGI3	-3.44	7.26	1.11E-21	4.05E-19	protein_coding
A_32_P175301	DENND3	-3.53	10.75	1.12E-21	4.08E-19	protein_coding
A_23_P368225	EME1	2.95	6.42	1.13E-21	4.08E-19	protein_coding
CUST_1676_PI428871386	ENST00000432195.1	-3.40	7.52	1.30E-21	4.67E-19	antisense
A_23_P130027	EPN3	4.84	7.04	1.32E-21	4.70E-19	protein_coding
A_23_P363174	HIST1H2AL	3.80	6.84	1.32E-21	4.70E-19	protein_coding
A_33_P3274049	ACOXL	-3.01	6.53	1.33E-21	4.70E-19	protein_coding
A_32_P471485	RTKN2	-4.09	7.06	1.49E-21	5.16E-19	protein_coding
A_24_P105933	VIPR1	-5.24	8.13	1.49E-21	5.16E-19	protein_coding
A_23_P349566	CCDC85A	-4.37	6.99	1.50E-21	5.16E-19	protein_coding
A_24_P305050	CD300LG	-2.87	5.77	1.50E-21	5.16E-19	protein_coding
A_23_P131935	FERMT1	4.86	8.69	1.51E-21	5.16E-19	protein_coding
A_33_P3284453	C6orf174	-5.68	8.65	1.52E-21	5.16E-19	protein_coding
A_24_P291658	ADH1A	-6.39	8.80	1.53E-21	5.16E-19	protein_coding
A_33_P3240328	PITX1	5.92	8.60	1.57E-21	5.27E-19	protein_coding
CUST_5514_PI428871386	ENST00000425578.1	-3.45	8.12	1.58E-21	5.28E-19	antisense
A_23_P79360	NOSTRIN	-5.57	9.57	1.66E-21	5.51E-19	protein_coding
CUST_255_PI428871386	ENST00000413472.1	-3.24	6.69	2.03E-21	6.67E-19	antisense
A_23_P41304	GYPE	-2.14	5.34	2.06E-21	6.73E-19	protein_coding
A_23_P417173	KCNA5	-2.79	5.88	2.23E-21	7.21E-19	protein_coding
A_24_P413884	CENPA	5.06	6.28	2.40E-21	7.68E-19	protein_coding
CUST_22068_PI428871386	ENST00000446372.1	-4.40	9.24	2.51E-21	8.02E-19	lincRNA
CUST_24753_PI428871386	ENST00000538355.1	3.90	7.28	2.64E-21	8.38E-19	antisense
A_24_P759674	OBFC1	-2.97	7.15	2.70E-21	8.51E-19	protein_coding
A_33_P3340025	GINS1	5.53	8.29	2.75E-21	8.64E-19	protein_coding
A_33_P3364180	FGD5	-4.40	10.21	3.01E-21	9.42E-19	protein_coding
A_24_P168925	CHRDL1	-6.56	9.48	3.18E-21	9.90E-19	protein_coding
A_24_P272310	MUSTN1	-2.90	7.21	3.36E-21	1.04E-18	protein_coding
CUST_35003_PI428871386	ENST00000573270.1	-3.87	6.50	3.41E-21	1.05E-18	lincRNA
A_23_P370989	MCM4	4.55	8.54	3.46E-21	1.05E-18	protein_coding
CUST_259_PI428871386	ENST00000445317.1	-3.39	6.78	3.48E-21	1.06E-18	antisense
A_24_P12626	CAV1	-5.86	10.93	3.56E-21	1.07E-18	protein_coding
CUST_22069_PI428871386	ENST00000446372.1	-5.98	8.90	3.57E-21	1.07E-18	lincRNA
CUST_22073_PI428871386	ENST00000434919.1	-6.03	8.95	3.68E-21	1.09E-18	lincRNA
CUST_31892_PI428871386	ENST00000560278.1	5.08	8.09	3.76E-21	1.11E-18	protein_coding
A_33_P3369371	GPX3	-4.77	9.37	3.80E-21	1.12E-18	protein_coding
A_24_P319374	GPA33	-5.42	7.68	3.98E-21	1.17E-18	protein_coding
A_23_P139682	PZP	-3.55	7.52	4.01E-21	1.17E-18	protein_coding
A_32_P524014	UTRN	-3.23	10.73	4.03E-21	1.17E-18	protein_coding
CUST_22072_PI428871386	ENST00000434919.1	-4.35	9.22	4.09E-21	1.18E-18	lincRNA
A_33_P3215640	PI16	-6.26	8.49	4.16E-21	1.20E-18	protein_coding
CUST_35002_PI428871386	ENST00000573270.1	-4.09	6.74	4.20E-21	1.20E-18	lincRNA
A_33_P3410235	DUOXA1	-3.16	8.74	4.39E-21	1.23E-18	protein_coding
CUST_26838_PI428871386	ENST00000552905.1	-2.43	5.53	4.39E-21	1.23E-18	antisense
A_23_P88731	RAD51	4.60	7.72	4.39E-21	1.23E-18	protein_coding
CUST_36575_PI428871386	ENST00000579003.1	-2.82	6.12	4.40E-21	1.23E-18	antisense
A_33_P3627001	PEBP4	-6.95	10.71	4.46E-21	1.24E-18	protein_coding
A_23_P216023	ANGPT1	-4.62	8.51	4.80E-21	1.33E-18	protein_coding
CUST_5515_PI428871386	ENST00000425578.1	-3.32	8.06	5.09E-21	1.40E-18	antisense

A_23_P334857	ZNF385B	-4.17	6.73	5.27E-21	1.44E-18	protein_coding
CUST_27336_PI428871386	ENST00000553165.1	-3.04	6.91	5.27E-21	1.44E-18	lincRNA
A_24_P316939	LRRFIP1	-3.23	8.95	5.34E-21	1.45E-18	protein_coding
CUST_25778_PI428871386	ENST00000504932.2	-2.10	6.23	5.45E-21	1.48E-18	antisense
A_23_P145631	GIMAP6	-3.96	8.55	6.33E-21	1.71E-18	protein_coding
A_23_P356684	ANLN	4.22	6.21	6.47E-21	1.73E-18	protein_coding
A_23_P365614	NOTCH4	-4.13	9.48	6.75E-21	1.80E-18	protein_coding
A_33_P3312499	MS4A15	-6.87	8.93	7.52E-21	2.00E-18	protein_coding
CUST_36574_PI428871386	ENST00000579003.1	-2.76	6.09	8.17E-21	2.16E-18	antisense
A_23_P308974	ADAMTSL3	-4.47	7.29	8.65E-21	2.27E-18	protein_coding
CUST_24750_PI428871386	ENST00000544553.1	4.59	6.92	8.73E-21	2.29E-18	antisense
CUST_20320_PI428871386	ENST00000458364.1	-3.09	6.55	8.77E-21	2.29E-18	lincRNA
A_32_P207767	ENST00000398832	-3.03	6.34	8.83E-21	2.29E-18	antisense
A_23_P161439	C10orf116	-5.48	12.62	8.95E-21	2.32E-18	protein_coding
A_33_P3361636	MGP	-4.50	13.99	9.73E-21	2.50E-18	protein_coding
CUST_8175_PI428871386	ENST00000421735.1	-3.25	6.78	1.05E-20	2.67E-18	antisense
A_33_P3400578	HLF	-5.89	8.48	1.05E-20	2.67E-18	protein_coding
A_23_P404481	S1PR1	-4.57	8.58	1.13E-20	2.85E-18	protein_coding
A_23_P422831	FAM189A2	-5.92	9.83	1.18E-20	2.94E-18	protein_coding
CUST_27164_PI428871386	ENST00000553141.1	-2.74	7.41	1.18E-20	2.95E-18	antisense
CUST_17017_PI428871386	ENST00000450544.1	-1.95	5.30	1.23E-20	3.03E-18	lincRNA
A_33_P3670415	NAT8L	-2.74	9.25	1.23E-20	3.03E-18	protein_coding
CUST_26837_PI428871386	ENST00000549388.1	-2.11	5.42	1.39E-20	3.41E-18	antisense
A_23_P133956	KIFC1	3.95	6.91	1.45E-20	3.53E-18	protein_coding
A_23_P150064	MMRN2	-2.73	8.18	1.50E-20	3.65E-18	protein_coding
CUST_16559_PI428871386	ENST00000420912.1	-3.64	6.15	1.51E-20	3.65E-18	lincRNA
CUST_33860_PI428871386	ENST00000563424.2	-5.30	9.18	1.56E-20	3.76E-18	lincRNA
CUST_15112_PI428871386	ENST00000586030.1	-2.07	5.34	1.57E-20	3.77E-18	antisense
CUST_8174_PI428871386	ENST00000421735.1	-3.02	6.66	1.61E-20	3.86E-18	antisense
CUST_36872_PI428871386	ENST00000580948.1	3.69	6.62	1.73E-20	4.10E-18	lincRNA
CUST_260_PI428871386	ENST00000445317.1	-3.11	6.71	1.76E-20	4.17E-18	antisense
A_24_P396702	CD302	-4.41	9.74	1.78E-20	4.20E-18	protein_coding
A_23_P250735	CBX7	-3.66	10.35	1.79E-20	4.20E-18	protein_coding
A_23_P118174	PLK1	2.74	6.33	1.82E-20	4.25E-18	protein_coding
A_23_P381261	ADCY4	-3.92	9.28	1.83E-20	4.25E-18	protein_coding
CUST_36635_PI428871386	ENST00000588604.1	-2.43	6.03	1.92E-20	4.44E-18	antisense
A_33_P3342305	ABCA8	-3.93	6.85	1.94E-20	4.46E-18	protein_coding
A_24_P116710	RAMP2	-2.15	6.10	1.98E-20	4.54E-18	protein_coding
A_32_P223777	IL6ST	-3.09	6.79	2.01E-20	4.61E-18	protein_coding
A_23_P25030	HSD17B6	-6.13	9.07	2.12E-20	4.82E-18	protein_coding
A_23_P32707	ESPL1	3.53	7.76	2.30E-20	5.19E-18	protein_coding
CUST_34620_PI428871386	ENST00000366314.4	-1.82	5.22	2.43E-20	5.43E-18	lincRNA
A_33_P3282556	TMEM204	-3.70	9.68	2.56E-20	5.70E-18	protein_coding
CUST_15137_PI428871386	ENST00000413945.1	-2.34	8.25	2.66E-20	5.91E-18	lincRNA
A_23_P214144	COL10A1	6.34	9.21	2.68E-20	5.94E-18	protein_coding
CUST_23273_PI428871386	ENST00000445873.1	-4.99	7.80	2.82E-20	6.22E-18	protein_coding
A_33_P3281552	RPGR	-2.20	6.30	2.92E-20	6.42E-18	protein_coding
A_23_P31996	SLC46A2	-2.75	6.69	3.04E-20	6.65E-18	protein_coding
A_23_P47410	ESAM	-3.46	8.47	3.13E-20	6.81E-18	protein_coding
A_23_P86021	SELENBP1	-4.23	12.59	3.28E-20	7.11E-18	protein_coding
A_24_P925040	CAV2	-4.36	7.99	3.29E-20	7.11E-18	protein_coding
CUST_1677_PI428871386	ENST00000432195.1	-2.85	7.27	3.90E-20	8.36E-18	antisense
A_33_P3334220	ACACB	-3.80	8.58	3.98E-20	8.51E-18	protein_coding
A_24_P109652	PEAK1	-2.65	10.09	4.48E-20	9.46E-18	protein_coding
CUST_253_PI428871386	ENST00000321399.3	-3.40	6.80	4.54E-20	9.57E-18	antisense
A_33_P3226212	JAM2	-4.59	9.48	4.89E-20	1.03E-17	protein_coding
A_23_P408955	E2F2	3.52	8.59	5.03E-20	1.05E-17	protein_coding
A_33_P3378051	SESTD1	-2.65	6.41	5.18E-20	1.07E-17	protein_coding
A_23_P81158	ADH1C	-6.65	10.20	5.18E-20	1.07E-17	protein_coding
A_23_P42358	FHL5	-3.06	6.28	5.24E-20	1.08E-17	protein_coding
A_24_P391230	CYYR1	-3.98	7.83	5.54E-20	1.14E-17	protein_coding
A_23_P85269	TTN	-4.33	7.25	5.80E-20	1.18E-17	protein_coding
A_23_P132718	SEMA3B	-4.39	10.79	5.81E-20	1.18E-17	protein_coding

A_33_P3340040	GINS4	3.58	7.86	5.82E-20	1.18E-17	protein_coding
A_23_P379614	OIP5	5.07	7.56	5.96E-20	1.21E-17	protein_coding
A_33_P3310104	SERPINB5	6.19	7.08	6.12E-20	1.24E-17	protein_coding
CUST_4626_PI428871386	ENST00000421976.2	-3.80	7.23	6.74E-20	1.35E-17	lincRNA
A_23_P50426	KANK2	-3.94	11.66	6.99E-20	1.39E-17	protein_coding
A_23_P217088	AK1	-3.33	10.26	7.29E-20	1.45E-17	protein_coding
CUST_15138_PI428871386	ENST00000413945.1	-2.96	7.12	7.53E-20	1.49E-17	lincRNA
CUST_15141_PI428871386	ENST00000426635.1	-3.21	6.42	7.72E-20	1.52E-17	lincRNA
A_24_P123347	PPAT	3.39	7.45	7.84E-20	1.54E-17	protein_coding
A_23_P97606	GSTM5	-3.19	8.49	7.99E-20	1.56E-17	protein_coding
A_33_P3295313	MS4A2	-2.94	6.20	8.19E-20	1.59E-17	protein_coding
A_24_P227091	KIF11	3.80	7.10	8.21E-20	1.59E-17	protein_coding
A_23_P85015	MAOB	-3.44	7.19	8.32E-20	1.61E-17	protein_coding
CUST_16064_PI428871386	ENST00000446476.1	-2.29	5.55	8.45E-20	1.63E-17	lincRNA
A_33_P3808996	SPATS2	2.19	8.68	8.54E-20	1.64E-17	protein_coding
CUST_39819_PI428871386	ENST00000599801.1	-2.22	7.21	8.54E-20	1.64E-17	lincRNA
A_23_P207742	THRA	-3.31	11.16	8.78E-20	1.68E-17	protein_coding
A_33_P3342410	EEF2K	-2.71	9.60	8.89E-20	1.69E-17	protein_coding
CUST_39823_PI428871386	ENST00000597702.1	-4.16	8.77	9.13E-20	1.73E-17	lincRNA
A_33_P3300837	LDB2	-2.79	7.15	9.17E-20	1.74E-17	protein_coding
A_24_P313186	CALM1	-3.11	9.29	9.23E-20	1.74E-17	protein_coding
CUST_40563_PI428871386	ENST00000449270.1	-5.61	7.43	9.38E-20	1.77E-17	lincRNA
A_33_P3279470	AGRP	-5.22	7.97	9.51E-20	1.77E-17	protein_coding
A_23_P53276	TIMELESS	3.31	10.19	9.51E-20	1.77E-17	protein_coding
A_23_P35219	NEK2	4.78	6.20	9.61E-20	1.79E-17	protein_coding
A_23_P897	C1orf116	-5.49	10.62	9.65E-20	1.79E-17	protein_coding
A_23_P70007	HMMR	5.21	7.83	9.93E-20	1.84E-17	protein_coding
A_23_P209200	CCNE1	4.25	9.02	1.02E-19	1.87E-17	protein_coding
CUST_40564_PI428871386	ENST00000449270.1	-4.85	7.23	1.08E-19	1.97E-17	lincRNA
A_23_P365817	PPP1R14B	3.14	11.69	1.09E-19	1.97E-17	protein_coding
A_32_P96036	MEX3A	5.07	8.06	1.09E-19	1.97E-17	protein_coding
A_33_P3212994	ZWINT	3.79	6.98	1.11E-19	2.00E-17	protein_coding
A_23_P416468	PIF1	3.00	7.43	1.12E-19	2.02E-17	protein_coding
A_24_P56363	CAB39L	-3.34	8.05	1.14E-19	2.04E-17	protein_coding
A_23_P426305	AOC3	-6.48	11.28	1.25E-19	2.21E-17	protein_coding
CUST_8193_PI428871386	ENST00000456560.2	-3.99	10.21	1.31E-19	2.31E-17	protein_coding
CUST_39818_PI428871386	ENST00000599801.1	-2.15	7.22	1.35E-19	2.36E-17	lincRNA
CUST_39822_PI428871386	ENST00000597702.1	-4.06	8.88	1.68E-19	2.91E-17	lincRNA
CUST_4545_PI428871386	ENST00000422449.1	-2.40	10.40	1.73E-19	2.99E-17	antisense
A_33_P3392187	CCDC85A	-3.65	6.70	1.77E-19	3.05E-17	protein_coding
A_23_P26386	TPPP3	-4.54	9.73	1.82E-19	3.12E-17	protein_coding
CUST_28463_PI428871386	ENST00000589800.1	-2.35	6.34	1.85E-19	3.16E-17	antisense
A_23_P7976	HIST1H1E	2.75	11.17	1.86E-19	3.17E-17	protein_coding
A_23_P1904	MS4A2	-4.10	7.43	1.88E-19	3.20E-17	protein_coding
A_32_P319200	GGTLC2	-3.00	8.39	1.92E-19	3.25E-17	protein_coding
A_24_P98249	TACC1	-4.03	10.19	1.93E-19	3.26E-17	protein_coding
A_23_P88630	BLM	3.77	7.92	1.95E-19	3.29E-17	protein_coding
A_24_P251599	CAV3	-3.44	7.14	2.04E-19	3.43E-17	protein_coding
A_23_P132763	VGLL3	-4.41	8.55	2.04E-19	3.43E-17	protein_coding
A_32_P103633	MCM2	3.83	9.19	2.20E-19	3.67E-17	protein_coding
A_33_P3363425	FRMD3	-3.85	7.84	2.26E-19	3.77E-17	protein_coding
A_23_P250564	PRKCE	-3.09	8.63	2.30E-19	3.82E-17	protein_coding
A_32_P465742	PIP5K1B	-4.00	7.40	2.34E-19	3.86E-17	protein_coding
CUST_28193_PI428871386	ENST00000376608.4	-2.85	6.44	2.52E-19	4.15E-17	protein_coding
A_33_P3331856	PDE1C	-3.31	7.05	2.54E-19	4.16E-17	protein_coding
A_33_P3249976	JAM2	-3.58	7.55	2.55E-19	4.17E-17	protein_coding
A_23_P211850	ABHD6	-3.09	8.80	2.73E-19	4.43E-17	protein_coding
A_23_P152804	NME1	2.30	12.62	2.90E-19	4.70E-17	protein_coding
CUST_8369_PI428871386	ENST00000474768.1	-2.51	6.02	3.03E-19	4.89E-17	antisense
CUST_31311_PI428871386	ENST00000434223.3	-2.07	6.27	3.14E-19	5.04E-17	lincRNA
A_23_P82990	OGN	-6.51	8.30	3.18E-19	5.10E-17	protein_coding
A_23_P5339	TMEM177	3.34	8.64	3.29E-19	5.21E-17	protein_coding
A_23_P200780	TGFBR3	-2.80	7.06	3.39E-19	5.36E-17	protein_coding

A_32_P96719	SHCBP1	3.50	6.52	3.63E-19	5.71E-17	protein_coding
A_23_P81859	HIST1H2AH	3.35	11.24	3.68E-19	5.78E-17	protein_coding
CUST_40740_PI428871386	ENST00000564492.1	-4.75	7.60	3.84E-19	5.99E-17	lincRNA
A_23_P41424	SLC39A8	-5.11	11.78	4.29E-19	6.60E-17	protein_coding
A_24_P217848	HIST1H2AK	3.31	12.03	4.30E-19	6.60E-17	protein_coding
CUST_42921_PI428871386	ENST00000420537.1	-2.96	6.52	4.69E-19	7.15E-17	antisense
A_33_P3411975	ABI3BP	-3.06	7.20	4.70E-19	7.15E-17	protein_coding
A_33_P3367860	CHRM1	-2.96	6.85	4.71E-19	7.15E-17	protein_coding
CUST_8368_PI428871386	ENST00000474768.1	-2.86	6.53	4.79E-19	7.23E-17	antisense
A_32_P5276	ARHGEF26	-3.67	8.67	4.88E-19	7.37E-17	protein_coding
A_33_P3325753	TRIOBP	-2.00	6.67	4.94E-19	7.44E-17	protein_coding
A_23_P132536	TRAK1	-2.28	9.29	5.28E-19	7.91E-17	protein_coding
CUST_26839_PI428871386	ENST00000552905.1	-2.25	5.44	5.32E-19	7.94E-17	antisense
CUST_4535_PI428871386	ENST00000421904.1	-2.34	10.36	5.33E-19	7.95E-17	antisense
A_23_P359540	HIST1H4F	3.59	9.01	5.44E-19	8.06E-17	protein_coding
CUST_34666_PI428871386	ENST00000597578.1	-2.34	6.14	5.55E-19	8.20E-17	lincRNA
A_33_P3214096	ATF3	-4.10	9.05	5.67E-19	8.37E-17	protein_coding
A_23_P30294	CDO1	-4.24	7.52	5.79E-19	8.53E-17	protein_coding
A_23_P363255	CCDC68	-2.96	6.62	6.01E-19	8.83E-17	protein_coding
CUST_29815_PI428871386	ENST00000555403.1	-2.79	7.39	6.09E-19	8.90E-17	lincRNA
A_23_P164451	TBX2	-4.57	10.69	6.09E-19	8.90E-17	protein_coding
CUST_29814_PI428871386	ENST00000555403.1	-2.79	7.09	6.16E-19	8.96E-17	lincRNA
A_23_P119562	CFD	-5.52	12.89	6.29E-19	9.09E-17	protein_coding
A_23_P157569	ADHFE1	-3.09	7.05	6.29E-19	9.09E-17	protein_coding
A_33_P3288754	C19orf48	3.08	9.72	6.32E-19	9.11E-17	protein_coding
CUST_256_PI428871386	ENST00000413472.1	-3.07	6.69	6.47E-19	9.31E-17	antisense
A_33_P3546033	ENST00000421976	-3.52	7.08	6.63E-19	9.52E-17	lincRNA
A_23_P52298	NPM3	2.84	9.34	6.99E-19	1.00E-16	protein_coding
A_33_P3420204	CRTC1	-2.53	9.03	7.10E-19	1.01E-16	protein_coding
A_23_P69100	ARHGEF26	-3.48	7.63	7.14E-19	1.02E-16	protein_coding
A_23_P146417	C9orf5	-2.68	10.45	7.26E-19	1.03E-16	protein_coding
A_32_P309404	SLC22A3	-2.91	6.16	7.47E-19	1.05E-16	protein_coding
CUST_5946_PI428871386	ENST00000450636.1	-2.97	6.25	7.79E-19	1.09E-16	lincRNA
A_32_P108655	AK4	4.57	9.16	8.13E-19	1.13E-16	protein_coding
A_23_P145006	SCGB3A2	-6.10	14.41	8.16E-19	1.13E-16	protein_coding
CUST_20875_PI428871386	ENST00000417887.1	-3.08	6.67	8.24E-19	1.14E-16	antisense
A_23_P156739	C6orf125	2.79	10.54	8.45E-19	1.16E-16	protein_coding
A_33_P3258117	HELLS	3.21	6.79	8.55E-19	1.17E-16	protein_coding
CUST_12640_PI428871386	ENST00000504068.1	-2.54	5.83	8.98E-19	1.22E-16	lincRNA
A_33_P3212172	SNX22	-3.61	7.63	9.04E-19	1.23E-16	protein_coding
A_23_P212284	POC1A	2.91	7.61	9.34E-19	1.26E-16	protein_coding
A_33_P3413523	DBF4	2.95	7.56	1.04E-18	1.40E-16	protein_coding
CUST_36873_PI428871386	ENST00000580948.1	3.58	6.41	1.11E-18	1.48E-16	lincRNA
CUST_8192_PI428871386	ENST00000456560.2	-3.78	10.09	1.11E-18	1.49E-16	protein_coding
A_33_P3212257	MMRN1	-4.76	8.45	1.13E-18	1.50E-16	protein_coding
A_33_P3285540	CLDN5	-4.77	11.61	1.13E-18	1.51E-16	protein_coding
CUST_20319_PI428871386	ENST00000458364.1	-2.95	6.73	1.14E-18	1.51E-16	lincRNA
A_33_P3356990	NPIPL3	1.53	5.06	1.17E-18	1.55E-16	protein_coding
A_23_P389141	SLFNL1	-2.93	6.54	1.18E-18	1.56E-16	protein_coding
A_33_P3394793	SUSD2	-3.09	7.20	1.35E-18	1.77E-16	protein_coding
A_33_P3237359	HMGB3	4.49	7.89	1.40E-18	1.83E-16	protein_coding
A_33_P3287879	HIST1H3H	3.88	8.77	1.40E-18	1.83E-16	protein_coding
CUST_15179_PI428871386	ENST00000438676.1	-2.75	6.87	1.51E-18	1.95E-16	lincRNA
A_23_P88069	LHFP	-4.20	10.93	1.53E-18	1.98E-16	protein_coding
CUST_12643_PI428871386	ENST00000515227.1	-1.93	5.69	1.54E-18	1.99E-16	lincRNA
A_33_P3369058	LRRK2	-5.87	8.81	1.56E-18	2.01E-16	protein_coding
A_32_P420009	ALS2CL	-2.06	6.92	1.57E-18	2.02E-16	protein_coding
CUST_27165_PI428871386	ENST00000553141.1	-2.53	6.63	1.64E-18	2.10E-16	antisense
A_23_P217637	TIMM8A	3.33	8.33	1.68E-18	2.13E-16	protein_coding
A_23_P29594	RPL39L	3.94	8.11	1.68E-18	2.13E-16	protein_coding
A_23_P49155	CDH3	3.91	9.42	1.71E-18	2.16E-16	protein_coding
CUST_31833_PI428871386	ENST00000565136.1	-2.53	6.18	1.72E-18	2.18E-16	lincRNA
A_33_P3363420	FRMD3	-3.23	7.25	1.82E-18	2.29E-16	protein_coding

CUST_22210_PI428871386	ENST00000443224.1	-2.69	5.72	1.85E-18	2.33E-16	antisense
A_33_P3386760	CHEK2	2.87	6.24	1.86E-18	2.34E-16	protein_coding
A_23_P21485	PID1	-5.18	9.06	1.87E-18	2.35E-16	protein_coding
A_24_P147169	PLA2G4F	-2.51	6.53	1.89E-18	2.36E-16	protein_coding
A_24_P306594	LOC100506310	-3.41	8.25	1.92E-18	2.40E-16	protein_coding
A_23_P44684	ECT2	3.18	6.88	2.15E-18	2.67E-16	protein_coding
A_23_P36753	ALDH2	-3.97	11.69	2.15E-18	2.67E-16	protein_coding
A_32_P187571	SCN2B	-3.14	6.63	2.28E-18	2.81E-16	protein_coding
A_24_P658584	SASH1	-3.13	8.03	2.29E-18	2.82E-16	protein_coding
A_33_P3223503	FRY	-2.56	6.62	2.34E-18	2.87E-16	protein_coding
A_24_P187799	C7orf58	-2.45	6.49	2.39E-18	2.92E-16	protein_coding
A_23_P119222	RETN	-4.95	7.99	2.39E-18	2.92E-16	protein_coding
A_33_P3216090	SHC3	-2.60	6.81	2.43E-18	2.95E-16	protein_coding
A_23_P53345	ARNTL2	4.14	7.22	2.45E-18	2.97E-16	protein_coding
A_23_P136460	FAM13B	-2.03	9.09	2.48E-18	3.00E-16	protein_coding
A_23_P14184	THSD1	-3.59	10.27	2.55E-18	3.07E-16	protein_coding
A_23_P204286	MGP	-3.66	12.88	2.70E-18	3.25E-16	protein_coding
CUST_40739_PI428871386	ENST00000564492.1	-5.05	8.24	2.78E-18	3.33E-16	lincRNA
CUST_5947_PI428871386	ENST00000450636.1	-2.97	6.59	2.90E-18	3.47E-16	lincRNA
CUST_15180_PI428871386	ENST00000438676.1	-2.65	6.84	2.99E-18	3.56E-16	lincRNA
CUST_27740_PI428871386	ENST00000528549.1	-4.08	8.60	3.08E-18	3.66E-16	antisense
CUST_18916_PI428871386	ENST00000499425.1	-3.40	6.19	3.08E-18	3.66E-16	lincRNA
A_23_P145197	BYSL	2.96	9.13	3.11E-18	3.69E-16	protein_coding
CUST_34634_PI428871386	ENST00000601250.1	-3.40	6.48	3.14E-18	3.71E-16	lincRNA
A_23_P314712	CABYR	5.59	7.31	3.15E-18	3.72E-16	protein_coding
A_23_P218858	ABI3BP	-3.98	7.76	3.32E-18	3.90E-16	protein_coding
A_33_P3334515	NDRG2	-3.66	11.92	3.35E-18	3.94E-16	protein_coding
CUST_34635_PI428871386	ENST00000601250.1	-3.27	6.40	3.51E-18	4.12E-16	lincRNA
CUST_25950_PI428871386	ENST00000540226.1	-5.38	8.11	3.53E-18	4.13E-16	antisense
A_33_P3289121	C2orf40	-6.30	8.83	3.61E-18	4.21E-16	protein_coding
CUST_28192_PI428871386	ENST00000376608.4	-2.48	6.28	3.70E-18	4.31E-16	protein_coding
A_33_P3395321	HN1	3.36	10.50	3.70E-18	4.31E-16	protein_coding
A_33_P3233871	F12	4.82	8.21	3.75E-18	4.36E-16	protein_coding
A_23_P425502	DONSON	2.72	6.61	4.13E-18	4.77E-16	protein_coding
A_33_P3217238	ATAD2	3.60	9.12	4.16E-18	4.80E-16	protein_coding
A_23_P64617	FZD4	-3.83	10.33	4.26E-18	4.89E-16	protein_coding
A_23_P303833	SCN4B	-3.59	8.50	4.29E-18	4.91E-16	protein_coding
A_24_P231104	LEPR	-4.12	7.01	4.29E-18	4.91E-16	protein_coding
A_33_P3659876	NCAPG2	2.68	6.52	4.32E-18	4.93E-16	protein_coding
A_32_P52785	DAAM2	-3.39	8.19	4.41E-18	5.03E-16	protein_coding
A_33_P3363245	NXPH4	3.30	7.25	4.48E-18	5.08E-16	protein_coding
CUST_16694_PI428871386	ENST00000578293.1	-2.75	7.36	4.58E-18	5.19E-16	antisense
A_33_P3237150	BMP2	-3.47	9.20	4.59E-18	5.19E-16	protein_coding
A_23_P145054	FAM162B	-3.85	8.91	4.60E-18	5.19E-16	protein_coding
A_23_P424561	RHOV	4.68	7.48	4.68E-18	5.27E-16	protein_coding
A_23_P7965	PGC	-6.20	9.91	4.74E-18	5.34E-16	protein_coding
CUST_20876_PI428871386	ENST00000417887.1	-2.94	6.57	4.91E-18	5.52E-16	antisense
CUST_30475_PI428871386	ENST00000558190.1	-2.14	9.57	4.98E-18	5.58E-16	protein_coding
CUST_42922_PI428871386	ENST00000420537.1	-3.04	6.61	5.14E-18	5.75E-16	antisense
A_32_P225816	PRDM16	-4.22	7.52	5.24E-18	5.85E-16	protein_coding
CUST_4278_PI428871386	ENST00000569008.1	-2.33	6.77	5.28E-18	5.88E-16	lincRNA
CUST_4279_PI428871386	ENST00000569008.1	-2.46	6.82	6.24E-18	6.90E-16	lincRNA
A_33_P3692756	LOC723809	-5.68	8.38	6.75E-18	7.43E-16	lincRNA
A_23_P36825	GPRC5A	-4.12	8.78	6.76E-18	7.43E-16	protein_coding
A_23_P66637	SGCA	-3.81	8.65	6.99E-18	7.67E-16	protein_coding
A_23_P144071	COL7A1	3.75	7.22	7.19E-18	7.86E-16	protein_coding
CUST_1445_PI428871386	ENST00000490006.2	-3.81	8.30	7.20E-18	7.86E-16	lincRNA
A_24_P697685	ESYT3	-2.27	6.15	7.36E-18	8.01E-16	protein_coding
CUST_34667_PI428871386	ENST00000597578.1	-2.35	6.17	7.62E-18	8.28E-16	lincRNA
A_23_P141405	NME2	2.03	15.05	7.69E-18	8.35E-16	protein_coding
CUST_25780_PI428871386	ENST00000532579.1	-2.50	6.54	7.76E-18	8.41E-16	antisense
A_23_P436281	HIST2H4B	3.17	10.38	7.93E-18	8.58E-16	protein_coding
A_23_P170679	COL4A3	-3.32	6.87	8.40E-18	9.05E-16	protein_coding

A_33_P3367447	ALDH3B1	-2.93	7.72	8.53E-18	9.18E-16	protein_coding
CUST_36865_PI428871386	ENST00000457958.2	4.34	6.81	9.29E-18	9.97E-16	lincRNA
CUST_36857_PI428871386	ENST00000581801.1	3.19	6.60	9.55E-18	1.02E-15	lincRNA
CUST_25951_PI428871386	ENST00000540226.1	-5.68	8.46	9.56E-18	1.02E-15	antisense
A_33_P3401647	PPP1R14A	-2.65	8.17	9.57E-18	1.02E-15	protein_coding
CUST_27337_PI428871386	ENST00000553165.1	-2.55	6.80	9.76E-18	1.04E-15	lincRNA
A_33_P3257993	RNF125	-2.84	8.39	9.78E-18	1.04E-15	protein_coding
A_33_P3387365	PXMP4	-4.20	8.52	1.03E-17	1.09E-15	protein_coding
CUST_31310_PI428871386	ENST00000434223.3	-1.94	6.07	1.07E-17	1.13E-15	lincRNA
A_23_P42588	GIMAP5	-3.68	8.77	1.07E-17	1.13E-15	protein_coding
A_33_P3691860	FAM122B	2.28	6.53	1.08E-17	1.14E-15	protein_coding
A_33_P3271430	MAP3K3	-2.26	11.00	1.08E-17	1.14E-15	protein_coding
A_33_P3387831	CENPM	3.02	9.99	1.09E-17	1.14E-15	protein_coding
A_33_P3242543	MAOA	-4.58	11.43	1.19E-17	1.25E-15	protein_coding
A_33_P3728167	CDH5	-2.10	7.18	1.20E-17	1.25E-15	protein_coding
CUST_24889_PI428871386	ENST00000342456.6	-3.18	10.38	1.22E-17	1.28E-15	protein_coding
A_23_P7679	NUP155	2.51	7.64	1.25E-17	1.30E-15	protein_coding
A_23_P207850	TNS4	4.01	8.16	1.26E-17	1.31E-15	protein_coding
A_33_P3861706	MTMR10	-2.78	9.89	1.27E-17	1.32E-15	protein_coding
A_23_P119353	RASIP1	-4.55	11.28	1.36E-17	1.40E-15	protein_coding
CUST_22211_PI428871386	ENST00000443224.1	-2.35	5.64	1.39E-17	1.44E-15	antisense
A_23_P126582	CASQ2	-2.31	5.41	1.42E-17	1.46E-15	protein_coding
A_24_P389916	LRRC32	-3.80	9.43	1.43E-17	1.47E-15	protein_coding
A_23_P99741	CDKL1	-2.50	7.78	1.48E-17	1.52E-15	protein_coding
A_33_P3738458	TNS1	-3.82	10.90	1.49E-17	1.53E-15	protein_coding
A_23_P500861	SYNE1	-3.84	9.79	1.53E-17	1.56E-15	protein_coding
A_23_P150583	SCGB1A1	-6.31	13.28	1.53E-17	1.56E-15	protein_coding
CUST_36654_PI428871386	ENST00000591313.1	-3.56	8.35	1.55E-17	1.57E-15	antisense
A_33_P3279590	OGN	-4.59	7.58	1.70E-17	1.72E-15	protein_coding
A_33_P3241393	SLC4A5	-2.95	6.55	1.71E-17	1.72E-15	protein_coding
CUST_20873_PI428871386	ENST00000420855.1	-2.88	6.63	1.73E-17	1.74E-15	antisense
CUST_3180_PI428871386	ENST00000425295.1	2.68	6.66	1.76E-17	1.76E-15	antisense
A_33_P3336686	CLIC3	-2.67	11.13	1.76E-17	1.76E-15	protein_coding
A_23_P212844	TACC3	2.85	7.71	1.81E-17	1.81E-15	protein_coding
CUST_36647_PI428871386	ENST00000592377.1	-3.54	8.36	1.81E-17	1.81E-15	antisense
A_24_P339514	CYP2B6	-5.36	11.06	1.89E-17	1.88E-15	protein_coding
CUST_27741_PI428871386	ENST00000528549.1	-3.26	7.59	1.95E-17	1.93E-15	antisense
A_23_P118025	DPEP2	-2.98	7.43	1.99E-17	1.97E-15	protein_coding
A_24_P260639	HIST1H1D	3.50	10.58	2.00E-17	1.97E-15	protein_coding
A_33_P3365810	MRPL12	2.56	9.41	2.05E-17	2.02E-15	protein_coding
CUST_8204_PI428871386	ENST00000433753.1	-2.49	6.29	2.07E-17	2.04E-15	protein_coding
CUST_3178_PI428871386	ENST00000417262.1	2.77	6.67	2.16E-17	2.12E-15	antisense
CUST_34668_PI428871386	ENST00000594398.1	-2.46	6.17	2.31E-17	2.26E-15	lincRNA
A_23_P121499	WFS1	-2.53	9.66	2.42E-17	2.36E-15	protein_coding
CUST_1444_PI428871386	ENST00000490006.2	-3.45	7.87	2.47E-17	2.41E-15	lincRNA
A_33_P3364571	TNXB	-2.15	6.05	2.57E-17	2.49E-15	protein_coding
A_32_P100109	REPS2	-2.84	7.19	2.62E-17	2.53E-15	protein_coding
A_23_P210869	COX4I2	-2.62	7.44	2.65E-17	2.56E-15	protein_coding
A_24_P330263	EDNRB	-2.51	6.55	2.71E-17	2.61E-15	protein_coding
CUST_4991_PI428871386	ENST00000409590.1	-1.68	8.65	2.73E-17	2.63E-15	lincRNA
CUST_7151_PI428871386	ENST00000416344.1	-1.91	5.43	2.77E-17	2.66E-15	antisense
A_33_P3288329	SORBS1	-2.11	6.58	2.80E-17	2.69E-15	protein_coding
A_23_P102611	WISP2	-5.06	11.28	2.82E-17	2.70E-15	protein_coding
CUST_29372_PI428871386	ENST00000553909.1	-3.57	9.53	2.83E-17	2.71E-15	protein_coding
A_23_P170352	MRPL12	2.52	9.97	3.07E-17	2.93E-15	protein_coding
A_23_P253896	NPNT	-4.17	9.95	3.09E-17	2.94E-15	protein_coding
A_33_P3404706	SPN	-2.91	7.40	3.13E-17	2.98E-15	protein_coding
A_23_P215875	DCAF13	2.83	10.11	3.18E-17	3.02E-15	protein_coding
CUST_37742_PI428871386	ENST00000584373.1	-3.53	7.02	3.20E-17	3.03E-15	lincRNA
CUST_24888_PI428871386	ENST00000342456.6	-3.41	9.51	3.48E-17	3.29E-15	protein_coding
A_23_P23829	CD34	-2.85	6.70	3.53E-17	3.33E-15	protein_coding
A_23_P30799	HIST1H3F	3.74	9.52	3.57E-17	3.36E-15	protein_coding
CUST_29156_PI428871386	ENST00000455487.1	-1.81	5.25	3.58E-17	3.37E-15	lincRNA

CUST_36651_PI428871386	ENST00000586706.1	-3.47	8.66	3.63E-17	3.41E-15	antisense
A_23_P58390	C4orf32	-2.96	8.30	3.71E-17	3.48E-15	protein_coding
CUST_18102_PI428871386	ENST00000438047.1	-3.17	7.86	3.73E-17	3.49E-15	antisense
CUST_36864_PI428871386	ENST00000457958.2	4.75	7.11	3.84E-17	3.59E-15	lincRNA
CUST_37740_PI428871386	ENST00000583490.1	-3.32	6.92	3.90E-17	3.64E-15	lincRNA
A_33_P3276638	HMBOX1	-2.01	9.24	3.96E-17	3.68E-15	protein_coding
A_33_P3311076	CYB5A	-3.84	12.27	4.09E-17	3.80E-15	protein_coding
A_23_P68547	MCM8	2.29	8.04	4.13E-17	3.83E-15	protein_coding
CUST_8200_PI428871386	ENST00000418576.1	-2.51	6.15	4.52E-17	4.18E-15	protein_coding
A_23_P41066	RASSF1	-2.06	10.61	4.53E-17	4.19E-15	protein_coding
CUST_16063_PI428871386	ENST00000446476.1	-2.27	5.67	4.66E-17	4.30E-15	lincRNA
A_33_P3322328	EPS15	-2.18	9.85	4.70E-17	4.33E-15	protein_coding
A_32_P159651	KAT2B	-2.69	8.14	4.71E-17	4.34E-15	protein_coding
A_24_P157156	CCDC150	3.08	5.78	4.81E-17	4.39E-15	protein_coding
CUST_36650_PI428871386	ENST00000586706.1	-3.47	8.40	4.90E-17	4.46E-15	antisense
CUST_18101_PI428871386	ENST00000438047.1	-3.24	7.90	4.94E-17	4.49E-15	antisense
A_23_P74914	URB2	2.35	7.67	5.18E-17	4.69E-15	protein_coding
CUST_22193_PI428871386	ENST00000431157.1	-3.31	6.84	5.24E-17	4.73E-15	antisense
A_23_P323751	FAM83D	4.21	7.42	5.37E-17	4.83E-15	protein_coding
CUST_13263_PI428871386	ENST00000413221.2	-2.00	7.23	5.44E-17	4.89E-15	lincRNA
CUST_36649_PI428871386	ENST00000590421.1	-3.56	8.81	5.65E-17	5.07E-15	antisense
A_23_P76109	RILPL2	-3.26	11.27	5.68E-17	5.08E-15	protein_coding
CUST_36656_PI428871386	ENST00000592009.1	-3.42	8.41	5.72E-17	5.11E-15	antisense
A_33_P3272231	MFSD2A	-4.21	10.00	5.77E-17	5.14E-15	protein_coding
A_23_P91390	THBD	-4.53	12.14	5.78E-17	5.15E-15	protein_coding
A_23_P407565	CX3CR1	-4.98	8.33	5.90E-17	5.24E-15	protein_coding
A_23_P202392	SUV39H2	2.98	7.20	5.95E-17	5.28E-15	protein_coding
A_24_P38347	DPYSL2	-3.87	9.31	5.99E-17	5.31E-15	protein_coding
A_23_P43800	BOP1	3.33	8.69	6.16E-17	5.45E-15	protein_coding
CUST_36653_PI428871386	ENST00000589814.1	-3.29	8.59	6.20E-17	5.47E-15	antisense
A_23_P354591	FAM125B	-2.19	7.54	6.40E-17	5.63E-15	protein_coding
CUST_15139_PI428871386	ENST00000436803.1	-2.45	6.20	6.45E-17	5.67E-15	lincRNA
CUST_20321_PI428871386	ENST00000442069.1	-2.88	6.72	6.48E-17	5.69E-15	lincRNA
A_33_P3293446	KIAA1462	-3.22	8.42	6.49E-17	5.69E-15	protein_coding
CUST_36655_PI428871386	ENST00000591313.1	-3.42	8.41	6.55E-17	5.73E-15	antisense
CUST_14093_PI428871386	ENST00000523242.1	-2.72	5.79	6.56E-17	5.73E-15	lincRNA
A_23_P99163	DRAM1	-3.85	11.69	6.72E-17	5.86E-15	protein_coding
A_23_P77103	SORD	2.67	9.06	6.77E-17	5.90E-15	protein_coding
A_33_P3335935	HN1L	2.60	6.85	6.91E-17	6.02E-15	protein_coding
CUST_36657_PI428871386	ENST00000592009.1	-3.47	8.38	6.93E-17	6.02E-15	antisense
A_24_P935103	ADCY9	-2.83	9.20	7.04E-17	6.11E-15	protein_coding
CUST_11170_PI428871386	ENST00000504050.1	-1.93	5.66	7.09E-17	6.15E-15	lincRNA
A_23_P202104	PPIF	2.67	10.91	7.16E-17	6.19E-15	protein_coding
A_24_P66780	FAM83B	5.16	6.14	7.29E-17	6.29E-15	protein_coding
A_33_P3399870	CERS6	2.56	8.01	7.36E-17	6.31E-15	protein_coding
A_23_P373054	C3orf23	-2.45	7.96	7.36E-17	6.31E-15	protein_coding
A_24_P462899	CENPW	3.48	8.70	7.54E-17	6.46E-15	protein_coding
A_33_P3245908	C10orf128	-2.83	7.26	8.27E-17	7.07E-15	protein_coding
A_23_P126120	CENPL	2.21	5.84	8.36E-17	7.12E-15	protein_coding
A_33_P3284463	ASAH1	-3.63	13.13	8.47E-17	7.21E-15	protein_coding
A_23_P66881	RGS9	-1.98	6.54	8.67E-17	7.37E-15	protein_coding
CUST_9469_PI428871386	ENST00000421498.1	4.63	6.50	8.68E-17	7.37E-15	antisense
CUST_33552_PI428871386	ENST00000562902.1	-2.91	7.43	9.21E-17	7.81E-15	lincRNA
A_33_P3396214	KREMEN2	4.99	6.51	9.31E-17	7.88E-15	protein_coding
A_33_P3257808	FANCD2	2.22	7.08	9.33E-17	7.89E-15	protein_coding
A_32_P112592	LOC339524	-3.10	7.38	9.77E-17	8.23E-15	lincRNA
A_33_P3418833	FLRT3	-5.28	9.65	9.84E-17	8.27E-15	protein_coding
A_24_P129232	SERINC1	-2.61	10.57	1.01E-16	8.47E-15	protein_coding
CUST_3181_PI428871386	ENST00000425295.1	2.68	6.60	1.01E-16	8.50E-15	antisense
CUST_9468_PI428871386	ENST00000421498.1	4.33	6.30	1.02E-16	8.57E-15	antisense
A_33_P3307495	STRA6	4.99	7.12	1.05E-16	8.77E-15	protein_coding
A_23_P136964	RPGR	-1.96	7.93	1.06E-16	8.88E-15	protein_coding
A_33_P3326210	ESCO2	3.28	5.85	1.08E-16	9.00E-15	protein_coding

A_33_P3388948	SNX30	-3.00	10.52	1.11E-16	9.23E-15	protein_coding
A_23_P35645	RBM17	-2.01	9.66	1.12E-16	9.29E-15	protein_coding
A_33_P3279629	UCN2	2.94	5.74	1.21E-16	1.00E-14	protein_coding
A_23_P256205	ABLIM3	-3.05	8.28	1.21E-16	1.00E-14	protein_coding
A_24_P413437	NONO	2.57	9.27	1.21E-16	1.00E-14	protein_coding
A_24_P192914	AMICA1	-3.75	11.68	1.21E-16	1.00E-14	protein_coding
A_23_P50990	CENPO	2.81	6.44	1.22E-16	1.00E-14	protein_coding
A_23_P213857	C7	-5.15	9.29	1.22E-16	1.00E-14	protein_coding
A_33_P3338698	IHH	-4.00	7.19	1.22E-16	1.00E-14	protein_coding
CUST_30474_PI428871386	ENST00000558190.1	-2.03	9.47	1.23E-16	1.01E-14	protein_coding
A_23_P130974	KIAA1683	-4.52	8.97	1.27E-16	1.04E-14	protein_coding
A_32_P89310	PLEKHM3	-2.28	8.24	1.27E-16	1.04E-14	protein_coding
A_23_P96965	SYNC	-2.68	8.42	1.29E-16	1.05E-14	protein_coding
A_23_P205567	PRKCH	-2.25	10.00	1.35E-16	1.10E-14	protein_coding
A_23_P77310	ZFP106	-2.09	10.18	1.38E-16	1.12E-14	protein_coding
A_33_P3368879	LMOD1	-2.64	7.17	1.41E-16	1.15E-14	protein_coding
A_33_P3281741	RFX8	-2.66	6.98	1.42E-16	1.15E-14	protein_coding
CUST_36652_PI428871386	ENST00000589814.1	-3.46	8.74	1.45E-16	1.18E-14	antisense
A_33_P3210160	ZNF865	-2.72	17.18	1.47E-16	1.19E-14	protein_coding
A_23_P101992	MARCO	-4.71	12.91	1.49E-16	1.21E-14	protein_coding
A_33_P3257030	LEPREL4	3.08	10.37	1.53E-16	1.23E-14	protein_coding
A_23_P15844	BRIP1	2.75	5.61	1.53E-16	1.23E-14	protein_coding
A_24_P274831	GIMAP7	-3.58	10.47	1.58E-16	1.27E-14	protein_coding
CUST_42099_PI428871386	ENST00000434081.1	-1.96	5.43	1.64E-16	1.32E-14	lincRNA
CUST_8201_PI428871386	ENST00000418576.1	-2.31	6.21	1.65E-16	1.33E-14	protein_coding
A_33_P3275878	SFTPB	-6.65	15.48	1.68E-16	1.35E-14	protein_coding
A_33_P3901921	C12orf48	2.29	5.68	1.70E-16	1.36E-14	protein_coding
A_23_P83328	ENG	-2.82	9.18	1.75E-16	1.40E-14	protein_coding
CUST_42097_PI428871386	ENST00000439088.1	-2.07	5.42	1.78E-16	1.42E-14	lincRNA
CUST_25781_PI428871386	ENST00000532579.1	-2.09	6.46	1.78E-16	1.42E-14	antisense
CUST_36648_PI428871386	ENST00000590421.1	-3.35	8.60	1.80E-16	1.44E-14	antisense
A_33_P3363168	SSH2	-2.40	9.02	1.81E-16	1.44E-14	protein_coding
CUST_8370_PI428871386	ENST00000481312.1	-1.63	5.76	1.92E-16	1.53E-14	antisense
CUST_3601_PI428871386	ENST00000440665.1	3.22	8.09	1.93E-16	1.53E-14	antisense
A_23_P501010	COL17A1	3.43	6.52	1.95E-16	1.54E-14	protein_coding
A_23_P20022	HILPDA	3.15	9.94	2.02E-16	1.59E-14	protein_coding
A_33_P3338300	CASZ1	-2.99	8.46	2.02E-16	1.59E-14	protein_coding
A_23_P137143	DKC1	2.57	9.69	2.04E-16	1.61E-14	protein_coding
A_23_P216596	SVEP1	-3.61	8.29	2.08E-16	1.63E-14	protein_coding
A_23_P214969	CITED2	-2.54	11.01	2.17E-16	1.69E-14	protein_coding
CUST_8753_PI428871386	ENST00000496994.1	-2.12	5.65	2.18E-16	1.70E-14	lincRNA
CUST_25079_PI428871386	ENST00000562197.1	-2.13	10.52	2.23E-16	1.74E-14	protein_coding
CUST_3603_PI428871386	ENST00000450783.1	3.26	8.04	2.24E-16	1.74E-14	antisense
A_23_P109452	CHEK2	2.47	7.34	2.28E-16	1.77E-14	protein_coding
CUST_22192_PI428871386	ENST00000431157.1	-2.83	6.49	2.28E-16	1.77E-14	antisense
A_33_P3331366	TRIM25	-1.86	10.42	2.31E-16	1.79E-14	protein_coding
A_23_P127533	DCUN1D5	2.70	10.25	2.37E-16	1.83E-14	protein_coding
A_33_P3357535	CYB5B	-2.00	7.10	2.37E-16	1.83E-14	protein_coding
A_33_P3313030	TXNDC15	-1.78	7.11	2.42E-16	1.87E-14	protein_coding
A_24_P20873	HIST1H4I	2.80	9.87	2.44E-16	1.87E-14	protein_coding
CUST_33551_PI428871386	ENST00000562902.1	-2.90	7.45	2.51E-16	1.93E-14	lincRNA
A_23_P212749	HTT	-2.26	8.72	2.52E-16	1.93E-14	protein_coding
A_33_P3302428	TNRC6C	-1.65	9.44	2.54E-16	1.94E-14	protein_coding
A_33_P3419696	FGF2	-2.57	6.38	2.64E-16	2.02E-14	protein_coding
CUST_31312_PI428871386	ENST00000560198.1	-1.72	6.39	2.67E-16	2.04E-14	lincRNA
CUST_16695_PI428871386	ENST00000578293.1	-2.58	7.28	2.71E-16	2.06E-14	antisense
A_23_P140876	ABCA3	-4.94	11.37	2.72E-16	2.07E-14	protein_coding
CUST_7970_PI428871386	ENST00000447181.1	2.03	5.78	2.74E-16	2.08E-14	antisense
A_23_P6909	CCRL1	-3.52	7.48	2.74E-16	2.08E-14	protein_coding
A_23_P109072	SALL4	3.48	6.59	2.75E-16	2.09E-14	protein_coding
A_33_P3280805	LMO7	-2.52	6.95	2.75E-16	2.09E-14	protein_coding
CUST_3602_PI428871386	ENST00000450783.1	3.21	8.10	2.80E-16	2.11E-14	antisense
A_23_P42802	PDIA4	2.46	9.85	2.88E-16	2.17E-14	protein_coding

CUST_8365_PI428871386	ENST00000460833.1	-2.29	6.39	2.95E-16	2.22E-14	antisense
A_33_P3216610	TMPRSS4	5.34	6.87	2.95E-16	2.22E-14	protein_coding
CUST_35385_PI428871386	ENST00000577684.1	-2.38	7.93	2.96E-16	2.22E-14	lincRNA
A_23_P258493	LMNB1	3.36	8.28	3.22E-16	2.41E-14	protein_coding
A_23_P502224	CYB5R3	-2.39	15.03	3.23E-16	2.41E-14	protein_coding
A_33_P3270384	PPP1R14B	1.99	12.56	3.51E-16	2.61E-14	protein_coding
CUST_5944_PI428871386	ENST00000416105.1	-1.96	5.83	4.03E-16	2.98E-14	lincRNA
A_23_P304450	GATA6	-3.60	9.07	4.07E-16	3.01E-14	protein_coding
A_23_P315892	ST6GALNAC6	-2.33	9.80	4.21E-16	3.11E-14	protein_coding
CUST_30176_PI428871386	ENST00000554032.1	3.88	7.43	4.22E-16	3.11E-14	lincRNA
CUST_36646_PI428871386	ENST00000592377.1	-3.32	8.40	4.23E-16	3.11E-14	antisense
A_23_P29773	LAMP3	-5.77	12.42	4.26E-16	3.13E-14	protein_coding
A_33_P3253596	KIF4A	2.43	5.80	4.38E-16	3.21E-14	protein_coding
A_23_P137697	SELP	-3.76	7.51	4.43E-16	3.25E-14	protein_coding
A_33_P3330608	PRAM1	-3.94	9.21	4.44E-16	3.26E-14	protein_coding
A_33_P3339375	ARHGAP11B	2.94	6.48	4.51E-16	3.30E-14	protein_coding
A_33_P3233834	IL6ST	-2.36	8.10	4.53E-16	3.31E-14	protein_coding
A_24_P205252	FAM13C	-1.88	5.53	4.53E-16	3.31E-14	protein_coding
CUST_36638_PI428871386	ENST00000585921.1	-1.46	5.48	4.63E-16	3.38E-14	antisense
A_24_P726336	PHACTR2	-3.02	8.93	4.71E-16	3.43E-14	protein_coding
A_33_P3867461	DKFZp686O1327	-2.00	13.63	4.84E-16	3.52E-14	lincRNA
CUST_8205_PI428871386	ENST00000433753.1	-2.47	6.35	4.90E-16	3.56E-14	protein_coding
CUST_15144_PI428871386	ENST00000421704.1	-2.11	6.12	5.03E-16	3.65E-14	lincRNA
CUST_37743_PI428871386	ENST00000584373.1	-3.00	6.72	5.20E-16	3.76E-14	lincRNA
A_33_P3716128	SMC4	2.77	9.21	5.29E-16	3.82E-14	protein_coding
A_33_P3326235	HBM	-2.46	6.35	5.50E-16	3.97E-14	protein_coding
CUST_22093_PI428871386	ENST00000428853.1	-1.89	5.11	5.54E-16	4.00E-14	lincRNA
A_23_P217015	SET	2.05	9.08	5.55E-16	4.00E-14	protein_coding
A_23_P169629	SHMT2	2.99	11.00	5.61E-16	4.03E-14	protein_coding
A_23_P152136	GINS3	2.41	8.58	5.63E-16	4.04E-14	protein_coding
CUST_35981_PI428871386	ENST00000581080.1	-1.76	6.52	5.75E-16	4.12E-14	antisense
A_33_P3283122	WWC2	-1.91	6.21	5.77E-16	4.13E-14	protein_coding
A_23_P204967	MPHOSPH8	-1.82	9.06	5.82E-16	4.16E-14	protein_coding
A_24_P940166	PAPSS2	-3.52	9.87	5.84E-16	4.17E-14	protein_coding
A_33_P3336642	THOC3	2.36	7.60	5.91E-16	4.21E-14	protein_coding
A_24_P561341	FAM150B	-4.24	7.77	5.95E-16	4.24E-14	protein_coding
A_23_P303072	GRIA1	-1.72	5.43	5.96E-16	4.24E-14	protein_coding
A_24_P217834	HIST1H3D	3.25	11.04	5.97E-16	4.24E-14	protein_coding
A_23_P307544	PLXNA2	-2.25	6.12	6.25E-16	4.44E-14	protein_coding
A_23_P386450	POLK	-2.18	8.95	6.35E-16	4.50E-14	protein_coding
A_32_P94722	BTBD9	-2.84	10.36	6.45E-16	4.56E-14	protein_coding
A_23_P400716	SHE	-3.37	7.20	6.52E-16	4.60E-14	protein_coding
A_23_P90542	ZNF540	-1.81	5.93	6.67E-16	4.70E-14	protein_coding
A_23_P404698	COL6A5	-3.24	6.90	6.93E-16	4.87E-14	protein_coding
A_32_P148672	SNRPD1	2.71	10.55	6.94E-16	4.87E-14	protein_coding
A_23_P68087	ATIC	2.07	9.15	6.99E-16	4.89E-14	protein_coding
A_33_P3280801	LMO7	-3.58	9.32	7.03E-16	4.91E-14	protein_coding
A_23_P67847	GALNT14	5.55	8.87	7.17E-16	5.01E-14	protein_coding
A_33_P3281468	STARD9	-1.89	6.41	7.25E-16	5.06E-14	protein_coding
A_33_P3286278	GRN	-3.13	15.47	7.48E-16	5.21E-14	protein_coding
A_24_P262407	THRA	-1.99	7.47	7.65E-16	5.32E-14	protein_coding
A_23_P161727	HSPB2	-3.31	9.07	8.04E-16	5.58E-14	protein_coding
A_33_P3375665	FOXO3	-1.99	11.18	8.10E-16	5.62E-14	protein_coding
A_24_P211565	C1QTNF6	2.80	7.98	8.22E-16	5.69E-14	protein_coding
A_23_P73982	TMEM48	2.79	8.64	8.36E-16	5.78E-14	protein_coding
CUST_30177_PI428871386	ENST00000554032.1	3.90	7.59	8.49E-16	5.86E-14	lincRNA
CUST_7618_PI428871386	ENST00000435651.1	-2.32	6.87	8.51E-16	5.87E-14	lincRNA
A_23_P207600	PSMD11	2.13	9.87	8.58E-16	5.91E-14	protein_coding
A_23_P133036	SLC34A2	-5.08	11.94	8.64E-16	5.95E-14	protein_coding
CUST_30178_PI428871386	ENST00000557691.1	3.93	7.52	8.65E-16	5.95E-14	lincRNA
A_23_P358917	CYP3A7	-4.53	7.73	8.82E-16	6.06E-14	protein_coding
A_23_P1361	ALDH18A1	2.20	8.48	9.02E-16	6.19E-14	protein_coding
A_23_P37702	TPSAB1	-4.18	9.51	9.27E-16	6.34E-14	protein_coding

A_24_P298174	CBX1	2.48	10.02	9.31E-16	6.36E-14	protein_coding
A_33_P3339212	TRIP13	4.37	8.18	9.46E-16	6.46E-14	protein_coding
CUST_16558_PI428871386	ENST00000420912.1	-2.24	5.68	9.55E-16	6.52E-14	lincRNA
A_33_P3267482	KIAA1804	-1.63	10.70	9.63E-16	6.56E-14	protein_coding
A_23_P344531	SYNPO	-2.97	10.13	9.82E-16	6.69E-14	protein_coding
CUST_4534_PI428871386	ENST00000421904.1	-1.56	9.63	9.98E-16	6.78E-14	antisense
CUST_15142_PI428871386	ENST00000426635.1	-2.28	5.88	1.02E-15	6.91E-14	lincRNA
CUST_3599_PI428871386	ENST00000416221.1	3.16	8.32	1.03E-15	7.00E-14	antisense
A_24_P86389	HIST1H2AM	2.42	11.71	1.07E-15	7.20E-14	protein_coding
A_23_P29803	POLR2H	2.60	11.30	1.07E-15	7.24E-14	protein_coding
CUST_20322_PI428871386	ENST00000442069.1	-2.47	6.41	1.11E-15	7.49E-14	lincRNA
CUST_25979_PI428871386	ENST00000318291.4	-2.05	8.16	1.12E-15	7.58E-14	antisense
A_23_P148047	PTGER4	-4.01	10.14	1.16E-15	7.80E-14	protein_coding
CUST_29155_PI428871386	ENST00000439299.1	-3.11	5.83	1.19E-15	8.00E-14	antisense
A_32_P34589	RSRC1	2.21	8.62	1.23E-15	8.20E-14	protein_coding
A_33_P3306146	PLAU	3.09	7.97	1.25E-15	8.37E-14	protein_coding
A_23_P258321	MRPS17	3.13	10.24	1.27E-15	8.46E-14	protein_coding
A_33_P3217465	MID1IP1	-3.12	11.87	1.28E-15	8.54E-14	protein_coding
A_33_P3214303	FOXP1	-1.79	11.64	1.33E-15	8.81E-14	protein_coding
A_23_P48217	APOLD1	-4.08	9.58	1.35E-15	8.93E-14	protein_coding
CUST_16540_PI428871386	ENST00000451368.1	-3.69	7.37	1.39E-15	9.22E-14	antisense
A_24_P8371	SPNS2	-3.49	10.82	1.42E-15	9.39E-14	protein_coding
CUST_3179_PI428871386	ENST00000417262.1	2.51	6.42	1.51E-15	9.94E-14	antisense
CUST_15951_PI428871386	ENST00000419134.1	-1.67	6.66	1.58E-15	1.04E-13	antisense
CUST_8714_PI428871386	ENST00000487814.1	-1.77	11.51	1.63E-15	1.07E-13	antisense
A_33_P3368301	BOLA3	2.46	8.46	1.65E-15	1.09E-13	protein_coding
CUST_8704_PI428871386	ENST00000570269.1	-2.94	9.12	1.73E-15	1.13E-13	lincRNA
A_24_P80500	BDH2	-2.23	6.33	1.76E-15	1.15E-13	protein_coding
CUST_3600_PI428871386	ENST00000440665.1	3.12	8.07	1.80E-15	1.18E-13	antisense
CUST_15780_PI428871386	ENST00000417800.1	-1.46	5.12	1.81E-15	1.18E-13	lincRNA
A_24_P321525	RERG	-3.20	8.26	1.84E-15	1.20E-13	protein_coding
A_24_P139901	GYPC	-2.68	8.92	1.86E-15	1.21E-13	protein_coding
A_33_P3217123	BCL2L12	1.84	11.56	1.88E-15	1.22E-13	protein_coding
CUST_34669_PI428871386	ENST00000594398.1	-1.94	6.00	1.88E-15	1.22E-13	lincRNA
A_23_P20035	GPR146	-2.85	7.50	1.90E-15	1.23E-13	protein_coding
CUST_26996_PI428871386	ENST00000546609.1	2.19	8.05	1.91E-15	1.24E-13	protein_coding
A_33_P3240333	PITX1	2.94	6.76	1.92E-15	1.24E-13	protein_coding
A_23_P168556	STX1A	1.87	8.23	1.93E-15	1.24E-13	protein_coding
A_23_P133770	CCDC167	2.62	8.71	1.93E-15	1.24E-13	protein_coding
A_33_P3313796	CCDC34	3.36	8.76	1.93E-15	1.24E-13	protein_coding
A_24_P238250	LGALS7	3.64	8.92	2.03E-15	1.30E-13	protein_coding
A_24_P29401	PIK3R1	-2.56	9.46	2.06E-15	1.33E-13	protein_coding
A_32_P166693	HEG1	-3.74	11.13	2.09E-15	1.34E-13	protein_coding
A_32_P85539	HCFC2	-2.13	8.07	2.24E-15	1.43E-13	protein_coding
A_24_P784765	CD59	-3.14	8.66	2.24E-15	1.44E-13	protein_coding
CUST_30179_PI428871386	ENST00000557691.1	3.69	7.17	2.24E-15	1.44E-13	lincRNA
A_23_P152655	ICAM2	-3.57	12.02	2.27E-15	1.45E-13	protein_coding
A_24_P120115	CFLAR	-2.65	8.95	2.28E-15	1.45E-13	protein_coding
A_33_P3378126	FBXO32	3.43	9.05	2.28E-15	1.45E-13	protein_coding
A_33_P3261505	EPG5	-1.67	7.08	2.29E-15	1.46E-13	protein_coding
A_23_P85800	CD52	-4.28	13.23	2.36E-15	1.50E-13	protein_coding
A_23_P41455	TRPC3	-2.06	6.31	2.36E-15	1.50E-13	protein_coding
A_23_P47879	STAT6	-2.31	11.90	2.37E-15	1.51E-13	protein_coding
A_24_P397107	CDC25A	3.15	6.24	2.38E-15	1.51E-13	protein_coding
A_24_P115511	RAB14	-1.65	6.64	2.39E-15	1.51E-13	protein_coding
A_33_P3210085	NET1	2.81	8.69	2.40E-15	1.52E-13	protein_coding
CUST_16775_PI428871386	ENST00000432866.2	-2.42	5.85	2.41E-15	1.53E-13	lincRNA
CUST_36856_PI428871386	ENST00000581801.1	2.91	6.43	2.44E-15	1.54E-13	lincRNA
A_33_P3418025	CTSO	-2.86	10.83	2.45E-15	1.54E-13	protein_coding
A_23_P33326	ADRA1B	-3.19	7.31	2.45E-15	1.54E-13	protein_coding
A_23_P125233	CNN1	-3.92	10.71	2.48E-15	1.56E-13	protein_coding
A_32_P49199	PGR	-2.10	5.68	2.53E-15	1.59E-13	protein_coding
A_23_P115064	CRABP2	3.50	8.37	2.53E-15	1.59E-13	protein_coding

A_33_P3417810	NOL10	1.95	9.32	2.63E-15	1.64E-13	protein_coding
A_33_P3335940	HN1L	2.31	7.53	2.64E-15	1.65E-13	protein_coding
A_33_P3391375	LANCL3	-1.68	9.38	2.66E-15	1.66E-13	protein_coding
A_24_P386622	ARRB1	-2.53	7.97	2.71E-15	1.69E-13	protein_coding
A_24_P260101	MME	-3.29	7.29	2.75E-15	1.71E-13	protein_coding
A_23_P21033	GMPS	2.75	10.81	2.78E-15	1.73E-13	protein_coding
CUST_33532_PI428871386	ENST00000561547.1	2.68	6.80	2.78E-15	1.73E-13	antisense
A_24_P251221	PPP2R5A	-2.30	9.25	2.84E-15	1.76E-13	protein_coding
CUST_24215_PI428871386	ENST00000531363.1	4.20	5.83	2.86E-15	1.77E-13	antisense
A_24_P342807	SLC30A6	1.64	6.33	2.89E-15	1.79E-13	protein_coding
A_23_P68730	PDXK	-2.63	10.74	2.90E-15	1.80E-13	protein_coding
A_32_P33114	KLB	-3.49	6.34	2.91E-15	1.80E-13	protein_coding
A_24_P37441	PDK1	2.92	8.68	2.96E-15	1.83E-13	protein_coding
A_23_P8801	CYP3A5	-4.63	7.67	2.97E-15	1.83E-13	protein_coding
A_33_P3416682	LOC400950	-1.67	6.43	2.98E-15	1.84E-13	protein_coding
A_23_P34888	CHIA	-5.21	8.74	3.01E-15	1.85E-13	protein_coding
A_23_P83403	LIMCH1	-3.39	8.39	3.08E-15	1.90E-13	protein_coding
A_23_P201790	PPP1R12B	-3.17	10.70	3.11E-15	1.91E-13	protein_coding
CUST_26710_PI428871386	ENST00000552933.1	2.91	7.34	3.19E-15	1.96E-13	antisense
A_32_P4018	ROR1	-3.13	7.30	3.23E-15	1.98E-13	protein_coding
CUST_29373_PI428871386	ENST00000553909.1	-3.24	9.18	3.42E-15	2.10E-13	protein_coding
CUST_16541_PI428871386	ENST00000451368.1	-3.63	7.72	3.44E-15	2.10E-13	antisense
CUST_23059_PI428871386	ENST00000413722.1	2.04	9.02	3.45E-15	2.11E-13	antisense
A_23_P69537	NMU	5.92	7.17	3.45E-15	2.11E-13	protein_coding
CUST_33533_PI428871386	ENST00000566956.1	2.65	6.82	3.50E-15	2.13E-13	antisense
A_24_P325520	SORT1	-2.61	9.93	3.53E-15	2.15E-13	protein_coding
A_33_P3511265	POSTN	4.16	9.90	3.53E-15	2.15E-13	protein_coding
CUST_4982_PI428871386	ENST00000452840.1	-1.73	10.85	3.66E-15	2.22E-13	lincRNA
CUST_12637_PI428871386	ENST00000514368.1	-1.75	5.56	3.67E-15	2.23E-13	lincRNA
A_23_P331700	SRRM3	2.96	6.33	3.71E-15	2.25E-13	protein_coding
A_33_P3333282	FGF11	3.47	6.03	3.81E-15	2.31E-13	protein_coding
A_23_P149259	TMEM79	3.09	7.75	4.28E-15	2.59E-13	protein_coding
A_24_P335620	SLC7A5	3.79	7.93	4.48E-15	2.71E-13	protein_coding
A_23_P140716	TPSD1	-1.98	6.21	4.63E-15	2.80E-13	protein_coding
A_23_P126212	CLSPN	2.71	5.73	4.97E-15	2.99E-13	protein_coding
A_24_P3783	HIST1H2BM	2.36	10.20	5.10E-15	3.06E-13	protein_coding
A_24_P356406	PAFAH1B1	-2.27	11.15	5.15E-15	3.09E-13	protein_coding
A_33_P3258702	SEC22C	-1.85	8.11	5.16E-15	3.09E-13	protein_coding
CUST_40239_PI428871386	ENST00000413496.1	-2.77	7.13	5.18E-15	3.10E-13	antisense
A_23_P24077	C10orf54	-2.37	10.03	5.20E-15	3.11E-13	protein_coding
A_23_P165783	MLPH	-3.91	8.77	5.42E-15	3.23E-13	protein_coding
CUST_20642_PI428871386	ENST00000433838.1	-1.78	5.21	5.66E-15	3.37E-13	antisense
A_23_P399078	TIMP3	-3.82	12.31	5.79E-15	3.44E-13	protein_coding
CUST_28829_PI428871386	ENST00000448411.1	-1.32	6.80	5.81E-15	3.44E-13	lincRNA
CUST_8705_PI428871386	ENST00000570269.1	-2.83	9.21	5.85E-15	3.46E-13	lincRNA
CUST_25978_PI428871386	ENST00000318291.4	-2.06	8.21	5.98E-15	3.54E-13	antisense
A_23_P353717	RMI2	4.27	8.78	6.04E-15	3.57E-13	protein_coding
A_23_P13425	CD81	-2.29	13.74	6.08E-15	3.59E-13	protein_coding
A_23_P27584	MYADM	-3.79	11.31	6.26E-15	3.69E-13	protein_coding
A_23_P30813	HIST1H4K	2.32	10.94	6.49E-15	3.82E-13	protein_coding
A_33_P3227400	COL4A4	-4.34	9.56	6.54E-15	3.84E-13	protein_coding
A_24_P211044	CSH1	-3.86	8.08	6.58E-15	3.87E-13	protein_coding
A_33_P3419383	PDLIM3	-2.80	7.00	6.61E-15	3.88E-13	protein_coding
A_23_P20427	RHOBTB2	-3.32	9.69	6.64E-15	3.89E-13	protein_coding
CUST_26711_PI428871386	ENST00000552933.1	3.08	7.25	6.67E-15	3.90E-13	antisense
CUST_3605_PI428871386	ENST00000454631.1	3.11	8.06	6.85E-15	4.00E-13	antisense
A_33_P3640690	ZEB1	-2.70	8.87	6.93E-15	4.04E-13	protein_coding
A_23_P205900	NTRK3	-1.60	5.87	7.15E-15	4.16E-13	protein_coding
CUST_3604_PI428871386	ENST00000454631.1	3.13	7.91	7.37E-15	4.28E-13	antisense
CUST_34812_PI428871386	ENST00000564394.1	-1.92	6.92	7.70E-15	4.47E-13	antisense
A_33_P3411296	PURA	-2.04	10.42	7.91E-15	4.58E-13	protein_coding
A_24_P105733	TNS1	-2.16	6.92	8.26E-15	4.78E-13	protein_coding
A_23_P305977	GRAMD2	-2.23	5.92	8.30E-15	4.80E-13	protein_coding

CUST_30840_PI428871386	ENST00000554735.1	-2.56	7.81	8.33E-15	4.81E-13	lincRNA
A_33_P3849275	FHL1	-2.38	12.01	8.35E-15	4.82E-13	protein_coding
A_23_P58588	SLIT3	-3.70	11.08	8.39E-15	4.84E-13	protein_coding
A_23_P8640	GPER	-4.14	8.69	8.57E-15	4.93E-13	protein_coding
A_23_P25293	NCAPD2	2.65	9.12	8.58E-15	4.93E-13	protein_coding
A_23_P74449	HPDL	4.10	6.78	8.69E-15	4.98E-13	protein_coding
A_23_P412562	C1orf162	-3.41	9.38	8.71E-15	4.99E-13	protein_coding
A_32_P197489	KLF13	-2.64	11.98	8.88E-15	5.08E-13	protein_coding
A_23_P12746	MRC1	-5.09	9.90	8.97E-15	5.13E-13	protein_coding
A_33_P3359115	LMBRD1	-1.97	11.13	9.03E-15	5.16E-13	protein_coding
A_23_P133995	PPIL1	2.24	10.56	9.15E-15	5.22E-13	protein_coding
A_32_P180958	PCYOX1	-2.35	10.52	9.17E-15	5.23E-13	protein_coding
A_33_P3281273	S1PR4	-3.85	10.64	9.17E-15	5.23E-13	protein_coding
CUST_3598_PI428871386	ENST00000416221.1	3.07	8.04	9.50E-15	5.40E-13	antisense
A_23_P32233	KLF4	-3.33	8.71	9.50E-15	5.40E-13	protein_coding
A_33_P3306264	LYPD3	3.11	6.46	9.60E-15	5.46E-13	protein_coding
CUST_26306_PI428871386	ENST00000538231.1	-1.93	5.98	9.94E-15	5.64E-13	antisense
A_23_P257111	FBP1	-4.77	11.73	1.02E-14	5.75E-13	protein_coding
A_24_P317907	SORBS1	-2.90	7.74	1.09E-14	6.13E-13	protein_coding
A_33_P3291294	EXTL3	-2.34	11.74	1.09E-14	6.14E-13	protein_coding
CUST_22291_PI428871386	ENST00000446807.1	-1.59	5.66	1.09E-14	6.16E-13	antisense
A_23_P47790	METTL1	2.07	8.39	1.12E-14	6.33E-13	protein_coding
A_24_P90216	LGR4	3.38	8.15	1.14E-14	6.40E-13	protein_coding
CUST_33205_PI428871386	ENST00000573379.1	-2.83	7.71	1.15E-14	6.47E-13	antisense
A_23_P321201	DENND5A	-1.95	10.05	1.17E-14	6.57E-13	protein_coding
A_23_P205177	F10	-2.34	7.08	1.17E-14	6.58E-13	protein_coding
A_23_P421032	SEC14L4	-3.85	8.40	1.20E-14	6.72E-13	protein_coding
CUST_7971_PI428871386	ENST00000447181.1	1.92	5.62	1.21E-14	6.76E-13	antisense
A_23_P66732	GSG2	2.47	5.41	1.22E-14	6.80E-13	protein_coding
A_23_P91283	CASS4	-2.27	6.81	1.23E-14	6.87E-13	protein_coding
A_33_P3422822	GJC2	-2.19	8.24	1.24E-14	6.89E-13	protein_coding
A_23_P33364	SH3D19	-3.03	11.17	1.24E-14	6.91E-13	protein_coding
CUST_11171_PI428871386	ENST00000504050.1	-1.91	5.77	1.31E-14	7.26E-13	lincRNA
A_23_P93772	HOXA5	-2.84	8.75	1.33E-14	7.39E-13	protein_coding
A_33_P3210288	FUT6	-2.42	16.73	1.35E-14	7.50E-13	protein_coding
CUST_33531_PI428871386	ENST00000561547.1	2.63	6.96	1.36E-14	7.54E-13	antisense
CUST_36136_PI428871386	ENST00000585620.1	-1.38	5.06	1.44E-14	7.96E-13	lincRNA
A_24_P945194	PDCD6IP	-1.92	8.60	1.46E-14	8.08E-13	protein_coding
A_23_P340722	XPOT	2.07	7.85	1.49E-14	8.23E-13	protein_coding
A_23_P256223	VBP1	1.96	8.05	1.49E-14	8.23E-13	protein_coding
A_23_P53176	FOLR1	-5.20	12.80	1.52E-14	8.35E-13	protein_coding
A_24_P381441	LMO3	-4.61	8.84	1.52E-14	8.35E-13	protein_coding
A_33_P3393766	C17orf96	3.78	8.79	1.54E-14	8.42E-13	protein_coding
CUST_7134_PI428871386	ENST00000343987.2	-2.06	8.24	1.54E-14	8.44E-13	lincRNA
CUST_29575_PI428871386	ENST00000552028.1	-2.53	6.88	1.55E-14	8.45E-13	lincRNA
A_33_P3410836	HIST1H4D	2.98	10.06	1.58E-14	8.63E-13	protein_coding
CUST_30843_PI428871386	ENST00000555174.1	-2.61	7.53	1.59E-14	8.68E-13	lincRNA
A_33_P3399090	DIXDC1	-2.29	8.41	1.63E-14	8.89E-13	protein_coding
A_24_P372223	MSR1	-4.23	8.98	1.66E-14	9.01E-13	protein_coding
CUST_35416_PI428871386	ENST00000435028.2	-2.52	7.03	1.74E-14	9.48E-13	lincRNA
A_23_P96325	ERCC6L	2.35	5.50	1.78E-14	9.67E-13	protein_coding
CUST_22638_PI428871386	ENST00000432535.1	-1.98	5.30	1.80E-14	9.75E-13	lincRNA
A_23_P303718	DST	-2.88	8.19	1.84E-14	9.99E-13	protein_coding
A_23_P212339	FYCO1	-1.93	9.12	1.91E-14	1.03E-12	protein_coding
A_24_P362881	IFT57	-2.00	5.96	1.91E-14	1.04E-12	protein_coding
A_33_P3378556	F11	-2.10	5.97	1.92E-14	1.04E-12	protein_coding
A_33_P3240063	C14orf48	-1.86	6.09	1.94E-14	1.05E-12	lincRNA
A_24_P214231	STIL	2.44	5.50	1.97E-14	1.07E-12	protein_coding
A_23_P215051	ECHDC1	-2.52	10.30	2.06E-14	1.11E-12	protein_coding
CUST_8708_PI428871386	ENST00000475939.1	-1.67	10.99	2.08E-14	1.12E-12	antisense
A_33_P3210139	PCF11	-1.94	9.77	2.12E-14	1.14E-12	protein_coding
CUST_37745_PI428871386	ENST00000579431.1	-1.97	5.47	2.12E-14	1.14E-12	lincRNA
A_33_P3289865	PLCL1	-2.16	6.39	2.13E-14	1.14E-12	protein_coding

A_23_P253752	FAM54A	2.61	6.58	2.13E-14	1.14E-12	protein_coding
A_23_P126291	SNRPE	2.18	11.47	2.13E-14	1.14E-12	protein_coding
CUST_3287_PI428871386	ENST00000458250.1	2.83	6.12	2.15E-14	1.15E-12	lincRNA
A_33_P3366053	ADPRH	-2.32	8.72	2.15E-14	1.15E-12	protein_coding
CUST_26229_PI428871386	ENST00000427111.3	-1.74	5.92	2.19E-14	1.17E-12	lincRNA
A_23_P70168	TARS	2.04	10.77	2.20E-14	1.17E-12	protein_coding
A_33_P3304668	COL1A1	4.28	12.71	2.29E-14	1.22E-12	protein_coding
CUST_22000_PI428871386	ENST00000454321.1	-2.42	7.50	2.29E-14	1.22E-12	antisense
A_24_P393844	DPH2	2.06	8.65	2.31E-14	1.23E-12	protein_coding
A_23_P167389	ARAP3	-3.05	9.78	2.34E-14	1.24E-12	protein_coding
A_33_P3310929	ADAM12	3.47	7.01	2.34E-14	1.24E-12	protein_coding
CUST_10848_PI428871386	ENST00000500765.1	-2.04	6.38	2.37E-14	1.26E-12	antisense
A_33_P3369760	GLIPR2	-3.11	9.25	2.39E-14	1.27E-12	protein_coding
A_24_P26073	LRPPRC	1.96	9.75	2.40E-14	1.27E-12	protein_coding
CUST_30024_PI428871386	ENST00000500036.2	-1.70	11.64	2.41E-14	1.28E-12	antisense
A_33_P3281795	MGLL	-3.85	11.22	2.42E-14	1.28E-12	protein_coding
CUST_4544_PI428871386	ENST00000422449.1	-1.46	9.71	2.43E-14	1.28E-12	antisense
CUST_25975_PI428871386	ENST00000537514.1	-1.73	7.04	2.43E-14	1.28E-12	antisense
A_23_P95060	EPHB3	2.73	7.55	2.44E-14	1.29E-12	protein_coding
A_23_P33093	ST6GALNAC5	-2.61	7.89	2.47E-14	1.30E-12	protein_coding
CUST_13738_PI428871386	ENST00000501695.3	-1.55	5.34	2.57E-14	1.35E-12	antisense
A_23_P132874	C3orf26	2.43	10.90	2.66E-14	1.40E-12	protein_coding
A_33_P3251901	APBB2	-1.59	8.12	2.67E-14	1.40E-12	protein_coding
A_24_P262201	SULT1A4	-2.40	10.90	2.70E-14	1.42E-12	protein_coding
A_33_P3413098	LOC100129550	-2.02	9.31	2.70E-14	1.42E-12	lincRNA
CUST_20262_PI428871386	ENST00000524499.1	2.42	7.05	2.77E-14	1.45E-12	antisense
A_33_P3265355	SNRPE	2.24	11.39	2.79E-14	1.46E-12	protein_coding
CUST_7745_PI428871386	ENST00000475197.1	-1.59	5.87	2.81E-14	1.47E-12	antisense
A_24_P234415	STAC	-2.78	6.46	2.84E-14	1.48E-12	protein_coding
A_23_P201248	SLC26A9	-4.05	7.67	2.90E-14	1.51E-12	protein_coding
A_23_P90130	NAPSA	-4.93	13.68	2.93E-14	1.52E-12	protein_coding
A_23_P55190	EFTUD2	1.60	11.58	3.03E-14	1.58E-12	protein_coding
A_23_P82979	LAMC3	-2.98	8.32	3.20E-14	1.66E-12	protein_coding
A_23_P394064	PTRF	-3.37	13.48	3.23E-14	1.68E-12	protein_coding
A_23_P323272	OSR1	-1.81	5.50	3.27E-14	1.69E-12	protein_coding
CUST_27534_PI428871386	ENST00000538559.2	-2.78	6.13	3.28E-14	1.70E-12	lincRNA
A_24_P943613	TBC1D1	-2.48	8.94	3.30E-14	1.70E-12	protein_coding
A_23_P163227	CKMT1A	2.57	8.40	3.30E-14	1.70E-12	protein_coding
A_23_P258612	ATP8A2	-3.26	7.68	3.35E-14	1.73E-12	protein_coding
CUST_35056_PI428871386	ENST00000443997.1	-1.98	6.52	3.39E-14	1.75E-12	antisense
CUST_24214_PI428871386	ENST00000531363.1	4.07	5.86	3.40E-14	1.75E-12	antisense
A_23_P354074	LYST	-2.13	8.35	3.53E-14	1.81E-12	protein_coding
A_24_P345451	CYBRD1	-2.25	9.18	3.54E-14	1.82E-12	protein_coding
A_23_P148372	CSTF2	1.83	8.60	3.59E-14	1.84E-12	protein_coding
CUST_17162_PI428871386	ENST00000451809.1	-2.48	7.73	3.68E-14	1.89E-12	antisense
A_23_P338890	PTPN1	-1.87	10.99	3.80E-14	1.94E-12	protein_coding
A_23_P166087	RASSF2	-3.56	10.59	3.81E-14	1.95E-12	protein_coding
A_33_P3212679	SNRPG	1.86	13.01	3.85E-14	1.96E-12	protein_coding
A_32_P95739	TPI1	1.90	13.05	3.85E-14	1.96E-12	protein_coding
CUST_26997_PI428871386	ENST00000546609.1	1.99	8.04	3.86E-14	1.96E-12	protein_coding
CUST_35430_PI428871386	ENST00000460772.2	-2.67	7.18	3.90E-14	1.98E-12	lincRNA
CUST_15140_PI428871386	ENST00000436803.1	-2.12	6.09	3.94E-14	2.00E-12	lincRNA
A_23_P47839	DDX55	1.72	7.80	4.01E-14	2.04E-12	protein_coding
CUST_30837_PI428871386	ENST00000556266.1	-2.51	7.47	4.02E-14	2.04E-12	lincRNA
CUST_35415_PI428871386	ENST00000442355.2	-2.58	7.05	4.06E-14	2.05E-12	lincRNA
A_33_P3232965	TDRD6	-2.29	6.11	4.06E-14	2.05E-12	protein_coding
CUST_15143_PI428871386	ENST00000421704.1	-1.97	6.18	4.06E-14	2.05E-12	lincRNA
CUST_4990_PI428871386	ENST00000409590.1	-1.58	9.11	4.08E-14	2.06E-12	lincRNA
CUST_18606_PI428871386	ENST00000523643.1	-3.15	7.51	4.14E-14	2.08E-12	antisense
CUST_33204_PI428871386	ENST00000573379.1	-2.58	7.33	4.14E-14	2.08E-12	antisense
A_24_P245108	USP7	-1.69	6.63	4.27E-14	2.15E-12	protein_coding
A_23_P50368	OSCAR	-3.01	9.85	4.28E-14	2.15E-12	protein_coding
CUST_17167_PI428871386	ENST00000424477.1	-2.65	8.02	4.34E-14	2.18E-12	antisense

CUST_13783_PI428871386	ENST00000518014.1	-2.54	7.95	4.34E-14	2.18E-12	lincRNA
A_33_P3881262	CSF3R	-3.20	10.44	4.37E-14	2.19E-12	protein_coding
A_23_P96041	TMEM164	-2.10	10.33	4.43E-14	2.22E-12	protein_coding
A_33_P3256725	PIP5K1B	-1.72	6.09	4.65E-14	2.32E-12	protein_coding
A_24_P53519	CHAF1A	2.05	9.38	4.77E-14	2.38E-12	protein_coding
A_23_P64860	SELPLG	-2.76	9.19	4.94E-14	2.46E-12	protein_coding
A_33_P3276435	FILIP1	-2.40	7.15	4.98E-14	2.47E-12	protein_coding
CUST_30846_PI428871386	ENST00000557532.1	-2.42	7.49	5.00E-14	2.48E-12	lincRNA
A_33_P3240507	KCTD12	-2.97	12.01	5.01E-14	2.49E-12	protein_coding
CUST_33534_PI428871386	ENST00000566956.1	2.50	6.78	5.18E-14	2.57E-12	antisense
A_23_P31414	ORC5	1.71	8.99	5.39E-14	2.66E-12	protein_coding
A_32_P34920	FOXD1	2.95	7.15	5.52E-14	2.72E-12	protein_coding
A_24_P190472	SLPI	-5.22	13.14	5.53E-14	2.72E-12	protein_coding
CUST_30847_PI428871386	ENST00000557532.1	-2.47	7.48	5.53E-14	2.72E-12	lincRNA
A_23_P326319	C16orf45	-2.18	8.51	5.56E-14	2.74E-12	protein_coding
A_33_P3230176	EPT1	2.55	6.44	5.57E-14	2.74E-12	protein_coding
A_23_P214950	PERP	3.48	12.62	5.64E-14	2.77E-12	protein_coding
CUST_30845_PI428871386	ENST00000557661.1	-2.61	7.76	5.65E-14	2.78E-12	lincRNA
A_24_P945228	CYP4V2	-1.99	6.40	5.69E-14	2.79E-12	protein_coding
A_23_P213518	CAST	-2.95	11.31	5.70E-14	2.80E-12	protein_coding
A_23_P163099	POLE2	2.53	6.38	5.72E-14	2.80E-12	protein_coding
A_23_P420942	ENST00000330439	-2.60	6.36	5.73E-14	2.80E-12	protein_coding
A_33_P3365735	THBS2	3.07	7.30	5.77E-14	2.82E-12	protein_coding
A_24_P104407	SYNM	-2.74	7.66	5.89E-14	2.88E-12	protein_coding
A_33_P3247022	CCNE2	3.41	6.71	5.99E-14	2.92E-12	protein_coding
CUST_26712_PI428871386	ENST00000547866.1	2.64	6.56	6.00E-14	2.92E-12	antisense
CUST_3286_PI428871386	ENST00000458250.1	2.85	6.12	6.00E-14	2.93E-12	lincRNA
CUST_17160_PI428871386	ENST00000422093.1	-2.50	7.89	6.04E-14	2.94E-12	antisense
CUST_31847_PI428871386	ENST00000560963.1	-1.45	5.04	6.12E-14	2.97E-12	antisense
A_33_P3254136	PKHD1L1	-2.12	5.89	6.23E-14	3.02E-12	protein_coding
A_23_P354297	CHTF18	2.57	8.90	6.27E-14	3.04E-12	protein_coding
A_33_P3413905	ADM2	1.95	6.74	6.37E-14	3.09E-12	protein_coding
A_33_P3714477	LOC285972	-1.54	5.30	6.41E-14	3.10E-12	lincRNA
A_24_P148750	SH3BP5	-2.63	8.84	6.61E-14	3.20E-12	protein_coding
CUST_27568_PI428871386	ENST00000551918.1	-2.19	6.86	6.75E-14	3.26E-12	antisense
CUST_22287_PI428871386	ENST00000414457.1	-1.63	5.94	6.77E-14	3.27E-12	antisense
A_24_P935491	COL3A1	4.61	10.01	7.10E-14	3.42E-12	protein_coding
A_23_P96590	GPRASP1	-2.63	7.40	7.12E-14	3.43E-12	protein_coding
CUST_8364_PI428871386	ENST00000460833.1	-1.98	6.19	7.31E-14	3.52E-12	antisense
A_23_P146830	SLC25A10	2.28	6.70	7.33E-14	3.52E-12	protein_coding
CUST_42442_PI428871386	ENST00000458178.1	-1.83	7.26	7.68E-14	3.68E-12	antisense
A_33_P3307363	LPHN2	-3.15	9.50	7.88E-14	3.77E-12	protein_coding
CUST_35414_PI428871386	ENST00000442355.2	-2.34	7.00	8.18E-14	3.91E-12	lincRNA
A_23_P397910	CBLC	2.98	5.98	8.23E-14	3.93E-12	protein_coding
A_32_P5480	CERS6	1.95	10.49	8.38E-14	3.99E-12	protein_coding
A_33_P3225046	CD34	-2.16	6.32	8.49E-14	4.04E-12	protein_coding
A_33_P3256510	KCNK12	3.11	7.29	8.62E-14	4.10E-12	protein_coding
A_23_P146187	RRS1	2.26	9.49	8.62E-14	4.10E-12	protein_coding
CUST_3668_PI428871386	ENST00000458044.1	-1.47	10.42	8.64E-14	4.10E-12	lincRNA
A_33_P3289820	IQSEC1	-2.08	10.72	8.78E-14	4.16E-12	protein_coding
CUST_23487_PI428871386	ENST00000435944.1	-1.99	6.01	8.80E-14	4.17E-12	antisense
A_23_P82775	SOX17	-3.80	8.89	8.91E-14	4.22E-12	protein_coding
A_23_P90589	MRPL44	1.91	8.28	9.90E-14	4.67E-12	protein_coding
CUST_22603_PI428871386	ENST00000435271.1	-2.15	6.32	9.99E-14	4.71E-12	antisense
CUST_13782_PI428871386	ENST00000518014.1	-2.46	7.72	1.01E-13	4.76E-12	lincRNA
CUST_31086_PI428871386	ENST00000551938.1	-2.62	7.54	1.03E-13	4.84E-12	antisense
CUST_37483_PI428871386	ENST00000581798.1	-1.59	5.54	1.04E-13	4.88E-12	lincRNA
CUST_42098_PI428871386	ENST00000439088.1	-1.73	5.40	1.06E-13	4.97E-12	lincRNA
A_33_P3419339	GNAI3	-1.82	7.95	1.07E-13	5.00E-12	protein_coding
CUST_8366_PI428871386	ENST00000485174.1	-1.89	5.55	1.07E-13	5.03E-12	antisense
A_23_P502312	CD97	-3.38	11.42	1.08E-13	5.03E-12	protein_coding
CUST_35433_PI428871386	ENST00000495691.2	-2.40	6.88	1.12E-13	5.22E-12	lincRNA
A_24_P129341	AKR1B10	5.22	7.12	1.12E-13	5.24E-12	protein_coding

A_33_P3270657	FAM111B	2.70	7.31	1.13E-13	5.28E-12	protein_coding
A_33_P3318746	CNIH4	-1.77	8.69	1.13E-13	5.28E-12	protein_coding
A_33_P3330952	ATP8A1	-3.96	10.42	1.14E-13	5.30E-12	protein_coding
A_23_P162579	HSPB8	-3.05	7.93	1.16E-13	5.40E-12	protein_coding
CUST_20293_PI428871386	ENST00000534626.1	1.63	5.23	1.16E-13	5.40E-12	protein_coding
A_33_P3806721	JMJD5	-1.58	8.75	1.22E-13	5.69E-12	protein_coding
A_23_P16166	PNPLA6	-2.13	7.83	1.23E-13	5.71E-12	protein_coding
CUST_9724_PI428871386	ENST00000439074.1	-1.79	5.28	1.26E-13	5.83E-12	lincRNA
CUST_20261_PI428871386	ENST00000524499.1	2.34	7.11	1.26E-13	5.85E-12	antisense
CUST_18604_PI428871386	ENST00000517521.1	-3.06	7.45	1.26E-13	5.86E-12	antisense
CUST_43763_PI428871386	ENST00000449111.1	-1.30	5.13	1.27E-13	5.87E-12	antisense
A_23_P254507	HOPX	-4.30	12.43	1.27E-13	5.89E-12	protein_coding
A_33_P3228325	SP100	-1.91	9.32	1.29E-13	5.93E-12	protein_coding
CUST_20294_PI428871386	ENST00000534626.1	1.72	5.33	1.30E-13	5.98E-12	protein_coding
CUST_30838_PI428871386	ENST00000554441.1	-2.36	7.50	1.30E-13	5.98E-12	lincRNA
A_23_P55020	CD300LF	-2.93	7.42	1.32E-13	6.07E-12	protein_coding
CUST_35432_PI428871386	ENST00000495691.2	-2.46	7.02	1.32E-13	6.09E-12	lincRNA
CUST_30848_PI428871386	ENST00000554694.1	-2.44	7.46	1.34E-13	6.14E-12	lincRNA
A_23_P207280	APPBP2	-1.88	9.55	1.34E-13	6.14E-12	protein_coding
A_23_P502350	RFX2	-2.98	7.97	1.36E-13	6.22E-12	protein_coding
A_23_P70318	ENPP4	-3.17	9.69	1.37E-13	6.25E-12	protein_coding
A_23_P398854	DOK7	-2.44	7.97	1.38E-13	6.29E-12	protein_coding
A_23_P361419	DEPDC1B	2.95	5.73	1.40E-13	6.37E-12	protein_coding
A_33_P3867534	MCM10	2.55	5.68	1.40E-13	6.39E-12	protein_coding
CUST_13262_PI428871386	ENST00000413221.2	-1.83	7.44	1.41E-13	6.42E-12	lincRNA
CUST_6252_PI428871386	ENST00000428623.1	-1.78	6.12	1.42E-13	6.45E-12	antisense
CUST_6962_PI428871386	ENST00000449346.1	-3.33	7.19	1.43E-13	6.50E-12	antisense
A_23_P28733	RBL1	1.94	6.68	1.43E-13	6.50E-12	protein_coding
A_23_P154037	AOX1	-3.62	7.49	1.44E-13	6.56E-12	protein_coding
CUST_41173_PI428871386	ENST00000425497.1	-2.15	6.39	1.45E-13	6.60E-12	lincRNA
A_23_P57709	PCOLCE2	-4.01	9.49	1.47E-13	6.67E-12	protein_coding
A_33_P3389153	STK10	-2.50	9.33	1.48E-13	6.71E-12	protein_coding
A_33_P3280094	LRFN4	2.17	10.28	1.49E-13	6.74E-12	protein_coding
CUST_15527_PI428871386	ENST00000314481.3	-2.29	5.89	1.49E-13	6.76E-12	antisense
A_24_P753161	BMPR2	-2.09	7.97	1.49E-13	6.76E-12	protein_coding
A_23_P403588	USP47	-1.92	7.33	1.51E-13	6.80E-12	protein_coding
A_24_P810290	PPAPDC1A	3.32	5.96	1.51E-13	6.82E-12	protein_coding
A_23_P140725	IFT140	-1.64	7.30	1.51E-13	6.83E-12	protein_coding
CUST_22092_PI428871386	ENST00000428853.1	-1.59	5.46	1.60E-13	7.21E-12	lincRNA
A_33_P3221960	IL18RAP	-1.78	8.13	1.60E-13	7.21E-12	protein_coding
CUST_32487_PI428871386	ENST00000558334.1	-1.93	6.73	1.64E-13	7.35E-12	antisense
A_33_P3337931	PDE4DIP	-2.16	8.72	1.66E-13	7.43E-12	protein_coding
A_33_P3231923	ENST00000409590	-1.55	9.24	1.68E-13	7.55E-12	lincRNA
A_23_P252335	MIS18A	2.13	8.88	1.70E-13	7.63E-12	protein_coding
A_23_P340848	PTGIR	-2.50	8.16	1.72E-13	7.69E-12	protein_coding
A_24_P209455	GIMAP4	-2.85	9.65	1.72E-13	7.71E-12	protein_coding
A_33_P3260605	CTNNAL1	-2.63	10.93	1.73E-13	7.73E-12	protein_coding
A_24_P406060	RNF144B	-3.08	8.93	1.75E-13	7.83E-12	protein_coding
CUST_21975_PI428871386	ENST00000543008.1	3.46	5.73	1.76E-13	7.83E-12	antisense
A_33_P3242503	ZNF365	-2.82	6.02	1.77E-13	7.90E-12	protein_coding
CUST_1228_PI428871386	ENST00000418244.1	3.96	6.36	1.82E-13	8.10E-12	antisense
CUST_30841_PI428871386	ENST00000554735.1	-2.52	7.68	1.82E-13	8.10E-12	lincRNA
A_23_P328259	FHDC1	-2.13	6.16	1.83E-13	8.13E-12	protein_coding
A_23_P31315	CBX3	1.96	11.76	1.83E-13	8.14E-12	protein_coding
A_23_P165061	AES	-2.48	12.38	1.87E-13	8.29E-12	protein_coding
A_23_P80098	GART	2.03	9.79	1.87E-13	8.30E-12	protein_coding
A_33_P3275751	SEC14L1	-2.56	10.71	1.90E-13	8.40E-12	protein_coding
A_33_P3412087	C6orf97	-3.42	7.74	1.92E-13	8.48E-12	protein_coding
A_23_P428129	CDKN1C	-3.00	11.60	1.93E-13	8.52E-12	protein_coding
CUST_36844_PI428871386	ENST00000443037.1	-2.81	10.04	1.93E-13	8.53E-12	lincRNA
CUST_30842_PI428871386	ENST00000555174.1	-2.40	7.45	1.97E-13	8.71E-12	lincRNA
A_24_P133488	CDCA4	2.50	6.32	1.98E-13	8.72E-12	protein_coding
A_23_P369899	TMEM158	3.46	6.92	1.99E-13	8.78E-12	protein_coding

A_32_P114574	CACYBP	2.12	9.93	2.01E-13	8.87E-12	protein_coding
CUST_6961_PI428871386	ENST00000449346.1	-3.15	7.22	2.03E-13	8.92E-12	antisense
CUST_16778_PI428871386	ENST00000458087.2	-1.86	5.69	2.05E-13	9.00E-12	lincRNA
CUST_17166_PI428871386	ENST00000424477.1	-2.42	7.83	2.07E-13	9.09E-12	antisense
CUST_15113_PI428871386	ENST00000450998.1	-1.97	7.49	2.08E-13	9.14E-12	antisense
A_23_P9603	PRKDC	2.31	7.67	2.10E-13	9.20E-12	protein_coding
CUST_8751_PI428871386	ENST00000462528.1	-1.72	5.39	2.13E-13	9.32E-12	lincRNA
A_23_P361773	CCND3	-2.38	9.59	2.14E-13	9.35E-12	protein_coding
CUST_34632_PI428871386	ENST00000599841.1	-1.82	5.35	2.20E-13	9.63E-12	lincRNA
A_32_P71788	FKBP4	2.66	6.86	2.22E-13	9.71E-12	protein_coding
A_23_P23171	EIF2C4	-1.46	7.62	2.24E-13	9.76E-12	protein_coding
A_23_P134851	DOK2	-2.62	7.13	2.31E-13	1.01E-11	protein_coding
A_24_P932418	AP2A2	-1.87	10.11	2.35E-13	1.02E-11	protein_coding
CUST_3567_PI428871386	ENST00000436334.1	2.34	6.03	2.36E-13	1.03E-11	lincRNA
A_33_P3224070	CSRNP1	-2.94	7.82	2.44E-13	1.06E-11	protein_coding
A_23_P348253	CDHR3	-4.20	6.85	2.45E-13	1.06E-11	protein_coding
A_23_P111452	AGAP3	-1.84	11.30	2.49E-13	1.08E-11	protein_coding
CUST_25444_PI428871386	ENST00000561746.1	-1.34	7.38	2.52E-13	1.09E-11	lincRNA
CUST_35057_PI428871386	ENST00000443997.1	-1.60	6.27	2.57E-13	1.11E-11	antisense
A_24_P133905	CCL23	-3.09	7.31	2.58E-13	1.11E-11	protein_coding
CUST_22602_PI428871386	ENST00000435271.1	-2.33	6.71	2.63E-13	1.13E-11	antisense
CUST_1226_PI428871386	ENST00000426393.1	3.97	6.34	2.66E-13	1.15E-11	antisense
CUST_30844_PI428871386	ENST00000557661.1	-2.34	7.44	2.71E-13	1.17E-11	lincRNA
A_23_P200741	DPT	-3.15	10.32	2.76E-13	1.19E-11	protein_coding
A_33_P3585268	GNAI2	-2.25	14.71	2.76E-13	1.19E-11	protein_coding
CUST_11631_PI428871386	ENST00000504509.1	-2.91	6.25	2.87E-13	1.24E-11	lincRNA
CUST_30651_PI428871386	ENST00000555595.1	-1.94	5.75	2.98E-13	1.28E-11	lincRNA
CUST_13785_PI428871386	ENST00000412431.2	-1.65	9.30	3.06E-13	1.31E-11	protein_coding
A_23_P4592	SIGLEC6	-1.38	6.04	3.09E-13	1.33E-11	protein_coding
A_33_P3398156	CYS1	-3.39	7.81	3.18E-13	1.36E-11	protein_coding
A_33_P3700794	METTL1	1.90	7.69	3.24E-13	1.39E-11	protein_coding
CUST_23873_PI428871386	ENST00000534291.1	-1.71	6.57	3.26E-13	1.39E-11	lincRNA
CUST_43062_PI428871386	ENST00000380711.3	2.04	7.73	3.32E-13	1.42E-11	antisense
CUST_15952_PI428871386	ENST00000419134.1	-1.46	6.58	3.37E-13	1.44E-11	antisense
CUST_35417_PI428871386	ENST00000435028.2	-2.36	6.79	3.40E-13	1.45E-11	lincRNA
A_23_P152428	MARVELD3	2.48	6.50	3.42E-13	1.46E-11	protein_coding
CUST_30836_PI428871386	ENST00000556266.1	-2.32	7.42	3.44E-13	1.47E-11	lincRNA
A_24_P344416	DSC3	4.24	5.83	3.44E-13	1.47E-11	protein_coding
A_33_P3362900	LOC375190	-1.95	8.67	3.55E-13	1.51E-11	protein_coding
CUST_42441_PI428871386	ENST00000458178.1	-1.77	7.12	3.55E-13	1.51E-11	antisense
A_33_P3260614	PLCB2	-2.57	10.18	3.64E-13	1.55E-11	protein_coding
A_23_P146512	GOLM1	2.80	8.35	3.72E-13	1.58E-11	protein_coding
CUST_15114_PI428871386	ENST00000450998.1	-2.05	7.32	3.72E-13	1.58E-11	antisense
CUST_35431_PI428871386	ENST00000460772.2	-2.49	7.01	3.73E-13	1.58E-11	lincRNA
A_33_P3257708	APOA1BP	2.06	10.75	3.76E-13	1.59E-11	protein_coding
A_23_P141730	DSG2	2.17	7.44	3.78E-13	1.60E-11	protein_coding
A_23_P162087	TMEM9B	-1.92	12.15	3.79E-13	1.60E-11	protein_coding
CUST_26863_PI428871386	ENST00000548347.1	-2.27	5.94	3.79E-13	1.60E-11	antisense
CUST_7807_PI428871386	ENST00000424349.1	-1.75	10.11	3.81E-13	1.61E-11	antisense
A_23_P7412	BTNL8	-2.44	6.22	3.91E-13	1.65E-11	protein_coding
CUST_41177_PI428871386	ENST00000422459.1	-2.07	6.44	3.98E-13	1.68E-11	lincRNA
A_24_P319923	MYLK	-3.24	11.18	3.99E-13	1.68E-11	protein_coding
A_33_P3284029	CSE1L	2.22	9.99	4.13E-13	1.74E-11	protein_coding
CUST_30025_PI428871386	ENST00000500036.2	-1.74	13.68	4.13E-13	1.74E-11	antisense
A_32_P152437	AKAP12	-3.54	8.49	4.20E-13	1.76E-11	protein_coding
A_23_P423309	PCDH12	-2.55	7.15	4.35E-13	1.82E-11	protein_coding
A_23_P131208	NR4A2	-3.52	8.94	4.41E-13	1.85E-11	protein_coding
CUST_23753_PI428871386	ENST00000533920.1	2.35	6.99	4.48E-13	1.88E-11	lincRNA
A_23_P201319	DISP1	-2.87	8.37	4.49E-13	1.88E-11	protein_coding
CUST_26862_PI428871386	ENST00000548347.1	-2.26	6.49	4.50E-13	1.88E-11	antisense
A_23_P501722	TSPAN32	-1.91	6.67	4.57E-13	1.91E-11	protein_coding
CUST_1232_PI428871386	ENST00000431294.1	3.83	6.29	4.60E-13	1.92E-11	antisense
A_23_P374844	GAL	4.53	6.88	4.64E-13	1.93E-11	protein_coding

A_24_P29975	ARPC5L	1.48	10.69	4.64E-13	1.93E-11	protein_coding
A_24_P88565	SLC50A1	2.12	8.32	4.69E-13	1.95E-11	protein_coding
A_23_P31816	DEFA3	-5.38	8.59	4.72E-13	1.96E-11	protein_coding
CUST_6654_PI428871386	ENST00000426615.2	4.85	6.67	4.80E-13	1.99E-11	lincRNA
A_33_P3234855	UTRN	-1.57	7.64	4.95E-13	2.05E-11	protein_coding
A_23_P138461	C10orf2	1.95	6.30	4.96E-13	2.06E-11	protein_coding
A_23_P211167	C21orf2	-1.69	6.11	4.98E-13	2.06E-11	protein_coding
CUST_26713_PI428871386	ENST00000547866.1	2.56	6.49	4.98E-13	2.06E-11	antisense
A_32_P209208	CAPS2	-2.68	7.37	5.01E-13	2.07E-11	protein_coding
A_32_P107029	NAPSA	-5.10	11.72	5.04E-13	2.09E-11	protein_coding
A_23_P145134	FGFR1OP	2.17	7.89	5.06E-13	2.09E-11	protein_coding
CUST_32816_PI428871386	ENST00000595428.1	-1.28	6.55	5.46E-13	2.25E-11	lincRNA
A_33_P3308446	RHOB	-2.70	9.69	5.50E-13	2.27E-11	protein_coding
CUST_7682_PI428871386	ENST00000600323.1	-4.45	7.23	5.57E-13	2.30E-11	antisense
CUST_41174_PI428871386	ENST00000425497.1	-2.06	6.42	5.64E-13	2.32E-11	lincRNA
CUST_16781_PI428871386	ENST00000430518.1	-1.79	5.78	5.69E-13	2.34E-11	lincRNA
CUST_12310_PI428871386	ENST00000509629.1	-1.66	5.47	5.75E-13	2.37E-11	lincRNA
CUST_34260_PI428871386	ENST00000574178.1	-2.53	5.65	5.80E-13	2.38E-11	lincRNA
CUST_12056_PI428871386	ENST00000507444.1	-1.57	5.25	5.83E-13	2.40E-11	lincRNA
CUST_17165_PI428871386	ENST00000448195.1	-2.36	7.61	5.86E-13	2.41E-11	antisense
CUST_17163_PI428871386	ENST00000451809.1	-1.97	7.07	5.87E-13	2.41E-11	antisense
CUST_1231_PI428871386	ENST00000449386.1	4.01	6.28	5.88E-13	2.41E-11	antisense
A_23_P18422	MRPL3	1.85	10.50	5.89E-13	2.41E-11	protein_coding
A_23_P18213	DNAH1	-1.58	5.96	5.97E-13	2.45E-11	protein_coding
A_33_P3251703	CRIP1	-3.67	14.52	5.98E-13	2.45E-11	protein_coding
A_23_P169428	TRUB2	1.69	9.18	6.02E-13	2.46E-11	protein_coding
CUST_23750_PI428871386	ENST00000528245.1	2.38	7.04	6.10E-13	2.49E-11	lincRNA
CUST_21998_PI428871386	ENST00000448685.1	-2.25	7.69	6.13E-13	2.50E-11	antisense
CUST_18608_PI428871386	ENST00000519753.1	-3.17	7.65	6.17E-13	2.52E-11	antisense
A_33_P3251989	CRIPT	1.54	8.79	6.18E-13	2.52E-11	protein_coding
A_23_P204736	GPD1	-2.40	6.71	6.21E-13	2.53E-11	protein_coding
CUST_7679_PI428871386	ENST00000450920.1	-4.43	7.16	6.34E-13	2.58E-11	antisense
CUST_15115_PI428871386	ENST00000587397.1	-1.99	6.98	6.56E-13	2.67E-11	antisense
A_23_P59388	DST	3.72	7.15	6.60E-13	2.68E-11	protein_coding
A_33_P3276455	KDM5B	2.22	8.08	6.61E-13	2.69E-11	protein_coding
A_23_P137935	MNDA	-3.14	8.73	6.67E-13	2.71E-11	protein_coding
CUST_38632_PI428871386	ENST00000592758.1	-2.12	9.29	6.69E-13	2.71E-11	antisense
A_33_P3333317	OPTN	-1.96	9.30	6.77E-13	2.74E-11	protein_coding
A_23_P147388	KIF13B	-2.65	10.47	6.86E-13	2.78E-11	protein_coding
CUST_26307_PI428871386	ENST00000538231.1	-1.95	6.10	6.88E-13	2.78E-11	antisense
CUST_36845_PI428871386	ENST00000443037.1	-2.72	10.03	6.91E-13	2.79E-11	lincRNA
A_23_P77000	VASH1	-2.41	10.29	7.01E-13	2.83E-11	protein_coding
A_33_P3805090	FNIP2	-2.45	9.60	7.07E-13	2.86E-11	protein_coding
A_23_P48109	NINJ2	-2.54	8.64	7.13E-13	2.87E-11	protein_coding
CUST_17170_PI428871386	ENST00000448636.1	-2.41	7.83	7.17E-13	2.89E-11	antisense
CUST_6808_PI428871386	ENST00000431752.1	-1.47	5.77	7.24E-13	2.91E-11	antisense
CUST_17698_PI428871386	ENST00000432045.1	-1.74	5.91	7.31E-13	2.94E-11	lincRNA
CUST_9934_PI428871386	ENST00000570130.1	2.23	9.06	7.35E-13	2.95E-11	lincRNA
A_23_P83175	PTPLAD2	-2.69	7.99	7.35E-13	2.95E-11	protein_coding
CUST_23058_PI428871386	ENST00000413722.1	1.95	8.80	7.41E-13	2.97E-11	antisense
CUST_20874_PI428871386	ENST00000420855.1	-1.59	5.62	7.45E-13	2.99E-11	antisense
A_32_P35947	LOC728763	-2.68	6.23	7.50E-13	3.01E-11	protein_coding
CUST_42956_PI428871386	ENST00000420096.1	2.17	6.46	7.54E-13	3.02E-11	lincRNA
A_24_P394698	ORC5	1.73	7.00	7.71E-13	3.08E-11	protein_coding
A_24_P140475	SORBS2	-2.11	6.69	7.89E-13	3.15E-11	protein_coding
CUST_20232_PI428871386	ENST00000533004.1	2.30	6.45	7.89E-13	3.15E-11	lincRNA
A_24_P185117	RILP	-1.89	7.93	7.94E-13	3.17E-11	protein_coding
A_33_P3278313	MSRB3	-3.33	8.99	8.01E-13	3.19E-11	protein_coding
A_33_P3307197	PTGFRN	2.25	8.82	8.04E-13	3.20E-11	protein_coding
A_23_P215751	NDUFA5	-1.45	11.17	8.50E-13	3.38E-11	protein_coding
CUST_1442_PI428871386	ENST00000469312.2	-1.88	6.34	8.55E-13	3.40E-11	lincRNA
A_23_P216355	TONSL	2.08	6.07	8.64E-13	3.43E-11	protein_coding
A_23_P206920	MYH11	-3.98	11.51	8.77E-13	3.48E-11	protein_coding

A_23_P80773	SRPRB	1.85	10.40	8.78E-13	3.48E-11	protein_coding
CUST_43583_PI428871386	ENST00000555831.1	-2.08	5.91	8.81E-13	3.49E-11	antisense
CUST_37482_PI428871386	ENST00000581798.1	-1.42	5.50	8.84E-13	3.50E-11	lincRNA
A_33_P3290714	HS6ST2	3.58	6.20	8.94E-13	3.54E-11	protein_coding
A_24_P389415	PNMA2	-2.77	6.79	9.13E-13	3.60E-11	protein_coding
A_33_P3339100	SELP	-2.65	7.13	9.13E-13	3.60E-11	protein_coding
A_33_P3285629	DNAI2	-3.46	8.49	9.18E-13	3.62E-11	protein_coding
A_33_P3388855	LRPPRC	1.89	7.35	9.59E-13	3.78E-11	protein_coding
CUST_14092_PI428871386	ENST00000523242.1	-2.17	5.76	9.61E-13	3.78E-11	lincRNA
CUST_42100_PI428871386	ENST00000434081.1	-1.77	5.49	1.01E-12	3.98E-11	lincRNA
A_23_P148297	SH3BGRL	-2.37	10.89	1.01E-12	3.98E-11	protein_coding
CUST_23872_PI428871386	ENST00000534291.1	-1.72	6.53	1.02E-12	4.00E-11	lincRNA
CUST_35286_PI428871386	ENST00000584141.1	2.58	8.32	1.03E-12	4.06E-11	antisense
CUST_1229_PI428871386	ENST00000418244.1	3.32	5.98	1.05E-12	4.12E-11	antisense
CUST_1227_PI428871386	ENST00000426393.1	4.00	6.34	1.05E-12	4.13E-11	antisense
CUST_1230_PI428871386	ENST00000449386.1	3.87	6.26	1.06E-12	4.14E-11	antisense
CUST_34810_PI428871386	ENST00000563087.1	-1.90	7.24	1.06E-12	4.14E-11	antisense
CUST_7681_PI428871386	ENST00000600323.1	-4.54	7.31	1.07E-12	4.18E-11	antisense
A_23_P91350	GPCPD1	-2.35	10.35	1.10E-12	4.27E-11	protein_coding
A_23_P23616	PLEKHN1	2.00	6.42	1.11E-12	4.32E-11	protein_coding
CUST_40271_PI428871386	ENST00000596330.1	-1.36	5.05	1.14E-12	4.43E-11	lincRNA
CUST_6253_PI428871386	ENST00000428623.1	-1.72	6.10	1.16E-12	4.53E-11	antisense
A_33_P3388391	GJB4	2.86	5.77	1.17E-12	4.56E-11	protein_coding
A_23_P57658	HRASLS	4.36	6.45	1.19E-12	4.64E-11	protein_coding
A_33_P3315719	PLEKHH2	-2.37	7.87	1.20E-12	4.66E-11	protein_coding
CUST_7680_PI428871386	ENST00000450920.1	-4.75	7.46	1.21E-12	4.68E-11	antisense
A_24_P178503	ABCC9	-2.18	8.17	1.21E-12	4.70E-11	protein_coding
A_32_P228268	DNAH10	-3.19	6.42	1.22E-12	4.73E-11	protein_coding
A_33_P3394404	TSPAN32	-1.61	6.01	1.22E-12	4.74E-11	protein_coding
A_24_P26897	INPP5A	-1.88	9.20	1.23E-12	4.74E-11	protein_coding
CUST_15480_PI428871386	ENST00000531702.1	-1.77	6.66	1.24E-12	4.80E-11	antisense
A_23_P161918	CCDC86	1.58	11.54	1.24E-12	4.80E-11	protein_coding
CUST_5554_PI428871386	ENST00000451384.2	-1.65	6.69	1.24E-12	4.81E-11	antisense
CUST_2649_PI428871386	ENST00000598917.1	1.61	7.37	1.24E-12	4.81E-11	antisense
A_23_P145694	ASNS	2.45	11.25	1.25E-12	4.81E-11	protein_coding
A_33_P3327479	ZDHHC3	-2.17	10.35	1.26E-12	4.88E-11	protein_coding
CUST_37747_PI428871386	ENST00000584201.1	-1.81	5.90	1.30E-12	4.99E-11	lincRNA
CUST_24718_PI428871386	ENST00000543624.1	-1.30	5.48	1.32E-12	5.09E-11	antisense
A_33_P3270034	NCOA4	-1.90	13.44	1.33E-12	5.09E-11	protein_coding
A_33_P3775848	CLIC2	-3.19	9.83	1.34E-12	5.14E-11	protein_coding
A_23_P205389	MOAP1	-2.19	8.18	1.37E-12	5.24E-11	protein_coding
A_33_P3316621	BMP3	-3.42	6.93	1.37E-12	5.26E-11	protein_coding
A_24_P353619	ALPL	-3.37	7.92	1.40E-12	5.36E-11	protein_coding
CUST_34896_PI428871386	ENST00000425081.2	2.39	9.42	1.40E-12	5.37E-11	antisense
A_23_P150768	SLCO2B1	-3.68	10.60	1.41E-12	5.38E-11	protein_coding
A_23_P12199	FAM46B	-2.56	6.85	1.41E-12	5.38E-11	protein_coding
A_24_P251534	CTDSPL	-2.15	8.97	1.42E-12	5.44E-11	protein_coding
CUST_19294_PI428871386	ENST00000522183.1	4.51	5.80	1.44E-12	5.49E-11	lincRNA
A_23_P143143	ID2	-2.29	11.81	1.45E-12	5.52E-11	protein_coding
A_24_P346587	MTFR1	2.19	7.04	1.45E-12	5.52E-11	protein_coding
A_23_P109420	BMS1	1.93	9.53	1.49E-12	5.69E-11	protein_coding
A_24_P225616	RRM2	3.03	6.45	1.53E-12	5.81E-11	protein_coding
A_33_P3304242	DNAI1	-2.37	6.48	1.53E-12	5.83E-11	protein_coding
CUST_5942_PI428871386	ENST00000446595.1	-1.58	5.87	1.54E-12	5.87E-11	lincRNA
CUST_15117_PI428871386	ENST00000585882.1	-1.98	6.99	1.57E-12	5.98E-11	antisense
A_23_P2573	TMEM117	2.33	7.32	1.58E-12	5.98E-11	protein_coding
A_33_P3399980	TYRP1	-2.30	5.98	1.58E-12	5.99E-11	protein_coding
A_33_P3278220	RABEPK	-1.36	9.90	1.63E-12	6.19E-11	protein_coding
A_24_P365506	FERMT1	2.32	5.69	1.64E-12	6.20E-11	protein_coding
A_33_P3812669	GABARAPL1	-1.98	11.55	1.64E-12	6.23E-11	protein_coding
A_23_P503064	KL	-2.54	6.16	1.65E-12	6.26E-11	protein_coding
A_23_P388433	C4orf3	-2.05	11.44	1.67E-12	6.30E-11	protein_coding
A_24_P330773	CALCOCO2	-1.66	7.67	1.67E-12	6.31E-11	protein_coding

CUST_35287_PI428871386	ENST00000584141.1	2.49	8.32	1.71E-12	6.46E-11	antisense
A_23_P29394	ATP13A4	-2.27	6.38	1.72E-12	6.48E-11	protein_coding
A_32_P51848	FAM105A	-1.98	5.87	1.77E-12	6.69E-11	protein_coding
A_23_P39237	ZFP36	-4.17	13.14	1.80E-12	6.78E-11	protein_coding
A_24_P100996	ANO5	-2.35	6.17	1.82E-12	6.85E-11	protein_coding
A_23_P126584	CD5L	-2.06	5.66	1.82E-12	6.87E-11	protein_coding
A_33_P3338928	DAB2	-3.32	10.73	1.83E-12	6.87E-11	protein_coding
A_33_P3240229	CREBBP	-1.67	8.84	1.84E-12	6.90E-11	protein_coding
A_33_P3276918	FAM64A	2.82	5.78	1.85E-12	6.95E-11	protein_coding
A_24_P346855	MKI67	2.42	5.58	1.87E-12	7.02E-11	protein_coding
CUST_18607_PI428871386	ENST00000519753.1	-2.80	7.98	1.88E-12	7.03E-11	antisense
A_33_P3341429	NEXN	-2.45	8.53	1.88E-12	7.05E-11	protein_coding
A_33_P3252925	PHF17	-2.05	9.46	1.89E-12	7.06E-11	protein_coding
A_33_P3331125	SLC2A12	-1.77	5.76	1.90E-12	7.10E-11	protein_coding
A_23_P98565	MS4A14	-2.32	6.66	1.91E-12	7.14E-11	protein_coding
A_33_P3223780	LAMB2	-2.49	10.51	1.92E-12	7.18E-11	protein_coding
CUST_264_PI428871386	ENST00000321336.1	-1.99	6.31	1.96E-12	7.30E-11	antisense
CUST_1233_PI428871386	ENST00000431294.1	3.71	6.22	1.98E-12	7.40E-11	antisense
A_23_P429184	GNPNAT1	2.21	8.88	1.99E-12	7.42E-11	protein_coding
CUST_43584_PI428871386	ENST00000555831.1	-1.98	5.72	2.00E-12	7.45E-11	antisense
A_23_P1056	GPR89B	1.64	7.99	2.02E-12	7.52E-11	protein_coding
CUST_37752_PI428871386	ENST00000578741.1	-1.67	4.98	2.07E-12	7.70E-11	lincRNA
A_24_P122403	TCEB3	-1.60	8.80	2.12E-12	7.89E-11	protein_coding
CUST_18605_PI428871386	ENST00000523643.1	-2.77	7.90	2.14E-12	7.94E-11	antisense
A_33_P3223497	FRY	-1.44	5.74	2.14E-12	7.94E-11	protein_coding
A_23_P27306	COLEC12	-3.27	8.88	2.19E-12	8.10E-11	protein_coding
A_33_P3280721	WLS	-2.65	9.28	2.19E-12	8.11E-11	protein_coding
A_23_P127891	BDNF	-2.43	6.41	2.26E-12	8.34E-11	protein_coding
A_23_P17393	CSE1L	1.95	11.04	2.29E-12	8.45E-11	protein_coding
A_24_P337000	TTC7A	-1.90	9.79	2.30E-12	8.47E-11	protein_coding
CUST_6655_PI428871386	ENST00000426615.2	4.88	6.69	2.31E-12	8.52E-11	lincRNA
A_23_P80336	TOMM22	1.47	9.66	2.32E-12	8.57E-11	protein_coding
A_23_P208866	GMFG	-3.20	12.91	2.34E-12	8.63E-11	protein_coding
CUST_39939_PI428871386	ENST00000590022.1	-1.38	5.41	2.35E-12	8.64E-11	lincRNA
A_33_P3376095	SYPL2	-2.07	6.54	2.36E-12	8.68E-11	protein_coding
CUST_23748_PI428871386	ENST00000500447.1	1.82	6.35	2.41E-12	8.86E-11	lincRNA
A_24_P409330	MRPL52	1.64	10.90	2.44E-12	8.94E-11	protein_coding
CUST_10154_PI428871386	ENST00000509244.1	1.14	5.10	2.44E-12	8.97E-11	lincRNA
A_23_P32577	DACH1	-3.34	7.17	2.49E-12	9.11E-11	protein_coding
CUST_6666_PI428871386	ENST00000438143.1	-1.34	5.32	2.49E-12	9.11E-11	lincRNA
CUST_43737_PI428871386	ENST00000412882.1	-1.76	5.96	2.50E-12	9.13E-11	antisense
A_23_P215517	KLHL7	2.54	8.09	2.52E-12	9.23E-11	protein_coding
CUST_32759_PI428871386	ENST00000557962.1	-1.40	9.56	2.53E-12	9.23E-11	lincRNA
A_33_P3292769	NFAM1	-3.19	9.00	2.53E-12	9.23E-11	protein_coding
A_24_P194688	EFHA2	-2.14	6.99	2.53E-12	9.23E-11	protein_coding
CUST_27342_PI428871386	ENST00000552167.1	-1.64	5.32	2.63E-12	9.61E-11	lincRNA
A_23_P202939	APLP2	-2.34	13.02	2.64E-12	9.61E-11	protein_coding
A_23_P159390	TOPBP1	1.98	8.38	2.71E-12	9.87E-11	protein_coding
A_33_P3336103	TIGD5	1.51	6.97	2.73E-12	9.92E-11	protein_coding
A_23_P216307	RUNX1T1	-3.24	8.70	2.73E-12	9.92E-11	protein_coding
A_23_P51126	IL1RL1	-3.31	6.33	2.73E-12	9.94E-11	protein_coding
CUST_17171_PI428871386	ENST00000448636.1	-2.01	7.10	2.74E-12	9.94E-11	antisense
CUST_34813_PI428871386	ENST00000564394.1	-1.82	6.57	2.77E-12	1.01E-10	antisense
A_23_P1029	MFAP2	2.88	8.29	2.78E-12	1.01E-10	protein_coding
A_24_P37519	LZTFL1	-1.86	7.34	2.78E-12	1.01E-10	protein_coding
A_33_P3332215	MUC1	-2.79	7.25	2.79E-12	1.01E-10	protein_coding
A_33_P3281333	SNTB2	-1.78	8.84	2.80E-12	1.02E-10	protein_coding
A_23_P51187	PRKCZ	-2.09	12.02	2.83E-12	1.02E-10	protein_coding
A_23_P34915	ATF3	-3.96	12.21	2.85E-12	1.03E-10	protein_coding
CUST_15686_PI428871386	ENST00000419627.1	-1.68	5.59	2.85E-12	1.03E-10	antisense
CUST_12636_PI428871386	ENST00000514368.1	-1.54	5.48	2.86E-12	1.03E-10	lincRNA
A_23_P414281	C16orf71	-2.21	5.59	2.91E-12	1.05E-10	protein_coding
A_23_P28263	CTDSP1	-1.49	13.29	2.93E-12	1.06E-10	protein_coding

A_33_P3311371	PDLIM2	-2.23	8.59	2.94E-12	1.06E-10	protein_coding
CUST_7935_PI428871386	ENST00000455984.1	4.04	6.04	3.00E-12	1.08E-10	lincRNA
A_33_P3221408	NTNG1	-3.67	7.01	3.08E-12	1.11E-10	protein_coding
A_23_P87351	RRM1	2.09	10.72	3.12E-12	1.12E-10	protein_coding
CUST_42172_PI428871386	ENST00000447037.1	1.85	6.80	3.17E-12	1.14E-10	antisense
A_23_P315364	CXCL2	-6.01	12.34	3.20E-12	1.15E-10	protein_coding
A_23_P156025	IRX2	-3.88	10.21	3.28E-12	1.18E-10	protein_coding
A_23_P51884	PUSL1	1.53	9.80	3.30E-12	1.18E-10	protein_coding
A_23_P212458	SEC61A1	1.26	9.32	3.31E-12	1.18E-10	protein_coding
CUST_15683_PI428871386	ENST00000451017.1	-1.74	5.49	3.32E-12	1.19E-10	antisense
CUST_43473_PI428871386	ENST00000418369.1	-1.40	5.17	3.38E-12	1.21E-10	lincRNA
CUST_13566_PI428871386	ENST00000503470.1	-1.31	5.10	3.39E-12	1.21E-10	antisense
CUST_15528_PI428871386	ENST00000314481.3	-2.23	5.70	3.44E-12	1.23E-10	antisense
CUST_20231_PI428871386	ENST00000533004.1	2.38	6.56	3.46E-12	1.24E-10	lincRNA
A_33_P3884230	NFIX	-1.81	7.97	3.50E-12	1.25E-10	protein_coding
A_33_P3227443	C16orf54	-3.36	9.20	3.54E-12	1.26E-10	protein_coding
A_33_P3339361	ARHGAP11A	1.98	5.49	3.55E-12	1.27E-10	protein_coding
A_33_P3224105	C15orf23	2.38	9.89	3.56E-12	1.27E-10	protein_coding
CUST_9935_PI428871386	ENST00000570130.1	2.26	8.71	3.56E-12	1.27E-10	lincRNA
CUST_29154_PI428871386	ENST00000439299.1	-2.37	5.89	3.60E-12	1.28E-10	antisense
A_24_P675386	C11orf58	-1.56	8.30	3.62E-12	1.28E-10	protein_coding
CUST_19296_PI428871386	ENST00000521147.1	4.58	5.81	3.67E-12	1.30E-10	lincRNA
A_33_P3393971	PKP1	3.82	6.05	3.77E-12	1.34E-10	protein_coding
CUST_4765_PI428871386	ENST00000415640.1	1.98	7.82	3.93E-12	1.39E-10	antisense
A_24_P405621	NISCH	-1.75	9.45	3.99E-12	1.41E-10	protein_coding
A_23_P217384	AP1S2	-3.06	9.02	4.03E-12	1.43E-10	protein_coding
CUST_34911_PI428871386	ENST00000573127.1	-2.35	8.96	4.06E-12	1.43E-10	lincRNA
CUST_32983_PI428871386	ENST00000562166.1	-1.25	5.04	4.13E-12	1.46E-10	antisense
A_33_P3360072	RANBP3L	-2.15	6.01	4.15E-12	1.46E-10	protein_coding
A_33_P3254096	PI15	3.14	6.81	4.27E-12	1.51E-10	protein_coding
CUST_8363_PI428871386	ENST00000466225.2	-1.47	5.40	4.28E-12	1.51E-10	antisense
A_23_P146134	DUSP26	-2.83	7.72	4.30E-12	1.51E-10	protein_coding
A_23_P397969	FOXK2	1.63	9.21	4.32E-12	1.52E-10	protein_coding
A_33_P3232011	RAB17	-3.28	10.18	4.33E-12	1.52E-10	protein_coding
A_33_P3335386	FAM83G	2.13	9.80	4.35E-12	1.53E-10	protein_coding
CUST_16542_PI428871386	ENST00000451264.1	-2.72	6.58	4.37E-12	1.53E-10	antisense
A_32_P95067	AK4	1.76	7.06	4.37E-12	1.54E-10	protein_coding
A_23_P204503	PRKAB1	-1.85	8.28	4.38E-12	1.54E-10	protein_coding
A_32_P116206	RELL1	-1.89	10.19	4.41E-12	1.55E-10	protein_coding
A_23_P23356	RRP15	1.68	8.03	4.43E-12	1.55E-10	protein_coding
A_23_P211631	FBLN1	-3.50	13.18	4.48E-12	1.57E-10	protein_coding
A_23_P21207	UBA7	-2.24	8.42	4.53E-12	1.59E-10	protein_coding
A_23_P77731	CRYM	-3.77	9.20	4.54E-12	1.59E-10	protein_coding
A_24_P184799	COCH	3.46	6.03	4.54E-12	1.59E-10	protein_coding
A_33_P3489646	SPI1	-2.84	10.14	4.71E-12	1.64E-10	protein_coding
A_23_P502425	MRPL47	2.20	11.11	4.75E-12	1.65E-10	protein_coding
CUST_26587_PI428871386	ENST00000417422.1	-2.27	6.08	4.76E-12	1.66E-10	lincRNA
A_33_P3338116	LAMB2	-2.28	12.02	4.79E-12	1.67E-10	protein_coding
A_33_P3393135	ENST00000358739	2.44	5.46	4.84E-12	1.68E-10	protein_coding
A_33_P3344332	RPL39	1.76	12.83	4.84E-12	1.68E-10	protein_coding
CUST_5584_PI428871386	ENST00000597654.1	-1.79	6.77	4.94E-12	1.71E-10	antisense
A_32_P8551	NRN1L	-1.69	6.40	4.95E-12	1.72E-10	protein_coding
A_33_P3878964	NAA25	1.78	7.28	5.03E-12	1.74E-10	protein_coding
A_24_P113144	ATAD5	2.15	5.75	5.14E-12	1.78E-10	protein_coding
A_23_P168629	RBM28	1.48	8.79	5.14E-12	1.78E-10	protein_coding
A_23_P152087	FAM82A2	-2.32	10.87	5.16E-12	1.78E-10	protein_coding
CUST_1234_PI428871386	ENST00000427268.1	3.73	6.28	5.16E-12	1.79E-10	antisense
CUST_1235_PI428871386	ENST00000427268.1	3.78	6.30	5.21E-12	1.80E-10	antisense
A_23_P325924	FAM59B	1.73	6.73	5.39E-12	1.86E-10	protein_coding
A_33_P3368750	PAQR5	-3.28	9.07	5.40E-12	1.86E-10	protein_coding
A_23_P40866	ZBTB20	-2.27	8.39	5.44E-12	1.87E-10	protein_coding
A_33_P3245321	CENPP	2.16	7.39	5.47E-12	1.88E-10	protein_coding
A_33_P3407780	PPM1F	-2.61	11.77	5.49E-12	1.89E-10	protein_coding

CUST_32758_PI428871386	ENST00000557962.1	-1.47	9.65	5.53E-12	1.90E-10	lincRNA
A_23_P37892	GPT2	3.27	7.08	5.69E-12	1.96E-10	protein_coding
CUST_37107_PI428871386	ENST00000576963.1	2.61	5.73	5.69E-12	1.96E-10	lincRNA
CUST_41178_PI428871386	ENST00000422459.1	-1.97	6.38	5.75E-12	1.97E-10	lincRNA
A_33_P3328365	ENST00000430181	-1.59	6.34	5.76E-12	1.98E-10	antisense
CUST_7814_PI428871386	ENST00000420195.1	-1.77	7.22	5.89E-12	2.02E-10	antisense
CUST_43061_PI428871386	ENST00000380711.3	1.96	7.47	5.96E-12	2.04E-10	antisense
A_23_P88691	CHRNA5	3.52	6.26	6.00E-12	2.06E-10	protein_coding
CUST_23181_PI428871386	ENST00000451737.1	-1.55	5.23	6.01E-12	2.06E-10	antisense
A_32_P15512	C1orf194	-4.63	7.92	6.01E-12	2.06E-10	protein_coding
A_24_P71973	KDR	-2.97	7.93	6.03E-12	2.06E-10	protein_coding
A_24_P925062	MXRA7	-1.94	8.75	6.06E-12	2.07E-10	protein_coding
A_23_P386384	C1orf87	-2.84	6.43	6.06E-12	2.07E-10	protein_coding
CUST_17153_PI428871386	ENST00000429408.1	-2.48	8.95	6.11E-12	2.09E-10	antisense
CUST_22290_PI428871386	ENST00000446807.1	-1.56	5.39	6.14E-12	2.09E-10	antisense
A_33_P3211929	RCOR2	3.15	7.47	6.15E-12	2.09E-10	protein_coding
A_23_P204079	NPFF	-2.15	8.98	6.15E-12	2.09E-10	protein_coding
A_33_P3271651	HLA-DPB1	-3.51	12.56	6.18E-12	2.10E-10	protein_coding
CUST_36397_PI428871386	ENST00000506504.3	1.54	6.29	6.25E-12	2.13E-10	antisense
A_33_P3227209	PA2G4	1.46	12.49	6.27E-12	2.13E-10	protein_coding
A_33_P3363933	FCRL6	-2.09	7.30	6.39E-12	2.17E-10	protein_coding
CUST_7747_PI428871386	ENST00000538717.1	-1.27	5.54	6.41E-12	2.18E-10	antisense
A_33_P3725227	COBL	-3.04	7.59	6.48E-12	2.20E-10	protein_coding
A_33_P3227375	THBS2	3.78	10.28	6.58E-12	2.23E-10	protein_coding
A_33_P3216955	ANAPC16	-1.51	8.16	6.63E-12	2.25E-10	protein_coding
A_23_P333683	IGSF10	-2.49	6.52	6.89E-12	2.33E-10	protein_coding
CUST_37305_PI428871386	ENST00000580756.1	-1.63	5.46	6.92E-12	2.34E-10	lincRNA
A_24_P106112	PKD2	-1.95	8.76	6.95E-12	2.35E-10	protein_coding
A_33_P3784253	PAK1	1.97	9.64	7.03E-12	2.37E-10	protein_coding
A_33_P3333360	C16orf5	-1.41	8.51	7.26E-12	2.45E-10	protein_coding
A_23_P63067	DAP3	1.65	12.96	7.26E-12	2.45E-10	protein_coding
A_33_P3212823	LOC100132247	-1.94	15.55	7.27E-12	2.45E-10	protein_coding
A_24_P373174	RAB27A	-2.56	8.08	7.29E-12	2.46E-10	protein_coding
CUST_8367_PI428871386	ENST00000485174.1	-1.69	5.60	7.36E-12	2.48E-10	antisense
A_24_P153456	ZDHHC11	-3.57	8.71	7.41E-12	2.49E-10	protein_coding
CUST_22194_PI428871386	ENST00000423551.1	-1.60	6.14	7.44E-12	2.50E-10	antisense
A_32_P83784	ARAP2	-2.25	8.60	7.46E-12	2.51E-10	protein_coding
A_24_P257348	ARL6IP5	-2.12	10.66	7.48E-12	2.51E-10	protein_coding
CUST_12471_PI428871386	ENST00000508123.1	-1.64	5.67	7.52E-12	2.52E-10	antisense
A_33_P3243439	GPR162	-2.44	9.05	7.54E-12	2.53E-10	protein_coding
CUST_24719_PI428871386	ENST00000543624.1	-1.52	5.33	7.58E-12	2.54E-10	antisense
CUST_1909_PI428871386	ENST00000448680.1	-2.32	5.78	7.67E-12	2.56E-10	lincRNA
A_23_P71727	CKS2	2.36	11.09	7.79E-12	2.60E-10	protein_coding
CUST_33662_PI428871386	ENST00000565152.1	-1.34	5.45	7.79E-12	2.60E-10	antisense
CUST_1596_PI428871386	ENST00000438509.1	-1.88	7.14	7.83E-12	2.61E-10	antisense
CUST_31087_PI428871386	ENST00000551938.1	-2.24	7.69	7.83E-12	2.61E-10	antisense
A_24_P65098	TMEM87A	-1.43	6.38	8.10E-12	2.70E-10	protein_coding
A_23_P157404	AP1S1	1.72	8.18	8.17E-12	2.72E-10	protein_coding
A_23_P88234	FAM158A	1.78	8.65	8.35E-12	2.78E-10	protein_coding
A_24_P346431	TNS3	-2.61	9.79	8.36E-12	2.78E-10	protein_coding
CUST_22672_PI428871386	ENST00000425290.1	-1.14	4.94	8.42E-12	2.80E-10	antisense
A_23_P116264	NRGN	-3.34	9.27	8.60E-12	2.86E-10	protein_coding
A_24_P61490	NKX2-1	-3.28	10.65	8.64E-12	2.87E-10	protein_coding
CUST_32486_PI428871386	ENST00000558334.1	-1.84	6.94	8.76E-12	2.90E-10	antisense
A_23_P99292	RAD51AP1	2.28	5.76	8.77E-12	2.90E-10	protein_coding
CUST_31905_PI428871386	ENST00000583044.1	-1.73	6.08	8.80E-12	2.91E-10	antisense
A_23_P151405	CKAP2	2.36	8.21	8.86E-12	2.93E-10	protein_coding
A_23_P20832	SPTAN1	-1.61	10.92	8.98E-12	2.97E-10	protein_coding
A_24_P276932	ATP6V1C2	1.85	6.76	9.04E-12	2.98E-10	protein_coding
A_23_P152234	CMTM2	-2.98	6.19	9.12E-12	3.01E-10	protein_coding
A_23_P382043	NT5DC1	-1.68	9.31	9.22E-12	3.04E-10	protein_coding
A_33_P3222932	SHISA8	-1.76	7.19	9.23E-12	3.04E-10	protein_coding
CUST_27569_PI428871386	ENST00000551918.1	-2.06	6.71	9.26E-12	3.05E-10	antisense

A_23_P95302	RFC5	2.12	9.26	9.26E-12	3.05E-10	protein_coding
CUST_42171_PI428871386	ENST00000447037.1	2.16	8.02	9.28E-12	3.05E-10	antisense
CUST_17154_PI428871386	ENST00000426835.1	-2.24	7.45	9.57E-12	3.15E-10	antisense
A_23_P55649	FPR2	-2.46	6.63	9.63E-12	3.16E-10	protein_coding
CUST_8736_PI428871386	ENST00000496154.1	-1.24	5.24	9.71E-12	3.18E-10	antisense
CUST_23751_PI428871386	ENST00000528245.1	2.15	6.71	9.71E-12	3.18E-10	lincRNA
CUST_34128_PI428871386	ENST00000568767.1	-2.07	6.28	9.81E-12	3.21E-10	lincRNA
A_23_P44466	CCDC102B	-2.74	9.16	9.85E-12	3.23E-10	protein_coding
CUST_7934_PI428871386	ENST00000455984.1	3.92	6.11	9.90E-12	3.24E-10	lincRNA
CUST_34913_PI428871386	ENST00000576489.1	-2.26	7.71	9.94E-12	3.25E-10	lincRNA
CUST_23752_PI428871386	ENST00000533920.1	2.16	6.74	1.00E-11	3.27E-10	lincRNA
CUST_37890_PI428871386	ENST00000573479.1	1.66	8.05	1.02E-11	3.32E-10	lincRNA
CUST_18603_PI428871386	ENST00000517521.1	-2.80	7.79	1.02E-11	3.32E-10	antisense
A_33_P3209346	IARS	1.83	7.40	1.02E-11	3.35E-10	protein_coding
CUST_19292_PI428871386	ENST00000504531.2	4.53	5.94	1.04E-11	3.38E-10	lincRNA
A_23_P331748	CD33	-3.24	8.85	1.04E-11	3.39E-10	protein_coding
A_33_P3335366	MAML3	-1.65	5.95	1.05E-11	3.43E-10	protein_coding
A_33_P3314659	SPEF2	-1.82	6.18	1.06E-11	3.44E-10	protein_coding
A_23_P146294	EFCAB1	-4.19	7.40	1.07E-11	3.49E-10	protein_coding
A_24_P160466	GPRIN1	2.14	6.28	1.08E-11	3.51E-10	protein_coding
CUST_30873_PI428871386	ENST00000559402.1	1.26	5.65	1.08E-11	3.52E-10	lincRNA
CUST_7911_PI428871386	ENST00000436123.1	1.77	10.19	1.09E-11	3.54E-10	lincRNA
A_23_P56922	HSPE1	1.80	13.44	1.10E-11	3.57E-10	protein_coding
A_23_P209337	METTL21A	2.06	8.03	1.10E-11	3.58E-10	protein_coding
CUST_25785_PI428871386	ENST00000524453.1	-1.29	5.11	1.10E-11	3.58E-10	antisense
A_32_P160045	TCTEX1D1	-1.72	5.42	1.11E-11	3.58E-10	protein_coding
A_33_P3320548	NUPL2	1.52	8.37	1.12E-11	3.62E-10	protein_coding
A_33_P3253653	GPR155	-2.10	16.87	1.12E-11	3.62E-10	protein_coding
CUST_25065_PI428871386	ENST00000533008.1	-3.07	9.51	1.12E-11	3.62E-10	antisense
A_24_P70002	LATS2	-1.93	7.42	1.12E-11	3.63E-10	protein_coding
A_23_P501831	C5orf4	-3.62	10.05	1.14E-11	3.69E-10	protein_coding
A_24_P940803	EPT1	1.90	8.68	1.16E-11	3.75E-10	protein_coding
CUST_24210_PI428871386	ENST00000525302.1	2.71	5.47	1.17E-11	3.76E-10	antisense
CUST_24706_PI428871386	ENST00000539303.1	2.26	6.78	1.17E-11	3.78E-10	lincRNA
CUST_30650_PI428871386	ENST00000555595.1	-1.69	5.73	1.18E-11	3.80E-10	lincRNA
CUST_5138_PI428871386	ENST00000423168.1	-1.45	5.44	1.19E-11	3.84E-10	lincRNA
A_32_P358887	SLC4A4	-3.28	7.87	1.20E-11	3.84E-10	protein_coding
A_24_P481783	UBAP1L	-1.49	7.51	1.20E-11	3.86E-10	protein_coding
A_24_P317708	SDR16C5	-3.27	7.40	1.21E-11	3.90E-10	protein_coding
CUST_19293_PI428871386	ENST00000522183.1	4.04	5.62	1.25E-11	4.00E-10	lincRNA
A_33_P3394710	SLC35A1	-1.71	7.76	1.27E-11	4.05E-10	protein_coding
A_24_P763243	EEF1A1	-1.57	16.34	1.29E-11	4.14E-10	protein_coding
A_33_P3388135	MKKS	1.56	9.95	1.30E-11	4.17E-10	protein_coding
A_33_P3260430	SPRR2A	3.43	7.76	1.32E-11	4.21E-10	protein_coding
A_23_P120504	C20orf46	-2.14	7.26	1.32E-11	4.21E-10	protein_coding
A_23_P218047	KRT5	2.38	8.05	1.35E-11	4.32E-10	protein_coding
A_24_P396231	LAMP2	-2.20	9.07	1.36E-11	4.33E-10	protein_coding
A_24_P301846	GART	1.68	7.23	1.36E-11	4.33E-10	protein_coding
CUST_34906_PI428871386	ENST00000571595.1	-2.39	10.25	1.36E-11	4.34E-10	lincRNA
CUST_19291_PI428871386	ENST00000504531.2	4.17	5.63	1.39E-11	4.44E-10	lincRNA
A_23_P25097	LLPH	1.60	9.00	1.40E-11	4.47E-10	protein_coding
A_23_P103465	PLA2G5	-2.08	7.27	1.40E-11	4.47E-10	protein_coding
CUST_35325_PI428871386	ENST00000480811.1	1.94	6.60	1.43E-11	4.55E-10	antisense
CUST_8917_PI428871386	ENST00000505721.1	-1.11	5.01	1.43E-11	4.56E-10	antisense
CUST_13801_PI428871386	ENST00000523781.1	-1.10	10.67	1.44E-11	4.57E-10	lincRNA
CUST_25064_PI428871386	ENST00000533008.1	-3.00	9.36	1.46E-11	4.64E-10	antisense
CUST_17269_PI428871386	ENST00000418395.1	-1.64	5.38	1.48E-11	4.69E-10	lincRNA
A_23_P381577	ZNF25	-1.36	7.11	1.48E-11	4.69E-10	protein_coding
A_23_P49279	C16orf87	1.81	8.42	1.49E-11	4.73E-10	protein_coding
A_23_P157352	MRPS33	1.76	10.45	1.50E-11	4.76E-10	protein_coding
CUST_32642_PI428871386	ENST00000559505.1	-1.49	5.84	1.54E-11	4.87E-10	antisense
CUST_36639_PI428871386	ENST00000585921.1	-1.13	5.48	1.54E-11	4.88E-10	antisense
A_23_P424316	TCF20	1.43	7.74	1.55E-11	4.91E-10	protein_coding

CUST_21976_PI428871386	ENST00000543008.1	2.81	5.57	1.57E-11	4.97E-10	antisense
A_23_P69058	MLH1	-1.37	11.09	1.58E-11	5.00E-10	protein_coding
CUST_24707_PI428871386	ENST00000539303.1	2.28	6.73	1.60E-11	5.03E-10	lincRNA
A_23_P257201	RNF146	-1.64	7.98	1.62E-11	5.12E-10	protein_coding
A_23_P30913	HLA-DPA1	-3.75	14.30	1.65E-11	5.19E-10	protein_coding
A_32_P28685	SNRPA1	1.68	9.52	1.66E-11	5.22E-10	protein_coding
CUST_29951_PI428871386	ENST00000554360.1	-1.49	8.45	1.66E-11	5.24E-10	antisense
CUST_34811_PI428871386	ENST00000563087.1	-1.58	6.90	1.67E-11	5.25E-10	antisense
A_33_P3277527	LAMC3	-2.39	6.99	1.67E-11	5.26E-10	protein_coding
A_33_P3816688	PPARGC1B	-2.49	7.51	1.68E-11	5.26E-10	protein_coding
A_24_P355267	SLC25A25	-2.43	10.11	1.68E-11	5.29E-10	protein_coding
A_24_P154948	GARS	2.07	8.47	1.68E-11	5.29E-10	protein_coding
CUST_32374_PI428871386	ENST00000527801.1	-1.31	4.99	1.69E-11	5.31E-10	antisense
CUST_37207_PI428871386	ENST00000576784.1	1.54	6.20	1.69E-11	5.31E-10	antisense
A_23_P74663	TAF1A	1.56	7.44	1.74E-11	5.45E-10	protein_coding
CUST_16543_PI428871386	ENST00000451264.1	-2.64	6.63	1.75E-11	5.48E-10	antisense
A_23_P343963	FAM83F	2.94	6.16	1.77E-11	5.54E-10	protein_coding
CUST_4755_PI428871386	ENST00000445520.1	-1.60	8.54	1.78E-11	5.55E-10	antisense
CUST_17268_PI428871386	ENST00000418395.1	-1.36	5.35	1.78E-11	5.55E-10	lincRNA
A_23_P144369	NAP1L5	-2.11	7.17	1.78E-11	5.56E-10	protein_coding
CUST_17402_PI428871386	ENST00000433514.1	-1.42	4.94	1.79E-11	5.57E-10	antisense
A_24_P42681	PSMD2	1.76	10.20	1.81E-11	5.63E-10	protein_coding
A_23_P46429	CYR61	-3.81	13.05	1.81E-11	5.64E-10	protein_coding
CUST_34907_PI428871386	ENST00000571595.1	-2.37	10.10	1.82E-11	5.65E-10	lincRNA
A_24_P335358	PUS1	1.86	7.99	1.82E-11	5.65E-10	protein_coding
A_33_P3367392	FAM167B	-2.17	8.57	1.84E-11	5.71E-10	protein_coding
A_23_P150394	FXYD6	-2.58	9.07	1.84E-11	5.71E-10	protein_coding
A_24_P133253	KITLG	-3.19	9.72	1.86E-11	5.78E-10	protein_coding
A_24_P398147	NEBL	-2.80	8.09	1.91E-11	5.92E-10	protein_coding
CUST_9465_PI428871386	ENST00000451742.1	3.61	5.94	1.92E-11	5.95E-10	antisense
A_24_P178093	TOMM40	1.96	7.31	1.95E-11	6.03E-10	protein_coding
A_23_P59718	SRI	-1.98	7.71	1.97E-11	6.10E-10	protein_coding
CUST_26583_PI428871386	ENST00000563933.1	-2.20	6.05	2.00E-11	6.19E-10	lincRNA
A_23_P37415	SECISBP2L	-1.89	8.16	2.02E-11	6.25E-10	protein_coding
A_23_P210920	GSS	1.38	10.34	2.04E-11	6.30E-10	protein_coding
A_33_P3331237	G3BP1	-1.22	6.21	2.06E-11	6.35E-10	protein_coding
A_23_P48988	SH3GL3	-2.69	6.92	2.06E-11	6.35E-10	protein_coding
A_33_P3325497	FIBIN	-2.80	7.11	2.06E-11	6.36E-10	protein_coding
A_23_P48307	PABPC3	1.76	12.83	2.07E-11	6.39E-10	protein_coding
CUST_4764_PI428871386	ENST00000415640.1	1.72	7.00	2.09E-11	6.45E-10	antisense
CUST_27535_PI428871386	ENST00000538559.2	-1.84	6.14	2.10E-11	6.45E-10	lincRNA
A_24_P84428	CACYBP	1.78	9.66	2.11E-11	6.50E-10	protein_coding
A_33_P3332406	LDLRAD1	-3.40	6.91	2.13E-11	6.55E-10	protein_coding
A_23_P39550	TMEM163	-2.42	7.38	2.15E-11	6.59E-10	protein_coding
A_23_P361049	MYO1B	-1.94	9.93	2.15E-11	6.61E-10	protein_coding
A_24_P237389	EIF1AX	-1.75	9.34	2.18E-11	6.68E-10	protein_coding
A_23_P159907	MAGED4B	3.08	7.39	2.19E-11	6.72E-10	protein_coding
A_23_P38505	CXCL16	-1.87	9.18	2.19E-11	6.72E-10	protein_coding
A_24_P73290	ATP2A2	-1.42	9.18	2.22E-11	6.80E-10	protein_coding
CUST_37206_PI428871386	ENST00000576784.1	1.57	6.29	2.22E-11	6.80E-10	antisense
CUST_8068_PI428871386	ENST00000432377.1	-1.73	6.25	2.24E-11	6.86E-10	antisense
A_24_P233917	KIAA0494	-1.81	10.23	2.25E-11	6.88E-10	protein_coding
CUST_28140_PI428871386	ENST00000537998.1	-1.51	5.55	2.25E-11	6.89E-10	lincRNA
CUST_7152_PI428871386	ENST00000416344.1	-1.43	5.31	2.27E-11	6.94E-10	antisense
A_23_P85453	CD244	-1.85	6.50	2.28E-11	6.96E-10	protein_coding
A_23_P23296	PKP1	3.80	6.16	2.30E-11	7.01E-10	protein_coding
CUST_263_PI428871386	ENST00000321336.1	-1.68	6.38	2.31E-11	7.06E-10	antisense
A_23_P170649	C8orf84	-3.26	7.36	2.34E-11	7.12E-10	protein_coding
CUST_34897_PI428871386	ENST00000425081.2	2.25	9.12	2.35E-11	7.16E-10	antisense
A_33_P3290532	LOC255480	-1.21	5.28	2.36E-11	7.16E-10	antisense
A_24_P21507	SPAG6	-2.58	5.82	2.37E-11	7.21E-10	protein_coding
CUST_34184_PI428871386	ENST00000572067.1	-1.77	6.56	2.38E-11	7.22E-10	lincRNA
A_23_P368681	GIMAP2	-2.26	8.52	2.40E-11	7.27E-10	protein_coding

CUST_43389_PI428871386	ENST00000561973.1	-1.69	5.84	2.42E-11	7.33E-10	lincRNA
A_23_P142447	MYO1F	-2.05	10.35	2.44E-11	7.40E-10	protein_coding
A_24_P48495	LYPD3	2.67	6.01	2.44E-11	7.40E-10	protein_coding
A_33_P3332018	FGL2	-2.73	7.78	2.45E-11	7.40E-10	protein_coding
CUST_8354_PI428871386	ENST00000493124.1	-1.32	4.95	2.45E-11	7.41E-10	antisense
CUST_5348_PI428871386	ENST00000439192.1	2.51	6.49	2.48E-11	7.50E-10	antisense
CUST_34915_PI428871386	ENST00000575626.1	-2.05	7.55	2.49E-11	7.51E-10	lincRNA
A_32_P101031	LYPD1	3.68	7.22	2.49E-11	7.52E-10	protein_coding
CUST_18230_PI428871386	ENST00000519726.1	-1.44	6.44	2.50E-11	7.55E-10	antisense
CUST_22292_PI428871386	ENST00000423223.1	-1.35	5.34	2.52E-11	7.59E-10	antisense
A_32_P158355	ALG1L	1.90	8.48	2.53E-11	7.61E-10	protein_coding
CUST_20641_PI428871386	ENST00000433838.1	-1.37	5.32	2.57E-11	7.72E-10	antisense
A_23_P73097	RGS20	2.93	5.84	2.58E-11	7.75E-10	protein_coding
A_24_P655849	SMAD9	-2.15	6.52	2.59E-11	7.77E-10	protein_coding
CUST_15522_PI428871386	ENST00000452675.1	2.19	5.82	2.61E-11	7.83E-10	lincRNA
CUST_19295_PI428871386	ENST00000521147.1	3.66	5.71	2.72E-11	8.16E-10	lincRNA
A_33_P3325748	TRIOBP	1.13	4.85	2.75E-11	8.24E-10	protein_coding
CUST_14609_PI428871386	ENST00000436804.1	-1.87	6.25	2.75E-11	8.25E-10	lincRNA
CUST_2627_PI428871386	ENST00000415675.2	-1.74	5.25	2.83E-11	8.47E-10	antisense
CUST_12057_PI428871386	ENST00000507444.1	-1.40	5.31	2.84E-11	8.49E-10	lincRNA
A_33_P3270489	C6orf97	-2.82	6.71	2.85E-11	8.50E-10	protein_coding
A_23_P71316	RBPMS	-2.06	8.96	2.86E-11	8.54E-10	protein_coding
A_24_P56194	CREBL2	-2.04	8.14	2.92E-11	8.71E-10	protein_coding
CUST_37106_PI428871386	ENST00000576963.1	2.38	5.42	3.00E-11	8.93E-10	lincRNA
A_33_P3251562	RSPO1	-1.74	5.48	3.07E-11	9.15E-10	protein_coding
CUST_42089_PI428871386	ENST00000430181.1	-1.51	6.36	3.08E-11	9.18E-10	antisense
CUST_41175_PI428871386	ENST00000445003.1	-2.97	9.53	3.11E-11	9.27E-10	lincRNA
CUST_15110_PI428871386	ENST00000432050.1	-1.53	5.14	3.12E-11	9.29E-10	antisense
A_33_P3236921	PARP1	1.65	8.64	3.21E-11	9.53E-10	protein_coding
CUST_7147_PI428871386	ENST00000433296.1	-1.30	5.37	3.21E-11	9.54E-10	antisense
CUST_20890_PI428871386	ENST00000432148.1	-2.23	6.38	3.21E-11	9.54E-10	lincRNA
CUST_18918_PI428871386	ENST00000523786.1	-1.40	5.30	3.25E-11	9.65E-10	lincRNA
CUST_25336_PI428871386	ENST00000542198.1	-3.98	6.82	3.27E-11	9.69E-10	lincRNA
A_24_P97342	PROK2	-3.12	6.40	3.29E-11	9.76E-10	protein_coding
A_33_P3702055	SLC11A1	-2.10	8.71	3.31E-11	9.79E-10	protein_coding
A_23_P217009	C9orf24	-5.85	9.73	3.31E-11	9.80E-10	protein_coding
A_23_P362694	C4orf7	3.50	8.44	3.31E-11	9.80E-10	protein_coding
CUST_19993_PI428871386	ENST00000521122.1	2.91	6.38	3.39E-11	1.00E-09	lincRNA
A_23_P137391	ENO1	2.27	12.56	3.42E-11	1.01E-09	protein_coding
A_23_P64611	P2RY6	2.44	8.13	3.46E-11	1.02E-09	protein_coding
A_23_P390116	SPATA13	-1.85	9.31	3.47E-11	1.02E-09	protein_coding
CUST_19298_PI428871386	ENST00000523313.1	4.18	5.75	3.47E-11	1.02E-09	lincRNA
A_23_P127522	HYLS1	1.86	7.23	3.51E-11	1.03E-09	protein_coding
CUST_3782_PI428871386	ENST00000439849.1	-1.35	4.86	3.52E-11	1.04E-09	antisense
A_33_P3521643	PPP1R12C	-1.71	12.18	3.53E-11	1.04E-09	protein_coding
CUST_4624_PI428871386	ENST00000455424.1	-1.21	5.28	3.57E-11	1.05E-09	antisense
CUST_29503_PI428871386	ENST00000558478.1	-1.22	6.41	3.57E-11	1.05E-09	lincRNA
CUST_34661_PI428871386	ENST00000600553.1	-1.69	5.78	3.62E-11	1.06E-09	lincRNA
A_23_P71415	WDYHV1	2.01	7.60	3.63E-11	1.07E-09	protein_coding
A_23_P302550	RGS18	-2.40	7.68	3.67E-11	1.08E-09	protein_coding
CUST_17159_PI428871386	ENST00000414797.1	-2.08	6.78	3.69E-11	1.08E-09	antisense
A_33_P3417745	IQGAP1	-1.68	13.01	3.70E-11	1.08E-09	protein_coding
CUST_29502_PI428871386	ENST00000558478.1	-1.39	5.99	3.76E-11	1.10E-09	lincRNA
CUST_12142_PI428871386	ENST00000561606.1	1.90	6.98	3.76E-11	1.10E-09	antisense
A_23_P50897	MKI67IP	1.84	8.76	3.77E-11	1.10E-09	protein_coding
CUST_1144_PI428871386	ENST00000419531.1	-1.31	4.96	3.80E-11	1.11E-09	lincRNA
A_24_P381029	GLRX3	1.58	10.24	3.82E-11	1.12E-09	protein_coding
A_32_P460973	HLA-E	-2.38	14.64	3.82E-11	1.12E-09	protein_coding
A_23_P205789	GABPB1	1.95	7.70	3.83E-11	1.12E-09	protein_coding
A_23_P62741	ELTD1	-2.19	7.15	3.84E-11	1.12E-09	protein_coding
A_23_P206806	ITGAL	-2.76	8.85	3.85E-11	1.12E-09	protein_coding
A_33_P3387771	USP9X	-1.71	8.21	3.86E-11	1.13E-09	protein_coding
CUST_19297_PI428871386	ENST00000523313.1	3.90	5.67	3.88E-11	1.13E-09	lincRNA

A_23_P63371	TAL1	-1.87	6.24	3.91E-11	1.14E-09	protein_coding
CUST_23687_PI428871386	ENST00000443633.1	-1.72	5.16	3.93E-11	1.14E-09	lincRNA
A_33_P3397418	ZC3HAV1	-1.23	10.65	3.94E-11	1.14E-09	protein_coding
A_33_P3327270	TMED7-TICAM2	-1.18	9.15	3.95E-11	1.15E-09	protein_coding
CUST_25337_PI428871386	ENST00000542198.1	-4.22	7.32	4.03E-11	1.17E-09	lincRNA
CUST_11381_PI428871386	ENST00000508269.1	-2.11	6.85	4.07E-11	1.18E-09	antisense
A_23_P70991	AIMP2	1.94	9.70	4.15E-11	1.20E-09	protein_coding
A_23_P423695	MXD4	-1.51	8.86	4.16E-11	1.20E-09	protein_coding
CUST_15685_PI428871386	ENST00000419627.1	-1.47	5.59	4.18E-11	1.21E-09	antisense
CUST_38907_PI428871386	ENST00000587762.1	-3.34	12.24	4.20E-11	1.21E-09	lincRNA
A_23_P333063	SMARCE1	1.30	8.18	4.24E-11	1.23E-09	protein_coding
CUST_7220_PI428871386	ENST00000562038.1	-1.84	6.66	4.24E-11	1.23E-09	lincRNA
A_32_P449380	WDR38	-2.11	6.03	4.26E-11	1.23E-09	protein_coding
CUST_1732_PI428871386	ENST00000419428.1	-1.90	6.28	4.29E-11	1.24E-09	lincRNA
CUST_15089_PI428871386	ENST00000419979.1	-2.16	7.23	4.34E-11	1.25E-09	antisense
A_23_P25403	HCFC2	-1.39	8.61	4.34E-11	1.25E-09	protein_coding
A_23_P389118	ANO6	-2.02	10.18	4.36E-11	1.25E-09	protein_coding
A_23_P349966	TMEM130	-2.83	8.62	4.46E-11	1.28E-09	protein_coding
A_33_P3361257	NOP16	1.84	9.50	4.49E-11	1.29E-09	protein_coding
A_23_P64173	CARD16	-2.69	9.56	4.51E-11	1.29E-09	protein_coding
CUST_15128_PI428871386	ENST00000589255.1	-1.62	5.50	4.55E-11	1.31E-09	antisense
A_32_P205637	PARD6B	-2.10	8.15	4.56E-11	1.31E-09	protein_coding
A_32_P355396	TECPR2	-1.92	7.40	4.59E-11	1.31E-09	protein_coding
A_24_P134195	MYADM	-1.68	8.12	4.63E-11	1.33E-09	protein_coding
A_23_P167030	PTH1R	-1.86	6.30	4.66E-11	1.33E-09	protein_coding
A_33_P3356462	C2CD4A	4.41	6.39	4.70E-11	1.34E-09	protein_coding
CUST_31271_PI428871386	ENST00000560886.1	-1.11	4.73	4.72E-11	1.35E-09	lincRNA
CUST_5943_PI428871386	ENST00000446595.1	-1.59	5.94	4.72E-11	1.35E-09	lincRNA
A_23_P87742	IFFO1	-2.69	10.40	4.77E-11	1.36E-09	protein_coding
A_33_P3272553	NCAPH2	1.61	6.96	4.77E-11	1.36E-09	protein_coding
A_23_P432610	N4BP1	-1.70	8.59	4.83E-11	1.38E-09	protein_coding
A_33_P3216933	SIK2	-1.60	7.19	4.84E-11	1.38E-09	protein_coding
CUST_30835_PI428871386	ENST00000553575.1	-1.57	5.84	4.86E-11	1.39E-09	lincRNA
CUST_31434_PI428871386	ENST00000500949.2	-1.61	11.04	4.90E-11	1.40E-09	antisense
A_23_P133293	MCTP1	-2.16	7.84	4.90E-11	1.40E-09	protein_coding
CUST_23686_PI428871386	ENST00000443633.1	-1.51	5.05	4.92E-11	1.40E-09	lincRNA
A_23_P347508	SELRC1	1.75	8.41	4.99E-11	1.42E-09	protein_coding
CUST_14825_PI428871386	ENST00000420293.1	-1.38	5.17	5.02E-11	1.43E-09	antisense
A_33_P3230254	NCAPG	1.93	5.35	5.03E-11	1.43E-09	protein_coding
A_24_P413988	TGOLN2	-1.80	11.94	5.07E-11	1.44E-09	protein_coding
A_23_P51996	STXBP3	-1.38	10.19	5.13E-11	1.45E-09	protein_coding
A_23_P60028	TCEB1	1.76	13.00	5.16E-11	1.46E-09	protein_coding
A_33_P3214199	ZNF532	1.76	7.74	5.23E-11	1.48E-09	protein_coding
A_33_P3209960	RASGRP2	-2.14	8.53	5.26E-11	1.49E-09	protein_coding
A_33_P3344204	ZDHHC11	-3.75	10.05	5.29E-11	1.50E-09	protein_coding
CUST_6630_PI428871386	ENST00000416928.2	-1.82	8.23	5.30E-11	1.50E-09	antisense
A_33_P3234277	HLA-DPA1	-3.66	13.00	5.31E-11	1.50E-09	protein_coding
A_23_P402287	LNX2	-1.79	9.33	5.32E-11	1.50E-09	protein_coding
A_33_P3249125	UNC45B	-1.38	5.22	5.33E-11	1.51E-09	protein_coding
A_33_P3310533	DNAJC21	1.35	9.81	5.39E-11	1.52E-09	protein_coding
A_33_P3366758	ST8SIA6	-1.58	5.40	5.42E-11	1.53E-09	protein_coding
CUST_16267_PI428871386	ENST00000457484.2	-1.57	6.34	5.56E-11	1.57E-09	lincRNA
A_33_P3311863	AP2A2	-1.50	6.42	5.57E-11	1.57E-09	protein_coding
A_33_P3270863	XDH	4.27	7.14	5.58E-11	1.57E-09	protein_coding
A_33_P3702364	SNX24	-1.79	10.50	5.60E-11	1.58E-09	protein_coding
A_23_P500000	SCEL	-4.01	9.18	5.63E-11	1.58E-09	protein_coding
CUST_19987_PI428871386	ENST00000522963.1	2.68	6.49	5.65E-11	1.59E-09	lincRNA
A_23_P748	IRF6	2.42	7.22	5.75E-11	1.61E-09	protein_coding
CUST_3164_PI428871386	ENST00000415582.1	2.51	6.38	5.75E-11	1.61E-09	antisense
A_23_P154500	DNMT3A	1.76	8.34	5.78E-11	1.62E-09	protein_coding
CUST_25276_PI428871386	ENST00000526206.1	-1.97	6.59	5.89E-11	1.65E-09	lincRNA
A_24_P206776	CRYAB	-2.62	9.67	5.95E-11	1.67E-09	protein_coding
A_33_P3271635	HLA-DPB1	-3.23	11.44	6.00E-11	1.68E-09	protein_coding

A_23_P29067	TMPRSS2	-3.12	8.87	6.08E-11	1.70E-09	protein_coding
A_24_P275073	ADAMTS14	1.98	6.63	6.11E-11	1.71E-09	protein_coding
A_23_P352684	DCAF5	-1.26	8.29	6.12E-11	1.71E-09	protein_coding
A_23_P73780	IRAK1	1.69	9.19	6.14E-11	1.71E-09	protein_coding
CUST_11380_PI428871386	ENST00000508269.1	-2.43	7.02	6.19E-11	1.73E-09	antisense
CUST_17720_PI428871386	ENST00000429901.1	-2.58	9.01	6.21E-11	1.73E-09	antisense
CUST_3632_PI428871386	ENST00000435574.1	-1.50	5.63	6.24E-11	1.74E-09	lincRNA
CUST_17152_PI428871386	ENST00000429408.1	-2.36	8.79	6.25E-11	1.74E-09	antisense
CUST_5351_PI428871386	ENST00000413452.1	2.63	6.74	6.29E-11	1.75E-09	antisense
A_32_P204205	SIX4	2.83	6.47	6.34E-11	1.76E-09	protein_coding
A_23_P259357	SLC35E4	-1.84	15.90	6.35E-11	1.77E-09	protein_coding
A_33_P3214550	CXCR2	-3.12	7.00	6.37E-11	1.77E-09	protein_coding
CUST_18870_PI428871386	ENST00000520357.1	2.02	6.07	6.39E-11	1.78E-09	antisense
A_23_P341275	POP1	2.00	8.75	6.42E-11	1.78E-09	protein_coding
CUST_9467_PI428871386	ENST00000425330.1	2.86	5.69	6.48E-11	1.80E-09	antisense
CUST_38633_PI428871386	ENST00000592758.1	-1.90	9.15	6.48E-11	1.80E-09	antisense
A_33_P3335910	SYNE1	-1.89	6.47	6.51E-11	1.80E-09	protein_coding
A_33_P3317628	PKP3	2.58	11.91	6.54E-11	1.81E-09	protein_coding
CUST_8355_PI428871386	ENST00000493124.1	-1.17	5.00	6.61E-11	1.83E-09	antisense
CUST_10817_PI428871386	ENST00000510016.1	2.92	5.79	6.62E-11	1.83E-09	antisense
A_24_P141688	PCBP2	-1.41	9.25	6.71E-11	1.85E-09	protein_coding
A_23_P140384	CTSG	-3.53	8.37	6.84E-11	1.89E-09	protein_coding
A_33_P3424217	HLA-DQB1	-3.49	11.12	6.88E-11	1.90E-09	protein_coding
A_33_P3423949	CBX2	3.26	8.51	6.94E-11	1.91E-09	protein_coding
CUST_19984_PI428871386	ENST00000523427.1	2.37	6.56	6.97E-11	1.92E-09	lincRNA
A_23_P45294	FAM199X	1.82	9.67	6.98E-11	1.92E-09	protein_coding
A_33_P3249354	CCDC99	1.63	6.45	7.01E-11	1.93E-09	protein_coding
A_23_P58002	TCTA	-1.39	8.50	7.04E-11	1.94E-09	protein_coding
A_23_P114670	ARHGEF16	2.17	8.78	7.06E-11	1.94E-09	protein_coding
A_23_P214080	EGR1	-4.04	13.10	7.06E-11	1.94E-09	protein_coding
A_33_P3298387	PLK1	1.51	5.52	7.10E-11	1.95E-09	protein_coding
CUST_10843_PI428871386	ENST00000506814.1	1.67	5.27	7.28E-11	2.00E-09	antisense
A_23_P386420	GTF2H3	1.56	7.22	7.37E-11	2.02E-09	protein_coding
CUST_31661_PI428871386	ENST00000559672.1	2.61	5.64	7.39E-11	2.02E-09	lincRNA
CUST_2648_PI428871386	ENST00000598917.1	1.52	9.45	7.65E-11	2.10E-09	antisense
A_33_P3363898	TUBG1	1.62	11.45	7.66E-11	2.10E-09	protein_coding
A_23_P140069	FBXL3	-1.60	9.06	7.66E-11	2.10E-09	protein_coding
CUST_1461_PI428871386	ENST00000587165.1	-1.83	6.35	7.68E-11	2.10E-09	lincRNA
CUST_37858_PI428871386	ENST00000581856.1	2.10	5.24	7.87E-11	2.15E-09	antisense
A_33_P3224212	R3HDM1	1.42	5.68	7.91E-11	2.16E-09	protein_coding
CUST_24211_PI428871386	ENST00000525302.1	2.38	5.43	7.95E-11	2.17E-09	antisense
A_23_P77529	MSLN	-3.56	7.77	8.02E-11	2.19E-09	protein_coding
A_23_P201386	DDAH1	-2.34	10.16	8.05E-11	2.20E-09	protein_coding
CUST_37891_PI428871386	ENST00000573479.1	1.56	7.98	8.14E-11	2.22E-09	lincRNA
CUST_11383_PI428871386	ENST00000503066.1	-2.02	6.98	8.15E-11	2.22E-09	antisense
CUST_17415_PI428871386	ENST00000453666.1	-2.11	6.35	8.17E-11	2.23E-09	antisense
A_32_P74409	C11orf96	-4.00	13.71	8.19E-11	2.23E-09	protein_coding
CUST_27343_PI428871386	ENST00000552167.1	-1.34	5.29	8.27E-11	2.25E-09	lincRNA
CUST_20001_PI428871386	ENST00000517838.1	2.71	7.47	8.33E-11	2.27E-09	lincRNA
A_23_P162970	IPO4	1.81	7.92	8.40E-11	2.28E-09	protein_coding
CUST_1599_PI428871386	ENST00000456582.1	-1.64	7.10	8.46E-11	2.30E-09	antisense
A_23_P29922	TLR3	-2.41	8.19	8.47E-11	2.30E-09	protein_coding
A_32_P52018	PHACTR1	-1.71	7.06	8.66E-11	2.35E-09	protein_coding
CUST_35058_PI428871386	ENST00000572453.1	-1.60	6.19	8.74E-11	2.37E-09	antisense
CUST_17158_PI428871386	ENST00000414797.1	-2.31	7.73	8.77E-11	2.38E-09	antisense
CUST_9951_PI428871386	ENST00000441644.1	2.11	6.54	8.94E-11	2.42E-09	antisense
CUST_18922_PI428871386	ENST00000523664.1	-1.25	5.16	8.98E-11	2.43E-09	lincRNA
A_33_P3297621	KIAA0090	1.67	7.57	9.03E-11	2.45E-09	protein_coding
A_33_P3393796	TRMT5	1.65	8.80	9.04E-11	2.45E-09	protein_coding
A_33_P3313283	CILP2	2.69	6.26	9.08E-11	2.46E-09	protein_coding
CUST_32643_PI428871386	ENST00000559505.1	-1.58	5.61	9.10E-11	2.46E-09	antisense
A_33_P3286859	ATP8A2	-2.23	7.06	9.12E-11	2.47E-09	protein_coding
A_23_P400378	GPBAR1	-1.49	6.79	9.13E-11	2.47E-09	protein_coding

CUST_40889_PI428871386	ENST00000569087.1	-1.60	10.32	9.18E-11	2.48E-09	lincRNA
CUST_17156_PI428871386	ENST00000446159.1	-2.22	7.47	9.22E-11	2.49E-09	antisense
A_23_P2041	MICALCL	-2.72	7.54	9.32E-11	2.52E-09	protein_coding
CUST_4304_PI428871386	ENST00000567540.1	-1.68	6.75	9.40E-11	2.53E-09	lincRNA
A_24_P98524	PPP3CB	-1.48	9.54	9.46E-11	2.55E-09	protein_coding
A_33_P3766959	TDP1	2.34	9.04	9.50E-11	2.56E-09	protein_coding
A_23_P143885	ARHGEF3	-1.88	8.18	9.60E-11	2.58E-09	protein_coding
A_24_P204675	LOC100132247	-1.67	12.43	9.62E-11	2.59E-09	protein_coding
A_33_P3257714	RPS23	-2.12	10.19	9.68E-11	2.60E-09	protein_coding
A_23_P215931	LEPROTL1	-1.59	10.76	9.74E-11	2.62E-09	protein_coding
CUST_12913_PI428871386	ENST00000507387.1	2.46	5.09	9.77E-11	2.62E-09	lincRNA
A_23_P165007	RASGRP4	-1.46	6.79	9.77E-11	2.62E-09	protein_coding
CUST_23754_PI428871386	ENST00000534540.1	1.98	6.51	9.80E-11	2.63E-09	lincRNA
A_23_P40588	HSCB	1.55	8.63	9.81E-11	2.63E-09	protein_coding
A_23_P319719	PGAM5	1.66	6.04	9.87E-11	2.65E-09	protein_coding
CUST_5589_PI428871386	ENST00000601509.1	-1.52	6.82	1.01E-10	2.69E-09	antisense
CUST_13312_PI428871386	ENST00000508517.1	-1.90	7.46	1.01E-10	2.70E-09	antisense
A_24_P345993	CANX	-1.99	7.56	1.02E-10	2.72E-09	protein_coding
A_23_P394395	JPH2	-1.67	6.23	1.02E-10	2.74E-09	protein_coding
A_23_P256724	TNFRSF10C	-2.21	8.69	1.03E-10	2.76E-09	protein_coding
A_33_P3209351	IARS	1.45	8.38	1.03E-10	2.76E-09	protein_coding
CUST_19066_PI428871386	ENST00000518078.1	-1.21	5.32	1.04E-10	2.77E-09	antisense
A_23_P81912	TUBB	1.91	11.10	1.04E-10	2.77E-09	protein_coding
CUST_18917_PI428871386	ENST00000523786.1	-1.67	5.37	1.04E-10	2.78E-09	lincRNA
CUST_2766_PI428871386	ENST00000441851.1	2.73	5.47	1.04E-10	2.78E-09	lincRNA
CUST_31660_PI428871386	ENST00000559672.1	2.90	5.83	1.04E-10	2.78E-09	lincRNA
A_24_P320328	SUB1	1.50	10.41	1.05E-10	2.79E-09	protein_coding
CUST_40996_PI428871386	ENST00000417578.1	1.74	9.38	1.05E-10	2.79E-09	lincRNA
A_23_P127579	PTS	1.61	9.96	1.05E-10	2.79E-09	protein_coding
CUST_28210_PI428871386	ENST00000448748.1	-1.20	4.95	1.07E-10	2.85E-09	lincRNA
A_33_P3792328	LSM3	-1.36	5.84	1.08E-10	2.87E-09	protein_coding
A_23_P58770	HAND1	-1.44	5.09	1.08E-10	2.87E-09	protein_coding
CUST_11814_PI428871386	ENST00000578387.1	-1.43	6.01	1.08E-10	2.87E-09	lincRNA
A_23_P15542	HSD17B1	2.83	7.46	1.08E-10	2.88E-09	protein_coding
CUST_17716_PI428871386	ENST00000431189.1	-2.05	8.03	1.08E-10	2.88E-09	antisense
CUST_36004_PI428871386	ENST00000377540.1	-2.94	7.71	1.09E-10	2.90E-09	protein_coding
CUST_34909_PI428871386	ENST00000571091.1	-2.26	9.62	1.11E-10	2.95E-09	lincRNA
A_23_P348383	CC2D2A	-1.67	7.03	1.11E-10	2.96E-09	protein_coding
A_24_P242581	SLC5A9	-1.28	5.18	1.12E-10	2.96E-09	protein_coding
A_33_P3398897	QDPR	-1.90	8.90	1.13E-10	3.00E-09	protein_coding
A_23_P95790	ITLN1	-3.89	7.78	1.13E-10	3.00E-09	protein_coding
CUST_20421_PI428871386	ENST00000452643.1	3.08	6.32	1.13E-10	3.00E-09	lincRNA
A_23_P156748	ANKS1A	-1.84	10.45	1.14E-10	3.01E-09	protein_coding
A_23_P8108	HLA-DQB1	-3.38	12.45	1.15E-10	3.04E-09	protein_coding
CUST_35324_PI428871386	ENST00000480811.1	1.92	7.36	1.16E-10	3.06E-09	antisense
CUST_3566_PI428871386	ENST00000436334.1	2.10	5.99	1.16E-10	3.07E-09	lincRNA
CUST_5881_PI428871386	ENST00000441075.1	1.97	9.53	1.17E-10	3.08E-09	lincRNA
A_33_P3211633	WDR3	1.75	6.88	1.17E-10	3.09E-09	protein_coding
CUST_39938_PI428871386	ENST00000590022.1	-1.16	5.80	1.17E-10	3.09E-09	lincRNA
A_23_P77117	TM6SF1	-1.86	6.92	1.18E-10	3.10E-09	protein_coding
CUST_34904_PI428871386	ENST00000570416.1	-2.45	9.40	1.19E-10	3.13E-09	lincRNA
A_33_P3359084	TAS1R3	2.77	7.31	1.20E-10	3.16E-09	protein_coding
CUST_24669_PI428871386	ENST00000539921.1	2.27	11.29	1.20E-10	3.17E-09	lincRNA
CUST_15479_PI428871386	ENST00000531702.1	-1.70	6.83	1.21E-10	3.19E-09	antisense
CUST_8752_PI428871386	ENST00000496994.1	-1.47	5.65	1.21E-10	3.19E-09	lincRNA
A_33_P3357082	METTL8	1.72	7.34	1.22E-10	3.21E-09	protein_coding
A_23_P159937	SLC6A8	1.87	8.15	1.22E-10	3.21E-09	protein_coding
A_24_P307724	FLJ33360	-1.29	5.26	1.23E-10	3.22E-09	lincRNA
A_33_P3216297	NR3C1	-1.66	8.69	1.23E-10	3.23E-09	protein_coding
A_23_P258769	HLA-DPB1	-3.37	13.87	1.24E-10	3.24E-09	protein_coding
CUST_2626_PI428871386	ENST00000415675.2	-1.71	5.46	1.24E-10	3.26E-09	antisense
CUST_7231_PI428871386	ENST00000438978.1	-2.09	7.91	1.24E-10	3.26E-09	antisense
CUST_32648_PI428871386	ENST00000502125.2	-2.13	7.14	1.24E-10	3.26E-09	antisense

A_23_P34527	FLAD1	1.72	8.59	1.26E-10	3.31E-09	protein_coding
A_33_P3366102	KLHL7	2.08	7.62	1.26E-10	3.31E-09	protein_coding
A_33_P3388016	CCDC72	-1.68	11.01	1.27E-10	3.32E-09	protein_coding
CUST_10067_PI428871386	ENST00000503709.1	-1.09	7.51	1.28E-10	3.34E-09	antisense
A_23_P429977	KCNQ1	-3.28	10.42	1.28E-10	3.34E-09	protein_coding
CUST_3163_PI428871386	ENST00000419190.1	2.54	6.42	1.29E-10	3.38E-09	antisense
CUST_13313_PI428871386	ENST00000508517.1	-1.80	7.15	1.30E-10	3.38E-09	antisense
CUST_20422_PI428871386	ENST00000452643.1	3.10	6.20	1.30E-10	3.38E-09	lincRNA
A_23_P13797	C12orf49	-1.90	8.04	1.30E-10	3.39E-09	protein_coding
A_33_P3236813	GPR19	2.49	5.69	1.32E-10	3.45E-09	protein_coding
A_32_P181077	DOCK8	-2.69	9.69	1.34E-10	3.50E-09	protein_coding
A_23_P348159	ENST00000439203	-2.34	7.15	1.35E-10	3.52E-09	protein_coding
A_24_P115621	EIF4EBP2	-1.52	9.80	1.36E-10	3.56E-09	protein_coding
A_23_P319583	RIMS3	-2.10	7.37	1.37E-10	3.57E-09	protein_coding
A_23_P59616	GTF2IRD2	-1.66	7.51	1.37E-10	3.57E-09	protein_coding
A_33_P3303810	LAD1	2.73	11.03	1.39E-10	3.60E-09	protein_coding
A_33_P3253707	LRR1	1.69	8.27	1.39E-10	3.61E-09	protein_coding
CUST_32982_PI428871386	ENST00000562166.1	-1.39	5.08	1.39E-10	3.62E-09	antisense
A_23_P76034	PVRL1	1.94	5.98	1.40E-10	3.64E-09	protein_coding
CUST_34919_PI428871386	ENST00000574016.1	-1.82	6.49	1.41E-10	3.65E-09	lincRNA
A_32_P132589	ZYG11A	2.55	5.28	1.42E-10	3.67E-09	protein_coding
CUST_9129_PI428871386	ENST00000489690.1	-1.14	4.87	1.42E-10	3.68E-09	antisense
A_23_P411814	OSTC	1.56	10.79	1.42E-10	3.68E-09	protein_coding
CUST_2555_PI428871386	ENST00000453136.1	-2.14	6.65	1.43E-10	3.71E-09	antisense
A_33_P3298861	SLC12A8	1.90	6.17	1.43E-10	3.71E-09	protein_coding
CUST_1595_PI428871386	ENST00000452846.1	-1.72	7.08	1.44E-10	3.72E-09	antisense
CUST_10849_PI428871386	ENST00000500765.1	-1.41	5.99	1.44E-10	3.73E-09	antisense
A_24_P398500	MPHOSPH9	1.37	6.27	1.46E-10	3.77E-09	protein_coding
A_23_P45940	TFB2M	1.78	8.76	1.46E-10	3.77E-09	protein_coding
A_23_P136978	SRPX2	2.46	7.97	1.47E-10	3.80E-09	protein_coding
CUST_17718_PI428871386	ENST00000416999.1	-2.32	8.17	1.48E-10	3.83E-09	antisense
CUST_16942_PI428871386	ENST00000422831.1	-1.11	5.45	1.49E-10	3.83E-09	lincRNA
A_23_P155477	C3orf18	-1.82	8.52	1.49E-10	3.83E-09	protein_coding
A_33_P3343690	FDX1	-1.72	7.52	1.49E-10	3.83E-09	protein_coding
CUST_3165_PI428871386	ENST00000415582.1	2.34	6.48	1.53E-10	3.94E-09	antisense
A_23_P48740	DIO2	2.93	7.39	1.54E-10	3.95E-09	protein_coding
A_33_P3277514	BTK	-2.53	9.28	1.54E-10	3.95E-09	protein_coding
A_23_P137856	MUC1	-3.24	12.43	1.55E-10	3.98E-09	protein_coding
A_23_P85543	RNF2	1.50	6.81	1.56E-10	4.00E-09	protein_coding
CUST_38906_PI428871386	ENST00000587762.1	-3.10	12.27	1.56E-10	4.00E-09	lincRNA
CUST_17719_PI428871386	ENST00000429901.1	-2.45	8.92	1.57E-10	4.02E-09	antisense
A_33_P3410279	DOCK9	-2.09	8.08	1.58E-10	4.06E-09	protein_coding
A_23_P157072	EIF3B	1.58	11.34	1.59E-10	4.07E-09	protein_coding
CUST_34912_PI428871386	ENST00000576489.1	-1.94	7.48	1.60E-10	4.11E-09	lincRNA
CUST_16780_PI428871386	ENST00000430518.1	-1.54	5.44	1.62E-10	4.15E-09	lincRNA
A_33_P3233841	IL6ST	-2.00	10.48	1.64E-10	4.19E-09	protein_coding
CUST_3470_PI428871386	ENST00000438158.1	2.61	5.74	1.65E-10	4.20E-09	lincRNA
CUST_14438_PI428871386	ENST00000561592.1	1.86	5.47	1.66E-10	4.24E-09	antisense
A_23_P14564	GPR65	-2.74	8.25	1.67E-10	4.26E-09	protein_coding
CUST_6256_PI428871386	ENST00000595449.1	-1.38	5.30	1.69E-10	4.32E-09	antisense
CUST_34901_PI428871386	ENST00000574306.1	-2.28	9.41	1.70E-10	4.34E-09	lincRNA
CUST_25282_PI428871386	ENST00000533672.1	-1.99	6.72	1.71E-10	4.36E-09	antisense
A_23_P351913	LRRN4	-1.47	5.63	1.73E-10	4.40E-09	protein_coding
A_33_P3301920	USP50	-1.31	5.45	1.75E-10	4.45E-09	protein_coding
A_23_P166899	DNAJB11	1.89	10.58	1.76E-10	4.47E-09	protein_coding
CUST_41176_PI428871386	ENST00000445003.1	-2.93	9.86	1.76E-10	4.48E-09	lincRNA
A_33_P3274811	SPRR2F	3.55	8.06	1.78E-10	4.52E-09	protein_coding
CUST_26228_PI428871386	ENST00000427111.3	-1.68	6.36	1.79E-10	4.53E-09	lincRNA
CUST_17164_PI428871386	ENST00000448195.1	-2.07	6.80	1.79E-10	4.53E-09	antisense
CUST_33863_PI428871386	ENST00000570167.1	-1.75	5.61	1.80E-10	4.56E-09	antisense
A_24_P289178	C16orf74	2.26	6.91	1.82E-10	4.61E-09	protein_coding
CUST_26227_PI428871386	ENST00000499762.1	-1.73	6.88	1.87E-10	4.72E-09	antisense
A_23_P414312	KNDC1	-1.35	5.37	1.88E-10	4.75E-09	protein_coding

A_23_P69493	RHOA	-1.67	11.81	1.88E-10	4.75E-09	protein_coding
A_24_P25346	CIRH1A	1.69	9.03	1.91E-10	4.81E-09	protein_coding
CUST_16551_PI428871386	ENST00000449537.1	-1.20	5.02	1.91E-10	4.81E-09	lincRNA
A_33_P3221989	CACNB4	-2.33	6.56	1.93E-10	4.88E-09	protein_coding
A_33_P3411848	CNFN	2.44	8.04	1.93E-10	4.88E-09	protein_coding
A_33_P3313258	ATAD3B	1.64	7.84	1.94E-10	4.88E-09	protein_coding
CUST_5349_PI428871386	ENST00000439192.1	2.55	6.55	1.94E-10	4.89E-09	antisense
CUST_9464_PI428871386	ENST00000451742.1	3.34	5.76	1.98E-10	4.97E-09	antisense
A_23_P3849	TRAP1	1.66	8.17	2.03E-10	5.09E-09	protein_coding
CUST_15191_PI428871386	ENST00000455530.1	-2.01	6.00	2.04E-10	5.12E-09	lincRNA
CUST_20000_PI428871386	ENST00000519481.1	2.87	7.02	2.04E-10	5.13E-09	lincRNA
CUST_36396_PI428871386	ENST00000506504.3	1.40	6.26	2.05E-10	5.13E-09	antisense
A_23_P405942	LARP4B	-1.14	10.26	2.06E-10	5.17E-09	protein_coding
CUST_24675_PI428871386	ENST00000537068.1	2.23	11.32	2.07E-10	5.20E-09	lincRNA
CUST_25957_PI428871386	ENST00000540136.1	-1.67	5.57	2.08E-10	5.21E-09	antisense
CUST_8115_PI428871386	ENST00000431705.1	-1.40	5.31	2.08E-10	5.21E-09	antisense
A_24_P134235	KHSRP	1.56	6.42	2.08E-10	5.21E-09	protein_coding
CUST_7815_PI428871386	ENST00000420195.1	-1.56	6.98	2.09E-10	5.23E-09	antisense
CUST_12646_PI428871386	ENST00000508512.1	-2.07	5.73	2.15E-10	5.39E-09	lincRNA
A_23_P156402	NME5	-2.50	6.97	2.17E-10	5.42E-09	protein_coding
A_33_P3294252	ATF1	-1.42	9.74	2.18E-10	5.44E-09	protein_coding
A_33_P3221064	LTBP4	-1.70	11.46	2.18E-10	5.44E-09	protein_coding
A_23_P3681	NETO2	2.37	8.57	2.21E-10	5.51E-09	protein_coding
CUST_1597_PI428871386	ENST00000438509.1	-1.73	7.29	2.24E-10	5.58E-09	antisense
A_23_P425681	CCK	-2.83	6.43	2.26E-10	5.63E-09	protein_coding
A_24_P217365	ANKRD28	-1.49	8.65	2.26E-10	5.63E-09	protein_coding
CUST_24671_PI428871386	ENST00000540725.1	2.19	11.57	2.26E-10	5.63E-09	lincRNA
A_33_P3251771	CYLD	-1.73	7.81	2.26E-10	5.63E-09	protein_coding
CUST_42955_PI428871386	ENST00000420096.1	1.96	6.25	2.27E-10	5.64E-09	lincRNA
CUST_5603_PI428871386	ENST00000596740.1	-1.47	6.87	2.27E-10	5.64E-09	antisense
A_32_P142440	PCSK9	-3.12	8.45	2.27E-10	5.64E-09	protein_coding
A_23_P360964	DACT3	-2.65	9.32	2.28E-10	5.66E-09	protein_coding
A_23_P369479	MSI2	2.19	6.39	2.28E-10	5.67E-09	protein_coding
CUST_10385_PI428871386	ENST00000504219.1	-1.46	5.21	2.31E-10	5.73E-09	antisense
CUST_12641_PI428871386	ENST00000504068.1	-1.55	5.71	2.32E-10	5.75E-09	lincRNA
A_33_P3376551	PTGFR	-2.95	6.95	2.33E-10	5.77E-09	protein_coding
A_33_P3294372	LOC100128881	2.41	5.97	2.36E-10	5.84E-09	antisense
A_33_P3767927	NR3C1	-1.92	12.09	2.40E-10	5.93E-09	protein_coding
CUST_17161_PI428871386	ENST00000422093.1	-1.70	6.84	2.41E-10	5.95E-09	antisense
A_33_P3407835	PPM1M	-1.84	7.34	2.41E-10	5.95E-09	protein_coding
A_23_P28953	DNMT3B	2.43	7.14	2.43E-10	5.98E-09	protein_coding
CUST_29950_PI428871386	ENST00000554360.1	-1.51	8.62	2.43E-10	6.00E-09	antisense
A_33_P3277965	NFIC	-1.18	5.88	2.44E-10	6.00E-09	protein_coding
A_23_P134395	TBL2	1.34	10.72	2.50E-10	6.15E-09	protein_coding
A_33_P3228266	CST3	-1.96	12.69	2.55E-10	6.28E-09	protein_coding
CUST_6254_PI428871386	ENST00000421083.1	-1.53	6.03	2.55E-10	6.28E-09	antisense
CUST_3162_PI428871386	ENST00000419190.1	2.48	6.47	2.56E-10	6.30E-09	antisense
CUST_24692_PI428871386	ENST00000538266.1	2.22	8.40	2.57E-10	6.31E-09	lincRNA
A_23_P142075	ACP5	-3.13	14.00	2.57E-10	6.32E-09	protein_coding
CUST_34826_PI428871386	ENST00000562866.1	2.31	5.95	2.59E-10	6.37E-09	antisense
CUST_19991_PI428871386	ENST00000523068.1	2.56	6.28	2.60E-10	6.37E-09	lincRNA
A_23_P22682	ARMCX1	-2.39	9.54	2.60E-10	6.39E-09	protein_coding
A_23_P123256	PDAP1	1.22	9.68	2.61E-10	6.40E-09	protein_coding
CUST_7232_PI428871386	ENST00000438978.1	-2.06	7.89	2.61E-10	6.40E-09	antisense
A_23_P132956	UCHL1	4.12	10.45	2.62E-10	6.41E-09	protein_coding
CUST_37860_PI428871386	ENST00000578119.1	2.05	5.33	2.62E-10	6.41E-09	antisense
A_33_P3421490	KIAA1024	1.74	6.02	2.66E-10	6.51E-09	protein_coding
CUST_7744_PI428871386	ENST00000475197.1	-1.33	5.93	2.67E-10	6.53E-09	antisense
CUST_37520_PI428871386	ENST00000581571.1	3.77	6.25	2.70E-10	6.60E-09	lincRNA
CUST_5555_PI428871386	ENST00000451384.2	-1.48	6.66	2.72E-10	6.65E-09	antisense
A_23_P128532	C12orf45	1.51	9.33	2.74E-10	6.70E-09	protein_coding
CUST_33301_PI428871386	ENST00000565178.1	-1.36	5.71	2.75E-10	6.71E-09	antisense
A_32_P43349	LOC400456	-1.94	5.81	2.75E-10	6.71E-09	lincRNA

A_23_P64404	FADS3	-1.85	7.74	2.75E-10	6.71E-09	protein_coding
A_23_P378288	IKZF4	-1.67	7.81	2.78E-10	6.79E-09	protein_coding
A_33_P3332006	CBX1	1.67	9.40	2.81E-10	6.85E-09	protein_coding
A_33_P3242973	IGF2BP2	2.71	6.22	2.81E-10	6.85E-09	protein_coding
A_23_P153562	C5AR1	-2.93	9.33	2.81E-10	6.85E-09	protein_coding
A_33_P3325704	SPRR2E	3.84	7.82	2.81E-10	6.85E-09	protein_coding
A_24_P129632	DLG5	1.77	8.57	2.82E-10	6.86E-09	protein_coding
CUST_40995_PI428871386	ENST00000417578.1	1.82	9.48	2.84E-10	6.90E-09	lincRNA
CUST_34898_PI428871386	ENST00000334146.3	-2.38	9.39	2.84E-10	6.90E-09	lincRNA
CUST_33502_PI428871386	ENST00000505035.1	-1.24	5.02	2.84E-10	6.91E-09	lincRNA
CUST_24689_PI428871386	ENST00000541615.1	2.12	9.14	2.91E-10	7.06E-09	lincRNA
A_33_P3374293	PYROXD1	-1.50	8.73	2.91E-10	7.07E-09	protein_coding
A_33_P3386671	RORC	-3.43	9.42	2.92E-10	7.09E-09	protein_coding
CUST_32817_PI428871386	ENST00000595428.1	-1.21	6.46	2.92E-10	7.10E-09	lincRNA
A_33_P3214481	P4HA1	1.81	9.96	2.93E-10	7.12E-09	protein_coding
A_32_P158786	C12orf56	2.91	5.63	2.94E-10	7.12E-09	protein_coding
CUST_32627_PI428871386	ENST00000556899.1	-1.97	5.85	2.95E-10	7.16E-09	lincRNA
CUST_3864_PI428871386	ENST00000437798.1	-1.11	4.95	2.97E-10	7.19E-09	antisense
A_32_P141418	ARMC4	-2.92	6.37	2.98E-10	7.22E-09	protein_coding
A_33_P3256303	SELPLG	-1.84	6.31	2.98E-10	7.22E-09	protein_coding
CUST_32989_PI428871386	ENST00000562807.1	-1.24	8.49	3.00E-10	7.25E-09	lincRNA
A_23_P44648	ADAMTS12	1.66	5.36	3.00E-10	7.25E-09	protein_coding
CUST_25148_PI428871386	ENST00000527778.1	2.15	5.98	3.07E-10	7.42E-09	antisense
A_23_P503233	EDARADD	1.98	11.99	3.08E-10	7.44E-09	protein_coding
A_33_P3420747	YTHDC2	-1.29	8.76	3.09E-10	7.45E-09	protein_coding
A_23_P256735	PGCP	-2.33	9.13	3.09E-10	7.45E-09	protein_coding
A_23_P35414	PPP1R3C	-3.10	8.92	3.16E-10	7.61E-09	protein_coding
A_24_P200854	HOXA2	-1.48	6.43	3.19E-10	7.67E-09	protein_coding
A_33_P3274105	C2orf63	-1.12	9.40	3.24E-10	7.78E-09	protein_coding
CUST_27024_PI428871386	ENST00000552378.1	-1.06	4.80	3.27E-10	7.85E-09	lincRNA
A_23_P28334	IL18RAP	-2.28	7.65	3.32E-10	7.97E-09	protein_coding
CUST_7806_PI428871386	ENST00000424349.1	-1.54	10.15	3.32E-10	7.97E-09	antisense
CUST_8506_PI428871386	ENST00000476021.1	1.17	4.91	3.33E-10	7.99E-09	antisense
A_23_P39616	ORC2	1.25	7.28	3.37E-10	8.07E-09	protein_coding
CUST_1594_PI428871386	ENST00000452846.1	-1.67	7.28	3.38E-10	8.11E-09	antisense
A_23_P70670	CD83	-2.91	10.96	3.41E-10	8.16E-09	protein_coding
A_32_P225345	C11orf88	-3.71	7.11	3.45E-10	8.25E-09	protein_coding
CUST_23755_PI428871386	ENST00000534540.1	1.86	6.20	3.46E-10	8.28E-09	lincRNA
A_24_P97405	CCRL2	-1.70	5.87	3.47E-10	8.30E-09	protein_coding
CUST_19999_PI428871386	ENST00000519481.1	2.62	7.43	3.48E-10	8.31E-09	lincRNA
CUST_5350_PI428871386	ENST00000413452.1	2.57	6.47	3.50E-10	8.34E-09	antisense
A_23_P371835	GATA5	-1.59	6.06	3.51E-10	8.38E-09	protein_coding
A_33_P3418942	NUDT21	1.70	7.99	3.53E-10	8.41E-09	protein_coding
A_33_P3362321	FHL2	2.31	8.15	3.53E-10	8.42E-09	protein_coding
A_33_P3260062	PIAS4	1.07	5.12	3.54E-10	8.44E-09	protein_coding
CUST_34903_PI428871386	ENST00000576749.1	-2.30	9.36	3.55E-10	8.47E-09	lincRNA
A_33_P3407299	ANP32E	2.57	7.56	3.58E-10	8.53E-09	protein_coding
A_23_P139585	PDE1B	-1.58	6.38	3.59E-10	8.53E-09	protein_coding
A_23_P401774	ELMOD1	-1.53	5.35	3.59E-10	8.54E-09	protein_coding
A_33_P3249716	ENST00000415640	1.70	6.94	3.66E-10	8.71E-09	antisense
CUST_24212_PI428871386	ENST00000530430.1	2.61	5.44	3.70E-10	8.80E-09	antisense
A_23_P44974	MRPL13	1.76	11.48	3.73E-10	8.85E-09	protein_coding
A_33_P3410093	LTA4H	-2.13	9.51	3.74E-10	8.87E-09	protein_coding
A_23_P305092	CRTAM	-2.46	7.01	3.74E-10	8.88E-09	protein_coding
CUST_7148_PI428871386	ENST00000433296.1	-1.39	5.47	3.75E-10	8.89E-09	antisense
A_23_P157793	CA9	2.76	5.86	3.76E-10	8.91E-09	protein_coding
CUST_37815_PI428871386	ENST00000568797.1	-1.38	4.82	3.76E-10	8.92E-09	antisense
CUST_23749_PI428871386	ENST00000500447.1	1.80	6.40	3.79E-10	8.97E-09	lincRNA
A_24_P83615	NLRP1	-1.48	6.11	3.79E-10	8.97E-09	protein_coding
A_23_P141315	NLE1	1.88	8.55	3.81E-10	9.01E-09	protein_coding
A_33_P3258392	EDN1	-2.78	8.30	3.82E-10	9.03E-09	protein_coding
CUST_18914_PI428871386	ENST00000519144.1	-1.45	5.75	3.83E-10	9.06E-09	lincRNA
A_33_P3280066	PTRF	-1.78	15.92	3.84E-10	9.07E-09	protein_coding

A_32_P53486	BOLA2B	1.75	12.39	3.90E-10	9.21E-09	protein_coding
A_23_P62188	ZC4H2	-2.08	8.07	3.92E-10	9.26E-09	protein_coding
A_23_P258912	MYOM2	-1.55	6.38	3.98E-10	9.39E-09	protein_coding
A_33_P3367301	GJD3	-2.29	8.38	4.04E-10	9.52E-09	protein_coding
CUST_12143_PI428871386	ENST00000561606.1	1.85	6.19	4.07E-10	9.59E-09	antisense
CUST_40240_PI428871386	ENST00000413496.1	-1.80	6.35	4.08E-10	9.60E-09	antisense
CUST_5602_PI428871386	ENST00000596740.1	-1.49	6.82	4.09E-10	9.64E-09	antisense
CUST_35980_PI428871386	ENST00000581080.1	-1.44	6.30	4.10E-10	9.64E-09	antisense
A_33_P3745146	CADM1	-1.68	7.68	4.11E-10	9.67E-09	protein_coding
CUST_17717_PI428871386	ENST00000416999.1	-2.33	8.19	4.11E-10	9.68E-09	antisense
CUST_18729_PI428871386	ENST00000523507.1	-1.43	12.53	4.22E-10	9.91E-09	antisense
CUST_806_PI428871386	ENST00000311990.4	-1.54	7.16	4.23E-10	9.93E-09	lincRNA
CUST_29216_PI428871386	ENST00000423246.1	-1.41	5.19	4.23E-10	9.94E-09	antisense
CUST_37753_PI428871386	ENST00000578741.1	-1.45	5.19	4.29E-10	1.01E-08	lincRNA
CUST_805_PI428871386	ENST00000311990.4	-1.48	6.98	4.31E-10	1.01E-08	lincRNA
CUST_14124_PI428871386	ENST00000515513.1	-1.23	5.93	4.32E-10	1.01E-08	lincRNA
A_32_P162250	ARHGAP18	-2.25	8.55	4.35E-10	1.02E-08	protein_coding
A_23_P252556	BAI3	-2.17	5.84	4.36E-10	1.02E-08	protein_coding
A_23_P39116	LIG1	1.61	9.36	4.43E-10	1.04E-08	protein_coding
A_33_P3320272	TMEM79	2.27	6.78	4.46E-10	1.04E-08	protein_coding
CUST_34827_PI428871386	ENST00000562866.1	2.31	6.07	4.49E-10	1.05E-08	antisense
A_32_P14850	NPIPL2	-1.65	12.45	4.51E-10	1.06E-08	protein_coding
CUST_1460_PI428871386	ENST00000587165.1	-1.86	6.44	4.52E-10	1.06E-08	lincRNA
A_23_P70688	LY86	-2.68	9.58	4.52E-10	1.06E-08	protein_coding
A_32_P5251	RARA	-1.58	9.30	4.57E-10	1.07E-08	protein_coding
CUST_3471_PI428871386	ENST00000438158.1	2.57	5.76	4.58E-10	1.07E-08	lincRNA
A_23_P34176	WWC3	-1.95	9.59	4.60E-10	1.07E-08	protein_coding
CUST_8362_PI428871386	ENST00000466225.2	-1.35	5.62	4.60E-10	1.07E-08	antisense
A_33_P3212109	DCDC2	-2.52	7.25	4.66E-10	1.09E-08	protein_coding
CUST_40988_PI428871386	ENST00000423536.1	1.76	9.43	4.68E-10	1.09E-08	lincRNA
CUST_17703_PI428871386	ENST00000418546.1	-1.42	5.33	4.71E-10	1.10E-08	lincRNA
CUST_13567_PI428871386	ENST00000503470.1	-1.23	5.00	4.79E-10	1.11E-08	antisense
A_23_P326760	MYRIP	-3.22	6.85	4.79E-10	1.12E-08	protein_coding
A_23_P393607	SNAP47	1.33	10.66	4.83E-10	1.12E-08	protein_coding
A_24_P153840	FGD3	-1.92	9.09	4.89E-10	1.14E-08	protein_coding
CUST_40345_PI428871386	ENST00000593109.1	-1.41	5.18	4.93E-10	1.15E-08	lincRNA
CUST_19994_PI428871386	ENST00000521122.1	2.56	6.13	4.94E-10	1.15E-08	lincRNA
CUST_11757_PI428871386	ENST00000502661.1	-1.19	5.11	4.98E-10	1.16E-08	lincRNA
A_23_P63980	LRFN4	1.54	9.08	5.00E-10	1.16E-08	protein_coding
A_23_P92928	C6	-2.36	6.21	5.02E-10	1.17E-08	protein_coding
A_23_P170978	ZNF692	1.52	9.24	5.06E-10	1.17E-08	protein_coding
CUST_23254_PI428871386	ENST00000452391.1	1.89	5.78	5.07E-10	1.18E-08	antisense
A_33_P3241681	CDKL2	-1.92	6.39	5.11E-10	1.19E-08	protein_coding
A_33_P3317005	SLC11A1	-1.93	6.49	5.18E-10	1.20E-08	protein_coding
A_33_P3267248	TDRD10	-1.80	6.36	5.21E-10	1.21E-08	protein_coding
A_23_P64898	KLRG1	-2.11	7.87	5.25E-10	1.22E-08	protein_coding
A_33_P3245415	N4BP2L1	-1.67	7.38	5.27E-10	1.22E-08	protein_coding
A_33_P3226357	FOXE1	4.10	5.72	5.38E-10	1.24E-08	protein_coding
CUST_17230_PI428871386	ENST00000594469.1	-2.01	6.07	5.38E-10	1.24E-08	antisense
CUST_8081_PI428871386	ENST00000568686.1	-1.71	5.65	5.40E-10	1.25E-08	lincRNA
A_33_P3211734	SIKE1	1.59	9.27	5.43E-10	1.25E-08	protein_coding
CUST_35332_PI428871386	ENST00000484836.1	1.68	7.03	5.46E-10	1.26E-08	antisense
CUST_34261_PI428871386	ENST00000574178.1	-2.33	5.79	5.47E-10	1.26E-08	lincRNA
A_23_P74950	RCC2	1.70	11.12	5.47E-10	1.26E-08	protein_coding
CUST_19997_PI428871386	ENST00000520913.1	2.54	6.37	5.49E-10	1.27E-08	lincRNA
A_23_P15678	TEKT1	-2.12	5.66	5.52E-10	1.27E-08	protein_coding
A_33_P3329769	BEND6	1.58	5.62	5.53E-10	1.28E-08	protein_coding
CUST_18923_PI428871386	ENST00000524338.1	-1.31	5.21	5.57E-10	1.28E-08	lincRNA
A_33_P3284129	LYPD1	2.86	6.32	5.61E-10	1.29E-08	protein_coding
CUST_20002_PI428871386	ENST00000517838.1	2.70	7.03	5.62E-10	1.29E-08	lincRNA
A_23_P332326	ARHGEF19	1.76	9.22	5.65E-10	1.30E-08	protein_coding
CUST_17157_PI428871386	ENST00000446159.1	-1.90	6.55	5.65E-10	1.30E-08	antisense
CUST_40990_PI428871386	ENST00000436764.1	1.76	9.69	5.66E-10	1.30E-08	lincRNA

A_23_P70355	SERPINB6	-1.58	9.57	5.70E-10	1.31E-08	protein_coding
A_33_P3421118	IL20RA	-2.27	6.66	5.71E-10	1.31E-08	protein_coding
CUST_16779_PI428871386	ENST00000458087.2	-1.50	5.61	5.73E-10	1.32E-08	lincRNA
A_23_P33022	POLR2L	-1.33	14.19	5.83E-10	1.34E-08	protein_coding
A_24_P254551	ARHGEF9	-1.59	7.18	5.87E-10	1.35E-08	protein_coding
A_24_P4705	PPME1	2.31	7.69	5.90E-10	1.35E-08	protein_coding
CUST_34900_PI428871386	ENST00000574306.1	-2.30	9.16	5.92E-10	1.36E-08	lincRNA
A_23_P142389	LSR	1.71	8.65	5.98E-10	1.37E-08	protein_coding
A_33_P3230264	GPC3	-3.02	10.05	5.98E-10	1.37E-08	protein_coding
CUST_4210_PI428871386	ENST00000418970.1	2.06	5.87	5.99E-10	1.37E-08	antisense
CUST_30432_PI428871386	ENST00000556942.1	-3.91	7.00	6.02E-10	1.38E-08	antisense
A_24_P751074	ETS1	-1.47	6.98	6.03E-10	1.38E-08	protein_coding
CUST_24213_PI428871386	ENST00000530430.1	2.31	5.36	6.06E-10	1.38E-08	antisense
A_24_P106624	MEOX2	-1.95	6.48	6.15E-10	1.40E-08	protein_coding
A_33_P3422679	FAM149B1	-1.39	8.24	6.21E-10	1.42E-08	protein_coding
A_33_P3212615	TFPI	-3.66	9.91	6.24E-10	1.42E-08	protein_coding
A_24_P166094	ARFIP1	-1.55	10.26	6.32E-10	1.44E-08	protein_coding
CUST_7205_PI428871386	ENST00000419922.1	-1.65	5.75	6.36E-10	1.45E-08	lincRNA
A_33_P3398251	FOXP3	1.22	5.42	6.36E-10	1.45E-08	protein_coding
A_24_P416997	APOL3	-2.00	7.64	6.37E-10	1.45E-08	protein_coding
CUST_933_PI428871386	ENST00000445226.1	1.39	5.81	6.56E-10	1.49E-08	antisense
CUST_3536_PI428871386	ENST00000412855.1	2.02	5.92	6.57E-10	1.49E-08	lincRNA
A_23_P91221	PKIG	-1.83	8.13	6.59E-10	1.50E-08	protein_coding
A_23_P62731	MRPS14	1.45	9.81	6.62E-10	1.50E-08	protein_coding
CUST_19990_PI428871386	ENST00000518528.1	2.45	6.51	6.63E-10	1.51E-08	lincRNA
A_23_P501822	JUP	1.79	10.78	6.72E-10	1.52E-08	protein_coding
CUST_17292_PI428871386	ENST00000435257.1	2.53	5.43	6.79E-10	1.54E-08	antisense
A_33_P3236676	C9orf152	-3.05	8.20	6.81E-10	1.54E-08	protein_coding
A_23_P92107	SLC15A2	-1.24	5.55	6.91E-10	1.57E-08	protein_coding
CUST_34902_PI428871386	ENST00000576749.1	-2.32	9.22	6.95E-10	1.57E-08	lincRNA
A_23_P44505	KLF11	-1.58	9.07	6.96E-10	1.58E-08	protein_coding
A_33_P3422888	CLEC16A	1.20	5.67	6.99E-10	1.58E-08	protein_coding
A_24_P162287	CEP250	1.76	6.26	7.01E-10	1.59E-08	protein_coding
CUST_37746_PI428871386	ENST00000584201.1	-1.61	6.01	7.04E-10	1.59E-08	lincRNA
A_23_P25150	HOXC9	3.98	6.33	7.05E-10	1.59E-08	protein_coding
A_23_P200203	ECHDC2	-2.14	12.41	7.06E-10	1.59E-08	protein_coding
CUST_6581_PI428871386	ENST00000436922.1	1.37	5.44	7.06E-10	1.60E-08	antisense
A_33_P3282434	SLC16A1	2.61	6.61	7.14E-10	1.61E-08	protein_coding
CUST_35052_PI428871386	ENST00000572547.1	-1.36	6.04	7.18E-10	1.62E-08	antisense
A_33_P3221859	SHOX2	1.92	5.80	7.20E-10	1.62E-08	protein_coding
A_32_P128701	USP53	-2.11	8.98	7.40E-10	1.67E-08	protein_coding
A_33_P3221203	MMP13	4.09	5.95	7.43E-10	1.67E-08	protein_coding
CUST_43118_PI428871386	ENST00000381106.3	1.56	6.34	7.53E-10	1.70E-08	lincRNA
A_33_P3249936	C3orf67	3.05	6.11	7.56E-10	1.70E-08	protein_coding
A_23_P141636	EIF4A3	1.52	11.89	7.68E-10	1.73E-08	protein_coding
CUST_19998_PI428871386	ENST00000520913.1	2.45	6.40	7.73E-10	1.74E-08	lincRNA
A_24_P339869	ZNF295	-1.55	8.00	7.82E-10	1.76E-08	protein_coding
A_32_P163125	SGMS1	-2.09	9.47	7.82E-10	1.76E-08	protein_coding
A_32_P162187	C2	-2.02	10.73	7.87E-10	1.77E-08	protein_coding
A_33_P3324909	JUND	-2.03	8.64	7.90E-10	1.77E-08	protein_coding
A_23_P13102	CASP12	-1.28	5.26	7.93E-10	1.78E-08	protein_coding
CUST_5587_PI428871386	ENST00000598737.1	-1.47	6.61	7.95E-10	1.78E-08	antisense
A_23_P252388	SPEF2	-1.65	6.41	8.10E-10	1.82E-08	protein_coding
CUST_35054_PI428871386	ENST00000573222.1	-1.51	7.67	8.23E-10	1.84E-08	lincRNA
CUST_8802_PI428871386	ENST00000468859.1	-1.61	7.05	8.27E-10	1.85E-08	lincRNA
A_23_P409951	OAZ1	-1.54	15.58	8.28E-10	1.85E-08	protein_coding
CUST_19985_PI428871386	ENST00000517790.1	2.31	6.00	8.30E-10	1.86E-08	lincRNA
A_23_P257355	OTC	-1.35	5.36	8.34E-10	1.87E-08	protein_coding
A_23_P335452	ZCCHC24	-1.56	8.17	8.38E-10	1.87E-08	protein_coding
CUST_1734_PI428871386	ENST00000451023.1	-1.73	6.23	8.39E-10	1.88E-08	lincRNA
A_33_P3226985	CDK5R1	1.87	6.87	8.40E-10	1.88E-08	protein_coding
CUST_36748_PI428871386	ENST00000584047.1	2.07	6.44	8.46E-10	1.89E-08	lincRNA
CUST_9128_PI428871386	ENST00000489690.1	-1.13	4.89	8.48E-10	1.89E-08	antisense

CUST_31435_PI428871386	ENST00000500949.2	-1.50	11.04	8.49E-10	1.89E-08	antisense
CUST_43633_PI428871386	ENST00000441492.1	2.24	6.76	8.51E-10	1.90E-08	lincRNA
CUST_7213_PI428871386	ENST00000415479.1	1.95	5.18	8.58E-10	1.91E-08	lincRNA
A_32_P140706	PMS2	1.41	7.38	8.74E-10	1.95E-08	protein_coding
CUST_26079_PI428871386	ENST00000544842.1	3.13	5.75	8.74E-10	1.95E-08	lincRNA
A_33_P3274930	PMP22	-1.61	6.60	8.77E-10	1.95E-08	protein_coding
CUST_40622_PI428871386	ENST00000441428.1	1.13	4.89	8.77E-10	1.95E-08	antisense
A_32_P143880	MEMO1	1.59	6.94	8.79E-10	1.96E-08	protein_coding
A_33_P3278435	CHCHD8	1.67	10.33	8.85E-10	1.97E-08	protein_coding
CUST_14505_PI428871386	ENST00000427276.1	-1.62	5.06	9.02E-10	2.00E-08	lincRNA
A_23_P407840	FNDC1	3.04	7.64	9.08E-10	2.02E-08	protein_coding
A_23_P257583	DENND2A	-1.90	6.78	9.10E-10	2.02E-08	protein_coding
CUST_5871_PI428871386	ENST00000419736.1	1.93	9.50	9.14E-10	2.03E-08	lincRNA
CUST_143_PI428871386	ENST00000473798.1	1.52	7.11	9.24E-10	2.05E-08	lincRNA
A_33_P3288104	DAAM2	-1.50	6.49	9.27E-10	2.05E-08	protein_coding
CUST_25445_PI428871386	ENST00000561746.1	-1.09	6.40	9.31E-10	2.06E-08	lincRNA
A_33_P3389188	TFAM	1.53	11.04	9.35E-10	2.07E-08	protein_coding
A_23_P132277	MCM5	1.52	9.28	9.37E-10	2.07E-08	protein_coding
A_23_P389250	C12orf70	2.03	5.95	9.38E-10	2.07E-08	protein_coding
A_33_P3409934	SEH1L	1.67	6.99	9.40E-10	2.08E-08	protein_coding
A_33_P3296852	ENST00000381105	-1.93	16.57	9.41E-10	2.08E-08	lincRNA
CUST_25283_PI428871386	ENST00000533672.1	-2.01	6.80	9.47E-10	2.09E-08	antisense
CUST_5586_PI428871386	ENST00000598737.1	-1.40	6.41	9.55E-10	2.11E-08	antisense
A_24_P206121	KCNMB1	-1.12	8.23	9.58E-10	2.11E-08	protein_coding
A_23_P12874	GTPBP4	1.66	10.27	9.63E-10	2.12E-08	protein_coding
CUST_31128_PI428871386	ENST00000552334.1	-2.53	7.61	9.67E-10	2.13E-08	lincRNA
A_23_P58993	MOCS1	-1.77	7.01	9.71E-10	2.14E-08	protein_coding
CUST_13333_PI428871386	ENST00000507558.1	-1.45	6.22	9.77E-10	2.15E-08	antisense
A_23_P203023	RDX	-1.82	8.30	9.97E-10	2.20E-08	protein_coding
CUST_11022_PI428871386	ENST00000508286.1	1.49	5.34	9.98E-10	2.20E-08	antisense
CUST_16742_PI428871386	ENST00000440034.1	-1.63	6.78	1.00E-09	2.21E-08	lincRNA
A_23_P2223	MYL6B	1.54	10.44	1.01E-09	2.21E-08	protein_coding
A_23_P170453	CST5	-1.98	10.30	1.01E-09	2.22E-08	protein_coding
CUST_43632_PI428871386	ENST00000457876.1	2.28	7.08	1.01E-09	2.23E-08	lincRNA
CUST_25149_PI428871386	ENST00000527778.1	2.17	6.08	1.02E-09	2.24E-08	antisense
A_33_P3368895	ENST00000427050	2.65	9.10	1.02E-09	2.24E-08	antisense
CUST_4753_PI428871386	ENST00000449569.1	-1.20	6.19	1.02E-09	2.24E-08	antisense
CUST_16265_PI428871386	ENST00000437621.2	-1.48	6.09	1.03E-09	2.25E-08	lincRNA
CUST_24673_PI428871386	ENST00000537925.1	2.13	11.57	1.04E-09	2.27E-08	lincRNA
CUST_36747_PI428871386	ENST00000579629.1	2.10	6.42	1.04E-09	2.28E-08	lincRNA
CUST_908_PI428871386	ENST00000431759.1	2.16	6.12	1.05E-09	2.31E-08	lincRNA
CUST_21997_PI428871386	ENST00000448685.1	-1.82	6.91	1.06E-09	2.32E-08	antisense
A_23_P155596	FMO3	-2.55	8.06	1.07E-09	2.34E-08	protein_coding
CUST_2767_PI428871386	ENST00000441851.1	2.63	5.42	1.07E-09	2.34E-08	lincRNA
A_23_P18887	MCCC2	1.52	6.80	1.08E-09	2.36E-08	protein_coding
A_33_P3260426	SPRR2A	2.37	6.40	1.09E-09	2.37E-08	protein_coding
CUST_15447_PI428871386	ENST00000458693.1	-1.13	5.61	1.09E-09	2.38E-08	lincRNA
A_33_P3276693	PGF	2.40	9.00	1.10E-09	2.41E-08	protein_coding
CUST_4754_PI428871386	ENST00000445520.1	-1.40	8.03	1.11E-09	2.41E-08	antisense
A_32_P83098	SCNN1B	-2.01	6.96	1.12E-09	2.43E-08	protein_coding
A_23_P335848	GIT2	-1.51	9.90	1.12E-09	2.44E-08	protein_coding
CUST_5588_PI428871386	ENST00000601509.1	-1.42	6.48	1.13E-09	2.45E-08	antisense
CUST_11839_PI428871386	ENST00000510284.1	-1.71	5.76	1.13E-09	2.45E-08	lincRNA
A_23_P77328	GCHFR	-2.25	9.91	1.13E-09	2.46E-08	protein_coding
A_23_P161352	PTPLA	-2.38	8.08	1.13E-09	2.46E-08	protein_coding
A_23_P213959	PPARGC1B	-2.14	7.63	1.13E-09	2.47E-08	protein_coding
CUST_25145_PI428871386	ENST00000528075.1	2.06	6.07	1.15E-09	2.50E-08	antisense
A_33_P3299066	NR4A2	-2.91	8.90	1.16E-09	2.52E-08	protein_coding
A_24_P339560	SIGLEC11	-1.43	5.92	1.17E-09	2.53E-08	protein_coding
CUST_8069_PI428871386	ENST00000432377.1	-1.40	6.22	1.17E-09	2.54E-08	antisense
CUST_8938_PI428871386	ENST00000474250.1	1.78	6.96	1.18E-09	2.56E-08	antisense
A_23_P9293	TJP2	-1.88	10.37	1.19E-09	2.57E-08	protein_coding
CUST_11023_PI428871386	ENST00000508286.1	1.72	5.82	1.19E-09	2.57E-08	antisense

A_23_P331813	ZNF687	1.34	7.50	1.20E-09	2.60E-08	protein_coding
A_23_P47073	WDR37	-1.40	8.46	1.21E-09	2.62E-08	protein_coding
A_33_P3316878	CHPF	1.66	11.92	1.22E-09	2.63E-08	protein_coding
A_23_P204847	LCP1	-2.82	12.41	1.22E-09	2.63E-08	protein_coding
A_24_P316305	AQR	-1.44	8.65	1.23E-09	2.66E-08	protein_coding
A_33_P3330498	ALDH7A1	-1.79	6.16	1.24E-09	2.68E-08	protein_coding
CUST_17538_PI428871386	ENST00000456775.1	-1.52	5.12	1.25E-09	2.70E-08	antisense
A_23_P147665	OLFML1	-1.43	6.68	1.27E-09	2.73E-08	protein_coding
CUST_907_PI428871386	ENST00000431759.1	2.19	6.06	1.27E-09	2.74E-08	lincRNA
A_23_P70719	LAMA2	-2.33	8.24	1.27E-09	2.74E-08	protein_coding
CUST_7219_PI428871386	ENST00000562038.1	-1.51	5.70	1.27E-09	2.74E-08	lincRNA
CUST_14823_PI428871386	ENST00000430595.1	-1.27	5.32	1.27E-09	2.75E-08	antisense
CUST_2554_PI428871386	ENST00000453136.1	-2.22	6.76	1.28E-09	2.75E-08	antisense
A_23_P88435	FOXN3	-1.46	8.23	1.28E-09	2.75E-08	protein_coding
CUST_25277_PI428871386	ENST00000526206.1	-1.60	6.44	1.28E-09	2.76E-08	lincRNA
A_23_P73150	TTC25	-2.79	7.58	1.31E-09	2.82E-08	protein_coding
CUST_8346_PI428871386	ENST00000470447.1	-1.20	5.05	1.32E-09	2.83E-08	antisense
CUST_38886_PI428871386	ENST00000589120.1	1.21	9.09	1.33E-09	2.86E-08	antisense
CUST_18450_PI428871386	ENST00000521025.1	-1.41	6.58	1.33E-09	2.86E-08	antisense
A_24_P346368	C7orf42	-1.36	11.23	1.33E-09	2.86E-08	protein_coding
CUST_34905_PI428871386	ENST00000570416.1	-2.20	9.35	1.35E-09	2.89E-08	lincRNA
A_23_P407684	ZNF598	1.44	8.18	1.36E-09	2.91E-08	protein_coding
CUST_18921_PI428871386	ENST00000523664.1	-1.36	5.15	1.36E-09	2.91E-08	lincRNA
CUST_20054_PI428871386	ENST00000524275.1	-1.23	4.96	1.36E-09	2.92E-08	lincRNA
A_32_P212471	RIIAD1	-2.70	7.87	1.37E-09	2.93E-08	protein_coding
A_24_P274615	ARRDC3	-1.83	8.78	1.37E-09	2.93E-08	protein_coding
A_23_P62901	BTG2	-2.62	12.26	1.37E-09	2.94E-08	protein_coding
A_33_P3718734	ENST00000432377	-1.42	6.26	1.40E-09	2.99E-08	antisense
CUST_9950_PI428871386	ENST00000441644.1	1.96	6.39	1.40E-09	2.99E-08	antisense
CUST_25005_PI428871386	ENST00000532530.1	-1.38	6.41	1.40E-09	3.00E-08	lincRNA
A_23_P159125	SLC16A5	-2.69	11.22	1.40E-09	3.00E-08	protein_coding
A_23_P99642	SLC7A7	-2.84	10.05	1.41E-09	3.01E-08	protein_coding
A_23_P166248	RCAN1	-2.73	9.84	1.41E-09	3.01E-08	protein_coding
A_33_P3251552	RSPO4	-2.04	6.55	1.41E-09	3.02E-08	protein_coding
A_24_P201171	STXBP1	-2.15	7.86	1.41E-09	3.02E-08	protein_coding
A_23_P214079	SPINK1	5.28	8.27	1.42E-09	3.04E-08	protein_coding
A_23_P256542	FAM162A	1.86	11.54	1.42E-09	3.04E-08	protein_coding
A_33_P3269723	ZSWIM7	-1.81	8.87	1.43E-09	3.05E-08	protein_coding
A_24_P88921	XPC	-1.28	7.72	1.43E-09	3.05E-08	protein_coding
A_23_P19663	CTGF	-3.14	9.63	1.43E-09	3.05E-08	protein_coding
A_23_P39766	GLS	-2.67	10.72	1.43E-09	3.06E-08	protein_coding
CUST_25146_PI428871386	ENST00000526585.1	2.16	6.21	1.44E-09	3.06E-08	antisense
A_24_P31235	EIF5A	1.55	10.03	1.44E-09	3.06E-08	protein_coding
CUST_4889_PI428871386	ENST00000421759.1	-1.32	5.84	1.47E-09	3.12E-08	antisense
A_24_P136866	SLC8A1	-2.42	8.52	1.47E-09	3.12E-08	protein_coding
A_23_P107775	TMEM190	-3.75	8.04	1.47E-09	3.13E-08	protein_coding
CUST_19988_PI428871386	ENST00000522963.1	2.50	6.14	1.47E-09	3.13E-08	lincRNA
CUST_7910_PI428871386	ENST00000436123.1	1.60	8.75	1.48E-09	3.14E-08	lincRNA
A_23_P315836	BAIAP2	-2.18	7.28	1.48E-09	3.15E-08	protein_coding
A_23_P13753	NFE2	-2.77	8.17	1.49E-09	3.16E-08	protein_coding
CUST_43034_PI428871386	ENST00000438810.1	1.72	5.23	1.50E-09	3.19E-08	lincRNA
A_23_P66158	C16orf88	1.81	8.58	1.50E-09	3.19E-08	protein_coding
CUST_18919_PI428871386	ENST00000521483.1	-1.24	5.14	1.50E-09	3.19E-08	lincRNA
A_33_P3209229	RAB26	2.35	7.15	1.51E-09	3.20E-08	protein_coding
CUST_17416_PI428871386	ENST00000453666.1	-2.21	6.05	1.52E-09	3.22E-08	antisense
A_24_P39101	KCTD10	-1.41	8.33	1.52E-09	3.22E-08	protein_coding
A_23_P75786	SLC15A3	-2.52	10.59	1.53E-09	3.23E-08	protein_coding
CUST_10816_PI428871386	ENST00000510016.1	2.74	5.73	1.53E-09	3.23E-08	antisense
A_23_P137381	ID3	-1.99	10.31	1.53E-09	3.23E-08	protein_coding
CUST_24685_PI428871386	ENST00000545440.1	2.09	9.01	1.53E-09	3.23E-08	lincRNA
A_24_P74160	SNRPD2	1.49	12.00	1.53E-09	3.24E-08	protein_coding
CUST_34993_PI428871386	ENST00000576086.1	-1.37	5.80	1.54E-09	3.25E-08	antisense
CUST_8080_PI428871386	ENST00000568686.1	-1.76	5.77	1.54E-09	3.26E-08	lincRNA

A_33_P3348884	CCDC141	-1.35	5.79	1.55E-09	3.27E-08	protein_coding
A_23_P68665	ADRM1	1.34	8.91	1.55E-09	3.27E-08	protein_coding
A_23_P45811	DIO1	-1.31	5.06	1.55E-09	3.28E-08	protein_coding
CUST_32601_PI428871386	ENST00000556030.1	-1.55	5.65	1.55E-09	3.28E-08	lincRNA
A_23_P83098	ALDH1A1	-3.11	10.70	1.55E-09	3.28E-08	protein_coding
CUST_30303_PI428871386	ENST00000556781.1	-1.16	4.97	1.56E-09	3.29E-08	lincRNA
CUST_11344_PI428871386	ENST00000512692.1	-1.29	5.53	1.58E-09	3.34E-08	antisense
CUST_7609_PI428871386	ENST00000441386.2	-1.50	6.34	1.60E-09	3.36E-08	antisense
A_23_P36305	ATG16L2	-1.89	10.21	1.60E-09	3.38E-08	protein_coding
A_23_P214139	REV3L	-1.60	9.26	1.61E-09	3.39E-08	protein_coding
A_23_P211504	KDELR3	2.01	8.07	1.61E-09	3.40E-08	protein_coding
CUST_34829_PI428871386	ENST00000562298.1	2.20	5.96	1.62E-09	3.41E-08	antisense
A_33_P3250612	MOCS1	-1.28	7.20	1.62E-09	3.41E-08	protein_coding
A_24_P311926	HLA-G	-2.26	16.10	1.63E-09	3.43E-08	protein_coding
CUST_40272_PI428871386	ENST00000596330.1	-1.08	5.15	1.63E-09	3.43E-08	lincRNA
CUST_22144_PI428871386	ENST00000437232.1	-2.06	8.00	1.63E-09	3.43E-08	antisense
CUST_18365_PI428871386	ENST00000521411.1	-1.47	5.21	1.64E-09	3.44E-08	lincRNA
CUST_18869_PI428871386	ENST00000520357.1	1.76	5.89	1.64E-09	3.45E-08	antisense
A_23_P88589	NR2F2	-2.36	10.82	1.65E-09	3.46E-08	protein_coding
CUST_16711_PI428871386	ENST00000582145.1	-1.42	7.53	1.65E-09	3.46E-08	antisense
A_33_P3840512	SLC25A15	1.53	6.35	1.65E-09	3.46E-08	protein_coding
CUST_15521_PI428871386	ENST00000452675.1	2.27	6.07	1.67E-09	3.49E-08	lincRNA
A_24_P354689	SPOCK1	2.77	7.21	1.67E-09	3.49E-08	protein_coding
A_33_P3281940	RPE	1.39	8.34	1.67E-09	3.50E-08	protein_coding
CUST_6129_PI428871386	ENST00000419784.1	-1.17	4.86	1.68E-09	3.52E-08	lincRNA
A_33_P3249349	PRAME	2.86	7.74	1.70E-09	3.55E-08	protein_coding
A_24_P43876	C9orf40	1.77	6.69	1.73E-09	3.63E-08	protein_coding
A_23_P160800	NR0B2	-2.56	6.82	1.74E-09	3.64E-08	protein_coding
A_33_P3229397	CCT4	1.48	12.47	1.77E-09	3.70E-08	protein_coding
A_23_P27147	ANAPC11	1.37	14.32	1.77E-09	3.71E-08	protein_coding
A_23_P102832	CEP250	1.60	7.83	1.80E-09	3.75E-08	protein_coding
CUST_2592_PI428871386	ENST00000441085.1	2.06	5.30	1.80E-09	3.76E-08	lincRNA
CUST_17705_PI428871386	ENST00000423414.1	-1.27	6.18	1.80E-09	3.76E-08	antisense
CUST_18229_PI428871386	ENST00000519726.1	-1.29	6.26	1.82E-09	3.80E-08	antisense
CUST_10947_PI428871386	ENST00000513793.1	2.02	5.43	1.84E-09	3.85E-08	lincRNA
A_23_P156826	C6orf105	-2.73	7.38	1.85E-09	3.85E-08	protein_coding
CUST_22141_PI428871386	ENST00000417542.1	-1.18	5.01	1.87E-09	3.90E-08	lincRNA
CUST_40989_PI428871386	ENST00000436764.1	1.65	9.20	1.87E-09	3.90E-08	lincRNA
CUST_37082_PI428871386	ENST00000587575.1	2.28	6.82	1.88E-09	3.91E-08	lincRNA
CUST_17317_PI428871386	ENST00000430027.2	2.11	5.00	1.88E-09	3.92E-08	antisense
A_23_P116091	DPP3	1.61	9.31	1.89E-09	3.93E-08	protein_coding
A_24_P158718	DTX4	-1.62	6.42	1.89E-09	3.93E-08	protein_coding
CUST_30833_PI428871386	ENST00000556120.1	-1.48	5.77	1.90E-09	3.95E-08	lincRNA
A_33_P3272330	DNMT3A	1.65	9.55	1.91E-09	3.96E-08	protein_coding
A_33_P3306177	TAPT1	-1.68	7.73	1.91E-09	3.96E-08	protein_coding
A_33_P3308105	GGH	2.99	10.36	1.91E-09	3.96E-08	protein_coding
CUST_30220_PI428871386	ENST00000553470.1	-1.06	5.43	1.93E-09	4.00E-08	antisense
A_23_P216052	FAM83A	3.86	6.43	1.93E-09	4.01E-08	protein_coding
A_33_P3392405	C10orf99	2.27	5.94	1.95E-09	4.04E-08	protein_coding
A_24_P313993	CAPS	-3.19	8.99	1.95E-09	4.05E-08	protein_coding
CUST_2399_PI428871386	ENST00000452399.1	2.17	8.69	1.96E-09	4.06E-08	lincRNA
A_24_P491397	LDLRAD1	-3.57	7.29	1.96E-09	4.06E-08	protein_coding
A_23_P385126	DEPDC7	2.33	6.01	1.97E-09	4.07E-08	protein_coding
A_33_P3285299	GPRIN2	-2.59	9.72	1.97E-09	4.07E-08	protein_coding
CUST_34828_PI428871386	ENST00000562298.1	2.36	6.15	1.97E-09	4.08E-08	antisense
A_33_P3358295	ABTB1	-1.53	8.97	1.97E-09	4.09E-08	protein_coding
A_24_P415601	RNH1	-1.45	10.53	1.98E-09	4.10E-08	protein_coding
A_23_P3038	GPX2	3.78	7.42	1.99E-09	4.11E-08	protein_coding
CUST_5582_PI428871386	ENST00000599435.1	-1.35	6.96	2.01E-09	4.15E-08	antisense
A_23_P104151	TMEM69	1.58	7.98	2.03E-09	4.19E-08	protein_coding
CUST_17231_PI428871386	ENST00000594469.1	-2.05	6.03	2.03E-09	4.20E-08	antisense
A_23_P20697	ADAMTSL2	-1.62	7.46	2.06E-09	4.25E-08	protein_coding
CUST_15130_PI428871386	ENST00000586974.1	-1.31	5.44	2.06E-09	4.26E-08	antisense

A_23_P398566	NR4A3	-3.86	10.07	2.08E-09	4.30E-08	protein_coding
A_33_P3211198	C1orf130	-2.78	8.33	2.08E-09	4.30E-08	protein_coding
CUST_23255_PI428871386	ENST00000452391.1	1.68	5.73	2.12E-09	4.38E-08	antisense
CUST_22777_PI428871386	ENST00000457758.1	1.42	6.39	2.14E-09	4.41E-08	antisense
A_23_P344853	WDR43	1.67	8.35	2.15E-09	4.43E-08	protein_coding
CUST_13743_PI428871386	ENST00000520980.1	-1.10	5.96	2.16E-09	4.44E-08	antisense
CUST_33862_PI428871386	ENST00000570167.1	-1.65	5.65	2.18E-09	4.49E-08	antisense
A_24_P112447	ENTPD7	1.51	5.83	2.21E-09	4.55E-08	protein_coding
A_23_P41854	CARD6	-2.30	10.33	2.22E-09	4.57E-08	protein_coding
A_33_P3222018	CBX3	1.45	5.62	2.22E-09	4.57E-08	protein_coding
A_33_P3370094	MME	-2.60	7.95	2.23E-09	4.59E-08	protein_coding
A_23_P129209	IDH2	1.93	9.52	2.25E-09	4.63E-08	protein_coding
A_33_P3367731	SLC24A2	1.00	5.15	2.25E-09	4.63E-08	protein_coding
CUST_13193_PI428871386	ENST00000508339.1	2.09	5.34	2.26E-09	4.63E-08	lincRNA
CUST_11153_PI428871386	ENST00000515769.1	-1.21	4.93	2.26E-09	4.64E-08	lincRNA
CUST_30433_PI428871386	ENST00000556942.1	-3.80	7.28	2.27E-09	4.66E-08	antisense
CUST_37522_PI428871386	ENST00000580197.1	3.62	6.19	2.28E-09	4.68E-08	lincRNA
A_23_P143958	RPL22L1	2.48	12.89	2.29E-09	4.70E-08	protein_coding
A_23_P34045	EDA	-1.73	6.18	2.30E-09	4.70E-08	protein_coding
A_33_P3387300	FXR1	1.79	10.36	2.32E-09	4.75E-08	protein_coding
CUST_1820_PI428871386	ENST00000566942.1	-2.62	7.75	2.32E-09	4.75E-08	lincRNA
CUST_23180_PI428871386	ENST00000451737.1	-1.17	5.24	2.33E-09	4.77E-08	antisense
A_32_P54137	UQCRH	1.76	13.40	2.34E-09	4.78E-08	protein_coding
A_23_P93792	PMS2	1.56	7.26	2.34E-09	4.79E-08	protein_coding
CUST_10129_PI428871386	ENST00000505448.1	-1.92	7.83	2.34E-09	4.79E-08	lincRNA
A_23_P55073	NOL11	1.45	9.07	2.34E-09	4.79E-08	protein_coding
CUST_42161_PI428871386	ENST00000455567.1	1.84	6.74	2.38E-09	4.85E-08	antisense
CUST_43109_PI428871386	ENST00000438107.1	1.49	6.45	2.39E-09	4.87E-08	lincRNA
A_33_P3251108	SLC33A1	1.40	7.63	2.40E-09	4.90E-08	protein_coding
A_33_P3364646	RASGEF1B	-1.79	8.30	2.41E-09	4.92E-08	protein_coding
A_23_P109881	ITIH4	-1.88	8.53	2.42E-09	4.93E-08	protein_coding
CUST_1443_PI428871386	ENST00000469312.2	-1.33	5.99	2.44E-09	4.97E-08	lincRNA
A_33_P3666884	PNPLA7	-2.69	9.79	2.45E-09	4.99E-08	protein_coding
A_33_P3248629	DENND2A	-1.23	5.32	2.46E-09	5.01E-08	protein_coding
CUST_7746_PI428871386	ENST00000538717.1	-1.18	5.87	2.46E-09	5.02E-08	antisense
CUST_34992_PI428871386	ENST00000576086.1	-1.46	6.00	2.47E-09	5.03E-08	antisense
CUST_22417_PI428871386	ENST00000568976.1	2.70	6.70	2.51E-09	5.10E-08	lincRNA
A_33_P3217819	CCNE2	1.98	5.46	2.52E-09	5.12E-08	protein_coding
CUST_6552_PI428871386	ENST00000426475.1	-2.21	7.38	2.52E-09	5.13E-08	lincRNA
CUST_5467_PI428871386	ENST00000423846.1	1.77	9.66	2.55E-09	5.18E-08	lincRNA
A_23_P61280	COPG2	1.66	7.49	2.55E-09	5.18E-08	protein_coding
CUST_34916_PI428871386	ENST00000573075.1	-1.85	6.53	2.57E-09	5.21E-08	lincRNA
A_23_P37545	AAGAB	1.24	9.99	2.57E-09	5.22E-08	protein_coding
CUST_34921_PI428871386	ENST00000577164.1	-1.57	5.75	2.61E-09	5.30E-08	lincRNA
CUST_936_PI428871386	ENST00000412378.1	1.36	5.90	2.63E-09	5.34E-08	antisense
CUST_144_PI428871386	ENST00000473798.1	1.55	7.62	2.64E-09	5.35E-08	lincRNA
A_33_P3258612	PCNA	1.73	9.17	2.64E-09	5.35E-08	protein_coding
CUST_1735_PI428871386	ENST00000451023.1	-1.63	6.55	2.65E-09	5.36E-08	lincRNA
CUST_36746_PI428871386	ENST00000579629.1	2.01	6.30	2.65E-09	5.36E-08	lincRNA
A_33_P3293573	VPS37D	2.02	8.51	2.67E-09	5.39E-08	protein_coding
CUST_38887_PI428871386	ENST00000589120.1	1.17	9.41	2.68E-09	5.42E-08	antisense
A_32_P211418	CXorf41	-2.45	5.91	2.73E-09	5.51E-08	protein_coding
A_23_P93823	RFC2	1.55	8.28	2.73E-09	5.52E-08	protein_coding
A_24_P911676	SOX4	2.22	8.03	2.73E-09	5.52E-08	protein_coding
CUST_7930_PI428871386	ENST00000425195.1	3.09	5.63	2.78E-09	5.61E-08	lincRNA
A_23_P33583	DNAH7	-2.02	6.03	2.79E-09	5.63E-08	protein_coding
A_24_P873764	BCR	-1.63	9.51	2.79E-09	5.64E-08	protein_coding
CUST_24067_PI428871386	ENST00000534477.1	3.47	6.54	2.82E-09	5.68E-08	lincRNA
CUST_20613_PI428871386	ENST00000436360.1	-2.24	9.41	2.82E-09	5.69E-08	antisense
A_23_P69573	GUCY1A3	-2.21	10.42	2.86E-09	5.75E-08	protein_coding
CUST_6255_PI428871386	ENST00000421083.1	-1.49	6.08	2.88E-09	5.80E-08	antisense
CUST_9466_PI428871386	ENST00000425330.1	2.54	5.46	2.89E-09	5.81E-08	antisense
A_33_P3275422	C14orf70	-1.06	8.89	2.93E-09	5.90E-08	lincRNA

A_24_P81947	CORO1C	-1.79	9.59	2.95E-09	5.93E-08	protein_coding
CUST_22195_PI428871386	ENST00000423551.1	-1.33	6.14	2.96E-09	5.94E-08	antisense
CUST_43631_PI428871386	ENST00000457876.1	2.25	6.95	2.98E-09	5.98E-08	lincRNA
CUST_24679_PI428871386	ENST00000537869.1	2.06	11.34	2.99E-09	6.01E-08	lincRNA
A_24_P267592	SAMHD1	-1.69	7.62	3.02E-09	6.06E-08	protein_coding
A_23_P39251	PLIN5	-2.18	7.01	3.04E-09	6.11E-08	protein_coding
CUST_24205_PI428871386	ENST00000531798.1	-1.30	4.79	3.08E-09	6.17E-08	lincRNA
A_23_P76159	EEA1	-1.31	9.17	3.11E-09	6.23E-08	protein_coding
CUST_8718_PI428871386	ENST00000478301.1	-1.82	7.58	3.13E-09	6.27E-08	lincRNA
A_23_P10077	PNPLA2	-1.52	8.09	3.16E-09	6.33E-08	protein_coding
A_24_P211151	EXOSC5	1.70	6.95	3.17E-09	6.34E-08	protein_coding
A_23_P101407	C3	-3.44	12.68	3.18E-09	6.36E-08	protein_coding
CUST_6409_PI428871386	ENST00000428651.1	2.20	5.62	3.20E-09	6.41E-08	lincRNA
A_33_P3319905	TREM1	-2.59	7.61	3.21E-09	6.41E-08	protein_coding
CUST_20936_PI428871386	ENST00000451596.1	-1.73	6.63	3.21E-09	6.41E-08	lincRNA
CUST_5585_PI428871386	ENST00000597654.1	-1.46	6.74	3.23E-09	6.44E-08	antisense
A_23_P39955	ACTG2	-2.88	12.08	3.25E-09	6.48E-08	protein_coding
CUST_13332_PI428871386	ENST00000507558.1	-1.22	6.00	3.26E-09	6.50E-08	antisense
A_23_P62642	CCDC19	-2.45	6.94	3.27E-09	6.52E-08	protein_coding
A_23_P59192	SNRPC	1.48	11.87	3.31E-09	6.60E-08	protein_coding
A_33_P3329078	HBG1	-1.90	7.84	3.32E-09	6.61E-08	protein_coding
A_33_P3424222	HLA-DQB1	-3.07	12.20	3.34E-09	6.66E-08	protein_coding
CUST_14608_PI428871386	ENST00000436804.1	-1.59	5.69	3.36E-09	6.69E-08	lincRNA
A_33_P3419785	BNIP3	2.55	11.40	3.41E-09	6.78E-08	protein_coding
CUST_28141_PI428871386	ENST00000537998.1	-1.27	5.27	3.42E-09	6.81E-08	lincRNA
CUST_17704_PI428871386	ENST00000418546.1	-1.27	5.32	3.44E-09	6.85E-08	lincRNA
CUST_11017_PI428871386	ENST00000512637.1	1.36	5.42	3.46E-09	6.88E-08	antisense
A_33_P3705907	FAM207A	1.58	8.08	3.50E-09	6.95E-08	protein_coding
A_23_P48175	TMEM106C	1.79	10.94	3.50E-09	6.95E-08	protein_coding
A_24_P331704	KRT80	2.90	7.71	3.51E-09	6.98E-08	protein_coding
CUST_24691_PI428871386	ENST00000541578.1	2.00	8.44	3.59E-09	7.12E-08	lincRNA
A_24_P398940	CASC4	-1.64	10.14	3.59E-09	7.13E-08	protein_coding
A_32_P49423	NPM1	1.46	11.35	3.72E-09	7.37E-08	protein_coding
CUST_43738_PI428871386	ENST00000412882.1	-1.48	6.08	3.75E-09	7.43E-08	antisense
A_33_P3339103	POLR1C	1.72	10.26	3.76E-09	7.44E-08	protein_coding
CUST_21982_PI428871386	ENST00000545372.1	2.53	5.52	3.77E-09	7.46E-08	antisense
CUST_8719_PI428871386	ENST00000478301.1	-1.69	7.30	3.80E-09	7.51E-08	lincRNA
CUST_1908_PI428871386	ENST00000448680.1	-1.92	5.87	3.80E-09	7.51E-08	lincRNA
A_33_P3413962	PFKP	2.46	9.48	3.81E-09	7.53E-08	protein_coding
CUST_37861_PI428871386	ENST00000578119.1	2.03	5.41	3.84E-09	7.58E-08	antisense
CUST_5945_PI428871386	ENST00000416105.1	-1.30	5.47	3.85E-09	7.60E-08	lincRNA
CUST_1598_PI428871386	ENST00000456582.1	-1.59	7.00	3.87E-09	7.64E-08	antisense
A_23_P436284	OSTBETA	-1.65	6.04	3.87E-09	7.64E-08	protein_coding
A_23_P258964	IARS	1.44	9.89	3.88E-09	7.65E-08	protein_coding
A_23_P128084	ITGA7	-2.15	9.23	3.88E-09	7.65E-08	protein_coding
A_32_P189790	DKFZp779M0652	-1.60	6.31	3.88E-09	7.65E-08	lincRNA
A_24_P268786	MYNN	1.45	7.96	3.90E-09	7.69E-08	protein_coding
CUST_30431_PI428871386	ENST00000555407.1	-1.69	6.52	3.91E-09	7.71E-08	antisense
A_23_P339098	SLC35F2	2.08	8.54	3.93E-09	7.73E-08	protein_coding
CUST_25956_PI428871386	ENST00000540136.1	-1.59	5.66	4.00E-09	7.88E-08	antisense
A_23_P53856	N4BP2L2	-1.61	11.91	4.01E-09	7.89E-08	protein_coding
CUST_5460_PI428871386	ENST00000419680.2	1.74	9.83	4.02E-09	7.92E-08	lincRNA
A_23_P145541	FIG4	-1.48	7.71	4.05E-09	7.96E-08	protein_coding
A_33_P3391418	TMEM40	1.57	5.82	4.05E-09	7.96E-08	protein_coding
A_33_P3260575	CERCAM	2.17	10.32	4.08E-09	8.01E-08	protein_coding
A_23_P169117	RRAGA	-1.65	11.88	4.09E-09	8.02E-08	protein_coding
A_23_P409168	NBEAL2	-1.47	8.28	4.09E-09	8.02E-08	protein_coding
A_23_P143535	WDR4	1.63	6.18	4.15E-09	8.14E-08	protein_coding
CUST_43634_PI428871386	ENST00000441492.1	2.13	6.81	4.15E-09	8.14E-08	lincRNA
A_24_P13083	TSPAN18	-1.99	8.45	4.15E-09	8.14E-08	protein_coding
A_24_P158421	SAR1A	-1.46	9.97	4.16E-09	8.15E-08	protein_coding
A_33_P3365193	AMY1C	-3.03	9.69	4.17E-09	8.16E-08	protein_coding
CUST_32649_PI428871386	ENST00000502125.2	-1.87	7.80	4.21E-09	8.25E-08	antisense

CUST_35053_PI428871386	ENST00000572547.1	-1.23	6.70	4.27E-09	8.36E-08	antisense
CUST_3669_PI428871386	ENST00000458044.1	-1.16	9.19	4.30E-09	8.42E-08	lincRNA
CUST_37304_PI428871386	ENST00000580756.1	-1.31	5.65	4.31E-09	8.42E-08	lincRNA
A_23_P155351	BTD	-1.43	6.16	4.32E-09	8.45E-08	protein_coding
A_23_P96833	SLAMF9	1.77	7.20	4.36E-09	8.52E-08	protein_coding
A_24_P161036	ACOT1	-2.13	11.04	4.43E-09	8.65E-08	protein_coding
A_23_P140373	FLVCR2	-1.52	6.21	4.50E-09	8.78E-08	protein_coding
A_23_P40347	HM13	1.17	10.62	4.51E-09	8.79E-08	protein_coding
A_33_P3228366	XPO5	1.69	7.33	4.52E-09	8.82E-08	protein_coding
A_33_P3325502	ARHGAP29	-1.47	5.86	4.56E-09	8.88E-08	protein_coding
A_33_P3330109	HLA-C	-2.31	15.85	4.56E-09	8.89E-08	protein_coding
A_24_P370887	VAMP3	-1.50	10.26	4.57E-09	8.89E-08	protein_coding
A_24_P411186	BCL11A	2.83	6.89	4.61E-09	8.97E-08	protein_coding
A_33_P3310232	L2HGDH	-1.64	15.00	4.64E-09	9.03E-08	protein_coding
A_33_P3276913	TTC3	1.48	6.97	4.65E-09	9.04E-08	protein_coding
A_33_P3256685	TTF2	1.50	8.48	4.65E-09	9.04E-08	protein_coding
A_24_P269432	BET1	1.85	7.66	4.65E-09	9.04E-08	protein_coding
CUST_37523_PI428871386	ENST00000580197.1	3.33	6.31	4.68E-09	9.09E-08	lincRNA
A_23_P142631	FKBP1B	-2.13	8.79	4.69E-09	9.12E-08	protein_coding
CUST_34129_PI428871386	ENST00000568767.1	-1.76	6.10	4.71E-09	9.16E-08	lincRNA
CUST_26734_PI428871386	ENST00000550468.1	1.35	7.64	4.73E-09	9.18E-08	antisense
A_33_P3276703	VGF	2.56	6.50	4.74E-09	9.20E-08	protein_coding
A_33_P3318414	HMHA1	-1.81	12.64	4.76E-09	9.23E-08	protein_coding
A_23_P373724	PPFIBP1	-1.48	9.92	4.82E-09	9.34E-08	protein_coding
CUST_26050_PI428871386	ENST00000539135.1	-1.60	6.24	4.86E-09	9.41E-08	antisense
A_33_P3216890	PAG1	-1.87	8.27	4.90E-09	9.49E-08	protein_coding
A_33_P3253249	FTH1	-1.94	16.15	4.93E-09	9.54E-08	protein_coding
A_24_P192805	CARD17	-2.24	8.64	4.94E-09	9.57E-08	protein_coding
CUST_17573_PI428871386	ENST00000428449.1	3.40	5.85	5.03E-09	9.74E-08	antisense
A_23_P212854	GYPB	-1.03	4.72	5.07E-09	9.81E-08	protein_coding
A_23_P202269	ANK3	-2.51	10.14	5.10E-09	9.87E-08	protein_coding
A_33_P3251896	APBB2	-1.52	6.58	5.13E-09	9.92E-08	protein_coding
A_23_P67466	PSMD8	1.47	11.85	5.13E-09	9.92E-08	protein_coding
CUST_34920_PI428871386	ENST00000577164.1	-1.52	5.96	5.20E-09	1.01E-07	lincRNA
CUST_13773_PI428871386	ENST00000519898.1	-1.32	6.75	5.21E-09	1.01E-07	lincRNA
CUST_5876_PI428871386	ENST00000308604.5	1.75	10.99	5.24E-09	1.01E-07	lincRNA
CUST_19989_PI428871386	ENST00000518528.1	2.38	6.34	5.25E-09	1.01E-07	lincRNA
CUST_16563_PI428871386	ENST00000430426.1	-1.17	5.14	5.26E-09	1.02E-07	lincRNA
CUST_24204_PI428871386	ENST00000531798.1	-1.12	4.88	5.34E-09	1.03E-07	lincRNA
CUST_10850_PI428871386	ENST00000511543.1	-1.06	5.07	5.35E-09	1.03E-07	antisense
CUST_43117_PI428871386	ENST00000381106.3	1.57	6.42	5.36E-09	1.03E-07	lincRNA
CUST_34868_PI428871386	ENST00000572499.1	-1.88	6.59	5.40E-09	1.04E-07	antisense
CUST_36005_PI428871386	ENST00000377540.1	-2.49	7.31	5.42E-09	1.05E-07	protein_coding
A_33_P3525263	A2ML1	1.70	5.09	5.43E-09	1.05E-07	protein_coding
A_23_P211252	LSS	-1.54	6.56	5.44E-09	1.05E-07	protein_coding
CUST_36567_PI428871386	ENST00000580022.1	-1.62	6.65	5.45E-09	1.05E-07	antisense
A_24_P401491	MORN5	-3.30	6.83	5.52E-09	1.06E-07	protein_coding
CUST_10685_PI428871386	ENST00000567197.1	2.86	6.27	5.56E-09	1.07E-07	lincRNA
CUST_16710_PI428871386	ENST00000582145.1	-1.30	7.34	5.58E-09	1.07E-07	antisense
CUST_42155_PI428871386	ENST00000414659.1	1.88	6.59	5.59E-09	1.07E-07	antisense
CUST_31129_PI428871386	ENST00000552334.1	-2.16	7.10	5.63E-09	1.08E-07	lincRNA
A_23_P308722	CCDC164	-2.73	6.45	5.65E-09	1.08E-07	protein_coding
A_23_P362659	MYD88	-1.81	9.44	5.65E-09	1.09E-07	protein_coding
A_23_P154447	NOP58	1.46	11.88	5.66E-09	1.09E-07	protein_coding
CUST_932_PI428871386	ENST00000446167.1	1.33	6.13	5.70E-09	1.09E-07	antisense
CUST_2501_PI428871386	ENST00000601909.1	2.30	6.59	5.75E-09	1.10E-07	lincRNA
A_24_P276888	CENPO	1.71	6.79	5.76E-09	1.10E-07	protein_coding
CUST_10221_PI428871386	ENST00000573308.1	1.69	7.76	5.77E-09	1.11E-07	antisense
A_33_P3285734	FCRL6	-1.49	5.86	5.79E-09	1.11E-07	protein_coding
CUST_5466_PI428871386	ENST00000423846.1	1.74	9.84	5.80E-09	1.11E-07	lincRNA
CUST_32626_PI428871386	ENST00000556899.1	-1.73	5.58	5.87E-09	1.12E-07	lincRNA
CUST_40987_PI428871386	ENST00000423536.1	1.69	9.21	5.95E-09	1.14E-07	lincRNA
A_33_P3302577	GPR133	-1.18	5.20	5.97E-09	1.14E-07	protein_coding

CUST_11838_PI428871386	ENST00000510284.1	-1.90	5.94	5.97E-09	1.14E-07	lincRNA
A_23_P52647	EHD1	-1.76	11.30	5.99E-09	1.14E-07	protein_coding
CUST_1243_PI428871386	ENST00000415842.1	-1.02	5.20	6.05E-09	1.16E-07	lincRNA
A_23_P53567	CCDC53	-1.27	9.71	6.14E-09	1.17E-07	protein_coding
A_23_P152066	UBR1	-1.47	9.08	6.24E-09	1.19E-07	protein_coding
A_23_P19510	HLA-DQB2	-3.20	10.04	6.26E-09	1.19E-07	protein_coding
A_23_P157580	SDCBP	-1.72	10.96	6.26E-09	1.19E-07	protein_coding
A_23_P1492	AVPI1	-1.93	9.71	6.27E-09	1.20E-07	protein_coding
CUST_17155_PI428871386	ENST00000426835.1	-1.62	6.43	6.29E-09	1.20E-07	antisense
CUST_17404_PI428871386	ENST00000449764.1	-1.43	5.00	6.31E-09	1.20E-07	antisense
CUST_12912_PI428871386	ENST00000507387.1	2.21	5.18	6.44E-09	1.22E-07	lincRNA
CUST_19986_PI428871386	ENST00000517790.1	2.08	6.36	6.57E-09	1.25E-07	lincRNA
A_23_P1320	MYOZ1	-1.51	5.96	6.60E-09	1.25E-07	protein_coding
CUST_24690_PI428871386	ENST00000541578.1	1.89	7.35	6.60E-09	1.25E-07	lincRNA
A_24_P322474	PDE4A	-2.09	10.64	6.67E-09	1.27E-07	protein_coding
A_33_P3411477	NCCRP1	2.46	7.85	6.71E-09	1.27E-07	protein_coding
CUST_6071_PI428871386	ENST00000567613.1	0.99	4.85	6.72E-09	1.28E-07	lincRNA
A_33_P3367361	FAM107B	-1.78	9.82	6.76E-09	1.28E-07	protein_coding
A_23_P118038	NUTF2	1.09	9.52	6.78E-09	1.29E-07	protein_coding
CUST_15292_PI428871386	ENST00000433843.1	-1.73	9.57	6.78E-09	1.29E-07	lincRNA
A_23_P39647	SLC4A3	1.84	6.40	6.80E-09	1.29E-07	protein_coding
A_23_P47682	NRIP3	2.16	6.77	6.82E-09	1.29E-07	protein_coding
CUST_22778_PI428871386	ENST00000394864.2	1.47	6.39	6.85E-09	1.30E-07	antisense
A_24_P50908	TRIM11	1.45	7.16	6.88E-09	1.30E-07	protein_coding
A_23_P129956	DUSP3	-1.40	9.63	6.89E-09	1.30E-07	protein_coding
CUST_43390_PI428871386	ENST00000561973.1	-1.47	5.75	6.91E-09	1.31E-07	lincRNA
A_23_P255701	LRRC48	-1.71	5.75	6.91E-09	1.31E-07	protein_coding
CUST_19973_PI428871386	ENST00000504719.2	2.23	6.23	6.91E-09	1.31E-07	lincRNA
A_32_P198923	YWHAZ	2.00	10.58	6.98E-09	1.32E-07	protein_coding
CUST_34314_PI428871386	ENST00000563823.2	1.14	5.24	6.99E-09	1.32E-07	lincRNA
A_33_P3262138	ANAPC1	1.39	6.81	7.01E-09	1.32E-07	protein_coding
A_33_P3841621	MYO5B	-1.74	6.65	7.02E-09	1.33E-07	protein_coding
CUST_7117_PI428871386	ENST00000430494.1	2.14	6.65	7.03E-09	1.33E-07	lincRNA
CUST_16264_PI428871386	ENST00000437621.2	-1.30	6.12	7.04E-09	1.33E-07	lincRNA
CUST_6553_PI428871386	ENST00000426475.1	-2.18	7.50	7.06E-09	1.33E-07	lincRNA
A_23_P214208	CNR1	-2.56	7.18	7.07E-09	1.33E-07	protein_coding
CUST_43511_PI428871386	ENST00000413528.1	-1.05	5.26	7.08E-09	1.33E-07	antisense
A_33_P3369178	PRG4	-2.03	5.62	7.11E-09	1.34E-07	protein_coding
A_24_P686965	SH2D5	2.09	7.04	7.18E-09	1.35E-07	protein_coding
CUST_11856_PI428871386	ENST00000514884.1	-1.12	5.07	7.19E-09	1.35E-07	antisense
A_23_P167599	FAM134B	-2.70	8.77	7.19E-09	1.35E-07	protein_coding
A_32_P356316	HLA-DOA	-3.31	11.70	7.19E-09	1.35E-07	protein_coding
CUST_20614_PI428871386	ENST00000436360.1	-2.20	9.04	7.21E-09	1.36E-07	antisense
CUST_18926_PI428871386	ENST00000518943.1	-1.19	5.23	7.33E-09	1.38E-07	lincRNA
A_24_P126181	NR2C2AP	1.49	8.46	7.34E-09	1.38E-07	protein_coding
A_23_P321223	PMCH	1.58	5.39	7.38E-09	1.39E-07	protein_coding
CUST_5461_PI428871386	ENST00000419680.2	1.64	9.93	7.41E-09	1.39E-07	lincRNA
A_23_P314642	ZNF280C	1.65	7.00	7.41E-09	1.39E-07	protein_coding
A_33_P3331085	SEC24A	1.47	6.94	7.42E-09	1.39E-07	protein_coding
CUST_34633_PI428871386	ENST00000599841.1	-1.40	5.58	7.45E-09	1.40E-07	lincRNA
A_23_P168610	TSPAN13	-2.19	9.64	7.48E-09	1.40E-07	protein_coding
CUST_34899_PI428871386	ENST00000334146.3	-2.01	7.39	7.54E-09	1.41E-07	lincRNA
CUST_17577_PI428871386	ENST00000424404.1	2.73	5.52	7.63E-09	1.43E-07	antisense
A_33_P3888485	BCO2	-1.55	6.57	7.70E-09	1.44E-07	protein_coding
A_23_P99661	ARHGEF40	-2.15	10.65	7.73E-09	1.45E-07	protein_coding
CUST_42160_PI428871386	ENST00000444998.1	1.20	5.93	7.74E-09	1.45E-07	antisense
CUST_29862_PI428871386	ENST00000554055.1	1.95	8.03	7.80E-09	1.46E-07	antisense
A_33_P3333863	CCZ1	1.25	8.89	7.85E-09	1.47E-07	protein_coding
CUST_38303_PI428871386	ENST00000579651.1	-1.82	6.00	7.88E-09	1.47E-07	lincRNA
CUST_8809_PI428871386	ENST00000510244.1	-1.55	7.73	7.96E-09	1.49E-07	lincRNA
A_24_P33895	ATF3	-2.65	8.15	7.97E-09	1.49E-07	protein_coding
A_33_P3279831	PNRC1	-1.58	10.09	7.97E-09	1.49E-07	protein_coding
A_24_P101114	CNOT1	1.36	7.34	7.98E-09	1.49E-07	protein_coding

A_33_P3305368	DDX31	1.39	7.06	8.02E-09	1.50E-07	protein_coding
A_24_P202840	SIRPB2	-1.50	6.55	8.02E-09	1.50E-07	protein_coding
A_33_P3340655	KLHL32	-1.26	5.14	8.04E-09	1.50E-07	protein_coding
A_24_P158385	ZMYND19	1.48	6.99	8.07E-09	1.51E-07	protein_coding
A_24_P216456	MAP1LC3C	-1.63	5.49	8.08E-09	1.51E-07	protein_coding
CUST_5880_PI428871386	ENST00000441075.1	1.79	9.46	8.13E-09	1.52E-07	lincRNA
CUST_21794_PI428871386	ENST00000584807.1	-2.36	6.26	8.13E-09	1.52E-07	lincRNA
A_33_P3418010	NUP62	1.27	9.93	8.19E-09	1.53E-07	protein_coding
A_24_P314477	TUBB2B	3.18	6.47	8.21E-09	1.53E-07	protein_coding
CUST_27538_PI428871386	ENST00000548886.1	-1.51	5.55	8.27E-09	1.54E-07	lincRNA
CUST_30834_PI428871386	ENST00000553575.1	-1.32	5.63	8.31E-09	1.55E-07	lincRNA
A_33_P3314643	SPEF1	-3.05	7.99	8.34E-09	1.55E-07	protein_coding
A_23_P211627	NUP50	1.37	7.21	8.36E-09	1.56E-07	protein_coding
CUST_5465_PI428871386	ENST00000455131.1	1.72	9.68	8.38E-09	1.56E-07	lincRNA
A_33_P3422728	CNTNAP3	-1.84	6.47	8.41E-09	1.56E-07	protein_coding
CUST_41501_PI428871386	ENST00000435697.1	-1.23	8.02	8.45E-09	1.57E-07	lincRNA
A_33_P3888629	MECOM	-2.26	9.27	8.51E-09	1.58E-07	protein_coding
CUST_11149_PI428871386	ENST00000508111.1	-2.44	7.67	8.54E-09	1.59E-07	lincRNA
CUST_14504_PI428871386	ENST00000427276.1	-1.68	5.14	8.63E-09	1.60E-07	lincRNA
A_23_P73801	TCEAL1	-1.38	9.24	8.63E-09	1.60E-07	protein_coding
A_33_P3333054	SYBU	-1.88	6.85	8.68E-09	1.61E-07	protein_coding
CUST_7214_PI428871386	ENST00000415479.1	1.64	5.20	8.75E-09	1.62E-07	lincRNA
A_33_P3290124	ASB10	-1.40	8.74	8.78E-09	1.63E-07	protein_coding
A_23_P1998	APBB1	-1.62	7.04	8.79E-09	1.63E-07	protein_coding
A_24_P35228	GRHL2	1.95	7.64	8.79E-09	1.63E-07	protein_coding
CUST_10684_PI428871386	ENST00000567197.1	2.70	6.06	8.86E-09	1.64E-07	lincRNA
CUST_19974_PI428871386	ENST00000504719.2	2.32	6.27	8.88E-09	1.64E-07	lincRNA
A_32_P220307	RPL39	1.35	15.11	8.95E-09	1.66E-07	protein_coding
A_32_P86763	TGM2	-2.58	9.15	9.00E-09	1.66E-07	protein_coding
A_33_P3628409	PKI55	-1.29	7.38	9.04E-09	1.67E-07	lincRNA
A_24_P827037	LRRC15	3.43	6.49	9.05E-09	1.67E-07	protein_coding
A_23_P259621	LAT2	-2.42	11.06	9.08E-09	1.68E-07	protein_coding
A_24_P149645	PUF60	1.38	11.12	9.09E-09	1.68E-07	protein_coding
CUST_33081_PI428871386	ENST00000570843.1	-1.68	6.60	9.21E-09	1.70E-07	lincRNA
CUST_20805_PI428871386	ENST00000446626.1	-1.62	9.06	9.22E-09	1.70E-07	lincRNA
A_33_P3328375	TTC29	-2.18	5.67	9.25E-09	1.71E-07	protein_coding
A_23_P94998	LETM1	1.54	7.47	9.26E-09	1.71E-07	protein_coding
CUST_31191_PI428871386	ENST00000561392.1	-1.40	5.33	9.30E-09	1.71E-07	lincRNA
A_33_P3209962	RASGRP2	-1.38	7.20	9.34E-09	1.72E-07	protein_coding
A_23_P85682	NFIA	-2.12	9.25	9.40E-09	1.73E-07	protein_coding
A_23_P86540	ARMC3	-3.00	6.63	9.41E-09	1.73E-07	protein_coding
CUST_30831_PI428871386	ENST00000557109.1	-1.40	5.70	9.43E-09	1.74E-07	lincRNA
CUST_40020_PI428871386	ENST00000602172.1	-1.80	8.55	9.45E-09	1.74E-07	antisense
CUST_30430_PI428871386	ENST00000555407.1	-1.65	6.01	9.45E-09	1.74E-07	antisense
A_23_P390596	PSKH1	-1.20	10.15	9.48E-09	1.74E-07	protein_coding
CUST_33314_PI428871386	ENST00000577048.1	-1.16	5.21	9.50E-09	1.75E-07	antisense
A_23_P218111	SERPINA1	-2.88	13.10	9.50E-09	1.75E-07	protein_coding
A_24_P522998	C1orf173	-2.62	6.20	9.56E-09	1.76E-07	protein_coding
A_33_P3332180	RBP3	1.01	4.86	9.60E-09	1.76E-07	protein_coding
A_33_P3386581	ANKRD40	-1.12	9.86	9.81E-09	1.80E-07	protein_coding
CUST_26735_PI428871386	ENST00000550468.1	1.40	7.76	9.84E-09	1.81E-07	antisense
CUST_33581_PI428871386	ENST00000565014.1	2.04	5.95	9.84E-09	1.81E-07	antisense
A_23_P132826	SERPINI2	-1.83	5.97	9.87E-09	1.81E-07	protein_coding
CUST_4305_PI428871386	ENST00000567540.1	-1.47	6.71	9.90E-09	1.82E-07	lincRNA
CUST_36749_PI428871386	ENST00000584047.1	2.02	6.49	1.00E-08	1.83E-07	lincRNA
CUST_34869_PI428871386	ENST00000572499.1	-1.80	6.60	1.01E-08	1.84E-07	antisense
CUST_37518_PI428871386	ENST00000578278.1	3.43	6.18	1.01E-08	1.84E-07	lincRNA
A_33_P3253574	OR8B3	-1.10	5.13	1.02E-08	1.87E-07	protein_coding
A_24_P944458	INSIG2	-1.52	7.80	1.03E-08	1.88E-07	protein_coding
A_33_P3274501	KLRF1	-1.70	5.69	1.03E-08	1.88E-07	protein_coding
A_23_P202138	SFXN3	-1.39	8.84	1.03E-08	1.89E-07	protein_coding
A_23_P31124	COL21A1	-3.09	7.61	1.04E-08	1.90E-07	protein_coding
CUST_8353_PI428871386	ENST00000474313.1	-1.23	5.49	1.04E-08	1.90E-07	antisense

A_33_P3294509	CD44	-2.64	13.30	1.05E-08	1.92E-07	protein_coding
A_33_P3418125	GLIPR1	-1.84	7.34	1.05E-08	1.92E-07	protein_coding
CUST_24066_PI428871386	ENST00000534477.1	3.59	6.43	1.06E-08	1.93E-07	lincRNA
A_23_P408285	PRICKLE1	-2.30	8.84	1.06E-08	1.94E-07	protein_coding
CUST_24681_PI428871386	ENST00000539975.1	1.93	10.26	1.07E-08	1.94E-07	lincRNA
A_33_P3286422	FANCA	1.78	5.64	1.07E-08	1.95E-07	protein_coding
A_23_P399255	RNF182	-2.99	7.03	1.08E-08	1.97E-07	protein_coding
A_23_P45955	TEKT2	-3.28	8.09	1.09E-08	1.99E-07	protein_coding
CUST_10689_PI428871386	ENST00000563602.1	2.13	5.39	1.10E-08	2.00E-07	lincRNA
A_23_P162386	BIN2	-1.65	7.28	1.10E-08	2.00E-07	protein_coding
A_33_P3311403	MRVI1	-2.28	9.88	1.10E-08	2.01E-07	protein_coding
A_23_P91850	IL20RB	3.13	7.79	1.11E-08	2.03E-07	protein_coding
CUST_10824_PI428871386	ENST00000318186.3	-1.20	5.62	1.12E-08	2.03E-07	antisense
A_33_P3260342	NFASC	-1.17	5.75	1.12E-08	2.04E-07	protein_coding
CUST_24874_PI428871386	ENST00000511677.1	-1.31	6.22	1.13E-08	2.05E-07	lincRNA
CUST_16591_PI428871386	ENST00000517641.1	-1.85	6.95	1.14E-08	2.07E-07	antisense
A_24_P743869	BBIP1	-1.18	5.81	1.14E-08	2.07E-07	protein_coding
CUST_38503_PI428871386	ENST00000586061.1	-1.35	5.71	1.14E-08	2.08E-07	lincRNA
A_33_P3245824	PQLC1	-1.40	10.56	1.14E-08	2.08E-07	protein_coding
CUST_33582_PI428871386	ENST00000565014.1	2.19	5.98	1.15E-08	2.08E-07	antisense
CUST_40346_PI428871386	ENST00000593109.1	-1.26	5.23	1.15E-08	2.09E-07	lincRNA
CUST_1821_PI428871386	ENST00000566942.1	-2.60	7.88	1.15E-08	2.09E-07	lincRNA
CUST_2500_PI428871386	ENST00000601909.1	2.25	6.60	1.16E-08	2.11E-07	lincRNA
A_23_P75063	DYDC2	-2.78	6.42	1.17E-08	2.11E-07	protein_coding
CUST_24064_PI428871386	ENST00000532541.1	3.59	6.57	1.17E-08	2.13E-07	lincRNA
CUST_36417_PI428871386	ENST00000499842.1	-1.86	5.88	1.17E-08	2.13E-07	lincRNA
A_23_P22672	ALG13	-1.49	9.14	1.18E-08	2.13E-07	protein_coding
CUST_43365_PI428871386	ENST00000374922.4	-1.59	8.00	1.18E-08	2.14E-07	antisense
CUST_13094_PI428871386	ENST00000504474.1	-1.51	5.98	1.18E-08	2.14E-07	antisense
A_23_P170901	PACRG	-1.76	6.88	1.19E-08	2.15E-07	protein_coding
A_24_P299318	FAM101B	-2.55	9.47	1.19E-08	2.16E-07	protein_coding
A_23_P28485	GCA	-1.60	11.10	1.21E-08	2.19E-07	protein_coding
A_23_P7325	BST1	-1.91	7.27	1.21E-08	2.20E-07	protein_coding
CUST_3280_PI428871386	ENST00000431096.1	2.96	10.10	1.22E-08	2.21E-07	lincRNA
A_24_P272967	AVL9	1.13	6.50	1.23E-08	2.22E-07	protein_coding
CUST_16106_PI428871386	ENST00000454185.1	-1.87	5.98	1.23E-08	2.23E-07	lincRNA
A_33_P3331588	ATAD3B	1.65	11.04	1.23E-08	2.23E-07	protein_coding
A_33_P3279019	SERPINB13	2.77	5.83	1.24E-08	2.23E-07	protein_coding
A_32_P106732	FANCM	1.27	7.74	1.24E-08	2.24E-07	protein_coding
A_23_P32913	ARMC10	1.43	9.90	1.25E-08	2.25E-07	protein_coding
A_33_P3221438	XXYLT1	1.90	8.60	1.25E-08	2.26E-07	protein_coding
A_23_P415643	ZNF48	1.71	7.74	1.28E-08	2.30E-07	protein_coding
A_23_P82859	OSGIN2	-1.10	6.49	1.28E-08	2.30E-07	protein_coding
A_23_P23017	C1orf123	-1.26	9.67	1.29E-08	2.31E-07	protein_coding
CUST_3116_PI428871386	ENST00000432296.1	-1.09	4.87	1.29E-08	2.33E-07	lincRNA
A_24_P586523	ALG1	1.45	8.07	1.30E-08	2.34E-07	protein_coding
A_24_P45446	GBP4	-2.44	10.11	1.30E-08	2.34E-07	protein_coding
CUST_11662_PI428871386	ENST00000512929.1	-1.14	4.75	1.30E-08	2.34E-07	antisense
A_23_P202071	CELF2	-2.15	9.30	1.32E-08	2.38E-07	protein_coding
A_23_P125705	NAP1L2	-1.65	5.94	1.33E-08	2.38E-07	protein_coding
CUST_40338_PI428871386	ENST00000591172.1	-1.26	5.28	1.33E-08	2.39E-07	lincRNA
CUST_43668_PI428871386	ENST00000429841.1	-2.38	6.81	1.33E-08	2.39E-07	antisense
A_33_P3407675	TIPRL	1.49	8.74	1.34E-08	2.40E-07	protein_coding
A_23_P209360	KLHL29	-2.41	8.95	1.35E-08	2.41E-07	protein_coding
CUST_5470_PI428871386	ENST00000437561.1	1.70	10.10	1.35E-08	2.42E-07	lincRNA
A_23_P160934	ANP32E	2.06	9.67	1.36E-08	2.44E-07	protein_coding
A_23_P23206	MAD2L2	1.81	8.57	1.37E-08	2.45E-07	protein_coding
A_23_P144096	CISH	-1.80	9.05	1.37E-08	2.45E-07	protein_coding
A_23_P168551	SLC29A4	1.84	6.01	1.37E-08	2.46E-07	protein_coding
A_32_P37592	SCARNA17	-1.94	12.03	1.40E-08	2.51E-07	antisense
A_23_P370434	C1QBP	1.32	12.85	1.40E-08	2.51E-07	protein_coding
CUST_3277_PI428871386	ENST00000429156.1	2.99	10.11	1.41E-08	2.52E-07	lincRNA
A_23_P213199	DNAJB14	-1.47	9.52	1.43E-08	2.55E-07	protein_coding

CUST_19992_PI428871386	ENST00000523068.1	2.07	6.39	1.43E-08	2.55E-07	lincRNA
A_33_P3282634	ALDH1L2	1.74	6.80	1.43E-08	2.56E-07	protein_coding
CUST_17700_PI428871386	ENST00000447307.1	-1.67	6.41	1.44E-08	2.56E-07	lincRNA
A_23_P63681	IDE	1.50	8.70	1.44E-08	2.56E-07	protein_coding
A_23_P46928	PFKP	2.14	11.02	1.44E-08	2.57E-07	protein_coding
CUST_6580_PI428871386	ENST00000436922.1	1.30	5.49	1.45E-08	2.58E-07	antisense
A_33_P3610123	POLR1A	1.28	8.19	1.45E-08	2.58E-07	protein_coding
A_23_P133543	KLHL3	-1.80	7.91	1.45E-08	2.59E-07	protein_coding
A_33_P3420235	ARPC5	-1.55	6.99	1.45E-08	2.59E-07	protein_coding
A_33_P3260445	DYNLT1	-1.29	6.46	1.46E-08	2.60E-07	protein_coding
A_23_P79968	PCSK2	-1.70	5.32	1.48E-08	2.63E-07	protein_coding
A_24_P392958	TBC1D20	-1.34	7.10	1.48E-08	2.63E-07	protein_coding
CUST_29059_PI428871386	ENST00000437334.1	-1.22	5.16	1.49E-08	2.64E-07	lincRNA
CUST_43666_PI428871386	ENST00000454385.1	-2.23	6.65	1.49E-08	2.65E-07	antisense
A_33_P3350621	CNRIP1	-1.17	5.49	1.49E-08	2.65E-07	protein_coding
CUST_34800_PI428871386	ENST00000562855.1	-2.44	7.88	1.50E-08	2.66E-07	protein_coding
CUST_16355_PI428871386	ENST00000424460.1	1.54	5.71	1.54E-08	2.73E-07	antisense
A_33_P3227920	SLC16A4	-2.82	9.25	1.54E-08	2.73E-07	protein_coding
A_32_P8156	C10orf107	-1.84	6.22	1.56E-08	2.77E-07	protein_coding
CUST_5244_PI428871386	ENST00000416395.1	-1.54	8.26	1.56E-08	2.77E-07	antisense
CUST_23695_PI428871386	ENST00000553459.1	-1.30	4.75	1.58E-08	2.80E-07	antisense
CUST_11837_PI428871386	ENST00000511703.1	-1.63	5.82	1.58E-08	2.81E-07	lincRNA
CUST_17882_PI428871386	ENST00000489488.1	-1.46	6.42	1.59E-08	2.81E-07	antisense
A_23_P258088	PACSIN1	1.91	6.98	1.59E-08	2.82E-07	protein_coding
A_23_P342709	FBXO15	-2.18	7.21	1.60E-08	2.83E-07	protein_coding
CUST_37519_PI428871386	ENST00000578278.1	3.25	6.28	1.61E-08	2.85E-07	lincRNA
A_33_P3395758	C14orf28	-1.23	5.96	1.62E-08	2.86E-07	protein_coding
A_33_P3221843	COQ3	1.29	6.00	1.62E-08	2.86E-07	protein_coding
A_24_P113264	FBXO27	2.12	6.51	1.62E-08	2.86E-07	protein_coding
A_33_P3343972	RSPH1	-3.81	8.53	1.64E-08	2.90E-07	protein_coding
CUST_2398_PI428871386	ENST00000452399.1	2.06	8.63	1.64E-08	2.90E-07	lincRNA
CUST_36598_PI428871386	ENST00000587125.1	1.41	7.86	1.67E-08	2.94E-07	lincRNA
A_23_P136683	HLA-DQB1	-2.69	10.95	1.67E-08	2.95E-07	protein_coding
A_23_P317465	RAB8B	-1.43	7.67	1.69E-08	2.98E-07	protein_coding
A_23_P426196	MAST3	-1.42	7.38	1.70E-08	2.99E-07	protein_coding
CUST_35333_PI428871386	ENST00000484836.1	1.66	6.93	1.71E-08	3.01E-07	antisense
A_33_P3391135	GPR133	-1.38	5.91	1.72E-08	3.03E-07	protein_coding
CUST_8301_PI428871386	ENST00000479018.1	1.39	5.33	1.73E-08	3.06E-07	antisense
CUST_37083_PI428871386	ENST00000587575.1	2.25	6.74	1.74E-08	3.06E-07	lincRNA
A_23_P212595	VPRBP	-0.99	5.80	1.74E-08	3.07E-07	protein_coding
CUST_27689_PI428871386	ENST00000548846.1	-1.75	6.48	1.75E-08	3.08E-07	lincRNA
A_24_P305933	TMCC3	-1.50	7.19	1.75E-08	3.08E-07	protein_coding
A_23_P38584	KRT27	-1.61	5.47	1.75E-08	3.08E-07	protein_coding
A_32_P177024	SBDS	-1.41	9.49	1.75E-08	3.08E-07	protein_coding
CUST_18748_PI428871386	ENST00000519764.1	1.58	5.20	1.76E-08	3.09E-07	lincRNA
A_24_P408704	DOCK2	-1.60	6.70	1.77E-08	3.11E-07	protein_coding
A_33_P3212112	SLC12A6	-1.44	8.08	1.78E-08	3.13E-07	protein_coding
A_33_P3413038	PLXNB3	1.56	5.87	1.78E-08	3.13E-07	protein_coding
A_32_P7316	BDNF	-1.25	5.08	1.80E-08	3.16E-07	protein_coding
CUST_8234_PI428871386	ENST00000294241.6	-1.11	10.68	1.80E-08	3.16E-07	protein_coding
A_23_P42884	MRPS24	1.38	13.11	1.82E-08	3.19E-07	protein_coding
CUST_16105_PI428871386	ENST00000454185.1	-1.86	5.96	1.83E-08	3.21E-07	lincRNA
A_33_P3341601	WDR86	2.63	7.52	1.84E-08	3.23E-07	protein_coding
CUST_21793_PI428871386	ENST00000584807.1	-2.09	6.04	1.85E-08	3.24E-07	lincRNA
CUST_23339_PI428871386	ENST00000369884.4	-1.31	6.16	1.86E-08	3.26E-07	antisense
A_23_P36464	C12orf11	1.62	8.40	1.87E-08	3.27E-07	protein_coding
A_33_P3391496	SLIT3	2.35	7.57	1.88E-08	3.29E-07	protein_coding
A_23_P90014	GPR108	-1.42	8.72	1.89E-08	3.31E-07	protein_coding
A_33_P3211263	GPATCH4	1.47	9.48	1.90E-08	3.32E-07	protein_coding
CUST_43115_PI428871386	ENST00000451781.1	1.48	6.55	1.91E-08	3.34E-07	lincRNA
A_23_P255876	DNAI1	-1.89	5.79	1.93E-08	3.37E-07	protein_coding
A_33_P3211634	PPIL6	-1.73	6.14	1.94E-08	3.40E-07	protein_coding
CUST_19983_PI428871386	ENST00000523427.1	2.17	6.21	1.95E-08	3.40E-07	lincRNA

A_32_P2452	TMTC1	-2.59	8.71	1.96E-08	3.43E-07	protein_coding
CUST_13192_PI428871386	ENST00000508339.1	2.24	5.48	1.97E-08	3.44E-07	lincRNA
A_23_P165698	C2orf49	1.47	7.10	1.98E-08	3.45E-07	protein_coding
A_33_P3252359	BDH1	2.09	8.33	1.98E-08	3.45E-07	protein_coding
A_24_P276791	LRRC42	1.15	6.09	1.99E-08	3.47E-07	protein_coding
A_23_P61426	MSRA	-2.30	9.28	2.00E-08	3.48E-07	protein_coding
A_23_P29225	FBXO7	-1.23	11.19	2.00E-08	3.49E-07	protein_coding
CUST_10946_PI428871386	ENST00000513793.1	2.12	5.42	2.01E-08	3.51E-07	lincRNA
A_23_P141505	CLEC10A	-2.51	7.94	2.02E-08	3.53E-07	protein_coding
A_23_P320829	CEP128	1.36	6.20	2.06E-08	3.59E-07	protein_coding
CUST_27688_PI428871386	ENST00000548846.1	-1.62	6.29	2.06E-08	3.59E-07	lincRNA
A_24_P110799	LSS	-1.49	9.44	2.08E-08	3.62E-07	protein_coding
CUST_27226_PI428871386	ENST00000426250.2	-1.45	5.26	2.09E-08	3.64E-07	antisense
A_33_P3387951	KIAA1244	-2.26	8.26	2.13E-08	3.71E-07	protein_coding
A_33_P3392537	TK2	-1.36	7.08	2.14E-08	3.71E-07	protein_coding
A_23_P129157	NEIL1	-1.85	8.53	2.15E-08	3.74E-07	protein_coding
A_23_P83453	SMARCC1	1.31	8.95	2.15E-08	3.74E-07	protein_coding
A_23_P96285	REEP1	-2.11	6.64	2.16E-08	3.75E-07	protein_coding
A_33_P3239569	RPS21	1.46	6.23	2.16E-08	3.76E-07	protein_coding
A_24_P172993	UBE2O	1.38	9.80	2.17E-08	3.77E-07	protein_coding
A_33_P3362562	RPS6KB1	1.25	7.16	2.17E-08	3.77E-07	protein_coding
A_23_P87257	MRPL17	1.54	8.79	2.19E-08	3.80E-07	protein_coding
A_24_P350744	KIAA0907	1.53	6.71	2.21E-08	3.83E-07	protein_coding
CUST_11857_PI428871386	ENST00000514884.1	-1.04	5.09	2.22E-08	3.84E-07	antisense
A_23_P137139	BTK	-1.93	7.03	2.22E-08	3.84E-07	protein_coding
CUST_6499_PI428871386	ENST00000425636.2	1.33	5.64	2.23E-08	3.86E-07	antisense
CUST_8114_PI428871386	ENST00000431705.1	-1.17	5.23	2.25E-08	3.89E-07	antisense
CUST_34997_PI428871386	ENST00000577064.1	-1.31	5.90	2.28E-08	3.94E-07	antisense
A_33_P3404779	TCTEX1D4	-1.93	6.30	2.28E-08	3.94E-07	protein_coding
A_33_P3629678	COL5A1	2.46	11.37	2.28E-08	3.94E-07	protein_coding
CUST_29863_PI428871386	ENST00000554055.1	1.99	7.94	2.28E-08	3.95E-07	antisense
A_33_P3328254	IL5RA	-1.52	6.09	2.31E-08	3.99E-07	protein_coding
A_33_P3256695	PRPF40A	-1.53	8.31	2.31E-08	3.99E-07	protein_coding
A_23_P340717	ONECUT1	1.96	5.35	2.31E-08	4.00E-07	protein_coding
A_24_P253723	MIR22HG	-1.81	9.22	2.34E-08	4.04E-07	lincRNA
A_32_P36235	IER2	-2.25	14.45	2.35E-08	4.06E-07	protein_coding
CUST_24549_PI428871386	ENST00000534162.1	1.04	5.49	2.35E-08	4.06E-07	antisense
A_33_P3274069	RHBDD3	1.21	9.95	2.36E-08	4.08E-07	protein_coding
CUST_31306_PI428871386	ENST00000559232.1	-1.06	5.02	2.39E-08	4.12E-07	lincRNA
CUST_24670_PI428871386	ENST00000540725.1	1.88	11.57	2.39E-08	4.13E-07	lincRNA
CUST_931_PI428871386	ENST00000446167.1	1.36	6.12	2.40E-08	4.14E-07	antisense
CUST_17293_PI428871386	ENST00000435257.1	2.49	5.34	2.41E-08	4.16E-07	antisense
A_33_P3241316	PLA2G4F	-1.04	5.50	2.42E-08	4.17E-07	protein_coding
A_23_P78888	FBL	1.77	13.79	2.42E-08	4.17E-07	protein_coding
A_23_P417974	AQP11	1.51	5.52	2.44E-08	4.20E-07	protein_coding
A_23_P34578	GNL2	1.29	10.27	2.44E-08	4.20E-07	protein_coding
A_33_P3250680	CD40LG	-2.22	6.79	2.46E-08	4.23E-07	protein_coding
A_33_P3329023	FAM69A	1.49	6.00	2.47E-08	4.26E-07	protein_coding
A_33_P3289696	OSCP1	-1.48	6.72	2.48E-08	4.27E-07	protein_coding
CUST_8297_PI428871386	ENST00000462497.1	1.07	5.28	2.49E-08	4.28E-07	antisense
CUST_33286_PI428871386	ENST00000571639.1	1.93	5.91	2.51E-08	4.32E-07	lincRNA
CUST_26026_PI428871386	ENST00000513358.2	-1.57	6.94	2.52E-08	4.34E-07	lincRNA
CUST_5597_PI428871386	ENST00000594273.1	-1.13	5.18	2.54E-08	4.37E-07	antisense
CUST_16266_PI428871386	ENST00000457484.2	-1.19	6.10	2.56E-08	4.40E-07	lincRNA
A_23_P212002	NKTR	-1.26	10.09	2.60E-08	4.47E-07	protein_coding
A_23_P27810	ZNF607	1.39	8.43	2.61E-08	4.49E-07	protein_coding
A_33_P3339860	PLEKHA2	-1.95	11.61	2.63E-08	4.51E-07	protein_coding
CUST_31888_PI428871386	ENST00000558050.1	-1.08	4.70	2.66E-08	4.57E-07	antisense
A_33_P3399935	RPS6KA2	-1.22	6.13	2.68E-08	4.60E-07	protein_coding
CUST_8939_PI428871386	ENST00000474250.1	1.78	7.86	2.73E-08	4.68E-07	antisense
CUST_32988_PI428871386	ENST00000562807.1	-1.14	8.49	2.75E-08	4.71E-07	lincRNA
CUST_2558_PI428871386	ENST00000430312.1	-1.38	5.64	2.77E-08	4.74E-07	antisense
A_33_P3215134	ZNF777	1.30	7.12	2.77E-08	4.74E-07	protein_coding

A_24_P653603	C17orf89	1.54	12.70	2.80E-08	4.79E-07	protein_coding
CUST_10687_PI428871386	ENST00000562355.1	2.54	5.83	2.80E-08	4.80E-07	lincRNA
A_23_P332960	TMEM80	-1.76	10.03	2.81E-08	4.80E-07	protein_coding
A_24_P82466	GAS7	-1.56	6.32	2.82E-08	4.83E-07	protein_coding
CUST_42164_PI428871386	ENST00000421927.1	1.24	5.96	2.84E-08	4.85E-07	antisense
A_23_P131990	VSX1	-1.21	6.14	2.85E-08	4.87E-07	protein_coding
A_24_P22976	ARRDC2	-1.67	8.51	2.85E-08	4.87E-07	protein_coding
A_24_P383850	EIF4G3	1.27	7.24	2.91E-08	4.97E-07	protein_coding
A_33_P3278118	CASP3	1.55	7.11	2.91E-08	4.97E-07	protein_coding
A_33_P3242548	PRDM5	-1.48	6.77	2.92E-08	4.98E-07	protein_coding
A_23_P200560	CDC42	-1.15	12.97	2.95E-08	5.03E-07	protein_coding
CUST_22774_PI428871386	ENST00000457147.1	1.42	6.28	2.95E-08	5.04E-07	antisense
CUST_5471_PI428871386	ENST00000437561.1	1.63	9.92	2.97E-08	5.06E-07	lincRNA
A_23_P119362	EMP3	-1.88	10.48	2.97E-08	5.06E-07	protein_coding
A_24_P287941	PSMC3IP	1.66	6.97	2.98E-08	5.07E-07	protein_coding
CUST_16107_PI428871386	ENST00000439207.1	-1.22	5.14	2.99E-08	5.09E-07	lincRNA
CUST_37035_PI428871386	ENST00000366365.2	1.53	6.64	2.99E-08	5.09E-07	lincRNA
CUST_11596_PI428871386	ENST00000507152.1	2.29	5.57	3.01E-08	5.13E-07	protein_coding
CUST_15684_PI428871386	ENST00000451017.1	-1.34	4.96	3.03E-08	5.15E-07	antisense
A_33_P3256168	SNX30	-1.29	6.46	3.05E-08	5.20E-07	protein_coding
A_33_P3409886	VAMP2	-0.96	8.41	3.06E-08	5.21E-07	protein_coding
CUST_24687_PI428871386	ENST00000542112.1	1.94	9.37	3.09E-08	5.25E-07	lincRNA
A_23_P31602	BUD31	1.03	10.24	3.10E-08	5.28E-07	protein_coding
A_23_P38244	APOH	-3.64	7.38	3.11E-08	5.29E-07	protein_coding
A_33_P3535649	FLYWCH1	-1.26	6.13	3.12E-08	5.30E-07	protein_coding
CUST_578_PI428871386	ENST00000566551.1	1.45	6.32	3.12E-08	5.30E-07	lincRNA
A_33_P3392882	NCKAP5	-1.79	6.08	3.14E-08	5.34E-07	protein_coding
CUST_4888_PI428871386	ENST00000421759.1	-1.31	6.13	3.14E-08	5.34E-07	antisense
A_23_P210253	DGKD	-1.61	9.84	3.18E-08	5.39E-07	protein_coding
CUST_37744_PI428871386	ENST00000579431.1	-1.29	5.69	3.19E-08	5.40E-07	lincRNA
A_33_P3287760	RASGEF1A	1.89	6.25	3.19E-08	5.40E-07	protein_coding
CUST_30688_PI428871386	ENST00000360899.2	-1.03	8.82	3.19E-08	5.41E-07	lincRNA
A_24_P180680	LAPTM4B	2.14	12.00	3.20E-08	5.43E-07	protein_coding
A_24_P22079	FOXO1	-1.67	8.55	3.24E-08	5.49E-07	protein_coding
A_24_P852756	HLA-DQA2	-2.94	11.08	3.26E-08	5.53E-07	protein_coding
A_33_P3390284	FAM122B	1.25	6.29	3.27E-08	5.54E-07	protein_coding
CUST_20753_PI428871386	ENST00000435586.1	-1.50	8.47	3.28E-08	5.55E-07	lincRNA
A_23_P252052	FILIP1L	-1.92	8.90	3.28E-08	5.55E-07	protein_coding
CUST_43880_PI428871386	ENST00000328819.4	-1.10	6.76	3.29E-08	5.57E-07	lincRNA
CUST_32693_PI428871386	ENST00000559321.1	3.66	5.85	3.30E-08	5.58E-07	lincRNA
CUST_31328_PI428871386	ENST00000560484.1	-1.04	4.82	3.30E-08	5.58E-07	lincRNA
A_23_P204579	TDG	1.42	10.87	3.30E-08	5.58E-07	protein_coding
A_23_P81392	WWC1	-2.04	10.33	3.30E-08	5.58E-07	protein_coding
A_24_P924862	RAPH1	-1.74	9.87	3.30E-08	5.58E-07	protein_coding
CUST_43116_PI428871386	ENST00000451781.1	1.42	6.26	3.34E-08	5.64E-07	lincRNA
A_33_P3359027	CCDC42B	-1.81	5.74	3.34E-08	5.64E-07	protein_coding
A_33_P3276997	MAP6	-2.15	7.53	3.36E-08	5.67E-07	protein_coding
A_23_P73809	LRCH2	-1.71	6.33	3.36E-08	5.67E-07	protein_coding
A_33_P3362498	ATR	1.37	7.60	3.39E-08	5.71E-07	protein_coding
A_24_P153568	MPEG1	-2.22	9.36	3.39E-08	5.71E-07	protein_coding
A_23_P200792	NOTCH2	-1.56	11.37	3.40E-08	5.74E-07	protein_coding
A_23_P88626	ANPEP	-1.83	7.01	3.40E-08	5.74E-07	protein_coding
A_24_P382489	SLC27A1	-1.73	10.95	3.41E-08	5.75E-07	protein_coding
CUST_3278_PI428871386	ENST00000366437.3	2.85	9.90	3.42E-08	5.75E-07	lincRNA
A_23_P350001	GUCY1A2	-1.72	6.76	3.46E-08	5.83E-07	protein_coding
A_33_P3365432	NCF4	-1.93	8.39	3.50E-08	5.89E-07	protein_coding
CUST_35151_PI428871386	ENST00000585303.1	-1.10	5.11	3.51E-08	5.90E-07	antisense
CUST_17578_PI428871386	ENST00000424404.1	2.96	5.54	3.51E-08	5.90E-07	antisense
CUST_3835_PI428871386	ENST00000566446.1	-1.33	9.66	3.51E-08	5.90E-07	lincRNA
A_24_P253251	SLC7A1	1.67	7.71	3.54E-08	5.94E-07	protein_coding
CUST_4211_PI428871386	ENST00000418970.1	1.78	5.62	3.54E-08	5.94E-07	antisense
CUST_17552_PI428871386	ENST00000436097.1	1.01	5.03	3.62E-08	6.07E-07	antisense
A_32_P143000	FAM189A1	-1.74	6.22	3.63E-08	6.08E-07	protein_coding

CUST_17311_PI428871386	ENST00000416502.1	-1.39	5.68	3.63E-08	6.08E-07	antisense
CUST_14439_PI428871386	ENST00000561592.1	1.95	5.35	3.66E-08	6.14E-07	antisense
A_23_P61202	FAM207A	1.51	8.23	3.67E-08	6.15E-07	protein_coding
A_24_P229638	C9orf117	-2.12	6.25	3.70E-08	6.20E-07	protein_coding
CUST_17517_PI428871386	ENST00000439070.1	-1.34	5.25	3.70E-08	6.20E-07	antisense
A_32_P103945	B4GALT4	1.66	8.20	3.72E-08	6.22E-07	protein_coding
CUST_33080_PI428871386	ENST00000570843.1	-1.71	6.69	3.75E-08	6.28E-07	lincRNA
A_33_P3443165	RAE1	1.45	11.25	3.76E-08	6.28E-07	protein_coding
CUST_22776_PI428871386	ENST00000457758.1	1.36	6.29	3.81E-08	6.36E-07	antisense
CUST_11150_PI428871386	ENST00000511919.1	-2.32	7.67	3.82E-08	6.39E-07	lincRNA
A_23_P28688	CPSF3	1.36	9.09	3.82E-08	6.39E-07	protein_coding
CUST_3276_PI428871386	ENST00000429156.1	2.93	10.32	3.83E-08	6.40E-07	lincRNA
CUST_29427_PI428871386	ENST00000535351.1	-1.00	4.75	3.85E-08	6.43E-07	antisense
A_33_P3233843	IL6ST	-1.42	7.52	3.90E-08	6.51E-07	protein_coding
A_33_P3822503	CTF1	-1.37	8.57	3.90E-08	6.51E-07	protein_coding
A_24_P66001	UQCR10	-1.53	8.11	3.91E-08	6.53E-07	protein_coding
A_33_P3336257	IRX1	-3.12	8.95	3.91E-08	6.53E-07	protein_coding
A_23_P212675	NME9	-1.34	5.36	3.92E-08	6.53E-07	protein_coding
A_23_P145514	IL20RA	-2.08	7.31	3.92E-08	6.54E-07	protein_coding
CUST_24063_PI428871386	ENST00000504230.2	3.26	6.38	3.92E-08	6.54E-07	lincRNA
A_23_P76761	VRK1	1.66	8.20	3.93E-08	6.55E-07	protein_coding
CUST_26582_PI428871386	ENST00000563933.1	-1.73	5.96	3.94E-08	6.56E-07	lincRNA
A_23_P258689	HEATR2	1.17	8.96	3.97E-08	6.61E-07	protein_coding
A_24_P124558	HOXC8	2.14	5.69	4.01E-08	6.67E-07	protein_coding
CUST_1774_PI428871386	ENST00000554749.1	-1.47	7.91	4.01E-08	6.67E-07	antisense
A_24_P406334	STEAP1	2.18	7.43	4.03E-08	6.70E-07	protein_coding
A_23_P104073	S100A3	-2.23	8.11	4.05E-08	6.73E-07	protein_coding
A_23_P251795	GPC2	2.17	7.10	4.06E-08	6.74E-07	protein_coding
CUST_42027_PI428871386	ENST00000448049.1	1.81	5.82	4.09E-08	6.79E-07	lincRNA
A_23_P72025	SLC25A20	-1.50	7.41	4.12E-08	6.83E-07	protein_coding
A_23_P111981	LYNX1	-1.46	7.55	4.13E-08	6.86E-07	protein_coding
CUST_11013_PI428871386	ENST00000436413.1	1.44	5.49	4.17E-08	6.91E-07	antisense
A_23_P45799	ORC1	1.81	5.34	4.21E-08	6.98E-07	protein_coding
CUST_26797_PI428871386	ENST00000564531.1	-1.60	6.04	4.28E-08	7.09E-07	lincRNA
A_23_P47800	DIABLO	1.06	10.13	4.30E-08	7.11E-07	protein_coding
A_33_P3401156	ETV1	-1.76	7.00	4.31E-08	7.13E-07	protein_coding
CUST_36968_PI428871386	ENST00000578226.1	1.15	5.16	4.33E-08	7.16E-07	antisense
A_24_P27977	TRPM2	1.94	7.37	4.33E-08	7.17E-07	protein_coding
A_33_P3276856	HYDIN	-1.66	5.96	4.35E-08	7.19E-07	protein_coding
A_23_P155288	GFM1	1.44	9.44	4.35E-08	7.20E-07	protein_coding
CUST_25886_PI428871386	ENST00000446631.1	-3.02	7.15	4.39E-08	7.25E-07	antisense
A_23_P259172	SSR4	1.60	13.20	4.39E-08	7.25E-07	protein_coding
CUST_17574_PI428871386	ENST00000428449.1	2.18	5.93	4.40E-08	7.26E-07	antisense
A_33_P3224735	ZC3H12B	-1.27	5.66	4.42E-08	7.30E-07	protein_coding
A_23_P250413	PARVG	-1.94	7.72	4.45E-08	7.34E-07	protein_coding
A_23_P78037	CCL7	1.96	5.52	4.47E-08	7.37E-07	protein_coding
CUST_17880_PI428871386	ENST00000498397.1	-1.31	5.86	4.47E-08	7.37E-07	antisense
CUST_17362_PI428871386	ENST00000412754.1	1.47	6.34	4.47E-08	7.37E-07	antisense
A_23_P70643	C6orf103	-1.69	5.46	4.51E-08	7.44E-07	protein_coding
CUST_3317_PI428871386	ENST00000448567.1	-1.13	4.85	4.53E-08	7.47E-07	lincRNA
CUST_29233_PI428871386	ENST00000425483.1	-1.01	8.49	4.55E-08	7.49E-07	lincRNA
CUST_42156_PI428871386	ENST00000414659.1	1.65	6.46	4.58E-08	7.54E-07	antisense
A_33_P3313125	ENST00000429567	1.87	5.41	4.63E-08	7.61E-07	antisense
CUST_6549_PI428871386	ENST00000442456.1	-1.58	5.59	4.67E-08	7.68E-07	lincRNA
A_33_P3421733	EIF3C	1.18	9.95	4.82E-08	7.92E-07	protein_coding
CUST_21981_PI428871386	ENST00000545372.1	2.41	5.56	4.85E-08	7.96E-07	antisense
A_33_P3280521	MFAP3L	-2.08	7.55	4.87E-08	8.00E-07	protein_coding
A_23_P135474	MRPL37	1.41	10.25	4.87E-08	8.00E-07	protein_coding
A_23_P73837	TLR8	-2.14	7.04	4.88E-08	8.00E-07	protein_coding
A_33_P3415052	NIPAL4	2.99	5.96	4.89E-08	8.03E-07	protein_coding
CUST_39065_PI428871386	ENST00000594678.1	-1.01	4.84	4.89E-08	8.03E-07	antisense
A_33_P3240543	AGAP3	-1.59	10.63	4.92E-08	8.07E-07	protein_coding
CUST_24672_PI428871386	ENST00000537925.1	1.80	11.86	4.93E-08	8.09E-07	lincRNA

CUST_37859_PI428871386	ENST00000581856.1	1.89	5.29	4.96E-08	8.14E-07	antisense
A_33_P3316903	ENST00000519753	-1.16	6.43	4.98E-08	8.17E-07	antisense
A_23_P83818	COL5A1	2.12	7.04	5.04E-08	8.26E-07	protein_coding
A_23_P30223	SRD5A1	1.75	6.58	5.07E-08	8.29E-07	protein_coding
CUST_27687_PI428871386	ENST00000552663.1	-1.57	6.40	5.09E-08	8.33E-07	lincRNA
A_23_P436369	FILIP1	-1.93	7.22	5.10E-08	8.35E-07	protein_coding
CUST_25004_PI428871386	ENST00000532530.1	-1.33	6.51	5.11E-08	8.36E-07	lincRNA
CUST_35055_PI428871386	ENST00000573222.1	-1.34	7.45	5.14E-08	8.41E-07	lincRNA
A_33_P3846177	B4GALNT1	2.62	6.64	5.14E-08	8.41E-07	protein_coding
A_23_P503127	SEPT3_	1.83	5.99	5.15E-08	8.42E-07	protein_coding
A_24_P273143	LINC00152	1.59	9.67	5.23E-08	8.55E-07	lincRNA
CUST_508_PI428871386	ENST00000439577.1	1.48	6.87	5.30E-08	8.66E-07	antisense
CUST_8341_PI428871386	ENST00000485770.1	-1.35	7.13	5.32E-08	8.69E-07	lincRNA
CUST_22775_PI428871386	ENST00000457147.1	1.31	6.38	5.37E-08	8.77E-07	antisense
A_23_P160849	FCER1G	-1.95	11.64	5.40E-08	8.81E-07	protein_coding
A_33_P3539223	C9orf100	1.63	5.74	5.43E-08	8.86E-07	protein_coding
A_23_P58337	FIP1L1	1.29	9.87	5.49E-08	8.95E-07	protein_coding
CUST_24693_PI428871386	ENST00000538266.1	1.86	8.12	5.50E-08	8.96E-07	lincRNA
A_33_P3214988	EFCAB1	-2.38	6.22	5.52E-08	8.99E-07	protein_coding
CUST_2459_PI428871386	ENST00000577853.1	1.90	7.26	5.53E-08	9.01E-07	antisense
A_23_P59613	FZD9	1.47	6.81	5.55E-08	9.04E-07	protein_coding
A_33_P3293049	HLA-DQA1	-2.81	12.23	5.70E-08	9.28E-07	protein_coding
A_33_P3217437	GBP6	2.49	5.64	5.72E-08	9.31E-07	protein_coding
CUST_3279_PI428871386	ENST00000366437.3	2.85	10.32	5.74E-08	9.33E-07	lincRNA
CUST_39100_PI428871386	ENST00000597028.1	-1.42	13.31	5.74E-08	9.35E-07	antisense
A_23_P216630	SLC44A1	1.41	11.36	5.75E-08	9.35E-07	protein_coding
A_23_P250156	IGF2BP2	2.37	8.14	5.75E-08	9.35E-07	protein_coding
A_33_P3892608	KCNT2	-1.33	5.75	5.82E-08	9.47E-07	protein_coding
A_33_P3363655	ANAPC1	1.40	6.45	5.83E-08	9.47E-07	protein_coding
A_23_P65870	FBXO22	1.52	8.32	5.85E-08	9.50E-07	protein_coding
A_24_P116669	CANT1	1.32	6.41	5.85E-08	9.51E-07	protein_coding
CUST_36193_PI428871386	ENST00000344686.2	-1.58	10.09	5.91E-08	9.59E-07	protein_coding
A_23_P165355	FARSB	1.39	7.57	5.97E-08	9.68E-07	protein_coding
CUST_11382_PI428871386	ENST00000503066.1	-1.68	6.76	5.98E-08	9.70E-07	antisense
A_33_P3253394	LAIR1	-2.68	10.45	6.00E-08	9.73E-07	protein_coding
A_23_P134854	CLDN23	-1.76	8.55	6.00E-08	9.73E-07	protein_coding
A_23_P166826	DCP1A	-1.08	10.73	6.01E-08	9.73E-07	protein_coding
CUST_918_PI428871386	ENST00000444386.1	-1.04	5.02	6.04E-08	9.79E-07	antisense
CUST_34538_PI428871386	ENST00000564138.1	-1.68	7.04	6.05E-08	9.79E-07	protein_coding
CUST_22372_PI428871386	ENST00000426283.1	1.64	7.69	6.09E-08	9.86E-07	lincRNA
A_23_P52738	DCPS	1.39	9.28	6.13E-08	9.92E-07	protein_coding
CUST_25176_PI428871386	ENST00000527321.1	1.18	9.57	6.31E-08	1.02E-06	antisense
A_23_P135977	CKAP5	1.20	8.91	6.33E-08	1.02E-06	protein_coding
CUST_1021_PI428871386	ENST00000588291.1	-1.21	6.44	6.39E-08	1.03E-06	antisense
A_23_P34537	EPHX1	-1.67	9.46	6.40E-08	1.03E-06	protein_coding
A_33_P3531857	DARS	1.14	11.53	6.43E-08	1.04E-06	protein_coding
A_23_P13701	TMBIM4	-1.48	9.80	6.44E-08	1.04E-06	protein_coding
A_23_P423074	FAM169A	2.30	6.54	6.46E-08	1.04E-06	protein_coding
A_33_P3360972	EXOC3L4	-1.72	7.21	6.47E-08	1.04E-06	protein_coding
CUST_12051_PI428871386	ENST00000514848.1	-0.88	4.92	6.48E-08	1.04E-06	antisense
A_23_P500433	CARD9	-2.13	8.48	6.63E-08	1.07E-06	protein_coding
A_24_P944222	TMTC3	1.44	6.34	6.67E-08	1.07E-06	protein_coding
A_23_P308581	RRP36	1.19	11.71	6.76E-08	1.09E-06	protein_coding
A_23_P136012	FBXO8	-1.29	8.12	6.81E-08	1.10E-06	protein_coding
A_23_P204304	PTPRO	-2.21	6.91	6.82E-08	1.10E-06	protein_coding
CUST_39973_PI428871386	ENST00000598170.1	2.07	5.58	6.85E-08	1.10E-06	lincRNA
A_33_P3334313	ACTR3B	1.65	6.68	6.86E-08	1.10E-06	protein_coding
A_33_P3334743	RAPGEF2	-1.30	7.92	6.87E-08	1.11E-06	protein_coding
A_23_P96641	PRPS2	1.43	6.48	6.87E-08	1.11E-06	protein_coding
A_24_P85775	C1orf38	-2.29	8.83	7.01E-08	1.13E-06	protein_coding
A_23_P205098	PDS5B	-1.33	7.41	7.02E-08	1.13E-06	protein_coding
A_33_P3209279	SASH1	-1.20	6.70	7.14E-08	1.15E-06	protein_coding
A_23_P137103	EIF4A1	1.28	12.12	7.15E-08	1.15E-06	protein_coding

A_23_P63026	LGALS8	1.42	8.06	7.19E-08	1.15E-06	protein_coding
A_33_P3246007	APOA1BP	1.57	11.19	7.20E-08	1.15E-06	protein_coding
A_23_P134744	RNF122	-1.91	9.98	7.22E-08	1.16E-06	protein_coding
A_33_P3215422	LYL1	-1.40	6.38	7.26E-08	1.16E-06	protein_coding
A_33_P3331345	DUSP11	1.15	6.62	7.28E-08	1.17E-06	protein_coding
CUST_21999_PI428871386	ENST00000454321.1	-1.48	6.76	7.29E-08	1.17E-06	antisense
A_23_P48977	MRPS11	1.18	9.96	7.30E-08	1.17E-06	protein_coding
A_23_P209477	PPM1G	1.38	9.18	7.30E-08	1.17E-06	protein_coding
A_24_P205130	FNBP1	-1.50	8.39	7.31E-08	1.17E-06	protein_coding
A_23_P206684	WWP2	-1.29	8.23	7.33E-08	1.17E-06	protein_coding
A_23_P157726	DENND4C	-1.40	9.53	7.34E-08	1.17E-06	protein_coding
CUST_40443_PI428871386	ENST00000596379.1	2.40	7.05	7.43E-08	1.19E-06	lincRNA
A_24_P913115	PTEN	-1.59	8.41	7.43E-08	1.19E-06	protein_coding
CUST_17310_PI428871386	ENST00000416502.1	-1.32	5.81	7.46E-08	1.19E-06	antisense
CUST_2458_PI428871386	ENST00000577853.1	1.95	7.33	7.47E-08	1.19E-06	antisense
CUST_20899_PI428871386	ENST00000429567.1	2.13	5.49	7.53E-08	1.20E-06	antisense
CUST_6550_PI428871386	ENST00000429172.1	-1.81	7.98	7.67E-08	1.22E-06	lincRNA
CUST_20438_PI428871386	ENST00000447950.1	-1.15	5.01	7.70E-08	1.23E-06	lincRNA
A_33_P3405424	IL4I1	2.62	10.18	7.73E-08	1.23E-06	protein_coding
A_33_P3209866	KANK1	-1.52	8.49	7.76E-08	1.24E-06	protein_coding
A_33_P3334443	FAM69A	1.50	8.87	7.77E-08	1.24E-06	protein_coding
A_33_P3251369	GAB3	-1.66	6.98	7.86E-08	1.25E-06	protein_coding
A_33_P3420380	ITIH5	-1.64	6.16	7.87E-08	1.25E-06	protein_coding
A_33_P3257187	PRKAA1	-1.24	7.87	7.90E-08	1.26E-06	protein_coding
CUST_9149_PI428871386	ENST00000461943.1	-1.13	5.61	7.90E-08	1.26E-06	antisense
A_33_P3502640	DTX2	1.48	7.53	7.96E-08	1.27E-06	protein_coding
CUST_26878_PI428871386	ENST00000439545.1	1.10	5.14	7.96E-08	1.27E-06	antisense
A_24_P171182	ACBD3	1.09	7.31	7.98E-08	1.27E-06	protein_coding
A_33_P3331376	EPHB2	1.54	5.86	7.99E-08	1.27E-06	protein_coding
A_23_P68978	EFCAB6	-1.34	5.83	8.03E-08	1.28E-06	protein_coding
CUST_8324_PI428871386	ENST00000468961.2	-1.01	6.06	8.04E-08	1.28E-06	antisense
A_33_P3327500	UFSP1	1.16	6.36	8.06E-08	1.28E-06	protein_coding
A_33_P3279353	AZU1	-1.21	5.21	8.06E-08	1.28E-06	protein_coding
CUST_8077_PI428871386	ENST00000422681.1	-1.12	6.00	8.09E-08	1.29E-06	antisense
A_33_P3307500	STRA6	1.94	5.29	8.10E-08	1.29E-06	protein_coding
A_24_P96593	EVI5	-1.20	7.74	8.12E-08	1.29E-06	protein_coding
CUST_2774_PI428871386	ENST00000420691.1	2.12	5.58	8.12E-08	1.29E-06	lincRNA
A_33_P3249595	BCL11A	1.73	5.68	8.13E-08	1.29E-06	protein_coding
A_24_P413669	PFKFB2	-1.42	6.25	8.18E-08	1.30E-06	protein_coding
A_23_P76435	GATC	1.37	6.96	8.20E-08	1.30E-06	protein_coding
CUST_3281_PI428871386	ENST00000431096.1	2.81	10.16	8.23E-08	1.31E-06	lincRNA
A_23_P28318	C2orf56	1.26	9.28	8.26E-08	1.31E-06	protein_coding
A_33_P3231878	PIK3C2A	-1.26	10.71	8.27E-08	1.31E-06	protein_coding
A_23_P70095	CD74	-2.71	9.96	8.27E-08	1.31E-06	protein_coding
CUST_15126_PI428871386	ENST00000585945.1	-1.33	5.46	8.29E-08	1.31E-06	antisense
CUST_21623_PI428871386	ENST00000455981.1	-1.47	6.04	8.33E-08	1.32E-06	lincRNA
CUST_18962_PI428871386	ENST00000520881.1	-1.34	5.38	8.38E-08	1.33E-06	lincRNA
CUST_8808_PI428871386	ENST00000510244.1	-1.40	8.12	8.42E-08	1.33E-06	lincRNA
A_33_P3359012	DUSP8	-1.22	10.47	8.47E-08	1.34E-06	protein_coding
A_23_P130352	KCTD1	1.96	7.26	8.50E-08	1.34E-06	protein_coding
CUST_10384_PI428871386	ENST00000504219.1	-1.18	5.37	8.52E-08	1.35E-06	antisense
A_23_P46356	TNFAIP8L2	-1.93	8.45	8.56E-08	1.35E-06	protein_coding
A_23_P71889	ODF2	1.29	6.55	8.57E-08	1.35E-06	protein_coding
A_24_P278747	CCND2	-1.90	8.87	8.60E-08	1.36E-06	protein_coding
CUST_5464_PI428871386	ENST00000455131.1	1.56	9.86	8.64E-08	1.36E-06	lincRNA
CUST_17363_PI428871386	ENST00000412754.1	1.39	6.27	8.66E-08	1.37E-06	antisense
A_23_P154675	SNRPB	1.54	10.44	8.68E-08	1.37E-06	protein_coding
CUST_24674_PI428871386	ENST00000537068.1	1.80	11.35	8.69E-08	1.37E-06	lincRNA
CUST_507_PI428871386	ENST00000439577.1	1.55	7.01	8.72E-08	1.38E-06	antisense
A_23_P134650	PTCD1	1.32	10.12	8.75E-08	1.38E-06	protein_coding
CUST_42162_PI428871386	ENST00000455567.1	1.66	6.56	8.77E-08	1.38E-06	antisense
A_23_P148984	DARS2	1.74	6.88	8.89E-08	1.40E-06	protein_coding
CUST_3834_PI428871386	ENST00000566446.1	-1.30	9.85	8.91E-08	1.40E-06	lincRNA

A_23_P162142	TSKU	1.65	8.95	8.95E-08	1.41E-06	protein_coding
CUST_3290_PI428871386	ENST00000440276.1	2.87	10.23	8.96E-08	1.41E-06	lincRNA
CUST_18149_PI428871386	ENST00000518009.1	-1.06	5.59	9.01E-08	1.42E-06	antisense
CUST_6244_PI428871386	ENST00000422799.1	-1.10	5.03	9.03E-08	1.42E-06	antisense
CUST_4774_PI428871386	ENST00000599268.1	-1.00	4.87	9.06E-08	1.42E-06	antisense
CUST_4083_PI428871386	ENST00000450794.1	-1.03	4.61	9.09E-08	1.43E-06	lincRNA
A_23_P40049	CAD	1.35	8.72	9.09E-08	1.43E-06	protein_coding
CUST_40621_PI428871386	ENST00000441428.1	0.95	4.83	9.14E-08	1.43E-06	antisense
CUST_17319_PI428871386	ENST00000431497.1	2.05	5.35	9.15E-08	1.44E-06	antisense
A_32_P173298	SF3B3	1.41	7.10	9.32E-08	1.46E-06	protein_coding
A_23_P164237	UTP6	1.18	8.13	9.32E-08	1.46E-06	protein_coding
CUST_40444_PI428871386	ENST00000596379.1	2.41	6.93	9.49E-08	1.49E-06	lincRNA
A_23_P85922	BMP8A	1.68	5.62	9.51E-08	1.49E-06	protein_coding
CUST_33288_PI428871386	ENST00000575792.1	1.81	5.98	9.53E-08	1.49E-06	lincRNA
A_23_P34741	ZNF593	1.26	10.75	9.58E-08	1.50E-06	protein_coding
CUST_42898_PI428871386	ENST00000416406.1	-1.67	7.36	9.58E-08	1.50E-06	antisense
A_23_P431410	RBMS1	-1.47	12.25	9.66E-08	1.51E-06	protein_coding
A_23_P255805	ZNF7	1.30	8.69	9.68E-08	1.52E-06	protein_coding
CUST_20829_PI428871386	ENST00000305709.5	-1.53	9.41	9.73E-08	1.52E-06	lincRNA
CUST_19880_PI428871386	ENST00000530778.1	1.23	6.39	9.74E-08	1.52E-06	lincRNA
CUST_4986_PI428871386	ENST00000427421.1	-1.21	5.14	9.79E-08	1.53E-06	lincRNA
A_33_P3335525	FCRL5	1.94	5.56	9.80E-08	1.53E-06	protein_coding
A_24_P89512	BCLAF1	-1.09	11.10	9.85E-08	1.54E-06	protein_coding
CUST_25144_PI428871386	ENST00000528075.1	1.89	5.95	9.89E-08	1.54E-06	antisense
A_23_P14559	RIPK3	-1.58	6.82	9.90E-08	1.55E-06	protein_coding
CUST_8298_PI428871386	ENST00000475371.1	1.24	5.34	9.94E-08	1.55E-06	antisense
A_23_P128372	FKBP4	1.12	10.00	9.97E-08	1.56E-06	protein_coding
A_33_P3251198	PLCB1	1.90	6.75	9.98E-08	1.56E-06	protein_coding
A_23_P20578	FAM166B	-1.95	6.32	1.01E-07	1.58E-06	protein_coding
A_23_P8961	IL7	-1.60	7.46	1.02E-07	1.58E-06	protein_coding
A_23_P8269	TAAR8	-1.09	4.99	1.02E-07	1.58E-06	protein_coding
A_23_P27215	UBB	-1.38	14.73	1.02E-07	1.59E-06	protein_coding
CUST_5462_PI428871386	ENST00000414584.1	1.56	10.84	1.02E-07	1.59E-06	lincRNA
A_24_P82155	ANKDD1A	-1.08	5.66	1.03E-07	1.60E-06	protein_coding
A_33_P3313830	FLJ33630	-1.26	7.17	1.03E-07	1.60E-06	lincRNA
CUST_8300_PI428871386	ENST00000479018.1	1.17	5.28	1.03E-07	1.60E-06	antisense
A_23_P96542	VMA21	1.52	9.87	1.03E-07	1.61E-06	protein_coding
A_23_P137573	LEFTY2	-1.56	5.27	1.04E-07	1.61E-06	protein_coding
CUST_24065_PI428871386	ENST00000532541.1	3.50	6.99	1.04E-07	1.61E-06	lincRNA
A_24_P363278	MAK	-1.63	5.65	1.04E-07	1.61E-06	protein_coding
CUST_13139_PI428871386	ENST00000512856.1	-1.30	5.83	1.04E-07	1.62E-06	antisense
CUST_20806_PI428871386	ENST00000446626.1	-1.46	8.56	1.04E-07	1.62E-06	lincRNA
A_23_P76488	EMP1	-2.72	12.66	1.05E-07	1.63E-06	protein_coding
A_23_P150693	FJX1	1.90	8.40	1.06E-07	1.64E-06	protein_coding
A_33_P3402404	SCN3B	-1.89	6.56	1.06E-07	1.65E-06	protein_coding
CUST_24678_PI428871386	ENST00000537869.1	1.76	11.63	1.07E-07	1.66E-06	lincRNA
A_23_P355517	SYNPO2L	-1.35	5.69	1.07E-07	1.66E-06	protein_coding
CUST_33287_PI428871386	ENST00000571639.1	1.84	5.96	1.08E-07	1.67E-06	lincRNA
A_23_P14804	TSPAN3	-1.72	12.22	1.08E-07	1.68E-06	protein_coding
CUST_22857_PI428871386	ENST00000416398.1	-1.90	6.26	1.09E-07	1.68E-06	lincRNA
CUST_15192_PI428871386	ENST00000455530.1	-1.58	5.71	1.09E-07	1.69E-06	lincRNA
A_23_P62081	SCG5	3.15	6.74	1.10E-07	1.70E-06	protein_coding
A_24_P329635	TSC1	-1.26	8.73	1.11E-07	1.72E-06	protein_coding
CUST_20710_PI428871386	ENST00000455995.1	-1.32	5.66	1.12E-07	1.73E-06	lincRNA
A_24_P222872	UGT1A6	3.10	6.07	1.12E-07	1.73E-06	protein_coding
A_33_P3341365	RNF216	1.31	8.93	1.12E-07	1.73E-06	protein_coding
A_33_P3213432	ARMC10	1.27	9.70	1.13E-07	1.74E-06	protein_coding
CUST_2545_PI428871386	ENST00000452962.1	1.52	7.15	1.13E-07	1.74E-06	antisense
A_23_P139146	MS4A8B	-2.98	7.02	1.13E-07	1.75E-06	protein_coding
A_23_P127288	IL2RA	2.31	6.82	1.13E-07	1.75E-06	protein_coding
A_32_P342064	FTH1	-1.76	15.75	1.13E-07	1.75E-06	protein_coding
A_23_P60990	C2orf54	-2.16	6.95	1.13E-07	1.75E-06	protein_coding
CUST_34539_PI428871386	ENST00000564138.1	-1.65	6.88	1.14E-07	1.75E-06	protein_coding

CUST_42028_PI428871386	ENST00000448049.1	1.72	5.81	1.14E-07	1.75E-06	lincRNA
A_33_P3241984	PTPN22	-1.98	7.80	1.14E-07	1.76E-06	protein_coding
CUST_9943_PI428871386	ENST00000452051.1	-0.97	4.87	1.15E-07	1.77E-06	antisense
CUST_18449_PI428871386	ENST00000521025.1	-1.40	6.59	1.15E-07	1.77E-06	antisense
A_24_P38143	AHI1	-1.26	7.11	1.15E-07	1.77E-06	protein_coding
A_32_P234827	ARMC1	1.32	9.47	1.16E-07	1.78E-06	protein_coding
A_23_P167595	UBE2B	-1.17	7.24	1.16E-07	1.78E-06	protein_coding
CUST_22286_PI428871386	ENST00000414457.1	-1.15	6.37	1.16E-07	1.78E-06	antisense
CUST_42260_PI428871386	ENST00000440005.1	2.18	5.83	1.16E-07	1.79E-06	antisense
CUST_43106_PI428871386	ENST00000456563.1	1.65	8.23	1.16E-07	1.79E-06	lincRNA
A_33_P3269919	C1orf129	-1.16	4.76	1.17E-07	1.79E-06	protein_coding
CUST_34994_PI428871386	ENST00000577176.1	-1.19	5.99	1.19E-07	1.83E-06	antisense
A_24_P312519	PBLD	1.35	6.36	1.20E-07	1.85E-06	protein_coding
A_33_P3405531	TMF1	-1.21	7.57	1.21E-07	1.85E-06	protein_coding
CUST_24686_PI428871386	ENST00000542112.1	1.61	9.24	1.22E-07	1.87E-06	lincRNA
CUST_8299_PI428871386	ENST00000475371.1	1.22	5.37	1.22E-07	1.87E-06	antisense
A_33_P3291244	CCDC60	-1.79	5.86	1.22E-07	1.88E-06	protein_coding
A_32_P217510	WDR75	1.39	8.91	1.23E-07	1.88E-06	protein_coding
A_23_P14105	RCBTB2	-1.52	8.19	1.23E-07	1.88E-06	protein_coding
A_23_P66328	ACSM2B	-1.04	5.21	1.23E-07	1.88E-06	protein_coding
A_33_P3329467	STYXL1	1.28	10.93	1.23E-07	1.89E-06	protein_coding
A_33_P3210099	ALPK3	-2.19	8.72	1.23E-07	1.89E-06	protein_coding
CUST_13384_PI428871386	ENST00000512105.1	-1.76	5.76	1.23E-07	1.89E-06	lincRNA
A_23_P50269	CXCL17	-1.79	11.15	1.24E-07	1.89E-06	protein_coding
CUST_32381_PI428871386	ENST00000561409.1	-0.95	5.14	1.25E-07	1.91E-06	antisense
CUST_9956_PI428871386	ENST00000424769.1	1.71	7.79	1.26E-07	1.92E-06	antisense
A_23_P149975	FAM107B	-1.73	10.68	1.26E-07	1.93E-06	protein_coding
CUST_28281_PI428871386	ENST00000445646.2	-1.22	7.39	1.27E-07	1.94E-06	lincRNA
A_23_P51699	ARHGEF2	-1.34	7.92	1.27E-07	1.95E-06	protein_coding
A_23_P98382	TIMM8B	1.39	12.96	1.27E-07	1.95E-06	protein_coding
CUST_36563_PI428871386	ENST00000578025.1	-1.43	6.69	1.28E-07	1.96E-06	antisense
A_33_P3402868	GRIN2D	1.89	6.35	1.29E-07	1.97E-06	protein_coding
CUST_5473_PI428871386	ENST00000413202.1	1.61	11.08	1.29E-07	1.98E-06	lincRNA
CUST_43033_PI428871386	ENST00000438810.1	1.67	5.28	1.30E-07	1.98E-06	lincRNA
CUST_3538_PI428871386	ENST00000440540.1	-2.76	6.89	1.30E-07	1.98E-06	antisense
A_33_P3242623	SLC7A11	3.70	7.72	1.30E-07	1.99E-06	protein_coding
CUST_2544_PI428871386	ENST00000452962.1	1.52	6.86	1.31E-07	2.00E-06	antisense
A_24_P171268	RASSF5	-1.76	8.83	1.32E-07	2.01E-06	protein_coding
CUST_22018_PI428871386	ENST00000449648.1	-2.09	8.22	1.33E-07	2.03E-06	lincRNA
A_24_P310256	LGI4	-1.41	7.52	1.33E-07	2.03E-06	protein_coding
CUST_22145_PI428871386	ENST00000437232.1	-1.51	7.07	1.34E-07	2.05E-06	antisense
A_33_P3268378	FAM167A	-1.19	5.50	1.35E-07	2.06E-06	protein_coding
A_23_P202219	CALHM2	-1.87	8.64	1.38E-07	2.09E-06	protein_coding
CUST_30302_PI428871386	ENST00000556781.1	-1.04	4.99	1.39E-07	2.12E-06	lincRNA
CUST_6551_PI428871386	ENST00000429172.1	-1.77	7.82	1.40E-07	2.13E-06	lincRNA
A_24_P208567	IL18R1	-1.84	6.95	1.40E-07	2.13E-06	protein_coding
CUST_20830_PI428871386	ENST00000305709.5	-1.44	7.59	1.40E-07	2.13E-06	lincRNA
CUST_32630_PI428871386	ENST00000556053.1	-1.10	4.76	1.40E-07	2.13E-06	lincRNA
CUST_8842_PI428871386	ENST00000485218.1	0.88	5.59	1.41E-07	2.14E-06	antisense
A_23_P70818	SMO	2.27	8.18	1.41E-07	2.15E-06	protein_coding
A_23_P165937	DSN1	1.49	8.62	1.43E-07	2.17E-06	protein_coding
A_23_P324523	IQCK	-1.38	8.94	1.44E-07	2.19E-06	protein_coding
A_23_P108244	COX6B1	1.32	12.91	1.46E-07	2.21E-06	protein_coding
A_33_P3414482	GOT2	1.19	12.49	1.47E-07	2.24E-06	protein_coding
CUST_41809_PI428871386	ENST00000599421.1	1.91	6.80	1.48E-07	2.25E-06	antisense
CUST_9958_PI428871386	ENST00000415244.1	1.66	7.79	1.49E-07	2.25E-06	antisense
A_33_P3880302	EPHB2	1.80	6.13	1.49E-07	2.25E-06	protein_coding
A_33_P3272773	ZNF217	1.49	8.79	1.49E-07	2.26E-06	protein_coding
CUST_33896_PI428871386	ENST00000569993.1	-1.93	5.64	1.50E-07	2.27E-06	lincRNA
CUST_9959_PI428871386	ENST00000415244.1	1.63	7.95	1.50E-07	2.27E-06	antisense
CUST_20878_PI428871386	ENST00000590767.1	-1.28	7.80	1.52E-07	2.30E-06	antisense
A_23_P357284	GPR4	-1.87	7.41	1.52E-07	2.31E-06	protein_coding
CUST_9824_PI428871386	ENST00000415451.1	-1.45	5.94	1.53E-07	2.31E-06	antisense

CUST_24684_PI428871386	ENST00000545440.1	1.63	9.12	1.53E-07	2.32E-06	lincRNA
CUST_12035_PI428871386	ENST00000508201.1	-0.93	4.50	1.53E-07	2.32E-06	lincRNA
A_23_P17144	GPN1	1.32	10.69	1.53E-07	2.32E-06	protein_coding
CUST_3298_PI428871386	ENST00000441672.1	-1.52	6.30	1.55E-07	2.34E-06	antisense
A_24_P77681	PAIP1	1.25	9.26	1.55E-07	2.35E-06	protein_coding
A_33_P3415097	CLCN4	-2.08	7.57	1.56E-07	2.35E-06	protein_coding
CUST_17881_PI428871386	ENST00000489488.1	-1.33	6.20	1.56E-07	2.36E-06	antisense
A_24_P63136	P2RY13	-1.55	5.88	1.56E-07	2.36E-06	protein_coding
CUST_4752_PI428871386	ENST00000449569.1	-1.13	6.02	1.57E-07	2.37E-06	antisense
A_24_P16326	ROBO2	-1.47	6.10	1.57E-07	2.37E-06	protein_coding
A_23_P372925	KIAA1737	-1.15	8.99	1.57E-07	2.38E-06	protein_coding
A_32_P78816	PSPH	2.51	9.53	1.58E-07	2.39E-06	protein_coding
A_23_P75769	MS4A4A	-2.60	10.74	1.60E-07	2.41E-06	protein_coding
A_33_P3306192	KBTBD13	1.53	8.79	1.60E-07	2.41E-06	protein_coding
A_23_P29924	TMEM128	-1.36	8.61	1.60E-07	2.41E-06	protein_coding
A_24_P658427	NFIB	-2.12	10.04	1.61E-07	2.43E-06	protein_coding
A_33_P3257891	GSTA3	-1.45	5.93	1.61E-07	2.43E-06	protein_coding
CUST_19009_PI428871386	ENST00000530033.1	-1.10	4.88	1.62E-07	2.45E-06	lincRNA
A_24_P237804	POTED	3.47	5.63	1.63E-07	2.45E-06	protein_coding
A_23_P67932	CXCR1	-1.77	5.90	1.64E-07	2.47E-06	protein_coding
A_32_P170444	SUB1	-1.46	8.84	1.65E-07	2.48E-06	protein_coding
CUST_6092_PI428871386	ENST00000375987.3	-1.45	6.50	1.65E-07	2.48E-06	lincRNA
CUST_276_PI428871386	ENST00000452079.1	-1.47	7.07	1.65E-07	2.48E-06	antisense
CUST_37932_PI428871386	ENST00000589281.1	-1.43	6.20	1.66E-07	2.50E-06	antisense
A_23_P218784	DDX17	-1.59	11.53	1.67E-07	2.50E-06	protein_coding
A_24_P374445	C17orf57	-1.11	5.31	1.67E-07	2.51E-06	protein_coding
CUST_26051_PI428871386	ENST00000539135.1	-1.48	6.30	1.67E-07	2.51E-06	antisense
CUST_31517_PI428871386	ENST00000560750.1	-1.15	6.44	1.69E-07	2.54E-06	lincRNA
A_23_P202978	CASP1	-2.21	9.89	1.71E-07	2.57E-06	protein_coding
A_23_P151232	TMEM132C	-1.20	5.34	1.73E-07	2.59E-06	protein_coding
A_24_P282237	PRIM2	1.34	5.64	1.74E-07	2.61E-06	protein_coding
CUST_25784_PI428871386	ENST00000524453.1	-1.02	5.16	1.75E-07	2.62E-06	antisense
A_33_P3332970	CLEC2B	-1.81	7.91	1.75E-07	2.63E-06	protein_coding
A_33_P3411741	SIDT2	-1.39	7.18	1.78E-07	2.66E-06	protein_coding
A_33_P3285580	GLYCTK	-1.34	6.24	1.80E-07	2.70E-06	protein_coding
A_32_P223140	RASGEF1A	1.87	6.09	1.81E-07	2.72E-06	protein_coding
CUST_39101_PI428871386	ENST00000597028.1	-1.38	13.01	1.82E-07	2.72E-06	antisense
A_24_P411815	C7orf11	1.07	9.02	1.82E-07	2.72E-06	protein_coding
A_33_P3214012	HMGCLL1	-1.18	5.83	1.83E-07	2.74E-06	protein_coding
CUST_16720_PI428871386	ENST00000579174.1	-1.28	7.45	1.84E-07	2.75E-06	antisense
CUST_29232_PI428871386	ENST00000425483.1	-0.98	8.50	1.84E-07	2.75E-06	lincRNA
A_33_P3344406	MEF2B	1.43	7.22	1.85E-07	2.76E-06	protein_coding
A_23_P79628	PSME4	1.40	8.22	1.86E-07	2.77E-06	protein_coding
A_23_P161615	POLA2	1.36	6.91	1.86E-07	2.77E-06	protein_coding
A_23_P94319	KBTBD11	-2.27	9.73	1.86E-07	2.78E-06	protein_coding
CUST_2593_PI428871386	ENST00000441085.1	2.10	5.26	1.88E-07	2.81E-06	lincRNA
CUST_25887_PI428871386	ENST00000446631.1	-2.72	6.93	1.88E-07	2.81E-06	antisense
A_23_P87329	NAT10	1.10	8.67	1.89E-07	2.81E-06	protein_coding
CUST_38796_PI428871386	ENST00000592371.1	-0.98	4.61	1.89E-07	2.81E-06	lincRNA
CUST_38502_PI428871386	ENST00000586061.1	-1.20	5.42	1.90E-07	2.83E-06	lincRNA
CUST_17706_PI428871386	ENST00000423414.1	-1.08	6.24	1.91E-07	2.84E-06	antisense
CUST_5868_PI428871386	ENST00000409569.2	1.53	10.91	1.91E-07	2.84E-06	lincRNA
A_33_P3342375	MAGEA6	5.67	6.25	1.93E-07	2.88E-06	protein_coding
A_33_P3330404	FAM180B	-1.31	6.57	1.93E-07	2.88E-06	protein_coding
CUST_34996_PI428871386	ENST00000577064.1	-1.21	5.91	1.94E-07	2.89E-06	antisense
A_32_P149251	DNAJC18	-1.17	7.06	1.95E-07	2.90E-06	protein_coding
A_23_P382188	STAP2	1.57	8.33	1.95E-07	2.91E-06	protein_coding
A_23_P131526	C2orf47	1.18	9.31	1.96E-07	2.91E-06	protein_coding
CUST_43667_PI428871386	ENST00000429841.1	-1.99	6.45	1.99E-07	2.95E-06	antisense
CUST_37758_PI428871386	ENST00000581274.1	1.89	5.65	1.99E-07	2.96E-06	antisense
A_32_P74579	GAS2L2	-1.45	5.98	2.01E-07	2.98E-06	protein_coding
A_33_P3285809	SSX2IP	1.61	6.35	2.04E-07	3.02E-06	protein_coding
A_23_P75978	CLPB	1.55	7.95	2.04E-07	3.03E-06	protein_coding

CUST_5490_PI428871386	ENST00000567067.1	-1.37	8.54	2.06E-07	3.05E-06	lincRNA
A_23_P13852	STRAP	1.32	11.89	2.06E-07	3.06E-06	protein_coding
A_23_P359647	NFAT5	-1.34	10.39	2.08E-07	3.08E-06	protein_coding
CUST_17784_PI428871386	ENST00000425981.2	0.92	4.91	2.09E-07	3.09E-06	antisense
A_33_P3377229	C11orf21	-1.15	6.30	2.09E-07	3.09E-06	protein_coding
CUST_32631_PI428871386	ENST00000556053.1	-1.11	4.79	2.09E-07	3.10E-06	lincRNA
A_23_P118435	SUMO2	1.13	11.75	2.09E-07	3.10E-06	protein_coding
A_33_P3341474	ZBTB38	-1.19	6.70	2.09E-07	3.10E-06	protein_coding
A_33_P3377364	ITGB4	2.25	10.51	2.10E-07	3.11E-06	protein_coding
A_32_P69166	ANKRD42	-1.05	6.18	2.10E-07	3.11E-06	protein_coding
CUST_9245_PI428871386	ENST00000474477.1	-1.55	6.83	2.11E-07	3.12E-06	lincRNA
A_24_P941831	TMEM237	-1.73	7.49	2.12E-07	3.13E-06	protein_coding
A_23_P153745	IFI30	-2.24	14.04	2.13E-07	3.15E-06	protein_coding
A_33_P3333488	HAGH	-1.53	11.96	2.14E-07	3.15E-06	protein_coding
A_24_P329065	BTN3A1	-1.70	8.81	2.14E-07	3.16E-06	protein_coding
CUST_33865_PI428871386	ENST00000570241.2	-1.30	5.44	2.15E-07	3.17E-06	antisense
A_33_P3368388	DNAAF1	-1.27	5.05	2.15E-07	3.17E-06	protein_coding
CUST_9151_PI428871386	ENST00000476886.1	2.52	7.73	2.16E-07	3.18E-06	antisense
A_23_P356330	PPPDE2	1.53	8.22	2.16E-07	3.18E-06	protein_coding
A_32_P75299	TOMM5	1.39	11.87	2.19E-07	3.24E-06	protein_coding
A_33_P3284951	MCM5	1.47	7.90	2.19E-07	3.24E-06	protein_coding
CUST_42030_PI428871386	ENST00000342757.2	1.81	5.78	2.20E-07	3.24E-06	lincRNA
CUST_20686_PI428871386	ENST00000423499.2	-1.33	5.05	2.21E-07	3.25E-06	lincRNA
A_23_P416965	FAM149A	-1.47	7.04	2.22E-07	3.27E-06	protein_coding
A_33_P3343785	C20orf72	1.50	9.68	2.22E-07	3.27E-06	protein_coding
CUST_373_PI428871386	ENST00000447600.1	1.64	6.21	2.23E-07	3.28E-06	antisense
A_23_P8834	EPHX2	-1.82	9.03	2.23E-07	3.28E-06	protein_coding
A_23_P98900	CCDC92	-1.14	9.00	2.23E-07	3.29E-06	protein_coding
A_24_P130041	CYP51A1	-1.46	10.35	2.24E-07	3.29E-06	protein_coding
CUST_30832_PI428871386	ENST00000556120.1	-1.24	5.56	2.24E-07	3.30E-06	lincRNA
A_23_P1912	ZP1	1.00	5.21	2.25E-07	3.30E-06	protein_coding
A_33_P3327165	CCDC18	1.28	6.68	2.25E-07	3.30E-06	protein_coding
CUST_22293_PI428871386	ENST00000423223.1	-1.11	5.48	2.25E-07	3.31E-06	antisense
CUST_6168_PI428871386	ENST00000598565.1	1.16	5.06	2.26E-07	3.31E-06	antisense
CUST_26027_PI428871386	ENST00000513358.2	-1.47	6.93	2.26E-07	3.32E-06	lincRNA
CUST_10128_PI428871386	ENST00000505448.1	-1.59	7.33	2.28E-07	3.35E-06	lincRNA
CUST_10688_PI428871386	ENST00000563602.1	2.33	5.75	2.31E-07	3.39E-06	lincRNA
A_24_P374586	PMS2	1.18	7.42	2.31E-07	3.39E-06	protein_coding
A_23_P2355	CBX5	1.56	8.55	2.32E-07	3.40E-06	protein_coding
CUST_4017_PI428871386	ENST00000438436.1	1.35	6.02	2.32E-07	3.40E-06	antisense
CUST_9965_PI428871386	ENST00000414354.1	1.64	7.80	2.32E-07	3.40E-06	antisense
CUST_31907_PI428871386	ENST00000581636.1	-1.07	5.64	2.34E-07	3.43E-06	antisense
A_23_P78134	TMEM93	1.15	11.11	2.34E-07	3.43E-06	protein_coding
CUST_34887_PI428871386	ENST00000573877.1	-1.81	5.80	2.35E-07	3.44E-06	antisense
CUST_30158_PI428871386	ENST00000555276.1	1.13	10.04	2.36E-07	3.45E-06	protein_coding
CUST_19643_PI428871386	ENST00000518749.1	-1.52	7.37	2.36E-07	3.46E-06	antisense
CUST_15122_PI428871386	ENST00000590780.1	-1.14	5.43	2.37E-07	3.47E-06	antisense
A_23_P202004	PRTFDC1	1.83	7.61	2.37E-07	3.47E-06	protein_coding
CUST_37034_PI428871386	ENST00000366365.2	1.48	6.72	2.37E-07	3.47E-06	lincRNA
A_33_P3376239	MLL5	-1.17	10.35	2.37E-07	3.47E-06	protein_coding
CUST_11985_PI428871386	ENST00000506335.1	2.78	5.44	2.38E-07	3.49E-06	antisense
A_33_P3329784	ANKLE2	1.15	7.82	2.41E-07	3.52E-06	protein_coding
A_24_P166407	HIST1H4B	1.92	12.25	2.41E-07	3.53E-06	protein_coding
A_23_P82693	PABPC1	1.34	15.31	2.42E-07	3.53E-06	protein_coding
A_23_P151307	RAPGEF3	-1.15	6.34	2.44E-07	3.56E-06	protein_coding
A_33_P3337044	SLC2A6	-1.23	11.30	2.44E-07	3.56E-06	protein_coding
CUST_42259_PI428871386	ENST00000440005.1	2.19	5.73	2.44E-07	3.57E-06	antisense
CUST_24688_PI428871386	ENST00000541615.1	1.61	9.36	2.45E-07	3.57E-06	lincRNA
A_23_P89799	ACAA2	-1.93	9.93	2.46E-07	3.58E-06	protein_coding
A_33_P3312258	TUBB	1.51	10.90	2.46E-07	3.59E-06	protein_coding
A_23_P154688	SLC4A11	2.32	7.39	2.46E-07	3.59E-06	protein_coding
A_23_P146058	ATP6V1C1	1.25	10.04	2.48E-07	3.61E-06	protein_coding
A_23_P68031	STAT4	-1.48	7.35	2.49E-07	3.64E-06	protein_coding

A_23_P218442	CEACAM6	-3.87	11.48	2.51E-07	3.66E-06	protein_coding
A_24_P101402	NOP56	1.35	11.12	2.52E-07	3.66E-06	protein_coding
CUST_20754_PI428871386	ENST00000435586.1	-1.44	7.89	2.53E-07	3.68E-06	lincRNA
CUST_34878_PI428871386	ENST00000570974.1	-0.94	6.29	2.55E-07	3.71E-06	antisense
CUST_13393_PI428871386	ENST00000506053.1	-1.76	5.82	2.56E-07	3.73E-06	antisense
CUST_18150_PI428871386	ENST00000518009.1	-0.94	5.60	2.56E-07	3.73E-06	antisense
A_23_P148990	HMCN1	-2.20	8.08	2.57E-07	3.74E-06	protein_coding
A_23_P210608	ZNF217	1.34	9.00	2.58E-07	3.75E-06	protein_coding
CUST_15124_PI428871386	ENST00000434683.1	-1.30	5.72	2.59E-07	3.77E-06	antisense
CUST_30159_PI428871386	ENST00000555276.1	1.16	10.15	2.60E-07	3.78E-06	protein_coding
A_23_P163353	PIAS1	-1.06	8.51	2.64E-07	3.83E-06	protein_coding
CUST_10066_PI428871386	ENST00000503709.1	-0.99	7.45	2.64E-07	3.84E-06	antisense
A_33_P3867584	LOC285758	-1.36	6.69	2.64E-07	3.84E-06	lincRNA
CUST_11836_PI428871386	ENST00000511703.1	-1.55	5.84	2.65E-07	3.84E-06	lincRNA
CUST_20794_PI428871386	ENST00000429818.1	-1.38	6.98	2.65E-07	3.84E-06	lincRNA
CUST_9725_PI428871386	ENST00000439074.1	-1.41	5.29	2.65E-07	3.84E-06	lincRNA
CUST_7206_PI428871386	ENST00000419922.1	-1.31	5.64	2.65E-07	3.85E-06	lincRNA
A_33_P3413987	SERPING1	-1.87	10.59	2.66E-07	3.86E-06	protein_coding
CUST_9964_PI428871386	ENST00000414354.1	1.65	7.94	2.69E-07	3.90E-06	antisense
A_24_P206328	PDE1C	-1.37	6.00	2.69E-07	3.90E-06	protein_coding
CUST_39464_PI428871386	ENST00000592680.1	1.29	5.53	2.70E-07	3.91E-06	antisense
A_23_P29953	IL15	-1.84	8.81	2.71E-07	3.92E-06	protein_coding
CUST_13742_PI428871386	ENST00000520980.1	-1.09	6.05	2.72E-07	3.94E-06	antisense
CUST_3291_PI428871386	ENST00000440276.1	2.76	10.37	2.73E-07	3.95E-06	lincRNA
A_33_P3407700	TMEM192	-0.99	6.34	2.77E-07	4.00E-06	protein_coding
A_24_P261383	TAF1D	1.29	9.63	2.77E-07	4.01E-06	protein_coding
CUST_7933_PI428871386	ENST00000414382.1	1.92	5.02	2.80E-07	4.05E-06	lincRNA
A_24_P450285	CCDC153	-1.85	7.72	2.81E-07	4.07E-06	protein_coding
A_23_P310410	CD1E	-1.31	5.32	2.83E-07	4.09E-06	protein_coding
A_23_P304237	RAPGEF1	-1.48	8.07	2.85E-07	4.12E-06	protein_coding
A_23_P18447	PPARGC1A	-2.18	6.38	2.87E-07	4.15E-06	protein_coding
A_23_P167559	RNF145	-1.66	10.17	2.88E-07	4.16E-06	protein_coding
A_32_P9842	MTMR14	-1.26	11.63	2.89E-07	4.17E-06	protein_coding
A_33_P3404899	LPHN2	-1.41	6.17	2.90E-07	4.19E-06	protein_coding
CUST_42897_PI428871386	ENST00000416406.1	-1.56	7.12	2.95E-07	4.25E-06	antisense
CUST_34671_PI428871386	ENST00000593604.1	-1.00	5.33	2.96E-07	4.27E-06	lincRNA
A_23_P159039	SCRIB	1.45	11.76	2.97E-07	4.29E-06	protein_coding
CUST_15729_PI428871386	ENST00000421378.2	-1.00	5.03	2.98E-07	4.29E-06	lincRNA
A_23_P83298	PRRX2	2.21	7.25	3.00E-07	4.32E-06	protein_coding
A_33_P3328559	TBC1D10C	-2.17	10.19	3.02E-07	4.35E-06	protein_coding
CUST_24208_PI428871386	ENST00000526061.1	1.99	5.27	3.04E-07	4.38E-06	antisense
A_33_P3407065	KIF2A	1.05	7.66	3.04E-07	4.38E-06	protein_coding
CUST_32641_PI428871386	ENST00000560800.1	-1.04	5.31	3.05E-07	4.39E-06	antisense
A_23_P144453	MRFAP1	-1.34	12.30	3.07E-07	4.42E-06	protein_coding
A_23_P206960	SEC14L1	-1.40	10.89	3.07E-07	4.42E-06	protein_coding
CUST_26869_PI428871386	ENST00000512916.2	2.38	5.78	3.07E-07	4.42E-06	antisense
A_33_P3300217	MYLK3	-1.55	5.25	3.10E-07	4.45E-06	protein_coding
A_32_P44394	AIM2	3.16	8.58	3.11E-07	4.46E-06	protein_coding
A_23_P15603	MRM1	1.37	7.39	3.13E-07	4.49E-06	protein_coding
A_33_P3330099	ARSD	-1.86	10.26	3.13E-07	4.50E-06	protein_coding
CUST_41810_PI428871386	ENST00000599421.1	1.89	7.01	3.14E-07	4.51E-06	antisense
CUST_23734_PI428871386	ENST00000524824.1	-1.31	5.49	3.16E-07	4.53E-06	lincRNA
A_23_P381368	HOXD10	2.34	5.93	3.16E-07	4.53E-06	protein_coding
CUST_9957_PI428871386	ENST00000424769.1	1.65	7.78	3.16E-07	4.54E-06	antisense
CUST_4687_PI428871386	ENST00000565283.1	-1.94	8.52	3.16E-07	4.54E-06	lincRNA
A_24_P32139	HTR3E	-0.93	4.82	3.17E-07	4.55E-06	protein_coding
A_23_P6891	EIF1B	-1.15	9.65	3.18E-07	4.55E-06	protein_coding
A_33_P3279880	CYP4Z1	-1.41	5.88	3.18E-07	4.57E-06	protein_coding
A_23_P212213	THUMPD3	1.25	8.99	3.19E-07	4.57E-06	protein_coding
A_23_P45087	ZNF107	1.33	7.14	3.22E-07	4.62E-06	protein_coding
A_24_P218805	HOXC10	1.62	5.30	3.22E-07	4.62E-06	protein_coding
CUST_39972_PI428871386	ENST00000598170.1	2.17	5.57	3.23E-07	4.63E-06	lincRNA
A_24_P191781	PARM1	-2.32	8.10	3.24E-07	4.65E-06	protein_coding

A_23_P431268	PLEKHA6	-2.15	8.03	3.26E-07	4.66E-06	protein_coding
A_24_P103264	UGT8	2.58	6.27	3.28E-07	4.70E-06	protein_coding
CUST_26799_PI428871386	ENST00000564363.1	-1.52	6.09	3.29E-07	4.70E-06	lincRNA
A_24_P291588	DVL3	1.49	8.29	3.30E-07	4.73E-06	protein_coding
A_33_P3340613	FAM95B1	-1.33	5.77	3.32E-07	4.75E-06	lincRNA
A_23_P79231	CREB1	-1.04	8.42	3.32E-07	4.75E-06	protein_coding
A_23_P154022	C2orf44	1.34	7.03	3.34E-07	4.77E-06	protein_coding
CUST_33289_PI428871386	ENST00000575792.1	1.91	6.09	3.34E-07	4.77E-06	lincRNA
A_33_P3281695	NLRP3	-1.39	6.18	3.35E-07	4.78E-06	protein_coding
A_23_P212196	OGG1	-1.18	7.83	3.38E-07	4.83E-06	protein_coding
A_24_P85181	ZFYVE20	-1.10	9.03	3.40E-07	4.85E-06	protein_coding
CUST_24070_PI428871386	ENST00000531402.1	2.40	5.62	3.40E-07	4.86E-06	lincRNA
CUST_33897_PI428871386	ENST00000569993.1	-1.95	5.91	3.41E-07	4.86E-06	lincRNA
CUST_7850_PI428871386	ENST00000593741.1	0.98	5.00	3.43E-07	4.89E-06	antisense
A_32_P32413	SETBP1	-1.96	8.43	3.43E-07	4.90E-06	protein_coding
CUST_13089_PI428871386	ENST00000507491.1	-0.90	4.53	3.43E-07	4.90E-06	lincRNA
CUST_6406_PI428871386	ENST00000448255.1	2.04	6.04	3.45E-07	4.92E-06	lincRNA
CUST_28250_PI428871386	ENST00000422148.1	0.93	4.95	3.46E-07	4.93E-06	antisense
A_33_P3369079	LOC100144603	1.28	6.41	3.46E-07	4.93E-06	antisense
CUST_18747_PI428871386	ENST00000519764.1	1.52	5.21	3.49E-07	4.97E-06	lincRNA
CUST_24945_PI428871386	ENST00000561588.1	1.91	6.49	3.53E-07	5.02E-06	lincRNA
A_23_P308097	PPP2CB	-1.37	9.85	3.54E-07	5.04E-06	protein_coding
A_24_P265856	SENP7	-1.24	9.30	3.59E-07	5.11E-06	protein_coding
CUST_5877_PI428871386	ENST00000308604.5	1.52	11.24	3.63E-07	5.16E-06	lincRNA
CUST_24680_PI428871386	ENST00000539975.1	1.74	11.93	3.63E-07	5.16E-06	lincRNA
CUST_31685_PI428871386	ENST00000561318.1	-1.07	5.16	3.66E-07	5.21E-06	lincRNA
A_33_P3412016	SEMA4B	1.83	6.73	3.66E-07	5.21E-06	protein_coding
A_23_P501933	CACNG6	-2.97	7.49	3.69E-07	5.24E-06	protein_coding
A_24_P77364	BOLA3	1.19	11.86	3.73E-07	5.30E-06	protein_coding
A_32_P453321	C1orf228	-1.31	6.89	3.73E-07	5.30E-06	protein_coding
A_23_P7212	CFI	-2.28	10.21	3.73E-07	5.30E-06	protein_coding
A_24_P226755	TOX	-1.70	6.59	3.75E-07	5.33E-06	protein_coding
A_33_P3225882	BET1L	1.29	6.83	3.76E-07	5.33E-06	protein_coding
CUST_26976_PI428871386	ENST00000556850.1	-1.25	5.98	3.77E-07	5.36E-06	protein_coding
A_24_P355944	EFNB2	-2.04	9.63	3.79E-07	5.38E-06	protein_coding
CUST_36192_PI428871386	ENST00000344686.2	-1.51	10.24	3.80E-07	5.39E-06	protein_coding
A_23_P64343	TIMM10	1.41	10.82	3.81E-07	5.41E-06	protein_coding
CUST_26647_PI428871386	ENST00000551432.1	-1.70	8.06	3.82E-07	5.42E-06	lincRNA
A_24_P396167	CTSW	-1.49	6.43	3.83E-07	5.44E-06	protein_coding
A_32_P168349	C6orf25	-1.04	5.96	3.84E-07	5.44E-06	protein_coding
A_23_P204640	NANOG	-1.38	5.99	3.84E-07	5.45E-06	protein_coding
CUST_8507_PI428871386	ENST00000476021.1	0.98	4.83	3.85E-07	5.45E-06	antisense
A_24_P386771	PPP1CC	1.22	10.12	3.86E-07	5.46E-06	protein_coding
CUST_28205_PI428871386	ENST00000503695.2	-1.03	4.80	3.88E-07	5.49E-06	lincRNA
A_33_P3312676	MYT1	-1.34	5.69	3.90E-07	5.52E-06	protein_coding
A_23_P83438	UBE2Z	1.03	10.27	3.91E-07	5.54E-06	protein_coding
CUST_24209_PI428871386	ENST00000526061.1	2.06	5.34	3.94E-07	5.58E-06	antisense
CUST_9962_PI428871386	ENST00000437064.1	1.62	7.85	3.98E-07	5.63E-06	antisense
CUST_9244_PI428871386	ENST00000474477.1	-1.45	6.94	3.99E-07	5.64E-06	lincRNA
A_23_P371729	GJA5	-1.66	7.00	4.03E-07	5.69E-06	protein_coding
CUST_2750_PI428871386	ENST00000417644.1	0.93	5.03	4.05E-07	5.73E-06	antisense
CUST_376_PI428871386	ENST00000435388.1	1.56	6.29	4.06E-07	5.74E-06	antisense
A_32_P114896	PTGES3	1.44	8.74	4.07E-07	5.74E-06	protein_coding
A_23_P144677	LNPEP	-1.24	9.26	4.09E-07	5.78E-06	protein_coding
CUST_14441_PI428871386	ENST00000439891.1	-0.99	5.18	4.12E-07	5.81E-06	lincRNA
A_32_P103837	FAM60A	1.19	9.37	4.12E-07	5.82E-06	protein_coding
A_33_P3222380	AHNAK2	2.95	8.05	4.13E-07	5.82E-06	protein_coding
CUST_5030_PI428871386	ENST00000443946.1	1.18	6.61	4.17E-07	5.87E-06	lincRNA
A_23_P3663	FAM195A	1.67	10.50	4.18E-07	5.89E-06	protein_coding
A_23_P126241	EIF4G3	1.15	9.37	4.19E-07	5.90E-06	protein_coding
A_33_P3335248	SDCCAG3	1.32	7.62	4.21E-07	5.92E-06	protein_coding
CUST_1733_PI428871386	ENST00000419428.1	-1.27	6.39	4.21E-07	5.93E-06	lincRNA
CUST_14453_PI428871386	ENST00000443546.1	1.16	5.27	4.23E-07	5.95E-06	antisense

CUST_43049_PI428871386	ENST00000393264.2	-1.11	5.29	4.24E-07	5.97E-06	lincRNA
A_23_P160354	AKT3	-1.66	8.27	4.25E-07	5.97E-06	protein_coding
A_33_P3305885	WNK2	1.27	5.49	4.25E-07	5.97E-06	protein_coding
A_33_P3326588	TNFRSF10D	-2.11	8.69	4.27E-07	6.00E-06	protein_coding
A_33_P3298535	ROMO1	1.13	12.90	4.30E-07	6.04E-06	protein_coding
A_23_P501547	ADCY6	-1.37	7.35	4.32E-07	6.07E-06	protein_coding
A_24_P212152	MZT2B	1.11	14.43	4.34E-07	6.09E-06	protein_coding
A_23_P162746	CRYL1	-1.52	10.10	4.35E-07	6.12E-06	protein_coding
A_32_P222695	ARHGEF37	-2.06	7.82	4.37E-07	6.13E-06	protein_coding
CUST_5463_PI428871386	ENST00000414584.1	1.51	11.07	4.38E-07	6.14E-06	lincRNA
CUST_24904_PI428871386	ENST00000527514.1	-1.17	5.26	4.39E-07	6.16E-06	protein_coding
A_33_P3398065	TPD52L1	1.91	6.55	4.40E-07	6.18E-06	protein_coding
A_33_P3307795	FAM124B	-1.28	5.54	4.41E-07	6.18E-06	protein_coding
CUST_40890_PI428871386	ENST00000569087.1	-1.30	9.73	4.41E-07	6.19E-06	lincRNA
A_33_P3270485	CBX6	-1.71	11.64	4.43E-07	6.21E-06	protein_coding
A_23_P209347	ANKRD44	-1.48	7.44	4.47E-07	6.27E-06	protein_coding
A_23_P365738	ARC	-1.54	6.19	4.51E-07	6.31E-06	protein_coding
CUST_4686_PI428871386	ENST00000565283.1	-1.86	8.60	4.52E-07	6.33E-06	lincRNA
CUST_5454_PI428871386	ENST00000331944.6	1.50	10.78	4.57E-07	6.39E-06	lincRNA
CUST_582_PI428871386	ENST00000427796.1	-1.30	7.25	4.59E-07	6.42E-06	antisense
CUST_9462_PI428871386	ENST00000432385.1	1.31	5.03	4.60E-07	6.43E-06	antisense
A_24_P392022	FAM86A	1.21	7.19	4.60E-07	6.43E-06	protein_coding
A_23_P80321	POLR2F	1.10	12.16	4.62E-07	6.46E-06	protein_coding
CUST_39465_PI428871386	ENST00000592680.1	1.26	5.40	4.62E-07	6.47E-06	antisense
A_33_P3396224	C12orf69	-1.05	4.91	4.63E-07	6.47E-06	protein_coding
CUST_34918_PI428871386	ENST00000574016.1	-1.86	7.13	4.69E-07	6.56E-06	lincRNA
A_23_P31721	E2F5	1.57	6.69	4.71E-07	6.58E-06	protein_coding
A_33_P3297921	MRPL37	1.33	9.22	4.71E-07	6.58E-06	protein_coding
A_23_P128993	GZMH	-2.27	9.68	4.75E-07	6.63E-06	protein_coding
A_23_P86283	LAPTM5	-2.41	10.80	4.75E-07	6.63E-06	protein_coding
A_32_P515088	KDSR	-1.21	7.85	4.75E-07	6.63E-06	protein_coding
A_33_P3554318	TRIM3	-1.12	6.17	4.79E-07	6.68E-06	protein_coding
CUST_18809_PI428871386	ENST00000521802.1	1.63	5.44	4.80E-07	6.69E-06	antisense
CUST_23737_PI428871386	ENST00000531076.1	-1.30	5.40	4.80E-07	6.70E-06	lincRNA
CUST_32325_PI428871386	ENST00000560097.1	2.63	6.11	4.85E-07	6.75E-06	lincRNA
A_23_P44112	LAT	-1.38	9.52	4.86E-07	6.77E-06	protein_coding
A_23_P312132	ITGAX	-2.22	8.88	4.86E-07	6.77E-06	protein_coding
A_33_P3240353	SLC39A4	1.75	10.87	4.86E-07	6.77E-06	protein_coding
CUST_16774_PI428871386	ENST00000432866.2	-1.11	5.34	4.87E-07	6.78E-06	lincRNA
CUST_7345_PI428871386	ENST00000432481.2	1.77	5.16	4.94E-07	6.88E-06	lincRNA
A_32_P34552	POLB	1.45	6.86	4.96E-07	6.90E-06	protein_coding
CUST_2629_PI428871386	ENST00000431862.1	-1.02	4.75	4.99E-07	6.94E-06	antisense
CUST_13385_PI428871386	ENST00000512105.1	-1.40	5.43	5.05E-07	7.02E-06	lincRNA
A_23_P156310	SKP2	1.66	6.44	5.08E-07	7.06E-06	protein_coding
CUST_41602_PI428871386	ENST00000447405.1	-1.16	6.91	5.11E-07	7.11E-06	lincRNA
CUST_15645_PI428871386	ENST00000419061.1	-1.23	5.22	5.16E-07	7.18E-06	lincRNA
A_23_P384329	DENND4A	-1.30	6.19	5.17E-07	7.18E-06	protein_coding
CUST_37090_PI428871386	ENST00000590023.1	2.06	6.22	5.20E-07	7.22E-06	lincRNA
A_24_P673786	PIP4K2A	-1.42	9.36	5.23E-07	7.26E-06	protein_coding
CUST_20900_PI428871386	ENST00000429567.1	2.08	5.42	5.25E-07	7.29E-06	antisense
A_24_P588897	SLCO3A1	-1.49	8.10	5.28E-07	7.33E-06	protein_coding
A_32_P211045	DHFR	1.66	7.77	5.32E-07	7.38E-06	protein_coding
A_33_P3394489	LOC100144603	1.14	6.59	5.32E-07	7.38E-06	antisense
A_23_P19691	HEBP2	1.39	12.38	5.33E-07	7.39E-06	protein_coding
A_24_P280868	FAM86B2	1.29	8.48	5.36E-07	7.43E-06	protein_coding
CUST_7118_PI428871386	ENST00000430494.1	2.22	5.80	5.39E-07	7.46E-06	lincRNA
A_23_P132845	CLCN2	1.50	6.08	5.39E-07	7.46E-06	protein_coding
A_23_P97860	LIPA	-2.71	12.45	5.40E-07	7.48E-06	protein_coding
A_23_P93032	ZBED3	-1.63	10.47	5.46E-07	7.55E-06	protein_coding
CUST_29969_PI428871386	ENST00000553657.1	-1.27	9.42	5.46E-07	7.55E-06	antisense
A_33_P3262635	CECR1	-2.10	10.51	5.46E-07	7.55E-06	protein_coding
A_33_P3236798	SRMS	-1.69	6.90	5.46E-07	7.55E-06	protein_coding
A_33_P3233550	RTN4	-1.57	14.40	5.49E-07	7.60E-06	protein_coding

CUST_6245_PI428871386	ENST00000422799.1	-1.05	4.95	5.50E-07	7.60E-06	antisense
CUST_37093_PI428871386	ENST00000591384.1	2.20	5.93	5.52E-07	7.63E-06	lincRNA
A_24_P228302	CEACAM7	-3.17	9.89	5.55E-07	7.67E-06	protein_coding
A_23_P139912	IGFBP6	-2.42	8.39	5.60E-07	7.73E-06	protein_coding
A_23_P55682	ZSCAN18	-2.07	10.70	5.61E-07	7.75E-06	protein_coding
CUST_43512_PI428871386	ENST00000413528.1	-0.92	5.27	5.64E-07	7.79E-06	antisense
A_33_P3421571	RAPH1	2.13	11.24	5.65E-07	7.80E-06	protein_coding
CUST_38782_PI428871386	ENST00000593581.1	-1.06	8.70	5.65E-07	7.80E-06	antisense
A_33_P3833211	DNA2	1.30	5.83	5.66E-07	7.81E-06	protein_coding
A_23_P64499	MOB2	-1.01	9.38	5.67E-07	7.81E-06	protein_coding
CUST_14968_PI428871386	ENST00000448327.1	2.91	5.65	5.67E-07	7.81E-06	antisense
CUST_26585_PI428871386	ENST00000457989.1	-1.16	5.20	5.67E-07	7.82E-06	lincRNA
CUST_26657_PI428871386	ENST00000547851.1	-1.83	8.39	5.70E-07	7.86E-06	lincRNA
CUST_23101_PI428871386	ENST00000454270.1	-1.49	5.29	5.71E-07	7.87E-06	antisense
A_24_P31627	KCNB1	-1.21	5.39	5.75E-07	7.92E-06	protein_coding
A_33_P3226832	F3	-2.23	10.48	5.80E-07	7.98E-06	protein_coding
A_33_P3335522	FCRL5	1.76	6.05	5.82E-07	8.01E-06	protein_coding
A_32_P174908	WAPAL	-1.07	10.63	5.85E-07	8.04E-06	protein_coding
A_33_P3423230	PCDH19	1.84	5.26	5.86E-07	8.05E-06	protein_coding
CUST_22207_PI428871386	ENST00000566763.1	-1.03	5.39	5.86E-07	8.06E-06	lincRNA
A_33_P3240053	UFSP1	1.20	6.79	5.89E-07	8.09E-06	protein_coding
CUST_33864_PI428871386	ENST00000570241.2	-1.38	5.40	5.89E-07	8.09E-06	antisense
CUST_30615_PI428871386	ENST00000495064.1	1.58	6.18	5.91E-07	8.12E-06	lincRNA
A_33_P3356216	ARCN1	1.35	9.94	5.92E-07	8.13E-06	protein_coding
A_23_P259741	SATB1	-1.63	9.33	5.95E-07	8.17E-06	protein_coding
A_33_P3399318	GNG12	-1.31	9.76	5.98E-07	8.20E-06	protein_coding
A_23_P145485	ULBP2	1.58	6.18	5.98E-07	8.20E-06	protein_coding
A_24_P940517	PCYOX1	-1.45	9.52	5.98E-07	8.20E-06	protein_coding
CUST_927_PI428871386	ENST00000418149.1	1.30	5.55	6.03E-07	8.27E-06	antisense
A_33_P3360301	USP19	-0.94	9.86	6.06E-07	8.31E-06	protein_coding
CUST_36969_PI428871386	ENST00000578226.1	1.08	5.17	6.07E-07	8.33E-06	antisense
A_23_P55421	CBX8	1.18	6.29	6.10E-07	8.35E-06	protein_coding
A_23_P233	FMO5	-1.88	6.62	6.13E-07	8.39E-06	protein_coding
A_23_P251043	SYNDIG1	2.25	6.97	6.13E-07	8.40E-06	protein_coding
A_33_P3210379	SCGB3A1	-2.99	14.24	6.14E-07	8.40E-06	protein_coding
CUST_17326_PI428871386	ENST00000458352.1	1.36	5.38	6.21E-07	8.50E-06	antisense
A_23_P85140	TCEAL2	-1.63	7.77	6.22E-07	8.51E-06	protein_coding
A_23_P128728	ARG2	2.34	6.33	6.24E-07	8.54E-06	protein_coding
CUST_37959_PI428871386	ENST00000601445.1	1.26	5.11	6.27E-07	8.58E-06	lincRNA
CUST_30613_PI428871386	ENST00000435624.1	1.62	6.51	6.28E-07	8.58E-06	lincRNA
A_24_P43810	FAM83A	2.82	5.44	6.29E-07	8.60E-06	protein_coding
A_33_P3241269	CES1	-3.32	13.54	6.34E-07	8.66E-06	protein_coding
CUST_5879_PI428871386	ENST00000442293.1	1.50	11.48	6.35E-07	8.67E-06	lincRNA
A_33_P3330353	MOSPD1	1.40	6.64	6.37E-07	8.70E-06	protein_coding
CUST_38302_PI428871386	ENST00000579651.1	-1.06	5.33	6.38E-07	8.71E-06	lincRNA
A_24_P873414	PLEKHB2	-1.30	11.31	6.41E-07	8.75E-06	protein_coding
CUST_28280_PI428871386	ENST00000445646.2	-1.14	7.36	6.41E-07	8.75E-06	lincRNA
A_33_P3501900	LOC386758	-1.26	7.42	6.48E-07	8.84E-06	lincRNA
A_33_P3359160	FAM189B	1.61	10.07	6.49E-07	8.86E-06	protein_coding
A_23_P430271	LRRC27	-1.00	4.65	6.52E-07	8.90E-06	protein_coding
A_24_P352445	MRPL42	1.14	8.72	6.53E-07	8.90E-06	protein_coding
CUST_36961_PI428871386	ENST00000585075.1	0.98	5.62	6.55E-07	8.92E-06	antisense
CUST_19709_PI428871386	ENST00000518932.1	2.27	5.65	6.55E-07	8.93E-06	antisense
CUST_7938_PI428871386	ENST00000356047.3	1.68	5.24	6.59E-07	8.98E-06	lincRNA
CUST_29968_PI428871386	ENST00000553657.1	-1.26	9.64	6.60E-07	8.99E-06	antisense
CUST_9963_PI428871386	ENST00000437064.1	1.59	7.96	6.63E-07	9.02E-06	antisense
CUST_28554_PI428871386	ENST00000585327.1	-1.08	4.93	6.63E-07	9.03E-06	lincRNA
A_23_P30474	WDR70	1.00	9.19	6.64E-07	9.03E-06	protein_coding
CUST_6576_PI428871386	ENST00000416080.1	1.38	6.05	6.70E-07	9.11E-06	antisense
CUST_41405_PI428871386	ENST00000567259.1	1.48	5.64	6.70E-07	9.11E-06	lincRNA
A_33_P3214665	MAP2	-2.73	9.38	6.71E-07	9.11E-06	protein_coding
A_23_P254888	ZYX	-1.68	9.83	6.75E-07	9.17E-06	protein_coding
A_33_P3213374	CITED2	-1.25	7.09	6.79E-07	9.23E-06	protein_coding

A_33_P3244956	NRIP3	1.80	5.83	6.80E-07	9.24E-06	protein_coding
A_24_P2948	RUSC1	1.27	8.58	6.82E-07	9.25E-06	protein_coding
A_24_P319736	MEIS1	-1.66	8.35	6.82E-07	9.26E-06	protein_coding
CUST_43665_PI428871386	ENST00000454385.1	-1.92	6.42	6.83E-07	9.27E-06	antisense
CUST_27341_PI428871386	ENST00000551032.1	-0.93	5.31	6.84E-07	9.28E-06	lincRNA
A_33_P3211432	NCF1	-2.25	8.84	6.85E-07	9.30E-06	protein_coding
A_23_P302094	IMP4	1.15	9.72	6.86E-07	9.30E-06	protein_coding
A_33_P3369393	NCF1	-1.90	10.27	6.94E-07	9.41E-06	protein_coding
CUST_1337_PI428871386	ENST00000445976.1	2.54	5.43	6.94E-07	9.41E-06	lincRNA
CUST_577_PI428871386	ENST00000566551.1	1.43	6.90	6.95E-07	9.41E-06	lincRNA
CUST_35984_PI428871386	ENST00000577557.1	2.14	6.91	6.98E-07	9.45E-06	lincRNA
A_23_P163458	EHD4	-1.29	9.44	6.98E-07	9.46E-06	protein_coding
CUST_31229_PI428871386	ENST00000561999.1	-1.19	8.24	7.08E-07	9.58E-06	lincRNA
A_24_P336759	MCL1	-1.50	10.77	7.12E-07	9.63E-06	protein_coding
CUST_25124_PI428871386	ENST00000530759.1	1.59	7.48	7.17E-07	9.70E-06	lincRNA
CUST_1010_PI428871386	ENST00000564479.1	-1.13	5.94	7.21E-07	9.75E-06	antisense
A_23_P93302	GABBR1	-1.46	6.88	7.24E-07	9.79E-06	protein_coding
A_23_P147900	POU6F2	1.38	5.39	7.25E-07	9.80E-06	protein_coding
A_32_P4262	C20orf26	-1.94	6.33	7.25E-07	9.80E-06	protein_coding
A_23_P137848	MRPL24	1.48	10.60	7.28E-07	9.83E-06	protein_coding
A_24_P353289	C22orf13	-1.20	11.01	7.31E-07	9.88E-06	protein_coding
CUST_1020_PI428871386	ENST00000588291.1	-1.02	6.53	7.33E-07	9.89E-06	antisense
CUST_36566_PI428871386	ENST00000580022.1	-1.34	6.91	7.34E-07	9.91E-06	antisense
CUST_29411_PI428871386	ENST00000555688.1	-1.09	5.75	7.35E-07	9.92E-06	lincRNA
A_33_P3862354	TRABD	1.34	6.78	7.41E-07	9.99E-06	protein_coding
CUST_32692_PI428871386	ENST00000559321.1	3.24	6.43	7.44E-07	1.00E-05	lincRNA
CUST_43277_PI428871386	ENST00000452690.1	-0.94	4.95	7.45E-07	1.00E-05	lincRNA
CUST_30612_PI428871386	ENST00000435624.1	1.55	6.41	7.47E-07	1.01E-05	lincRNA
A_23_P216549	RUSC2	-1.16	8.23	7.47E-07	1.01E-05	protein_coding
CUST_26640_PI428871386	ENST00000550426.1	-1.68	8.23	7.47E-07	1.01E-05	lincRNA
A_23_P339119	ACSS3	-2.01	7.39	7.50E-07	1.01E-05	protein_coding
A_33_P3245289	PTPRG	-1.16	5.78	7.52E-07	1.01E-05	protein_coding
CUST_35952_PI428871386	ENST00000583195.1	-1.08	5.42	7.53E-07	1.01E-05	lincRNA
A_23_P379746	DENND5B	1.21	6.24	7.55E-07	1.02E-05	protein_coding
CUST_10814_PI428871386	ENST00000508406.1	1.88	4.89	7.56E-07	1.02E-05	lincRNA
A_23_P422193	SUV39H1	1.32	6.44	7.56E-07	1.02E-05	protein_coding
A_33_P3392892	ULK4	-1.42	6.33	7.60E-07	1.02E-05	protein_coding
A_23_P167509	CYFIP2	-1.49	7.45	7.61E-07	1.02E-05	protein_coding
CUST_374_PI428871386	ENST00000447600.1	1.67	6.43	7.64E-07	1.03E-05	antisense
A_33_P3409844	HOOK1	1.75	7.16	7.67E-07	1.03E-05	protein_coding
CUST_42257_PI428871386	ENST00000438934.1	1.42	6.05	7.73E-07	1.04E-05	antisense
CUST_41504_PI428871386	ENST00000453910.1	-1.60	6.34	7.73E-07	1.04E-05	lincRNA
CUST_36292_PI428871386	ENST00000584391.1	1.07	6.35	7.85E-07	1.06E-05	antisense
A_23_P93282	HIST1H3J	1.41	9.87	7.90E-07	1.06E-05	protein_coding
CUST_17699_PI428871386	ENST00000447307.1	-1.48	6.52	7.91E-07	1.06E-05	lincRNA
A_32_P59811	PRPF18	1.06	6.48	7.94E-07	1.07E-05	protein_coding
CUST_28494_PI428871386	ENST00000454681.1	-1.12	5.39	7.96E-07	1.07E-05	lincRNA
CUST_41037_PI428871386	ENST00000570096.1	1.62	5.65	8.00E-07	1.07E-05	antisense
CUST_9827_PI428871386	ENST00000423600.1	0.85	4.93	8.00E-07	1.07E-05	lincRNA
CUST_25254_PI428871386	ENST00000531869.1	1.21	5.71	8.05E-07	1.08E-05	antisense
CUST_5878_PI428871386	ENST00000442293.1	1.48	11.34	8.07E-07	1.08E-05	lincRNA
CUST_1336_PI428871386	ENST00000445976.1	2.53	5.41	8.08E-07	1.08E-05	lincRNA
A_33_P3369939	DNAJC21	-1.07	8.41	8.13E-07	1.09E-05	protein_coding
CUST_3313_PI428871386	ENST00000423222.1	1.70	8.16	8.13E-07	1.09E-05	lincRNA
A_23_P29257	H1F0	1.62	10.93	8.15E-07	1.09E-05	protein_coding
A_33_P3252141	TMX3	-1.43	9.48	8.18E-07	1.10E-05	protein_coding
CUST_14286_PI428871386	ENST00000506340.1	1.23	7.01	8.20E-07	1.10E-05	antisense
A_23_P128470	CLEC12A	-1.29	6.16	8.23E-07	1.10E-05	protein_coding
A_33_P3214446	RFC3	1.37	6.09	8.27E-07	1.11E-05	protein_coding
A_23_P15727	FKBP10	1.89	9.83	8.32E-07	1.11E-05	protein_coding
A_23_P146572	NPDC1	-1.79	10.88	8.33E-07	1.11E-05	protein_coding
A_33_P3410935	C17orf89	1.38	12.01	8.34E-07	1.11E-05	protein_coding
A_33_P3258617	PLIN2	2.26	6.68	8.45E-07	1.13E-05	protein_coding

CUST_10271_PI428871386	ENST00000506514.1	-1.49	5.17	8.45E-07	1.13E-05	antisense
CUST_7133_PI428871386	ENST00000343987.2	-0.99	9.64	8.47E-07	1.13E-05	lincRNA
A_33_P3368188	SEPT9_	1.51	7.58	8.56E-07	1.14E-05	protein_coding
A_33_P3214105	ATF3	-2.24	8.98	8.59E-07	1.15E-05	protein_coding
CUST_11148_PI428871386	ENST00000508111.1	-1.84	6.79	8.65E-07	1.15E-05	lincRNA
A_33_P3294053	ANKRD39	1.04	10.17	8.72E-07	1.16E-05	protein_coding
CUST_33302_PI428871386	ENST00000569858.1	-1.23	6.87	8.73E-07	1.16E-05	antisense
A_33_P3392921	CTTN	1.43	6.20	8.75E-07	1.17E-05	protein_coding
A_23_P205216	UTP14A	1.12	6.63	8.77E-07	1.17E-05	protein_coding
A_33_P3414122	ZNF260	1.50	8.87	8.78E-07	1.17E-05	protein_coding
A_23_P128698	SPRY2	-2.05	9.32	8.80E-07	1.17E-05	protein_coding
CUST_5472_PI428871386	ENST00000413202.1	1.49	10.75	8.81E-07	1.17E-05	lincRNA
A_23_P74042	LPHN2	-1.80	8.35	8.83E-07	1.18E-05	protein_coding
A_23_P70748	REPS1	1.26	10.03	8.85E-07	1.18E-05	protein_coding
CUST_43945_PI428871386	ENST00000434164.1	0.95	4.86	8.87E-07	1.18E-05	antisense
CUST_6260_PI428871386	ENST00000432608.1	-0.99	4.63	8.88E-07	1.18E-05	lincRNA
CUST_22373_PI428871386	ENST00000426283.1	1.56	8.00	8.88E-07	1.18E-05	lincRNA
A_33_P3293336	GFRA1	-1.39	6.90	8.90E-07	1.18E-05	protein_coding
A_32_P29118	SEMA3D	-2.14	7.19	8.90E-07	1.18E-05	protein_coding
A_23_P151975	RHCG	2.13	6.75	8.91E-07	1.18E-05	protein_coding
CUST_22299_PI428871386	ENST00000455774.1	-1.16	6.75	8.92E-07	1.19E-05	antisense
A_33_P3301040	HHLA2	-1.11	5.63	8.93E-07	1.19E-05	protein_coding
A_33_P3212167	ACTN2	-0.95	5.22	8.95E-07	1.19E-05	protein_coding
A_33_P3210059	SF3A1	-1.20	9.24	8.96E-07	1.19E-05	protein_coding
A_23_P310274	PRSS2	2.68	6.64	8.99E-07	1.19E-05	protein_coding
A_33_P3225066	DNAJC12	2.33	5.87	9.00E-07	1.19E-05	protein_coding
CUST_26648_PI428871386	ENST00000551898.1	-1.62	8.20	9.03E-07	1.20E-05	lincRNA
A_33_P3878772	JAK2	-1.63	7.93	9.03E-07	1.20E-05	protein_coding
CUST_41510_PI428871386	ENST00000413645.1	-1.11	5.19	9.04E-07	1.20E-05	lincRNA
CUST_42084_PI428871386	ENST00000426578.1	-1.00	4.71	9.05E-07	1.20E-05	antisense
A_33_P3242483	ARHGAP5	-1.57	8.41	9.05E-07	1.20E-05	protein_coding
CUST_3306_PI428871386	ENST00000480052.1	-1.78	8.32	9.08E-07	1.20E-05	antisense
CUST_19647_PI428871386	ENST00000499653.1	-1.27	7.07	9.10E-07	1.21E-05	antisense
CUST_5846_PI428871386	ENST00000458252.1	-1.28	5.34	9.12E-07	1.21E-05	antisense
CUST_38784_PI428871386	ENST00000597407.1	-1.09	8.53	9.16E-07	1.21E-05	antisense
A_33_P3290945	PTCD3	1.26	7.80	9.22E-07	1.22E-05	protein_coding
CUST_15127_PI428871386	ENST00000589255.1	-1.15	5.29	9.26E-07	1.23E-05	antisense
A_32_P225355	CPEB2	-1.21	5.91	9.26E-07	1.23E-05	protein_coding
A_24_P136161	HNRNPCL1	1.17	7.64	9.35E-07	1.24E-05	protein_coding
CUST_38105_PI428871386	ENST00000589662.1	1.39	5.22	9.40E-07	1.24E-05	lincRNA
A_23_P48747	DHRS1	-1.30	8.86	9.43E-07	1.25E-05	protein_coding
CUST_1474_PI428871386	ENST00000415584.2	-1.01	5.20	9.43E-07	1.25E-05	antisense
A_23_P218086	TPCN1	-1.52	10.69	9.49E-07	1.25E-05	protein_coding
CUST_19915_PI428871386	ENST00000521991.1	-0.95	4.75	9.54E-07	1.26E-05	lincRNA
CUST_11815_PI428871386	ENST00000578387.1	-1.22	6.13	9.55E-07	1.26E-05	lincRNA
CUST_3225_PI428871386	ENST00000421166.1	-1.07	5.03	9.59E-07	1.27E-05	antisense
CUST_17811_PI428871386	ENST00000467537.1	-1.58	8.48	9.62E-07	1.27E-05	antisense
A_23_P500353	KCNN2	-1.38	6.42	9.64E-07	1.27E-05	protein_coding
A_33_P3214844	NEB	1.70	5.42	9.64E-07	1.27E-05	protein_coding
CUST_37761_PI428871386	ENST00000581177.1	1.85	5.70	9.65E-07	1.27E-05	antisense
CUST_24517_PI428871386	ENST00000527239.1	-1.09	5.27	9.66E-07	1.27E-05	antisense
CUST_38797_PI428871386	ENST00000592371.1	-0.97	4.64	9.67E-07	1.27E-05	lincRNA
A_33_P3388312	SKAP2	-1.58	10.18	9.76E-07	1.29E-05	protein_coding
A_23_P88963	ALDOA	1.58	13.05	9.77E-07	1.29E-05	protein_coding
A_24_P283341	MICAL1	-1.41	8.63	9.80E-07	1.29E-05	protein_coding
A_32_P15320	EEF1A1	-1.50	13.24	9.80E-07	1.29E-05	protein_coding
A_33_P3267665	ZDHHC2	-1.89	8.90	9.84E-07	1.30E-05	protein_coding
CUST_20731_PI428871386	ENST00000591605.1	-1.23	5.24	1.01E-06	1.33E-05	lincRNA
CUST_26649_PI428871386	ENST00000551898.1	-1.72	8.29	1.01E-06	1.33E-05	lincRNA
A_23_P21838	CNP	0.99	12.82	1.03E-06	1.36E-05	protein_coding
A_33_P3412613	TMPO	1.49	7.82	1.04E-06	1.36E-05	protein_coding
CUST_1024_PI428871386	ENST00000591675.1	-1.09	6.79	1.04E-06	1.37E-05	antisense
CUST_862_PI428871386	ENST00000566366.1	1.29	5.42	1.05E-06	1.37E-05	antisense

A_23_P501754	CSF3	-3.07	7.42	1.05E-06	1.38E-05	protein_coding
A_23_P382602	BCL9	1.51	7.34	1.05E-06	1.38E-05	protein_coding
A_24_P288722	CASK	1.36	7.40	1.05E-06	1.38E-05	protein_coding
A_24_P787947	YPEL2	-1.42	7.86	1.06E-06	1.39E-05	protein_coding
A_23_P162607	STAB2	-1.61	6.40	1.06E-06	1.39E-05	protein_coding
A_23_P132793	MANF	1.34	12.13	1.06E-06	1.39E-05	protein_coding
A_24_P35891	ZNF219	-1.48	9.27	1.06E-06	1.39E-05	protein_coding
A_33_P3250887	SYCE3	1.43	5.99	1.07E-06	1.40E-05	protein_coding
A_33_P3314594	RAB37	-1.25	7.09	1.07E-06	1.40E-05	protein_coding
A_23_P406986	MOSPD2	-1.32	7.31	1.07E-06	1.41E-05	protein_coding
A_32_P159234	KIAA1456	-1.15	5.23	1.08E-06	1.41E-05	protein_coding
A_33_P3365805	CLN5	-1.36	8.44	1.09E-06	1.42E-05	protein_coding
A_33_P3348164	TTLL7	-1.28	6.28	1.09E-06	1.43E-05	protein_coding
A_23_P384532	CCDC11	-2.08	7.00	1.09E-06	1.43E-05	protein_coding
A_33_P3414574	DNAJA2	-1.40	8.16	1.09E-06	1.43E-05	protein_coding
A_33_P3387691	SCML4	-1.84	8.17	1.10E-06	1.43E-05	protein_coding
A_24_P286054	ZFYVE16	-1.09	7.97	1.10E-06	1.43E-05	protein_coding
A_24_P88850	MRAS	-1.53	9.04	1.10E-06	1.43E-05	protein_coding
A_23_P154025	SSB	1.00	11.75	1.10E-06	1.44E-05	protein_coding
A_23_P122852	SMARCD3	-1.73	10.10	1.12E-06	1.46E-05	protein_coding
CUST_4127_PI428871386	ENST00000589024.1	-0.97	4.89	1.13E-06	1.48E-05	lincRNA
A_32_P110390	TMEM171	1.43	5.85	1.14E-06	1.48E-05	protein_coding
A_24_P73389	STK24	1.14	6.85	1.15E-06	1.50E-05	protein_coding
CUST_30934_PI428871386	ENST00000551271.1	1.43	5.84	1.15E-06	1.50E-05	antisense
A_33_P3287348	CHN2	-1.68	8.03	1.15E-06	1.50E-05	protein_coding
CUST_22019_PI428871386	ENST00000561822.1	-1.18	6.06	1.15E-06	1.50E-05	lincRNA
A_24_P113131	BZRAP1	-1.52	7.58	1.15E-06	1.50E-05	protein_coding
A_24_P109633	ITPK1	-1.06	5.04	1.15E-06	1.50E-05	protein_coding
A_23_P128991	SLIRP	1.20	12.61	1.15E-06	1.50E-05	protein_coding
A_24_P62530	RHOU	-1.77	8.84	1.16E-06	1.51E-05	protein_coding
A_33_P3251073	TMEM52	2.11	8.06	1.16E-06	1.51E-05	protein_coding
CUST_27686_PI428871386	ENST00000552663.1	-1.34	6.28	1.17E-06	1.53E-05	lincRNA
CUST_24072_PI428871386	ENST00000526388.1	2.32	5.61	1.17E-06	1.53E-05	lincRNA
A_33_P3220202	PKN3	-1.23	9.44	1.18E-06	1.53E-05	protein_coding
A_24_P269814	PLEKHA1	-1.35	10.19	1.18E-06	1.54E-05	protein_coding
CUST_22823_PI428871386	ENST00000456638.1	-1.32	7.61	1.19E-06	1.54E-05	antisense
A_33_P3271445	COL4A3BP	-1.32	9.98	1.19E-06	1.54E-05	protein_coding
CUST_34795_PI428871386	ENST00000565008.1	-1.04	4.88	1.19E-06	1.55E-05	lincRNA
CUST_18915_PI428871386	ENST00000499425.1	-1.15	5.52	1.20E-06	1.55E-05	lincRNA
A_23_P420692	PPFIA4	1.46	5.51	1.20E-06	1.56E-05	protein_coding
A_33_P3406939	KIF24	1.16	6.13	1.23E-06	1.60E-05	protein_coding
A_33_P3879920	TRIM39-RPP21	1.22	11.87	1.24E-06	1.61E-05	protein_coding
A_23_P312300	SCGB2A1	-2.36	6.64	1.25E-06	1.62E-05	protein_coding
CUST_10171_PI428871386	ENST00000503938.1	1.05	5.14	1.25E-06	1.63E-05	lincRNA
A_24_P390070	SRSF9	0.93	9.87	1.25E-06	1.63E-05	protein_coding
CUST_19690_PI428871386	ENST00000562917.1	-0.89	4.55	1.26E-06	1.63E-05	lincRNA
CUST_8390_PI428871386	ENST00000597950.1	-0.97	5.21	1.27E-06	1.64E-05	antisense
CUST_2632_PI428871386	ENST00000443364.1	1.76	5.64	1.27E-06	1.65E-05	lincRNA
A_33_P3257607	PPP1R12B	-1.19	6.14	1.28E-06	1.65E-05	protein_coding
CUST_13496_PI428871386	ENST00000514573.1	-1.06	7.85	1.28E-06	1.66E-05	lincRNA
A_23_P37988	CPNE2	-1.31	8.72	1.29E-06	1.67E-05	protein_coding
CUST_14452_PI428871386	ENST00000443546.1	1.20	5.24	1.31E-06	1.69E-05	antisense
A_23_P433188	GZF1	-1.21	7.42	1.31E-06	1.70E-05	protein_coding
CUST_32375_PI428871386	ENST00000527801.1	-0.98	5.11	1.32E-06	1.71E-05	antisense
CUST_37952_PI428871386	ENST00000600644.1	2.26	5.42	1.32E-06	1.71E-05	lincRNA
A_33_P3294162	PMPCB	-1.04	6.47	1.33E-06	1.72E-05	protein_coding
CUST_16141_PI428871386	ENST00000436492.1	1.69	6.42	1.33E-06	1.72E-05	lincRNA
A_23_P96291	MAGEA1	3.74	5.64	1.35E-06	1.74E-05	protein_coding
A_33_P3256054	MLST8	1.06	9.06	1.36E-06	1.75E-05	protein_coding
A_24_P370670	ZMYM6NB	-1.47	9.63	1.36E-06	1.76E-05	protein_coding
A_23_P32454	TG	1.38	6.37	1.36E-06	1.76E-05	protein_coding
A_33_P3273230	HORMAD1	2.70	6.20	1.37E-06	1.77E-05	protein_coding
A_33_P3213557	CCZ1	1.08	7.05	1.38E-06	1.78E-05	protein_coding

CUST_16580_PI428871386	ENST00000517635.2	-0.95	4.74	1.38E-06	1.78E-05	antisense
A_23_P77440	NFATC3	-1.17	7.28	1.38E-06	1.78E-05	protein_coding
A_24_P68783	IL36RN	2.02	5.26	1.38E-06	1.78E-05	protein_coding
A_33_P3258782	AP1S2	-1.73	8.18	1.38E-06	1.79E-05	protein_coding
A_23_P81058	BMP3	-1.81	6.21	1.39E-06	1.79E-05	protein_coding
CUST_10686_PI428871386	ENST00000562355.1	2.19	5.84	1.39E-06	1.80E-05	lincRNA
CUST_7563_PI428871386	ENST00000446593.1	-1.00	4.98	1.39E-06	1.80E-05	lincRNA
CUST_26408_PI428871386	ENST00000540811.1	-1.76	6.54	1.40E-06	1.80E-05	lincRNA
A_33_P3259339	COL11A1	1.46	5.08	1.40E-06	1.81E-05	protein_coding
A_33_P3417695	ODF3B	-1.75	10.83	1.41E-06	1.81E-05	protein_coding
CUST_32640_PI428871386	ENST00000560800.1	-1.03	5.08	1.41E-06	1.81E-05	antisense
A_23_P717	TMEM206	1.34	7.34	1.43E-06	1.84E-05	protein_coding
CUST_5455_PI428871386	ENST00000331944.6	1.44	10.65	1.43E-06	1.85E-05	lincRNA
CUST_3314_PI428871386	ENST00000534914.1	1.47	7.56	1.44E-06	1.85E-05	lincRNA
CUST_3539_PI428871386	ENST00000440540.1	-2.28	6.79	1.44E-06	1.85E-05	antisense
A_23_P1072	ATP1A1	-1.32	12.82	1.45E-06	1.86E-05	protein_coding
CUST_20877_PI428871386	ENST00000590767.1	-1.16	7.88	1.45E-06	1.86E-05	antisense
CUST_33410_PI428871386	ENST00000567370.1	-1.13	5.47	1.46E-06	1.87E-05	lincRNA
A_33_P3214586	ARTN	1.44	7.12	1.46E-06	1.87E-05	protein_coding
A_33_P3307337	MAPK4	-2.07	6.40	1.46E-06	1.88E-05	protein_coding
A_33_P3312039	RAD23B	1.14	8.37	1.46E-06	1.88E-05	protein_coding
CUST_1876_PI428871386	ENST00000416193.1	1.54	7.02	1.47E-06	1.89E-05	antisense
A_32_P26401	C21orf54	0.86	5.03	1.47E-06	1.89E-05	lincRNA
A_24_P27234	SOX5	-1.43	5.94	1.48E-06	1.90E-05	protein_coding
CUST_7263_PI428871386	ENST00000441749.1	-1.22	6.75	1.49E-06	1.91E-05	antisense
A_23_P63847	SUPV3L1	1.05	7.86	1.49E-06	1.91E-05	protein_coding
A_24_P329795	C10orf10	-2.25	10.87	1.50E-06	1.92E-05	protein_coding
A_23_P49376	CETP	-1.29	5.85	1.50E-06	1.92E-05	protein_coding
A_24_P360206	PCDHA11	-1.71	7.05	1.50E-06	1.92E-05	protein_coding
A_23_P70480	HIST1H4L	1.87	12.96	1.50E-06	1.92E-05	protein_coding
CUST_18879_PI428871386	ENST00000522541.1	-1.01	5.11	1.50E-06	1.92E-05	antisense
CUST_20113_PI428871386	ENST00000521793.1	-0.91	5.26	1.51E-06	1.92E-05	lincRNA
A_33_P3230541	MPRIP	0.93	5.13	1.51E-06	1.93E-05	protein_coding
A_32_P135348	TANC1	-1.48	9.99	1.52E-06	1.94E-05	protein_coding
A_23_P301247	HIST2H2AC	1.47	11.49	1.52E-06	1.94E-05	protein_coding
A_23_P169978	ZNF608	-1.36	6.77	1.52E-06	1.95E-05	protein_coding
A_33_P3406623	TNFSF12	-1.20	6.00	1.53E-06	1.95E-05	protein_coding
A_23_P4551	SETBP1	-1.96	9.57	1.53E-06	1.96E-05	protein_coding
CUST_26641_PI428871386	ENST00000550426.1	-1.67	8.27	1.53E-06	1.96E-05	lincRNA
A_24_P237586	ANKRD37	-1.67	9.48	1.53E-06	1.96E-05	protein_coding
A_23_P153098	HDHD2	-1.28	10.62	1.54E-06	1.97E-05	protein_coding
A_33_P3803639	TRAF2	1.21	11.08	1.55E-06	1.98E-05	protein_coding
CUST_36712_PI428871386	ENST00000584959.1	-1.24	6.68	1.55E-06	1.98E-05	antisense
A_24_P364970	DHX33	1.18	6.76	1.56E-06	1.99E-05	protein_coding
A_23_P13065	ZDHHC13	1.49	7.69	1.56E-06	1.99E-05	protein_coding
A_32_P506600	RAN	1.14	13.87	1.57E-06	2.00E-05	protein_coding
A_23_P39931	DYSF	-1.59	7.16	1.57E-06	2.00E-05	protein_coding
CUST_14108_PI428871386	ENST00000522571.1	0.87	5.90	1.57E-06	2.00E-05	lincRNA
A_23_P56734	HNMT	-1.74	8.12	1.58E-06	2.01E-05	protein_coding
CUST_27170_PI428871386	ENST00000548900.1	-2.79	8.54	1.58E-06	2.01E-05	antisense
A_24_P270728	NUPR1	-1.52	13.38	1.59E-06	2.02E-05	protein_coding
A_33_P3368313	MT1H	2.12	5.92	1.59E-06	2.02E-05	protein_coding
CUST_19282_PI428871386	ENST00000518128.1	1.28	4.98	1.59E-06	2.02E-05	antisense
A_23_P215484	CCL26	1.72	5.93	1.59E-06	2.02E-05	protein_coding
A_23_P42746	NCF1	-2.20	8.59	1.59E-06	2.02E-05	protein_coding
A_24_P184555	PXN	-1.37	11.88	1.59E-06	2.03E-05	protein_coding
CUST_5869_PI428871386	ENST00000409569.2	1.43	10.78	1.60E-06	2.03E-05	lincRNA
CUST_13771_PI428871386	ENST00000505254.1	-1.07	5.49	1.60E-06	2.03E-05	lincRNA
A_23_P372771	C15orf17	-1.17	6.72	1.60E-06	2.04E-05	protein_coding
A_23_P67127	TMEM145	1.60	6.42	1.61E-06	2.05E-05	protein_coding
CUST_32097_PI428871386	ENST00000563592.1	-1.06	4.96	1.62E-06	2.05E-05	antisense
A_32_P178800	ITGA2	1.79	6.09	1.62E-06	2.06E-05	protein_coding
A_23_P345710	XXYLT1	1.26	7.56	1.62E-06	2.06E-05	protein_coding

A_24_P167825	VPS39	-1.05	7.48	1.63E-06	2.07E-05	protein_coding
CUST_38692_PI428871386	ENST00000591657.1	1.21	6.19	1.64E-06	2.08E-05	antisense
A_33_P3256272	KRTAP10-5	-0.95	5.70	1.65E-06	2.09E-05	protein_coding
A_23_P326204	SGMS2	-1.87	7.86	1.66E-06	2.10E-05	protein_coding
CUST_31255_PI428871386	ENST00000503496.1	0.91	4.83	1.66E-06	2.11E-05	antisense
CUST_13389_PI428871386	ENST00000510972.1	-1.49	5.90	1.67E-06	2.12E-05	antisense
CUST_38830_PI428871386	ENST00000344893.3	-1.13	5.27	1.67E-06	2.12E-05	lincRNA
CUST_34725_PI428871386	ENST00000378417.1	-1.04	5.22	1.68E-06	2.13E-05	lincRNA
A_24_P165450	TTLL7	-1.29	6.17	1.68E-06	2.13E-05	protein_coding
CUST_34995_PI428871386	ENST00000577176.1	-1.13	5.89	1.68E-06	2.13E-05	antisense
CUST_27550_PI428871386	ENST00000546421.1	1.06	5.90	1.68E-06	2.13E-05	antisense
A_23_P32805	GRID1	-1.24	5.85	1.69E-06	2.14E-05	protein_coding
A_23_P203376	MS4A6A	-1.85	7.07	1.70E-06	2.15E-05	protein_coding
A_33_P3253747	CYP1A2	-1.12	6.88	1.71E-06	2.16E-05	protein_coding
CUST_14566_PI428871386	ENST00000453179.1	-1.14	5.24	1.71E-06	2.16E-05	lincRNA
CUST_42720_PI428871386	ENST00000442126.1	-1.19	5.25	1.73E-06	2.19E-05	antisense
CUST_29963_PI428871386	ENST00000556225.1	-1.14	7.27	1.74E-06	2.20E-05	antisense
A_23_P375549	FUNDC1	1.16	8.70	1.74E-06	2.20E-05	protein_coding
A_24_P331779	RSPH4A	-1.42	5.76	1.74E-06	2.20E-05	protein_coding
A_24_P115651	ENKUR	-1.97	6.15	1.75E-06	2.21E-05	protein_coding
CUST_26963_PI428871386	ENST00000548731.1	-0.95	4.90	1.75E-06	2.21E-05	antisense
CUST_13314_PI428871386	ENST00000515570.1	-0.87	5.18	1.75E-06	2.22E-05	antisense
A_23_P252928	MAGEA12	2.67	5.95	1.76E-06	2.22E-05	protein_coding
A_24_P409346	NPEPPS	1.07	8.04	1.76E-06	2.22E-05	protein_coding
A_33_P3336282	TAF4B	1.13	5.43	1.77E-06	2.23E-05	protein_coding
A_23_P43107	TM7SF4	-2.40	7.16	1.77E-06	2.24E-05	protein_coding
A_23_P153197	TGIF1	1.25	8.85	1.77E-06	2.24E-05	protein_coding
A_33_P3236906	LINC00271	-0.88	5.02	1.77E-06	2.24E-05	lincRNA
CUST_28558_PI428871386	ENST00000587571.1	-1.03	4.96	1.78E-06	2.24E-05	lincRNA
CUST_36588_PI428871386	ENST00000585190.1	-1.54	7.79	1.78E-06	2.24E-05	antisense
CUST_37092_PI428871386	ENST00000591384.1	2.27	6.24	1.79E-06	2.25E-05	lincRNA
CUST_42891_PI428871386	ENST00000412067.1	0.94	5.44	1.79E-06	2.26E-05	antisense
CUST_31882_PI428871386	ENST00000560622.1	1.10	5.74	1.79E-06	2.26E-05	antisense
A_33_P3292179	ABCA9	-1.52	13.93	1.79E-06	2.26E-05	protein_coding
A_23_P371824	TUFT1	1.86	10.75	1.80E-06	2.27E-05	protein_coding
A_23_P5757	TPRKB	1.24	9.45	1.82E-06	2.30E-05	protein_coding
A_33_P3209950	RASGRP2	-1.08	6.87	1.83E-06	2.30E-05	protein_coding
CUST_8267_PI428871386	ENST00000470427.1	-1.05	4.97	1.84E-06	2.31E-05	antisense
A_33_P3333587	FBXL20	-1.35	10.21	1.84E-06	2.31E-05	protein_coding
A_23_P34968	SCNM1	1.19	11.23	1.86E-06	2.34E-05	protein_coding
A_23_P28105	TSN	1.18	8.33	1.86E-06	2.34E-05	protein_coding
A_23_P211584	C22orf32	-0.98	5.49	1.87E-06	2.35E-05	protein_coding
CUST_3305_PI428871386	ENST00000437764.1	-1.81	8.73	1.87E-06	2.36E-05	antisense
A_33_P3821660	C9orf86	1.19	6.70	1.88E-06	2.36E-05	protein_coding
CUST_39064_PI428871386	ENST00000594678.1	-0.96	4.78	1.88E-06	2.36E-05	antisense
CUST_27537_PI428871386	ENST00000541282.1	-1.02	4.74	1.89E-06	2.38E-05	lincRNA
A_23_P66694	EVI2B	-1.92	8.95	1.90E-06	2.39E-05	protein_coding
CUST_21395_PI428871386	ENST00000427548.1	1.68	6.13	1.91E-06	2.40E-05	lincRNA
CUST_20671_PI428871386	ENST00000452923.1	1.00	6.13	1.93E-06	2.42E-05	lincRNA
A_23_P144697	RAD1	1.13	8.25	1.94E-06	2.43E-05	protein_coding
A_23_P162879	APOPT1	1.26	9.86	1.95E-06	2.45E-05	protein_coding
CUST_24062_PI428871386	ENST00000504230.2	2.52	6.63	1.95E-06	2.45E-05	lincRNA
CUST_27332_PI428871386	ENST00000549527.1	-0.95	4.77	1.96E-06	2.46E-05	lincRNA
CUST_32322_PI428871386	ENST00000560054.1	2.63	6.15	1.96E-06	2.46E-05	lincRNA
A_32_P208403	GNG2	-1.51	7.16	1.97E-06	2.47E-05	protein_coding
CUST_3811_PI428871386	ENST00000443763.1	0.94	4.88	1.97E-06	2.48E-05	antisense
CUST_42274_PI428871386	ENST00000565162.1	1.20	5.91	1.98E-06	2.48E-05	antisense
CUST_41874_PI428871386	ENST00000454980.1	1.38	5.80	1.98E-06	2.48E-05	antisense
CUST_13503_PI428871386	ENST00000501702.2	-1.18	7.83	1.99E-06	2.49E-05	lincRNA
A_33_P3374085	LRRFIP1	-1.13	8.59	1.99E-06	2.50E-05	protein_coding
A_23_P254978	TATDN1	1.47	7.84	2.00E-06	2.50E-05	protein_coding
A_23_P83184	LCN6	-1.24	5.30	2.00E-06	2.50E-05	protein_coding
A_24_P411121	TNFRSF18	1.64	6.26	2.00E-06	2.51E-05	protein_coding

CUST_17084_PI428871386	ENST00000567919.1	0.97	5.74	2.01E-06	2.51E-05	lincRNA
A_33_P3284077	NUP133	1.12	6.61	2.02E-06	2.53E-05	protein_coding
CUST_14114_PI428871386	ENST00000517582.1	0.89	5.01	2.02E-06	2.53E-05	antisense
A_23_P77908	SLC47A2	1.82	5.30	2.03E-06	2.54E-05	protein_coding
CUST_30830_PI428871386	ENST00000557109.1	-1.07	5.64	2.04E-06	2.55E-05	lincRNA
CUST_37760_PI428871386	ENST00000581177.1	1.77	5.75	2.05E-06	2.56E-05	antisense
CUST_23613_PI428871386	ENST00000456514.1	0.88	4.98	2.05E-06	2.56E-05	lincRNA
CUST_31906_PI428871386	ENST00000581636.1	-1.07	5.62	2.05E-06	2.56E-05	antisense
CUST_25280_PI428871386	ENST00000528660.1	-1.01	5.42	2.06E-06	2.57E-05	antisense
A_33_P3399248	UFM1	1.14	10.49	2.06E-06	2.57E-05	protein_coding
A_23_P44295	CLASP2	-1.04	9.71	2.07E-06	2.59E-05	protein_coding
CUST_16582_PI428871386	ENST00000518046.1	-1.21	5.94	2.08E-06	2.59E-05	antisense
A_23_P374782	SH3KBP1	-1.51	9.84	2.08E-06	2.60E-05	protein_coding
CUST_27539_PI428871386	ENST00000548886.1	-1.11	5.34	2.08E-06	2.60E-05	lincRNA
A_32_P133072	SPON1	-2.20	9.70	2.10E-06	2.62E-05	protein_coding
CUST_20012_PI428871386	ENST00000522414.1	1.40	6.32	2.11E-06	2.63E-05	lincRNA
A_23_P86599	DMBT1	-3.97	12.52	2.12E-06	2.64E-05	protein_coding
A_23_P138655	CYP26A1	2.63	5.75	2.12E-06	2.64E-05	protein_coding
A_23_P134347	CPVL	-2.22	8.96	2.14E-06	2.66E-05	protein_coding
A_23_P502470	IL6ST	-1.40	8.61	2.14E-06	2.66E-05	protein_coding
A_33_P3397865	TNNT1	3.01	9.05	2.15E-06	2.68E-05	protein_coding
CUST_43150_PI428871386	ENST00000412485.1	-2.01	6.18	2.16E-06	2.69E-05	lincRNA
A_33_P3256560	ZER1	-1.03	11.19	2.17E-06	2.70E-05	protein_coding
CUST_12634_PI428871386	ENST00000503106.1	-1.00	4.80	2.17E-06	2.70E-05	lincRNA
A_23_P376557	MMP25	-1.75	7.41	2.17E-06	2.71E-05	protein_coding
CUST_2710_PI428871386	ENST00000457106.1	1.56	6.54	2.18E-06	2.71E-05	antisense
CUST_3303_PI428871386	ENST00000475406.1	-1.74	8.44	2.19E-06	2.72E-05	antisense
A_24_P20630	LEF1	1.62	8.78	2.20E-06	2.73E-05	protein_coding
CUST_31265_PI428871386	ENST00000501169.2	-1.15	5.26	2.20E-06	2.73E-05	lincRNA
A_24_P195476	PMS2	1.03	8.50	2.21E-06	2.75E-05	protein_coding
CUST_28672_PI428871386	ENST00000433480.2	-0.97	5.03	2.21E-06	2.75E-05	lincRNA
A_23_P85893	C1orf85	1.09	9.18	2.21E-06	2.75E-05	protein_coding
A_23_P256682	APEX2	1.07	9.10	2.22E-06	2.75E-05	protein_coding
A_33_P3260989	TAS1R3	1.04	5.54	2.23E-06	2.76E-05	protein_coding
A_33_P3379377	MARS	0.89	7.12	2.23E-06	2.76E-05	protein_coding
A_23_P110445	APBB3	-1.05	8.45	2.23E-06	2.77E-05	protein_coding
A_24_P48248	C17orf53	1.30	5.24	2.24E-06	2.78E-05	protein_coding
CUST_389_PI428871386	ENST00000400892.2	-1.32	7.32	2.24E-06	2.78E-05	antisense
CUST_22017_PI428871386	ENST00000449648.1	-1.82	8.04	2.25E-06	2.79E-05	lincRNA
CUST_32121_PI428871386	ENST00000567257.1	1.45	6.46	2.25E-06	2.79E-05	antisense
A_24_P30194	IFIT5	-1.43	9.78	2.25E-06	2.79E-05	protein_coding
A_23_P129425	TSNAXIP1	-1.28	5.83	2.26E-06	2.80E-05	protein_coding
A_24_P944253	KLHL6	-1.90	7.52	2.26E-06	2.80E-05	protein_coding
CUST_37338_PI428871386	ENST00000580781.1	1.05	5.16	2.27E-06	2.81E-05	lincRNA
A_33_P3262742	DAPL1	2.64	5.99	2.27E-06	2.81E-05	protein_coding
CUST_15461_PI428871386	ENST00000525151.1	-1.03	10.45	2.28E-06	2.82E-05	antisense
A_23_P88710	TMEM87A	-1.38	12.33	2.29E-06	2.83E-05	protein_coding
CUST_26638_PI428871386	ENST00000547626.1	-1.55	8.17	2.31E-06	2.85E-05	lincRNA
CUST_3312_PI428871386	ENST00000423222.1	1.61	8.21	2.32E-06	2.87E-05	lincRNA
CUST_27171_PI428871386	ENST00000548900.1	-2.75	8.70	2.32E-06	2.87E-05	antisense
CUST_38780_PI428871386	ENST00000597785.1	-1.05	8.92	2.33E-06	2.87E-05	antisense
A_23_P207058	SOCS3	-2.32	10.89	2.33E-06	2.87E-05	protein_coding
CUST_25142_PI428871386	ENST00000533437.1	0.94	5.55	2.33E-06	2.88E-05	antisense
CUST_5870_PI428871386	ENST00000419736.1	1.45	10.91	2.33E-06	2.89E-05	lincRNA
A_23_P156471	CDC5L	1.24	9.82	2.34E-06	2.90E-05	protein_coding
CUST_20439_PI428871386	ENST00000430766.1	-1.07	5.19	2.35E-06	2.91E-05	lincRNA
A_23_P56590	C1D	1.12	8.65	2.36E-06	2.92E-05	protein_coding
A_23_P91764	TNFRSF13C	1.36	5.91	2.36E-06	2.92E-05	protein_coding
CUST_18924_PI428871386	ENST00000524338.1	-1.04	5.12	2.37E-06	2.92E-05	lincRNA
CUST_34886_PI428871386	ENST00000573877.1	-1.58	5.68	2.38E-06	2.94E-05	antisense
A_23_P77401	CPPED1	-1.64	7.97	2.39E-06	2.95E-05	protein_coding
A_33_P3374878	FAT4	-1.52	7.09	2.39E-06	2.95E-05	protein_coding
A_33_P3240843	TMEM71	-1.47	6.29	2.40E-06	2.96E-05	protein_coding

CUST_26724_PI428871386	ENST00000552893.1	1.21	7.01	2.42E-06	2.99E-05	antisense
CUST_13188_PI428871386	ENST00000505527.1	1.63	5.02	2.43E-06	2.99E-05	lincRNA
A_23_P47709	FOLR2	-1.88	9.58	2.43E-06	3.00E-05	protein_coding
A_23_P340333	ITPRIP	-1.48	10.81	2.44E-06	3.01E-05	protein_coding
A_33_P3353345	SULT4A1	2.40	6.10	2.45E-06	3.01E-05	protein_coding
A_23_P150189	MRE11A	1.13	8.95	2.45E-06	3.02E-05	protein_coding
A_23_P151166	HVCN1	-1.66	7.90	2.45E-06	3.02E-05	protein_coding
CUST_11124_PI428871386	ENST00000518701.1	0.87	5.02	2.46E-06	3.03E-05	lincRNA
A_23_P12680	PSAP	-1.93	12.72	2.48E-06	3.06E-05	protein_coding
A_33_P3287770	TELO2	1.02	10.66	2.50E-06	3.07E-05	protein_coding
A_33_P3211793	ENST00000538304	-1.16	6.75	2.50E-06	3.07E-05	lincRNA
A_23_P422083	TMEM55A	-1.39	8.18	2.51E-06	3.09E-05	protein_coding
A_23_P31844	ATP6V1B2	-1.68	9.40	2.51E-06	3.09E-05	protein_coding
A_33_P3674851	VPS36	-1.33	9.32	2.52E-06	3.10E-05	protein_coding
A_23_P65262	N4BP2L2	-1.44	7.86	2.53E-06	3.11E-05	protein_coding
CUST_2768_PI428871386	ENST00000422548.1	1.17	4.93	2.53E-06	3.11E-05	lincRNA
CUST_31516_PI428871386	ENST00000560750.1	-1.04	6.44	2.56E-06	3.14E-05	lincRNA
CUST_9825_PI428871386	ENST00000415451.1	-1.00	5.66	2.56E-06	3.15E-05	antisense
A_23_P89812	CNDP2	-1.23	10.27	2.56E-06	3.15E-05	protein_coding
A_24_P58337	FTH1	-1.62	15.48	2.59E-06	3.18E-05	protein_coding
A_24_P405430	TIA1	-1.24	8.41	2.60E-06	3.19E-05	protein_coding
A_33_P3278877	ROBO2	-1.27	5.67	2.61E-06	3.20E-05	protein_coding
A_24_P235049	MTHFD1L	1.18	7.65	2.62E-06	3.22E-05	protein_coding
A_23_P301855	LSAMP	-1.59	7.11	2.63E-06	3.23E-05	protein_coding
A_33_P3358208	PADI1	1.72	5.29	2.65E-06	3.24E-05	protein_coding
A_24_P30206	BCCIP	1.14	7.77	2.65E-06	3.24E-05	protein_coding
A_23_P336513	GEMIN5	1.15	8.87	2.65E-06	3.25E-05	protein_coding
CUST_4946_PI428871386	ENST00000431130.2	-0.84	4.91	2.65E-06	3.25E-05	antisense
CUST_30614_PI428871386	ENST00000495064.1	1.50	6.25	2.66E-06	3.26E-05	lincRNA
CUST_22673_PI428871386	ENST00000425290.1	-0.89	4.94	2.68E-06	3.28E-05	antisense
CUST_35982_PI428871386	ENST00000578280.1	2.07	6.96	2.69E-06	3.29E-05	lincRNA
A_33_P3251796	PDSS1	1.33	6.97	2.69E-06	3.29E-05	protein_coding
A_23_P346884	RBPJL	-1.27	5.64	2.70E-06	3.30E-05	protein_coding
A_24_P865226	LOC440356	1.36	5.35	2.70E-06	3.30E-05	antisense
CUST_42268_PI428871386	ENST00000424407.1	-0.94	5.04	2.71E-06	3.31E-05	antisense
A_33_P3255544	RNF130	-1.44	10.23	2.72E-06	3.33E-05	protein_coding
A_23_P37191	PSMB5	1.16	11.89	2.73E-06	3.34E-05	protein_coding
CUST_22298_PI428871386	ENST00000455774.1	-1.14	6.54	2.75E-06	3.36E-05	antisense
CUST_39252_PI428871386	ENST00000593324.1	-0.98	5.09	2.77E-06	3.38E-05	lincRNA
CUST_1775_PI428871386	ENST00000554749.1	-1.22	7.83	2.78E-06	3.40E-05	antisense
A_23_P14769	FES	-1.70	8.16	2.79E-06	3.40E-05	protein_coding
A_33_P3221313	CENPI	1.23	5.38	2.80E-06	3.41E-05	protein_coding
CUST_22792_PI428871386	ENST00000600206.1	-0.95	5.57	2.82E-06	3.44E-05	antisense
A_33_P3251144	CDCA7L	1.24	5.87	2.83E-06	3.45E-05	protein_coding
A_33_P3418294	DNAH14	1.49	5.68	2.86E-06	3.48E-05	protein_coding
CUST_22598_PI428871386	ENST00000561565.1	0.87	4.87	2.87E-06	3.50E-05	lincRNA
CUST_15644_PI428871386	ENST00000430296.1	-1.16	5.18	2.87E-06	3.50E-05	lincRNA
A_23_P55632	SERPINB3	3.07	6.79	2.88E-06	3.51E-05	protein_coding
A_23_P40295	C20orf103	2.02	7.94	2.88E-06	3.52E-05	protein_coding
CUST_26725_PI428871386	ENST00000552893.1	1.17	6.94	2.89E-06	3.52E-05	antisense
CUST_30935_PI428871386	ENST00000551271.1	1.29	5.80	2.91E-06	3.54E-05	antisense
A_23_P63032	GUCA2B	-1.38	6.56	2.92E-06	3.56E-05	protein_coding
A_23_P22134	BNC1	2.26	5.41	2.92E-06	3.56E-05	protein_coding
CUST_5452_PI428871386	ENST00000409054.1	1.51	9.39	2.93E-06	3.56E-05	lincRNA
A_33_P3289356	CD58	-1.51	8.91	2.93E-06	3.56E-05	protein_coding
CUST_26295_PI428871386	ENST00000545914.1	1.45	6.44	2.94E-06	3.57E-05	antisense
A_23_P142634	METTL5	1.13	10.56	2.94E-06	3.58E-05	protein_coding
A_33_P3398091	ZNF562	1.11	8.28	2.96E-06	3.60E-05	protein_coding
A_23_P331598	IPO7	1.23	8.96	2.96E-06	3.60E-05	protein_coding
CUST_11012_PI428871386	ENST00000436413.1	1.07	5.45	2.96E-06	3.60E-05	antisense
A_23_P352535	PPP1R16B	-1.30	9.82	2.98E-06	3.62E-05	protein_coding
CUST_19644_PI428871386	ENST00000518749.1	-1.30	7.44	2.98E-06	3.62E-05	antisense
CUST_14744_PI428871386	ENST00000429600.1	-1.39	7.68	2.98E-06	3.62E-05	lincRNA

CUST_7204_PI428871386	ENST00000423530.1	-1.09	5.58	2.99E-06	3.63E-05	lincRNA
A_23_P92517	TTC29	-1.95	5.99	3.07E-06	3.73E-05	protein_coding
A_33_P3228072	NPHP3	-1.10	8.31	3.08E-06	3.74E-05	protein_coding
CUST_34794_PI428871386	ENST00000565008.1	-0.97	4.89	3.09E-06	3.75E-05	lincRNA
A_23_P148546	NAA10	1.24	9.15	3.10E-06	3.76E-05	protein_coding
CUST_6661_PI428871386	ENST00000295549.3	2.56	6.78	3.10E-06	3.76E-05	lincRNA
CUST_31759_PI428871386	ENST00000567865.1	-0.99	5.26	3.12E-06	3.78E-05	lincRNA
CUST_18812_PI428871386	ENST00000520890.1	1.58	5.48	3.12E-06	3.78E-05	antisense
CUST_15475_PI428871386	ENST00000440001.2	-0.99	10.53	3.12E-06	3.78E-05	antisense
CUST_24906_PI428871386	ENST00000532296.1	1.13	5.46	3.13E-06	3.79E-05	antisense
CUST_38781_PI428871386	ENST00000597785.1	-1.04	8.73	3.14E-06	3.80E-05	antisense
CUST_32600_PI428871386	ENST00000556030.1	-1.30	5.49	3.14E-06	3.80E-05	lincRNA
CUST_24069_PI428871386	ENST00000529328.1	2.23	5.51	3.15E-06	3.81E-05	lincRNA
CUST_24761_PI428871386	ENST00000457725.1	-1.03	5.32	3.15E-06	3.81E-05	lincRNA
CUST_43648_PI428871386	ENST00000453528.1	-1.08	4.96	3.15E-06	3.82E-05	lincRNA
A_23_P355824	MGRN1	-1.30	11.55	3.16E-06	3.82E-05	protein_coding
A_23_P212423	XYLB	0.97	5.32	3.16E-06	3.82E-05	protein_coding
A_24_P390495	CX3CL1	-1.90	7.42	3.16E-06	3.83E-05	protein_coding
CUST_22856_PI428871386	ENST00000416398.1	-1.54	6.37	3.17E-06	3.83E-05	lincRNA
CUST_13251_PI428871386	ENST00000504081.1	2.32	5.38	3.17E-06	3.84E-05	antisense
CUST_15519_PI428871386	ENST00000427157.1	1.23	5.16	3.18E-06	3.84E-05	lincRNA
A_23_P363399	SLC38A1	1.58	10.48	3.18E-06	3.85E-05	protein_coding
A_24_P940921	SETX	-1.12	9.19	3.19E-06	3.86E-05	protein_coding
A_33_P3242174	C2orf82	1.36	6.66	3.20E-06	3.87E-05	protein_coding
A_23_P26674	KIAA0430	-1.13	10.66	3.21E-06	3.87E-05	protein_coding
A_23_P58647	CTNNA1	-1.13	11.03	3.22E-06	3.88E-05	protein_coding
CUST_31188_PI428871386	ENST00000557989.1	-1.00	5.71	3.24E-06	3.91E-05	antisense
A_33_P3364811	PTPRC	-2.02	9.49	3.27E-06	3.94E-05	protein_coding
A_24_P144439	EFHB	-1.09	5.36	3.28E-06	3.95E-05	protein_coding
A_24_P227141	ELF5	-2.12	6.77	3.28E-06	3.96E-05	protein_coding
A_24_P603224	SF3A3	1.16	10.23	3.31E-06	3.99E-05	protein_coding
CUST_26639_PI428871386	ENST00000547626.1	-1.59	8.14	3.31E-06	3.99E-05	lincRNA
CUST_375_PI428871386	ENST00000435388.1	1.52	6.31	3.32E-06	4.00E-05	antisense
CUST_15009_PI428871386	ENST00000592500.1	0.94	4.86	3.36E-06	4.05E-05	antisense
A_24_P183094	ATP13A3	1.13	10.37	3.39E-06	4.09E-05	protein_coding
A_23_P216225	EGR3	-1.86	6.64	3.41E-06	4.10E-05	protein_coding
CUST_30839_PI428871386	ENST00000554441.1	-0.95	5.64	3.42E-06	4.12E-05	lincRNA
A_24_P349151	SASS6	1.16	5.56	3.43E-06	4.13E-05	protein_coding
CUST_34917_PI428871386	ENST00000573075.1	-1.72	7.02	3.44E-06	4.14E-05	lincRNA
CUST_41038_PI428871386	ENST00000570096.1	1.63	5.75	3.45E-06	4.15E-05	antisense
CUST_40453_PI428871386	ENST00000594816.1	-1.29	6.21	3.46E-06	4.16E-05	lincRNA
CUST_10825_PI428871386	ENST00000318186.3	-1.05	6.07	3.46E-06	4.16E-05	antisense
CUST_7241_PI428871386	ENST00000447289.1	0.87	6.62	3.46E-06	4.16E-05	antisense
CUST_12991_PI428871386	ENST00000509783.1	0.87	5.29	3.49E-06	4.19E-05	lincRNA
CUST_3457_PI428871386	ENST00000434398.1	-0.85	4.85	3.49E-06	4.19E-05	lincRNA
CUST_34660_PI428871386	ENST00000600553.1	-1.13	5.63	3.49E-06	4.20E-05	lincRNA
CUST_39897_PI428871386	ENST00000590369.1	1.60	5.15	3.52E-06	4.22E-05	antisense
CUST_9674_PI428871386	ENST00000446091.1	1.20	7.07	3.52E-06	4.22E-05	lincRNA
CUST_20460_PI428871386	ENST00000442260.1	0.88	4.91	3.53E-06	4.24E-05	lincRNA
CUST_11984_PI428871386	ENST00000506335.1	2.63	5.31	3.53E-06	4.24E-05	antisense
CUST_5136_PI428871386	ENST00000433396.1	-1.03	5.26	3.55E-06	4.26E-05	lincRNA
CUST_11151_PI428871386	ENST00000511919.1	-1.62	6.76	3.56E-06	4.27E-05	lincRNA
CUST_3307_PI428871386	ENST00000480052.1	-1.70	8.29	3.56E-06	4.27E-05	antisense
A_24_P296508	SLC43A2	-1.46	8.85	3.56E-06	4.27E-05	protein_coding
A_23_P435941	SAMD1	1.23	7.49	3.60E-06	4.31E-05	protein_coding
CUST_3606_PI428871386	ENST00000425412.1	1.17	5.47	3.63E-06	4.35E-05	antisense
A_23_P84872	SECISBP2	-1.06	7.50	3.65E-06	4.37E-05	protein_coding
A_23_P52082	INTS7	1.19	7.36	3.66E-06	4.39E-05	protein_coding
A_33_P3357843	SGCB	-1.47	8.61	3.66E-06	4.39E-05	protein_coding
CUST_24668_PI428871386	ENST00000539921.1	1.59	11.39	3.67E-06	4.40E-05	lincRNA
A_24_P365721	SLC6A14	-2.98	8.23	3.68E-06	4.40E-05	protein_coding
A_23_P133359	ZFP2	-1.06	6.07	3.70E-06	4.43E-05	protein_coding
A_33_P3222648	STK4	-0.94	9.71	3.71E-06	4.44E-05	protein_coding

A_23_P148609	PLAC1	2.06	5.46	3.72E-06	4.45E-05	protein_coding
CUST_32323_PI428871386	ENST00000560054.1	2.61	6.18	3.73E-06	4.46E-05	lincRNA
A_23_P136573	ST3GAL5	-1.85	9.44	3.73E-06	4.46E-05	protein_coding
CUST_36416_PI428871386	ENST00000499842.1	-1.50	5.88	3.74E-06	4.47E-05	lincRNA
A_24_P706752	PLA2G12A	-1.17	7.76	3.74E-06	4.48E-05	protein_coding
A_23_P426944	PAX9	1.64	6.34	3.75E-06	4.49E-05	protein_coding
CUST_1959_PI428871386	ENST00000457043.1	-1.15	6.80	3.76E-06	4.49E-05	antisense
A_24_P99795	ISOC2	1.14	8.84	3.76E-06	4.49E-05	protein_coding
A_23_P32615	MNAT1	1.29	9.03	3.77E-06	4.50E-05	protein_coding
A_24_P237443	SASH3	-1.90	8.66	3.80E-06	4.54E-05	protein_coding
CUST_29962_PI428871386	ENST00000556225.1	-1.19	7.42	3.83E-06	4.57E-05	antisense
CUST_16163_PI428871386	ENST00000433388.1	-1.08	5.63	3.84E-06	4.58E-05	lincRNA
A_23_P46812	CPEB3	-0.98	6.10	3.86E-06	4.60E-05	protein_coding
CUST_28204_PI428871386	ENST00000503695.2	-1.00	4.74	3.87E-06	4.61E-05	lincRNA
A_33_P3210363	LOC100128191	1.16	5.48	3.88E-06	4.63E-05	antisense
A_33_P3222942	GPR78	-0.97	4.76	3.90E-06	4.64E-05	protein_coding
A_33_P3386547	SGPP2	2.00	7.60	3.90E-06	4.65E-05	protein_coding
A_32_P101689	FAM3C	1.50	8.81	3.92E-06	4.67E-05	protein_coding
A_33_P3232955	F2RL3	-1.47	8.85	3.93E-06	4.68E-05	protein_coding
A_33_P3241646	PANK3	1.11	7.23	3.94E-06	4.69E-05	protein_coding
A_23_P26037	FRMD5	1.63	5.34	3.94E-06	4.69E-05	protein_coding
A_24_P128442	TBX15	1.96	5.84	3.94E-06	4.69E-05	protein_coding
CUST_2654_PI428871386	ENST00000427339.1	-0.98	5.11	3.95E-06	4.70E-05	lincRNA
CUST_30527_PI428871386	ENST00000554522.1	-0.97	4.85	3.96E-06	4.71E-05	lincRNA
A_23_P28590	FASTKD1	1.06	8.76	3.96E-06	4.71E-05	protein_coding
CUST_36562_PI428871386	ENST00000578025.1	-1.23	6.77	4.00E-06	4.76E-05	antisense
CUST_34670_PI428871386	ENST00000593604.1	-0.96	5.11	4.02E-06	4.78E-05	lincRNA
A_33_P3251148	TSPO	-1.47	14.20	4.02E-06	4.78E-05	protein_coding
CUST_34594_PI428871386	ENST00000566773.1	1.36	7.97	4.04E-06	4.81E-05	antisense
A_33_P3288569	MBIP	-1.86	9.94	4.09E-06	4.87E-05	protein_coding
CUST_6955_PI428871386	ENST00000432711.1	-1.45	5.70	4.11E-06	4.88E-05	lincRNA
CUST_24844_PI428871386	ENST00000533759.1	0.81	5.23	4.12E-06	4.90E-05	lincRNA
CUST_25862_PI428871386	ENST00000525716.2	-0.98	5.52	4.16E-06	4.94E-05	lincRNA
A_23_P42738	C7orf70	1.15	9.05	4.17E-06	4.95E-05	protein_coding
CUST_31348_PI428871386	ENST00000558846.1	-1.33	5.44	4.17E-06	4.95E-05	lincRNA
A_24_P169343	C9orf21	-1.34	8.35	4.18E-06	4.96E-05	protein_coding
A_24_P333733	ATP6V0A1	-1.26	12.07	4.19E-06	4.97E-05	protein_coding
CUST_17812_PI428871386	ENST00000467537.1	-1.51	8.35	4.20E-06	4.98E-05	antisense
CUST_21983_PI428871386	ENST00000442008.2	1.54	6.66	4.22E-06	5.01E-05	antisense
A_24_P274814	TBXAS1	-1.54	8.88	4.23E-06	5.01E-05	protein_coding
A_23_P88893	DEF8	1.41	9.88	4.26E-06	5.05E-05	protein_coding
A_33_P3263867	P2RX7	-2.03	8.23	4.30E-06	5.09E-05	protein_coding
CUST_13250_PI428871386	ENST00000504081.1	2.28	5.37	4.30E-06	5.09E-05	antisense
CUST_38769_PI428871386	ENST00000601797.1	1.47	6.47	4.30E-06	5.09E-05	antisense
CUST_42158_PI428871386	ENST00000432735.1	1.07	5.94	4.32E-06	5.11E-05	antisense
A_33_P3340164	NDUFB5	1.24	8.91	4.33E-06	5.13E-05	protein_coding
A_24_P343377	ATP5J2	1.03	12.07	4.34E-06	5.13E-05	protein_coding
A_24_P921321	PTPRJ	-1.50	8.66	4.39E-06	5.19E-05	protein_coding
A_23_P253571	PHF5A	1.02	8.62	4.39E-06	5.19E-05	protein_coding
A_24_P208345	SLC45A3	-1.50	8.92	4.39E-06	5.19E-05	protein_coding
CUST_17782_PI428871386	ENST00000439694.1	2.20	5.32	4.41E-06	5.22E-05	antisense
CUST_37524_PI428871386	ENST00000578497.1	2.34	5.57	4.43E-06	5.24E-05	lincRNA
CUST_43105_PI428871386	ENST00000456563.1	1.50	8.76	4.47E-06	5.29E-05	lincRNA
CUST_21986_PI428871386	ENST00000478294.1	1.99	5.27	4.48E-06	5.29E-05	lincRNA
A_23_P340019	NLRC3	-1.70	8.48	4.52E-06	5.34E-05	protein_coding
A_23_P143845	TIPARP	-1.89	10.56	4.59E-06	5.42E-05	protein_coding
CUST_35059_PI428871386	ENST00000572453.1	-1.01	5.58	4.61E-06	5.44E-05	antisense
CUST_33411_PI428871386	ENST00000567370.1	-1.14	5.52	4.65E-06	5.49E-05	lincRNA
CUST_17588_PI428871386	ENST00000593910.1	0.89	5.01	4.67E-06	5.51E-05	antisense
A_33_P3406836	HTR1F	-1.43	6.13	4.69E-06	5.53E-05	protein_coding
CUST_6860_PI428871386	ENST00000429929.1	2.00	6.24	4.72E-06	5.57E-05	antisense
CUST_6498_PI428871386	ENST00000425636.2	1.16	5.79	4.72E-06	5.57E-05	antisense
A_23_P81507	FAT2	1.46	5.40	4.74E-06	5.59E-05	protein_coding

CUST_6143_PI428871386	ENST00000420184.1	-0.86	6.36	4.75E-06	5.59E-05	lincRNA
A_33_P3240518	AURKAIP1	1.15	11.18	4.76E-06	5.60E-05	protein_coding
A_33_P3240115	SEPT8_	-0.97	6.59	4.77E-06	5.61E-05	protein_coding
CUST_28211_PI428871386	ENST00000448748.1	-1.03	4.81	4.83E-06	5.69E-05	lincRNA
A_32_P144920	NECAP1	-1.26	7.04	4.89E-06	5.76E-05	protein_coding
CUST_41021_PI428871386	ENST00000440918.1	-0.98	5.27	4.89E-06	5.76E-05	lincRNA
A_23_P162288	MYO1A	-1.29	6.00	4.90E-06	5.77E-05	protein_coding
A_33_P3279920	TARDBP	1.07	7.04	4.91E-06	5.78E-05	protein_coding
A_33_P3240018	PDE3B	-1.36	7.24	4.92E-06	5.78E-05	protein_coding
CUST_31489_PI428871386	ENST00000564805.1	-0.99	5.29	4.93E-06	5.79E-05	antisense
CUST_1496_PI428871386	ENST00000443802.1	1.96	7.37	4.94E-06	5.81E-05	lincRNA
CUST_23661_PI428871386	ENST00000456581.1	-0.89	6.19	4.96E-06	5.83E-05	lincRNA
A_33_P3399840	USP39	1.11	10.64	4.96E-06	5.83E-05	protein_coding
A_23_P362824	CSTF1	1.10	9.43	4.98E-06	5.85E-05	protein_coding
A_33_P3272563	NMT2	-1.23	6.54	4.98E-06	5.85E-05	protein_coding
CUST_40937_PI428871386	ENST00000435366.1	-1.41	10.83	4.99E-06	5.87E-05	antisense
A_24_P232365	APBB1IP	-1.72	8.56	4.99E-06	5.87E-05	protein_coding
A_23_P164228	ATP5G1	1.29	10.57	5.00E-06	5.87E-05	protein_coding
CUST_42282_PI428871386	ENST00000600937.1	-0.98	4.93	5.00E-06	5.87E-05	antisense
A_23_P139786	OASL	-2.12	9.16	5.02E-06	5.90E-05	protein_coding
A_23_P51487	GBP3	-1.88	8.63	5.04E-06	5.92E-05	protein_coding
CUST_43793_PI428871386	ENST00000424126.1	1.65	5.08	5.05E-06	5.93E-05	antisense
A_33_P3249135	NBEAL1	-1.39	7.81	5.10E-06	5.98E-05	protein_coding
A_24_P47547	RAN	1.22	10.98	5.11E-06	5.99E-05	protein_coding
CUST_42029_PI428871386	ENST00000342757.2	1.56	5.88	5.13E-06	6.02E-05	lincRNA
CUST_8371_PI428871386	ENST00000481312.1	-0.95	5.32	5.13E-06	6.02E-05	antisense
CUST_37703_PI428871386	ENST00000584531.1	0.89	5.14	5.14E-06	6.02E-05	lincRNA
CUST_21647_PI428871386	ENST00000444184.1	-1.32	9.94	5.14E-06	6.03E-05	lincRNA
CUST_1497_PI428871386	ENST00000443802.1	1.99	7.39	5.15E-06	6.03E-05	lincRNA
A_24_P185314	NAPG	-1.14	9.72	5.16E-06	6.04E-05	protein_coding
A_33_P3290622	PDZD11	1.18	7.90	5.16E-06	6.05E-05	protein_coding
A_23_P313632	FUT8	1.27	9.12	5.17E-06	6.05E-05	protein_coding
A_24_P233915	MED8	1.27	7.80	5.19E-06	6.07E-05	protein_coding
CUST_41502_PI428871386	ENST00000435697.1	-1.35	5.76	5.21E-06	6.10E-05	lincRNA
A_33_P3226395	LOC389634	-1.68	7.23	5.25E-06	6.15E-05	lincRNA
A_23_P368805	HHLA2	-1.21	5.19	5.29E-06	6.19E-05	protein_coding
A_23_P152727	PLEKHM1	-1.39	10.95	5.30E-06	6.20E-05	protein_coding
CUST_20211_PI428871386	ENST00000518049.1	1.40	5.90	5.30E-06	6.20E-05	antisense
A_23_P252764	SMARCA2	-1.43	9.00	5.31E-06	6.21E-05	protein_coding
CUST_238_PI428871386	ENST00000448624.2	-0.95	5.92	5.35E-06	6.25E-05	antisense
A_23_P54929	LYRM1	-1.33	7.66	5.36E-06	6.27E-05	protein_coding
CUST_5765_PI428871386	ENST00000419650.1	1.20	7.69	5.38E-06	6.28E-05	lincRNA
A_23_P42282	C4B	-2.09	10.79	5.38E-06	6.29E-05	protein_coding
A_33_P3364904	ZNF662	-1.35	6.87	5.39E-06	6.30E-05	protein_coding
A_32_P22401	MAP7D1	-1.48	11.31	5.41E-06	6.32E-05	protein_coding
A_32_P142818	DLX1	1.66	5.13	5.42E-06	6.33E-05	protein_coding
A_24_P942250	CHD9	-1.03	8.26	5.43E-06	6.33E-05	protein_coding
A_33_P3314441	FBXL17	-1.06	7.38	5.44E-06	6.35E-05	protein_coding
A_23_P160751	FCRL2	1.38	5.95	5.44E-06	6.35E-05	protein_coding
CUST_3304_PI428871386	ENST00000437764.1	-1.69	8.38	5.45E-06	6.36E-05	antisense
CUST_1180_PI428871386	ENST00000439156.1	1.27	5.22	5.46E-06	6.37E-05	lincRNA
A_24_P333571	ARHGAP29	-1.57	6.42	5.46E-06	6.37E-05	protein_coding
CUST_36221_PI428871386	ENST00000586348.1	-1.24	6.45	5.50E-06	6.41E-05	lincRNA
CUST_36713_PI428871386	ENST00000584959.1	-1.16	6.30	5.50E-06	6.41E-05	antisense
A_23_P62115	TIMP1	2.17	11.84	5.52E-06	6.43E-05	protein_coding
A_33_P3364821	PTPRC	-1.85	11.97	5.54E-06	6.46E-05	protein_coding
CUST_14287_PI428871386	ENST00000506340.1	1.19	7.03	5.56E-06	6.47E-05	antisense
A_23_P134835	CSGALNACT1	-2.06	8.77	5.56E-06	6.47E-05	protein_coding
CUST_26801_PI428871386	ENST00000550301.1	0.97	5.69	5.58E-06	6.49E-05	antisense
CUST_43869_PI428871386	ENST00000421353.1	0.93	5.06	5.60E-06	6.52E-05	lincRNA
CUST_5491_PI428871386	ENST00000567067.1	-1.21	8.38	5.60E-06	6.52E-05	lincRNA
A_32_P205053	UBXN10	-1.29	5.75	5.61E-06	6.52E-05	protein_coding
CUST_6660_PI428871386	ENST00000295549.3	2.36	7.21	5.61E-06	6.53E-05	lincRNA

A_33_P3336622	ALDH3A2	-1.78	10.35	5.64E-06	6.56E-05	protein_coding
A_24_P293530	CYP4X1	-2.18	7.54	5.65E-06	6.57E-05	protein_coding
A_23_P214091	LYPLA1	1.25	9.38	5.65E-06	6.57E-05	protein_coding
A_33_P3281985	CR2	2.73	6.76	5.67E-06	6.59E-05	protein_coding
CUST_38693_PI428871386	ENST00000591657.1	1.13	6.15	5.68E-06	6.60E-05	antisense
A_24_P706953	TMEM213	-2.26	6.26	5.69E-06	6.61E-05	protein_coding
CUST_1479_PI428871386	ENST00000528692.1	-1.15	7.01	5.70E-06	6.62E-05	antisense
CUST_17683_PI428871386	ENST00000587038.1	-0.92	4.97	5.74E-06	6.67E-05	antisense
A_24_P126651	TCEA1	1.45	8.95	5.75E-06	6.67E-05	protein_coding
CUST_36914_PI428871386	ENST00000544677.1	-0.93	4.79	5.79E-06	6.72E-05	lincRNA
CUST_25328_PI428871386	ENST00000530422.1	-1.81	11.30	5.80E-06	6.73E-05	antisense
A_33_P3390172	ADAMDEC1	1.76	5.47	5.84E-06	6.77E-05	protein_coding
A_32_P183609	ASB1	-1.15	6.99	5.84E-06	6.77E-05	protein_coding
A_24_P390909	PRX	-0.95	5.06	5.84E-06	6.77E-05	protein_coding
A_33_P3389286	SFN	2.22	13.06	5.85E-06	6.78E-05	protein_coding
A_33_P3387766	LOC284276	-1.35	5.97	5.85E-06	6.78E-05	lincRNA
CUST_8361_PI428871386	ENST00000597944.1	-0.90	5.06	5.86E-06	6.79E-05	antisense
A_33_P3318288	CFH	-1.76	11.36	5.91E-06	6.85E-05	protein_coding
CUST_32324_PI428871386	ENST00000560097.1	2.54	6.13	5.93E-06	6.88E-05	lincRNA
A_33_P3257042	ZNF850	1.86	6.50	5.95E-06	6.90E-05	protein_coding
CUST_29194_PI428871386	ENST00000561786.1	-1.22	5.24	5.98E-06	6.92E-05	lincRNA
CUST_26977_PI428871386	ENST00000556850.1	-1.01	6.10	6.00E-06	6.95E-05	protein_coding
A_33_P3365087	PRR20B	-1.25	6.26	6.00E-06	6.95E-05	protein_coding
CUST_33528_PI428871386	ENST00000563565.1	0.97	6.22	6.00E-06	6.95E-05	antisense
A_33_P3214209	GUSBP1	1.42	7.55	6.02E-06	6.96E-05	lincRNA
CUST_27984_PI428871386	ENST00000545767.1	-0.89	4.89	6.10E-06	7.05E-05	lincRNA
CUST_37218_PI428871386	ENST00000582106.1	1.17	6.21	6.11E-06	7.06E-05	antisense
CUST_26047_PI428871386	ENST00000543206.1	-1.95	6.41	6.11E-06	7.06E-05	lincRNA
A_33_P3252083	GRIP1	1.04	5.39	6.11E-06	7.07E-05	protein_coding
A_24_P407259	SOX21	1.54	5.49	6.13E-06	7.09E-05	protein_coding
CUST_31488_PI428871386	ENST00000564805.1	-1.02	5.04	6.16E-06	7.11E-05	antisense
A_24_P193592	CCNF	0.93	5.17	6.16E-06	7.12E-05	protein_coding
CUST_20889_PI428871386	ENST00000432148.1	-1.13	6.81	6.18E-06	7.13E-05	lincRNA
CUST_3299_PI428871386	ENST00000441672.1	-1.42	6.29	6.19E-06	7.14E-05	antisense
CUST_3550_PI428871386	ENST00000445817.1	1.53	8.37	6.19E-06	7.14E-05	lincRNA
A_33_P3315243	C17orf72	-1.46	6.86	6.22E-06	7.18E-05	protein_coding
A_23_P502553	TRIM35	-1.08	8.24	6.23E-06	7.19E-05	protein_coding
CUST_28673_PI428871386	ENST00000433480.2	-0.94	5.25	6.26E-06	7.22E-05	lincRNA
CUST_41187_PI428871386	ENST00000431019.1	0.89	4.97	6.27E-06	7.23E-05	antisense
A_33_P3406196	KLRD1	-1.30	6.01	6.27E-06	7.23E-05	protein_coding
A_23_P163209	BCL2L10	1.63	5.47	6.28E-06	7.24E-05	protein_coding
CUST_33525_PI428871386	ENST00000568183.1	1.05	5.92	6.29E-06	7.25E-05	antisense
A_24_P248240	SYT11	-1.38	7.17	6.30E-06	7.26E-05	protein_coding
A_33_P3289236	HPR	-2.78	10.64	6.33E-06	7.29E-05	protein_coding
CUST_24907_PI428871386	ENST00000532296.1	1.14	5.54	6.33E-06	7.29E-05	antisense
A_33_P3251727	RYR2	-1.44	5.40	6.34E-06	7.30E-05	protein_coding
A_23_P78170	MYBBP1A	1.19	7.47	6.35E-06	7.32E-05	protein_coding
CUST_11597_PI428871386	ENST00000507152.1	1.78	5.36	6.38E-06	7.34E-05	protein_coding
A_23_P59426	PAXIP1	1.09	8.66	6.42E-06	7.39E-05	protein_coding
A_23_P14975	C16orf48	-1.18	8.90	6.48E-06	7.46E-05	protein_coding
CUST_26646_PI428871386	ENST00000551432.1	-1.49	8.05	6.49E-06	7.47E-05	lincRNA
A_23_P85598	ADCK3	-1.21	8.35	6.49E-06	7.47E-05	protein_coding
A_24_P309317	PSAP	-1.92	11.15	6.50E-06	7.47E-05	protein_coding
CUST_32120_PI428871386	ENST00000567257.1	1.48	6.52	6.50E-06	7.47E-05	antisense
A_23_P503182	ABR	-1.59	11.34	6.53E-06	7.50E-05	protein_coding
CUST_28362_PI428871386	ENST00000323380.4	0.95	5.19	6.53E-06	7.51E-05	antisense
CUST_24068_PI428871386	ENST00000529328.1	2.21	5.64	6.55E-06	7.53E-05	lincRNA
CUST_16164_PI428871386	ENST00000433388.1	-1.02	5.63	6.55E-06	7.53E-05	lincRNA
A_33_P3282075	SP6	1.82	7.02	6.55E-06	7.53E-05	protein_coding
A_24_P124992	PSMA4	1.13	11.44	6.61E-06	7.59E-05	protein_coding
A_24_P418816	GPX7	1.30	6.82	6.62E-06	7.60E-05	protein_coding
CUST_14569_PI428871386	ENST00000423504.1	-1.01	5.59	6.63E-06	7.61E-05	lincRNA
A_33_P3213747	ZNHIT2	1.10	7.37	6.68E-06	7.67E-05	protein_coding

A_23_P56256	POLR2I	1.27	10.72	6.73E-06	7.72E-05	protein_coding
A_23_P152235	IRX3	-2.07	9.98	6.74E-06	7.73E-05	protein_coding
CUST_16387_PI428871386	ENST00000418428.2	-1.38	5.32	6.78E-06	7.77E-05	lincRNA
A_33_P3286958	B3GALT2	-1.44	6.21	6.83E-06	7.83E-05	protein_coding
A_24_P251661	KCNMB3	1.28	5.89	6.83E-06	7.83E-05	protein_coding
CUST_25870_PI428871386	ENST00000416553.1	-1.43	5.41	6.84E-06	7.84E-05	lincRNA
A_32_P205553	RPL26L1	0.98	10.98	6.85E-06	7.84E-05	protein_coding
A_33_P3210762	ADAMTS7	-0.95	5.54	6.89E-06	7.89E-05	protein_coding
CUST_14639_PI428871386	ENST00000421692.1	0.97	5.56	6.91E-06	7.91E-05	antisense
A_33_P3225327	ATP13A3	1.04	7.62	6.93E-06	7.93E-05	protein_coding
CUST_33526_PI428871386	ENST00000568183.1	1.00	5.97	6.93E-06	7.93E-05	antisense
CUST_24968_PI428871386	ENST00000533327.1	-0.99	4.82	6.93E-06	7.93E-05	antisense
A_23_P371787	KIAA0247	-1.32	11.34	6.96E-06	7.97E-05	protein_coding
A_23_P146209	RNF170	1.14	8.05	7.04E-06	8.05E-05	protein_coding
A_24_P11436	TTC22	1.16	5.93	7.06E-06	8.07E-05	protein_coding
CUST_5453_PI428871386	ENST00000409054.1	1.34	8.77	7.07E-06	8.08E-05	lincRNA
CUST_36220_PI428871386	ENST00000586348.1	-1.13	6.49	7.07E-06	8.08E-05	lincRNA
CUST_30256_PI428871386	ENST00000560419.1	-1.50	7.19	7.09E-06	8.10E-05	antisense
A_24_P381505	GLIPR1L2	-1.08	5.90	7.09E-06	8.10E-05	protein_coding
CUST_39527_PI428871386	ENST00000424536.1	1.16	6.51	7.09E-06	8.10E-05	protein_coding
CUST_15125_PI428871386	ENST00000585945.1	-1.16	5.64	7.12E-06	8.13E-05	antisense
A_23_P202587	KIAA1598	-1.36	9.27	7.12E-06	8.13E-05	protein_coding
A_24_P142503	SLC47A1	-1.67	6.56	7.13E-06	8.14E-05	protein_coding
A_32_P56392	RBMX	1.01	6.89	7.14E-06	8.15E-05	protein_coding
CUST_37953_PI428871386	ENST00000600644.1	2.13	5.36	7.14E-06	8.15E-05	lincRNA
CUST_34861_PI428871386	ENST00000570711.1	-1.43	6.07	7.17E-06	8.18E-05	antisense
A_33_P3271051	CYTH3	-1.34	10.77	7.22E-06	8.24E-05	protein_coding
A_23_P8380	C7orf49	1.03	8.67	7.23E-06	8.24E-05	protein_coding
CUST_19981_PI428871386	ENST00000517525.1	1.05	5.82	7.23E-06	8.24E-05	lincRNA
CUST_861_PI428871386	ENST00000566366.1	1.18	5.27	7.23E-06	8.25E-05	antisense
CUST_20275_PI428871386	ENST00000527086.1	-0.91	7.10	7.24E-06	8.25E-05	antisense
A_33_P3317603	B4GALNT4	1.45	5.17	7.26E-06	8.27E-05	protein_coding
CUST_2635_PI428871386	ENST00000423943.1	1.69	6.07	7.29E-06	8.30E-05	lincRNA
CUST_14141_PI428871386	ENST00000510150.1	1.01	5.21	7.33E-06	8.35E-05	lincRNA
A_32_P219942	ZIC5	2.12	5.27	7.37E-06	8.39E-05	protein_coding
CUST_4011_PI428871386	ENST00000416463.1	-1.21	7.77	7.42E-06	8.44E-05	lincRNA
A_32_P9382	MZT1	1.27	8.80	7.43E-06	8.45E-05	protein_coding
A_23_P389391	SLC12A4	-1.22	8.22	7.48E-06	8.50E-05	protein_coding
A_23_P310560	NUDT16	-1.17	7.99	7.49E-06	8.52E-05	protein_coding
CUST_21411_PI428871386	ENST00000448674.1	-0.91	6.39	7.56E-06	8.60E-05	lincRNA
CUST_6341_PI428871386	ENST00000420548.1	0.90	4.82	7.57E-06	8.61E-05	lincRNA
CUST_37757_PI428871386	ENST00000579356.1	1.59	5.51	7.60E-06	8.63E-05	antisense
A_33_P3883912	ZCCHC10	-0.97	8.79	7.60E-06	8.64E-05	protein_coding
CUST_949_PI428871386	ENST00000440985.1	1.04	5.67	7.69E-06	8.74E-05	antisense
A_33_P3335845	WDR33	1.19	10.38	7.71E-06	8.76E-05	protein_coding
A_33_P3422010	DNAH12	-1.48	5.64	7.71E-06	8.76E-05	protein_coding
CUST_38783_PI428871386	ENST00000593581.1	-0.99	8.84	7.74E-06	8.78E-05	antisense
CUST_6807_PI428871386	ENST00000431752.1	-0.92	5.62	7.75E-06	8.79E-05	antisense
A_33_P3272291	AKR1C4	2.58	7.52	7.76E-06	8.80E-05	protein_coding
A_32_P104746	ZFYVE28	-1.32	7.35	7.76E-06	8.80E-05	protein_coding
A_24_P131066	CD101	-1.06	5.49	7.83E-06	8.88E-05	protein_coding
A_33_P3411628	CDKN2A	2.88	7.24	7.85E-06	8.90E-05	protein_coding
A_23_P154849	OLIG1	-1.65	6.00	7.87E-06	8.92E-05	protein_coding
CUST_5074_PI428871386	ENST00000441630.1	-1.00	5.21	7.90E-06	8.95E-05	antisense
CUST_33583_PI428871386	ENST00000398859.3	1.72	5.60	7.90E-06	8.95E-05	antisense
A_24_P109214	APOC1	-2.61	12.58	7.91E-06	8.96E-05	protein_coding
A_23_P358597	POPDC3	1.90	5.77	7.92E-06	8.97E-05	protein_coding
A_23_P333852	TTLL11	-0.89	5.88	7.94E-06	8.98E-05	protein_coding
A_33_P3308833	C21orf58	1.06	5.74	7.94E-06	8.99E-05	protein_coding
CUST_8666_PI428871386	ENST00000563632.1	-1.41	7.23	7.98E-06	9.03E-05	antisense
A_24_P943095	SPECC1	1.05	5.80	7.99E-06	9.04E-05	protein_coding
CUST_27705_PI428871386	ENST00000551125.1	-0.86	5.46	8.02E-06	9.08E-05	antisense
CUST_14440_PI428871386	ENST00000439891.1	-0.99	5.14	8.05E-06	9.11E-05	lincRNA

CUST_40340_PI428871386	ENST00000589888.1	-1.03	5.29	8.11E-06	9.17E-05	lincRNA
A_23_P80827	FYTTD1	1.00	9.48	8.11E-06	9.17E-05	protein_coding
CUST_33584_PI428871386	ENST00000398859.3	1.78	5.66	8.14E-06	9.20E-05	antisense
CUST_16594_PI428871386	ENST00000521687.1	-1.12	5.66	8.14E-06	9.20E-05	antisense
CUST_29900_PI428871386	ENST00000556417.1	1.61	5.28	8.16E-06	9.22E-05	lincRNA
CUST_43794_PI428871386	ENST00000424126.1	1.72	4.99	8.20E-06	9.27E-05	antisense
A_33_P3365117	AKR1C1	2.22	5.88	8.22E-06	9.28E-05	protein_coding
A_33_P3251841	DSEL	-1.49	7.97	8.22E-06	9.28E-05	protein_coding
CUST_37219_PI428871386	ENST00000582106.1	1.09	6.17	8.23E-06	9.29E-05	antisense
CUST_27020_PI428871386	ENST00000547590.1	1.02	5.09	8.26E-06	9.33E-05	antisense
A_33_P3276718	HGF	-1.66	6.41	8.33E-06	9.40E-05	protein_coding
A_24_P167614	INTS6	1.15	7.87	8.33E-06	9.40E-05	protein_coding
A_33_P3280950	LOC144571	-1.29	7.82	8.37E-06	9.44E-05	antisense
CUST_19611_PI428871386	ENST00000523831.1	1.22	5.25	8.37E-06	9.44E-05	antisense
CUST_928_PI428871386	ENST00000418149.1	1.31	5.53	8.38E-06	9.45E-05	antisense
CUST_29596_PI428871386	ENST00000552511.1	1.57	5.26	8.38E-06	9.45E-05	antisense
CUST_26078_PI428871386	ENST00000544842.1	1.87	5.86	8.41E-06	9.47E-05	lincRNA
CUST_19127_PI428871386	ENST00000523265.1	1.26	5.41	8.42E-06	9.48E-05	lincRNA
A_23_P203255	API5	1.00	7.35	8.45E-06	9.52E-05	protein_coding
A_33_P3366336	SBK1	2.05	7.59	8.47E-06	9.53E-05	protein_coding
CUST_5245_PI428871386	ENST00000416395.1	-1.28	8.36	8.49E-06	9.55E-05	antisense
A_23_P84154	ARHGAP15	-1.63	7.63	8.50E-06	9.56E-05	protein_coding
A_23_P29684	VILL	-1.46	7.91	8.65E-06	9.72E-05	protein_coding
A_23_P27744	FBXO46	0.98	8.84	8.68E-06	9.76E-05	protein_coding
CUST_8931_PI428871386	ENST00000459861.1	1.61	5.05	8.68E-06	9.76E-05	lincRNA
A_33_P3255914	MYLIP	-1.57	10.60	8.71E-06	9.79E-05	protein_coding
CUST_19707_PI428871386	ENST00000520594.1	2.53	5.48	8.75E-06	9.83E-05	antisense
CUST_26049_PI428871386	ENST00000536141.1	-1.84	6.29	8.77E-06	9.86E-05	lincRNA
CUST_40454_PI428871386	ENST00000594816.1	-1.24	6.44	8.80E-06	9.88E-05	lincRNA
A_23_P250294	ABHD5	-1.39	9.49	8.86E-06	9.95E-05	protein_coding
CUST_34914_PI428871386	ENST00000575626.1	-1.55	6.93	8.90E-06	9.99E-05	lincRNA
CUST_5031_PI428871386	ENST00000443946.1	1.01	6.59	8.94E-06	1.00E-04	lincRNA
CUST_40339_PI428871386	ENST00000589888.1	-1.01	5.39	8.94E-06	1.00E-04	lincRNA
CUST_7218_PI428871386	ENST00000413311.1	-1.32	6.34	8.97E-06	1.01E-04	lincRNA
A_23_P308305	TTC39C	1.44	7.93	9.00E-06	1.01E-04	protein_coding
CUST_3880_PI428871386	ENST00000592090.1	-1.10	6.59	9.03E-06	1.01E-04	lincRNA
A_23_P27013	HOXB9	2.70	5.84	9.05E-06	1.01E-04	protein_coding
A_33_P3232945	F2RL1	1.76	7.68	9.11E-06	1.02E-04	protein_coding
A_33_P3342160	ATG2B	-1.00	8.45	9.13E-06	1.02E-04	protein_coding
A_33_P3242124	HAUS8	1.11	5.91	9.16E-06	1.03E-04	protein_coding
A_32_P182299	C1orf168	-1.50	5.76	9.23E-06	1.03E-04	protein_coding
CUST_15090_PI428871386	ENST00000419979.1	-1.47	5.96	9.24E-06	1.03E-04	antisense
CUST_17316_PI428871386	ENST00000430027.2	1.97	5.20	9.25E-06	1.03E-04	antisense
A_24_P288890	FAM101A	1.63	5.38	9.26E-06	1.04E-04	protein_coding
A_33_P3223544	TMBIM4	-1.14	12.67	9.28E-06	1.04E-04	protein_coding
CUST_37525_PI428871386	ENST00000578497.1	2.09	5.53	9.30E-06	1.04E-04	lincRNA
CUST_37037_PI428871386	ENST00000582422.1	-1.20	5.58	9.34E-06	1.04E-04	lincRNA
CUST_21515_PI428871386	ENST00000429482.1	-0.98	5.54	9.41E-06	1.05E-04	antisense
A_23_P25121	FKBP11	1.86	11.33	9.47E-06	1.06E-04	protein_coding
CUST_18805_PI428871386	ENST00000523459.1	1.62	5.41	9.51E-06	1.06E-04	antisense
A_24_P273253	AHNAK2	2.07	6.47	9.52E-06	1.06E-04	protein_coding
A_33_P3239587	MXRA7	-1.40	13.73	9.56E-06	1.07E-04	protein_coding
CUST_24073_PI428871386	ENST00000526388.1	2.17	5.62	9.71E-06	1.08E-04	lincRNA
A_24_P414371	PPP3CA	-1.04	8.74	9.77E-06	1.09E-04	protein_coding
A_33_P3264042	EFCAB3	1.24	6.00	9.80E-06	1.09E-04	protein_coding
A_23_P389907	ATXN2	-0.97	10.32	9.81E-06	1.09E-04	protein_coding
CUST_28495_PI428871386	ENST00000454681.1	-0.96	5.36	9.96E-06	1.11E-04	lincRNA
A_23_P394448	DPY19L1	1.55	7.07	1.00E-05	1.12E-04	protein_coding
CUST_16583_PI428871386	ENST00000518046.1	-1.23	5.91	1.00E-05	1.12E-04	antisense
A_32_P92399	COG8	1.09	9.62	1.01E-05	1.12E-04	protein_coding
CUST_29898_PI428871386	ENST00000554221.1	1.61	5.24	1.01E-05	1.12E-04	lincRNA
A_33_P3877349	LOC440173	1.69	5.26	1.01E-05	1.12E-04	lincRNA
A_23_P422851	CABLES1	-1.86	9.67	1.01E-05	1.13E-04	protein_coding

CUST_41894_PI428871386	ENST00000454482.2	1.09	5.61	1.01E-05	1.13E-04	lincRNA
CUST_6920_PI428871386	ENST00000599681.1	-0.85	4.87	1.01E-05	1.13E-04	antisense
CUST_37756_PI428871386	ENST00000579356.1	1.67	5.46	1.02E-05	1.13E-04	antisense
CUST_13189_PI428871386	ENST00000505527.1	1.71	5.57	1.02E-05	1.14E-04	lincRNA
CUST_18200_PI428871386	ENST00000523225.1	1.22	5.69	1.02E-05	1.14E-04	antisense
A_24_P388786	DNAH5	-2.36	7.97	1.02E-05	1.14E-04	protein_coding
CUST_41147_PI428871386	ENST00000441722.1	1.35	12.26	1.03E-05	1.15E-04	antisense
A_33_P3344169	DYX1C1	-1.18	6.47	1.03E-05	1.15E-04	protein_coding
A_33_P3250820	FREM2	-1.99	6.70	1.03E-05	1.15E-04	protein_coding
A_23_P3532	LITAF	-1.42	10.08	1.04E-05	1.15E-04	protein_coding
CUST_15967_PI428871386	ENST00000455229.1	-1.00	4.79	1.04E-05	1.15E-04	lincRNA
CUST_24071_PI428871386	ENST00000531402.1	2.27	5.57	1.04E-05	1.15E-04	lincRNA
A_23_P378690	TMEM64	-1.26	7.10	1.04E-05	1.15E-04	protein_coding
A_23_P211244	PRMT2	-1.09	8.29	1.04E-05	1.15E-04	protein_coding
A_23_P148255	MAGEA2B	3.33	6.39	1.04E-05	1.16E-04	protein_coding
CUST_21624_PI428871386	ENST00000455981.1	-1.15	6.01	1.04E-05	1.16E-04	lincRNA
CUST_22012_PI428871386	ENST00000445427.1	-1.45	6.52	1.05E-05	1.16E-04	lincRNA
A_33_P3213463	NDUFS8	1.15	11.79	1.05E-05	1.16E-04	protein_coding
A_24_P250335	SNRPA	0.98	9.24	1.05E-05	1.16E-04	protein_coding
CUST_12686_PI428871386	ENST00000502659.2	-1.68	5.48	1.05E-05	1.17E-04	lincRNA
CUST_21574_PI428871386	ENST00000588890.1	0.82	5.57	1.06E-05	1.17E-04	antisense
CUST_27391_PI428871386	ENST00000548575.1	-0.96	4.80	1.06E-05	1.17E-04	lincRNA
A_23_P127652	ZNF202	1.08	6.06	1.06E-05	1.18E-04	protein_coding
A_33_P3361182	PDCD11	1.09	6.56	1.06E-05	1.18E-04	protein_coding
A_23_P256773	TSSC1	1.10	9.94	1.06E-05	1.18E-04	protein_coding
CUST_35031_PI428871386	ENST00000570712.1	1.49	8.05	1.07E-05	1.18E-04	antisense
CUST_26409_PI428871386	ENST00000540811.1	-1.65	6.66	1.07E-05	1.18E-04	lincRNA
CUST_15064_PI428871386	ENST00000418368.1	1.53	9.03	1.07E-05	1.19E-04	lincRNA
CUST_15075_PI428871386	ENST00000429530.1	1.97	5.45	1.08E-05	1.19E-04	antisense
A_23_P371765	C21orf56	1.29	6.24	1.08E-05	1.19E-04	protein_coding
A_32_P184488	PHLDB3	1.61	8.68	1.08E-05	1.19E-04	protein_coding
CUST_19877_PI428871386	ENST00000519861.1	1.12	6.45	1.08E-05	1.20E-04	lincRNA
CUST_274_PI428871386	ENST00000418088.1	-1.21	6.80	1.08E-05	1.20E-04	antisense
CUST_6859_PI428871386	ENST00000429929.1	1.88	6.67	1.09E-05	1.20E-04	antisense
CUST_14969_PI428871386	ENST00000448327.1	2.28	5.57	1.10E-05	1.21E-04	antisense
A_23_P309850	RPUSD2	1.12	9.40	1.10E-05	1.21E-04	protein_coding
A_33_P3276475	CHMP1B	-1.07	9.10	1.10E-05	1.22E-04	protein_coding
A_23_P71053	MPP6	1.54	6.49	1.10E-05	1.22E-04	protein_coding
CUST_21648_PI428871386	ENST00000444184.1	-1.30	9.86	1.10E-05	1.22E-04	lincRNA
A_24_P380919	HNRNPK	-0.88	11.70	1.11E-05	1.22E-04	protein_coding
A_23_P64630	RNF26	1.18	10.02	1.11E-05	1.22E-04	protein_coding
CUST_25483_PI428871386	ENST00000529938.1	-0.90	4.72	1.11E-05	1.23E-04	lincRNA
CUST_34801_PI428871386	ENST00000562855.1	-1.05	10.58	1.12E-05	1.23E-04	protein_coding
A_33_P3363938	NAP1L4	1.05	6.01	1.12E-05	1.24E-04	protein_coding
CUST_27224_PI428871386	ENST00000550334.1	-1.14	5.11	1.13E-05	1.24E-04	antisense
A_23_P33683	MARCH2_	-1.15	6.86	1.13E-05	1.24E-04	protein_coding
A_23_P141863	ZNF544	1.12	7.48	1.13E-05	1.24E-04	protein_coding
A_24_P933418	ABI2	-1.21	8.20	1.14E-05	1.26E-04	protein_coding
CUST_1877_PI428871386	ENST00000416193.1	1.41	7.39	1.15E-05	1.26E-04	antisense
CUST_43790_PI428871386	ENST00000411474.1	1.48	5.24	1.15E-05	1.27E-04	antisense
CUST_38720_PI428871386	ENST00000590789.1	-0.93	5.96	1.15E-05	1.27E-04	lincRNA
CUST_13138_PI428871386	ENST00000512856.1	-1.20	5.91	1.15E-05	1.27E-04	antisense
A_24_P414376	KLF3	-1.10	10.59	1.17E-05	1.29E-04	protein_coding
CUST_10851_PI428871386	ENST00000511543.1	-1.02	6.34	1.17E-05	1.29E-04	antisense
CUST_43792_PI428871386	ENST00000445330.1	2.13	5.33	1.18E-05	1.30E-04	antisense
CUST_41790_PI428871386	ENST00000421051.1	-0.91	4.93	1.19E-05	1.31E-04	lincRNA
CUST_24760_PI428871386	ENST00000457725.1	-0.94	5.25	1.20E-05	1.32E-04	lincRNA
CUST_36552_PI428871386	ENST00000579527.1	-1.33	7.80	1.20E-05	1.32E-04	antisense
A_23_P387000	XKR6	-1.03	5.40	1.21E-05	1.33E-04	protein_coding
A_23_P161659	SYT13	3.00	6.12	1.23E-05	1.35E-04	protein_coding
CUST_7490_PI428871386	ENST00000446979.1	1.48	7.66	1.24E-05	1.36E-04	lincRNA
A_24_P123833	SEMA3E	-1.25	5.37	1.24E-05	1.36E-04	protein_coding
A_33_P3240702	RBBP8	1.48	8.93	1.24E-05	1.37E-04	protein_coding

A_33_P3298577	SBNO1	1.20	7.27	1.25E-05	1.37E-04	protein_coding
CUST_23735_PI428871386	ENST00000524824.1	-1.26	5.75	1.25E-05	1.37E-04	lincRNA
A_33_P3217649	C9orf116	-1.67	6.61	1.25E-05	1.37E-04	protein_coding
A_23_P3193	GOLGA5	1.00	8.73	1.25E-05	1.37E-04	protein_coding
CUST_25125_PI428871386	ENST00000530759.1	1.49	7.47	1.25E-05	1.37E-04	lincRNA
A_33_P3217480	HIRIP3	-1.28	10.17	1.25E-05	1.37E-04	protein_coding
A_33_P3334590	MTMR10	-1.10	6.97	1.25E-05	1.37E-04	protein_coding
A_24_P282762	TLK1	0.96	6.47	1.25E-05	1.38E-04	protein_coding
CUST_25329_PI428871386	ENST00000530422.1	-1.76	11.53	1.26E-05	1.38E-04	antisense
CUST_29599_PI428871386	ENST00000550680.1	1.69	5.26	1.26E-05	1.38E-04	antisense
A_23_P66421	NAT9	1.11	9.98	1.26E-05	1.38E-04	protein_coding
CUST_29597_PI428871386	ENST00000552511.1	1.58	5.24	1.27E-05	1.39E-04	antisense
CUST_37091_PI428871386	ENST00000590023.1	1.69	6.55	1.27E-05	1.39E-04	lincRNA
CUST_34726_PI428871386	ENST00000566351.1	-0.97	5.40	1.27E-05	1.39E-04	lincRNA
CUST_39516_PI428871386	ENST00000561778.1	-1.07	4.94	1.28E-05	1.40E-04	lincRNA
A_24_P377489	CCZ1	1.04	11.46	1.29E-05	1.41E-04	protein_coding
A_23_P27332	TCF4	-1.43	11.94	1.29E-05	1.41E-04	protein_coding
CUST_8534_PI428871386	ENST00000502999.1	1.24	10.23	1.29E-05	1.42E-04	antisense
A_33_P3280845	THY1	1.79	11.35	1.30E-05	1.42E-04	protein_coding
CUST_37956_PI428871386	ENST00000595135.1	1.00	5.15	1.30E-05	1.42E-04	lincRNA
CUST_42719_PI428871386	ENST00000442126.1	-1.04	5.31	1.30E-05	1.42E-04	antisense
A_33_P3304824	ENST00000435624	1.41	6.24	1.31E-05	1.43E-04	lincRNA
CUST_41500_PI428871386	ENST00000428669.1	-1.45	8.04	1.31E-05	1.43E-04	lincRNA
A_24_P397247	SDAD1	1.05	8.81	1.31E-05	1.43E-04	protein_coding
CUST_34908_PI428871386	ENST00000571091.1	-1.52	6.84	1.31E-05	1.43E-04	lincRNA
CUST_38876_PI428871386	ENST00000590065.1	-0.92	6.80	1.31E-05	1.43E-04	lincRNA
A_33_P3329974	CGN	-1.98	10.87	1.32E-05	1.44E-04	protein_coding
CUST_22240_PI428871386	ENST00000454991.1	0.86	5.04	1.32E-05	1.44E-04	lincRNA
CUST_30869_PI428871386	ENST00000559946.1	1.47	6.52	1.32E-05	1.44E-04	lincRNA
CUST_37220_PI428871386	ENST00000583492.1	1.09	6.22	1.33E-05	1.45E-04	antisense
A_24_P110983	AKT3	-1.65	8.59	1.34E-05	1.46E-04	protein_coding
CUST_12094_PI428871386	ENST00000511758.1	-1.31	5.32	1.34E-05	1.46E-04	lincRNA
CUST_37039_PI428871386	ENST00000581657.1	-1.11	5.67	1.34E-05	1.46E-04	lincRNA
CUST_14745_PI428871386	ENST00000429600.1	-1.27	7.70	1.35E-05	1.47E-04	lincRNA
CUST_36589_PI428871386	ENST00000585190.1	-1.45	7.71	1.35E-05	1.47E-04	antisense
CUST_24845_PI428871386	ENST00000533759.1	0.79	5.11	1.36E-05	1.48E-04	lincRNA
A_33_P3215128	IBA57	-1.05	6.79	1.37E-05	1.50E-04	protein_coding
CUST_43441_PI428871386	ENST00000445814.1	-1.26	5.55	1.41E-05	1.53E-04	lincRNA
CUST_14739_PI428871386	ENST00000453426.1	-1.33	7.67	1.41E-05	1.54E-04	lincRNA
A_33_P3326992	PDE4DIP	-1.42	7.56	1.41E-05	1.54E-04	protein_coding
CUST_17688_PI428871386	ENST00000469264.1	-1.05	5.04	1.42E-05	1.54E-04	antisense
A_33_P3666346	AHCTF1	1.21	6.71	1.42E-05	1.54E-04	protein_coding
CUST_26738_PI428871386	ENST00000553259.1	-1.05	7.23	1.42E-05	1.54E-04	antisense
CUST_3302_PI428871386	ENST00000475406.1	-1.62	8.41	1.42E-05	1.54E-04	antisense
CUST_10477_PI428871386	ENST00000411630.2	1.39	9.46	1.43E-05	1.55E-04	lincRNA
A_23_P416395	STC2	1.35	6.11	1.43E-05	1.55E-04	protein_coding
CUST_31883_PI428871386	ENST00000560622.1	1.02	5.63	1.43E-05	1.56E-04	antisense
CUST_40491_PI428871386	ENST00000447956.1	-1.98	6.94	1.43E-05	1.56E-04	lincRNA
CUST_9209_PI428871386	ENST00000484721.1	-0.91	4.67	1.44E-05	1.56E-04	antisense
A_33_P3239620	SPINT3	-0.91	5.15	1.44E-05	1.56E-04	protein_coding
A_24_P171983	NDUFAF4	1.20	8.49	1.44E-05	1.56E-04	protein_coding
A_23_P152284	SNRNP25	1.22	8.72	1.44E-05	1.56E-04	protein_coding
A_33_P3334535	GIN1	-0.97	6.92	1.44E-05	1.57E-04	protein_coding
A_23_P433050	RXFP1	-1.08	5.47	1.44E-05	1.57E-04	protein_coding
CUST_17421_PI428871386	ENST00000450686.1	-1.03	5.54	1.45E-05	1.57E-04	antisense
CUST_25186_PI428871386	ENST00000513207.2	-1.19	5.78	1.45E-05	1.58E-04	lincRNA
A_33_P3421028	ROS1	-1.91	6.91	1.45E-05	1.58E-04	protein_coding
A_24_P379512	PIGK	-1.18	7.48	1.45E-05	1.58E-04	protein_coding
CUST_43789_PI428871386	ENST00000411474.1	1.92	5.17	1.46E-05	1.58E-04	antisense
A_23_P43119	PSKH2	-1.01	5.00	1.46E-05	1.59E-04	protein_coding
CUST_14075_PI428871386	ENST00000519755.1	-0.88	4.67	1.47E-05	1.59E-04	lincRNA
A_33_P3308862	FAM22A	1.99	6.17	1.47E-05	1.59E-04	protein_coding
CUST_33852_PI428871386	ENST00000566876.1	1.65	5.40	1.47E-05	1.60E-04	lincRNA

A_33_P3367196	CNTNAP2	1.90	5.42	1.48E-05	1.61E-04	protein_coding
CUST_5860_PI428871386	ENST00000451884.1	1.23	6.69	1.50E-05	1.62E-04	lincRNA
A_32_P701268	MED12L	1.16	4.99	1.51E-05	1.63E-04	protein_coding
CUST_10170_PI428871386	ENST00000503938.1	1.06	4.95	1.51E-05	1.63E-04	lincRNA
A_33_P3344201	AKT1S1	0.92	8.29	1.51E-05	1.64E-04	protein_coding
A_23_P35782	NAA40	1.04	6.37	1.52E-05	1.64E-04	protein_coding
CUST_33623_PI428871386	ENST00000569752.1	1.13	5.74	1.52E-05	1.64E-04	antisense
CUST_17339_PI428871386	ENST00000431679.1	-1.28	7.26	1.52E-05	1.64E-04	lincRNA
A_23_P144384	GALNT7	1.27	9.47	1.52E-05	1.64E-04	protein_coding
CUST_8011_PI428871386	ENST00000366441.2	-1.05	5.74	1.52E-05	1.65E-04	antisense
CUST_18391_PI428871386	ENST00000517798.1	-0.93	5.24	1.54E-05	1.66E-04	antisense
CUST_16201_PI428871386	ENST00000465755.1	-0.86	4.57	1.54E-05	1.66E-04	lincRNA
A_23_P80068	BTG3	-1.28	12.13	1.55E-05	1.67E-04	protein_coding
A_24_P419132	CENPI	1.19	5.17	1.55E-05	1.67E-04	protein_coding
A_23_P119464	RHPN2	1.45	6.73	1.55E-05	1.67E-04	protein_coding
A_32_P121085	DOK3	-1.60	8.54	1.55E-05	1.67E-04	protein_coding
A_23_P32279	BARX1	1.95	5.40	1.55E-05	1.67E-04	protein_coding
CUST_9226_PI428871386	ENST00000492937.1	1.49	6.44	1.56E-05	1.68E-04	antisense
CUST_22822_PI428871386	ENST00000456638.1	-1.13	7.23	1.57E-05	1.69E-04	antisense
CUST_17370_PI428871386	ENST00000441882.1	-0.97	4.87	1.57E-05	1.69E-04	antisense
A_24_P916496	PRKCA	-1.80	9.37	1.57E-05	1.69E-04	protein_coding
A_23_P300826	C6orf136	1.07	7.68	1.57E-05	1.69E-04	protein_coding
CUST_43401_PI428871386	ENST00000431103.1	1.08	5.33	1.57E-05	1.69E-04	antisense
CUST_934_PI428871386	ENST00000445226.1	1.11	5.81	1.58E-05	1.70E-04	antisense
CUST_27704_PI428871386	ENST00000551125.1	-0.89	5.41	1.58E-05	1.70E-04	antisense
CUST_1402_PI428871386	ENST00000443939.1	0.82	5.09	1.59E-05	1.71E-04	antisense
A_24_P636332	CCDC84	-1.21	9.80	1.60E-05	1.72E-04	protein_coding
CUST_1181_PI428871386	ENST00000439156.1	1.21	5.13	1.60E-05	1.72E-04	lincRNA
CUST_34312_PI428871386	ENST00000570035.1	1.04	5.23	1.60E-05	1.73E-04	lincRNA
CUST_21645_PI428871386	ENST00000454968.1	-1.25	9.83	1.61E-05	1.73E-04	lincRNA
CUST_32883_PI428871386	ENST00000564390.1	-1.04	5.58	1.63E-05	1.76E-04	lincRNA
CUST_42102_PI428871386	ENST00000569966.1	-1.13	5.87	1.65E-05	1.78E-04	lincRNA
CUST_27551_PI428871386	ENST00000546421.1	1.04	5.82	1.65E-05	1.78E-04	antisense
A_23_P369733	RANBP10	-1.07	9.56	1.67E-05	1.79E-04	protein_coding
A_33_P3397840	WIPF3	1.35	5.68	1.67E-05	1.80E-04	protein_coding
CUST_41928_PI428871386	ENST00000417335.1	-0.94	4.70	1.68E-05	1.81E-04	lincRNA
CUST_15225_PI428871386	ENST00000418567.1	1.39	5.63	1.68E-05	1.81E-04	lincRNA
A_33_P3227666	AGGF1	-1.10	8.58	1.69E-05	1.81E-04	protein_coding
A_23_P207399	NBR1	-1.01	11.45	1.69E-05	1.81E-04	protein_coding
CUST_18811_PI428871386	ENST00000520890.1	1.55	5.43	1.69E-05	1.81E-04	antisense
CUST_10802_PI428871386	ENST00000509007.1	1.21	6.14	1.71E-05	1.83E-04	antisense
A_23_P64232	ZNF259	1.18	9.59	1.72E-05	1.84E-04	protein_coding
CUST_39656_PI428871386	ENST00000587413.1	1.27	6.36	1.72E-05	1.85E-04	antisense
A_23_P13604	PEBP1	-1.17	13.20	1.73E-05	1.85E-04	protein_coding
CUST_26886_PI428871386	ENST00000514702.1	1.89	5.32	1.73E-05	1.86E-04	antisense
A_33_P3281066	ZNF295	-1.21	9.06	1.74E-05	1.87E-04	protein_coding
A_33_P3224710	TFEC	-2.16	8.67	1.75E-05	1.87E-04	protein_coding
CUST_15441_PI428871386	ENST00000426737.1	0.84	5.30	1.75E-05	1.88E-04	antisense
A_23_P381203	KIAA0556	-0.92	6.05	1.76E-05	1.88E-04	protein_coding
CUST_11572_PI428871386	ENST00000513067.1	-1.23	5.43	1.79E-05	1.91E-04	antisense
CUST_41652_PI428871386	ENST00000567517.1	-1.03	5.44	1.79E-05	1.92E-04	antisense
A_33_P3292794	SMUG1	0.99	7.00	1.80E-05	1.93E-04	protein_coding
CUST_8340_PI428871386	ENST00000485770.1	-1.10	7.11	1.81E-05	1.93E-04	lincRNA
CUST_39676_PI428871386	ENST00000592100.1	1.45	6.78	1.81E-05	1.94E-04	lincRNA
A_33_P3575854	LOC642361	-1.03	9.77	1.81E-05	1.94E-04	lincRNA
A_24_P310894	CAPZA1	-1.08	12.23	1.81E-05	1.94E-04	protein_coding
CUST_37368_PI428871386	ENST00000582570.1	1.50	6.77	1.82E-05	1.95E-04	lincRNA
A_24_P376339	CCNL2	1.25	8.62	1.83E-05	1.95E-04	protein_coding
A_33_P3414487	WBSCR22	0.89	10.95	1.83E-05	1.95E-04	protein_coding
A_23_P254756	CD164	-1.25	10.43	1.83E-05	1.96E-04	protein_coding
A_33_P3292829	SCYL2	1.24	7.81	1.84E-05	1.96E-04	protein_coding
A_32_P331052	C20orf151	0.93	5.59	1.85E-05	1.97E-04	protein_coding
A_33_P3217700	USP9Y	-1.74	6.93	1.85E-05	1.98E-04	protein_coding

CUST_20296_PI428871386	ENST00000532846.1	1.01	5.11	1.86E-05	1.98E-04	protein_coding
CUST_6385_PI428871386	ENST00000447078.1	-0.84	4.47	1.86E-05	1.98E-04	lincRNA
A_23_P4679	ERF	1.33	7.23	1.87E-05	1.99E-04	protein_coding
CUST_7264_PI428871386	ENST00000441749.1	-1.11	6.84	1.87E-05	2.00E-04	antisense
A_33_P3239884	NCAM2	-1.39	6.69	1.88E-05	2.00E-04	protein_coding
CUST_14738_PI428871386	ENST00000453426.1	-1.29	7.76	1.88E-05	2.00E-04	lincRNA
A_24_P313262	ARPP19	-1.04	8.91	1.88E-05	2.00E-04	protein_coding
CUST_24113_PI428871386	ENST00000524885.1	1.38	5.76	1.89E-05	2.02E-04	lincRNA
CUST_26044_PI428871386	ENST00000545163.1	-0.96	4.92	1.90E-05	2.02E-04	antisense
CUST_26656_PI428871386	ENST00000547851.1	-1.42	7.74	1.90E-05	2.03E-04	lincRNA
A_33_P3251054	RNF38	-1.27	10.04	1.91E-05	2.03E-04	protein_coding
A_33_P3629247	ANKMY1	-0.86	6.85	1.92E-05	2.04E-04	protein_coding
A_33_P3211229	SLC35F2	1.23	5.69	1.92E-05	2.04E-04	protein_coding
CUST_41164_PI428871386	ENST00000435301.2	0.90	5.41	1.93E-05	2.05E-04	lincRNA
A_23_P344578	FAM154A	0.90	5.02	1.93E-05	2.06E-04	protein_coding
CUST_14960_PI428871386	ENST00000506206.1	1.91	5.29	1.95E-05	2.07E-04	lincRNA
A_33_P3396239	SNRPD3	1.13	9.94	1.95E-05	2.08E-04	protein_coding
CUST_40494_PI428871386	ENST00000411839.1	-1.94	6.97	1.96E-05	2.08E-04	lincRNA
A_23_P433016	FBLN1	-1.82	9.19	1.97E-05	2.09E-04	protein_coding
CUST_12544_PI428871386	ENST00000576302.1	-0.90	5.31	1.98E-05	2.10E-04	antisense
A_24_P288754	PIGA	-1.81	9.24	1.98E-05	2.10E-04	protein_coding
CUST_17040_PI428871386	ENST00000416366.1	0.88	4.99	1.99E-05	2.11E-04	lincRNA
CUST_29217_PI428871386	ENST00000423246.1	-0.99	5.35	1.99E-05	2.12E-04	antisense
A_33_P3746549	PLEKHG4B	1.68	6.39	2.00E-05	2.12E-04	protein_coding
CUST_7623_PI428871386	ENST00000414603.1	1.02	4.98	2.00E-05	2.12E-04	lincRNA
A_24_P185986	UPRT	-1.14	9.06	2.00E-05	2.12E-04	protein_coding
CUST_16354_PI428871386	ENST00000424460.1	1.22	4.99	2.01E-05	2.14E-04	antisense
A_23_P128396	RNF34	0.97	8.78	2.02E-05	2.14E-04	protein_coding
A_24_P336113	CABIN1	-1.02	8.58	2.02E-05	2.14E-04	protein_coding
A_23_P92672	OCLN	-1.50	7.34	2.02E-05	2.14E-04	protein_coding
CUST_31668_PI428871386	ENST00000560727.1	-1.07	5.27	2.02E-05	2.14E-04	antisense
A_23_P15108	YPEL3	-1.25	8.75	2.02E-05	2.14E-04	protein_coding
A_23_P306867	NR4A3	-1.57	5.75	2.03E-05	2.15E-04	protein_coding
CUST_31536_PI428871386	ENST00000560034.1	-0.99	5.00	2.04E-05	2.16E-04	antisense
A_23_P215675	C7orf44	1.04	8.91	2.04E-05	2.16E-04	protein_coding
CUST_8010_PI428871386	ENST00000366441.2	-1.14	5.88	2.05E-05	2.17E-04	antisense
A_23_P118122	RGS11	-1.71	7.75	2.06E-05	2.18E-04	protein_coding
A_33_P3257182	MYBPC2	1.47	5.59	2.07E-05	2.19E-04	protein_coding
A_24_P406814	FAM53B	-1.22	8.99	2.07E-05	2.19E-04	protein_coding
CUST_31666_PI428871386	ENST00000559977.1	-1.05	5.45	2.07E-05	2.19E-04	antisense
A_23_P74981	ZNF670	1.34	7.52	2.08E-05	2.20E-04	protein_coding
A_33_P3221432	ZNF284	0.96	5.57	2.08E-05	2.20E-04	protein_coding
A_23_P411188	CHRNA10	-1.15	6.19	2.10E-05	2.21E-04	protein_coding
CUST_34867_PI428871386	ENST00000573601.1	-1.33	6.19	2.10E-05	2.22E-04	antisense
CUST_18470_PI428871386	ENST00000502083.2	-1.07	6.45	2.11E-05	2.23E-04	antisense
CUST_7932_PI428871386	ENST00000414382.1	1.77	5.44	2.12E-05	2.24E-04	lincRNA
A_33_P3269453	BPTF	1.01	7.35	2.12E-05	2.24E-04	protein_coding
A_23_P2006	CNGA4	-1.06	5.25	2.12E-05	2.24E-04	protein_coding
A_23_P28772	DBNDD2	-1.44	10.11	2.13E-05	2.25E-04	protein_coding
A_24_P112032	KCNK17	-1.64	6.45	2.13E-05	2.25E-04	protein_coding
A_33_P3661631	PCBD2	1.14	7.65	2.14E-05	2.26E-04	protein_coding
CUST_41927_PI428871386	ENST00000417335.1	-0.90	4.73	2.15E-05	2.27E-04	lincRNA
A_23_P63190	NRAS	1.22	7.76	2.16E-05	2.28E-04	protein_coding
CUST_1011_PI428871386	ENST00000564479.1	-1.00	5.84	2.18E-05	2.30E-04	antisense
CUST_38102_PI428871386	ENST00000586467.1	-1.27	6.20	2.18E-05	2.30E-04	antisense
CUST_29442_PI428871386	ENST00000554730.1	0.97	5.29	2.19E-05	2.31E-04	antisense
CUST_39850_PI428871386	ENST00000597203.1	-1.02	6.29	2.19E-05	2.31E-04	antisense
CUST_17377_PI428871386	ENST00000419422.1	1.50	5.27	2.20E-05	2.32E-04	antisense
A_23_P388900	SLC22A15	-1.52	7.90	2.20E-05	2.32E-04	protein_coding
CUST_1370_PI428871386	ENST00000433521.1	-1.01	7.29	2.21E-05	2.32E-04	antisense
CUST_7138_PI428871386	ENST00000440574.1	1.19	6.79	2.21E-05	2.33E-04	antisense
A_33_P3392977	CCNDBP1	-1.21	9.59	2.22E-05	2.33E-04	protein_coding
A_33_P3366987	GPR125	1.43	6.58	2.22E-05	2.34E-04	protein_coding

A_33_P3213551	SHARPIN	1.08	11.53	2.23E-05	2.35E-04	protein_coding
A_23_P217098	VPS13A	-1.27	9.11	2.25E-05	2.36E-04	protein_coding
CUST_41352_PI428871386	ENST00000414042.1	-2.05	8.70	2.25E-05	2.37E-04	antisense
A_24_P80181	TMEM127	-1.11	10.53	2.26E-05	2.37E-04	protein_coding
CUST_23736_PI428871386	ENST00000531076.1	-1.23	5.56	2.27E-05	2.38E-04	lincRNA
A_33_P3367692	CFH	-1.61	9.18	2.28E-05	2.39E-04	protein_coding
A_23_P219060	GPSM3	-1.31	8.99	2.28E-05	2.40E-04	protein_coding
CUST_2751_PI428871386	ENST00000417644.1	0.78	4.98	2.29E-05	2.40E-04	antisense
A_24_P135748	GRTP1	1.61	6.27	2.29E-05	2.40E-04	protein_coding
CUST_4012_PI428871386	ENST00000423704.1	-1.20	8.03	2.29E-05	2.40E-04	lincRNA
A_32_P56713	BCR	-1.01	7.61	2.30E-05	2.41E-04	protein_coding
CUST_32122_PI428871386	ENST00000564194.1	1.44	6.48	2.30E-05	2.41E-04	antisense
A_23_P254944	GSTT1	-1.47	9.22	2.32E-05	2.43E-04	protein_coding
CUST_16873_PI428871386	ENST00000443162.1	1.15	10.07	2.32E-05	2.43E-04	antisense
A_33_P3311791	ZNF749	1.04	5.65	2.33E-05	2.44E-04	protein_coding
A_23_P4353	WSB1	-1.44	11.97	2.33E-05	2.44E-04	protein_coding
A_23_P64372	TCN1	2.80	5.69	2.35E-05	2.46E-04	protein_coding
A_23_P131202	HES6	1.39	6.07	2.35E-05	2.46E-04	protein_coding
CUST_18925_PI428871386	ENST00000518943.1	-0.99	5.16	2.36E-05	2.47E-04	lincRNA
A_33_P3724157	DERL3	1.24	5.71	2.37E-05	2.48E-04	protein_coding
A_33_P3216232	ITGB1BP1	-1.16	8.11	2.37E-05	2.49E-04	protein_coding
CUST_9148_PI428871386	ENST00000461943.1	-0.89	5.68	2.38E-05	2.49E-04	antisense
CUST_41893_PI428871386	ENST00000454482.2	1.06	5.53	2.38E-05	2.49E-04	lincRNA
CUST_5764_PI428871386	ENST00000419650.1	1.14	7.89	2.38E-05	2.49E-04	lincRNA
A_23_P60101	ZNF696	1.17	8.23	2.39E-05	2.50E-04	protein_coding
CUST_15912_PI428871386	ENST00000417502.1	-0.99	5.10	2.40E-05	2.51E-04	antisense
A_23_P80974	TDO2	1.60	6.49	2.41E-05	2.52E-04	protein_coding
CUST_41406_PI428871386	ENST00000567259.1	1.06	5.29	2.42E-05	2.53E-04	lincRNA
A_23_P54636	ATP6V0D1	-1.16	10.86	2.42E-05	2.53E-04	protein_coding
CUST_36382_PI428871386	ENST00000322227.2	-0.86	4.75	2.44E-05	2.55E-04	lincRNA
A_23_P392429	ZNF396	-0.93	5.11	2.45E-05	2.55E-04	protein_coding
CUST_4016_PI428871386	ENST00000438436.1	1.25	6.04	2.45E-05	2.56E-04	antisense
A_32_P22622	NOP14	1.04	6.21	2.48E-05	2.58E-04	protein_coding
A_23_P142154	GRWD1	0.96	9.52	2.48E-05	2.58E-04	protein_coding
A_23_P403335	EXPH5	-1.67	9.40	2.48E-05	2.59E-04	protein_coding
A_33_P3540143	IL17RA	-1.42	7.70	2.48E-05	2.59E-04	protein_coding
A_24_P237175	CST2	1.63	5.78	2.49E-05	2.60E-04	protein_coding
A_33_P3231252	NHLH2	-1.13	6.57	2.51E-05	2.62E-04	protein_coding
A_33_P3377209	ENSA	1.25	7.54	2.53E-05	2.64E-04	protein_coding
A_23_P67339	RCN3	1.49	7.81	2.54E-05	2.65E-04	protein_coding
A_24_P113824	TMEM50A	-1.08	9.30	2.55E-05	2.66E-04	protein_coding
A_33_P3275948	STARD3NL	-1.04	8.95	2.58E-05	2.68E-04	protein_coding
A_24_P196704	MLL5	-1.07	10.58	2.59E-05	2.70E-04	protein_coding
CUST_42739_PI428871386	ENST00000563812.1	1.15	7.51	2.59E-05	2.70E-04	antisense
A_24_P292253	ITIH5	-1.05	5.19	2.59E-05	2.70E-04	protein_coding
CUST_9094_PI428871386	ENST00000469812.1	0.93	5.02	2.61E-05	2.71E-04	lincRNA
CUST_14102_PI428871386	ENST00000520324.1	1.08	5.24	2.61E-05	2.72E-04	lincRNA
A_24_P349002	POM121	1.01	9.33	2.62E-05	2.72E-04	protein_coding
CUST_32382_PI428871386	ENST00000558980.1	-0.97	5.82	2.62E-05	2.73E-04	antisense
CUST_14899_PI428871386	ENST00000573382.1	-1.24	6.33	2.62E-05	2.73E-04	lincRNA
CUST_26198_PI428871386	ENST00000420040.2	-1.58	7.12	2.63E-05	2.73E-04	lincRNA
A_24_P241318	DCAF4	1.08	8.32	2.63E-05	2.73E-04	protein_coding
A_24_P372048	YKT6	1.13	10.15	2.64E-05	2.75E-04	protein_coding
A_33_P3871347	SNED1	-1.25	8.78	2.64E-05	2.75E-04	protein_coding
A_23_P391637	TBC1D24	-1.29	7.58	2.65E-05	2.75E-04	protein_coding
A_33_P3236065	CLEC1B	-1.07	5.26	2.66E-05	2.76E-04	protein_coding
CUST_3537_PI428871386	ENST00000412855.1	1.27	6.05	2.66E-05	2.76E-04	lincRNA
A_23_P363313	SLC16A11	-1.18	6.81	2.66E-05	2.76E-04	protein_coding
CUST_17864_PI428871386	ENST00000493248.1	-1.12	8.10	2.66E-05	2.77E-04	antisense
A_24_P102821	PTAFR	-1.64	8.75	2.67E-05	2.77E-04	protein_coding
A_33_P3234490	BOLA2B	1.28	8.26	2.68E-05	2.78E-04	protein_coding
CUST_36383_PI428871386	ENST00000322227.2	-0.94	4.77	2.68E-05	2.78E-04	lincRNA
A_23_P83094	TLE4	-1.28	8.35	2.68E-05	2.78E-04	protein_coding

A_23_P94009	NAA38	0.99	11.07	2.70E-05	2.80E-04	protein_coding
CUST_7217_PI428871386	ENST00000413311.1	-1.17	6.46	2.70E-05	2.80E-04	lincRNA
A_23_P155890	NAA11	1.17	7.64	2.70E-05	2.80E-04	protein_coding
A_23_P151710	PTGER2	-1.49	6.49	2.71E-05	2.80E-04	protein_coding
CUST_29899_PI428871386	ENST00000554221.1	1.58	5.29	2.71E-05	2.81E-04	lincRNA
CUST_17372_PI428871386	ENST00000448513.1	-1.04	4.91	2.72E-05	2.82E-04	antisense
A_23_P168828	KLF10	-1.52	9.69	2.72E-05	2.82E-04	protein_coding
CUST_26199_PI428871386	ENST00000420040.2	-1.67	7.49	2.72E-05	2.82E-04	lincRNA
CUST_26206_PI428871386	ENST00000535746.1	-1.11	6.67	2.74E-05	2.84E-04	lincRNA
CUST_20709_PI428871386	ENST00000455995.1	-1.12	5.53	2.74E-05	2.84E-04	lincRNA
CUST_7949_PI428871386	ENST00000413430.1	-1.08	5.92	2.75E-05	2.84E-04	antisense
A_23_P20384	LSM1	1.40	10.95	2.75E-05	2.84E-04	protein_coding
A_33_P3228305	ARHGAP26	-1.54	8.60	2.75E-05	2.85E-04	protein_coding
CUST_42011_PI428871386	ENST00000437426.1	-0.89	4.76	2.76E-05	2.85E-04	antisense
A_23_P356101	FBXO11	1.02	7.73	2.77E-05	2.87E-04	protein_coding
CUST_26716_PI428871386	ENST00000547395.1	0.94	5.43	2.77E-05	2.87E-04	antisense
CUST_31349_PI428871386	ENST00000558846.1	-1.20	5.56	2.77E-05	2.87E-04	lincRNA
A_23_P39088	PRMT1	1.08	10.14	2.78E-05	2.87E-04	protein_coding
A_33_P3288839	C14orf37	1.97	6.34	2.78E-05	2.87E-04	protein_coding
A_23_P422071	B3GALT4	-1.36	10.35	2.79E-05	2.88E-04	protein_coding
A_23_P74668	C1orf158	-1.03	8.06	2.82E-05	2.91E-04	protein_coding
A_23_P17053	IL36G	2.57	5.63	2.83E-05	2.92E-04	protein_coding
A_23_P255569	DUS1L	1.04	9.61	2.83E-05	2.92E-04	protein_coding
A_23_P27571	MCOLN1	-1.28	7.57	2.83E-05	2.92E-04	protein_coding
CUST_29074_PI428871386	ENST00000445737.1	1.51	6.13	2.86E-05	2.95E-04	lincRNA
CUST_15840_PI428871386	ENST00000455011.1	-1.14	5.72	2.86E-05	2.95E-04	lincRNA
CUST_13030_PI428871386	ENST00000514571.1	-1.06	5.68	2.88E-05	2.98E-04	antisense
CUST_40492_PI428871386	ENST00000447956.1	-2.05	7.39	2.89E-05	2.98E-04	lincRNA
A_33_P3338740	DHH	-1.24	5.75	2.90E-05	2.99E-04	protein_coding
A_23_P313223	C11orf84	0.96	6.04	2.90E-05	2.99E-04	protein_coding
CUST_23102_PI428871386	ENST00000423474.1	-1.26	5.23	2.91E-05	3.00E-04	antisense
CUST_26172_PI428871386	ENST00000372173.5	-1.17	6.23	2.94E-05	3.03E-04	antisense
CUST_15060_PI428871386	ENST00000418765.1	1.44	9.07	2.94E-05	3.03E-04	lincRNA
CUST_25041_PI428871386	ENST00000546324.1	-0.92	5.00	2.94E-05	3.03E-04	antisense
CUST_6922_PI428871386	ENST00000594023.1	-0.90	5.33	2.97E-05	3.06E-04	antisense
CUST_13391_PI428871386	ENST00000509993.1	-1.46	6.11	2.98E-05	3.08E-04	antisense
CUST_6548_PI428871386	ENST00000442456.1	-1.20	5.63	3.00E-05	3.09E-04	lincRNA
CUST_3533_PI428871386	ENST00000424332.1	-1.24	7.19	3.01E-05	3.10E-04	antisense
CUST_21480_PI428871386	ENST00000414544.1	-0.88	5.07	3.01E-05	3.10E-04	antisense
A_23_P252362	MRPS30	1.14	7.80	3.02E-05	3.10E-04	protein_coding
A_33_P3226080	C17orf87	-1.61	7.27	3.02E-05	3.11E-04	protein_coding
CUST_20723_PI428871386	ENST00000592873.1	-0.85	4.90	3.02E-05	3.11E-04	lincRNA
A_24_P54174	TNFRSF1B	-1.04	7.05	3.02E-05	3.11E-04	protein_coding
CUST_37527_PI428871386	ENST00000583316.1	2.06	5.50	3.02E-05	3.11E-04	lincRNA
CUST_19393_PI428871386	ENST00000524052.1	-1.07	5.54	3.03E-05	3.11E-04	antisense
A_23_P24922	LIPT2	1.32	7.41	3.03E-05	3.12E-04	protein_coding
A_24_P356338	GABARAPL2	-1.11	10.25	3.03E-05	3.12E-04	protein_coding
A_24_P37540	ARPC4-TTLL3	-1.01	7.01	3.04E-05	3.12E-04	protein_coding
CUST_7948_PI428871386	ENST00000413430.1	-1.00	5.81	3.04E-05	3.12E-04	antisense
CUST_21412_PI428871386	ENST00000448674.1	-0.84	6.32	3.05E-05	3.13E-04	lincRNA
CUST_40493_PI428871386	ENST00000411839.1	-1.90	7.01	3.05E-05	3.13E-04	lincRNA
A_33_P3305158	ZNF621	-0.90	9.12	3.06E-05	3.14E-04	protein_coding
CUST_26411_PI428871386	ENST00000446891.2	-1.35	6.52	3.07E-05	3.15E-04	lincRNA
CUST_6093_PI428871386	ENST00000375987.3	-1.18	6.32	3.07E-05	3.15E-04	lincRNA
A_23_P256581	PRDM13	2.78	5.32	3.08E-05	3.16E-04	protein_coding
A_32_P205624	SHC2	-1.54	8.30	3.11E-05	3.19E-04	protein_coding
CUST_17997_PI428871386	ENST00000485974.1	0.86	5.24	3.11E-05	3.19E-04	antisense
CUST_35007_PI428871386	ENST00000497885.1	1.03	5.69	3.11E-05	3.19E-04	antisense
CUST_24548_PI428871386	ENST00000534162.1	0.93	5.42	3.12E-05	3.20E-04	antisense
A_33_P3278590	AKAP14	-1.24	5.53	3.14E-05	3.22E-04	protein_coding
A_33_P3309804	EID1	-1.07	6.27	3.14E-05	3.22E-04	protein_coding
CUST_25871_PI428871386	ENST00000416553.1	-1.27	5.33	3.14E-05	3.22E-04	lincRNA
A_23_P115124	TRIM11	0.98	7.78	3.16E-05	3.23E-04	protein_coding

A_23_P208482	CLEC4M	-1.69	7.18	3.17E-05	3.25E-04	protein_coding
CUST_10991_PI428871386	ENST00000506527.1	1.14	5.86	3.18E-05	3.25E-04	antisense
A_33_P3335920	SYNE1	-0.93	9.25	3.18E-05	3.25E-04	protein_coding
A_23_P419641	DLL4	-1.20	5.66	3.19E-05	3.27E-04	protein_coding
A_33_P3671378	CERCAM	0.96	7.29	3.20E-05	3.27E-04	protein_coding
A_24_P191312	SLC1A4	1.17	6.09	3.22E-05	3.29E-04	protein_coding
CUST_43791_PI428871386	ENST00000445330.1	1.89	5.09	3.25E-05	3.33E-04	antisense
CUST_37222_PI428871386	ENST00000583521.1	0.98	5.17	3.25E-05	3.33E-04	antisense
CUST_13535_PI428871386	ENST00000422204.1	-0.84	4.91	3.25E-05	3.33E-04	antisense
CUST_22858_PI428871386	ENST00000526759.1	-0.96	4.94	3.26E-05	3.34E-04	lincRNA
CUST_10526_PI428871386	ENST00000510637.1	-1.01	6.48	3.27E-05	3.34E-04	antisense
A_23_P55998	SLC1A5	1.34	8.14	3.27E-05	3.34E-04	protein_coding
CUST_38104_PI428871386	ENST00000589662.1	1.58	5.25	3.27E-05	3.34E-04	lincRNA
CUST_26739_PI428871386	ENST00000553259.1	-1.03	7.21	3.28E-05	3.35E-04	antisense
A_23_P321855	ARHGEF7	-1.08	8.81	3.28E-05	3.35E-04	protein_coding
A_23_P83931	NET1	1.32	10.76	3.29E-05	3.35E-04	protein_coding
A_33_P3519250	NWD1	-1.22	5.41	3.30E-05	3.37E-04	protein_coding
A_23_P69586	FAT1	1.34	9.34	3.32E-05	3.39E-04	protein_coding
A_24_P354724	TAGAP	-1.71	7.81	3.33E-05	3.40E-04	protein_coding
CUST_31671_PI428871386	ENST00000559173.1	-1.01	5.50	3.35E-05	3.42E-04	antisense
A_23_P69810	AGPAT9	-1.74	8.30	3.36E-05	3.42E-04	protein_coding
A_33_P3286372	C2orf48	1.07	5.30	3.36E-05	3.42E-04	lincRNA
CUST_11806_PI428871386	ENST00000508968.1	0.84	5.58	3.36E-05	3.42E-04	antisense
CUST_37221_PI428871386	ENST00000583492.1	1.07	6.24	3.37E-05	3.43E-04	antisense
A_24_P120346	NIT2	1.17	11.27	3.37E-05	3.44E-04	protein_coding
CUST_14360_PI428871386	ENST00000445000.1	-0.84	5.21	3.38E-05	3.44E-04	lincRNA
CUST_25600_PI428871386	ENST00000530198.1	-0.88	4.74	3.39E-05	3.45E-04	antisense
CUST_197_PI428871386	ENST00000434150.1	1.05	5.71	3.40E-05	3.46E-04	lincRNA
CUST_8667_PI428871386	ENST00000563632.1	-1.35	7.45	3.43E-05	3.49E-04	antisense
CUST_21396_PI428871386	ENST00000427548.1	1.41	6.71	3.44E-05	3.50E-04	lincRNA
CUST_6248_PI428871386	ENST00000595109.1	-0.93	5.49	3.44E-05	3.50E-04	antisense
A_23_P88134	CINP	1.08	7.54	3.44E-05	3.50E-04	protein_coding
CUST_27227_PI428871386	ENST00000426250.2	-1.07	5.19	3.45E-05	3.51E-04	antisense
CUST_7315_PI428871386	ENST00000587099.1	1.95	5.93	3.45E-05	3.51E-04	lincRNA
CUST_38410_PI428871386	ENST00000583629.1	-0.89	4.78	3.46E-05	3.51E-04	lincRNA
A_23_P86470	CH25H	-2.61	10.19	3.46E-05	3.51E-04	protein_coding
CUST_3297_PI428871386	ENST00000445272.1	-1.35	6.33	3.47E-05	3.53E-04	antisense
CUST_37741_PI428871386	ENST00000583490.1	-1.01	5.01	3.48E-05	3.54E-04	lincRNA
CUST_23880_PI428871386	ENST00000415809.1	1.12	5.73	3.51E-05	3.56E-04	antisense
CUST_34595_PI428871386	ENST00000566773.1	1.05	7.53	3.51E-05	3.57E-04	antisense
A_23_P70571	SLC39A7	1.16	10.17	3.52E-05	3.58E-04	protein_coding
CUST_21985_PI428871386	ENST00000478294.1	1.79	5.12	3.52E-05	3.58E-04	lincRNA
CUST_16450_PI428871386	ENST00000451792.1	-1.66	6.63	3.53E-05	3.58E-04	lincRNA
A_24_P139094	SH3GL1	0.90	10.72	3.53E-05	3.58E-04	protein_coding
CUST_9780_PI428871386	ENST00000414120.1	1.10	5.01	3.54E-05	3.59E-04	lincRNA
A_33_P3799936	ARHGEF10L	-1.39	11.36	3.54E-05	3.59E-04	protein_coding
CUST_43149_PI428871386	ENST00000412485.1	-1.57	6.29	3.56E-05	3.61E-04	lincRNA
A_24_P92472	CFI	-1.50	7.95	3.56E-05	3.61E-04	protein_coding
CUST_18813_PI428871386	ENST00000518994.1	1.49	5.45	3.57E-05	3.62E-04	antisense
A_23_P501080	ZNF92	1.13	8.82	3.58E-05	3.62E-04	protein_coding
A_23_P48029	CLEC4A	-1.77	8.14	3.58E-05	3.63E-04	protein_coding
A_33_P3420224	ENTPD8	1.83	6.26	3.59E-05	3.63E-04	protein_coding
A_33_P3351554	ETNK2	1.32	8.30	3.60E-05	3.65E-04	protein_coding
CUST_23881_PI428871386	ENST00000415809.1	1.09	5.81	3.61E-05	3.65E-04	antisense
CUST_41290_PI428871386	ENST00000424205.1	1.70	5.56	3.63E-05	3.68E-04	lincRNA
A_23_P137434	RNF11	-1.07	11.63	3.65E-05	3.69E-04	protein_coding
CUST_25863_PI428871386	ENST00000525716.2	-0.86	5.64	3.65E-05	3.70E-04	lincRNA
A_23_P160466	SLC19A2	-1.48	9.24	3.68E-05	3.72E-04	protein_coding
A_23_P149678	PIAS3	1.24	10.09	3.69E-05	3.73E-04	protein_coding
A_23_P69988	RARS	0.97	11.85	3.69E-05	3.73E-04	protein_coding
A_24_P365015	HOXB13	2.13	5.38	3.69E-05	3.74E-04	protein_coding
CUST_17879_PI428871386	ENST00000498397.1	-1.18	6.04	3.69E-05	3.74E-04	antisense
CUST_42057_PI428871386	ENST00000411694.1	-1.33	7.20	3.70E-05	3.74E-04	lincRNA

CUST_12159_PI428871386	ENST00000502209.2	1.04	4.94	3.70E-05	3.74E-04	lincRNA
A_23_P413285	CC2D2A	-1.01	6.40	3.71E-05	3.75E-04	protein_coding
CUST_11934_PI428871386	ENST00000511785.1	-0.87	4.87	3.71E-05	3.75E-04	lincRNA
A_33_P3223990	TPM3	-1.19	6.67	3.72E-05	3.76E-04	protein_coding
CUST_26887_PI428871386	ENST00000514702.1	1.89	5.45	3.78E-05	3.82E-04	antisense
A_24_P63537	ERAP1	-0.90	5.48	3.81E-05	3.85E-04	protein_coding
CUST_26798_PI428871386	ENST00000564363.1	-1.13	5.81	3.81E-05	3.85E-04	lincRNA
CUST_38877_PI428871386	ENST00000590065.1	-0.92	6.84	3.82E-05	3.85E-04	lincRNA
A_32_P199301	TFDP1	1.05	7.77	3.82E-05	3.85E-04	protein_coding
CUST_18309_PI428871386	ENST00000522026.1	0.77	4.91	3.83E-05	3.86E-04	antisense
CUST_6128_PI428871386	ENST00000419784.1	-0.92	4.96	3.84E-05	3.87E-04	lincRNA
A_33_P3359183	AWAT2	-1.35	5.22	3.85E-05	3.89E-04	protein_coding
A_23_P137865	MTX1	1.15	9.27	3.86E-05	3.89E-04	protein_coding
A_33_P3272160	REXO4	1.04	10.86	3.86E-05	3.89E-04	protein_coding
A_33_P3360157	OR5AS1	-0.78	5.14	3.88E-05	3.91E-04	protein_coding
A_23_P784	LZIC	1.07	10.61	3.88E-05	3.91E-04	protein_coding
CUST_15564_PI428871386	ENST00000517965.1	1.06	6.65	3.88E-05	3.91E-04	antisense
CUST_26413_PI428871386	ENST00000536729.1	-1.36	6.65	3.88E-05	3.91E-04	lincRNA
A_24_P291231	PER3	-1.30	6.26	3.89E-05	3.92E-04	protein_coding
CUST_25601_PI428871386	ENST00000530198.1	-0.89	4.94	3.90E-05	3.93E-04	antisense
CUST_15066_PI428871386	ENST00000422882.1	1.48	8.93	3.91E-05	3.94E-04	lincRNA
A_23_P82950	PCM1	-1.04	11.14	3.93E-05	3.95E-04	protein_coding
A_23_P79911	PSMF1	-1.08	9.75	3.96E-05	3.98E-04	protein_coding
A_32_P213330	RGNEF	-1.28	6.47	3.96E-05	3.98E-04	protein_coding
A_23_P46844	TRIM8	-1.21	10.74	3.99E-05	4.01E-04	protein_coding
A_23_P388168	RAB3B	1.56	5.12	4.00E-05	4.02E-04	protein_coding
CUST_275_PI428871386	ENST00000452079.1	-1.09	6.61	4.00E-05	4.02E-04	antisense
CUST_29475_PI428871386	ENST00000553985.1	-1.01	6.93	4.01E-05	4.03E-04	antisense
A_24_P42436	ARHGAP32	1.23	6.37	4.03E-05	4.05E-04	protein_coding
CUST_15643_PI428871386	ENST00000430296.1	-1.03	5.40	4.03E-05	4.05E-04	lincRNA
CUST_13494_PI428871386	ENST00000501173.2	-0.99	8.04	4.04E-05	4.05E-04	lincRNA
CUST_30201_PI428871386	ENST00000556578.1	-1.04	5.90	4.04E-05	4.05E-04	antisense
A_33_P3277890	ENST00000399966	1.61	5.35	4.05E-05	4.07E-04	antisense
CUST_41898_PI428871386	ENST00000440629.1	1.04	5.60	4.05E-05	4.07E-04	lincRNA
CUST_25169_PI428871386	ENST00000532831.1	0.98	7.11	4.05E-05	4.07E-04	antisense
CUST_5590_PI428871386	ENST00000596356.1	-0.93	4.97	4.06E-05	4.07E-04	antisense
CUST_5084_PI428871386	ENST00000439964.1	1.02	5.15	4.06E-05	4.07E-04	lincRNA
A_23_P170186	OPLAH	1.60	9.10	4.08E-05	4.09E-04	protein_coding
A_32_P332551	C13orf30	-1.08	5.34	4.09E-05	4.10E-04	protein_coding
A_33_P3278058	PIH1D2	-1.29	6.63	4.09E-05	4.10E-04	protein_coding
CUST_8941_PI428871386	ENST00000470236.1	1.24	7.10	4.10E-05	4.10E-04	antisense
CUST_32548_PI428871386	ENST00000555864.1	1.89	5.29	4.10E-05	4.11E-04	lincRNA
A_23_P126363	ADAM30	0.88	5.06	4.11E-05	4.11E-04	protein_coding
CUST_14977_PI428871386	ENST00000431554.2	1.30	5.13	4.12E-05	4.13E-04	lincRNA
CUST_34315_PI428871386	ENST00000563823.2	1.02	5.16	4.15E-05	4.15E-04	lincRNA
A_24_P302802	PCCB	1.45	7.73	4.16E-05	4.16E-04	protein_coding
A_33_P3221498	STK31	1.39	5.61	4.20E-05	4.21E-04	protein_coding
CUST_19879_PI428871386	ENST00000530778.1	1.09	6.42	4.21E-05	4.21E-04	lincRNA
A_23_P420610	FCHO2	-1.14	8.16	4.22E-05	4.22E-04	protein_coding
CUST_26870_PI428871386	ENST00000424518.1	-0.98	9.28	4.22E-05	4.22E-04	antisense
CUST_17863_PI428871386	ENST00000493248.1	-1.05	7.90	4.27E-05	4.27E-04	antisense
CUST_18891_PI428871386	ENST00000521930.1	0.80	4.97	4.28E-05	4.27E-04	lincRNA
CUST_18897_PI428871386	ENST00000522711.1	-1.12	5.12	4.30E-05	4.29E-04	lincRNA
A_33_P3668839	LOC644656	1.20	7.00	4.30E-05	4.30E-04	antisense
CUST_15642_PI428871386	ENST00000442449.1	-1.17	5.54	4.31E-05	4.30E-04	antisense
CUST_34850_PI428871386	ENST00000565635.1	-1.03	7.62	4.32E-05	4.31E-04	lincRNA
CUST_2775_PI428871386	ENST00000420691.1	1.93	6.32	4.33E-05	4.32E-04	lincRNA
CUST_20732_PI428871386	ENST00000591605.1	-1.03	5.20	4.35E-05	4.34E-04	lincRNA
A_32_P174083	CYCS	-1.14	7.93	4.35E-05	4.34E-04	protein_coding
A_23_P74112	IL28RA	-1.25	8.07	4.37E-05	4.36E-04	protein_coding
A_23_P115608	ARHGAP21	-1.13	7.99	4.38E-05	4.36E-04	protein_coding
CUST_18371_PI428871386	ENST00000517454.1	0.87	4.99	4.38E-05	4.37E-04	antisense
CUST_7298_PI428871386	ENST00000597288.1	1.04	5.15	4.40E-05	4.38E-04	lincRNA

CUST_26046_PI428871386	ENST00000543206.1	-1.68	6.26	4.40E-05	4.39E-04	lincRNA
CUST_3582_PI428871386	ENST00000412647.1	-0.95	4.78	4.41E-05	4.40E-04	lincRNA
A_24_P236799	RAB31	-1.60	11.09	4.45E-05	4.44E-04	protein_coding
A_33_P3395605	TMEM119	-1.61	9.27	4.47E-05	4.45E-04	protein_coding
A_33_P3410459	SCARB2	-1.23	13.43	4.48E-05	4.46E-04	protein_coding
A_24_P76879	TSPYL1	-1.00	10.28	4.49E-05	4.48E-04	protein_coding
CUST_14125_PI428871386	ENST00000515513.1	-0.95	5.97	4.50E-05	4.48E-04	lincRNA
CUST_26412_PI428871386	ENST00000536729.1	-1.37	6.75	4.51E-05	4.49E-04	lincRNA
A_23_P46238	CELA2A	-1.24	5.87	4.52E-05	4.50E-04	protein_coding
CUST_15291_PI428871386	ENST00000433843.1	-1.28	10.16	4.52E-05	4.50E-04	lincRNA
A_23_P210690	TRIB3	1.58	11.10	4.54E-05	4.51E-04	protein_coding
A_33_P3334548	BZW1	1.32	9.54	4.55E-05	4.52E-04	protein_coding
CUST_27339_PI428871386	ENST00000548359.1	-0.87	4.86	4.57E-05	4.55E-04	lincRNA
CUST_37038_PI428871386	ENST00000581657.1	-1.12	5.69	4.57E-05	4.55E-04	lincRNA
CUST_3296_PI428871386	ENST00000445272.1	-1.30	6.49	4.58E-05	4.56E-04	antisense
A_33_P3409077	C6orf170	0.89	5.53	4.61E-05	4.58E-04	protein_coding
A_33_P3232798	RAB11FIP1	-1.63	9.34	4.63E-05	4.60E-04	protein_coding
CUST_11178_PI428871386	ENST00000509834.2	-1.17	6.03	4.64E-05	4.61E-04	lincRNA
CUST_39251_PI428871386	ENST00000593324.1	-0.99	4.98	4.65E-05	4.62E-04	lincRNA
A_23_P14072	KRT8	1.44	10.97	4.66E-05	4.63E-04	protein_coding
A_23_P79927	NOP56	1.11	9.53	4.67E-05	4.64E-04	protein_coding
A_33_P3287967	ANK2	-1.18	5.44	4.70E-05	4.66E-04	protein_coding
A_23_P314120	CHKB	-1.00	8.80	4.71E-05	4.67E-04	protein_coding
CUST_31387_PI428871386	ENST00000560415.1	-1.21	10.08	4.72E-05	4.69E-04	antisense
CUST_32123_PI428871386	ENST00000564194.1	1.35	6.52	4.73E-05	4.69E-04	antisense
A_23_P28815	CYP24A1	2.17	5.54	4.74E-05	4.70E-04	protein_coding
A_23_P202708	MADD	-0.99	8.86	4.74E-05	4.70E-04	protein_coding
A_23_P151059	FAM90A1	1.57	6.02	4.75E-05	4.71E-04	protein_coding
CUST_18810_PI428871386	ENST00000521802.1	1.47	5.46	4.77E-05	4.72E-04	antisense
CUST_22059_PI428871386	ENST00000458168.1	0.83	4.77	4.78E-05	4.74E-04	lincRNA
A_33_P3262191	CPNE7	2.11	8.86	4.80E-05	4.76E-04	protein_coding
A_33_P3363260	PGM2L1	1.63	8.90	4.80E-05	4.76E-04	protein_coding
A_23_P67529	KCNN4	1.83	8.92	4.82E-05	4.77E-04	protein_coding
A_33_P3259865	C1orf220	1.13	5.63	4.83E-05	4.79E-04	lincRNA
A_33_P3216869	CRABP1	1.50	6.43	4.85E-05	4.80E-04	protein_coding
A_33_P3283044	CCDC165	1.51	5.74	4.86E-05	4.81E-04	protein_coding
CUST_20426_PI428871386	ENST00000451142.1	-0.99	10.87	4.86E-05	4.81E-04	lincRNA
CUST_8235_PI428871386	ENST00000294241.6	-0.88	9.92	4.87E-05	4.82E-04	protein_coding
CUST_17130_PI428871386	ENST00000440088.1	-1.20	6.47	4.87E-05	4.82E-04	lincRNA
CUST_21633_PI428871386	ENST00000444125.1	1.41	9.06	4.90E-05	4.85E-04	lincRNA
A_23_P154806	EPB41L1	-1.29	10.89	4.91E-05	4.85E-04	protein_coding
CUST_21984_PI428871386	ENST00000442008.2	1.38	6.48	4.91E-05	4.85E-04	antisense
CUST_13580_PI428871386	ENST00000507035.1	1.39	5.37	4.92E-05	4.87E-04	lincRNA
A_24_P151582	TEF	-1.18	7.10	4.92E-05	4.87E-04	protein_coding
A_24_P362904	PFKFB4	1.31	7.82	4.93E-05	4.87E-04	protein_coding
A_24_P48177	ST3GAL2	-0.92	8.35	4.94E-05	4.88E-04	protein_coding
A_33_P3209457	PRPS1	0.78	5.02	4.94E-05	4.88E-04	protein_coding
A_33_P3394040	ATL2	1.33	8.49	4.94E-05	4.88E-04	protein_coding
A_33_P3419938	VHL	-1.02	13.17	4.94E-05	4.88E-04	protein_coding
A_24_P23245	NDUFA6	1.00	11.60	4.95E-05	4.89E-04	protein_coding
CUST_12083_PI428871386	ENST00000504494.1	0.87	4.93	4.96E-05	4.90E-04	lincRNA
CUST_16029_PI428871386	ENST00000430078.1	0.93	5.46	5.00E-05	4.93E-04	lincRNA
CUST_21605_PI428871386	ENST00000443631.1	1.41	7.88	5.05E-05	4.98E-04	antisense
A_23_P41487	TBC1D9	-1.29	9.19	5.06E-05	4.99E-04	protein_coding
A_23_P335495	ANO7	1.13	5.81	5.07E-05	5.00E-04	protein_coding
A_33_P3306964	PPP1R2	1.11	8.82	5.09E-05	5.02E-04	protein_coding
CUST_36572_PI428871386	ENST00000579859.1	-1.23	7.39	5.09E-05	5.02E-04	antisense
A_32_P452655	LGALS9C	-1.55	12.88	5.10E-05	5.02E-04	protein_coding
A_23_P86975	CARD18	1.82	5.00	5.10E-05	5.02E-04	protein_coding
A_33_P3393694	GLTSCR2	-1.02	13.95	5.12E-05	5.04E-04	protein_coding
A_33_P3211666	IL18R1	-1.66	6.72	5.13E-05	5.05E-04	protein_coding
CUST_31394_PI428871386	ENST00000558601.1	1.44	7.11	5.14E-05	5.06E-04	antisense
CUST_26084_PI428871386	ENST00000539206.1	-0.87	4.72	5.17E-05	5.09E-04	lincRNA

CUST_20200_PI428871386	ENST00000523031.1	1.35	6.44	5.18E-05	5.10E-04	antisense
A_23_P134614	COG5	0.92	8.59	5.21E-05	5.13E-04	protein_coding
CUST_22011_PI428871386	ENST00000445427.1	-1.36	6.81	5.21E-05	5.13E-04	lincRNA
A_32_P179676	TOB2	-0.99	9.16	5.22E-05	5.14E-04	protein_coding
A_23_P137097	SLC16A2	-1.54	7.95	5.23E-05	5.15E-04	protein_coding
A_33_P3243093	RGS5	-1.79	9.55	5.24E-05	5.15E-04	protein_coding
A_33_P3211384	KIAA1683	-0.83	5.54	5.25E-05	5.16E-04	protein_coding
A_32_P38623	PPP1R9A	-1.85	8.18	5.26E-05	5.17E-04	protein_coding
CUST_239_PI428871386	ENST00000432521.2	-1.00	5.94	5.26E-05	5.17E-04	antisense
CUST_6569_PI428871386	ENST00000458314.1	1.24	6.77	5.30E-05	5.21E-04	antisense
A_33_P3296119	CCDC169	1.33	5.18	5.31E-05	5.21E-04	protein_coding
CUST_20831_PI428871386	ENST00000476224.1	-1.11	7.60	5.31E-05	5.22E-04	lincRNA
CUST_9720_PI428871386	ENST00000433105.1	0.93	5.47	5.33E-05	5.24E-04	antisense
A_24_P922631	C5orf58	1.15	7.07	5.34E-05	5.24E-04	protein_coding
CUST_11129_PI428871386	ENST00000511064.1	-0.85	5.17	5.34E-05	5.24E-04	antisense
CUST_22732_PI428871386	ENST00000428753.1	1.10	6.77	5.35E-05	5.25E-04	antisense
CUST_43452_PI428871386	ENST00000429124.1	-1.08	9.68	5.36E-05	5.25E-04	lincRNA
CUST_10815_PI428871386	ENST00000508406.1	1.33	4.96	5.36E-05	5.26E-04	lincRNA
CUST_18972_PI428871386	ENST00000518750.1	0.83	4.98	5.36E-05	5.26E-04	lincRNA
A_23_P203891	NCOR2	-1.26	13.47	5.36E-05	5.26E-04	protein_coding
A_23_P28434	VAMP8	-1.24	13.89	5.37E-05	5.26E-04	protein_coding
A_23_P379327	FAM63B	-1.05	8.70	5.37E-05	5.27E-04	protein_coding
CUST_13772_PI428871386	ENST00000519898.1	-1.05	5.84	5.38E-05	5.27E-04	lincRNA
CUST_1540_PI428871386	ENST00000438777.1	-1.05	6.78	5.38E-05	5.27E-04	lincRNA
A_23_P364837	VPS13D	-0.90	6.32	5.40E-05	5.29E-04	protein_coding
A_33_P3412538	ANKRD17	-0.90	9.39	5.41E-05	5.30E-04	protein_coding
CUST_42469_PI428871386	ENST00000432032.1	-1.04	5.76	5.43E-05	5.32E-04	antisense
A_23_P259127	ESRP1	1.65	7.96	5.44E-05	5.32E-04	protein_coding
A_33_P3243168	MZF1	-1.21	10.20	5.44E-05	5.32E-04	protein_coding
CUST_20724_PI428871386	ENST00000592873.1	-0.88	4.96	5.45E-05	5.34E-04	lincRNA
CUST_5288_PI428871386	ENST00000602091.1	-0.85	5.28	5.48E-05	5.36E-04	antisense
CUST_9781_PI428871386	ENST00000414120.1	1.39	5.16	5.48E-05	5.36E-04	lincRNA
A_23_P116614	ME3	-1.64	9.21	5.50E-05	5.38E-04	protein_coding
CUST_16578_PI428871386	ENST00000522193.1	-1.09	5.90	5.50E-05	5.38E-04	antisense
A_33_P3268649	SLC2A11	1.28	6.22	5.50E-05	5.38E-04	protein_coding
CUST_36553_PI428871386	ENST00000579527.1	-1.30	7.99	5.50E-05	5.38E-04	antisense
CUST_23909_PI428871386	ENST00000600308.1	-1.14	6.20	5.51E-05	5.39E-04	lincRNA
CUST_8836_PI428871386	ENST00000488425.1	-1.00	4.90	5.53E-05	5.40E-04	lincRNA
CUST_24991_PI428871386	ENST00000524619.1	1.09	5.43	5.54E-05	5.41E-04	antisense
CUST_41179_PI428871386	ENST00000421019.1	-0.86	5.01	5.58E-05	5.45E-04	lincRNA
A_24_P32935	FOLR2	-1.60	7.46	5.59E-05	5.46E-04	protein_coding
A_23_P405148	MFF	0.98	12.24	5.59E-05	5.46E-04	protein_coding
A_33_P3782469	PYGO1	-1.27	6.88	5.60E-05	5.46E-04	protein_coding
A_23_P217114	ALAD	-1.11	7.54	5.60E-05	5.47E-04	protein_coding
CUST_7845_PI428871386	ENST00000414198.1	1.12	5.66	5.61E-05	5.47E-04	antisense
CUST_26294_PI428871386	ENST00000545914.1	1.37	6.47	5.61E-05	5.47E-04	antisense
A_23_P32064	NELF	1.20	6.86	5.63E-05	5.49E-04	protein_coding
A_24_P338145	STOML2	1.35	8.97	5.65E-05	5.51E-04	protein_coding
CUST_4013_PI428871386	ENST00000423704.1	-1.19	8.12	5.65E-05	5.51E-04	lincRNA
CUST_26197_PI428871386	ENST00000539348.1	-1.65	7.51	5.69E-05	5.55E-04	lincRNA
A_32_P177040	SPDYE5	-0.96	5.78	5.72E-05	5.58E-04	protein_coding
A_23_P145904	H2AFV	1.07	12.77	5.72E-05	5.58E-04	protein_coding
CUST_16611_PI428871386	ENST00000519935.1	1.28	5.61	5.73E-05	5.59E-04	antisense
A_23_P89780	LAMA3	-1.97	10.08	5.75E-05	5.60E-04	protein_coding
CUST_9191_PI428871386	ENST00000491862.1	-1.92	8.05	5.75E-05	5.60E-04	antisense
A_23_P317347	ESCO1	1.08	6.32	5.76E-05	5.61E-04	protein_coding
CUST_31309_PI428871386	ENST00000560819.1	-0.85	5.20	5.76E-05	5.61E-04	lincRNA
CUST_40938_PI428871386	ENST00000435366.1	-1.29	11.41	5.76E-05	5.61E-04	antisense
A_33_P3263841	RCHY1	-0.94	6.79	5.77E-05	5.62E-04	protein_coding
CUST_27648_PI428871386	ENST00000478808.2	-1.88	8.77	5.79E-05	5.63E-04	antisense
A_23_P364437	CDH23	-1.09	6.14	5.83E-05	5.67E-04	protein_coding
CUST_15076_PI428871386	ENST00000429530.1	1.67	5.28	5.84E-05	5.68E-04	antisense
A_23_P328621	UBQLNL	-1.12	6.14	5.84E-05	5.69E-04	protein_coding

A_23_P132294	GGA1	-0.89	11.79	5.85E-05	5.69E-04	protein_coding
CUST_19723_PI428871386	ENST00000577661.1	1.24	5.35	5.85E-05	5.69E-04	lincRNA
A_23_P74278	PDE4B	-1.79	9.47	5.86E-05	5.70E-04	protein_coding
CUST_22418_PI428871386	ENST00000421320.1	-0.84	5.14	5.86E-05	5.70E-04	lincRNA
A_32_P60223	ING5	-1.29	7.89	5.87E-05	5.71E-04	protein_coding
CUST_21606_PI428871386	ENST00000443631.1	1.44	8.65	5.88E-05	5.71E-04	antisense
CUST_41289_PI428871386	ENST00000424205.1	1.81	5.51	5.88E-05	5.71E-04	lincRNA
A_24_P637982	C1orf122	1.18	10.97	5.89E-05	5.72E-04	protein_coding
CUST_18961_PI428871386	ENST00000520881.1	-1.24	5.48	5.89E-05	5.72E-04	lincRNA
A_24_P332971	RPL7L1	1.04	9.83	5.91E-05	5.74E-04	protein_coding
A_23_P217737	ATP7A	-0.96	6.90	5.94E-05	5.77E-04	protein_coding
CUST_25765_PI428871386	ENST00000526777.1	-0.89	5.01	5.94E-05	5.77E-04	antisense
CUST_39430_PI428871386	ENST00000593063.1	0.84	4.96	5.94E-05	5.77E-04	lincRNA
CUST_12903_PI428871386	ENST00000446516.2	0.91	4.75	5.95E-05	5.77E-04	lincRNA
CUST_19706_PI428871386	ENST00000524045.1	2.03	5.47	5.95E-05	5.78E-04	antisense
A_33_P3222917	CD276	1.21	12.12	5.97E-05	5.79E-04	protein_coding
CUST_16610_PI428871386	ENST00000519935.1	1.35	5.59	5.97E-05	5.79E-04	antisense
A_23_P201079	PRDM2	-1.08	7.02	5.99E-05	5.81E-04	protein_coding
CUST_14897_PI428871386	ENST00000455005.1	0.93	5.14	6.00E-05	5.81E-04	lincRNA
A_24_P366859	USHBP1	-0.87	5.50	6.01E-05	5.83E-04	protein_coding
A_23_P169409	C9orf23	1.17	11.54	6.03E-05	5.84E-04	protein_coding
A_23_P422212	SLC35F3	2.43	6.97	6.03E-05	5.84E-04	protein_coding
CUST_16618_PI428871386	ENST00000522674.1	2.26	5.38	6.04E-05	5.85E-04	antisense
A_33_P3352019	SCARA3	-1.51	7.48	6.06E-05	5.87E-04	protein_coding
A_24_P342591	RERE	-0.88	6.97	6.08E-05	5.89E-04	protein_coding
A_23_P15876	ALPK2	1.30	5.06	6.09E-05	5.89E-04	protein_coding
A_23_P154771	DUSP15	0.80	9.38	6.09E-05	5.89E-04	protein_coding
CUST_38574_PI428871386	ENST00000586395.1	0.77	4.91	6.09E-05	5.89E-04	antisense
CUST_7940_PI428871386	ENST00000437506.1	0.92	5.33	6.11E-05	5.91E-04	lincRNA
A_32_P50522	FKBP1A	-1.01	10.14	6.13E-05	5.94E-04	protein_coding
A_23_P255257	DCAF12	0.97	8.15	6.14E-05	5.94E-04	protein_coding
A_23_P371266	DNM3	-1.83	7.37	6.14E-05	5.94E-04	protein_coding
A_23_P140928	TMC7	-1.06	5.79	6.16E-05	5.96E-04	protein_coding
A_33_P3400699	SLC26A5	-0.93	6.13	6.16E-05	5.96E-04	protein_coding
CUST_8266_PI428871386	ENST00000470427.1	-0.92	4.97	6.17E-05	5.97E-04	antisense
CUST_28355_PI428871386	ENST00000563843.1	-1.06	6.70	6.19E-05	5.99E-04	antisense
A_23_P352799	NPW	1.58	5.44	6.21E-05	6.00E-04	protein_coding
CUST_34735_PI428871386	ENST00000562705.1	1.07	5.26	6.21E-05	6.00E-04	lincRNA
A_33_P3330549	SLC44A2	-1.38	9.35	6.23E-05	6.02E-04	protein_coding
CUST_29603_PI428871386	ENST00000550118.1	0.92	4.80	6.23E-05	6.02E-04	antisense
CUST_41695_PI428871386	ENST00000433344.1	-0.83	6.77	6.27E-05	6.05E-04	lincRNA
A_33_P3222069	SPHK1	1.11	10.83	6.29E-05	6.07E-04	protein_coding
CUST_42275_PI428871386	ENST00000431090.1	1.14	5.66	6.30E-05	6.08E-04	antisense
A_23_P108708	RNF181	0.97	11.53	6.31E-05	6.09E-04	protein_coding
A_33_P3839760	C19orf24	0.95	11.91	6.31E-05	6.09E-04	protein_coding
CUST_21634_PI428871386	ENST00000444125.1	1.39	8.96	6.32E-05	6.10E-04	lincRNA
A_24_P113287	ZNF229	0.83	4.76	6.40E-05	6.17E-04	protein_coding
CUST_6410_PI428871386	ENST00000447019.1	1.06	5.87	6.40E-05	6.17E-04	lincRNA
CUST_2906_PI428871386	ENST00000452442.1	-0.90	4.94	6.44E-05	6.21E-04	antisense
A_33_P3214159	CDH2	2.04	7.12	6.45E-05	6.22E-04	protein_coding
A_33_P3413795	ENST00000427872	0.96	7.63	6.46E-05	6.22E-04	lincRNA
CUST_41351_PI428871386	ENST00000414042.1	-1.83	8.39	6.46E-05	6.22E-04	antisense
CUST_8076_PI428871386	ENST00000422681.1	-0.92	5.75	6.47E-05	6.23E-04	antisense
CUST_6574_PI428871386	ENST00000450443.1	1.34	6.38	6.48E-05	6.24E-04	antisense
CUST_18814_PI428871386	ENST00000518994.1	1.37	5.40	6.48E-05	6.24E-04	antisense
CUST_41196_PI428871386	ENST00000424566.1	1.26	6.45	6.49E-05	6.24E-04	antisense
A_33_P3366246	UGGT1	0.94	9.56	6.50E-05	6.26E-04	protein_coding
CUST_917_PI428871386	ENST00000444386.1	-0.95	4.95	6.54E-05	6.29E-04	antisense
CUST_26796_PI428871386	ENST00000564531.1	-1.14	6.03	6.57E-05	6.32E-04	lincRNA
CUST_5191_PI428871386	ENST00000415138.1	-0.87	4.96	6.58E-05	6.33E-04	antisense
A_24_P401739	ARHGAP17	-1.09	8.77	6.61E-05	6.36E-04	protein_coding
A_23_P47614	PHLDA2	2.29	10.11	6.63E-05	6.37E-04	protein_coding
CUST_38987_PI428871386	ENST00000589333.1	1.79	5.83	6.63E-05	6.37E-04	lincRNA

A_24_P74508	SLC25A18	-0.88	4.83	6.67E-05	6.41E-04	protein_coding
CUST_17781_PI428871386	ENST00000439694.1	1.77	5.16	6.75E-05	6.48E-04	antisense
A_23_P7761	C5orf22	0.94	6.90	6.76E-05	6.49E-04	protein_coding
A_23_P320578	RGS16	-1.80	11.67	6.77E-05	6.50E-04	protein_coding
A_24_P224998	ENST00000392994	1.11	6.59	6.77E-05	6.50E-04	protein_coding
CUST_17338_PI428871386	ENST00000431679.1	-1.19	6.99	6.79E-05	6.52E-04	lincRNA
A_24_P476086	KPNA5	-0.98	6.83	6.80E-05	6.52E-04	protein_coding
CUST_6156_PI428871386	ENST00000438432.1	0.93	5.03	6.81E-05	6.53E-04	antisense
CUST_19544_PI428871386	ENST00000562760.1	1.41	6.17	6.82E-05	6.54E-04	antisense
CUST_6663_PI428871386	ENST00000339037.3	1.34	6.24	6.82E-05	6.54E-04	lincRNA
A_33_P3411165	MAGEA2B	1.61	5.51	6.83E-05	6.54E-04	protein_coding
A_23_P500364	BCL7B	-0.84	8.58	6.83E-05	6.55E-04	protein_coding
CUST_29964_PI428871386	ENST00000556400.1	-0.93	5.92	6.84E-05	6.55E-04	antisense
CUST_20212_PI428871386	ENST00000518049.1	1.10	6.41	6.86E-05	6.57E-04	antisense
A_33_P3295358	ANGPTL4	2.36	9.91	6.86E-05	6.57E-04	protein_coding
CUST_37551_PI428871386	ENST00000579368.1	1.33	5.04	6.88E-05	6.59E-04	antisense
A_33_P3335999	B4GALT3	0.99	8.77	6.88E-05	6.59E-04	protein_coding
A_23_P256694	MCM3AP-AS1	1.15	5.84	6.90E-05	6.60E-04	antisense
A_33_P3419998	SLC4A8	-1.31	6.16	6.91E-05	6.61E-04	protein_coding
A_24_P224776	LSM4	1.13	9.20	6.93E-05	6.63E-04	protein_coding
A_23_P360240	MYEOV	1.66	6.24	6.93E-05	6.63E-04	protein_coding
A_33_P3241190	XRCC2	1.28	7.92	6.93E-05	6.63E-04	protein_coding
A_33_P3389178	RAPGEF3	-0.93	8.37	6.94E-05	6.63E-04	protein_coding
CUST_16875_PI428871386	ENST00000450016.1	1.12	10.10	7.00E-05	6.69E-04	antisense
A_23_P87013	TAGLN	-1.57	10.49	7.00E-05	6.70E-04	protein_coding
CUST_5847_PI428871386	ENST00000458252.1	-1.04	5.17	7.01E-05	6.70E-04	antisense
CUST_40441_PI428871386	ENST00000599889.1	-0.81	5.03	7.08E-05	6.77E-04	antisense
A_23_P204801	SLC41A2	1.36	6.77	7.10E-05	6.78E-04	protein_coding
A_23_P259251	KCND2	1.55	5.94	7.11E-05	6.79E-04	protein_coding
CUST_21449_PI428871386	ENST00000456032.1	-0.90	5.12	7.14E-05	6.82E-04	lincRNA
A_23_P5601	DOK1	-1.33	10.43	7.16E-05	6.83E-04	protein_coding
CUST_9897_PI428871386	ENST00000600382.1	-1.00	5.72	7.16E-05	6.83E-04	lincRNA
CUST_12095_PI428871386	ENST00000511758.1	-1.19	5.30	7.17E-05	6.85E-04	lincRNA
CUST_16151_PI428871386	ENST00000439703.1	0.85	5.12	7.22E-05	6.90E-04	lincRNA
A_23_P213754	PAIP2	-1.00	10.38	7.24E-05	6.91E-04	protein_coding
CUST_31286_PI428871386	ENST00000558089.1	-0.82	4.61	7.25E-05	6.92E-04	lincRNA
CUST_24516_PI428871386	ENST00000527239.1	-1.00	5.38	7.28E-05	6.94E-04	antisense
CUST_25916_PI428871386	ENST00000531710.1	0.84	4.73	7.29E-05	6.96E-04	antisense
CUST_921_PI428871386	ENST00000439057.1	1.11	5.09	7.30E-05	6.96E-04	antisense
A_33_P3419733	DNAJC5	1.00	8.01	7.34E-05	6.99E-04	protein_coding
A_32_P456318	SRSF12	1.80	5.48	7.35E-05	7.01E-04	protein_coding
CUST_34540_PI428871386	ENST00000561826.1	1.60	6.38	7.37E-05	7.02E-04	antisense
CUST_28564_PI428871386	ENST00000423211.1	-0.95	4.88	7.38E-05	7.04E-04	lincRNA
CUST_13038_PI428871386	ENST00000505109.1	0.83	4.98	7.39E-05	7.04E-04	lincRNA
CUST_20937_PI428871386	ENST00000436054.1	-0.89	5.44	7.40E-05	7.04E-04	lincRNA
A_33_P3227990	MBP	-1.08	6.20	7.41E-05	7.06E-04	protein_coding
CUST_33377_PI428871386	ENST00000565916.1	-0.96	4.76	7.47E-05	7.11E-04	antisense
CUST_23849_PI428871386	ENST00000597346.1	0.79	4.90	7.50E-05	7.13E-04	antisense
A_23_P17503	KIF16B	-1.25	8.24	7.52E-05	7.15E-04	protein_coding
CUST_11368_PI428871386	ENST00000499587.2	-0.94	5.12	7.54E-05	7.17E-04	antisense
A_33_P3225685	B3GAT3	1.01	9.49	7.55E-05	7.18E-04	protein_coding
A_32_P194115	SUN2	-1.13	8.01	7.55E-05	7.18E-04	protein_coding
A_24_P50972	GOLGA6L6	-1.32	7.07	7.55E-05	7.18E-04	protein_coding
A_24_P241815	JUNB	-1.66	8.73	7.60E-05	7.22E-04	protein_coding
CUST_22642_PI428871386	ENST00000450677.1	-0.97	5.27	7.66E-05	7.28E-04	lincRNA
A_32_P153071	VIPR2	-0.93	5.27	7.66E-05	7.28E-04	protein_coding
CUST_7941_PI428871386	ENST00000437506.1	0.94	4.80	7.66E-05	7.28E-04	lincRNA
CUST_37759_PI428871386	ENST00000581274.1	1.62	5.52	7.67E-05	7.28E-04	antisense
CUST_33941_PI428871386	ENST00000571340.1	1.10	5.52	7.69E-05	7.30E-04	lincRNA
CUST_34541_PI428871386	ENST00000561826.1	1.54	6.34	7.71E-05	7.32E-04	antisense
A_23_P26687	TMEM186	1.11	8.15	7.71E-05	7.32E-04	protein_coding
A_33_P3326099	FUBP3	0.94	6.88	7.74E-05	7.34E-04	protein_coding
A_33_P3504659	CASP10	-1.36	9.51	7.77E-05	7.37E-04	protein_coding

A_33_P3249489	WDR89	0.99	6.87	7.84E-05	7.44E-04	protein_coding
CUST_43740_PI428871386	ENST00000437981.1	-1.09	5.96	7.92E-05	7.51E-04	antisense
CUST_34860_PI428871386	ENST00000570711.1	-1.30	5.91	7.92E-05	7.51E-04	antisense
A_33_P3369153	KIF3C	1.28	6.95	7.93E-05	7.52E-04	protein_coding
CUST_22779_PI428871386	ENST00000394864.2	1.10	5.85	7.93E-05	7.52E-04	antisense
CUST_12887_PI428871386	ENST00000514840.1	-0.86	4.82	7.93E-05	7.52E-04	lincRNA
A_23_P144778	CKMT2	-1.20	6.01	7.95E-05	7.53E-04	protein_coding
CUST_18199_PI428871386	ENST00000523225.1	1.07	5.54	7.95E-05	7.53E-04	antisense
A_24_P273865	MED20	1.08	7.63	7.96E-05	7.54E-04	protein_coding
CUST_8359_PI428871386	ENST00000471990.2	-0.95	4.95	7.96E-05	7.54E-04	antisense
A_23_P29303	RRP7A	0.94	6.94	7.99E-05	7.57E-04	protein_coding
A_33_P3418516	E2F3	1.14	5.73	7.99E-05	7.57E-04	protein_coding
A_33_P3257027	FGF7	-1.30	7.88	8.00E-05	7.58E-04	protein_coding
CUST_8848_PI428871386	ENST00000468377.1	-1.79	8.33	8.01E-05	7.58E-04	antisense
CUST_30390_PI428871386	ENST00000556168.1	0.99	6.63	8.10E-05	7.66E-04	lincRNA
A_24_P284584	ZNF559	-0.92	6.52	8.12E-05	7.69E-04	protein_coding
CUST_14498_PI428871386	ENST00000566170.1	1.07	6.15	8.13E-05	7.69E-04	antisense
A_23_P253536	NPR3	-1.44	7.16	8.15E-05	7.71E-04	protein_coding
A_33_P3211604	RAP1B	-1.07	11.77	8.16E-05	7.72E-04	protein_coding
CUST_11995_PI428871386	ENST00000560688.1	-0.89	4.74	8.21E-05	7.76E-04	lincRNA
A_23_P317324	MECOM	-1.49	8.68	8.21E-05	7.76E-04	protein_coding
CUST_41777_PI428871386	ENST00000440052.1	-1.15	6.33	8.23E-05	7.78E-04	antisense
CUST_12181_PI428871386	ENST00000511443.1	-0.88	4.66	8.26E-05	7.80E-04	lincRNA
CUST_43946_PI428871386	ENST00000434164.1	0.78	4.88	8.28E-05	7.82E-04	antisense
CUST_2633_PI428871386	ENST00000443364.1	1.83	6.18	8.32E-05	7.86E-04	lincRNA
A_32_P827528	S1PR2	-1.23	9.86	8.34E-05	7.87E-04	protein_coding
A_33_P3257279	TMEM145	1.14	7.51	8.34E-05	7.88E-04	protein_coding
A_23_P218597	NPAS2	1.33	5.94	8.35E-05	7.88E-04	protein_coding
CUST_24471_PI428871386	ENST00000524565.1	-1.30	6.18	8.35E-05	7.89E-04	lincRNA
A_23_P77993	C1QL1	1.33	5.40	8.38E-05	7.91E-04	protein_coding
A_24_P942321	FRMD4A	-1.17	6.37	8.42E-05	7.95E-04	protein_coding
A_23_P207811	PAIP1	1.30	9.40	8.44E-05	7.96E-04	protein_coding
CUST_8728_PI428871386	ENST00000490351.1	1.05	5.11	8.46E-05	7.98E-04	antisense
CUST_11089_PI428871386	ENST00000491608.2	-0.98	7.16	8.46E-05	7.98E-04	lincRNA
CUST_8749_PI428871386	ENST00000493545.1	-0.89	4.87	8.47E-05	7.99E-04	lincRNA
CUST_28618_PI428871386	ENST00000522673.1	-1.04	7.18	8.49E-05	8.00E-04	antisense
A_23_P365719	TAPBP	-1.12	12.41	8.51E-05	8.02E-04	protein_coding
A_23_P162547	MYL2	-0.91	5.83	8.51E-05	8.02E-04	protein_coding
A_33_P3313456	CXorf30	-1.27	6.30	8.54E-05	8.05E-04	protein_coding
A_33_P3209869	TCP11L1	1.04	7.53	8.58E-05	8.08E-04	protein_coding
A_23_P167276	PAQR3	1.20	8.01	8.58E-05	8.08E-04	protein_coding
CUST_17845_PI428871386	ENST00000429630.1	-1.18	9.72	8.59E-05	8.08E-04	antisense
CUST_16943_PI428871386	ENST00000422831.1	-0.78	5.46	8.60E-05	8.09E-04	lincRNA
CUST_20425_PI428871386	ENST00000451142.1	-0.97	10.92	8.62E-05	8.11E-04	lincRNA
CUST_37974_PI428871386	ENST00000585627.1	1.90	8.79	8.62E-05	8.11E-04	lincRNA
A_23_P865	FRRS1	1.24	6.67	8.65E-05	8.14E-04	protein_coding
A_23_P87011	TAGLN	-1.31	11.44	8.67E-05	8.16E-04	protein_coding
CUST_279_PI428871386	ENST00000423764.1	-1.31	9.57	8.74E-05	8.22E-04	antisense
A_33_P3233645	MT1G	2.07	7.51	8.77E-05	8.25E-04	protein_coding
CUST_26048_PI428871386	ENST00000536141.1	-1.61	6.35	8.79E-05	8.27E-04	lincRNA
A_32_P231086	GOLGA6L9	-1.04	8.68	8.79E-05	8.27E-04	protein_coding
CUST_1800_PI428871386	ENST00000590826.1	1.13	5.95	8.82E-05	8.29E-04	antisense
A_32_P40375	ENST00000471090	1.13	7.14	8.83E-05	8.30E-04	lincRNA
A_33_P3293753	KRTAP10-9	-0.89	6.50	8.86E-05	8.33E-04	protein_coding
CUST_21704_PI428871386	ENST00000592466.1	-0.95	5.18	8.88E-05	8.34E-04	antisense
CUST_534_PI428871386	ENST00000426428.1	-0.80	4.94	8.89E-05	8.35E-04	lincRNA
A_23_P157865	TNC	1.86	8.39	8.89E-05	8.35E-04	protein_coding
CUST_30706_PI428871386	ENST00000522771.1	-1.76	6.83	8.97E-05	8.43E-04	lincRNA
CUST_20009_PI428871386	ENST00000523190.1	1.08	5.23	8.98E-05	8.43E-04	lincRNA
A_33_P3388651	ABLIM1	-1.46	8.88	8.99E-05	8.43E-04	protein_coding
A_32_P8402	SYNCRIP	0.99	10.27	9.05E-05	8.49E-04	protein_coding
CUST_24236_PI428871386	ENST00000501663.2	-1.01	6.29	9.11E-05	8.55E-04	antisense
A_32_P117313	C8orf83	-1.34	8.76	9.13E-05	8.56E-04	protein_coding

CUST_14961_PI428871386	ENST00000506206.1	1.82	5.27	9.14E-05	8.57E-04	lincRNA
CUST_18297_PI428871386	ENST00000518098.1	-0.91	5.37	9.15E-05	8.58E-04	lincRNA
A_23_P90659	LAPTM4A	-1.04	14.22	9.20E-05	8.62E-04	protein_coding
A_33_P3394689	SLC6A2	1.78	5.49	9.20E-05	8.62E-04	protein_coding
A_23_P47377	HSD17B12	-0.92	11.82	9.23E-05	8.65E-04	protein_coding
CUST_18920_PI428871386	ENST00000521483.1	-0.96	5.11	9.24E-05	8.66E-04	lincRNA
CUST_2726_PI428871386	ENST00000415000.1	-0.85	5.22	9.30E-05	8.71E-04	antisense
CUST_8581_PI428871386	ENST00000495228.1	-1.02	5.29	9.33E-05	8.74E-04	lincRNA
CUST_10586_PI428871386	ENST00000509782.1	0.78	4.88	9.34E-05	8.75E-04	lincRNA
CUST_919_PI428871386	ENST00000398804.3	1.10	5.03	9.36E-05	8.76E-04	antisense
CUST_7292_PI428871386	ENST00000596829.1	-0.80	5.12	9.37E-05	8.77E-04	antisense
CUST_935_PI428871386	ENST00000412378.1	1.05	5.86	9.39E-05	8.78E-04	antisense
CUST_26410_PI428871386	ENST00000446891.2	-1.24	6.47	9.40E-05	8.80E-04	lincRNA
CUST_26346_PI428871386	ENST00000418574.2	0.86	5.02	9.41E-05	8.80E-04	lincRNA
A_23_P137157	RENBP	-1.22	6.68	9.42E-05	8.81E-04	protein_coding
A_23_P215980	KIAA1429	1.05	6.79	9.53E-05	8.90E-04	protein_coding
CUST_30257_PI428871386	ENST00000560419.1	-1.35	7.21	9.54E-05	8.92E-04	antisense
A_23_P137016	SAT1	-1.50	13.89	9.57E-05	8.94E-04	protein_coding
A_32_P167396	ENST00000456333	-1.07	6.37	9.57E-05	8.94E-04	antisense
A_33_P3224858	EIF2AK2	1.18	8.08	9.58E-05	8.95E-04	protein_coding
CUST_11756_PI428871386	ENST00000502661.1	-0.90	5.13	9.60E-05	8.96E-04	lincRNA
CUST_6577_PI428871386	ENST00000416080.1	1.14	5.94	9.70E-05	9.05E-04	antisense
A_33_P3421626	KIAA1147	-1.19	9.57	9.71E-05	9.07E-04	protein_coding
A_33_P3417620	ZRSR2	-0.95	5.97	9.72E-05	9.07E-04	protein_coding
CUST_14177_PI428871386	ENST00000512934.1	-0.83	4.59	9.72E-05	9.07E-04	antisense
A_33_P3255404	CLDN11	-2.01	8.04	9.73E-05	9.08E-04	protein_coding
A_23_P51376	NKAIN1	2.18	5.77	9.74E-05	9.08E-04	protein_coding
CUST_15226_PI428871386	ENST00000418567.1	1.20	5.34	9.76E-05	9.10E-04	lincRNA
A_33_P3224745	PLA2G15	-1.21	12.11	9.80E-05	9.14E-04	protein_coding
A_23_P24515	ACAT1	-1.29	10.46	9.81E-05	9.15E-04	protein_coding
CUST_23694_PI428871386	ENST00000553459.1	-1.02	4.72	9.83E-05	9.16E-04	antisense
A_23_P92754	FGFR4	-0.88	6.83	9.84E-05	9.17E-04	protein_coding
CUST_19708_PI428871386	ENST00000520594.1	2.06	5.38	9.84E-05	9.17E-04	antisense
CUST_19067_PI428871386	ENST00000524309.1	-0.87	5.22	9.84E-05	9.17E-04	antisense
CUST_6547_PI428871386	ENST00000428156.1	-0.90	4.82	9.87E-05	9.19E-04	antisense
CUST_25255_PI428871386	ENST00000531869.1	0.93	5.91	9.95E-05	9.26E-04	antisense
CUST_32334_PI428871386	ENST00000440089.1	-0.87	4.75	9.98E-05	9.29E-04	lincRNA
CUST_31973_PI428871386	ENST00000565312.1	-0.98	5.64	9.98E-05	9.29E-04	antisense
CUST_42058_PI428871386	ENST00000411694.1	-1.27	7.52	1.00E-04	9.31E-04	lincRNA
A_33_P3275350	NCS1	1.15	5.90	1.00E-04	9.33E-04	protein_coding
A_32_P20523	TIGD2	1.14	7.79	1.01E-04	9.37E-04	protein_coding
A_33_P3395081	ACSM5	-1.10	5.87	1.01E-04	9.37E-04	protein_coding
A_33_P3281745	ENST00000423283	1.55	5.26	1.01E-04	9.43E-04	antisense
CUST_38452_PI428871386	ENST00000589723.1	-0.84	4.65	1.02E-04	9.47E-04	lincRNA
CUST_29072_PI428871386	ENST00000426037.1	1.42	6.12	1.02E-04	9.48E-04	lincRNA
A_33_P3772150	ADARB2	-1.93	6.39	1.02E-04	9.49E-04	protein_coding
A_33_P3261610	POLR3GL	-1.13	9.65	1.02E-04	9.51E-04	protein_coding
CUST_33626_PI428871386	ENST00000492040.1	1.05	5.34	1.03E-04	9.52E-04	antisense
CUST_26196_PI428871386	ENST00000539348.1	-1.50	7.01	1.03E-04	9.53E-04	lincRNA
CUST_30953_PI428871386	ENST00000460164.1	1.87	14.27	1.03E-04	9.54E-04	lincRNA
A_33_P3315258	CHD1L	1.11	7.03	1.03E-04	9.56E-04	protein_coding
A_24_P389038	WDR46	1.03	6.63	1.03E-04	9.57E-04	protein_coding
A_24_P239811	UBXN11	-0.94	7.18	1.04E-04	9.61E-04	protein_coding
A_23_P200143	DCAF8	-0.94	6.80	1.04E-04	9.64E-04	protein_coding
CUST_39616_PI428871386	ENST00000590657.1	1.29	6.42	1.04E-04	9.65E-04	lincRNA
CUST_16872_PI428871386	ENST00000443162.1	1.10	10.12	1.05E-04	9.71E-04	antisense
A_23_P73667	RIBC1	-1.09	7.44	1.05E-04	9.72E-04	protein_coding
CUST_30748_PI428871386	ENST00000522618.1	-0.96	5.66	1.06E-04	9.79E-04	lincRNA
CUST_1802_PI428871386	ENST00000585330.1	1.04	5.87	1.06E-04	9.81E-04	antisense
A_33_P3210218	GALNT1	1.44	9.71	1.06E-04	9.85E-04	protein_coding
A_23_P367899	EPOR	-1.02	7.07	1.07E-04	9.87E-04	protein_coding
CUST_17696_PI428871386	ENST00000447430.1	-0.88	5.41	1.07E-04	9.87E-04	lincRNA
CUST_17284_PI428871386	ENST00000435695.1	1.34	5.62	1.07E-04	9.92E-04	antisense

CUST_16874_PI428871386	ENST00000450016.1	1.11	10.13	1.07E-04	9.92E-04	antisense
CUST_4719_PI428871386	ENST00000413828.2	-1.10	5.96	1.07E-04	9.94E-04	antisense
CUST_16544_PI428871386	ENST00000439120.1	-0.87	5.03	1.08E-04	1.00E-03	lincRNA
A_32_P98502	COX5A	1.09	11.60	1.08E-04	1.00E-03	protein_coding
CUST_26209_PI428871386	ENST00000539795.1	-1.10	6.61	1.08E-04	1.00E-03	lincRNA
CUST_2534_PI428871386	ENST00000434575.1	-0.95	5.02	1.08E-04	1.00E-03	antisense
CUST_27737_PI428871386	ENST00000532697.1	-0.91	5.00	1.09E-04	1.01E-03	antisense
CUST_34523_PI428871386	ENST00000563750.1	1.10	6.45	1.09E-04	1.01E-03	lincRNA
A_24_P364236	NDUFC2	-1.06	8.31	1.09E-04	1.01E-03	protein_coding
A_23_P251132	SNTG2	-1.18	5.49	1.09E-04	1.01E-03	protein_coding
A_23_P126836	TNFSF4	1.40	6.37	1.10E-04	1.01E-03	protein_coding
CUST_15062_PI428871386	ENST00000399751.2	1.37	8.87	1.10E-04	1.01E-03	lincRNA
CUST_25345_PI428871386	ENST00000544019.1	0.91	5.30	1.10E-04	1.02E-03	lincRNA
CUST_10793_PI428871386	ENST00000508772.1	1.08	6.14	1.10E-04	1.02E-03	antisense
CUST_22883_PI428871386	ENST00000426234.1	-0.88	5.54	1.10E-04	1.02E-03	antisense
CUST_1006_PI428871386	ENST00000422306.1	-0.92	5.85	1.11E-04	1.02E-03	antisense
CUST_36573_PI428871386	ENST00000579859.1	-1.23	7.60	1.11E-04	1.02E-03	antisense
CUST_755_PI428871386	ENST00000445166.1	1.14	5.70	1.11E-04	1.03E-03	antisense
A_24_P915007	NACC1	0.92	9.67	1.12E-04	1.03E-03	protein_coding
CUST_3532_PI428871386	ENST00000424332.1	-1.22	7.43	1.12E-04	1.04E-03	antisense
CUST_33663_PI428871386	ENST00000565152.1	-0.87	5.05	1.13E-04	1.04E-03	antisense
A_23_P24244	FAM208B	0.93	7.80	1.13E-04	1.04E-03	protein_coding
CUST_12147_PI428871386	ENST00000416930.2	-1.14	5.45	1.13E-04	1.04E-03	antisense
CUST_9227_PI428871386	ENST00000492937.1	1.42	6.56	1.13E-04	1.04E-03	antisense
CUST_18986_PI428871386	ENST00000523683.1	-1.47	8.06	1.13E-04	1.04E-03	antisense
A_23_P53081	OSBPL5	-1.25	9.59	1.14E-04	1.05E-03	protein_coding
A_23_P425925	KRT222	-1.12	5.56	1.14E-04	1.05E-03	protein_coding
CUST_39966_PI428871386	ENST00000599817.1	1.55	5.47	1.15E-04	1.06E-03	lincRNA
A_23_P89460	AATF	0.93	9.76	1.15E-04	1.06E-03	protein_coding
A_24_P168726	NF2	0.94	6.48	1.15E-04	1.06E-03	protein_coding
CUST_31308_PI428871386	ENST00000560819.1	-0.86	5.22	1.16E-04	1.06E-03	lincRNA
A_32_P184518	RPL21	-1.10	15.54	1.17E-04	1.07E-03	protein_coding
A_33_P3283136	TMEM209	0.98	9.07	1.17E-04	1.07E-03	protein_coding
A_24_P38815	TPP1	-1.30	9.60	1.17E-04	1.07E-03	protein_coding
A_23_P164737	MED26	-1.03	9.59	1.17E-04	1.08E-03	protein_coding
CUST_2517_PI428871386	ENST00000411804.1	1.05	7.09	1.17E-04	1.08E-03	antisense
CUST_20832_PI428871386	ENST00000476224.1	-1.07	7.24	1.17E-04	1.08E-03	lincRNA
CUST_25974_PI428871386	ENST00000537514.1	-0.77	7.68	1.17E-04	1.08E-03	antisense
CUST_31889_PI428871386	ENST00000558050.1	-0.87	5.10	1.17E-04	1.08E-03	antisense
CUST_13739_PI428871386	ENST00000501695.3	-0.88	6.42	1.18E-04	1.08E-03	antisense
CUST_20276_PI428871386	ENST00000527086.1	-0.84	7.08	1.18E-04	1.09E-03	antisense
CUST_18005_PI428871386	ENST00000480632.1	-2.02	7.50	1.19E-04	1.09E-03	antisense
A_33_P3367565	FNBP1	-1.13	11.22	1.19E-04	1.09E-03	protein_coding
CUST_38096_PI428871386	ENST00000589125.1	1.46	5.31	1.19E-04	1.09E-03	lincRNA
CUST_17085_PI428871386	ENST00000567919.1	0.84	5.87	1.20E-04	1.10E-03	lincRNA
A_23_P140146	IFI27L2	-1.20	11.90	1.20E-04	1.10E-03	protein_coding
CUST_5851_PI428871386	ENST00000418615.1	-0.97	5.26	1.20E-04	1.11E-03	antisense
CUST_43024_PI428871386	ENST00000564152.1	1.14	7.84	1.21E-04	1.11E-03	antisense
CUST_314_PI428871386	ENST00000429480.2	1.15	5.37	1.21E-04	1.11E-03	lincRNA
CUST_38099_PI428871386	ENST00000592405.1	1.03	5.16	1.21E-04	1.11E-03	lincRNA
A_23_P207014	RAD51C	1.24	9.22	1.22E-04	1.12E-03	protein_coding
CUST_7066_PI428871386	ENST00000447111.1	0.92	5.15	1.23E-04	1.13E-03	antisense
A_33_P3310774	ENST00000510551	-1.27	5.74	1.23E-04	1.13E-03	lincRNA
CUST_922_PI428871386	ENST00000439057.1	1.00	4.96	1.23E-04	1.13E-03	antisense
A_23_P5586	MPHOSPH10	0.91	10.39	1.23E-04	1.13E-03	protein_coding
A_33_P3278941	REC8	-1.45	9.35	1.24E-04	1.13E-03	protein_coding
CUST_34730_PI428871386	ENST00000568031.1	1.15	5.16	1.24E-04	1.13E-03	lincRNA
A_33_P3252191	PRKCE	-1.04	6.00	1.24E-04	1.13E-03	protein_coding
CUST_41503_PI428871386	ENST00000453910.1	-1.24	6.62	1.24E-04	1.14E-03	lincRNA
A_33_P3271273	HOXB2	1.37	6.50	1.25E-04	1.14E-03	protein_coding
CUST_27185_PI428871386	ENST00000553135.1	1.11	8.10	1.25E-04	1.14E-03	antisense
A_23_P29185	TTC28	-1.10	8.12	1.25E-04	1.14E-03	protein_coding
CUST_9463_PI428871386	ENST00000432385.1	1.20	5.07	1.25E-04	1.14E-03	antisense

CUST_26173_PI428871386	ENST00000372173.5	-1.13	6.26	1.25E-04	1.14E-03	antisense
CUST_12082_PI428871386	ENST00000504494.1	0.80	4.93	1.26E-04	1.15E-03	lincRNA
CUST_10476_PI428871386	ENST00000411630.2	1.26	9.31	1.26E-04	1.15E-03	lincRNA
CUST_22014_PI428871386	ENST00000455810.1	-1.13	5.78	1.27E-04	1.16E-03	lincRNA
CUST_31386_PI428871386	ENST00000560415.1	-1.13	9.24	1.27E-04	1.16E-03	antisense
CUST_21219_PI428871386	ENST00000583864.1	0.92	8.11	1.27E-04	1.16E-03	lincRNA
A_33_P3240538	TUBE1	-1.05	8.38	1.28E-04	1.17E-03	protein_coding
A_24_P659836	SYCE3	1.40	6.97	1.28E-04	1.17E-03	protein_coding
CUST_19791_PI428871386	ENST00000531508.1	1.94	9.49	1.28E-04	1.17E-03	protein_coding
A_23_P125624	ACOT9	-1.02	10.86	1.29E-04	1.18E-03	protein_coding
CUST_9718_PI428871386	ENST00000426459.1	-1.33	6.97	1.29E-04	1.18E-03	antisense
CUST_27832_PI428871386	ENST00000539446.1	1.08	6.18	1.29E-04	1.18E-03	antisense
CUST_16579_PI428871386	ENST00000522193.1	-1.03	5.88	1.29E-04	1.18E-03	antisense
A_23_P500206	IL17RE	-1.07	7.65	1.29E-04	1.18E-03	protein_coding
CUST_5556_PI428871386	ENST00000458149.3	-0.97	6.42	1.29E-04	1.18E-03	antisense
CUST_22643_PI428871386	ENST00000450677.1	-1.04	5.25	1.29E-04	1.18E-03	lincRNA
A_23_P58228	ODAM	-1.50	6.82	1.30E-04	1.18E-03	protein_coding
CUST_31654_PI428871386	ENST00000559909.1	1.30	5.51	1.30E-04	1.18E-03	lincRNA
A_23_P93898	FSCN3	-0.90	5.26	1.30E-04	1.19E-03	protein_coding
CUST_38651_PI428871386	ENST00000592368.1	-0.81	4.80	1.31E-04	1.19E-03	lincRNA
A_33_P3222210	FAM108A1	-1.05	9.18	1.31E-04	1.19E-03	protein_coding
A_23_P15692	GPR172B	1.46	7.04	1.31E-04	1.20E-03	protein_coding
CUST_36721_PI428871386	ENST00000582940.1	1.32	7.02	1.32E-04	1.20E-03	lincRNA
CUST_42758_PI428871386	ENST00000430449.1	-0.81	5.06	1.32E-04	1.20E-03	lincRNA
CUST_33276_PI428871386	ENST00000575424.1	-1.17	6.00	1.32E-04	1.20E-03	lincRNA
A_23_P171397	RBMY1B	-1.04	4.82	1.32E-04	1.20E-03	protein_coding
CUST_14856_PI428871386	ENST00000414386.1	1.59	6.69	1.32E-04	1.20E-03	antisense
A_33_P3386932	FAM86A	1.06	8.92	1.32E-04	1.20E-03	protein_coding
A_24_P339429	KCNJ12	-1.62	7.19	1.32E-04	1.21E-03	protein_coding
A_24_P348265	FCAR	-1.31	5.70	1.32E-04	1.21E-03	protein_coding
A_23_P318396	CELF1	1.17	9.05	1.32E-04	1.21E-03	protein_coding
CUST_34139_PI428871386	ENST00000568560.1	-0.94	6.71	1.33E-04	1.21E-03	lincRNA
A_23_P167168	IGJ	-2.27	11.76	1.33E-04	1.21E-03	protein_coding
A_23_P252306	ID1	-1.85	12.18	1.33E-04	1.21E-03	protein_coding
CUST_20297_PI428871386	ENST00000524998.1	0.83	5.05	1.33E-04	1.21E-03	protein_coding
CUST_14857_PI428871386	ENST00000414386.1	1.57	6.69	1.34E-04	1.22E-03	antisense
A_23_P52974	GIF	-0.88	4.92	1.34E-04	1.22E-03	protein_coding
CUST_4284_PI428871386	ENST00000565044.1	1.54	5.13	1.35E-04	1.22E-03	lincRNA
CUST_9776_PI428871386	ENST00000456816.1	1.21	5.08	1.35E-04	1.23E-03	lincRNA
A_33_P3356341	MSL1	1.14	7.91	1.35E-04	1.23E-03	protein_coding
A_24_P73577	ALDH1A2	-1.15	5.82	1.35E-04	1.23E-03	protein_coding
CUST_19878_PI428871386	ENST00000519861.1	0.99	6.35	1.35E-04	1.23E-03	lincRNA
CUST_39526_PI428871386	ENST00000424536.1	1.15	7.06	1.35E-04	1.23E-03	protein_coding
A_24_P296568	CBX1	1.13	10.64	1.36E-04	1.23E-03	protein_coding
CUST_20087_PI428871386	ENST00000518674.1	0.95	6.43	1.36E-04	1.24E-03	antisense
CUST_41022_PI428871386	ENST00000440918.1	-1.05	5.44	1.36E-04	1.24E-03	lincRNA
A_23_P87580	ANP32D	0.96	7.43	1.37E-04	1.24E-03	protein_coding
A_23_P51936	TNFRSF9	1.15	5.88	1.37E-04	1.24E-03	protein_coding
A_23_P65129	SPRYD3	-0.81	8.88	1.37E-04	1.24E-03	protein_coding
CUST_21626_PI428871386	ENST00000316786.1	-1.25	6.15	1.38E-04	1.25E-03	lincRNA
CUST_37470_PI428871386	ENST00000582008.1	-1.00	8.34	1.38E-04	1.25E-03	lincRNA
A_24_P827	SUPT3H	1.13	5.85	1.39E-04	1.26E-03	protein_coding
CUST_11965_PI428871386	ENST00000514376.1	-1.06	5.03	1.39E-04	1.27E-03	antisense
A_23_P32414	MST4	1.23	9.58	1.40E-04	1.27E-03	protein_coding
A_23_P25615	SOHLH2	2.07	5.42	1.40E-04	1.27E-03	protein_coding
CUST_20672_PI428871386	ENST00000452923.1	0.95	6.07	1.40E-04	1.27E-03	lincRNA
A_23_P54605	RSL1D1	1.05	10.27	1.40E-04	1.27E-03	protein_coding
CUST_42760_PI428871386	ENST00000416995.1	-0.78	4.58	1.40E-04	1.27E-03	lincRNA
A_33_P3272209	MFSD6	-1.47	9.46	1.41E-04	1.27E-03	protein_coding
CUST_15058_PI428871386	ENST00000450081.1	1.38	8.95	1.41E-04	1.27E-03	lincRNA
A_23_P82503	PEG10	-2.02	8.00	1.41E-04	1.28E-03	protein_coding
CUST_1958_PI428871386	ENST00000457043.1	-1.00	6.76	1.41E-04	1.28E-03	antisense
CUST_32160_PI428871386	ENST00000568853.1	0.84	6.63	1.41E-04	1.28E-03	antisense

CUST_34521_PI428871386	ENST00000568107.1	1.24	8.63	1.41E-04	1.28E-03	protein_coding
CUST_23743_PI428871386	ENST00000527620.1	-1.51	7.69	1.42E-04	1.28E-03	antisense
A_33_P3280192	ANKRD27	1.04	6.65	1.42E-04	1.28E-03	protein_coding
CUST_9927_PI428871386	ENST00000429834.1	-0.90	5.07	1.42E-04	1.29E-03	lincRNA
CUST_17048_PI428871386	ENST00000452565.1	1.19	7.71	1.42E-04	1.29E-03	lincRNA
CUST_21646_PI428871386	ENST00000454968.1	-1.16	9.65	1.43E-04	1.29E-03	lincRNA
A_33_P3361758	C15orf33	1.44	5.51	1.43E-04	1.29E-03	protein_coding
CUST_30868_PI428871386	ENST00000559946.1	1.23	6.53	1.43E-04	1.29E-03	lincRNA
A_23_P88033	FGF14	-1.11	5.41	1.43E-04	1.29E-03	protein_coding
CUST_232_PI428871386	ENST00000449660.1	-0.87	5.72	1.45E-04	1.31E-03	antisense
CUST_34727_PI428871386	ENST00000566351.1	-0.88	5.53	1.45E-04	1.31E-03	lincRNA
CUST_20729_PI428871386	ENST00000593047.1	-0.92	5.15	1.45E-04	1.31E-03	lincRNA
CUST_391_PI428871386	ENST00000416696.1	-0.89	5.03	1.45E-04	1.31E-03	lincRNA
A_33_P3267543	DPM2	-0.78	5.49	1.45E-04	1.31E-03	protein_coding
A_23_P156861	RGS17	1.52	5.07	1.46E-04	1.32E-03	protein_coding
A_33_P3293009	NKAIN4	1.44	6.73	1.46E-04	1.32E-03	protein_coding
CUST_31942_PI428871386	ENST00000502156.1	1.14	5.22	1.46E-04	1.32E-03	lincRNA
CUST_14862_PI428871386	ENST00000432751.1	1.52	6.82	1.46E-04	1.32E-03	antisense
CUST_24990_PI428871386	ENST00000524619.1	1.10	5.46	1.46E-04	1.32E-03	antisense
A_33_P3239287	CHD3	-0.92	10.36	1.46E-04	1.32E-03	protein_coding
A_23_P390148	RALGPS1	-1.15	6.84	1.47E-04	1.32E-03	protein_coding
CUST_18839_PI428871386	ENST00000524011.1	-0.97	4.78	1.47E-04	1.33E-03	lincRNA
A_23_P160567	ZMYND12	-1.21	6.68	1.47E-04	1.33E-03	protein_coding
CUST_39680_PI428871386	ENST00000587477.1	1.30	6.81	1.48E-04	1.33E-03	lincRNA
CUST_14743_PI428871386	ENST00000413039.1	-1.15	9.52	1.48E-04	1.33E-03	lincRNA
A_24_P278460	FAR1	-1.11	7.71	1.48E-04	1.33E-03	protein_coding
A_24_P148717	CCR1	-1.64	9.40	1.48E-04	1.33E-03	protein_coding
A_33_P3211956	RGS19	-1.34	10.49	1.48E-04	1.34E-03	protein_coding
A_33_P3367830	EFEMP2	-1.42	10.74	1.48E-04	1.34E-03	protein_coding
A_33_P3400708	BNIPL	-1.88	8.08	1.49E-04	1.34E-03	protein_coding
A_23_P4572	MYL12A	-1.09	13.90	1.49E-04	1.34E-03	protein_coding
CUST_5304_PI428871386	ENST00000415060.2	-0.83	5.19	1.49E-04	1.34E-03	antisense
CUST_18129_PI428871386	ENST00000577187.1	0.80	4.85	1.50E-04	1.35E-03	antisense
CUST_39620_PI428871386	ENST00000449434.2	1.30	6.75	1.50E-04	1.35E-03	lincRNA
A_23_P208698	GYS1	0.85	9.34	1.50E-04	1.35E-03	protein_coding
CUST_7844_PI428871386	ENST00000414198.1	1.14	5.77	1.50E-04	1.35E-03	antisense
CUST_14228_PI428871386	ENST00000519603.1	0.82	10.20	1.50E-04	1.35E-03	lincRNA
CUST_38227_PI428871386	ENST00000585706.1	-0.99	5.10	1.50E-04	1.35E-03	lincRNA
CUST_12572_PI428871386	ENST00000505861.1	-0.89	4.70	1.50E-04	1.35E-03	lincRNA
CUST_15656_PI428871386	ENST00000419695.1	-0.82	4.64	1.51E-04	1.36E-03	lincRNA
A_23_P20316	CA3	-2.09	6.45	1.51E-04	1.36E-03	protein_coding
CUST_39696_PI428871386	ENST00000585890.1	1.80	6.77	1.51E-04	1.36E-03	antisense
CUST_41_PI428871386	ENST00000601486.1	-0.81	7.10	1.51E-04	1.36E-03	lincRNA
A_33_P3362891	HNRNPA2B1	0.95	12.29	1.51E-04	1.36E-03	protein_coding
CUST_11653_PI428871386	ENST00000563631.1	-0.86	5.08	1.51E-04	1.36E-03	lincRNA
CUST_35489_PI428871386	ENST00000578585.1	-1.36	9.77	1.51E-04	1.36E-03	lincRNA
CUST_34137_PI428871386	ENST00000569125.1	0.84	5.23	1.52E-04	1.36E-03	antisense
A_33_P3335865	WDR35	-1.07	7.55	1.52E-04	1.37E-03	protein_coding
CUST_15159_PI428871386	ENST00000421315.1	1.11	5.57	1.52E-04	1.37E-03	antisense
A_33_P3210278	SYNE2	-1.09	11.39	1.52E-04	1.37E-03	protein_coding
CUST_31395_PI428871386	ENST00000558601.1	1.31	7.18	1.54E-04	1.38E-03	antisense
CUST_26524_PI428871386	ENST00000551287.1	-0.77	4.73	1.54E-04	1.38E-03	lincRNA
A_24_P77676	HSPA9	1.01	9.62	1.55E-04	1.39E-03	protein_coding
CUST_29885_PI428871386	ENST00000555643.1	-0.87	4.70	1.55E-04	1.39E-03	lincRNA
CUST_42273_PI428871386	ENST00000565162.1	1.06	5.87	1.56E-04	1.40E-03	antisense
A_24_P99071	IPO5	1.21	8.94	1.56E-04	1.40E-03	protein_coding
A_23_P62377	PNCK	1.51	5.25	1.58E-04	1.41E-03	protein_coding
CUST_9717_PI428871386	ENST00000431512.1	-1.86	7.40	1.58E-04	1.41E-03	antisense
A_23_P63660	C10orf58	1.20	10.73	1.58E-04	1.42E-03	protein_coding
A_23_P254271	TUBB6	-1.60	13.35	1.60E-04	1.43E-03	protein_coding
CUST_1617_PI428871386	ENST00000427695.2	-0.81	6.39	1.61E-04	1.44E-03	lincRNA
CUST_31929_PI428871386	ENST00000560662.1	-0.83	4.66	1.61E-04	1.44E-03	lincRNA
A_23_P169017	DEFB103B	1.71	5.54	1.61E-04	1.44E-03	protein_coding

A_24_P46577	ZNRD1	1.06	10.93	1.62E-04	1.45E-03	protein_coding
CUST_33622_PI428871386	ENST00000569752.1	1.11	5.84	1.62E-04	1.45E-03	antisense
A_23_P306215	FAM84A	2.03	7.09	1.63E-04	1.46E-03	protein_coding
CUST_19394_PI428871386	ENST00000524052.1	-1.06	5.59	1.63E-04	1.46E-03	antisense
A_33_P3339066	RNPC3	-1.12	10.50	1.63E-04	1.46E-03	protein_coding
CUST_23890_PI428871386	ENST00000418080.1	0.87	5.93	1.63E-04	1.46E-03	antisense
A_23_P63432	RHBDL2	1.82	6.38	1.63E-04	1.46E-03	protein_coding
A_33_P3368358	NEDD9	-1.48	7.56	1.63E-04	1.46E-03	protein_coding
CUST_31304_PI428871386	ENST00000558897.1	-1.42	6.14	1.64E-04	1.47E-03	lincRNA
CUST_38339_PI428871386	ENST00000581541.1	1.14	5.04	1.65E-04	1.47E-03	lincRNA
CUST_42997_PI428871386	ENST00000432502.1	1.39	7.69	1.65E-04	1.47E-03	lincRNA
CUST_28229_PI428871386	ENST00000456737.1	0.96	5.62	1.65E-04	1.47E-03	antisense
A_23_P212159	NUP210	1.22	7.12	1.65E-04	1.48E-03	protein_coding
A_23_P12989	PRDX5	-1.07	13.38	1.65E-04	1.48E-03	protein_coding
A_33_P3389704	CES1	-2.18	7.90	1.65E-04	1.48E-03	protein_coding
A_33_P3237266	IDS	-1.00	6.46	1.66E-04	1.48E-03	protein_coding
CUST_581_PI428871386	ENST00000427796.1	-1.06	7.51	1.66E-04	1.49E-03	antisense
CUST_33853_PI428871386	ENST00000566876.1	1.46	5.33	1.67E-04	1.49E-03	lincRNA
A_23_P252681	PCYT1A	0.95	8.75	1.67E-04	1.49E-03	protein_coding
A_24_P379750	MXD1	-1.16	9.24	1.67E-04	1.49E-03	protein_coding
A_24_P122524	WDR3	1.19	8.18	1.68E-04	1.50E-03	protein_coding
CUST_7139_PI428871386	ENST00000448588.1	1.11	6.82	1.68E-04	1.50E-03	antisense
A_33_P3368855	GPC5	-0.95	4.84	1.68E-04	1.50E-03	protein_coding
A_33_P3389133	MINPP1	1.08	6.62	1.69E-04	1.51E-03	protein_coding
CUST_19710_PI428871386	ENST00000518932.1	1.97	5.19	1.69E-04	1.51E-03	antisense
CUST_7316_PI428871386	ENST00000587099.1	1.74	5.96	1.69E-04	1.51E-03	lincRNA
CUST_28691_PI428871386	ENST00000458725.1	1.26	7.19	1.70E-04	1.51E-03	antisense
CUST_42179_PI428871386	ENST00000438574.1	1.40	5.26	1.70E-04	1.52E-03	lincRNA
CUST_17708_PI428871386	ENST00000433079.1	-1.37	8.52	1.70E-04	1.52E-03	antisense
A_33_P3281532	RMND5A	0.98	6.95	1.71E-04	1.52E-03	protein_coding
A_23_P386411	PPID	1.04	8.77	1.71E-04	1.52E-03	protein_coding
CUST_41651_PI428871386	ENST00000567517.1	-0.91	5.03	1.72E-04	1.53E-03	antisense
CUST_35025_PI428871386	ENST00000571138.1	1.30	8.90	1.72E-04	1.53E-03	antisense
A_33_P3353672	RAB28	-0.89	9.89	1.73E-04	1.54E-03	protein_coding
A_23_P77066	SNRPN	-1.45	8.32	1.74E-04	1.55E-03	protein_coding
A_24_P95439	CARS	1.00	7.06	1.74E-04	1.55E-03	protein_coding
CUST_39700_PI428871386	ENST00000585397.1	1.53	6.06	1.74E-04	1.55E-03	antisense
CUST_4259_PI428871386	ENST00000418358.1	-0.86	4.82	1.74E-04	1.55E-03	lincRNA
A_33_P3224809	IL17RA	-1.00	10.00	1.74E-04	1.55E-03	protein_coding
A_33_P3662553	YWHAQ	1.02	9.73	1.74E-04	1.55E-03	protein_coding
CUST_10971_PI428871386	ENST00000506148.1	1.13	6.58	1.75E-04	1.56E-03	lincRNA
A_32_P109572	HNRNPL	0.81	10.00	1.75E-04	1.56E-03	protein_coding
CUST_17049_PI428871386	ENST00000452565.1	1.18	8.13	1.76E-04	1.56E-03	lincRNA
CUST_30742_PI428871386	ENST00000455531.1	-1.73	6.92	1.76E-04	1.56E-03	lincRNA
A_23_P205164	POU4F1	1.82	5.15	1.76E-04	1.57E-03	protein_coding
A_33_P3350710	EXOC6B	0.89	5.39	1.76E-04	1.57E-03	protein_coding
A_33_P3411075	FSCN1	1.57	12.13	1.77E-04	1.58E-03	protein_coding
CUST_7294_PI428871386	ENST00000597192.1	1.06	5.55	1.78E-04	1.58E-03	antisense
CUST_14860_PI428871386	ENST00000439386.1	1.62	7.09	1.78E-04	1.58E-03	antisense
CUST_3315_PI428871386	ENST00000534914.1	1.26	8.10	1.78E-04	1.58E-03	lincRNA
CUST_26207_PI428871386	ENST00000535746.1	-0.98	6.49	1.78E-04	1.58E-03	lincRNA
A_23_P138856	DRAP1	1.05	11.80	1.78E-04	1.58E-03	protein_coding
CUST_33527_PI428871386	ENST00000563565.1	0.85	6.42	1.79E-04	1.59E-03	antisense
CUST_28228_PI428871386	ENST00000456737.1	0.92	5.74	1.79E-04	1.59E-03	antisense
CUST_25168_PI428871386	ENST00000532831.1	0.94	5.55	1.80E-04	1.60E-03	antisense
A_23_P15402	SAT2	-1.19	12.72	1.80E-04	1.60E-03	protein_coding
CUST_8804_PI428871386	ENST00000597749.1	-0.83	5.17	1.81E-04	1.60E-03	lincRNA
CUST_43712_PI428871386	ENST00000458472.1	0.87	4.85	1.81E-04	1.61E-03	lincRNA
CUST_1241_PI428871386	ENST00000444349.1	-0.83	4.93	1.82E-04	1.61E-03	lincRNA
A_33_P3366575	NXT2	1.16	6.40	1.82E-04	1.61E-03	protein_coding
A_24_P396197	PRKCSH	0.86	8.05	1.82E-04	1.61E-03	protein_coding
A_23_P425332	PPP4R4	-1.52	6.00	1.82E-04	1.61E-03	protein_coding
CUST_1533_PI428871386	ENST00000436200.1	-1.01	12.26	1.82E-04	1.62E-03	lincRNA

CUST_37604_PI428871386	ENST00000586947.1	0.81	4.97	1.82E-04	1.62E-03	lincRNA
CUST_27649_PI428871386	ENST00000478808.2	-1.75	8.56	1.83E-04	1.62E-03	antisense
A_33_P3339481	KCNQ5	0.77	4.83	1.83E-04	1.62E-03	protein_coding
CUST_36252_PI428871386	ENST00000575126.1	1.08	7.14	1.83E-04	1.62E-03	lincRNA
CUST_42298_PI428871386	ENST00000506039.1	-0.91	4.92	1.83E-04	1.62E-03	antisense
CUST_30397_PI428871386	ENST00000554433.1	-0.90	4.77	1.83E-04	1.62E-03	antisense
A_23_P345591	PSMA2	1.07	11.71	1.83E-04	1.62E-03	protein_coding
CUST_21863_PI428871386	ENST00000434470.1	-0.85	4.89	1.83E-04	1.62E-03	antisense
CUST_16613_PI428871386	ENST00000523790.1	1.14	5.50	1.85E-04	1.64E-03	antisense
A_23_P377819	SRSF5	-0.91	12.53	1.85E-04	1.64E-03	protein_coding
CUST_39621_PI428871386	ENST00000449434.2	1.39	7.12	1.85E-04	1.64E-03	lincRNA
A_23_P74609	G0S2	-1.93	10.65	1.85E-04	1.64E-03	protein_coding
A_23_P47226	YIF1A	1.04	12.44	1.85E-04	1.64E-03	protein_coding
A_24_P106542	RSPO3	-1.62	8.34	1.86E-04	1.64E-03	protein_coding
CUST_7727_PI428871386	ENST00000524210.1	-1.04	6.82	1.86E-04	1.64E-03	antisense
A_24_P28619	ANGEL2	-0.94	8.25	1.86E-04	1.65E-03	protein_coding
CUST_198_PI428871386	ENST00000434150.1	0.96	5.68	1.87E-04	1.65E-03	lincRNA
A_23_P316501	NKAIN2	2.17	5.41	1.87E-04	1.65E-03	protein_coding
CUST_17403_PI428871386	ENST00000449764.1	-0.90	4.98	1.87E-04	1.66E-03	antisense
A_24_P649747	BMS1	0.96	8.54	1.88E-04	1.66E-03	protein_coding
A_24_P185945	MAGEA4	2.44	5.63	1.88E-04	1.66E-03	protein_coding
CUST_14116_PI428871386	ENST00000519942.1	0.80	4.90	1.88E-04	1.66E-03	antisense
A_24_P347480	NEK9	-1.15	9.95	1.89E-04	1.67E-03	protein_coding
CUST_3671_PI428871386	ENST00000549744.1	-0.84	4.68	1.89E-04	1.67E-03	lincRNA
A_23_P151133	TSPAN9	-1.19	7.59	1.89E-04	1.67E-03	protein_coding
CUST_8729_PI428871386	ENST00000490351.1	0.91	5.21	1.90E-04	1.68E-03	antisense
CUST_13582_PI428871386	ENST00000511256.1	1.39	5.37	1.90E-04	1.68E-03	lincRNA
A_23_P34827	HCN3	1.19	6.22	1.91E-04	1.68E-03	protein_coding
A_23_P256413	CMTM7	-1.15	11.45	1.92E-04	1.69E-03	protein_coding
A_33_P3354322	GPX1	-1.39	14.83	1.93E-04	1.70E-03	protein_coding
A_24_P930926	BAG4	1.21	6.78	1.94E-04	1.71E-03	protein_coding
CUST_25357_PI428871386	ENST00000279839.6	-0.92	7.24	1.94E-04	1.71E-03	protein_coding
A_33_P3417339	SHROOM3	-1.07	6.68	1.95E-04	1.72E-03	protein_coding
CUST_12146_PI428871386	ENST00000416930.2	-1.10	5.43	1.95E-04	1.72E-03	antisense
A_33_P3251876	IL18R1	-1.22	6.11	1.96E-04	1.73E-03	protein_coding
CUST_27833_PI428871386	ENST00000539446.1	1.18	6.32	1.96E-04	1.73E-03	antisense
CUST_13497_PI428871386	ENST00000514573.1	-0.91	7.98	1.96E-04	1.73E-03	lincRNA
CUST_17846_PI428871386	ENST00000429630.1	-1.18	9.84	1.96E-04	1.73E-03	antisense
A_23_P75453	MEN1	0.90	7.62	1.97E-04	1.73E-03	protein_coding
CUST_25436_PI428871386	ENST00000532422.1	-0.84	4.91	1.97E-04	1.73E-03	antisense
A_23_P381714	CA13	-1.47	7.80	1.97E-04	1.73E-03	protein_coding
A_23_P64044	FERMT3	-1.45	7.74	1.99E-04	1.75E-03	protein_coding
CUST_30135_PI428871386	ENST00000556301.1	1.57	6.06	1.99E-04	1.75E-03	antisense
CUST_26871_PI428871386	ENST00000424518.1	1.67	4.80	1.99E-04	1.75E-03	antisense
CUST_7523_PI428871386	ENST00000434306.1	-1.43	5.42	1.99E-04	1.75E-03	antisense
CUST_32645_PI428871386	ENST00000561402.1	-1.23	6.08	2.00E-04	1.76E-03	antisense
CUST_26586_PI428871386	ENST00000417422.1	-1.00	5.43	2.00E-04	1.76E-03	lincRNA
CUST_29680_PI428871386	ENST00000557761.1	0.82	4.95	2.00E-04	1.76E-03	antisense
A_33_P3264238	ZNF280B	1.31	5.41	2.00E-04	1.76E-03	protein_coding
A_23_P252882	CSN3	0.81	4.87	2.01E-04	1.76E-03	protein_coding
A_23_P57784	CLDN1	2.18	8.15	2.02E-04	1.77E-03	protein_coding
CUST_27598_PI428871386	ENST00000549807.1	1.43	5.28	2.02E-04	1.77E-03	antisense
CUST_27256_PI428871386	ENST00000551726.1	1.25	5.12	2.03E-04	1.78E-03	lincRNA
CUST_15381_PI428871386	ENST00000565695.1	1.10	6.54	2.03E-04	1.78E-03	antisense
A_23_P253321	PNOC	1.87	7.19	2.03E-04	1.78E-03	protein_coding
CUST_37975_PI428871386	ENST00000585627.1	1.74	7.87	2.05E-04	1.80E-03	lincRNA
CUST_36137_PI428871386	ENST00000585620.1	-0.80	4.77	2.05E-04	1.80E-03	lincRNA
CUST_43146_PI428871386	ENST00000451564.1	-1.07	4.94	2.06E-04	1.81E-03	antisense
A_23_P11032	SLC9A7	1.24	7.19	2.06E-04	1.81E-03	protein_coding
CUST_16241_PI428871386	ENST00000549241.1	0.89	7.09	2.07E-04	1.81E-03	antisense
A_23_P201647	SMG7	0.85	7.32	2.07E-04	1.81E-03	protein_coding
CUST_7524_PI428871386	ENST00000434306.1	-1.39	5.43	2.07E-04	1.82E-03	antisense
A_32_P184796	RPLP0	0.92	15.60	2.08E-04	1.82E-03	protein_coding

A_23_P87827	UNC119B	-1.07	9.41	2.08E-04	1.82E-03	protein_coding
A_32_P155416	ERI3	1.01	7.75	2.08E-04	1.82E-03	protein_coding
A_23_P342131	CYBASC3	-1.14	7.99	2.08E-04	1.82E-03	protein_coding
CUST_24468_PI428871386	ENST00000524488.1	-1.27	6.33	2.08E-04	1.83E-03	lincRNA
A_24_P259607	SUSD4	1.31	5.57	2.09E-04	1.83E-03	protein_coding
CUST_14539_PI428871386	ENST00000457670.1	-0.94	5.00	2.09E-04	1.83E-03	lincRNA
CUST_34575_PI428871386	ENST00000567342.1	-0.84	4.93	2.09E-04	1.83E-03	antisense
A_33_P3234657	PRTG	-1.03	5.65	2.09E-04	1.83E-03	protein_coding
A_24_P235988	CLEC7A	-1.14	5.99	2.11E-04	1.84E-03	protein_coding
A_33_P3214298	IMPDH2	1.12	9.74	2.11E-04	1.84E-03	protein_coding
A_33_P3250133	VSIG10	-1.33	7.38	2.11E-04	1.85E-03	protein_coding
A_23_P361448	SESN3	1.33	6.64	2.12E-04	1.85E-03	protein_coding
A_24_P124370	PARVA	-1.18	9.16	2.13E-04	1.86E-03	protein_coding
CUST_31667_PI428871386	ENST00000559977.1	-1.02	5.48	2.13E-04	1.87E-03	antisense
CUST_27903_PI428871386	ENST00000538710.1	1.13	5.80	2.15E-04	1.88E-03	lincRNA
A_23_P351667	ADAM23	1.82	6.02	2.16E-04	1.88E-03	protein_coding
CUST_9777_PI428871386	ENST00000456816.1	1.23	5.12	2.16E-04	1.89E-03	lincRNA
A_23_P304287	PSMC2	0.98	11.11	2.16E-04	1.89E-03	protein_coding
A_23_P25003	CLIP1	-1.15	10.19	2.16E-04	1.89E-03	protein_coding
A_32_P468743	ODF3L1	-1.22	5.87	2.17E-04	1.90E-03	protein_coding
CUST_32849_PI428871386	ENST00000567820.1	-0.97	5.18	2.17E-04	1.90E-03	antisense
CUST_40277_PI428871386	ENST00000594721.1	-1.40	6.24	2.17E-04	1.90E-03	lincRNA
CUST_16668_PI428871386	ENST00000416513.1	0.81	5.12	2.18E-04	1.90E-03	antisense
A_23_P99253	LIN7A	-1.07	6.47	2.18E-04	1.90E-03	protein_coding
A_33_P3324004	CCL15	-1.10	6.72	2.19E-04	1.91E-03	protein_coding
A_32_P435367	PSMD4	0.88	12.63	2.19E-04	1.91E-03	protein_coding
CUST_40753_PI428871386	ENST00000443744.1	-0.86	4.62	2.21E-04	1.93E-03	lincRNA
A_24_P309521	KCNJ5	-1.40	6.22	2.22E-04	1.93E-03	protein_coding
CUST_11152_PI428871386	ENST00000515769.1	-0.98	5.27	2.22E-04	1.94E-03	lincRNA
A_24_P56837	ASB16	-1.07	7.36	2.22E-04	1.94E-03	protein_coding
CUST_16438_PI428871386	ENST00000448664.1	-0.99	6.43	2.22E-04	1.94E-03	antisense
CUST_33672_PI428871386	ENST00000565137.1	-1.18	5.79	2.23E-04	1.94E-03	antisense
CUST_18469_PI428871386	ENST00000502083.2	-0.98	6.43	2.23E-04	1.94E-03	antisense
CUST_4191_PI428871386	ENST00000415657.1	1.32	6.39	2.25E-04	1.96E-03	lincRNA
CUST_17518_PI428871386	ENST00000439070.1	-0.99	5.22	2.25E-04	1.96E-03	antisense
A_24_P119259	BLZF1	0.93	6.36	2.27E-04	1.97E-03	protein_coding
CUST_31090_PI428871386	ENST00000549804.2	-0.84	6.49	2.27E-04	1.98E-03	antisense
A_23_P31116	ACOT13	1.26	10.87	2.28E-04	1.98E-03	protein_coding
A_33_P3308914	CIB2	1.15	8.57	2.28E-04	1.99E-03	protein_coding
CUST_10173_PI428871386	ENST00000501050.1	1.15	4.91	2.29E-04	1.99E-03	lincRNA
CUST_33640_PI428871386	ENST00000564901.1	0.86	5.52	2.29E-04	1.99E-03	antisense
CUST_34866_PI428871386	ENST00000573601.1	-1.15	6.10	2.29E-04	1.99E-03	antisense
A_33_P3252043	P4HB	1.01	14.63	2.29E-04	1.99E-03	protein_coding
CUST_11088_PI428871386	ENST00000491608.2	-0.97	6.88	2.29E-04	1.99E-03	lincRNA
A_33_P3209096	CD58	-1.21	7.95	2.29E-04	1.99E-03	protein_coding
CUST_22648_PI428871386	ENST00000414264.1	-1.24	5.45	2.30E-04	2.00E-03	lincRNA
CUST_22146_PI428871386	ENST00000456355.1	-0.86	4.66	2.32E-04	2.02E-03	antisense
A_33_P3212782	CALM2	-0.99	12.41	2.32E-04	2.02E-03	protein_coding
CUST_31270_PI428871386	ENST00000560886.1	-0.86	4.69	2.33E-04	2.02E-03	lincRNA
A_33_P3306948	LRP6	-1.10	9.52	2.33E-04	2.03E-03	protein_coding
CUST_39681_PI428871386	ENST00000587477.1	1.27	6.79	2.33E-04	2.03E-03	lincRNA
A_33_P3256347	TIMM8B	1.08	12.45	2.34E-04	2.04E-03	protein_coding
A_33_P3389728	NR5A2	-1.61	6.89	2.35E-04	2.04E-03	protein_coding
CUST_20053_PI428871386	ENST00000524275.1	-0.97	4.95	2.37E-04	2.06E-03	lincRNA
A_33_P3531828	LARS	0.90	10.20	2.38E-04	2.06E-03	protein_coding
A_33_P3228375	XPO5	0.97	5.52	2.38E-04	2.07E-03	protein_coding
A_23_P113417	C17orf70	0.92	8.59	2.39E-04	2.07E-03	protein_coding
CUST_14863_PI428871386	ENST00000432751.1	1.53	6.73	2.39E-04	2.08E-03	antisense
CUST_30952_PI428871386	ENST00000460164.1	1.74	13.90	2.40E-04	2.09E-03	lincRNA
A_33_P3389689	LEPRE1	1.04	5.66	2.41E-04	2.09E-03	protein_coding
CUST_8295_PI428871386	ENST00000474795.1	0.81	5.40	2.41E-04	2.09E-03	antisense
CUST_39896_PI428871386	ENST00000590369.1	1.27	5.26	2.41E-04	2.09E-03	antisense
CUST_34522_PI428871386	ENST00000563750.1	1.04	6.39	2.41E-04	2.09E-03	lincRNA

A_24_P228796	GAGE7	2.37	6.10	2.42E-04	2.10E-03	protein_coding
CUST_17707_PI428871386	ENST00000433079.1	-1.38	8.81	2.42E-04	2.10E-03	antisense
A_23_P54996	TEX14	-1.18	5.55	2.44E-04	2.11E-03	protein_coding
A_33_P3329444	MAMSTR	1.17	6.97	2.44E-04	2.11E-03	protein_coding
CUST_9038_PI428871386	ENST00000597953.1	1.15	6.46	2.45E-04	2.12E-03	lincRNA
A_23_P357104	ANXA6	-1.30	9.59	2.45E-04	2.12E-03	protein_coding
CUST_18708_PI428871386	ENST00000522718.1	1.18	5.21	2.47E-04	2.14E-03	lincRNA
A_33_P3445679	FLJ42392	0.94	6.03	2.48E-04	2.15E-03	lincRNA
CUST_23742_PI428871386	ENST00000527620.1	-1.48	7.54	2.49E-04	2.15E-03	antisense
CUST_30134_PI428871386	ENST00000556301.1	1.54	5.83	2.49E-04	2.15E-03	antisense
CUST_8916_PI428871386	ENST00000505721.1	-0.80	4.75	2.50E-04	2.16E-03	antisense
A_23_P8906	LRP12	1.57	8.26	2.51E-04	2.17E-03	protein_coding
CUST_6171_PI428871386	ENST00000594219.1	0.87	4.98	2.51E-04	2.17E-03	antisense
A_23_P16110	OR7E24	-1.02	8.07	2.52E-04	2.18E-03	protein_coding
CUST_39695_PI428871386	ENST00000588845.1	1.60	6.22	2.52E-04	2.18E-03	antisense
CUST_6115_PI428871386	ENST00000446213.2	1.07	4.97	2.53E-04	2.19E-03	antisense
A_24_P245246	PIP4K2B	-0.98	10.83	2.53E-04	2.19E-03	protein_coding
A_24_P400376	CHCHD2	1.28	11.31	2.54E-04	2.19E-03	protein_coding
A_23_P163567	SMPD3	-1.78	7.97	2.54E-04	2.19E-03	protein_coding
A_33_P3410724	SDHC	1.02	11.14	2.54E-04	2.19E-03	protein_coding
A_33_P3390441	KPNB1	0.90	12.12	2.54E-04	2.20E-03	protein_coding
CUST_6684_PI428871386	ENST00000428541.1	1.01	5.91	2.55E-04	2.20E-03	antisense
A_23_P141044	ZNF688	-0.93	9.29	2.55E-04	2.20E-03	protein_coding
A_23_P333705	NEK3	-1.02	7.41	2.55E-04	2.20E-03	protein_coding
CUST_26194_PI428871386	ENST00000537659.1	-1.45	7.43	2.56E-04	2.21E-03	lincRNA
CUST_27738_PI428871386	ENST00000531024.1	-0.87	5.57	2.58E-04	2.22E-03	antisense
CUST_33367_PI428871386	ENST00000563613.1	-0.84	4.72	2.58E-04	2.22E-03	lincRNA
A_33_P3294237	MTF1	-0.93	8.33	2.58E-04	2.23E-03	protein_coding
CUST_30708_PI428871386	ENST00000424076.3	-1.61	6.77	2.58E-04	2.23E-03	lincRNA
A_23_P33914	NUP62CL	1.19	5.91	2.59E-04	2.23E-03	protein_coding
CUST_35708_PI428871386	ENST00000563063.1	-0.85	4.84	2.59E-04	2.24E-03	lincRNA
A_23_P119130	RPS19	1.09	14.88	2.59E-04	2.24E-03	protein_coding
CUST_40430_PI428871386	ENST00000599728.1	-0.80	5.74	2.60E-04	2.24E-03	antisense
CUST_17422_PI428871386	ENST00000450686.1	-0.96	5.60	2.61E-04	2.25E-03	antisense
CUST_26231_PI428871386	ENST00000538219.1	-0.90	5.63	2.61E-04	2.25E-03	lincRNA
CUST_21578_PI428871386	ENST00000586374.1	0.84	5.62	2.62E-04	2.25E-03	antisense
CUST_4718_PI428871386	ENST00000413828.2	-1.12	5.86	2.62E-04	2.26E-03	antisense
A_24_P583040	C17orf67	1.09	6.08	2.62E-04	2.26E-03	protein_coding
CUST_5861_PI428871386	ENST00000451884.1	1.04	6.70	2.63E-04	2.26E-03	lincRNA
CUST_37271_PI428871386	ENST00000577023.1	0.97	5.79	2.63E-04	2.27E-03	antisense
A_23_P28834	PHACTR3	-1.74	6.80	2.64E-04	2.27E-03	protein_coding
A_33_P3399267	IL15RA	-0.94	5.97	2.65E-04	2.28E-03	protein_coding
CUST_26868_PI428871386	ENST00000512916.2	1.71	5.91	2.65E-04	2.28E-03	antisense
A_23_P154784	BPIFB1	-2.81	9.06	2.65E-04	2.28E-03	protein_coding
CUST_19704_PI428871386	ENST00000520433.1	0.94	4.97	2.66E-04	2.29E-03	antisense
CUST_25187_PI428871386	ENST00000513207.2	-0.90	5.45	2.66E-04	2.29E-03	lincRNA
CUST_20787_PI428871386	ENST00000438072.1	0.78	4.95	2.68E-04	2.30E-03	lincRNA
A_33_P3313401	CYCS	1.25	10.10	2.69E-04	2.31E-03	protein_coding
CUST_8875_PI428871386	ENST00000514145.1	-0.89	5.15	2.69E-04	2.31E-03	lincRNA
CUST_5785_PI428871386	ENST00000567491.1	-1.02	5.43	2.69E-04	2.31E-03	lincRNA
A_23_P102058	MATN3	-1.54	6.26	2.69E-04	2.31E-03	protein_coding
CUST_41542_PI428871386	ENST00000416002.1	0.84	4.97	2.70E-04	2.32E-03	lincRNA
A_24_P289208	TFF3	-1.61	7.71	2.71E-04	2.33E-03	protein_coding
CUST_24168_PI428871386	ENST00000531304.1	0.78	4.85	2.71E-04	2.33E-03	antisense
CUST_39967_PI428871386	ENST00000599817.1	1.49	5.33	2.72E-04	2.33E-03	lincRNA
A_24_P323104	DIAPH1	-1.00	12.63	2.73E-04	2.35E-03	protein_coding
A_23_P218131	INF2	1.12	8.49	2.73E-04	2.35E-03	protein_coding
A_23_P218412	SPAG7	-1.01	9.10	2.74E-04	2.35E-03	protein_coding
CUST_30035_PI428871386	ENST00000554254.1	1.10	5.56	2.74E-04	2.35E-03	lincRNA
CUST_5291_PI428871386	ENST00000596573.1	0.82	5.09	2.75E-04	2.36E-03	antisense
CUST_826_PI428871386	ENST00000419993.1	-0.80	6.03	2.75E-04	2.36E-03	antisense
A_33_P3333078	ARID1B	-1.04	7.44	2.76E-04	2.36E-03	protein_coding
A_24_P128205	PSMD1	0.88	9.51	2.76E-04	2.37E-03	protein_coding

CUST_33571_PI428871386	ENST00000563806.1	-1.59	7.81	2.76E-04	2.37E-03	antisense
A_23_P169819	EPHA3	-1.07	5.57	2.76E-04	2.37E-03	protein_coding
CUST_21922_PI428871386	ENST00000445932.1	-0.88	5.43	2.77E-04	2.37E-03	lincRNA
A_33_P3283611	IFIT3	-1.81	9.83	2.77E-04	2.38E-03	protein_coding
A_23_P130149	ENO3	1.39	8.11	2.77E-04	2.38E-03	protein_coding
CUST_4968_PI428871386	ENST00000432793.1	-0.98	5.05	2.78E-04	2.38E-03	antisense
A_23_P162037	ARNTL	-1.33	8.36	2.79E-04	2.39E-03	protein_coding
CUST_26584_PI428871386	ENST00000457989.1	-0.91	5.32	2.79E-04	2.39E-03	lincRNA
A_23_P155147	ZBED4	1.03	7.33	2.79E-04	2.39E-03	protein_coding
A_33_P3502037	CHORDC1	1.04	6.99	2.79E-04	2.39E-03	protein_coding
CUST_32848_PI428871386	ENST00000567820.1	-0.86	5.13	2.79E-04	2.39E-03	antisense
A_24_P203622	DOCK7	0.97	7.15	2.80E-04	2.40E-03	protein_coding
A_32_P163533	ZNF322	1.60	9.18	2.80E-04	2.40E-03	protein_coding
A_24_P225534	RHBDL2	1.26	5.86	2.81E-04	2.40E-03	protein_coding
CUST_19722_PI428871386	ENST00000521369.2	1.66	6.48	2.81E-04	2.40E-03	lincRNA
A_23_P24774	ABCC8	-1.59	6.32	2.81E-04	2.40E-03	protein_coding
A_33_P3737504	DAPK1	-0.94	5.88	2.81E-04	2.41E-03	protein_coding
CUST_24810_PI428871386	ENST00000532454.1	0.78	4.89	2.82E-04	2.41E-03	antisense
A_33_P3379106	LOC283867	-1.06	5.52	2.83E-04	2.42E-03	lincRNA
CUST_8940_PI428871386	ENST00000470236.1	1.14	7.10	2.83E-04	2.42E-03	antisense
A_24_P938614	CDS1	-1.18	9.10	2.83E-04	2.42E-03	protein_coding
CUST_42998_PI428871386	ENST00000432502.1	1.36	7.68	2.83E-04	2.42E-03	lincRNA
A_23_P468	C1orf114	-1.51	6.68	2.84E-04	2.43E-03	protein_coding
A_24_P68019	ZNF551	0.99	7.03	2.85E-04	2.43E-03	protein_coding
A_33_P3264224	CDC42BPG	1.06	6.16	2.85E-04	2.43E-03	protein_coding
A_23_P145718	AOAH	-1.33	7.08	2.85E-04	2.44E-03	protein_coding
CUST_41862_PI428871386	ENST00000413862.1	-1.03	5.74	2.86E-04	2.44E-03	antisense
CUST_40337_PI428871386	ENST00000591172.1	-0.94	5.36	2.86E-04	2.45E-03	lincRNA
A_33_P3288844	IL6R	-1.35	8.15	2.87E-04	2.45E-03	protein_coding
A_33_P3295550	TET1	1.98	8.38	2.87E-04	2.45E-03	protein_coding
CUST_20207_PI428871386	ENST00000521207.1	1.06	5.49	2.89E-04	2.46E-03	antisense
A_33_P3217322	ENST00000368586	-0.95	5.03	2.90E-04	2.47E-03	protein_coding
CUST_27184_PI428871386	ENST00000553135.1	1.09	7.74	2.91E-04	2.48E-03	antisense
CUST_23745_PI428871386	ENST00000527113.1	-1.31	7.45	2.91E-04	2.48E-03	antisense
CUST_25195_PI428871386	ENST00000534593.1	0.81	4.98	2.92E-04	2.49E-03	lincRNA
CUST_21950_PI428871386	ENST00000418372.1	-1.03	5.67	2.92E-04	2.49E-03	lincRNA
CUST_39937_PI428871386	ENST00000589594.1	0.80	5.49	2.93E-04	2.49E-03	lincRNA
CUST_36209_PI428871386	ENST00000253803.2	0.92	6.37	2.93E-04	2.50E-03	protein_coding
A_23_P166929	SERPINI1	1.18	6.39	2.93E-04	2.50E-03	protein_coding
CUST_36351_PI428871386	ENST00000464382.2	-0.80	5.14	2.93E-04	2.50E-03	antisense
A_23_P149099	DDOST	0.83	11.28	2.93E-04	2.50E-03	protein_coding
CUST_13025_PI428871386	ENST00000514158.1	-0.82	5.10	2.93E-04	2.50E-03	antisense
A_24_P371962	AMD1	-0.98	11.54	2.94E-04	2.50E-03	protein_coding
A_24_P394420	SFXN1	0.91	5.20	2.94E-04	2.50E-03	protein_coding
CUST_1737_PI428871386	ENST00000564063.1	-1.06	6.39	2.94E-04	2.50E-03	antisense
A_24_P288954	SRRD	1.03	7.48	2.94E-04	2.50E-03	protein_coding
CUST_18248_PI428871386	ENST00000519147.1	0.87	5.12	2.94E-04	2.50E-03	lincRNA
CUST_8930_PI428871386	ENST00000459861.1	1.28	5.06	2.94E-04	2.50E-03	lincRNA
CUST_26669_PI428871386	ENST00000552320.1	0.82	5.18	2.96E-04	2.52E-03	lincRNA
CUST_17378_PI428871386	ENST00000419422.1	1.23	5.27	2.96E-04	2.52E-03	antisense
CUST_37270_PI428871386	ENST00000577023.1	1.15	5.94	2.96E-04	2.52E-03	antisense
A_33_P3401322	ISG20L2	1.05	8.61	2.97E-04	2.53E-03	protein_coding
A_23_P216361	COL14A1	-1.82	7.34	2.97E-04	2.53E-03	protein_coding
A_24_P417935	LOC100130776	0.90	6.44	2.97E-04	2.53E-03	antisense
CUST_23642_PI428871386	ENST00000414581.1	0.78	4.92	2.99E-04	2.54E-03	lincRNA
A_23_P376239	PAPOLA	1.03	9.70	2.99E-04	2.54E-03	protein_coding
A_32_P184727	KPNB1	0.91	12.37	2.99E-04	2.54E-03	protein_coding
CUST_17047_PI428871386	ENST00000445681.1	1.19	7.75	3.01E-04	2.55E-03	lincRNA
CUST_36720_PI428871386	ENST00000582940.1	1.30	7.00	3.01E-04	2.55E-03	lincRNA
A_23_P209904	GPC1	1.47	10.39	3.01E-04	2.55E-03	protein_coding
A_33_P3291092	THSD1	-1.04	5.45	3.01E-04	2.56E-03	protein_coding
CUST_14859_PI428871386	ENST00000454812.1	1.53	6.99	3.01E-04	2.56E-03	antisense
A_23_P408787	C1orf150	-0.83	4.82	3.02E-04	2.57E-03	protein_coding

CUST_19715_PI428871386	ENST00000521622.1	1.08	4.94	3.03E-04	2.57E-03	antisense
CUST_43842_PI428871386	ENST00000399966.4	1.37	5.19	3.03E-04	2.57E-03	antisense
CUST_17893_PI428871386	ENST00000470435.1	-0.92	6.93	3.04E-04	2.58E-03	antisense
A_23_P147397	DYNC2H1	-1.21	7.44	3.04E-04	2.58E-03	protein_coding
CUST_7137_PI428871386	ENST00000440574.1	1.15	6.91	3.04E-04	2.58E-03	antisense
A_24_P110062	PTPMT1	-0.92	7.96	3.05E-04	2.58E-03	protein_coding
CUST_17046_PI428871386	ENST00000445681.1	1.18	7.98	3.05E-04	2.59E-03	lincRNA
A_23_P431381	C14orf80	1.13	7.58	3.07E-04	2.60E-03	protein_coding
CUST_26507_PI428871386	ENST00000538113.1	1.31	5.45	3.08E-04	2.61E-03	antisense
A_33_P3300312	DMBT1	-2.06	7.75	3.10E-04	2.62E-03	protein_coding
A_33_P3400943	C16orf5	-1.25	9.66	3.12E-04	2.64E-03	protein_coding
CUST_525_PI428871386	ENST00000431027.1	1.34	5.27	3.12E-04	2.64E-03	lincRNA
A_33_P3306504	ISYNA1	1.36	7.98	3.13E-04	2.65E-03	protein_coding
CUST_5826_PI428871386	ENST00000562613.1	-1.52	7.41	3.13E-04	2.65E-03	lincRNA
A_33_P3254412	ULK2	-1.03	7.43	3.14E-04	2.66E-03	protein_coding
CUST_41080_PI428871386	ENST00000430481.1	-0.83	4.85	3.15E-04	2.67E-03	antisense
A_33_P3298413	PPIH	1.02	9.23	3.16E-04	2.68E-03	protein_coding
CUST_6956_PI428871386	ENST00000432711.1	-1.20	5.75	3.17E-04	2.68E-03	lincRNA
A_24_P419039	PCDH19	1.50	6.38	3.17E-04	2.68E-03	protein_coding
CUST_6734_PI428871386	ENST00000456053.1	-1.09	7.03	3.18E-04	2.69E-03	antisense
CUST_22592_PI428871386	ENST00000443374.1	-0.88	7.26	3.19E-04	2.70E-03	antisense
CUST_17537_PI428871386	ENST00000456775.1	-0.91	5.13	3.19E-04	2.70E-03	antisense
CUST_9722_PI428871386	ENST00000444085.1	0.86	5.48	3.19E-04	2.70E-03	antisense
CUST_273_PI428871386	ENST00000418088.1	-1.00	6.32	3.19E-04	2.70E-03	antisense
CUST_8303_PI428871386	ENST00000495542.1	1.03	5.15	3.20E-04	2.70E-03	antisense
A_23_P169097	WISP1	1.13	5.26	3.20E-04	2.71E-03	protein_coding
CUST_34879_PI428871386	ENST00000570974.1	-0.74	6.20	3.20E-04	2.71E-03	antisense
A_24_P701776	ARHGEF35	1.46	8.57	3.22E-04	2.72E-03	protein_coding
CUST_4817_PI428871386	ENST00000451514.1	-0.81	4.85	3.23E-04	2.73E-03	antisense
A_23_P76364	CD9	-1.36	10.77	3.23E-04	2.73E-03	protein_coding
CUST_6323_PI428871386	ENST00000596970.1	0.97	5.30	3.24E-04	2.73E-03	lincRNA
CUST_24872_PI428871386	ENST00000529069.1	-1.01	6.48	3.24E-04	2.74E-03	lincRNA
A_23_P97195	FAM54B	-0.90	7.71	3.25E-04	2.74E-03	protein_coding
CUST_17498_PI428871386	ENST00000419905.1	-0.77	4.65	3.26E-04	2.75E-03	lincRNA
A_23_P35316	ZNF695	1.00	5.32	3.27E-04	2.76E-03	protein_coding
A_24_P285623	DGUOK	0.88	10.10	3.28E-04	2.77E-03	protein_coding
A_33_P3272921	ARID3A	1.37	8.30	3.30E-04	2.78E-03	protein_coding
A_23_P419213	KIAA1407	-1.07	6.49	3.32E-04	2.80E-03	protein_coding
A_23_P218892	EIF4G1	1.03	10.10	3.33E-04	2.81E-03	protein_coding
A_23_P325562	SLC1A7	-2.16	7.43	3.34E-04	2.81E-03	protein_coding
A_33_P3293266	TMEM175	-1.01	10.17	3.34E-04	2.82E-03	protein_coding
CUST_43001_PI428871386	ENST00000422971.1	1.36	5.44	3.35E-04	2.82E-03	antisense
A_32_P133670	ANP32A	0.89	7.13	3.35E-04	2.82E-03	protein_coding
A_23_P421423	TNFAIP2	-1.66	12.50	3.35E-04	2.82E-03	protein_coding
A_24_P174503	AMT	-1.06	6.56	3.36E-04	2.83E-03	protein_coding
A_33_P3210488	COL6A3	1.64	8.25	3.39E-04	2.85E-03	protein_coding
A_24_P157424	NCBP2	1.06	9.07	3.39E-04	2.85E-03	protein_coding
CUST_43770_PI428871386	ENST00000450989.1	-0.92	5.68	3.39E-04	2.86E-03	antisense
A_33_P3368139	MAP3K1	-0.98	10.97	3.39E-04	2.86E-03	protein_coding
CUST_1532_PI428871386	ENST00000436200.1	-0.98	12.18	3.41E-04	2.87E-03	lincRNA
A_23_P306987	SOX7	-1.27	5.94	3.42E-04	2.88E-03	protein_coding
CUST_16608_PI428871386	ENST00000519694.1	1.52	6.14	3.42E-04	2.88E-03	antisense
A_23_P217968	SUV420H1	1.31	7.42	3.42E-04	2.88E-03	protein_coding
CUST_15111_PI428871386	ENST00000586030.1	-0.88	6.62	3.43E-04	2.88E-03	antisense
A_33_P3395976	CTU1	0.86	10.14	3.43E-04	2.88E-03	protein_coding
A_33_P3364268	LBH	-1.20	8.08	3.44E-04	2.89E-03	protein_coding
CUST_27694_PI428871386	ENST00000443596.1	-0.89	7.95	3.44E-04	2.89E-03	lincRNA
CUST_23744_PI428871386	ENST00000527113.1	-1.32	7.33	3.45E-04	2.90E-03	antisense
CUST_1787_PI428871386	ENST00000440688.1	-0.82	6.88	3.45E-04	2.90E-03	antisense
A_33_P3214466	MESP1	1.65	7.57	3.45E-04	2.90E-03	protein_coding
A_33_P3233649	NADKD1	1.19	7.99	3.46E-04	2.91E-03	protein_coding
CUST_5493_PI428871386	ENST00000448734.1	-0.78	4.69	3.47E-04	2.91E-03	antisense
A_24_P280983	HOXA11-AS1	1.91	5.31	3.48E-04	2.92E-03	antisense

A_23_P255523	ALKBH4	0.88	7.70	3.48E-04	2.92E-03	protein_coding
A_33_P3277447	SLC26A2	-1.45	9.76	3.49E-04	2.92E-03	protein_coding
A_23_P202170	MGEA5	-0.90	12.06	3.49E-04	2.93E-03	protein_coding
CUST_37476_PI428871386	ENST00000577439.1	-0.94	6.62	3.49E-04	2.93E-03	lincRNA
A_23_P48561	EFS	1.49	10.48	3.50E-04	2.93E-03	protein_coding
A_33_P3386716	PPP1R15B	-1.18	11.20	3.50E-04	2.94E-03	protein_coding
A_23_P202334	FGFR2	-1.68	9.25	3.50E-04	2.94E-03	protein_coding
CUST_18939_PI428871386	ENST00000518556.1	0.84	5.34	3.51E-04	2.94E-03	lincRNA
A_24_P917833	TMED10	-1.01	10.64	3.51E-04	2.94E-03	protein_coding
A_24_P919899	ATPAF1	-0.83	5.36	3.52E-04	2.95E-03	protein_coding
A_33_P3317664	MGAT1	-1.24	10.04	3.52E-04	2.95E-03	protein_coding
CUST_33303_PI428871386	ENST00000569858.1	-0.94	6.30	3.53E-04	2.95E-03	antisense
A_23_P317683	TRAPPC10	-0.89	8.70	3.53E-04	2.95E-03	protein_coding
CUST_14742_PI428871386	ENST00000413039.1	-1.10	9.46	3.54E-04	2.97E-03	lincRNA
A_23_P340251	RAB2A	0.99	11.27	3.56E-04	2.98E-03	protein_coding
CUST_17460_PI428871386	ENST00000440971.1	-0.81	4.71	3.57E-04	2.99E-03	antisense
CUST_29901_PI428871386	ENST00000556417.1	1.09	5.30	3.59E-04	3.01E-03	lincRNA
CUST_39561_PI428871386	ENST00000588286.1	1.09	5.43	3.60E-04	3.01E-03	antisense
CUST_11216_PI428871386	ENST00000514222.1	0.79	5.00	3.60E-04	3.01E-03	lincRNA
CUST_6921_PI428871386	ENST00000594023.1	-0.81	5.36	3.60E-04	3.01E-03	antisense
CUST_526_PI428871386	ENST00000431027.1	1.24	5.29	3.60E-04	3.01E-03	lincRNA
CUST_35027_PI428871386	ENST00000413077.1	1.24	8.45	3.61E-04	3.02E-03	antisense
CUST_38785_PI428871386	ENST00000597407.1	-0.89	8.23	3.62E-04	3.03E-03	antisense
A_23_P61810	BAIAP2	-1.18	8.02	3.62E-04	3.03E-03	protein_coding
A_23_P146274	STMN2	1.60	5.97	3.63E-04	3.03E-03	protein_coding
A_24_P316257	NHLRC4	-1.07	6.52	3.63E-04	3.03E-03	protein_coding
CUST_23747_PI428871386	ENST00000533844.1	-1.35	7.54	3.64E-04	3.04E-03	antisense
A_33_P3396120	ZNF594	1.00	6.08	3.64E-04	3.04E-03	protein_coding
A_23_P112512	MCART1	1.14	6.99	3.65E-04	3.04E-03	protein_coding
A_24_P338788	CSNK1A1L	0.92	7.70	3.66E-04	3.05E-03	protein_coding
A_23_P207319	MAP3K14	-1.04	8.24	3.66E-04	3.06E-03	protein_coding
CUST_8173_PI428871386	ENST00000426302.1	-0.87	6.17	3.67E-04	3.07E-03	lincRNA
A_33_P3291636	ZNF74	1.12	6.54	3.68E-04	3.07E-03	protein_coding
A_23_P410998	RAB5B	-0.83	8.17	3.69E-04	3.08E-03	protein_coding
A_24_P152968	AKR1C1	2.44	9.26	3.69E-04	3.08E-03	protein_coding
CUST_13784_PI428871386	ENST00000412431.2	-0.94	8.57	3.72E-04	3.10E-03	protein_coding
A_24_P388570	MINA	1.00	7.01	3.74E-04	3.12E-03	protein_coding
CUST_14858_PI428871386	ENST00000454812.1	1.54	7.02	3.74E-04	3.12E-03	antisense
CUST_24944_PI428871386	ENST00000561588.1	1.03	5.95	3.75E-04	3.12E-03	lincRNA
CUST_26715_PI428871386	ENST00000552284.1	0.97	5.55	3.76E-04	3.13E-03	antisense
CUST_1025_PI428871386	ENST00000591675.1	-0.86	5.56	3.76E-04	3.13E-03	antisense
CUST_21652_PI428871386	ENST00000423918.1	-1.03	8.75	3.78E-04	3.15E-03	lincRNA
CUST_14794_PI428871386	ENST00000499560.2	1.05	5.41	3.78E-04	3.15E-03	antisense
CUST_14834_PI428871386	ENST00000448433.1	-0.90	7.61	3.78E-04	3.15E-03	antisense
CUST_40383_PI428871386	ENST00000602145.1	-0.94	10.35	3.78E-04	3.15E-03	lincRNA
CUST_37617_PI428871386	ENST00000586474.1	0.80	5.56	3.80E-04	3.16E-03	antisense
A_24_P28295	RABGAP1L	-1.14	9.12	3.82E-04	3.18E-03	protein_coding
CUST_19792_PI428871386	ENST00000531508.1	1.72	8.98	3.83E-04	3.18E-03	protein_coding
CUST_9039_PI428871386	ENST00000597953.1	1.13	6.72	3.83E-04	3.19E-03	lincRNA
A_24_P246841	SLC25A27	-1.05	6.35	3.84E-04	3.19E-03	protein_coding
CUST_26449_PI428871386	ENST00000500276.2	-1.06	7.49	3.85E-04	3.20E-03	antisense
A_23_P45851	HIAT1	-0.88	10.26	3.85E-04	3.20E-03	protein_coding
CUST_15842_PI428871386	ENST00000565399.1	0.79	4.95	3.86E-04	3.21E-03	lincRNA
A_23_P4798	ZNF581	1.23	7.59	3.87E-04	3.21E-03	protein_coding
A_33_P3314146	MINA	0.92	6.50	3.88E-04	3.22E-03	protein_coding
A_24_P122337	SYTL4	-1.25	6.74	3.88E-04	3.22E-03	protein_coding
A_23_P314222	LEO1	0.92	8.59	3.88E-04	3.22E-03	protein_coding
CUST_41155_PI428871386	ENST00000326677.5	1.14	12.32	3.89E-04	3.23E-03	antisense
CUST_7726_PI428871386	ENST00000524210.1	-1.10	7.02	3.89E-04	3.23E-03	antisense
A_33_P3417260	C9orf95	-1.21	8.85	3.90E-04	3.23E-03	protein_coding
A_24_P168398	ZNF177	-1.17	6.62	3.91E-04	3.25E-03	protein_coding
CUST_37813_PI428871386	ENST00000578701.1	-1.04	5.21	3.92E-04	3.25E-03	antisense
A_23_P4144	COASY	0.89	9.83	3.92E-04	3.25E-03	protein_coding

A_32_P150030	PPM1D	-1.05	8.13	3.94E-04	3.27E-03	protein_coding
A_23_P353574	NEK7	-0.86	6.58	3.96E-04	3.28E-03	protein_coding
CUST_43309_PI428871386	ENST00000456532.1	1.19	5.30	3.97E-04	3.29E-03	lincRNA
CUST_19705_PI428871386	ENST00000524045.1	1.60	6.42	3.97E-04	3.29E-03	antisense
A_23_P86504	C10orf76	-0.82	9.16	3.97E-04	3.29E-03	protein_coding
A_33_P3419720	MLH1	-0.84	11.37	3.97E-04	3.29E-03	protein_coding
CUST_43110_PI428871386	ENST00000438107.1	1.01	6.27	3.97E-04	3.29E-03	lincRNA
CUST_2703_PI428871386	ENST00000526176.1	0.99	5.93	3.98E-04	3.29E-03	antisense
A_33_P3273272	ZNF701	0.92	6.47	3.99E-04	3.30E-03	protein_coding
A_23_P66481	RTN4RL1	-1.31	6.44	3.99E-04	3.30E-03	protein_coding
CUST_7696_PI428871386	ENST00000517846.1	0.94	6.42	3.99E-04	3.30E-03	antisense
CUST_9067_PI428871386	ENST00000490375.1	1.15	5.88	4.00E-04	3.31E-03	antisense
A_24_P612446	C6orf89	-0.91	6.71	4.00E-04	3.31E-03	protein_coding
CUST_31189_PI428871386	ENST00000557989.1	-0.88	5.55	4.00E-04	3.31E-03	antisense
A_23_P129101	HEXA	-1.05	10.28	4.01E-04	3.32E-03	protein_coding
A_24_P362572	BZW1	1.11	9.70	4.02E-04	3.32E-03	protein_coding
A_23_P213137	LNX1	1.47	6.46	4.03E-04	3.33E-03	protein_coding
A_23_P52914	OR4C15	-0.78	5.23	4.03E-04	3.33E-03	protein_coding
CUST_10750_PI428871386	ENST00000505149.1	-0.99	5.31	4.04E-04	3.34E-03	antisense
CUST_16347_PI428871386	ENST00000451066.1	-0.82	5.91	4.04E-04	3.34E-03	antisense
A_23_P75867	OR10A4	-0.82	4.84	4.04E-04	3.34E-03	protein_coding
A_24_P277807	SNX3	-1.05	10.54	4.05E-04	3.35E-03	protein_coding
CUST_14830_PI428871386	ENST00000451810.1	-0.90	7.63	4.06E-04	3.36E-03	antisense
CUST_33336_PI428871386	ENST00000569048.1	1.02	5.76	4.07E-04	3.36E-03	lincRNA
CUST_29520_PI428871386	ENST00000552826.1	1.27	5.07	4.07E-04	3.36E-03	antisense
A_33_P3392525	ARL4D	1.49	7.57	4.07E-04	3.36E-03	protein_coding
A_33_P3222424	CSF3	-1.44	5.94	4.09E-04	3.37E-03	protein_coding
A_24_P138713	HEATR3	0.97	7.18	4.09E-04	3.38E-03	protein_coding
A_24_P273489	SH2D4B	-0.87	5.15	4.09E-04	3.38E-03	protein_coding
A_23_P52101	CYB5R1	-1.09	10.51	4.10E-04	3.38E-03	protein_coding
CUST_40428_PI428871386	ENST00000593374.1	-0.84	5.00	4.11E-04	3.39E-03	antisense
CUST_10803_PI428871386	ENST00000509007.1	0.99	6.06	4.11E-04	3.39E-03	antisense
A_23_P155376	CRELD1	-1.16	8.75	4.12E-04	3.40E-03	protein_coding
A_33_P3356910	TCEAL6	-1.18	10.16	4.12E-04	3.40E-03	protein_coding
CUST_23660_PI428871386	ENST00000456581.1	-0.81	6.16	4.13E-04	3.40E-03	lincRNA
CUST_10740_PI428871386	ENST00000507476.1	-1.01	5.27	4.13E-04	3.41E-03	antisense
CUST_28689_PI428871386	ENST00000235290.3	1.23	7.55	4.13E-04	3.41E-03	antisense
CUST_7920_PI428871386	ENST00000438096.1	0.76	4.92	4.14E-04	3.41E-03	lincRNA
A_23_P356494	SPINK5	-1.73	8.55	4.15E-04	3.42E-03	protein_coding
CUST_1875_PI428871386	ENST00000422022.1	1.15	5.92	4.17E-04	3.43E-03	lincRNA
CUST_16414_PI428871386	ENST00000425322.1	-0.77	4.59	4.18E-04	3.44E-03	lincRNA
CUST_27254_PI428871386	ENST00000515416.2	1.26	5.13	4.18E-04	3.44E-03	lincRNA
A_32_P144421	ZNF518B	1.28	8.05	4.18E-04	3.45E-03	protein_coding
CUST_26668_PI428871386	ENST00000552320.1	0.83	5.24	4.19E-04	3.45E-03	lincRNA
CUST_10172_PI428871386	ENST00000501050.1	0.87	4.97	4.20E-04	3.46E-03	lincRNA
A_23_P47616	FOLH1	1.75	7.36	4.20E-04	3.46E-03	protein_coding
A_24_P280762	KHDC1	1.26	5.90	4.21E-04	3.47E-03	protein_coding
CUST_11961_PI428871386	ENST00000399869.1	-0.95	5.30	4.21E-04	3.47E-03	antisense
A_33_P3341189	UBE2A	1.04	9.80	4.22E-04	3.47E-03	protein_coding
CUST_30578_PI428871386	ENST00000553445.1	1.40	7.38	4.22E-04	3.47E-03	antisense
CUST_32646_PI428871386	ENST00000560395.1	-1.23	6.56	4.24E-04	3.49E-03	antisense
CUST_13534_PI428871386	ENST00000422204.1	-0.88	4.86	4.24E-04	3.49E-03	antisense
CUST_32808_PI428871386	ENST00000527434.1	1.31	5.29	4.25E-04	3.49E-03	lincRNA
CUST_35488_PI428871386	ENST00000578585.1	-1.29	10.06	4.25E-04	3.50E-03	lincRNA
A_33_P3298128	ITPR2	-0.96	7.16	4.26E-04	3.51E-03	protein_coding
A_33_P3210343	ETV6	0.98	8.17	4.28E-04	3.52E-03	protein_coding
CUST_14216_PI428871386	ENST00000503263.1	1.18	5.70	4.29E-04	3.53E-03	antisense
CUST_26556_PI428871386	ENST00000535163.1	1.01	5.36	4.31E-04	3.54E-03	lincRNA
CUST_10935_PI428871386	ENST00000502883.1	-1.10	6.84	4.31E-04	3.55E-03	lincRNA
A_33_P3272828	RAD1	0.91	7.20	4.32E-04	3.55E-03	protein_coding
CUST_23103_PI428871386	ENST00000423474.1	-1.05	5.33	4.33E-04	3.56E-03	antisense
A_33_P3302957	PLEKHG4	1.46	7.85	4.33E-04	3.56E-03	protein_coding
A_23_P128734	ERH	0.89	12.06	4.35E-04	3.57E-03	protein_coding

CUST_16439_PI428871386	ENST00000448664.1	-0.98	6.71	4.36E-04	3.58E-03	antisense
CUST_26200_PI428871386	ENST00000536560.1	-0.86	5.42	4.37E-04	3.59E-03	lincRNA
CUST_4008_PI428871386	ENST00000439547.1	-1.09	8.03	4.41E-04	3.62E-03	lincRNA
CUST_7209_PI428871386	ENST00000451392.1	-0.81	4.99	4.41E-04	3.62E-03	lincRNA
CUST_24518_PI428871386	ENST00000531719.1	0.92	5.23	4.41E-04	3.62E-03	antisense
A_23_P154972	ZNF280A	1.44	5.59	4.42E-04	3.62E-03	protein_coding
A_23_P83976	CEP112	-1.03	6.86	4.42E-04	3.62E-03	protein_coding
CUST_5915_PI428871386	ENST00000437551.1	-1.55	9.28	4.45E-04	3.65E-03	antisense
A_33_P3226605	PSIP1	-1.24	10.08	4.45E-04	3.65E-03	protein_coding
CUST_34572_PI428871386	ENST00000565714.1	-1.17	5.66	4.47E-04	3.67E-03	antisense
CUST_26043_PI428871386	ENST00000545357.1	-0.89	5.06	4.49E-04	3.68E-03	antisense
A_33_P3847514	C6orf141	1.33	5.46	4.50E-04	3.69E-03	protein_coding
A_24_P288424	SLMO2	1.00	8.35	4.52E-04	3.70E-03	protein_coding
A_32_P171313	GNB4	-1.33	10.63	4.52E-04	3.70E-03	protein_coding
A_24_P11315	OLFML3	-1.56	7.56	4.53E-04	3.71E-03	protein_coding
A_33_P3407266	GTF3C4	0.94	6.34	4.54E-04	3.72E-03	protein_coding
CUST_33277_PI428871386	ENST00000575424.1	-1.09	6.01	4.54E-04	3.72E-03	lincRNA
CUST_11016_PI428871386	ENST00000512637.1	0.86	5.30	4.54E-04	3.72E-03	antisense
A_23_P134078	CDYL	1.03	7.90	4.55E-04	3.73E-03	protein_coding
A_23_P391228	MANEAL	1.68	7.33	4.55E-04	3.73E-03	protein_coding
CUST_32319_PI428871386	ENST00000558153.1	0.88	5.33	4.57E-04	3.74E-03	lincRNA
A_23_P55477	ADORA2B	1.59	8.39	4.58E-04	3.74E-03	protein_coding
CUST_37543_PI428871386	ENST00000580491.1	-0.95	4.87	4.59E-04	3.75E-03	antisense
A_24_P174294	LRRTM4	-0.98	5.00	4.59E-04	3.76E-03	protein_coding
CUST_10790_PI428871386	ENST00000511271.1	1.15	6.96	4.60E-04	3.76E-03	antisense
A_23_P7221	RPL34	-0.99	15.58	4.61E-04	3.76E-03	protein_coding
CUST_24873_PI428871386	ENST00000529069.1	-1.00	6.64	4.65E-04	3.80E-03	lincRNA
CUST_33572_PI428871386	ENST00000563806.1	-1.52	8.11	4.66E-04	3.81E-03	antisense
CUST_21582_PI428871386	ENST00000587355.1	0.81	5.52	4.66E-04	3.81E-03	antisense
CUST_39178_PI428871386	ENST00000593655.1	0.95	5.87	4.67E-04	3.82E-03	antisense
A_33_P3214310	FOXP1	-1.14	8.10	4.68E-04	3.82E-03	protein_coding
CUST_16036_PI428871386	ENST00000601203.1	0.78	4.91	4.68E-04	3.82E-03	antisense
CUST_39618_PI428871386	ENST00000585356.1	1.17	6.69	4.69E-04	3.83E-03	lincRNA
A_24_P159702	LMAN1	1.01	6.71	4.70E-04	3.84E-03	protein_coding
A_32_P57237	RPL21	-1.06	15.18	4.70E-04	3.84E-03	protein_coding
A_33_P3393200	SRRM4	-1.17	5.20	4.73E-04	3.86E-03	protein_coding
CUST_39560_PI428871386	ENST00000588286.1	1.08	5.50	4.75E-04	3.88E-03	antisense
CUST_21047_PI428871386	ENST00000586206.1	-0.83	4.85	4.76E-04	3.88E-03	antisense
A_32_P190416	MAP7	1.10	6.27	4.76E-04	3.88E-03	protein_coding
CUST_43967_PI428871386	ENST00000447937.1	-0.88	4.94	4.76E-04	3.88E-03	lincRNA
CUST_16097_PI428871386	ENST00000568025.1	-1.00	5.40	4.78E-04	3.90E-03	antisense
A_23_P207387	GHDC	-0.94	7.79	4.79E-04	3.90E-03	protein_coding
CUST_14898_PI428871386	ENST00000573382.1	-1.20	6.59	4.79E-04	3.91E-03	lincRNA
CUST_1926_PI428871386	ENST00000430316.1	-0.94	5.34	4.80E-04	3.92E-03	lincRNA
CUST_22571_PI428871386	ENST00000423283.1	1.54	5.43	4.81E-04	3.92E-03	antisense
CUST_16443_PI428871386	ENST00000415246.1	-1.04	6.90	4.81E-04	3.92E-03	antisense
A_23_P125505	PPEF1	1.14	5.30	4.81E-04	3.92E-03	protein_coding
CUST_11865_PI428871386	ENST00000505103.1	-0.74	5.03	4.83E-04	3.93E-03	antisense
A_33_P3336484	NPIP	-1.07	9.49	4.84E-04	3.95E-03	protein_coding
CUST_19384_PI428871386	ENST00000562577.1	0.80	5.13	4.85E-04	3.95E-03	antisense
CUST_16612_PI428871386	ENST00000523790.1	1.13	5.50	4.87E-04	3.96E-03	antisense
A_24_P754817	EFTUD1	0.89	6.02	4.87E-04	3.96E-03	protein_coding
A_32_P25050	RDH10	1.80	8.57	4.87E-04	3.97E-03	protein_coding
A_23_P90453	KRTDAP	2.08	5.93	4.89E-04	3.98E-03	protein_coding
A_24_P42693	CYP4F11	1.69	5.43	4.91E-04	4.00E-03	protein_coding
CUST_23746_PI428871386	ENST00000533844.1	-1.32	7.30	4.91E-04	4.00E-03	antisense
A_33_P3365002	TUBB2A	1.24	8.46	4.92E-04	4.00E-03	protein_coding
CUST_13112_PI428871386	ENST00000513235.1	0.76	4.94	4.92E-04	4.00E-03	antisense
CUST_15173_PI428871386	ENST00000436672.1	1.06	5.91	4.92E-04	4.00E-03	lincRNA
CUST_24813_PI428871386	ENST00000531155.1	0.84	5.05	4.92E-04	4.00E-03	antisense
CUST_27576_PI428871386	ENST00000547179.1	0.77	4.95	4.93E-04	4.01E-03	lincRNA
A_23_P329768	GREB1	1.11	5.62	4.95E-04	4.02E-03	protein_coding
CUST_18835_PI428871386	ENST00000521359.1	-0.90	5.15	4.95E-04	4.02E-03	lincRNA

A_23_P76684	RTN3	-1.01	8.70	4.95E-04	4.02E-03	protein_coding
CUST_38137_PI428871386	ENST00000587904.1	-0.87	4.64	4.95E-04	4.02E-03	lincRNA
A_33_P3239242	SPATA6	-1.02	6.32	4.96E-04	4.02E-03	protein_coding
CUST_15531_PI428871386	ENST00000519104.1	0.82	4.90	4.96E-04	4.02E-03	antisense
A_24_P72394	RBAK	0.92	5.59	4.96E-04	4.02E-03	protein_coding
CUST_18595_PI428871386	ENST00000521908.1	-0.85	4.95	4.96E-04	4.03E-03	lincRNA
A_33_P3307735	OPHN1	-1.05	6.26	4.97E-04	4.03E-03	protein_coding
CUST_43402_PI428871386	ENST00000431103.1	0.98	5.33	4.97E-04	4.03E-03	antisense
A_23_P3014	RNASE6	-1.45	8.78	4.97E-04	4.04E-03	protein_coding
CUST_1794_PI428871386	ENST00000457535.1	1.05	5.91	4.98E-04	4.04E-03	antisense
A_33_P3380263	MTG1	0.95	6.38	4.98E-04	4.04E-03	protein_coding
CUST_11998_PI428871386	ENST00000515086.1	1.08	5.40	4.98E-04	4.04E-03	lincRNA
CUST_4555_PI428871386	ENST00000352271.6	0.95	5.28	5.01E-04	4.06E-03	antisense
CUST_43817_PI428871386	ENST00000562749.1	1.25	7.63	5.02E-04	4.07E-03	antisense
A_33_P3354176	MYOF	-0.83	5.82	5.02E-04	4.07E-03	protein_coding
A_23_P151565	RALGAPA1	-1.15	9.71	5.03E-04	4.08E-03	protein_coding
A_33_P3394833	C3orf72	1.49	5.03	5.06E-04	4.10E-03	protein_coding
CUST_28843_PI428871386	ENST00000456627.1	1.03	5.03	5.07E-04	4.10E-03	lincRNA
CUST_20552_PI428871386	ENST00000522960.1	-0.84	5.12	5.07E-04	4.10E-03	lincRNA
CUST_30200_PI428871386	ENST00000556578.1	-0.87	6.30	5.07E-04	4.11E-03	antisense
CUST_42740_PI428871386	ENST00000563812.1	1.03	7.34	5.08E-04	4.11E-03	antisense
A_23_P168864	ZNF16	0.91	6.86	5.08E-04	4.12E-03	protein_coding
A_23_P72157	MFSD7	-1.15	8.81	5.10E-04	4.13E-03	protein_coding
CUST_13407_PI428871386	ENST00000506859.1	0.92	5.37	5.10E-04	4.13E-03	antisense
A_33_P3842556	IKZF1	-1.36	9.96	5.11E-04	4.14E-03	protein_coding
CUST_31720_PI428871386	ENST00000569661.1	0.76	4.83	5.12E-04	4.14E-03	lincRNA
A_33_P3233659	RPP30	-0.86	6.83	5.12E-04	4.14E-03	protein_coding
CUST_9230_PI428871386	ENST00000463449.1	1.16	5.87	5.17E-04	4.18E-03	antisense
CUST_19283_PI428871386	ENST00000522914.1	0.91	4.94	5.19E-04	4.20E-03	antisense
CUST_24910_PI428871386	ENST00000526897.1	-1.25	7.67	5.19E-04	4.20E-03	antisense
CUST_37002_PI428871386	ENST00000592622.1	0.84	5.51	5.19E-04	4.20E-03	antisense
CUST_30906_PI428871386	ENST00000554954.1	-0.87	6.22	5.22E-04	4.22E-03	lincRNA
A_33_P3282836	RPS28	-0.87	16.29	5.23E-04	4.23E-03	protein_coding
A_24_P393958	DNAJB4	-1.26	7.94	5.24E-04	4.23E-03	protein_coding
CUST_29798_PI428871386	ENST00000557062.1	-0.85	4.78	5.25E-04	4.24E-03	lincRNA
CUST_25299_PI428871386	ENST00000531994.1	1.04	5.22	5.25E-04	4.24E-03	antisense
A_23_P324718	SYNJ1	-0.84	6.44	5.26E-04	4.24E-03	protein_coding
A_33_P3210965	TCTN1	-0.99	10.19	5.28E-04	4.26E-03	protein_coding
CUST_28869_PI428871386	ENST00000452852.1	1.13	4.98	5.28E-04	4.26E-03	lincRNA
A_33_P3236986	ENST00000369884	-0.91	6.92	5.30E-04	4.27E-03	antisense
CUST_1039_PI428871386	ENST00000458151.1	0.99	5.85	5.31E-04	4.29E-03	antisense
CUST_14861_PI428871386	ENST00000439386.1	1.44	6.91	5.32E-04	4.29E-03	antisense
CUST_29348_PI428871386	ENST00000556738.1	-1.05	6.71	5.33E-04	4.30E-03	lincRNA
A_23_P63825	GOT1	1.14	9.65	5.33E-04	4.30E-03	protein_coding
CUST_1240_PI428871386	ENST00000444349.1	-0.84	4.76	5.34E-04	4.31E-03	lincRNA
A_33_P3741022	LINC00511	0.92	5.05	5.35E-04	4.31E-03	lincRNA
A_23_P253158	EP400	-0.85	7.03	5.35E-04	4.32E-03	protein_coding
CUST_16440_PI428871386	ENST00000452249.1	-0.97	6.52	5.39E-04	4.34E-03	antisense
A_24_P34155	RUNX1	1.20	6.60	5.40E-04	4.35E-03	protein_coding
A_23_P49559	GPR142	-0.82	5.01	5.42E-04	4.36E-03	protein_coding
A_33_P3335629	TMEM208	0.99	10.13	5.42E-04	4.36E-03	protein_coding
CUST_43470_PI428871386	ENST00000420327.1	-0.91	5.08	5.42E-04	4.37E-03	lincRNA
A_23_P371613	CHCHD1	0.92	10.74	5.43E-04	4.37E-03	protein_coding
CUST_14117_PI428871386	ENST00000519942.1	0.77	4.97	5.44E-04	4.38E-03	antisense
CUST_41286_PI428871386	ENST00000427794.1	1.37	5.37	5.44E-04	4.38E-03	lincRNA
CUST_20139_PI428871386	ENST00000523604.1	-0.78	5.33	5.45E-04	4.38E-03	antisense
A_23_P41166	B3GALNT1	-1.21	8.15	5.49E-04	4.42E-03	protein_coding
A_24_P49427	KIAA1486	-1.02	5.37	5.49E-04	4.42E-03	protein_coding
CUST_5596_PI428871386	ENST00000594273.1	-0.86	5.37	5.50E-04	4.42E-03	antisense
A_23_P29851	LRPAP1	-0.98	9.87	5.51E-04	4.43E-03	protein_coding
A_33_P3394828	NOC4L	0.88	9.33	5.53E-04	4.45E-03	protein_coding
A_23_P94118	GTF2E2	1.09	8.45	5.55E-04	4.46E-03	protein_coding
A_23_P106922	CHST6	1.91	7.60	5.57E-04	4.48E-03	protein_coding

CUST_31319_PI428871386	ENST00000560203.1	-0.81	5.27	5.58E-04	4.48E-03	lincRNA
CUST_3479_PI428871386	ENST00000427540.1	0.95	5.94	5.58E-04	4.48E-03	antisense
A_24_P335092	SAA1	1.89	8.34	5.59E-04	4.49E-03	protein_coding
CUST_14913_PI428871386	ENST00000415787.1	-1.24	5.06	5.60E-04	4.50E-03	antisense
A_33_P3330503	ALDH7A1	-1.15	6.26	5.60E-04	4.50E-03	protein_coding
A_23_P358394	FAM65B	-1.08	6.52	5.61E-04	4.51E-03	protein_coding
A_23_P209799	MYO7B	-0.83	5.12	5.61E-04	4.51E-03	protein_coding
A_23_P94275	DKK4	1.00	5.27	5.61E-04	4.51E-03	protein_coding
A_33_P3409513	MAPK11	-1.17	8.78	5.62E-04	4.51E-03	protein_coding
CUST_38388_PI428871386	ENST00000578803.1	0.87	5.80	5.62E-04	4.51E-03	lincRNA
A_23_P87310	LMO1	1.36	5.63	5.62E-04	4.51E-03	protein_coding
CUST_37369_PI428871386	ENST00000582570.1	1.37	7.28	5.67E-04	4.55E-03	lincRNA
CUST_321_PI428871386	ENST00000442889.1	1.27	5.99	5.67E-04	4.55E-03	antisense
A_23_P13359	NXF1	-0.87	9.28	5.67E-04	4.55E-03	protein_coding
A_23_P77201	C15orf41	1.03	6.48	5.68E-04	4.56E-03	protein_coding
CUST_7236_PI428871386	ENST00000414135.1	0.80	5.16	5.69E-04	4.56E-03	antisense
CUST_15375_PI428871386	ENST00000452482.1	-0.91	5.68	5.70E-04	4.57E-03	antisense
CUST_14965_PI428871386	ENST00000504928.1	1.18	5.17	5.70E-04	4.57E-03	lincRNA
A_23_P131227	TTC27	1.05	9.48	5.71E-04	4.57E-03	protein_coding
CUST_39599_PI428871386	ENST00000586962.1	0.98	5.76	5.72E-04	4.58E-03	antisense
CUST_31302_PI428871386	ENST00000557883.2	-1.29	6.04	5.72E-04	4.58E-03	lincRNA
A_23_P252283	RNF135	-0.91	10.72	5.77E-04	4.62E-03	protein_coding
A_33_P3281408	YPEL5	-1.03	11.98	5.77E-04	4.62E-03	protein_coding
CUST_14568_PI428871386	ENST00000423504.1	-0.83	5.69	5.78E-04	4.63E-03	lincRNA
CUST_13583_PI428871386	ENST00000511256.1	1.24	5.34	5.78E-04	4.63E-03	lincRNA
CUST_20598_PI428871386	ENST00000444956.1	1.26	5.37	5.79E-04	4.63E-03	antisense
A_23_P310331	RANBP3	-0.87	10.51	5.79E-04	4.64E-03	protein_coding
CUST_30396_PI428871386	ENST00000554433.1	-0.81	4.74	5.80E-04	4.64E-03	antisense
A_33_P3361417	C6orf57	1.03	8.08	5.81E-04	4.65E-03	protein_coding
CUST_4983_PI428871386	ENST00000452840.1	-0.80	4.69	5.83E-04	4.66E-03	lincRNA
A_23_P320658	BUB3	0.98	10.30	5.83E-04	4.67E-03	protein_coding
A_33_P3338341	PRODH	-1.81	9.38	5.84E-04	4.67E-03	protein_coding
A_32_P209960	CIITA	-1.46	9.17	5.85E-04	4.68E-03	protein_coding
A_24_P72479	ARPC1A	0.94	11.04	5.85E-04	4.68E-03	protein_coding
A_24_P935782	ZNF121	0.98	6.97	5.86E-04	4.69E-03	protein_coding
CUST_10936_PI428871386	ENST00000505709.1	-1.00	6.49	5.86E-04	4.69E-03	lincRNA
A_23_P364766	C20orf94	0.96	8.40	5.88E-04	4.70E-03	protein_coding
CUST_43553_PI428871386	ENST00000468762.1	0.75	4.84	5.88E-04	4.70E-03	lincRNA
CUST_9471_PI428871386	ENST00000435560.1	-0.81	4.59	5.89E-04	4.71E-03	lincRNA
CUST_27136_PI428871386	ENST00000541715.1	1.26	5.78	5.89E-04	4.71E-03	antisense
CUST_39694_PI428871386	ENST00000588845.1	1.63	6.64	5.90E-04	4.72E-03	antisense
A_23_P149121	DIRAS3	-1.54	6.24	5.90E-04	4.72E-03	protein_coding
CUST_14822_PI428871386	ENST00000430595.1	-0.81	4.78	5.91E-04	4.72E-03	antisense
A_33_P3359157	FAM189B	1.02	6.08	5.91E-04	4.72E-03	protein_coding
A_23_P305723	MIER1	-0.83	8.93	5.93E-04	4.73E-03	protein_coding
A_23_P43898	EPHX4	1.45	5.90	5.93E-04	4.73E-03	protein_coding
CUST_8849_PI428871386	ENST00000468377.1	-1.49	8.06	5.96E-04	4.76E-03	antisense
CUST_32549_PI428871386	ENST00000555864.1	1.63	5.30	5.98E-04	4.77E-03	lincRNA
A_23_P368145	GORAB	0.98	7.17	5.99E-04	4.78E-03	protein_coding
CUST_8344_PI428871386	ENST00000480831.1	-0.91	5.32	6.00E-04	4.79E-03	antisense
CUST_28136_PI428871386	ENST00000546264.1	1.42	7.55	6.01E-04	4.79E-03	lincRNA
CUST_18832_PI428871386	ENST00000519032.1	-0.80	4.98	6.02E-04	4.80E-03	lincRNA
A_23_P90444	RBM42	0.97	10.42	6.02E-04	4.80E-03	protein_coding
CUST_27658_PI428871386	ENST00000539987.1	-0.83	5.33	6.02E-04	4.80E-03	antisense
A_23_P254688	TMEM108	-1.37	7.76	6.04E-04	4.81E-03	protein_coding
CUST_15582_PI428871386	ENST00000476099.1	-0.78	4.70	6.05E-04	4.82E-03	antisense
CUST_8796_PI428871386	ENST00000594843.1	-1.00	7.12	6.05E-04	4.82E-03	lincRNA
CUST_7212_PI428871386	ENST00000417922.1	-0.81	5.39	6.05E-04	4.82E-03	lincRNA
CUST_13316_PI428871386	ENST00000333482.4	-1.11	6.27	6.08E-04	4.84E-03	lincRNA
CUST_22020_PI428871386	ENST00000561822.1	-0.85	5.67	6.09E-04	4.85E-03	lincRNA
CUST_7140_PI428871386	ENST00000448588.1	1.08	7.08	6.10E-04	4.86E-03	antisense
A_23_P32029	SLC35D2	-0.94	7.33	6.12E-04	4.87E-03	protein_coding
CUST_31670_PI428871386	ENST00000559173.1	-0.92	5.27	6.13E-04	4.88E-03	antisense

CUST_37915_PI428871386	ENST00000591141.1	-1.04	5.32	6.14E-04	4.89E-03	lincRNA
CUST_7624_PI428871386	ENST00000414603.1	0.89	4.99	6.16E-04	4.90E-03	lincRNA
A_23_P452	DDR2	-1.07	7.39	6.17E-04	4.91E-03	protein_coding
A_33_P3825869	CACNA1C	-1.31	7.23	6.19E-04	4.93E-03	protein_coding
CUST_8168_PI428871386	ENST00000439898.1	-1.00	6.40	6.20E-04	4.93E-03	lincRNA
A_33_P3364379	YIF1A	1.10	9.16	6.21E-04	4.94E-03	protein_coding
A_32_P208350	TDRD9	-1.37	6.08	6.21E-04	4.94E-03	protein_coding
A_32_P111639	CHST9	-1.69	5.97	6.21E-04	4.94E-03	protein_coding
CUST_17709_PI428871386	ENST00000451786.1	-1.26	8.86	6.23E-04	4.96E-03	antisense
CUST_1031_PI428871386	ENST00000446786.1	0.88	4.74	6.23E-04	4.96E-03	antisense
A_32_P223017	MAP3K6	-1.06	9.06	6.25E-04	4.96E-03	protein_coding
A_32_P75792	FAM132A	1.40	6.21	6.25E-04	4.97E-03	protein_coding
A_33_P3324814	ENST00000414198	1.17	6.17	6.28E-04	4.99E-03	antisense
A_23_P363878	RFTN2	-0.91	5.62	6.31E-04	5.01E-03	protein_coding
A_23_P348636	FOXJ1	-1.81	7.75	6.31E-04	5.01E-03	protein_coding
A_23_P213000	WDR1	-1.02	9.18	6.31E-04	5.01E-03	protein_coding
CUST_19982_PI428871386	ENST00000517525.1	0.89	5.75	6.33E-04	5.02E-03	lincRNA
CUST_7810_PI428871386	ENST00000426200.1	-0.85	10.08	6.33E-04	5.02E-03	antisense
A_23_P36795	SYT1	1.84	6.45	6.35E-04	5.03E-03	protein_coding
CUST_43002_PI428871386	ENST00000422971.1	1.60	5.66	6.36E-04	5.04E-03	antisense
CUST_16437_PI428871386	ENST00000424101.1	-0.97	6.68	6.38E-04	5.06E-03	antisense
A_23_P13382	LSP1	-1.50	10.94	6.38E-04	5.06E-03	protein_coding
CUST_37371_PI428871386	ENST00000580336.1	1.00	4.88	6.39E-04	5.06E-03	lincRNA
CUST_322_PI428871386	ENST00000442889.1	1.32	6.13	6.40E-04	5.07E-03	antisense
CUST_39595_PI428871386	ENST00000587412.1	1.23	6.12	6.41E-04	5.07E-03	lincRNA
A_24_P926507	SLC14A1	-1.21	5.78	6.42E-04	5.08E-03	protein_coding
CUST_43711_PI428871386	ENST00000458472.1	0.79	4.89	6.43E-04	5.09E-03	lincRNA
CUST_8535_PI428871386	ENST00000502999.1	1.05	10.13	6.44E-04	5.10E-03	antisense
A_24_P363896	COL27A1	1.00	8.25	6.45E-04	5.10E-03	protein_coding
CUST_27333_PI428871386	ENST00000549527.1	-0.83	4.75	6.47E-04	5.12E-03	lincRNA
A_33_P3241937	TBC1D16	1.02	7.11	6.48E-04	5.13E-03	protein_coding
A_23_P338603	CHCHD7	1.30	8.54	6.48E-04	5.13E-03	protein_coding
CUST_21128_PI428871386	ENST00000423380.1	-1.15	6.48	6.50E-04	5.14E-03	lincRNA
CUST_37618_PI428871386	ENST00000592820.1	-0.79	4.54	6.50E-04	5.14E-03	antisense
A_33_P3283833	FOXS1	-1.31	8.36	6.52E-04	5.16E-03	protein_coding
A_23_P148541	CTAG1A	2.56	6.38	6.53E-04	5.16E-03	protein_coding
CUST_33504_PI428871386	ENST00000418886.1	-0.87	5.08	6.54E-04	5.17E-03	lincRNA
CUST_38514_PI428871386	ENST00000588380.1	0.75	5.01	6.55E-04	5.18E-03	antisense
CUST_15787_PI428871386	ENST00000447494.1	1.06	6.25	6.58E-04	5.20E-03	antisense
A_23_P368484	C17orf76	-1.50	8.11	6.60E-04	5.22E-03	protein_coding
CUST_22415_PI428871386	ENST00000439913.1	0.81	4.74	6.61E-04	5.22E-03	lincRNA
CUST_24033_PI428871386	ENST00000503469.2	-0.91	4.92	6.62E-04	5.23E-03	lincRNA
CUST_21787_PI428871386	ENST00000423793.1	1.05	5.77	6.62E-04	5.23E-03	antisense
A_23_P157736	PPAPDC3	-1.18	7.13	6.62E-04	5.23E-03	protein_coding
A_23_P127557	KBTBD3	-1.09	6.68	6.63E-04	5.24E-03	protein_coding
A_24_P414803	PLN	-1.12	5.79	6.67E-04	5.26E-03	protein_coding
CUST_36046_PI428871386	ENST00000301683.3	-0.75	4.74	6.67E-04	5.27E-03	lincRNA
CUST_8393_PI428871386	ENST00000598783.1	0.91	6.34	6.68E-04	5.27E-03	antisense
CUST_17208_PI428871386	ENST00000452471.1	-1.11	6.05	6.69E-04	5.28E-03	antisense
CUST_42083_PI428871386	ENST00000426578.1	-0.86	4.77	6.69E-04	5.28E-03	antisense
A_23_P42649	POLR2J	0.81	10.05	6.70E-04	5.29E-03	protein_coding
CUST_20051_PI428871386	ENST00000519695.1	-0.82	4.97	6.76E-04	5.33E-03	lincRNA
CUST_30627_PI428871386	ENST00000554260.1	-0.84	4.78	6.78E-04	5.34E-03	lincRNA
A_23_P84596	MZB1	1.80	10.16	6.78E-04	5.35E-03	protein_coding
A_33_P3417990	VRK2	0.96	7.68	6.78E-04	5.35E-03	protein_coding
CUST_39641_PI428871386	ENST00000448373.2	1.22	7.03	6.79E-04	5.35E-03	antisense
A_23_P128323	SCNN1A	-1.58	8.60	6.79E-04	5.35E-03	protein_coding
A_24_P127564	LINC00085	-1.04	7.04	6.80E-04	5.36E-03	lincRNA
CUST_6182_PI428871386	ENST00000595624.1	1.01	5.26	6.81E-04	5.36E-03	antisense
CUST_29521_PI428871386	ENST00000552826.1	1.28	5.06	6.81E-04	5.37E-03	antisense
A_33_P3314436	FBXL17	-0.90	7.23	6.82E-04	5.37E-03	protein_coding
A_23_P144911	EGFLAM	1.22	7.28	6.86E-04	5.40E-03	protein_coding
A_33_P3589543	ENST00000527620	-1.30	7.37	6.90E-04	5.43E-03	antisense

A_33_P3242388	PIGX	1.04	8.55	6.91E-04	5.44E-03	protein_coding
CUST_43451_PI428871386	ENST00000429124.1	-0.99	8.44	6.92E-04	5.45E-03	lincRNA
A_23_P218456	ILF3	0.94	10.39	6.92E-04	5.45E-03	protein_coding
A_23_P22499	GNL3L	1.07	8.80	6.92E-04	5.45E-03	protein_coding
A_23_P205046	ANKRD10	-1.12	8.12	6.93E-04	5.45E-03	protein_coding
CUST_37370_PI428871386	ENST00000580336.1	0.92	4.94	6.93E-04	5.45E-03	lincRNA
CUST_39697_PI428871386	ENST00000585890.1	1.43	6.15	6.94E-04	5.46E-03	antisense
CUST_27735_PI428871386	ENST00000531202.1	-0.86	5.49	6.95E-04	5.47E-03	antisense
CUST_36570_PI428871386	ENST00000585236.1	-1.10	7.36	6.96E-04	5.47E-03	antisense
A_33_P3339687	ZNF669	0.86	5.97	6.98E-04	5.49E-03	protein_coding
CUST_26726_PI428871386	ENST00000547387.1	0.86	7.22	7.00E-04	5.50E-03	antisense
CUST_23486_PI428871386	ENST00000435944.1	-0.85	4.92	7.01E-04	5.51E-03	antisense
CUST_22644_PI428871386	ENST00000430431.1	-1.17	5.42	7.02E-04	5.51E-03	lincRNA
CUST_9408_PI428871386	ENST00000424690.1	1.13	4.96	7.02E-04	5.51E-03	antisense
CUST_41873_PI428871386	ENST00000454980.1	1.00	5.37	7.04E-04	5.52E-03	antisense
CUST_15181_PI428871386	ENST00000588761.1	0.78	4.95	7.06E-04	5.54E-03	antisense
CUST_37076_PI428871386	ENST00000586321.1	-0.77	4.90	7.08E-04	5.56E-03	lincRNA
CUST_4010_PI428871386	ENST00000416463.1	-1.06	7.88	7.10E-04	5.57E-03	lincRNA
CUST_9716_PI428871386	ENST00000431512.1	-1.60	7.13	7.11E-04	5.58E-03	antisense
A_23_P30495	HMGCR	-0.98	9.87	7.12E-04	5.59E-03	protein_coding
CUST_7100_PI428871386	ENST00000441223.1	0.80	4.82	7.14E-04	5.60E-03	lincRNA
A_23_P7727	HAPLN1	1.40	5.43	7.14E-04	5.60E-03	protein_coding
A_32_P175539	RCN2	1.08	11.47	7.14E-04	5.60E-03	protein_coding
CUST_17687_PI428871386	ENST00000469264.1	-0.82	5.15	7.16E-04	5.61E-03	antisense
A_23_P405873	C9orf72	-0.97	6.82	7.16E-04	5.62E-03	protein_coding
CUST_16826_PI428871386	ENST00000569710.1	-0.86	6.94	7.17E-04	5.62E-03	antisense
A_23_P48121	ADIPOR2	-1.03	10.06	7.19E-04	5.64E-03	protein_coding
A_23_P27180	NSRP1	-0.85	9.04	7.20E-04	5.65E-03	protein_coding
CUST_13388_PI428871386	ENST00000510972.1	-1.17	5.87	7.21E-04	5.65E-03	antisense
A_23_P25935	C14orf1	-1.06	8.82	7.24E-04	5.67E-03	protein_coding
A_24_P212443	UBE3C	0.89	6.01	7.27E-04	5.69E-03	protein_coding
CUST_30545_PI428871386	ENST00000555032.1	-0.81	4.86	7.28E-04	5.70E-03	lincRNA
A_23_P79661	CCDC93	-0.86	8.17	7.28E-04	5.70E-03	protein_coding
CUST_15430_PI428871386	ENST00000444910.1	-0.77	5.27	7.29E-04	5.71E-03	lincRNA
CUST_11024_PI428871386	ENST00000512129.1	0.84	5.19	7.32E-04	5.73E-03	antisense
CUST_39640_PI428871386	ENST00000448373.2	1.23	7.15	7.33E-04	5.74E-03	antisense
CUST_825_PI428871386	ENST00000419993.1	-0.85	5.76	7.33E-04	5.74E-03	antisense
A_23_P27075	GABARAP	-0.95	12.23	7.33E-04	5.74E-03	protein_coding
A_24_P40978	CYHR1	0.98	7.13	7.34E-04	5.74E-03	protein_coding
A_23_P70566	FKBPL	1.01	5.64	7.35E-04	5.75E-03	protein_coding
CUST_14819_PI428871386	ENST00000416948.1	-0.75	4.66	7.35E-04	5.75E-03	antisense
A_24_P237270	ADORA2A	-1.03	5.96	7.36E-04	5.76E-03	protein_coding
CUST_21584_PI428871386	ENST00000591408.1	0.84	5.58	7.37E-04	5.76E-03	antisense
CUST_35953_PI428871386	ENST00000583195.1	-0.88	5.36	7.38E-04	5.77E-03	lincRNA
A_23_P38618	PIGL	0.92	6.87	7.39E-04	5.77E-03	protein_coding
CUST_43450_PI428871386	ENST00000430772.1	-0.78	6.40	7.40E-04	5.78E-03	lincRNA
A_33_P3412767	SYDE2	-0.97	6.10	7.41E-04	5.79E-03	protein_coding
CUST_4554_PI428871386	ENST00000352271.6	0.85	5.31	7.44E-04	5.82E-03	antisense
CUST_849_PI428871386	ENST00000450157.1	-1.08	10.31	7.45E-04	5.82E-03	antisense
A_33_P3277988	IFNAR1	-0.95	9.19	7.45E-04	5.82E-03	protein_coding
CUST_43400_PI428871386	ENST00000424211.1	0.94	5.32	7.49E-04	5.85E-03	antisense
CUST_36571_PI428871386	ENST00000585236.1	-1.10	7.33	7.49E-04	5.85E-03	antisense
CUST_20582_PI428871386	ENST00000442432.1	-0.87	5.26	7.50E-04	5.85E-03	antisense
CUST_29965_PI428871386	ENST00000556400.1	-0.86	5.84	7.50E-04	5.86E-03	antisense
A_33_P3784283	JAK1	-0.91	7.91	7.52E-04	5.87E-03	protein_coding
CUST_8276_PI428871386	ENST00000464125.1	1.21	6.16	7.56E-04	5.90E-03	antisense
A_23_P215154	NUB1	-1.00	9.12	7.60E-04	5.93E-03	protein_coding
A_32_P6832	C5orf51	0.90	9.33	7.61E-04	5.93E-03	protein_coding
A_33_P3420792	PDAP1	0.96	6.49	7.62E-04	5.94E-03	protein_coding
CUST_4081_PI428871386	ENST00000431182.1	-0.83	4.63	7.64E-04	5.96E-03	lincRNA
CUST_17848_PI428871386	ENST00000429289.1	-1.04	6.29	7.65E-04	5.96E-03	antisense
CUST_27673_PI428871386	ENST00000553177.1	-0.75	5.17	7.68E-04	5.98E-03	lincRNA
A_23_P321388	RNF19B	1.27	8.17	7.68E-04	5.98E-03	protein_coding

A_33_P3238573	DENND4B	-0.86	11.06	7.69E-04	5.99E-03	protein_coding
CUST_26137_PI428871386	ENST00000539196.1	1.01	11.91	7.69E-04	5.99E-03	protein_coding
CUST_31305_PI428871386	ENST00000558897.1	-1.38	6.63	7.70E-04	5.99E-03	lincRNA
CUST_33256_PI428871386	ENST00000569407.1	-0.79	4.84	7.71E-04	6.00E-03	antisense
CUST_26888_PI428871386	ENST00000513533.1	0.80	8.16	7.72E-04	6.01E-03	antisense
A_33_P3406796	ENST00000511256	1.27	5.30	7.72E-04	6.01E-03	lincRNA
A_33_P3239084	FAM86A	0.82	7.51	7.73E-04	6.02E-03	protein_coding
A_33_P3276068	BET3L	-0.89	10.69	7.75E-04	6.03E-03	protein_coding
CUST_7808_PI428871386	ENST00000417835.1	-0.84	10.09	7.75E-04	6.03E-03	antisense
A_23_P214026	FBN2	1.89	5.69	7.75E-04	6.03E-03	protein_coding
A_33_P3417313	PYCRL	0.98	5.30	7.79E-04	6.06E-03	protein_coding
CUST_539_PI428871386	ENST00000421114.1	-0.76	5.37	7.79E-04	6.06E-03	lincRNA
CUST_5289_PI428871386	ENST00000602091.1	-0.81	5.22	7.81E-04	6.08E-03	antisense
CUST_13201_PI428871386	ENST00000514769.1	0.93	4.85	7.81E-04	6.08E-03	lincRNA
CUST_38853_PI428871386	ENST00000591838.1	-0.87	6.69	7.82E-04	6.08E-03	antisense
A_33_P3310293	PKIG	-1.15	9.50	7.83E-04	6.09E-03	protein_coding
A_23_P302018	TXK	-0.85	5.60	7.83E-04	6.09E-03	protein_coding
A_24_P376129	DFNB31	0.95	5.54	7.85E-04	6.10E-03	protein_coding
CUST_8817_PI428871386	ENST00000512384.1	-0.84	4.98	7.85E-04	6.10E-03	antisense
A_23_P160177	ATP1A4	-0.96	8.84	7.87E-04	6.11E-03	protein_coding
CUST_8345_PI428871386	ENST00000480831.1	-0.86	5.20	7.88E-04	6.13E-03	antisense
CUST_7939_PI428871386	ENST00000356047.3	1.04	5.35	7.88E-04	6.13E-03	lincRNA
CUST_20201_PI428871386	ENST00000517411.1	1.18	6.33	7.89E-04	6.13E-03	antisense
A_33_P3383236	C2orf62	-0.94	5.48	7.89E-04	6.13E-03	protein_coding
A_23_P158053	C9orf16	0.89	8.52	7.90E-04	6.14E-03	protein_coding
CUST_14447_PI428871386	ENST00000429060.1	-1.23	6.39	7.91E-04	6.14E-03	lincRNA
A_33_P3395191	KCNMB2	-1.32	5.43	7.92E-04	6.15E-03	protein_coding
A_33_P3361701	UBAP2L	0.98	8.48	7.95E-04	6.17E-03	protein_coding
CUST_21579_PI428871386	ENST00000590283.1	0.77	5.57	7.96E-04	6.18E-03	antisense
CUST_9791_PI428871386	ENST00000453671.1	1.21	8.05	7.97E-04	6.18E-03	antisense
A_33_P3286387	EHMT1	0.81	6.26	7.99E-04	6.20E-03	protein_coding
A_23_P347432	DVL1	0.96	7.41	8.00E-04	6.20E-03	protein_coding
CUST_25853_PI428871386	ENST00000530583.1	0.81	5.14	8.01E-04	6.21E-03	lincRNA
A_33_P3282005	VANGL2	1.42	6.13	8.01E-04	6.21E-03	protein_coding
CUST_24806_PI428871386	ENST00000534178.1	0.79	4.81	8.03E-04	6.23E-03	antisense
CUST_16441_PI428871386	ENST00000452249.1	-0.97	6.56	8.04E-04	6.23E-03	antisense
A_24_P944756	NECAB1	-0.99	5.35	8.04E-04	6.23E-03	protein_coding
CUST_15788_PI428871386	ENST00000447494.1	1.06	6.14	8.04E-04	6.23E-03	antisense
A_33_P3289996	USP45	0.82	6.46	8.07E-04	6.26E-03	protein_coding
A_23_P129569	PALB2	0.87	6.02	8.09E-04	6.27E-03	protein_coding
A_33_P3262012	CHPF	1.13	8.38	8.13E-04	6.30E-03	protein_coding
A_23_P142125	HRC	-1.08	6.06	8.14E-04	6.31E-03	protein_coding
CUST_21572_PI428871386	ENST00000592240.1	0.81	5.58	8.15E-04	6.31E-03	antisense
CUST_29438_PI428871386	ENST00000553792.1	-0.85	4.89	8.16E-04	6.32E-03	antisense
CUST_24961_PI428871386	ENST00000533170.1	0.86	5.08	8.19E-04	6.34E-03	lincRNA
CUST_10937_PI428871386	ENST00000505709.1	-0.99	6.45	8.20E-04	6.35E-03	lincRNA
CUST_10169_PI428871386	ENST00000503163.1	-0.78	4.63	8.21E-04	6.35E-03	lincRNA
CUST_237_PI428871386	ENST00000448624.2	-0.80	5.89	8.21E-04	6.36E-03	antisense
A_33_P3278407	MLLT4	-1.03	10.80	8.23E-04	6.37E-03	protein_coding
A_33_P3369844	CD24	1.85	12.07	8.24E-04	6.38E-03	protein_coding
A_23_P92614	HELQ	-0.82	6.85	8.24E-04	6.38E-03	protein_coding
A_24_P170753	ENST00000443539	0.92	5.93	8.26E-04	6.39E-03	protein_coding
CUST_10635_PI428871386	ENST00000508572.1	1.55	5.22	8.29E-04	6.41E-03	lincRNA
CUST_17712_PI428871386	ENST00000443623.1	-1.28	9.03	8.29E-04	6.41E-03	antisense
A_33_P3287745	KAT8	-0.88	10.66	8.30E-04	6.42E-03	protein_coding
A_23_P216376	CNGB3	0.88	5.05	8.31E-04	6.42E-03	protein_coding
A_23_P45831	CHD1L	1.03	8.75	8.33E-04	6.44E-03	protein_coding
CUST_19711_PI428871386	ENST00000509144.2	1.56	5.20	8.36E-04	6.46E-03	antisense
CUST_29804_PI428871386	ENST00000557160.1	-0.82	7.92	8.38E-04	6.47E-03	antisense
CUST_15386_PI428871386	ENST00000369123.3	0.77	6.57	8.39E-04	6.48E-03	lincRNA
CUST_37354_PI428871386	ENST00000578835.1	0.85	5.81	8.44E-04	6.52E-03	lincRNA
CUST_28693_PI428871386	ENST00000433070.1	1.01	6.08	8.44E-04	6.52E-03	antisense
CUST_21949_PI428871386	ENST00000418372.1	-0.87	5.40	8.45E-04	6.52E-03	lincRNA

CUST_37557_PI428871386	ENST00000579467.1	-0.83	5.20	8.51E-04	6.57E-03	antisense
A_23_P131626	SNRNP200	0.89	11.21	8.51E-04	6.57E-03	protein_coding
A_23_P31143	TPD52L1	1.65	10.30	8.52E-04	6.57E-03	protein_coding
A_23_P81529	ISL1	1.84	5.16	8.53E-04	6.58E-03	protein_coding
A_33_P3271105	PABPC4	0.87	5.47	8.56E-04	6.60E-03	protein_coding
A_33_P3285156	FLJ90757	-1.21	9.63	8.57E-04	6.61E-03	lincRNA
A_23_P253029	BOK	-1.05	8.95	8.61E-04	6.64E-03	protein_coding
CUST_37550_PI428871386	ENST00000579368.1	1.11	5.02	8.63E-04	6.65E-03	antisense
CUST_3113_PI428871386	ENST00000566394.1	-1.12	5.93	8.64E-04	6.67E-03	antisense
A_23_P122545	RDBP	0.93	11.10	8.66E-04	6.68E-03	protein_coding
A_23_P257911	USP16	-0.78	10.31	8.68E-04	6.69E-03	protein_coding
CUST_33703_PI428871386	ENST00000565549.1	-0.84	5.56	8.69E-04	6.70E-03	lincRNA
CUST_19920_PI428871386	ENST00000522865.1	-0.79	5.16	8.70E-04	6.71E-03	lincRNA
A_23_P251562	TUSC2	-0.85	10.93	8.71E-04	6.71E-03	protein_coding
CUST_33337_PI428871386	ENST00000569048.1	1.00	5.83	8.72E-04	6.72E-03	lincRNA
A_23_P41365	SMR3A	1.36	7.35	8.72E-04	6.72E-03	protein_coding
CUST_40302_PI428871386	ENST00000589456.1	-0.91	5.61	8.74E-04	6.73E-03	antisense
A_24_P304723	PPIB	0.93	11.67	8.75E-04	6.74E-03	protein_coding
A_23_P164196	DLX4	1.29	6.53	8.77E-04	6.75E-03	protein_coding
CUST_9662_PI428871386	ENST00000440726.1	1.39	5.35	8.78E-04	6.76E-03	lincRNA
A_33_P3354429	TRIM71	-1.32	5.63	8.79E-04	6.77E-03	protein_coding
A_23_P71855	C5	-1.59	7.36	8.80E-04	6.77E-03	protein_coding
A_23_P20606	NIPSNAP3A	-1.04	8.93	8.84E-04	6.80E-03	protein_coding
A_23_P368154	PODN	-1.11	6.17	8.86E-04	6.82E-03	protein_coding
CUST_19716_PI428871386	ENST00000521622.1	1.06	5.05	8.87E-04	6.83E-03	antisense
A_33_P3224803	NCF1	-1.26	6.83	8.88E-04	6.83E-03	protein_coding
A_33_P3238323	RBCK1	-1.09	7.86	8.92E-04	6.86E-03	protein_coding
A_33_P3230658	TSNAX	0.88	10.08	8.94E-04	6.87E-03	protein_coding
CUST_5496_PI428871386	ENST00000425953.1	1.02	5.67	8.94E-04	6.87E-03	protein_coding
A_33_P3262495	ZNF503	-1.25	10.68	8.96E-04	6.89E-03	protein_coding
A_23_P99172	CCDC77	1.11	8.58	8.97E-04	6.89E-03	protein_coding
CUST_43272_PI428871386	ENST00000438867.1	0.76	5.09	8.98E-04	6.90E-03	lincRNA
CUST_17714_PI428871386	ENST00000435523.1	-0.91	5.96	8.99E-04	6.90E-03	antisense
CUST_23399_PI428871386	ENST00000601505.1	-0.81	4.96	9.03E-04	6.94E-03	lincRNA
CUST_5558_PI428871386	ENST00000597941.1	-0.81	5.30	9.04E-04	6.94E-03	antisense
CUST_41000_PI428871386	ENST00000456953.1	0.82	7.65	9.06E-04	6.96E-03	lincRNA
CUST_18072_PI428871386	ENST00000435354.1	1.15	6.28	9.07E-04	6.97E-03	lincRNA
A_23_P405885	DPPA2	0.99	4.99	9.08E-04	6.97E-03	protein_coding
A_23_P65797	KLHL25	1.04	9.09	9.12E-04	7.00E-03	protein_coding
CUST_887_PI428871386	ENST00000429109.1	-0.99	6.11	9.12E-04	7.00E-03	antisense
A_33_P3270451	TXNDC5	1.29	10.02	9.13E-04	7.00E-03	protein_coding
A_23_P370454	KCNAB3	0.78	5.47	9.17E-04	7.04E-03	protein_coding
A_23_P319133	DNAJC10	0.96	10.01	9.18E-04	7.04E-03	protein_coding
CUST_36293_PI428871386	ENST00000584391.1	0.84	6.17	9.19E-04	7.05E-03	antisense
CUST_26166_PI428871386	ENST00000454799.2	-1.00	6.17	9.19E-04	7.05E-03	antisense
CUST_18831_PI428871386	ENST00000519032.1	-0.82	4.78	9.19E-04	7.05E-03	lincRNA
CUST_280_PI428871386	ENST00000423764.1	-1.18	9.60	9.20E-04	7.06E-03	antisense
A_33_P3418414	GPX6	-0.85	4.93	9.23E-04	7.07E-03	protein_coding
A_33_P3274851	COX5B	0.99	12.64	9.24E-04	7.08E-03	protein_coding
A_24_P262355	PHB	0.98	10.08	9.25E-04	7.08E-03	protein_coding
A_23_P130531	CDC37	-0.94	10.04	9.30E-04	7.13E-03	protein_coding
A_33_P3317618	SYN2	-1.66	6.02	9.31E-04	7.13E-03	protein_coding
CUST_10454_PI428871386	ENST00000507639.1	1.42	5.49	9.32E-04	7.14E-03	lincRNA
CUST_37036_PI428871386	ENST00000582422.1	-0.91	5.71	9.33E-04	7.15E-03	lincRNA
A_23_P44836	NT5DC2	1.27	8.96	9.34E-04	7.15E-03	protein_coding
CUST_20402_PI428871386	ENST00000440674.1	0.73	5.07	9.34E-04	7.15E-03	antisense
CUST_21220_PI428871386	ENST00000583864.1	0.86	7.93	9.36E-04	7.17E-03	lincRNA
A_33_P3323842	BDNF-AS1	-0.94	6.29	9.37E-04	7.17E-03	antisense
A_23_P250644	CDKN2AIPNL	0.93	7.42	9.37E-04	7.17E-03	protein_coding
CUST_16448_PI428871386	ENST00000454003.1	-1.27	5.79	9.41E-04	7.20E-03	lincRNA
CUST_26717_PI428871386	ENST00000547395.1	0.95	5.58	9.42E-04	7.21E-03	antisense
CUST_14113_PI428871386	ENST00000523279.1	0.74	5.05	9.43E-04	7.22E-03	antisense
A_33_P3629930	ZNF32-AS3	-0.86	5.01	9.46E-04	7.23E-03	antisense

CUST_7203_PI428871386	ENST00000423530.1	-0.92	5.56	9.46E-04	7.24E-03	lincRNA
A_23_P166677	MFSD1	-1.11	11.90	9.47E-04	7.24E-03	protein_coding
CUST_9210_PI428871386	ENST00000462531.1	0.76	4.97	9.48E-04	7.25E-03	lincRNA
A_23_P405878	C12orf54	1.39	6.13	9.55E-04	7.30E-03	protein_coding
A_23_P259071	AREG	-2.71	10.38	9.63E-04	7.36E-03	protein_coding
CUST_12890_PI428871386	ENST00000513899.1	0.80	5.05	9.68E-04	7.39E-03	antisense
CUST_36701_PI428871386	ENST00000582965.1	1.23	6.61	9.71E-04	7.42E-03	lincRNA
A_24_P943017	CERS3	1.35	4.96	9.72E-04	7.42E-03	protein_coding
A_24_P379104	PIM2	1.44	9.72	9.72E-04	7.42E-03	protein_coding
A_23_P14493	DNAAF2	1.00	8.27	9.72E-04	7.42E-03	protein_coding
A_33_P3272189	MFSD9	0.96	8.41	9.75E-04	7.44E-03	protein_coding
CUST_26298_PI428871386	ENST00000543515.1	1.26	6.54	9.80E-04	7.48E-03	antisense
A_23_P43276	GPR124	-1.22	8.77	9.81E-04	7.49E-03	protein_coding
A_33_P3365878	BMP8B	-1.02	12.91	9.81E-04	7.49E-03	protein_coding
CUST_3076_PI428871386	ENST00000417409.1	-0.79	4.81	9.81E-04	7.49E-03	lincRNA
CUST_3923_PI428871386	ENST00000589936.1	0.78	5.00	9.82E-04	7.49E-03	lincRNA
A_33_P3392092	ADCYAP1	-1.39	5.39	9.83E-04	7.50E-03	protein_coding
A_24_P912439	AKNA	-0.89	5.87	9.83E-04	7.50E-03	protein_coding
A_24_P167877	LOC100132247	-1.15	14.40	9.85E-04	7.51E-03	protein_coding
CUST_31930_PI428871386	ENST00000559298.1	-0.91	7.29	9.86E-04	7.52E-03	lincRNA
CUST_21119_PI428871386	ENST00000449990.1	-0.76	5.36	9.89E-04	7.54E-03	lincRNA
CUST_2891_PI428871386	ENST00000425771.1	1.26	6.76	9.90E-04	7.55E-03	lincRNA
CUST_43962_PI428871386	ENST00000454875.1	-1.15	5.71	9.91E-04	7.55E-03	lincRNA
A_33_P3232504	C9orf169	1.47	6.49	9.93E-04	7.57E-03	protein_coding
CUST_40358_PI428871386	ENST00000592146.1	-1.31	7.26	9.96E-04	7.59E-03	lincRNA
CUST_26208_PI428871386	ENST00000539795.1	-0.94	6.48	9.98E-04	7.60E-03	lincRNA
A_23_P36689	LRRC23	-0.95	7.46	9.99E-04	7.61E-03	protein_coding
A_23_P92012	TSEN2	-0.90	8.14	1.00E-03	7.63E-03	protein_coding
A_33_P3270767	ENST00000471222	1.26	4.98	1.00E-03	7.64E-03	lincRNA
CUST_43252_PI428871386	ENST00000412652.1	1.22	5.10	1.00E-03	7.64E-03	lincRNA
A_23_P13740	NAV3	-1.07	5.97	1.00E-03	7.65E-03	protein_coding
CUST_17335_PI428871386	ENST00000468960.2	-0.94	6.18	1.01E-03	7.67E-03	antisense
A_33_P3393341	LPIN3	1.14	10.18	1.01E-03	7.67E-03	protein_coding
CUST_31669_PI428871386	ENST00000560727.1	-0.93	5.62	1.01E-03	7.68E-03	antisense
A_24_P415327	HPS1	-0.96	8.13	1.01E-03	7.68E-03	protein_coding
A_33_P3416882	ARL9	1.52	7.16	1.01E-03	7.70E-03	protein_coding
A_23_P72077	IL12RB2	1.10	5.49	1.01E-03	7.71E-03	protein_coding
A_33_P3278265	PYROXD1	-0.97	8.44	1.02E-03	7.73E-03	protein_coding
CUST_29092_PI428871386	ENST00000451662.1	0.76	5.40	1.02E-03	7.74E-03	antisense
CUST_30579_PI428871386	ENST00000553445.1	1.39	7.46	1.02E-03	7.74E-03	antisense
A_33_P3258593	PRB1	1.23	7.87	1.02E-03	7.75E-03	protein_coding
CUST_524_PI428871386	ENST00000417884.1	1.21	5.22	1.02E-03	7.77E-03	lincRNA
CUST_207_PI428871386	ENST00000412228.1	-0.79	5.03	1.03E-03	7.79E-03	antisense
A_23_P139500	BHLHE41	-1.64	9.43	1.03E-03	7.80E-03	protein_coding
A_33_P3290040	CPT1C	1.38	7.36	1.03E-03	7.80E-03	protein_coding
CUST_26299_PI428871386	ENST00000543515.1	1.29	6.94	1.03E-03	7.81E-03	antisense
CUST_9942_PI428871386	ENST00000452051.1	-0.76	4.81	1.03E-03	7.81E-03	antisense
CUST_32237_PI428871386	ENST00000562716.1	-0.84	5.80	1.03E-03	7.82E-03	antisense
CUST_24811_PI428871386	ENST00000532454.1	0.76	4.93	1.03E-03	7.83E-03	antisense
CUST_15001_PI428871386	ENST00000425089.1	0.95	4.93	1.03E-03	7.84E-03	antisense
A_23_P200670	WDR78	-1.35	6.63	1.03E-03	7.84E-03	protein_coding
A_23_P347169	MTUS1	-1.00	7.86	1.03E-03	7.85E-03	protein_coding
CUST_20935_PI428871386	ENST00000451596.1	-0.92	6.10	1.04E-03	7.86E-03	lincRNA
A_23_P130304	TXNL4A	1.02	12.06	1.04E-03	7.91E-03	protein_coding
A_32_P9543	APOBEC3A	-1.70	7.07	1.04E-03	7.92E-03	protein_coding
A_24_P349965	TCF19	1.01	6.18	1.05E-03	7.93E-03	protein_coding
CUST_36963_PI428871386	ENST00000582668.1	0.79	5.31	1.05E-03	7.93E-03	antisense
CUST_8100_PI428871386	ENST00000429315.2	1.12	7.56	1.05E-03	7.93E-03	antisense
A_33_P3413083	RHEB	-0.87	8.40	1.05E-03	7.94E-03	protein_coding
CUST_43643_PI428871386	ENST00000433425.2	-1.49	8.83	1.05E-03	7.94E-03	lincRNA
CUST_3607_PI428871386	ENST00000425412.1	0.98	6.02	1.05E-03	7.94E-03	antisense
CUST_40400_PI428871386	ENST00000597973.1	1.01	5.42	1.05E-03	7.95E-03	antisense
CUST_2727_PI428871386	ENST00000415000.1	-0.72	5.24	1.05E-03	7.97E-03	antisense

CUST_24060_PI428871386	ENST00000527945.1	1.09	5.91	1.05E-03	7.98E-03	lincRNA
A_23_P91697	LARGE	-1.47	8.58	1.05E-03	7.98E-03	protein_coding
A_24_P944588	ZNF682	-1.15	6.37	1.05E-03	7.98E-03	protein_coding
A_23_P22352	FRMD4A	-1.28	8.41	1.05E-03	7.98E-03	protein_coding
CUST_6408_PI428871386	ENST00000428651.1	1.45	5.82	1.06E-03	8.00E-03	lincRNA
A_23_P371129	SLX4	0.87	7.42	1.06E-03	8.00E-03	protein_coding
A_33_P3255531	LOC100129216	-0.83	4.98	1.06E-03	8.02E-03	protein_coding
A_23_P325690	ANKRD35	-1.27	9.48	1.06E-03	8.02E-03	protein_coding
A_33_P3337771	TGS1	0.98	9.21	1.06E-03	8.03E-03	protein_coding
CUST_34700_PI428871386	ENST00000569147.1	-0.79	4.92	1.06E-03	8.04E-03	antisense
CUST_41778_PI428871386	ENST00000440052.1	-0.97	6.22	1.06E-03	8.05E-03	antisense
CUST_9242_PI428871386	ENST00000467995.1	-0.81	4.98	1.07E-03	8.06E-03	lincRNA
CUST_36700_PI428871386	ENST00000582965.1	1.23	6.74	1.07E-03	8.06E-03	lincRNA
CUST_29262_PI428871386	ENST00000548416.1	1.56	5.05	1.07E-03	8.06E-03	lincRNA
A_23_P415652	GALNT12	-1.16	7.00	1.07E-03	8.06E-03	protein_coding
CUST_34778_PI428871386	ENST00000565053.1	1.28	5.32	1.07E-03	8.07E-03	antisense
A_23_P120254	DUSP22	-0.88	9.29	1.07E-03	8.09E-03	protein_coding
CUST_853_PI428871386	ENST00000417869.1	-1.04	9.97	1.07E-03	8.10E-03	antisense
A_23_P62881	SGIP1	-0.94	5.71	1.07E-03	8.11E-03	protein_coding
A_33_P3329013	SSTR2	1.35	6.92	1.08E-03	8.13E-03	protein_coding
A_33_P3255794	POLE	0.83	5.30	1.08E-03	8.13E-03	protein_coding
A_24_P413920	FAM84A	1.06	6.62	1.08E-03	8.14E-03	protein_coding
CUST_3220_PI428871386	ENST00000447832.1	-1.20	6.03	1.08E-03	8.14E-03	antisense
A_24_P302584	SOX11	1.57	5.75	1.08E-03	8.15E-03	protein_coding
CUST_34355_PI428871386	ENST00000568137.1	-0.81	4.79	1.08E-03	8.16E-03	lincRNA
A_23_P409462	DCBLD1	0.97	7.37	1.09E-03	8.19E-03	protein_coding
CUST_2634_PI428871386	ENST00000423943.1	1.49	6.28	1.09E-03	8.20E-03	lincRNA
CUST_20202_PI428871386	ENST00000517411.1	1.20	6.42	1.09E-03	8.21E-03	antisense
CUST_29559_PI428871386	ENST00000549013.1	0.77	4.98	1.09E-03	8.21E-03	lincRNA
A_32_P44453	INPP1	-1.09	7.23	1.09E-03	8.22E-03	protein_coding
CUST_35759_PI428871386	ENST00000584721.1	-0.98	6.42	1.10E-03	8.27E-03	lincRNA
CUST_34910_PI428871386	ENST00000573127.1	-0.94	5.90	1.10E-03	8.28E-03	lincRNA
A_33_P3292919	TCOF1	0.84	9.57	1.10E-03	8.28E-03	protein_coding
A_24_P98555	FAM45A	0.88	7.47	1.10E-03	8.29E-03	protein_coding
CUST_34734_PI428871386	ENST00000562705.1	0.95	5.24	1.10E-03	8.31E-03	lincRNA
CUST_29894_PI428871386	ENST00000560267.1	1.13	5.45	1.10E-03	8.31E-03	lincRNA
A_23_P139143	STX3	-1.07	7.48	1.10E-03	8.31E-03	protein_coding
A_23_P337934	FBLIM1	1.10	8.46	1.11E-03	8.34E-03	protein_coding
A_23_P336554	IL1RAP	1.37	7.20	1.11E-03	8.35E-03	protein_coding
A_23_P136232	IMPAD1	1.01	8.13	1.11E-03	8.36E-03	protein_coding
A_33_P3278318	MSRB2	-0.92	6.93	1.11E-03	8.37E-03	protein_coding
CUST_4401_PI428871386	ENST00000419083.1	0.81	6.42	1.11E-03	8.37E-03	antisense
CUST_14190_PI428871386	ENST00000511565.1	-0.92	5.84	1.11E-03	8.37E-03	antisense
CUST_39698_PI428871386	ENST00000589025.1	1.37	6.02	1.11E-03	8.38E-03	antisense
A_33_P3280385	COL6A3	1.12	6.29	1.12E-03	8.39E-03	protein_coding
CUST_29042_PI428871386	ENST00000438290.1	0.93	6.36	1.12E-03	8.40E-03	lincRNA
CUST_30370_PI428871386	ENST00000553668.1	-0.87	4.97	1.12E-03	8.41E-03	lincRNA
A_23_P152791	SLC16A6	-1.23	6.85	1.12E-03	8.42E-03	protein_coding
CUST_19515_PI428871386	ENST00000521203.1	-0.80	5.12	1.12E-03	8.42E-03	antisense
CUST_13195_PI428871386	ENST00000507050.1	1.17	6.31	1.12E-03	8.43E-03	antisense
A_33_P3278856	POF1B	1.15	5.14	1.12E-03	8.45E-03	protein_coding
A_24_P10657	SLC44A2	-1.04	9.61	1.13E-03	8.47E-03	protein_coding
A_33_P3283971	NFKBIL1	0.91	7.99	1.13E-03	8.49E-03	protein_coding
CUST_17373_PI428871386	ENST00000448513.1	-0.83	4.88	1.13E-03	8.49E-03	antisense
A_33_P3264846	SAMD9L	-1.35	8.28	1.13E-03	8.51E-03	protein_coding
CUST_5914_PI428871386	ENST00000437551.1	-1.45	9.20	1.13E-03	8.52E-03	antisense
A_23_P368896	SNX12	0.86	6.27	1.14E-03	8.53E-03	protein_coding
A_33_P3240258	RPN2	0.85	12.67	1.14E-03	8.54E-03	protein_coding
CUST_3056_PI428871386	ENST00000569873.1	0.78	4.95	1.14E-03	8.54E-03	lincRNA
CUST_37428_PI428871386	ENST00000573177.1	1.14	5.57	1.15E-03	8.60E-03	antisense
A_23_P146217	BAG4	1.11	6.57	1.15E-03	8.60E-03	protein_coding
A_33_P3356320	C17orf97	-1.13	6.80	1.15E-03	8.60E-03	protein_coding
CUST_19916_PI428871386	ENST00000521991.1	-0.81	4.70	1.15E-03	8.61E-03	lincRNA

A_23_P302005	STON1	-1.20	8.41	1.15E-03	8.61E-03	protein_coding
CUST_15691_PI428871386	ENST00000417483.1	1.31	5.43	1.15E-03	8.62E-03	lincRNA
A_23_P151907	PCSK6	1.00	6.20	1.15E-03	8.62E-03	protein_coding
CUST_6407_PI428871386	ENST00000448255.1	1.34	5.36	1.15E-03	8.63E-03	lincRNA
A_23_P81898	UBD	2.32	11.43	1.15E-03	8.65E-03	protein_coding
A_23_P215744	CTTNBP2	-1.20	6.68	1.16E-03	8.66E-03	protein_coding
A_23_P50418	ZNF791	-0.80	8.04	1.16E-03	8.69E-03	protein_coding
A_32_P151933	FECH	-0.90	5.71	1.16E-03	8.69E-03	protein_coding
CUST_26448_PI428871386	ENST00000500276.2	-1.03	7.36	1.16E-03	8.70E-03	antisense
CUST_28137_PI428871386	ENST00000546264.1	1.34	7.44	1.16E-03	8.71E-03	lincRNA
A_24_P13381	TRPV4	1.01	6.20	1.16E-03	8.71E-03	protein_coding
A_23_P387057	TUBB	1.06	10.09	1.16E-03	8.71E-03	protein_coding
A_23_P145376	MAPK13	1.07	8.34	1.17E-03	8.73E-03	protein_coding
CUST_7811_PI428871386	ENST00000426200.1	-0.81	10.01	1.17E-03	8.74E-03	antisense
CUST_17690_PI428871386	ENST00000470348.1	-0.94	6.13	1.17E-03	8.74E-03	antisense
A_23_P129577	TIGD7	0.94	6.50	1.17E-03	8.75E-03	protein_coding
CUST_29938_PI428871386	ENST00000554160.1	1.03	4.91	1.17E-03	8.75E-03	lincRNA
CUST_523_PI428871386	ENST00000417884.1	1.12	5.42	1.17E-03	8.76E-03	lincRNA
CUST_13495_PI428871386	ENST00000501173.2	-0.87	7.96	1.17E-03	8.76E-03	lincRNA
A_33_P3259620	TTC37	-0.84	10.07	1.17E-03	8.77E-03	protein_coding
CUST_19571_PI428871386	ENST00000505564.2	0.82	4.87	1.18E-03	8.81E-03	lincRNA
A_33_P3306068	MYCL1	1.78	8.30	1.18E-03	8.82E-03	protein_coding
CUST_1387_PI428871386	ENST00000436742.1	-0.77	5.11	1.18E-03	8.82E-03	lincRNA
A_33_P3209651	WDFY4	-1.76	8.38	1.18E-03	8.82E-03	protein_coding
CUST_29406_PI428871386	ENST00000320322.2	0.83	4.97	1.18E-03	8.83E-03	lincRNA
A_23_P45025	MAPK10	-1.17	6.25	1.18E-03	8.84E-03	protein_coding
CUST_854_PI428871386	ENST00000417869.1	-1.07	10.72	1.18E-03	8.85E-03	antisense
CUST_29426_PI428871386	ENST00000535351.1	-0.82	4.87	1.19E-03	8.86E-03	antisense
A_33_P3339173	SRP9	0.86	10.12	1.19E-03	8.86E-03	protein_coding
A_24_P100234	MORC2	0.83	6.93	1.19E-03	8.87E-03	protein_coding
CUST_18006_PI428871386	ENST00000480632.1	-1.74	7.30	1.19E-03	8.89E-03	antisense
CUST_14836_PI428871386	ENST00000373170.2	-0.88	7.65	1.19E-03	8.90E-03	antisense
CUST_29972_PI428871386	ENST00000551597.2	-0.86	9.61	1.19E-03	8.91E-03	antisense
CUST_34354_PI428871386	ENST00000568137.1	-0.74	4.88	1.19E-03	8.92E-03	lincRNA
A_33_P3395274	ZADH2	-1.03	9.99	1.20E-03	8.97E-03	protein_coding
A_33_P3211213	TSEN34	0.94	12.34	1.20E-03	8.98E-03	protein_coding
A_23_P23502	OR6N1	-0.80	4.98	1.20E-03	8.98E-03	protein_coding
A_33_P3303649	MB	1.55	5.63	1.21E-03	9.00E-03	protein_coding
A_24_P91566	BMP7	1.38	5.51	1.21E-03	9.02E-03	protein_coding
CUST_13098_PI428871386	ENST00000507963.1	-1.17	7.79	1.21E-03	9.03E-03	antisense
CUST_39936_PI428871386	ENST00000589594.1	0.84	5.41	1.21E-03	9.04E-03	lincRNA
CUST_41871_PI428871386	ENST00000428667.1	0.92	5.39	1.21E-03	9.06E-03	antisense
CUST_13667_PI428871386	ENST00000523154.1	-0.93	5.02	1.22E-03	9.09E-03	lincRNA
A_24_P167984	ATMIN	0.89	8.85	1.22E-03	9.10E-03	protein_coding
CUST_33315_PI428871386	ENST00000577048.1	-0.83	5.30	1.22E-03	9.11E-03	antisense
CUST_28864_PI428871386	ENST00000428761.1	0.77	4.89	1.23E-03	9.15E-03	lincRNA
CUST_14884_PI428871386	ENST00000450671.1	-0.85	6.69	1.23E-03	9.15E-03	antisense
CUST_21127_PI428871386	ENST00000423380.1	-1.11	6.81	1.23E-03	9.15E-03	lincRNA
CUST_6456_PI428871386	ENST00000418968.2	1.27	6.21	1.23E-03	9.15E-03	antisense
CUST_20793_PI428871386	ENST00000429818.1	-1.01	7.66	1.24E-03	9.22E-03	lincRNA
A_33_P3282963	TJP2	-0.94	5.62	1.24E-03	9.23E-03	protein_coding
CUST_15653_PI428871386	ENST00000421310.1	-0.77	4.60	1.24E-03	9.23E-03	lincRNA
A_23_P137046	NYX	1.07	9.27	1.24E-03	9.23E-03	protein_coding
CUST_29625_PI428871386	ENST00000548096.1	0.76	5.04	1.24E-03	9.25E-03	antisense
A_23_P158662	GPS1	0.86	9.70	1.24E-03	9.26E-03	protein_coding
CUST_39705_PI428871386	ENST00000586176.1	0.96	5.41	1.25E-03	9.31E-03	lincRNA
A_33_P3406706	EXOG	-0.87	7.65	1.26E-03	9.34E-03	protein_coding
CUST_20011_PI428871386	ENST00000522414.1	1.12	6.53	1.26E-03	9.34E-03	lincRNA
CUST_41285_PI428871386	ENST00000427794.1	1.23	5.36	1.26E-03	9.35E-03	lincRNA
A_23_P40096	PROC	1.13	5.24	1.26E-03	9.36E-03	protein_coding
CUST_19464_PI428871386	ENST00000517505.1	-0.81	4.85	1.26E-03	9.38E-03	lincRNA
CUST_12566_PI428871386	ENST00000509844.1	-0.80	4.88	1.26E-03	9.39E-03	lincRNA
CUST_42101_PI428871386	ENST00000569966.1	-0.91	5.86	1.27E-03	9.41E-03	lincRNA

CUST_17061_PI428871386	ENST00000420758.1	0.84	4.98	1.27E-03	9.43E-03	lincRNA
CUST_7510_PI428871386	ENST00000418218.1	-0.84	5.16	1.27E-03	9.43E-03	antisense
CUST_25618_PI428871386	ENST00000357590.5	1.01	10.26	1.27E-03	9.46E-03	protein_coding
CUST_26879_PI428871386	ENST00000439545.1	0.89	4.98	1.27E-03	9.46E-03	antisense
A_32_P91250	UBE2L3	1.01	10.67	1.27E-03	9.47E-03	protein_coding
A_33_P3213910	ARHGAP30	-1.00	7.26	1.28E-03	9.48E-03	protein_coding
CUST_9582_PI428871386	ENST00000467313.1	1.45	5.33	1.28E-03	9.50E-03	lincRNA
CUST_17715_PI428871386	ENST00000431189.1	-0.76	5.83	1.28E-03	9.52E-03	antisense
A_33_P3272140	NUP35	0.93	8.39	1.28E-03	9.52E-03	protein_coding
CUST_5747_PI428871386	ENST00000443123.1	0.78	4.87	1.28E-03	9.52E-03	lincRNA
CUST_27255_PI428871386	ENST00000515416.2	1.11	5.09	1.28E-03	9.54E-03	lincRNA
CUST_31228_PI428871386	ENST00000561999.1	-0.80	7.76	1.29E-03	9.54E-03	lincRNA
CUST_28692_PI428871386	ENST00000433070.1	0.99	6.39	1.29E-03	9.54E-03	antisense
CUST_41480_PI428871386	ENST00000446301.1	0.79	5.33	1.29E-03	9.55E-03	antisense
CUST_35024_PI428871386	ENST00000571138.1	1.08	7.54	1.29E-03	9.55E-03	antisense
A_23_P3979	MRPS7	1.02	9.16	1.29E-03	9.57E-03	protein_coding
CUST_21811_PI428871386	ENST00000447221.1	0.92	6.40	1.29E-03	9.59E-03	antisense
CUST_2796_PI428871386	ENST00000416416.1	1.05	5.35	1.29E-03	9.59E-03	antisense
A_33_P3252989	KIAA0913	-0.91	10.94	1.30E-03	9.62E-03	protein_coding
CUST_8277_PI428871386	ENST00000464125.1	1.09	5.96	1.30E-03	9.64E-03	antisense
CUST_31519_PI428871386	ENST00000313807.4	-0.81	5.72	1.30E-03	9.65E-03	lincRNA
CUST_13651_PI428871386	ENST00000504413.1	-0.78	5.18	1.30E-03	9.66E-03	antisense
A_24_P107897	TXNDC11	-1.12	10.42	1.30E-03	9.66E-03	protein_coding
A_33_P3276207	FAM55C	-0.96	7.06	1.30E-03	9.67E-03	protein_coding
CUST_31326_PI428871386	ENST00000561058.1	0.91	5.29	1.30E-03	9.67E-03	lincRNA
A_23_P345081	ZNF655	-1.13	9.66	1.31E-03	9.68E-03	protein_coding
A_33_P3417437	CD164L2	-1.30	8.36	1.31E-03	9.68E-03	protein_coding
CUST_28555_PI428871386	ENST00000585327.1	-0.80	5.04	1.31E-03	9.69E-03	lincRNA
A_24_P53282	CPD	1.28	7.78	1.31E-03	9.69E-03	protein_coding
CUST_37272_PI428871386	ENST00000573207.1	1.02	5.87	1.31E-03	9.69E-03	antisense
CUST_10791_PI428871386	ENST00000511271.1	1.11	7.25	1.31E-03	9.70E-03	antisense
A_33_P3251538	MAPKAP1	0.86	7.65	1.31E-03	9.70E-03	protein_coding
A_33_P3305254	PSMD5	0.85	6.68	1.31E-03	9.71E-03	protein_coding
CUST_38831_PI428871386	ENST00000344893.3	-0.97	5.41	1.32E-03	9.74E-03	lincRNA
CUST_14229_PI428871386	ENST00000519603.1	0.75	10.85	1.32E-03	9.79E-03	lincRNA
CUST_6419_PI428871386	ENST00000442666.1	1.33	5.34	1.32E-03	9.79E-03	antisense
CUST_28562_PI428871386	ENST00000591799.1	-0.87	4.76	1.32E-03	9.80E-03	lincRNA
CUST_36219_PI428871386	ENST00000591950.1	-0.92	7.72	1.32E-03	9.80E-03	lincRNA
CUST_7694_PI428871386	ENST00000466431.2	0.82	5.86	1.33E-03	9.82E-03	antisense
CUST_6405_PI428871386	ENST00000442706.1	1.05	5.28	1.33E-03	9.83E-03	lincRNA
CUST_9576_PI428871386	ENST00000600750.1	0.80	5.11	1.33E-03	9.83E-03	lincRNA
A_33_P3612589	ATRX	-0.87	10.19	1.33E-03	9.86E-03	protein_coding
CUST_14715_PI428871386	ENST00000461287.1	-0.92	11.21	1.33E-03	9.86E-03	protein_coding
A_33_P3270776	HTRA3	1.29	7.60	1.33E-03	9.86E-03	protein_coding
A_33_P3356492	ENST00000451257	1.14	5.62	1.33E-03	9.86E-03	lincRNA
A_33_P3419012	DLG2	-0.82	4.85	1.34E-03	9.87E-03	protein_coding
CUST_9719_PI428871386	ENST00000426459.1	-0.95	5.29	1.34E-03	9.87E-03	antisense
A_33_P3300975	HOXC4	1.41	7.44	1.34E-03	9.91E-03	protein_coding
CUST_16096_PI428871386	ENST00000444219.1	-0.74	5.03	1.34E-03	9.91E-03	lincRNA
A_23_P82748	ENY2	0.96	12.19	1.34E-03	9.92E-03	protein_coding
CUST_1478_PI428871386	ENST00000528692.1	-0.99	7.00	1.34E-03	9.92E-03	antisense
CUST_740_PI428871386	ENST00000581333.1	-0.77	5.45	1.34E-03	9.92E-03	antisense
A_23_P333138	MON2	-0.83	5.91	1.34E-03	9.92E-03	protein_coding
A_23_P68922	MICALL1	1.02	6.62	1.35E-03	9.95E-03	protein_coding
CUST_11369_PI428871386	ENST00000499587.2	-0.85	5.21	1.35E-03	9.95E-03	antisense
CUST_16546_PI428871386	ENST00000430548.1	-0.76	4.97	1.35E-03	9.96E-03	lincRNA

Supplementary Table S6. Comparison of clinical characteristics of technical and biological validation sets subject to RT-qPCR.

	Technical set	Validation set	Total
N	29	38	67
**Age**			
mean (s.d.)	66.4 (8.5)	67.6 (8.4)	67.1 (8.4)
**Gender**			
Male : Female	21 : 8	29 : 9	50 : 17
**Histology**			
Adenocarcinoma	14	12	26
Squamous cell carcinoma	15	24	39
Other	-	2	2
**Tumour stage**			
T1	1	4	5
T2	21	30	51
T3	5	3	8
T4	2	1	3
**Nodal stage**			
N0	15	19	34
N1	9	12	21
N2	5	7	12
**Differentiation**			
Well	3	4	7
Moderate	17	19	36
Poor	9	11	20
Missing	-	4	4

Supplementary Figure S1

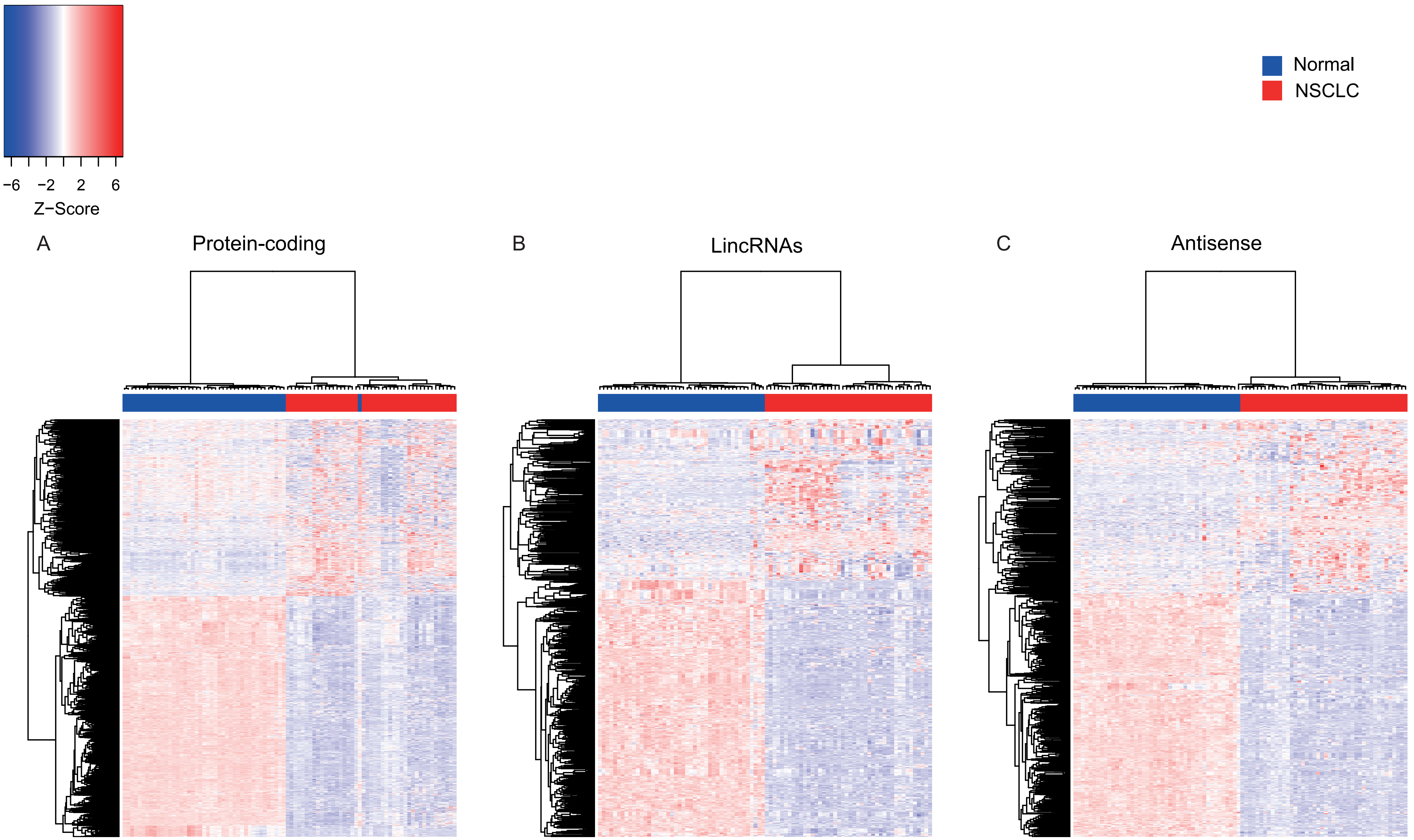

Supplementary Figure S2

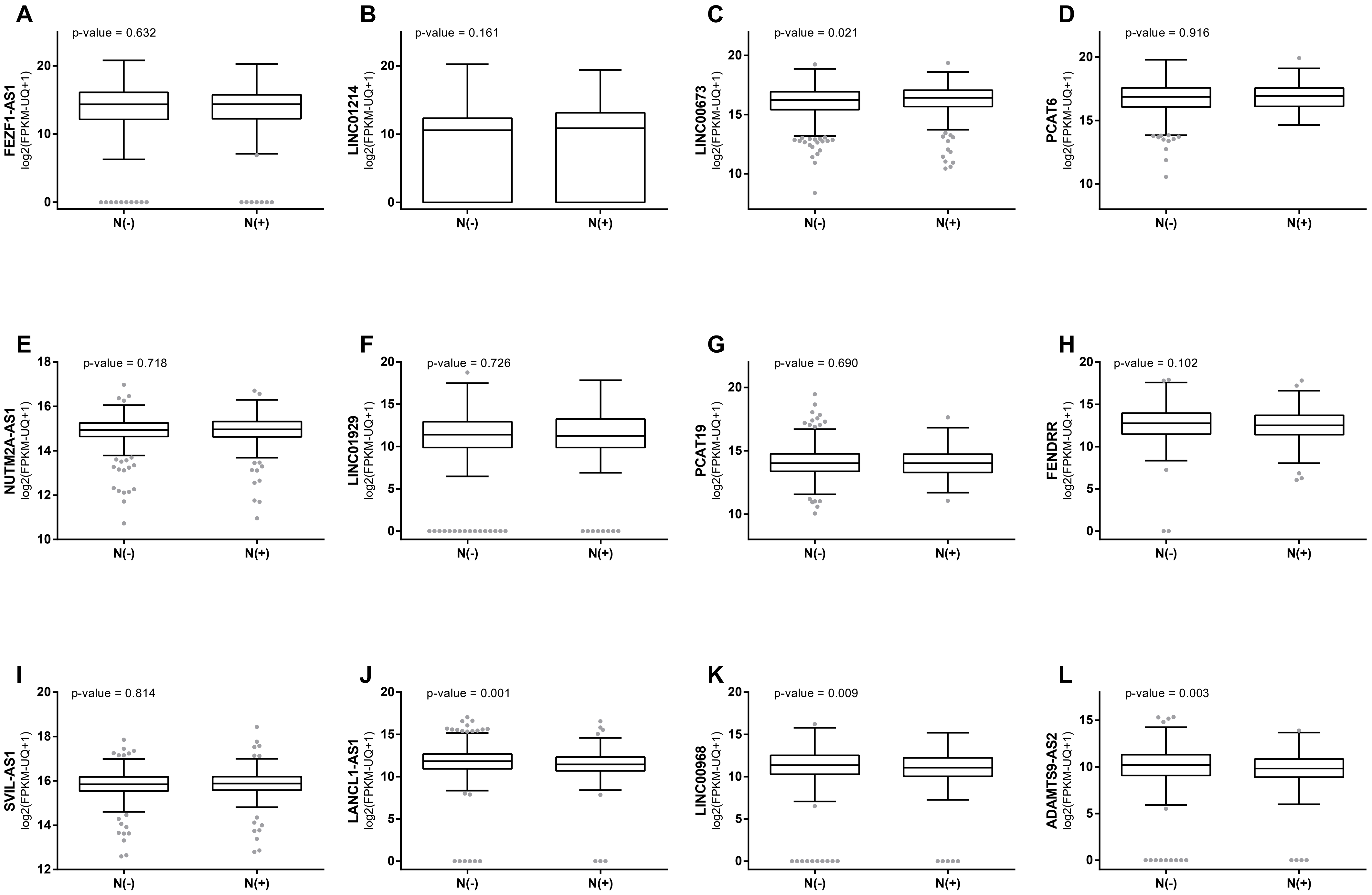

Supplementary Figure S3

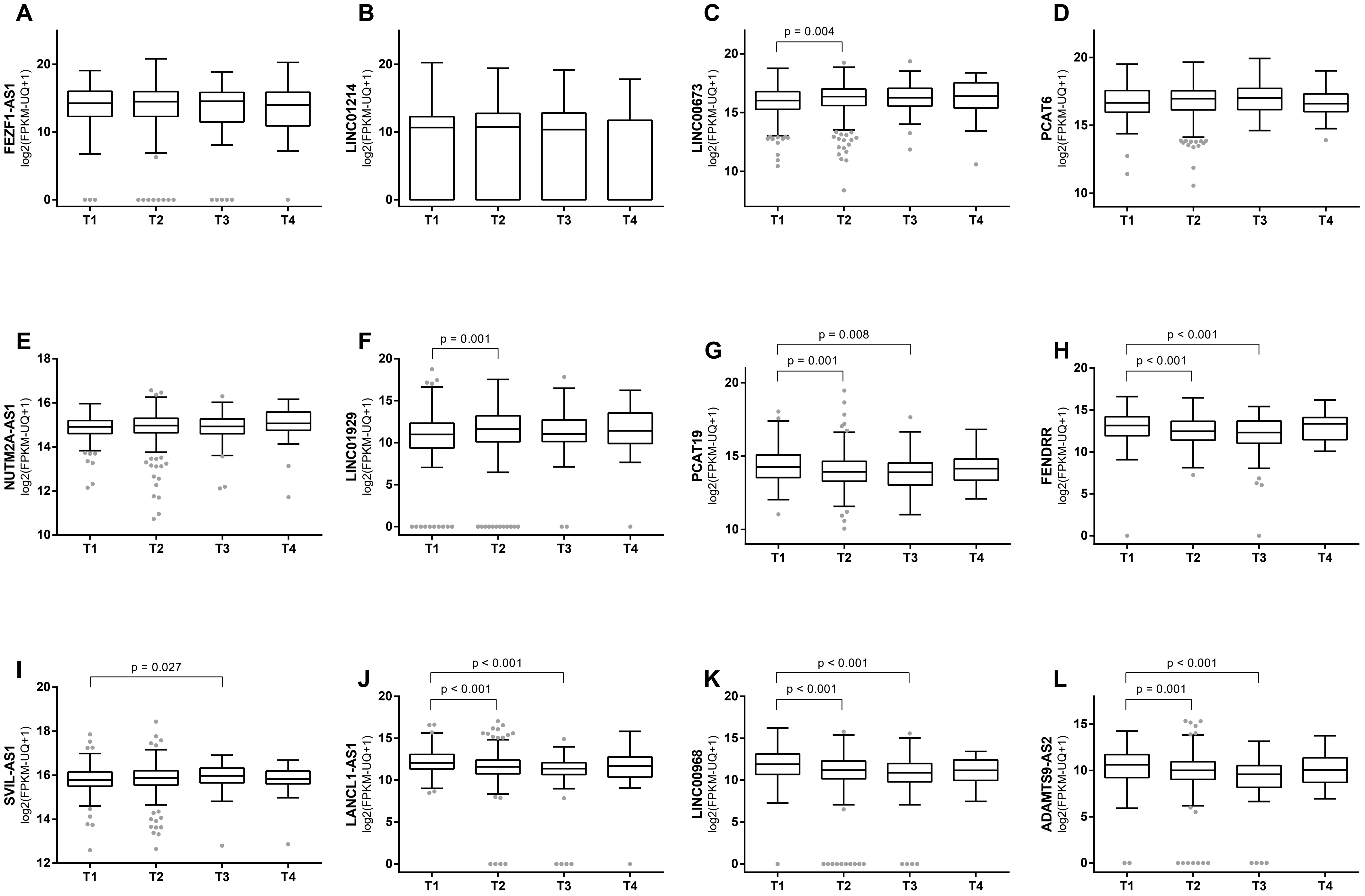

Supplementary Figure S4

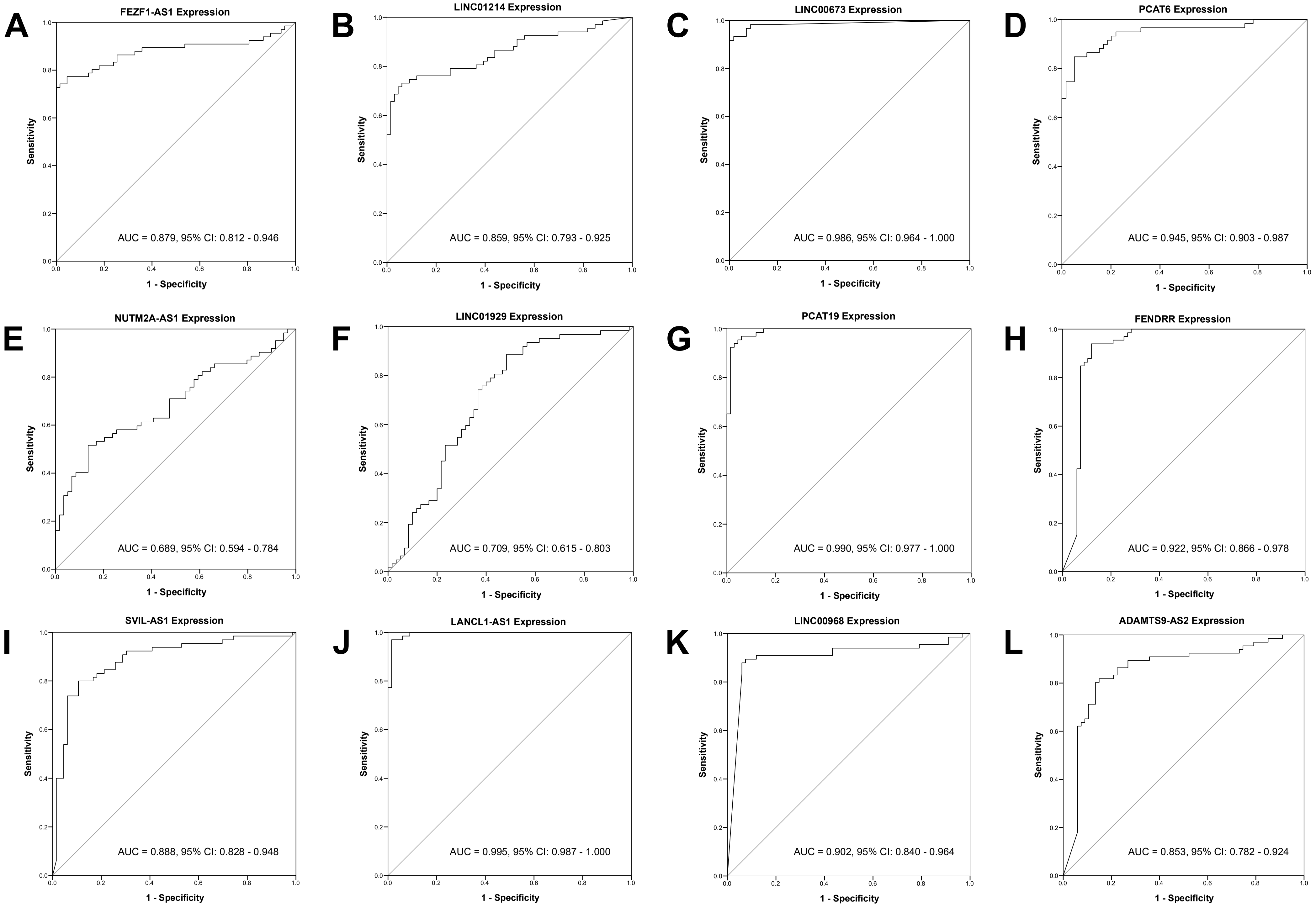

Supplementary Figure S5

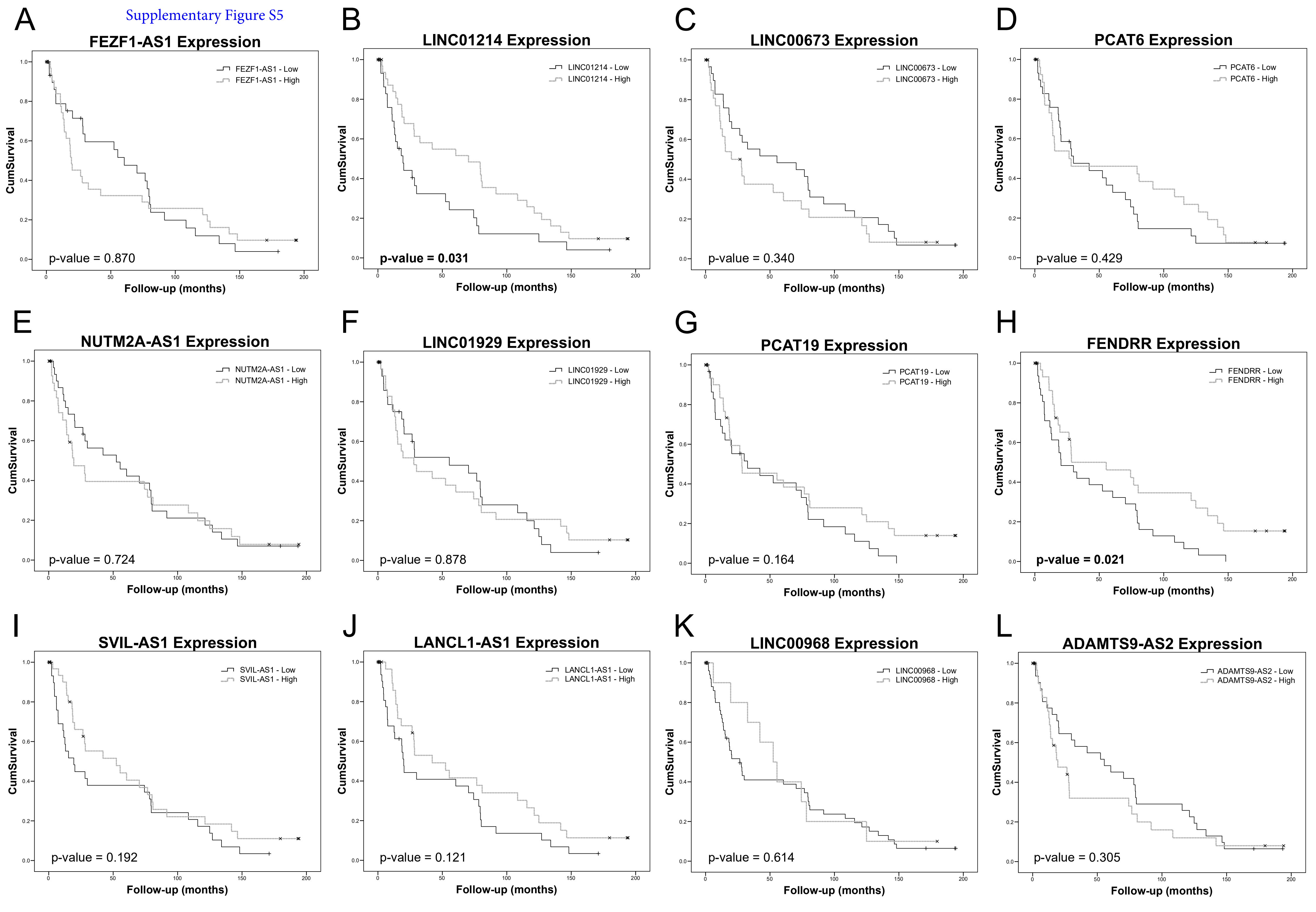

Supplementary Figure S6

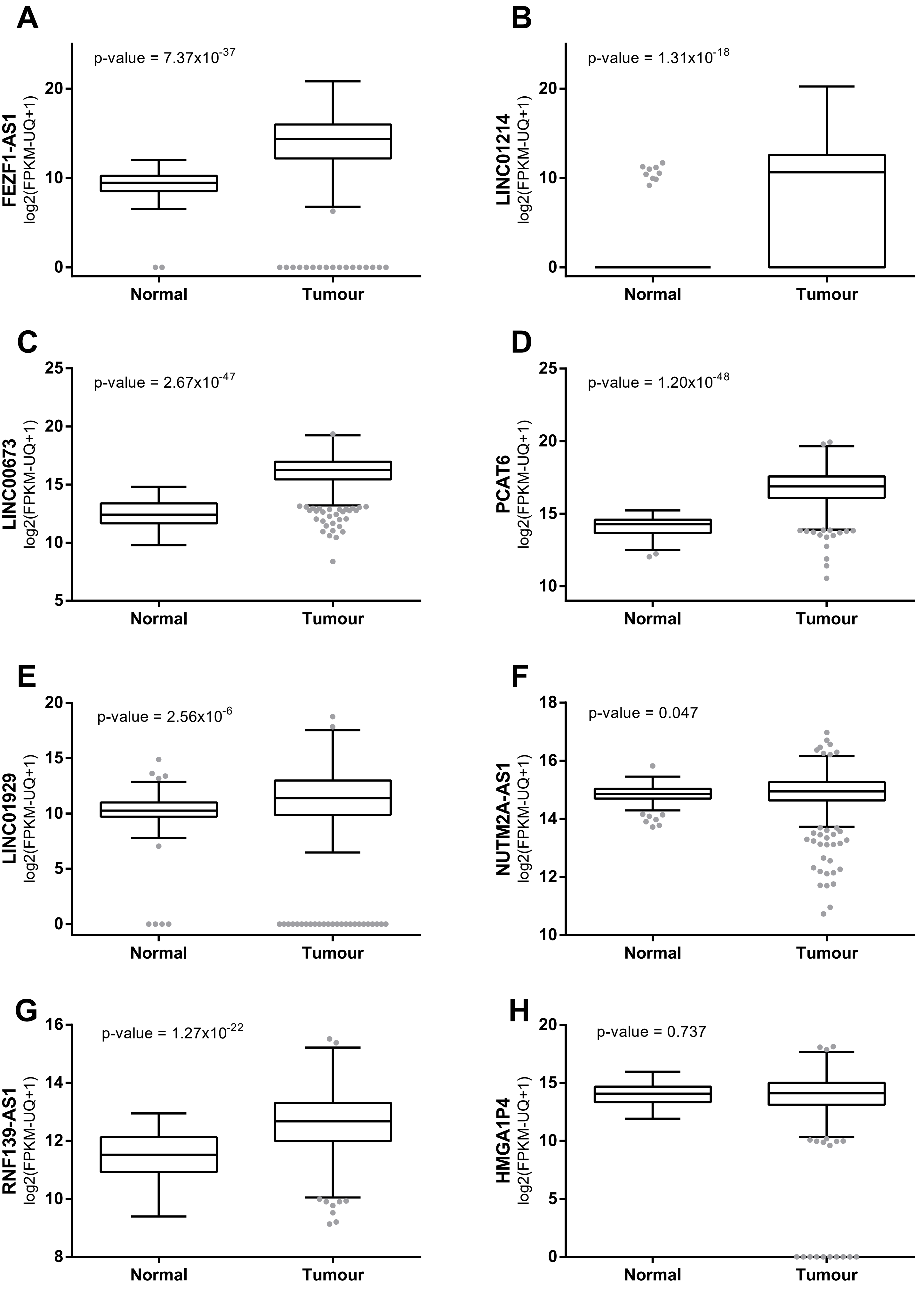

Supplementary Figure S7

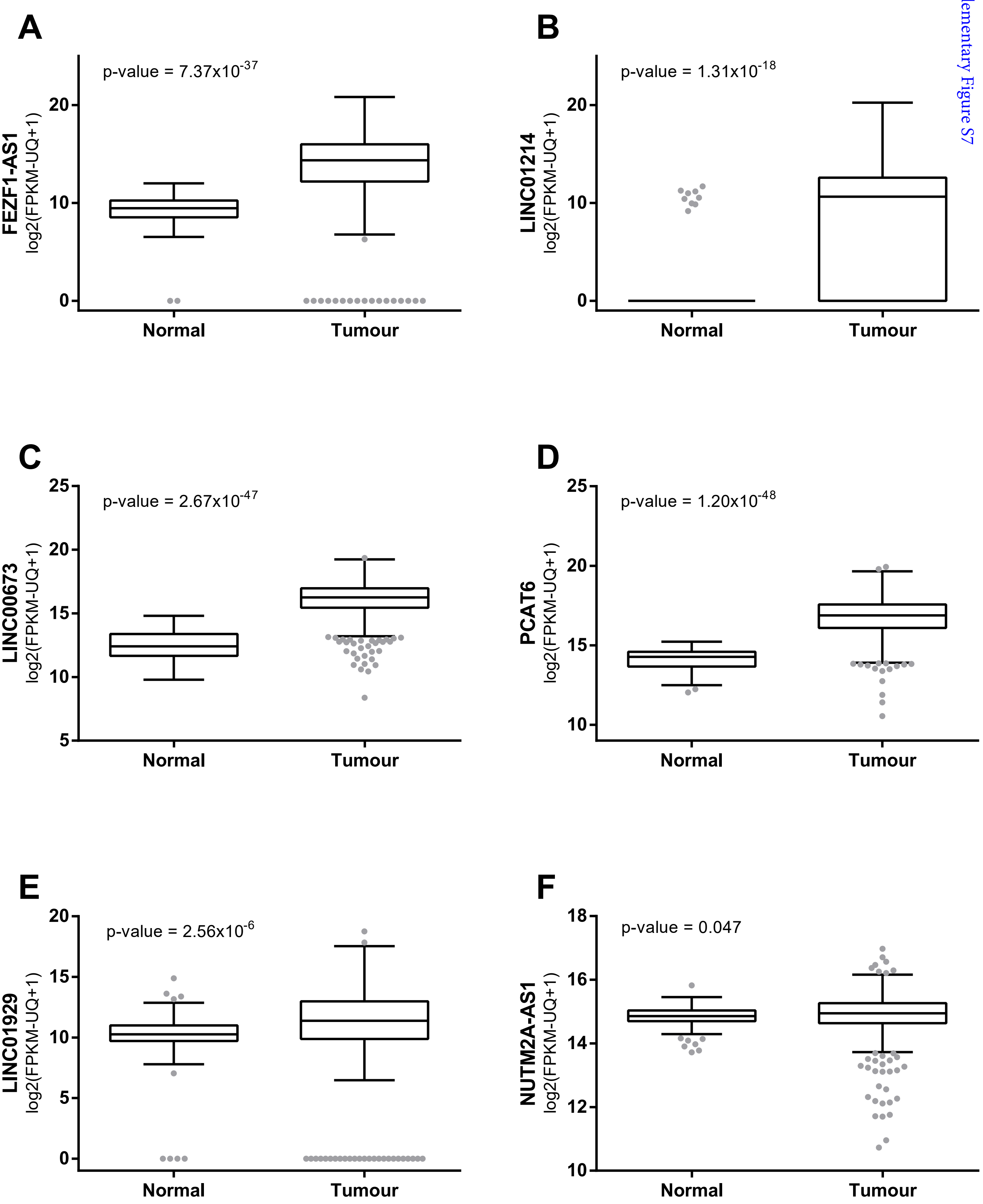

Supplementary Figure S8

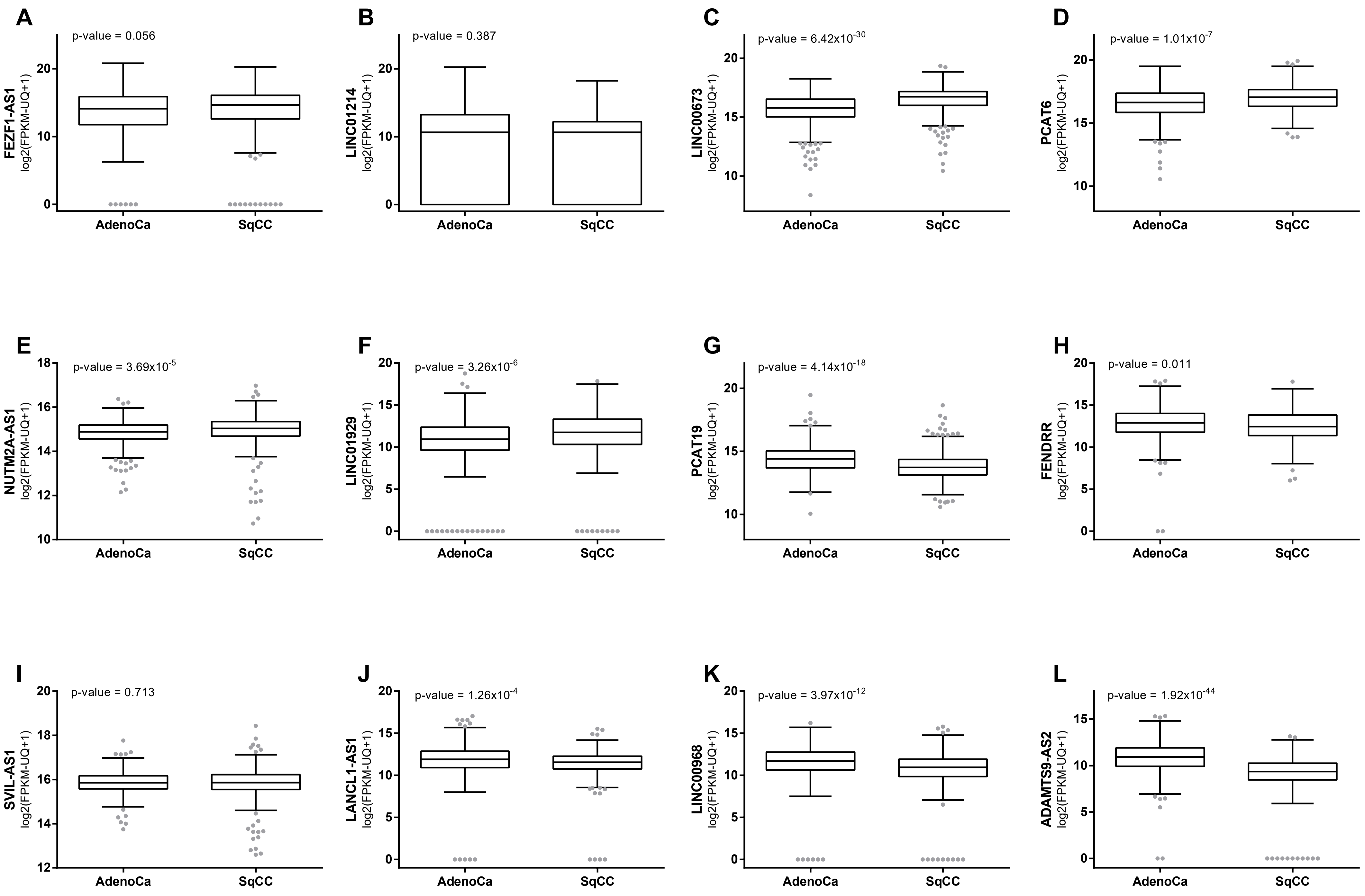

Supplementary Figure S9

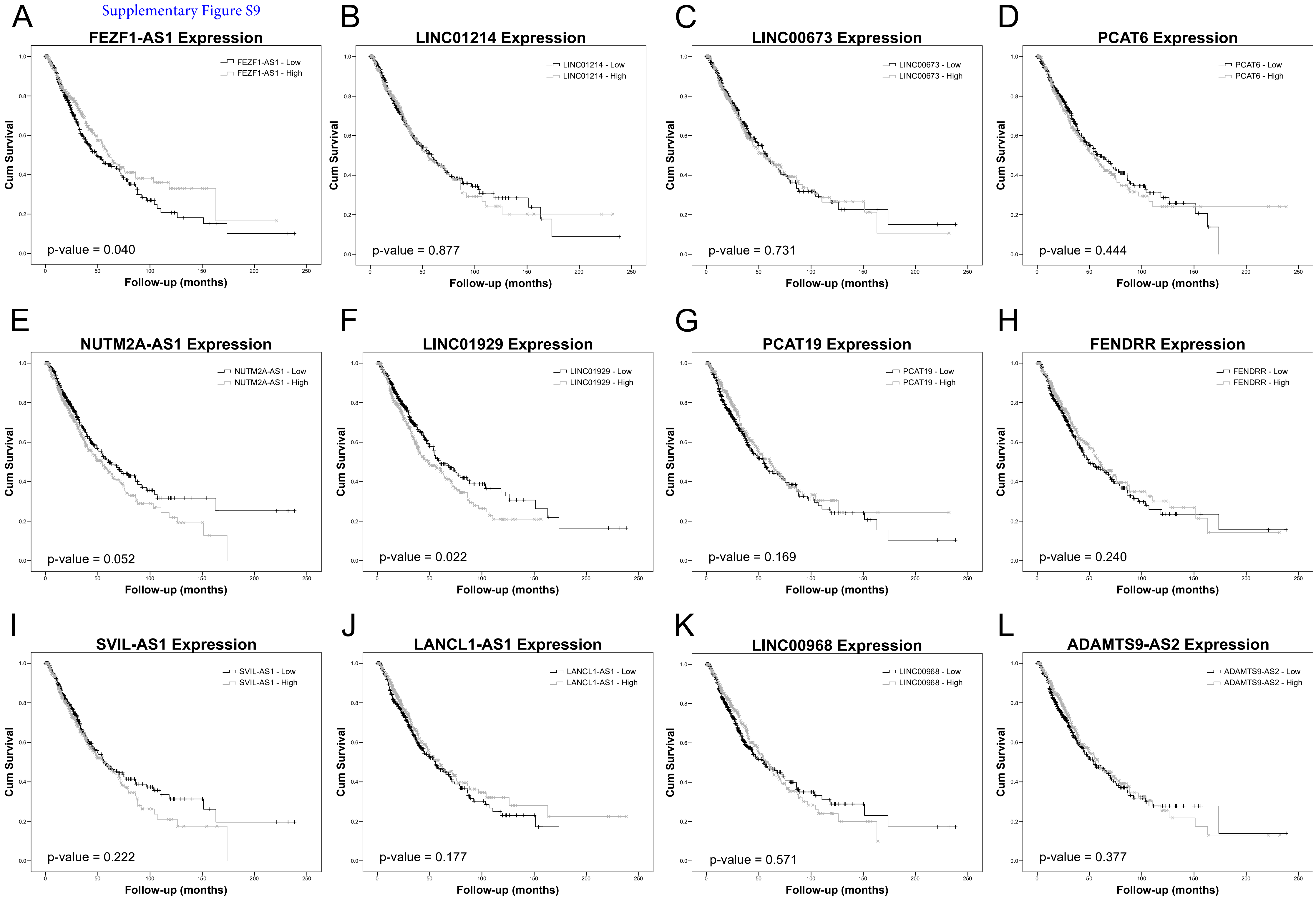

Supplement: Supplementary file 1 — Supplementary Information [file 41416_2020_742_MOESM1_ESM.pdf]
